# Mechanism of electro-acupuncture in alleviating intestinal injury in septic mice via polyamine-related M2-macrophage polarization

**DOI:** 10.3389/fimmu.2024.1373876

**Published:** 2024-04-22

**Authors:** Xinyi Xu, Xianglong Huang, Lu Xiao, Jiabao Wang, Xiaokun Yang, Yifan Wu

**Affiliations:** ^1^ Department of Emergency, First Teaching Hospital of Tianjin University of Traditional Chinese Medicine, Tianjin, China; ^2^ Department of Emergency, National Clinical Research Center for Chinese Medicine Acupuncture and Moxibustion, Tianjin, China; ^3^ Institute of Traditional Chinese Medicine, Tianjin University of Traditional Chinese Medicine, Tianjin, China

**Keywords:** electro-acupuncture, sepsis, intestinal injury, macrophage polarization, spermidine

## Abstract

**Objective:**

The objective of this study was to investigate the impact of electro-acupuncture (EA) on sepsis-related intestinal injury and its relationship with macrophage polarization.

**Methods:**

A sepsis model was established using cecal ligation and puncture (CLP) to assess the effectiveness of EA. The extent of pathological injury was evaluated using Chiu’s score, the expression of ZO-1 and Ocludin, and the impact on macrophage polarization was examined through flow cytometry and immunofluorescence staining. The expression of spermidine, one type of polyamine, and ornithine decarboxylase (ODC) was measured using ELISA and PCR. Once the efficacy was determined, a polyamine depletion model was created, and the role of polyamines was reassessed by evaluating efficacy and observing macrophage polarization.

**Results:**

EA treatment reduced the Chiu’s score and increased the expression of ZO-1 and Ocludin in the intestinal tissue of septic mice. It inhibited the secretion of IL-1β and TNF-α, promoted the polarization of M2-type macrophages, increased the secretion of IL-10, and upregulated the expression of Arg-1, spermidine, and ODC. However, after depleting polyamines, the beneficial effects of EA on alleviating intestinal tissue damage and modulating macrophage polarization disappeared.

**Conclusion:**

The mechanism underlying the alleviation of intestinal injury associated with CLP-induced sepsis by EA involves with the promotion of M2-type macrophage polarization mediated by spermidine expression.

## Graphical Abstract

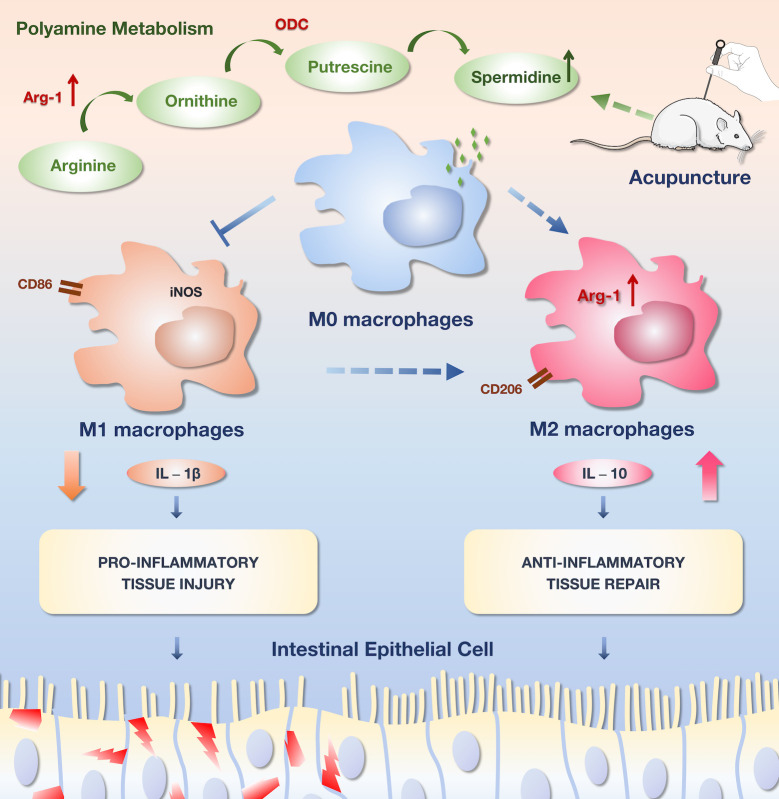

## 1 Introduction

Sepsis is a critical medical condition characterized by severe infection, which can lead to organ damage and an imbalanced immune response (1, 2). It is a leading cause of ICU admissions and mortality. The dysregulation of immune responses and multiple organ dysfunction associated with sepsis pose a significant risk and challenge to patient outcomes (3, 4). According to a global survey conducted in 2017, there are approximately 49 million cases of sepsis worldwide, resulting in about 11 million deaths (5). The incidence of sepsis continues to rise, posing a serious threat to human life and well-being.

The gastrointestinal tract plays a central role in the progression of sepsis and multiple organ dysfunction syndrome (MODS) (6, 7). Severe inflammation in the gastrointestinal tract affects the prognosis of septic patients. During severe infection, the body’s metabolic rate and oxygen consumption increase significantly (8, 9). As a result, blood flow is often redirected to vital organs such as the brain, lungs, and heart, which can cause ischemia, hypoxia, and reperfusion injury in the intestinal mucosa. During this process, a large amount of oxygen free radicals are produced, which can disrupt the formation and distribution of tight junctions between cells and cause damage to the intestinal epithelium (10–12). Simultaneously, the compromised intestinal microenvironment, weakened intestinal motility, and inadequate intestinal nutrition result in intestinal barrier dysfunction and increased permeability (13, 14). This allows pathogenic microorganisms and endotoxins to enter the circulatory and lymphatic systems, ultimately reaching distant organs and causing gut-origin sepsis (GOS) and MODS. These pathological processes create a vicious cycle characterized by imbalances in microbial, inflammatory, endotoxin, and intestinal homeostasis, contributing to the progression of sepsis (2, 7, 15).

Acupuncture is indeed a prominent and widely used modality in traditional Chinese medicine. It involves the insertion of thin needles into specific points on the body known as acupoints. By carefully diagnosing the patient’s condition and selecting appropriate acupoints, acupuncture offers several advantages, including safety, convenience, and remarkable efficacy (16, 17). In recent years, acupuncture has been widely applied in the treatment of gastrointestinal dysfunction caused by sepsis and has shown positive outcomes. Studies have demonstrated that acupuncture at specific acupoints can significantly improve gastrointestinal symptoms, enhance gastrointestinal motility, and reduce intra-abdominal pressure, intestinal rumbling frequency, and gastrointestinal dysfunction scores in patients (18–20). Additionally, acupuncture can decrease the levels of serum inflammatory markers such as C-reactive protein (CRP), procalcitonin (PCT), tumor necrosis factor-alpha (TNF-α), interleukin (IL)-6, IL-8, and IL-1β, while promoting the release of anti-inflammatory factors like IL-10 (16, 21, 22). However, the underlying mechanisms of acupuncture for the treatment of gastrointestinal dysfunction in sepsis are not yet fully understood.

Macrophages play critical roles in both innate and adaptive immunity. They effectively combat pathogen invasion and tissue damage through pattern recognition mechanisms. Macrophages exhibit plasticity, recruiting other immune cells to the infection site via the secretion of chemokines and inflammatory factors during pathogen invasion, thereby inducing a robust inflammatory response to eliminate the source of infection. Furthermore, macrophages contribute to tissue repair by secreting anti-inflammatory factors and growth factors during the later stages of injury. Thus, their primary function can be understood as maintaining immune homeostasis (23–25).

The plasticity of macrophages in infectious diseases underscores their significant role in intestinal inflammation and mucosal repair (26). Recent studies indicated that polyamines, important regulatory substances involved in cell proliferation and differentiation, play a crucial role in macrophage polarization and function (27, 28). Therefore, this study aims to explore the therapeutic effects of electro-acupuncture (EA) on mice with CLP-induced sepsis, focusing on spermidine expression and macrophage polarization. Furthermore, difluoromethylornithine (DFMO) will be employed to conduct further investigations into the correlation between polyamines and macrophage polarization in the context of EA therapy for sepsis-related gastrointestinal dysfunction.

## 2 Materials and methods

### 2.1 Ethics statement

All experiments and surgical procedures conducted in this study were approved by the Animal Care and Use Committee of the First Teaching Hospital of Tianjin University of Traditional Chinese Medicine.

### 2.2 Animal

C57BL/6 male mice (6-8 weeks old, weighing 20-25g) were obtained from SpePharm (Beijing) Biotechnology Co., Ltd (Beijing, China) with the Animal license number: SCXK (Jing) 2019-0010. The mice were housed individually in a standard animal care room under controlled conditions, including a 12-hour light-dark cycle, a temperature of 24 ± 2°C, daily cleaning, and ad libitum access to food and water. Prior to the formal experimental study, the mice were acclimated to the housing environment for one week.

### 2.3 CLP surgery

The mice were subjected to cecal ligation and puncture (CLP) surgery, which is the gold standard model for studying sepsis, or sham surgery as a control group (the CON group). After a 12-hour fasting period, the mice were anesthetized with 3% isoflurane and underwent a 2-cm midline laparotomy to expose the cecum and adjacent intestine. In the CLP group, the cecum was tightly ligated with a 3.0 silk suture at 50% below the ileocecal valve and a 0.8-1.0cm point from the cecal tip and punctured once with an 18-gauge needle to induce mid-grade sepsis. A small amount of feces was extruded through the puncture site by squeezing the cecum. The cecum was then returned to the peritoneal cavity, and the laparotomy was closed with 3.0 silk sutures. In the control group, all surgical procedures were performed except for the ligation and puncture of the cecum. Both groups received necessary support (1ml of NaCl 0.9%, subcutaneously) shortly after the CLP and sham surgery procedures.

### 2.4 Electro-acupuncture intervention

**Figure 1:**
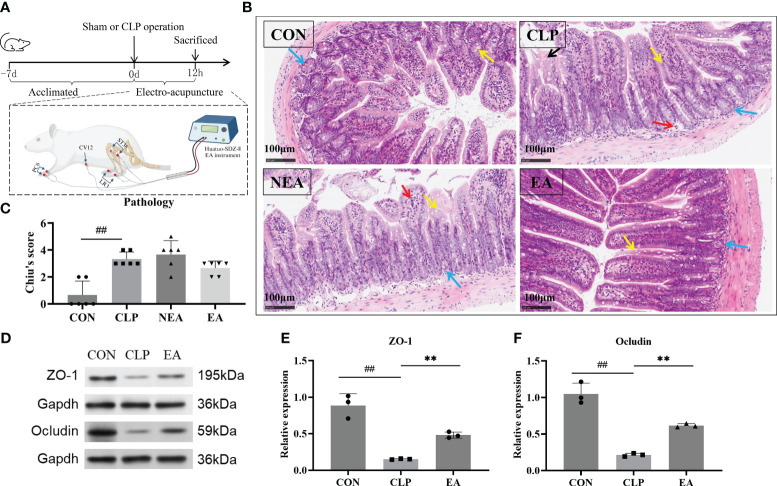
EA alleviates intestinal injury and regulates polyamine metabolism. **(A)** Flow chart of the experiment. **(B)** Immunohistochemical staining of intestine. (Black arrow: mucosal epithelial injury; Yellow arrow: Goblet cell; Blue arrow: Paneth cell; Red arrow: Inflammatory cell) **(C)** Chiu’s score of intestinal injury. **(D)** Western blotting of ZO-1 and Ocludin in intestine. **(E, F)** Relative expression of ZO-1 and Ocludin. All results presented as mean ± SD. Compared with the CLP group, ** means p < 0.01. Compared with the CON group, ## means p < 0.01.

Prior to the EA stimulation intervention, the mice were anesthetized with isoflurane and placed on a warming pad to maintain their body temperature. According to the T/CAAM 0002-2020 document titled “Name and Location of commonly used acupoints in experimental animals - Part 3: Mice”, the acupoints of Neiguan (PC6), Zusanli (ST36), Zhongwan (CV12), and Taichong (LR3) were chosen for EA in our study. PC6 is located bilaterally on the medial side of the forearm, between the radial and ulnar bones, at approximately 2 mm from the wrist joint. Acupuncture needles are typically inserted to a depth of 2 mm at this point. ST36 is located bilaterally posterolateral to the knee, approximately 2 mm below the fibula head, with acupuncture needles inserted to a depth of 3 mm. CV12 is located at the midpoint of the line between the umbilicus and the tip of the xiphoid process, with acupuncture needles inserted to a depth of 2 mm. LR3 is located in the depression between the first and second metatarsals of the dorsum of the hind limb, with acupuncture needles inserted to a depth of 1 mm (**Figure 1A**). Electrical stimulation was performed using an EA instrument (Huatuo-SDZ-II, Suzhou, China) with a current intensity of 2 mA, a frequency of 5/20 Hz, and a stimulation time of 30 minutes. At the same time, we selected non-meridian points electrical stimulation (the NEA group) as the comparison object of EA. The electrical stimulation of acupoints and non-meridian points was applied immediately after CLP.

### 2.5 Polyamine depletion

DFMO is a specific, irreversible inhibitor of the enzyme ornithine decarboxylase (ODC). According to published literature, mice were administered DFMO (MedChemExpress; Cat# 103461) to induce polyamine depletion by providing it in their drinking water for a duration of 7 days (29). Subsequently, a subset of the polyamine-depleted mice was randomly selected to establish a model of CLP-induced sepsis (the DFMO group), followed by electro-acupuncture intervention (the DEA group). Twelve hours after CLP surgery, all groups of mice were sampled for further testing.

### 2.6 Histopathological analysis

Mice’s intestinal tissue samples were collected and rinsed with PBS buffer (Solarbio; Cat# P1022). The samples were then processed for routine paraffin embedding and slicing them in 3 μm. Hematoxylin and eosin (H&E) staining was performed on those tissue slices. Chiu’s histological grading system, also known as Chiu’s score, was used to assess the severity of intestinal mucosal injury.

### 2.7 Western blot

The proteins from small intestinal tissue samples were extracted using tissue lysate and quantified using the BCA method. Subsequently, 20 μg of each sample was loaded into the SDS-PAGE gel for electrophoresis separation. After transfer to the membrane, the membrane was incubated with blocking antibody, primary antibodies for ZO-1 and Ocludin (Abcam; ab221547 and ab216327), and secondary antibody for development. Finally, the software of ImageJ 1.53t was used for analysis.

### 2.8 Flow cytometry analysis

**Figure 2:**
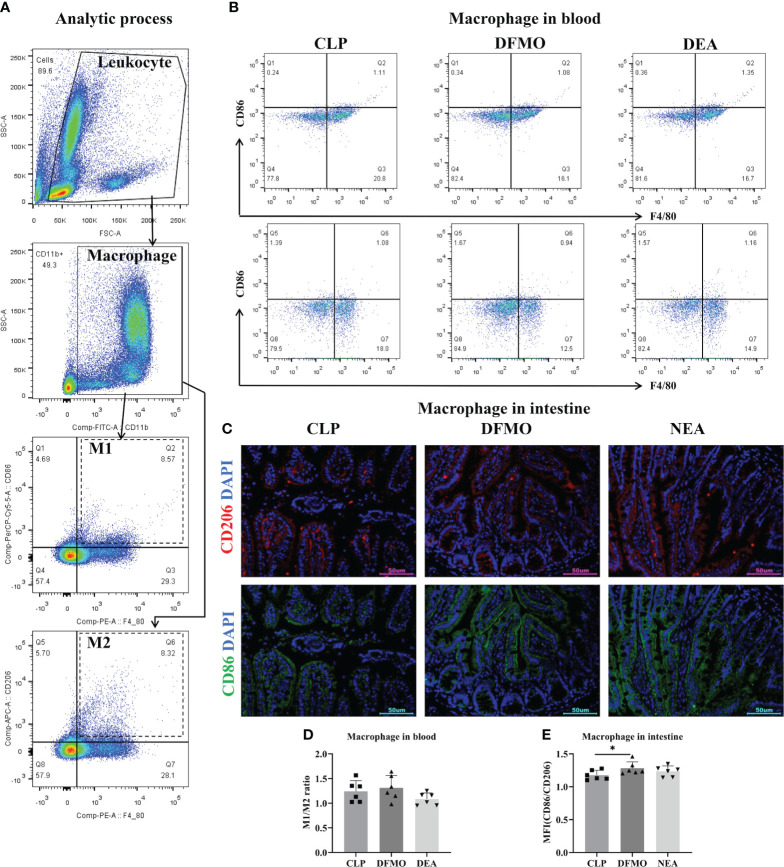
**(A)** Gate setting strategy of flow cytometry. **(B)** Flow cytometry of macrophages in blood. **(C)** Immunofluorescence staining of intestine. **(D)** M1/M2 ratio of macrophage in blood. **(E)** M1/M2 ratio of macrophage in intestine. All results presented as mean ± SD. Compared with the CON group, * means p < 0.05.

After collecting the small intestine tissue, it is processed by grinding and filtering to obtain a single-cell suspension. Similarly, whole blood samples were incubated with red cell lysate for 20 minutes and then suspended followed by washing. Cell pellets from both the small intestine tissue and whole blood are suspended in 100μL of PBS, and 2μL of TruStain FcX™ (Biolegend; Cat# 101319) is added to block the Fc receptor. Next, an antibody cocktail consisting of PE anti-mouse F4/80 (Biolegend; Cat# 111704), FITC anti-mouse CD11b(Biolegend; Cat# 101205), and PerCP anti-mouse CD86(Biolegend; Cat# 105025) was prepared. The antibody cocktail with 100uL PBS is added to the cell pellets, and the tubes are incubated at 4°C for 30 minutes in the dark. Subsequently, the cells were washed with PBS and a Fixation/Permeabilization buffer (eBioscience; Cat# 88-8824-00) was used to fix and permeate the cells. APC anti-mouse CD206 antibody (Biolegend; Cat# 141707) is then added to the cells and incubated at 4°C for 30 minutes in the dark. Finally, the cells were washed again and subjected to Flow cytometry (BD FACSCanto II) and analysis to determine the number and percentage of different subtypes of blood macrophages (**Figure 2A**).

### 2.9 ELISA

To begin with, we isolated the serum and extracted the intestinal protein. These samples were processed in accordance with the ELISA instructions. The procedure can be summarized as follows: Initially, we added the aforementioned samples to the enzyme-labeled plate of spermidine (MEIMIAN; Cat# MM-46605M1), inducible nitric oxide synthase (iNOS, MEIMIAN; Cat# MM-0454M1), arginase-1 (Arg-1, MEIMIAN; Cat# MM-0120M1), IL-1β(MEIMIAN; Cat# MM-0040M1) or IL-10(MEIMIAN; Cat# MM-0176M1), which induced an enzyme immunoreaction. Once the reaction concluded, we introduced TMB Solution and a termination solution. Subsequently, we employed an enzyme-label instrument to measure the optical density (OD) values of each sample.

### 2.10 Immunofluorescence detection

After dewaxing the small intestine sections, the antigen repair process was carried out to ensure optimal staining. Subsequently, a permeating membrane of Triton X-100 (Bioss; Cat# P0096) was applied in a sequential manner. To seal the small intestine sections effectively, a 1% BSA (Bioss; Cat# bs-0292P) solution was utilized. Next, the specific antibodies, namely anti-mouse CD206(Affinity; Cat# DF449) and anti-mouse CD86(Affinity; Cat# DF6332), along with their corresponding secondary antibodies, anti-rabbit IgG (Alexa Fluor^®^ 488 Conjugate; CST; Cat# 4412) and anti-rabbit IgG (CY3 Conjugate; Affinity; Cat# S0011) respectively, were carefully added to the sections. This incubation step was performed in a light-protected environment to prevent any potential light-induced degradation. After the incubation period, the sections were thoroughly cleansed to remove any unbound antibodies. To visualize the nuclei, the sections were counterstained with DAPI, a fluorescent dye that selectively binds to DNA. Finally, the immunofluorescence intensity of CD86 and CD206 was quantitatively analyzed for all images obtained.

### 2.11 RT-PCR

Total RNA was extracted from small intestinal tissues using the TRIzol RNA extraction protocol (Life Technologies Inc.). 1 μg of RNA was extracted and reverse transcribed to obtain the cDNA template. The real-time fluorescent quantitative PCR reaction system was set up, and 40 cycles of real-time detection were performed on the computer to obtain CT values. Subsequently, the relative expression levels were calculated. The primers for the target gene ODC and the internal reference (β-actin) are listed in **Table 1**.

**Table 1:** Primer information.

Gene Name	Primer	Sequence	Size (bp)
β-actin	Forward	AGGTCATCACTATTGGCAACGAG	167
	Reverse	TTGGCATAGAGGTCTTTACGGAT	
ODC	Forward	AGAGACCCAAGCCAGACGAGAAG	151
	Reverse	GAAAGTAGAAGCAGCAGCAACAGTG	

### 2.12 Statistical analysis

The collected data were subjected to statistical analysis using appropriate methods. If the data were normally distributed, a one-way analysis of variance (ANOVA) was employed. However, if the data did not meet the assumptions of normality, a Kruskal-Wallis analysis of variance was utilized instead. For further analysis and comparison between groups, *post hoc* analysis was conducted using the Dunn’s test. The significance level was set at *p<*0.05, indicating statistical significance. All statistical analyses were performed using GraphPad Prism 9.0.

## 3 Results

### 3.1 Electro-acupuncture improves intestinal injury in mice with sepsis

To evaluate the effect of EA on gastrointestinal injury in mice with sepsis, we constructed a mouse model of CLP-induced sepsis. Consistent with the existing studies, the intestinal mucosal layer of septic mice appeared abnormal, with disordered gland arrangement, mucous membrane erosion and shedding, shorten intestinal villi, and a significant infiltration of inflammatory cells in the submucosa (**Figure 1B**). Notably, NEA intervention did not improve intestinal injury, whereas EA treatment effectively reduced mucosal erosion and tissue abscission, decreased the spaces in the submucosal and lamina propria regions, and restored glandular arrangement to some extent. Chiu’s score (**Figure 1C**), which evaluates intestinal injury, showed an increase in mice with CLP compared to the control group, while EA intervention decreased the score. Mechanical injury is one of the primary forms of intestinal damage. Consequently, we investigated the marker proteins ZO-1 and Ocludin associated with mechanical injury. The results revealed a significant reduction in the expressions of ZO-1 and Ocludin in the CLP group (**Figure 1D–F**). However, EA (presumably a treatment or intervention) effectively increased their expressions. Therefore, EA can alleviate sepsis-related intestinal damage.

### 3.2 Electro-acupuncture regulates macrophage polarization in blood and the expression of macrophage-related cytokines

**Figure 3:**
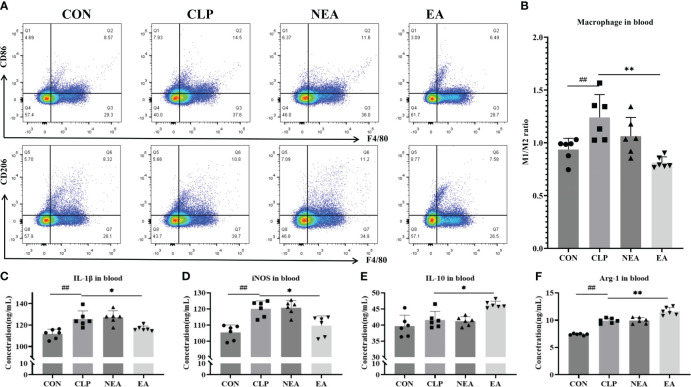
Electro-acupuncture regulates the polarization of macrophages in blood. **(A)** Flow cytometry of macrophages in blood. **(B)** M1/M2 ratio of macrophage. The expression of **(C)** IL-1β, **(D)** iNOS, **(E)** IL-10, and **(F)** ARG in blood. All results presented as mean ± SD. Compared with the CLP group, * means p < 0.05, ** means p < 0.01. Compared with the CON group, ## means p < 0.01.

Sepsis is often associated with polarization of M1 macrophages. Therefore, we observed the polarization of macrophages in the circulatory system. After sampling whole blood, blood cells were labeled with specific antibodies, and the macrophage population labeled with CD11b+F4/80+ was obtained using flow cytometry (**Figure 3A**). We then observed the proportion of CD86-positive cells (M1) and CD206-positive cells (M2) among these macrophages and calculated the M1/M2 macrophage ratio to analyze macrophage polarization characteristics. The results demonstrated that CLP increased the M1/M2 ratio (**Figures 3A, B**) and the expression of M1-related proteins IL-1β and iNOS (**Figures 3C, D**). However, after EA treatment, the M1/M2 ratio significantly decreased, along with a decrease in IL-1β and iNOS expression and an increase in the expression of M2-related proteins IL-10 and Arg-1 (**Figures 3E, F**). These findings indicate that EA promotes the transition of macrophages from the M1 phenotype to the M2 phenotype, reducing inflammation and tissue damage, and facilitating body recovery. We then questioned whether EA regulates macrophage polarization in intestinal tissue.

### 3.3 Electro-acupuncture regulates macrophage polarization in the intestine and the expression of macrophage-related cytokines

**Figure 4:**
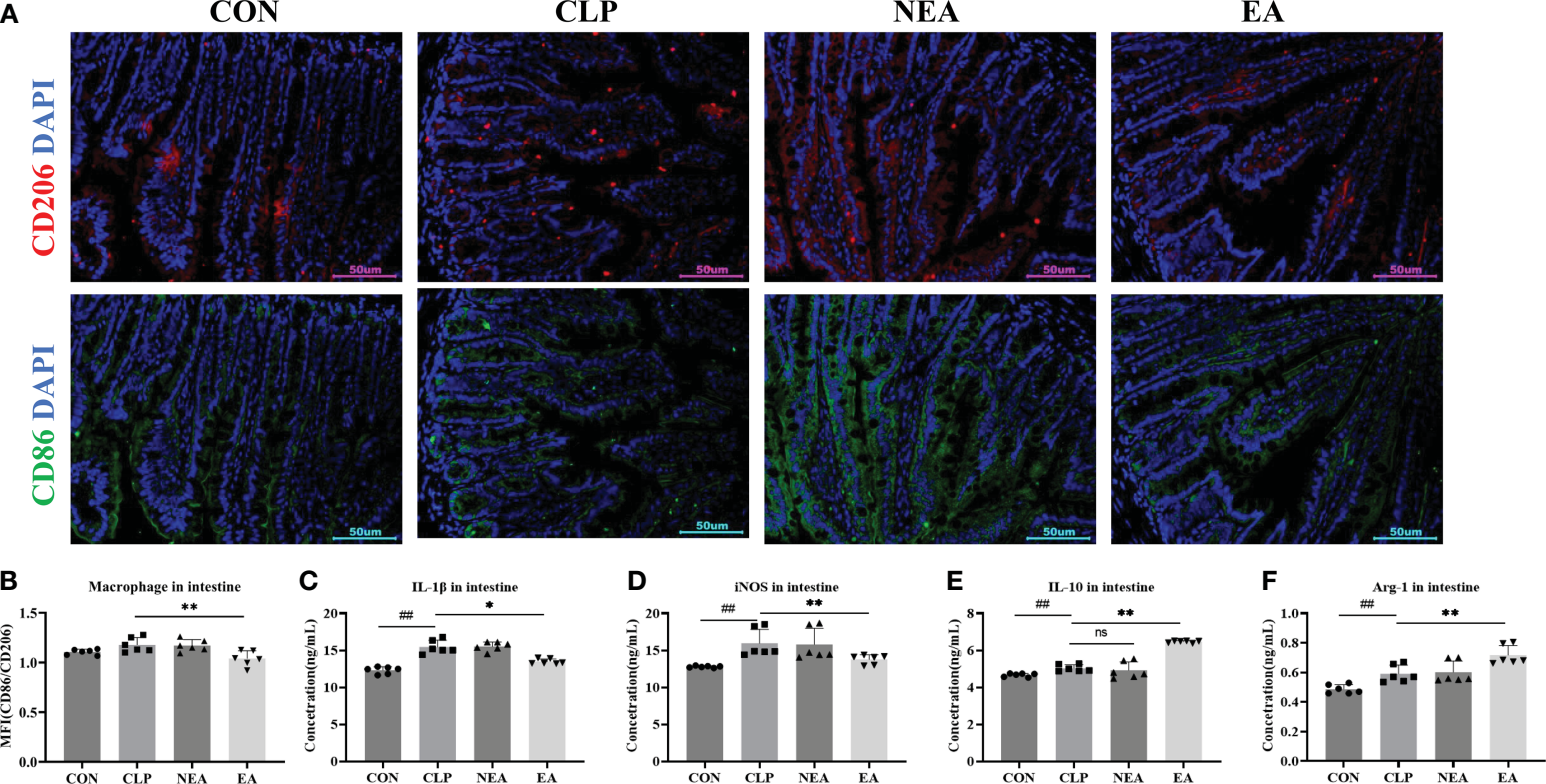
EA regulates the polarization of macrophages in intestine. **(A)** Immunofluorescence staining of intestine. **(B)** Mean fluorescence intensity of CD206/CD86. The expression of **(C)** IL-1β, **(D)** iNOS, **(E)** IL-10, and **(F)** ARG in intestine. All results presented as mean ± SD. Compared with the CLP group, * means p < 0.05, ** means p < 0.01, ns represents no significance. Compared with the CON group, ## means p < 0.01.

To address this question, we collected small intestinal tissue samples from mice and performed immunofluorescence staining. In the small intestine tissue slices of each group, M2 macrophages were labeled with an anti-CD86 antibody, and M2 macrophages were labeled with an anti-CD206 antibody (**Figure 4A**). By detecting the mean fluorescence intensity of CD86 and CD206, we calculated the M1/M2 ratio to determine the polarization of intestinal macrophages (**Figure 4B**). The results showed that CLP had the ability to promote the transformation of macrophages into the M1 phenotype and increase the expression of M1-related molecules IL-1β and iNOS (**Figures 4C, D**). However, after EA treatment, the M1/M2 ratio significantly decreased (**Figure 4B**). Additionally, EA significantly inhibited the expression of IL-1β and iNOS, while promoting the expression of M2-related molecules IL-10 and Arg-1 (**Figures 4E, F**). These findings indicate that EA can regulate the transition of small intestinal macrophages from the M1 phenotype to the M2 phenotype, thereby facilitating tissue repair.

### 3.4 Electro-acupuncture regulates polyamine metabolism

**Figure 5:**
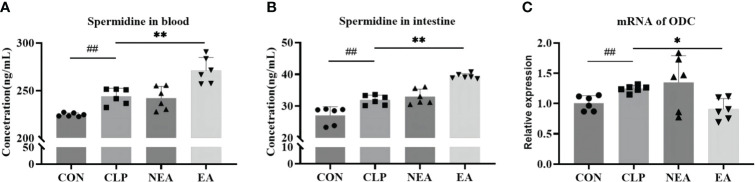
**(A)** Spermidine expression in blood. **(B)** Spermidine expression in intestine. **(C)** The mRNA expression of ODC. All results presented as mean ± SD. Compared with the CLP group, * means p < 0.05, ** means p < 0.01. Compared with the CON group, ## means p < 0.01.

In addition to being a marker protein of M2-type macrophages, Arg-1 can promote the metabolism of arginine to produce ornithine and generate polyamines under the action of ODC, thereby facilitating tissue repair. Therefore, we examined the expression of ODC and the secretion of spermidine. As mentioned earlier, spermidine, a polyamine, is produced by spermidine synthase (SPDS) catalyzing putrescine, which is generated by ODC from ornithine. The experimental results showed that CLP could promote the mRNA expression of ODC in intestinal tissues and increase the expression of spermidine (**Figures 5A–C**). Although EA inhibited the mRNA expression of ODC, it increased the expression of spermidine in blood and intestinal tissues. This sparked our interest in further clarifying the role of polyamines in the improvement of septic intestinal injury through EA.

### 3.5 Electro-acupuncture has no effect on sepsis in mice with depleted polyamines

**Figure 6:**
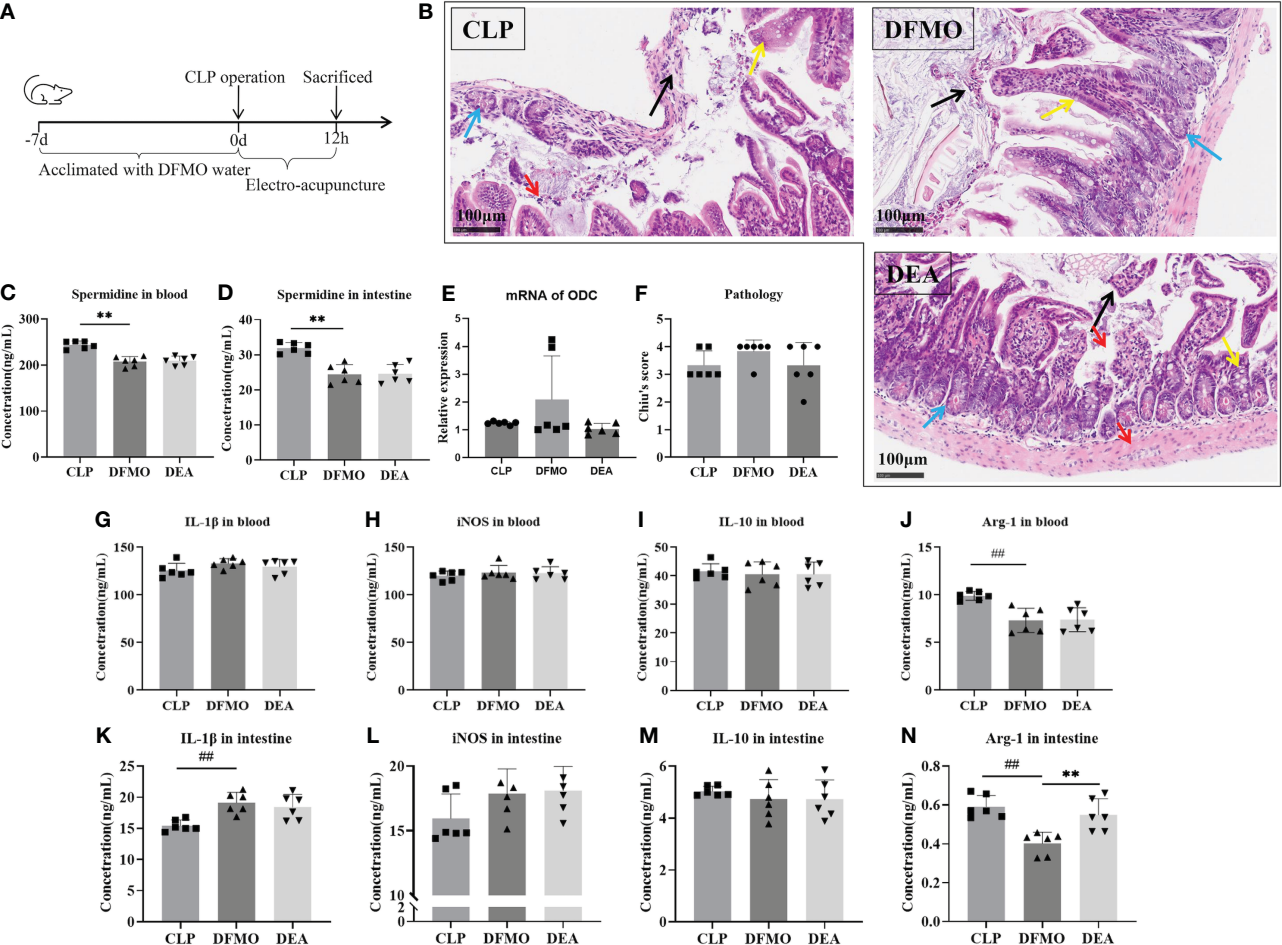
Electro-acupuncture has no effect on septic mouse with polyamine depleted. **(A)** Flow chart of the experiment. **(B)** Immunohistochemical staining of intestine. (Black arrow: mucosal epithelial injury; Yellow arrow: Goblet cell; Blue arrow: Paneth cell; Red arrow: Inflammatory cell). **(C)** Spermidine expression in blood. **(D)** Spermidine expression in intestine. **(E)** The mRNA expression of ODC. **(F)** Chiu’s score of intestinal injury. The expression of **(G)** IL-1β, **(H)** iNOS, **(I)** IL-10, and **(J)** ARG in blood. The expression of **(K)** IL-1β, **(L)** iNOS, **(M)** IL-10, and **(N)** ARG in intestine. All results presented as mean ± SD. Compared with the CLP group, ** means p < 0.01. Compared with the CON group, ## means p < 0.01.

To investigate this, polyamine depletion was induced by administering DFMO water for 7 days prior to the CLP operation, and the relevant indicators of sepsis in mice after 12 hours of EA treatment were observed (**Figure 6A, B, F**). First, by detecting spermidine levels in blood and the small intestine, we found that DFMO caused a decrease of spermidine expression (**Figures 6C–E**). Pathological results showed that DFMO exacerbated intestinal tissue injury to some extent compared to the CLP-induced sepsis group, while EA did not alleviate intestinal injury in polyamine-depleted mice (**Figures 6B, F**). Therefore, we further examined the polarization of macrophages in the circulatory system and intestinal tissues (**Figures 2B, C**). It was found that polyamine depletion increased the expression of IL-1β and the M1/M2 macrophage ratio in the intestine of mice (**Figures 2B–E**, **6G–N**), indicating the importance of spermine in septic mice. However, EA intervention did not improve macrophage polarization (**Figures 2B–E**). In summary, the experimental results indicate that spermine plays a key role in alleviating sepsis in mice, including improving intestinal injury, modulating macrophage polarization, inhibiting the expression of M1-related molecules, and promoting the expression of M2-related molecules.

## 4 Discussion

Macrophages are innate immune cells present in every tissue and critical for innate immunity, normal tissue development, homeostasis, and repair of damaged tissue. Based on the types and functions of cytokines, macrophages can be categorized into two main activation types: classical activation (M1) and alternative activation (M2) (30). In the classical activation pathway, macrophages are stimulated by cytokines such as interferon gamma (IFN-γ) and TNF-α, which activate several signaling pathways such as NF-κB, mitogen-activated protein kinase (MAPK), and signal transducer and activator of transcription 1 (STAT1), leading to their differentiation into M1 macrophages (31, 32). M1 macrophages produce various pro-inflammatory mediators, including TNF-α, IL-1, IL-6, active nitrogen and oxygen intermediates, which exhibit potent bactericidal and tumor-killing activities, give aid to infection clearance and trigger inflammation (33, 34). On the other hand, M2 macrophages are activated by cytokines such as IFN-α/β, IL-4, and IL-13 through the STAT6 pathway (35, 36). M2 macrophages secrete anti-inflammatory mediators (such as IL-10 and TGF-β) and cell growth factors, such as epidermal growth factor (EGF) and vascular endothelial growth factor (VEGF), promoting the suppression of inflammatory responses and facilitating tissue repair (35, 37). The balance between M1 and M2 macrophages reflects the pathological stage of a disease to some extent.

In the human and murine intestinal tissues, there is a significant population of local resident macrophages (38). Sepsis often coincides with gastrointestinal infections, during which the locally stationed macrophages act as the first line to eliminate pathogens of which damage the gastrointestinal tract. Simultaneously, bone marrow-derived monocytes are rapidly recruited from the circulatory system to the intestinal tissues, where they play a more robust role in combating infections (39–41). However, excessive inflammatory stimulation is detrimental to intestinal function recovery. It is well-established that sepsis often leads to an imbalance in the polarization of M1 and M2 macrophages in the intestinal tissue (41). One of the factors contributing to this imbalance is the gastrointestinal ischemia and hypoxia caused by sepsis. In the early stages of sepsis, macrophages become overactivated, resulting in an increase of pro-inflammatory M1 macrophages and the release of a large number of inflammatory cytokines (such as IL-1, IL-6, TNF-α, and iNOS). Uncontrolled inflammation and endotoxin attack can even trigger systemic inflammatory response syndrome (SIRS) and multiple organ dysfunction syndrome (MODS) (15). Therefore, effectively blocking the polarization of macrophages towards the M1 type is beneficial for tissue repair (26, 41). This study conducted on mice with CLP-induced sepsis suggests that gastrointestinal injury is accompanied by an abundance of M1 macrophages in the circulatory system and intestinal tissues, along with significantly increased levels of related inflammatory factors. Although the levels of M2-related proteins, such as IL-10 and Arg-1, were higher in the model mice compared to the control group, the mice in the model group still exhibited characteristics of intestinal tissue inflammation damage.

In this study, EA was chosen as an intervention method, targeting acupoints of PC6, ST36, CV12, and LR3. This intervention method demonstrated a certain degree of alleviation of intestinal injury. EA inhibited the expression of pro-inflammatory mediators, promoted the expression of IL-10 and Arg-1, and resulted in a dominance of M2 macrophages. Therefore, it can be concluded that EA’s effect on alleviating gastrointestinal damage related to sepsis is associated with macrophage polarization.

Existing research indicates that polyamines play a role in promoting intestinal mucosal integrity and barrier function. Moreover, polyamines have been shown to enhance the proliferation of intestinal epithelial cells and facilitate epithelial repair following injury (28). Polyamines are cationic aliphatic polymers found in living organisms, including various types such as putrescine, spermidine, spermine, and agmatine. Putrescine, spermidine, and spermine can be directly synthesized by mammalian cells, while agmatine is either ingested from food or produced by intestinal flora (42, 43). Polyamines are closely related to the life activities of mammals and participate in many fundamental cellular processes, including cell differentiation, apoptosis, and protein and DNA synthesis (27).

Arginine is one of the precursor substances for polyamine biosynthesis. Arginine is converted to ornithine through the action of Arg-1, which is further catalyzed to produce putrescine by ODC. Putrescine, as a base substance, is then converted to spermidine by SPDS, and spermidine is further converted to spermine by spermine synthase (SMS) (44). Studies have shown that spermidine can inhibit fatal sepsis and has beneficial effects on cardiac protection, anti-aging, and immune regulation (27, 45). In this study, the expression of Arg-1, ODC, and spermidine in polyamine metabolism were observed. It was confirmed that EA inhibited ODC mRNA levels but promoted Arg-1 expression, ultimately leading to increased spermidine levels. To further investigate the relationship between polyamines and macrophage polarization, a polyamine depletion model was created by using DFMO in mice. It was determined that after polyamine depletion, EA lost its protective effect against intestinal damage and failed to induce M2 macrophage differentiation. Similarly, *in vitro* experiments the relationship between spermidine and macrophage polarization were also confirmed. Spermidine reduced the secretion of TNF-α and IL-1β in LPS-stimulated RAW 264.7 macrophages and MCP-1 secretion in THP-1 macrophages treated with IFN-γ (46, 47).

In summary, this study established a polyamine depletion model and confirmed that the mechanism by which EA alleviates intestinal injury associated with CLP-induced sepsis is through promoting M2 macrophage polarization mediated by spermidine expression. This polarization inhibits the expression of inflammatory factors and promotes intestinal repair. However, polyamines, as guardians of the gastrointestinal tract, are derived from both endogenous intracellular amino acid metabolism and exogenous intestinal microbial metabolism. In future studies, further clarification of the endogenous and exogenous mechanisms by which acupuncture regulates polyamine metabolism and its relationship with intestinal immunity will be explored.

## Data availability statement

The original contributions presented in the study are included in the article/supplementary material. Further inquiries can be directed to the corresponding authors.

## Ethics statement

The animal study was approved by The Animal Ethics Committee of Tianjin University of Traditional Chinese Medicine. The study was conducted in accordance with the local legislation and institutional requirements.

## Author contributions

XX: Funding acquisition, Writing – review & editing, Writing – original draft, Methodology, Conceptualization. JW: Writing – review & editing, Writing – original draft, Methodology. LX: Funding acquisition, Writing – review & editing, Writing – original draft, Supervision, Data curation. XH: Writing – review & editing, Investigation. XY: Writing – review & editing, Funding acquisition. YW: Writing – review & editing, Formal Analysis.
