# Supplementary figures and images for: Mechanism of electro-acupuncture in alleviating intestinal injury in septic mice via polyamine-related M2-macrophage polarization (part 1 of 3)
Source: Front Immunol. 2024 Apr 22;15:1373876. doi: 10.3389/fimmu.2024.1373876 (PMC11075497; doi:10.3389/fimmu.2024.1373876)

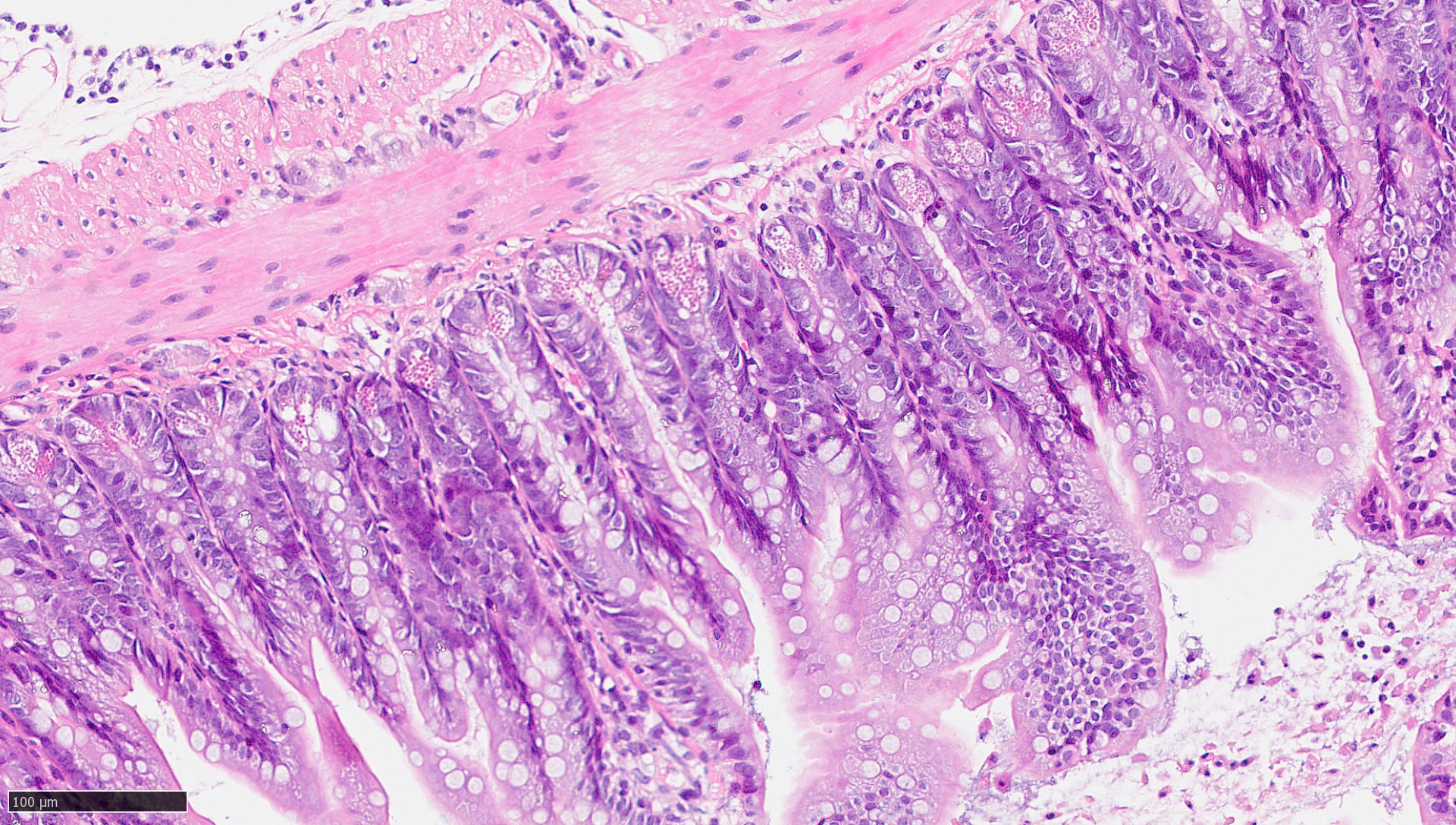

Supplement: Supplementary file 1 [file DataSheet_1.zip › Figure 5/Fig.5 Other files/full scans of Immunohistochemical staining of intestine/DFMO (3).jpg]

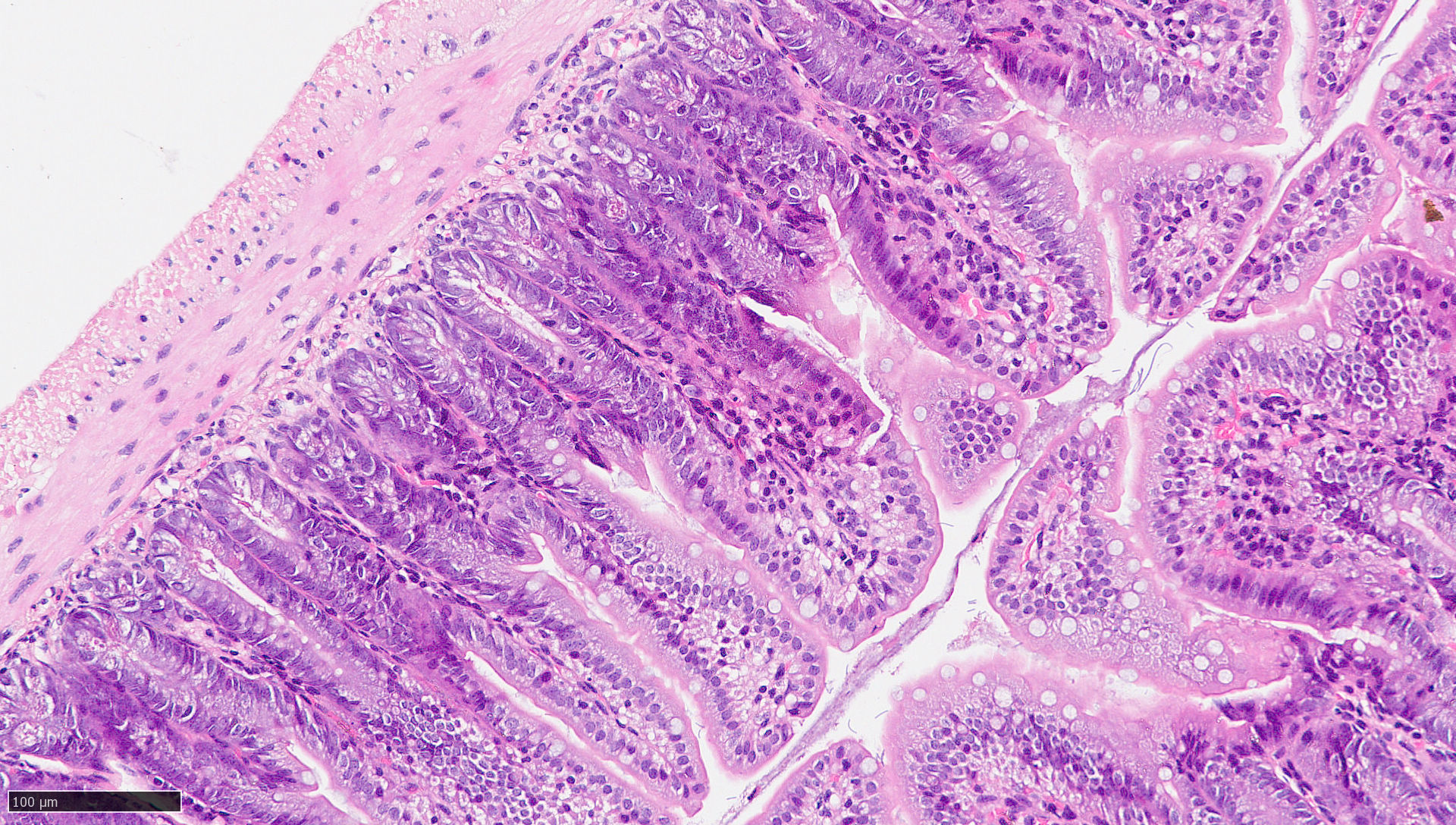

Supplement: Supplementary file 1 [file DataSheet_1.zip › Figure 5/Fig.5 Other files/full scans of Immunohistochemical staining of intestine/DFMO (2).jpg]

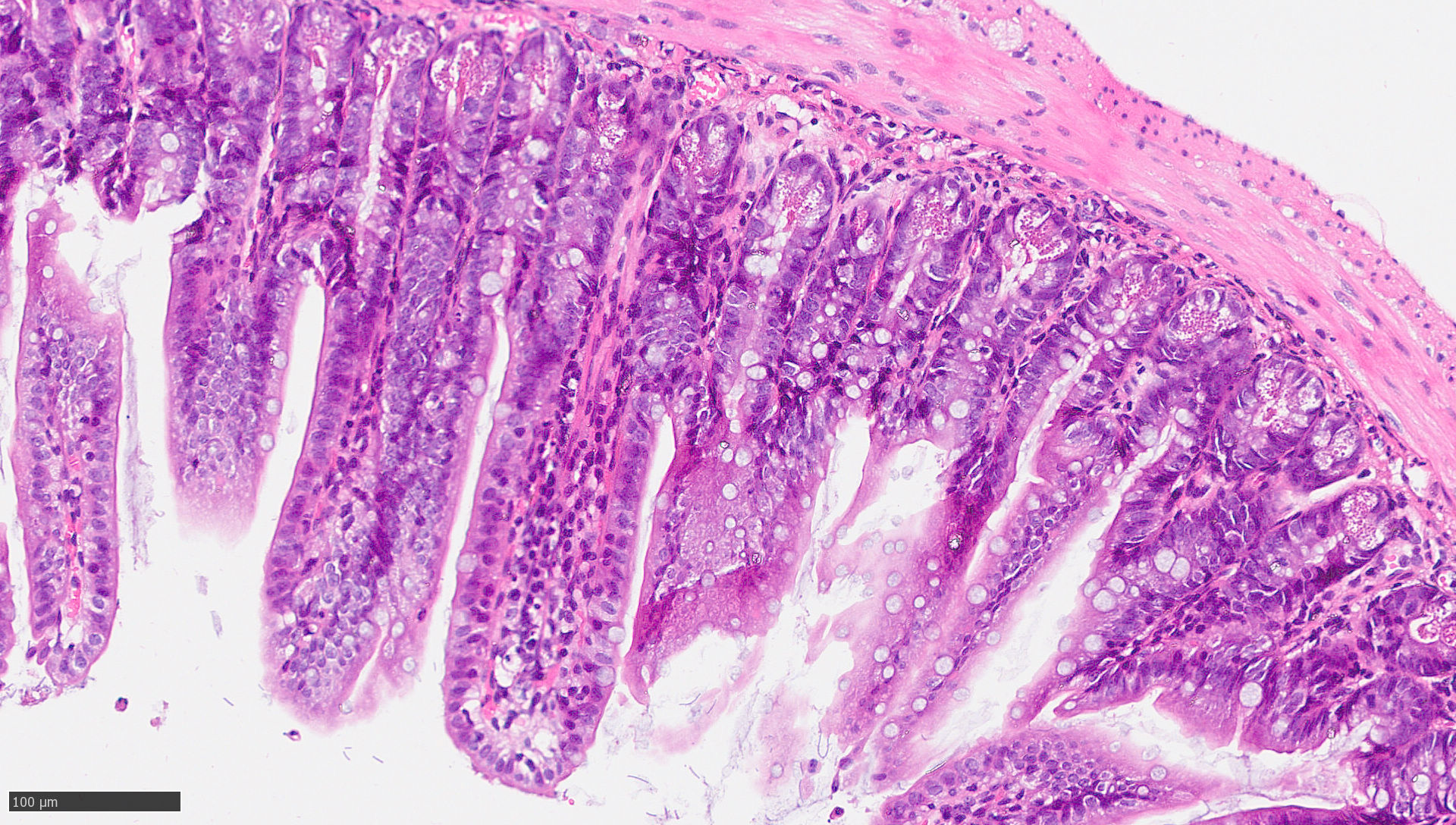

Supplement: Supplementary file 1 [file DataSheet_1.zip › Figure 5/Fig.5 Other files/full scans of Immunohistochemical staining of intestine/CLP (1).jpg]

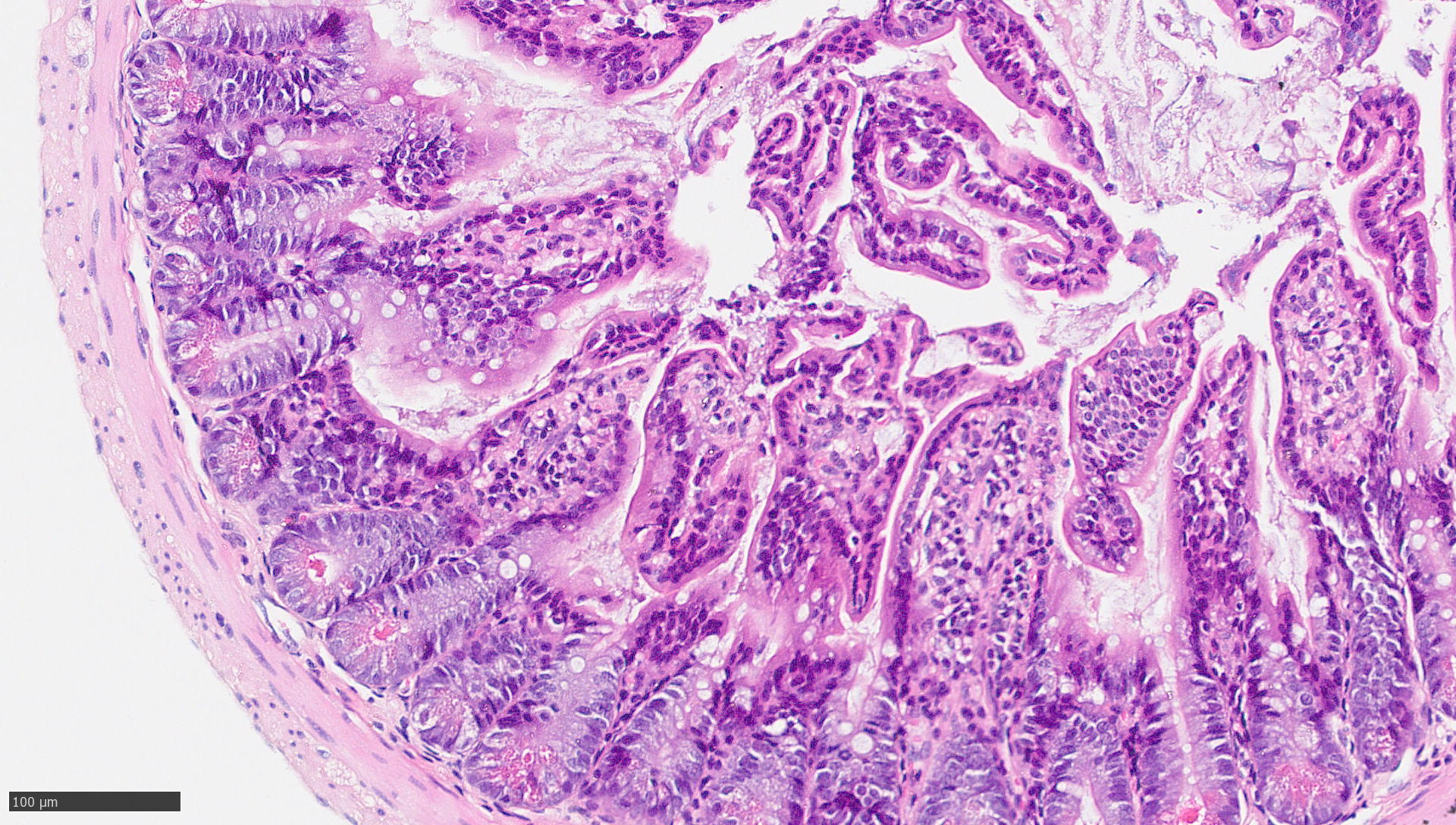

Supplement: Supplementary file 1 [file DataSheet_1.zip › Figure 5/Fig.5 Other files/full scans of Immunohistochemical staining of intestine/DEA (1).jpg]

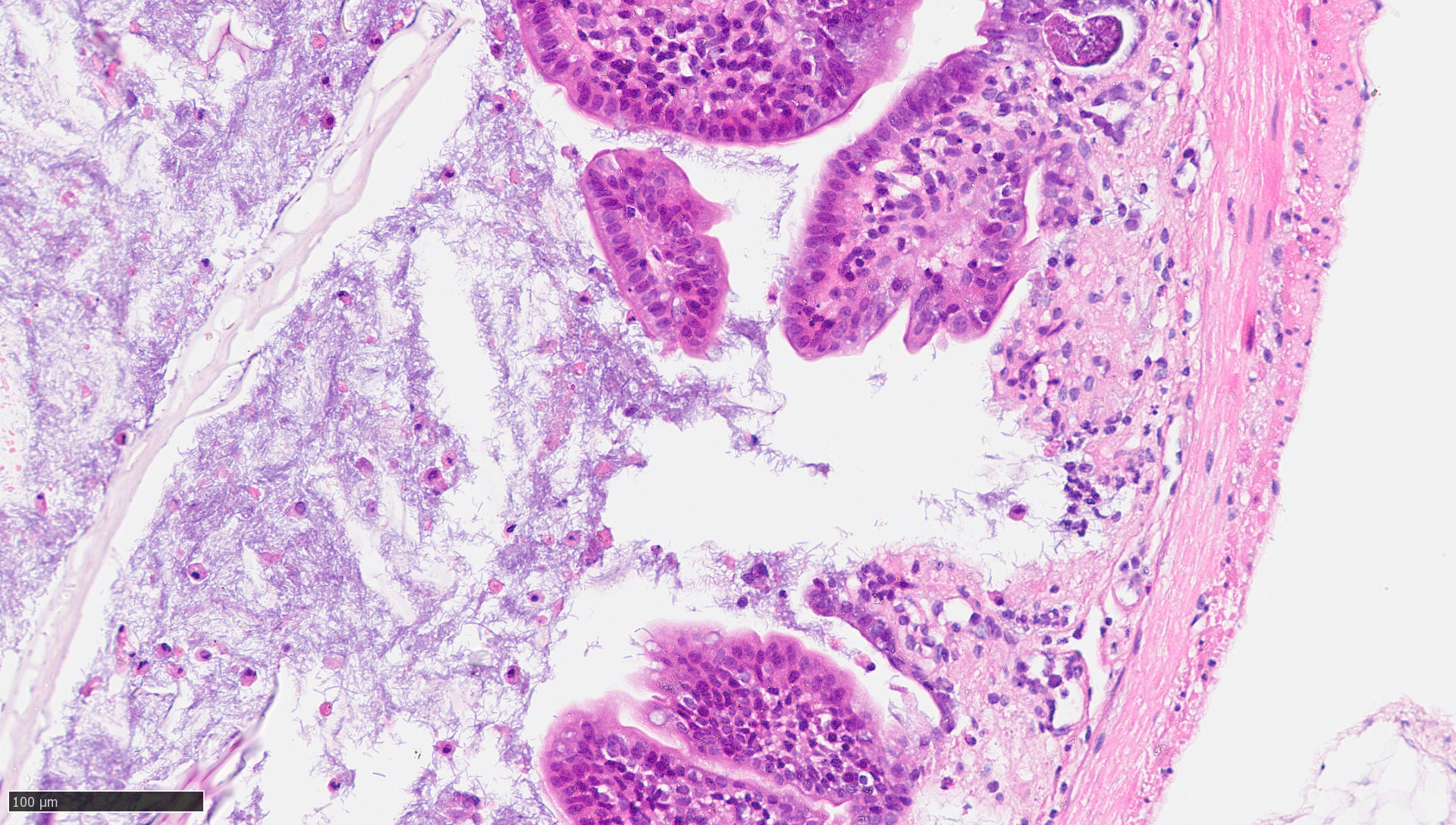

Supplement: Supplementary file 1 [file DataSheet_1.zip › Figure 5/Fig.5 Other files/full scans of Immunohistochemical staining of intestine/DEA (6).jpg]

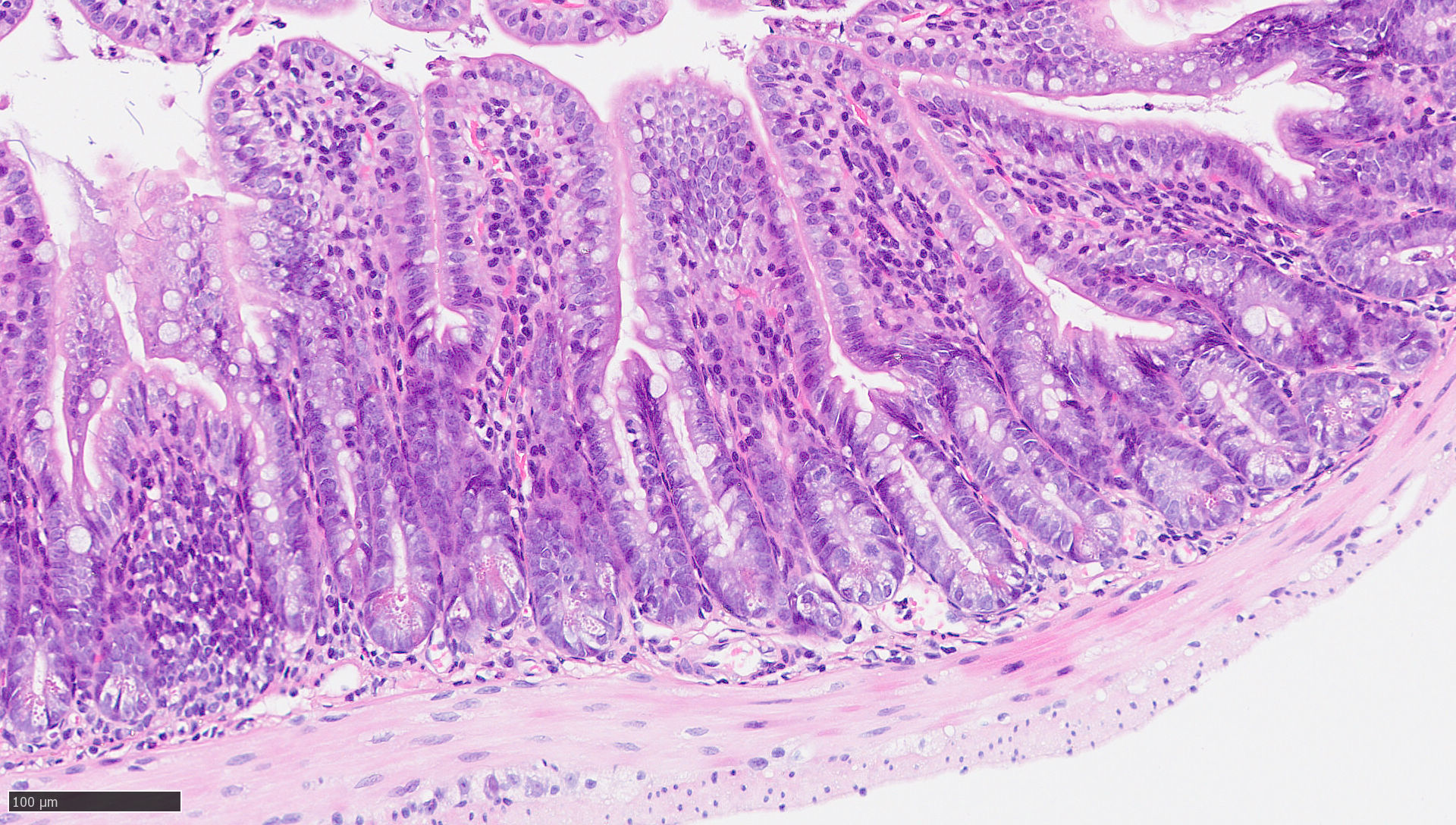

Supplement: Supplementary file 1 [file DataSheet_1.zip › Figure 5/Fig.5 Other files/full scans of Immunohistochemical staining of intestine/CLP (6).jpg]

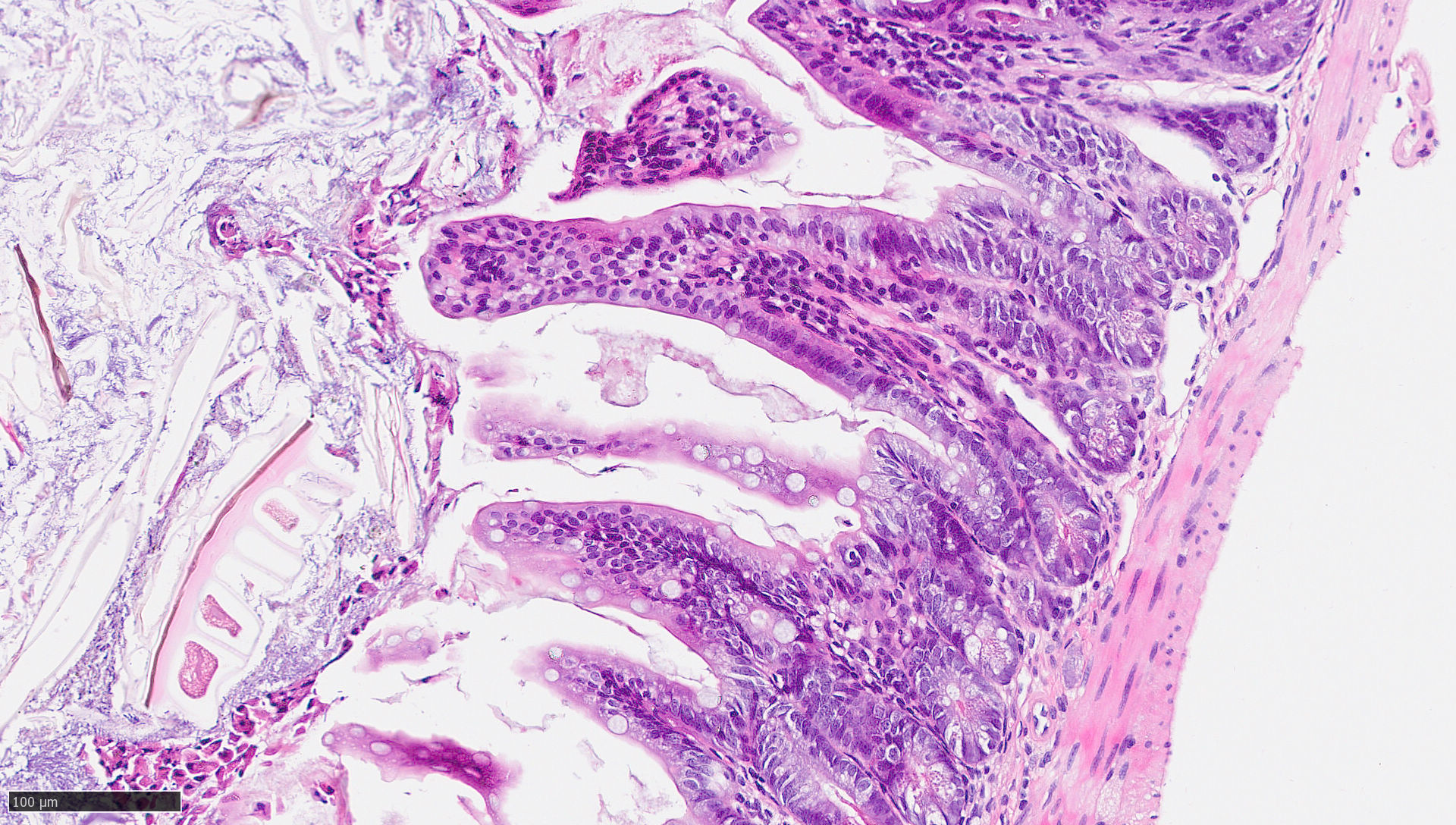

Supplement: Supplementary file 1 [file DataSheet_1.zip › Figure 5/Fig.5 Other files/full scans of Immunohistochemical staining of intestine/DFMO (5).jpg]

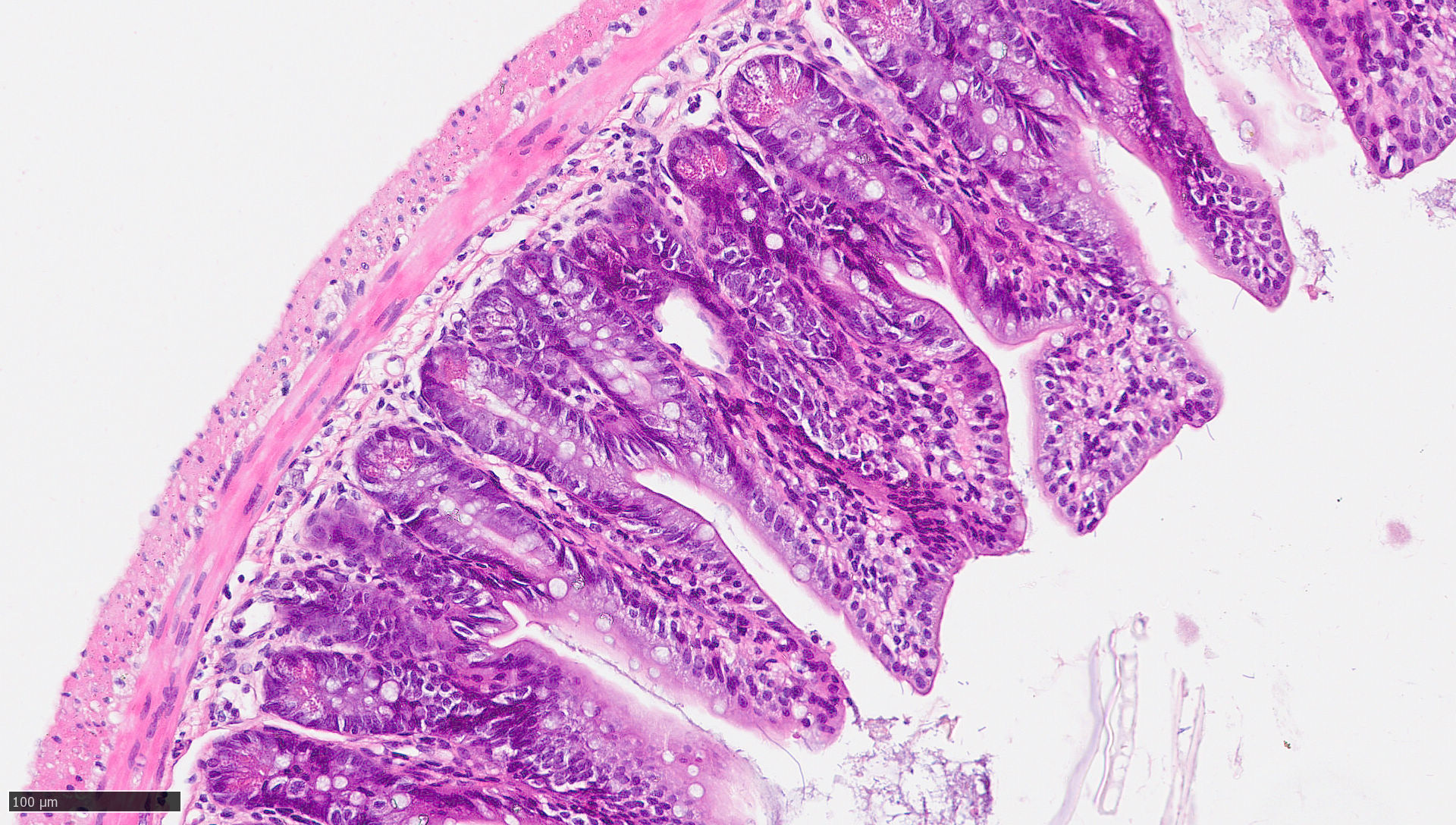

Supplement: Supplementary file 1 [file DataSheet_1.zip › Figure 5/Fig.5 Other files/full scans of Immunohistochemical staining of intestine/DFMO (4).jpg]

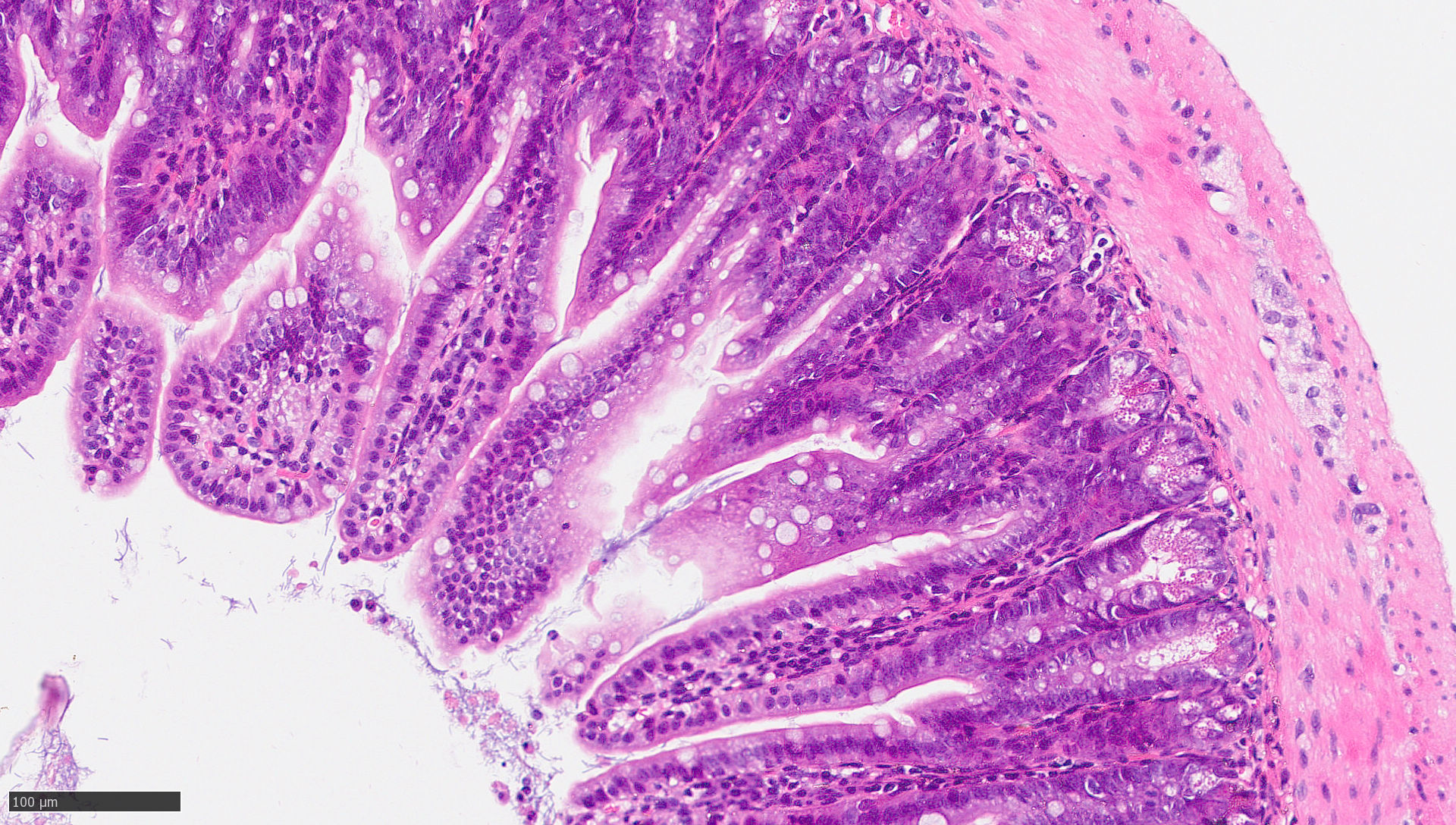

Supplement: Supplementary file 1 [file DataSheet_1.zip › Figure 5/Fig.5 Other files/full scans of Immunohistochemical staining of intestine/CLP (4).jpg]

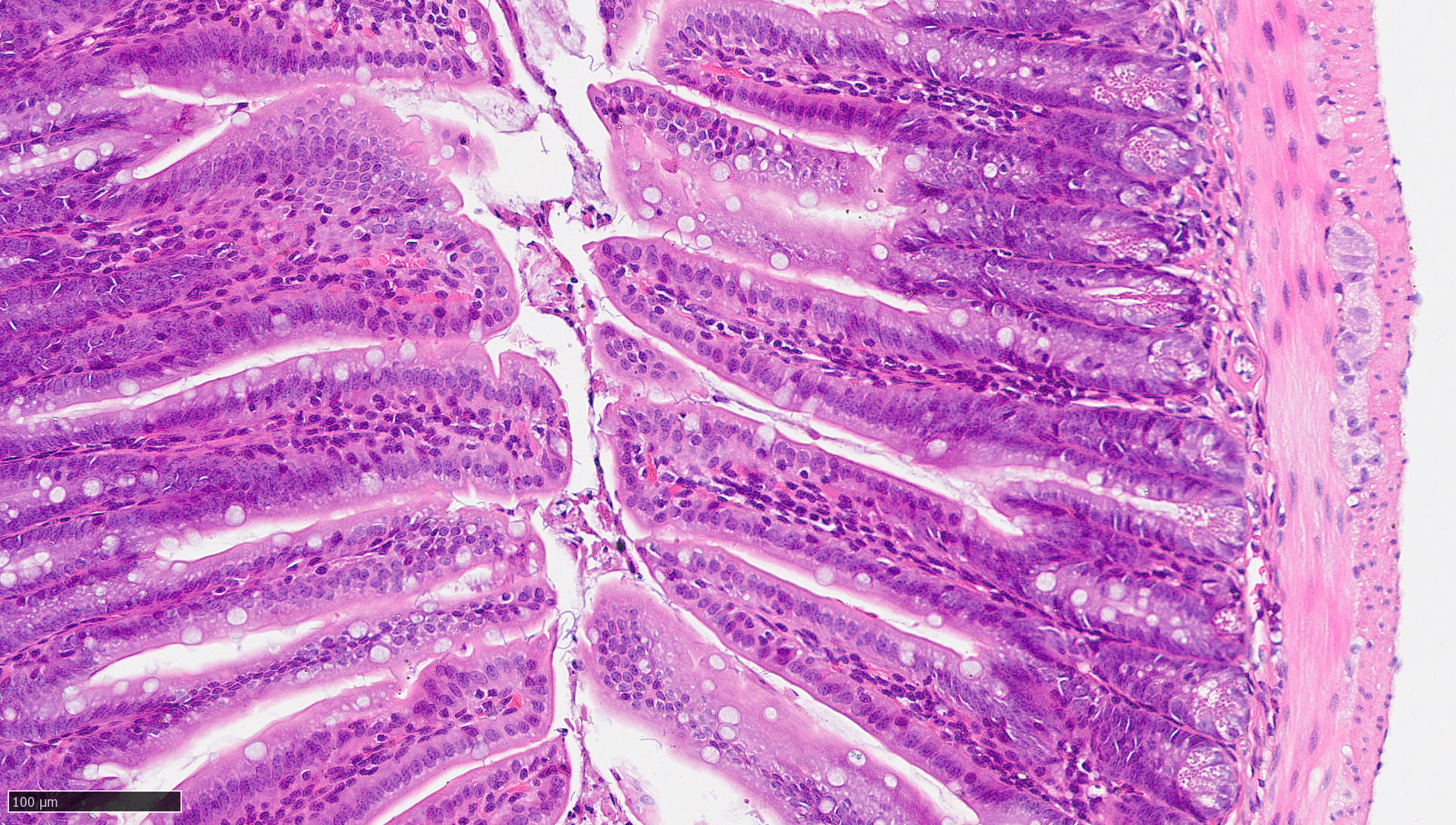

Supplement: Supplementary file 1 [file DataSheet_1.zip › Figure 5/Fig.5 Other files/full scans of Immunohistochemical staining of intestine/DEA (4).jpg]

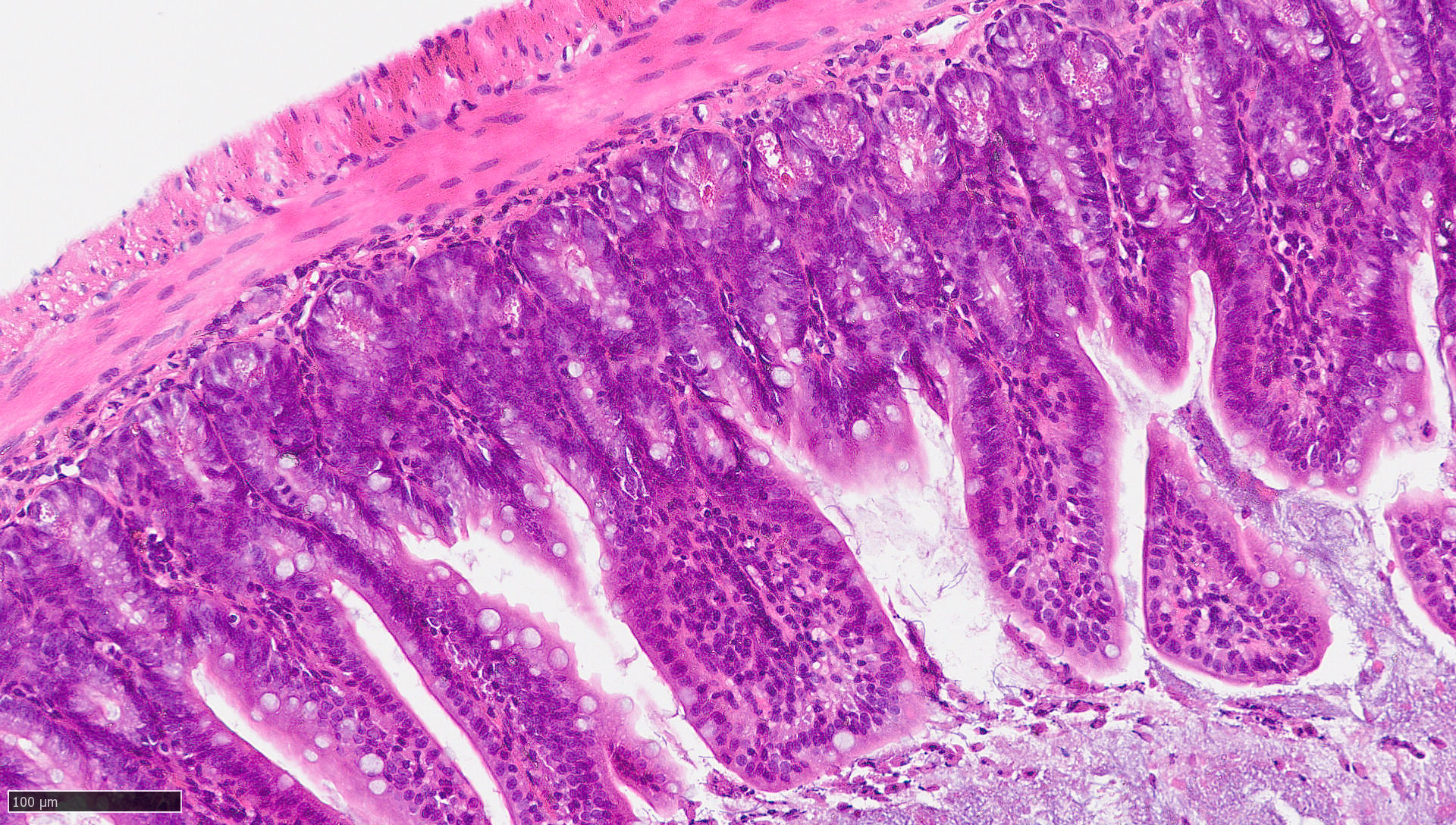

Supplement: Supplementary file 1 [file DataSheet_1.zip › Figure 5/Fig.5 Other files/full scans of Immunohistochemical staining of intestine/DEA (5).jpg]

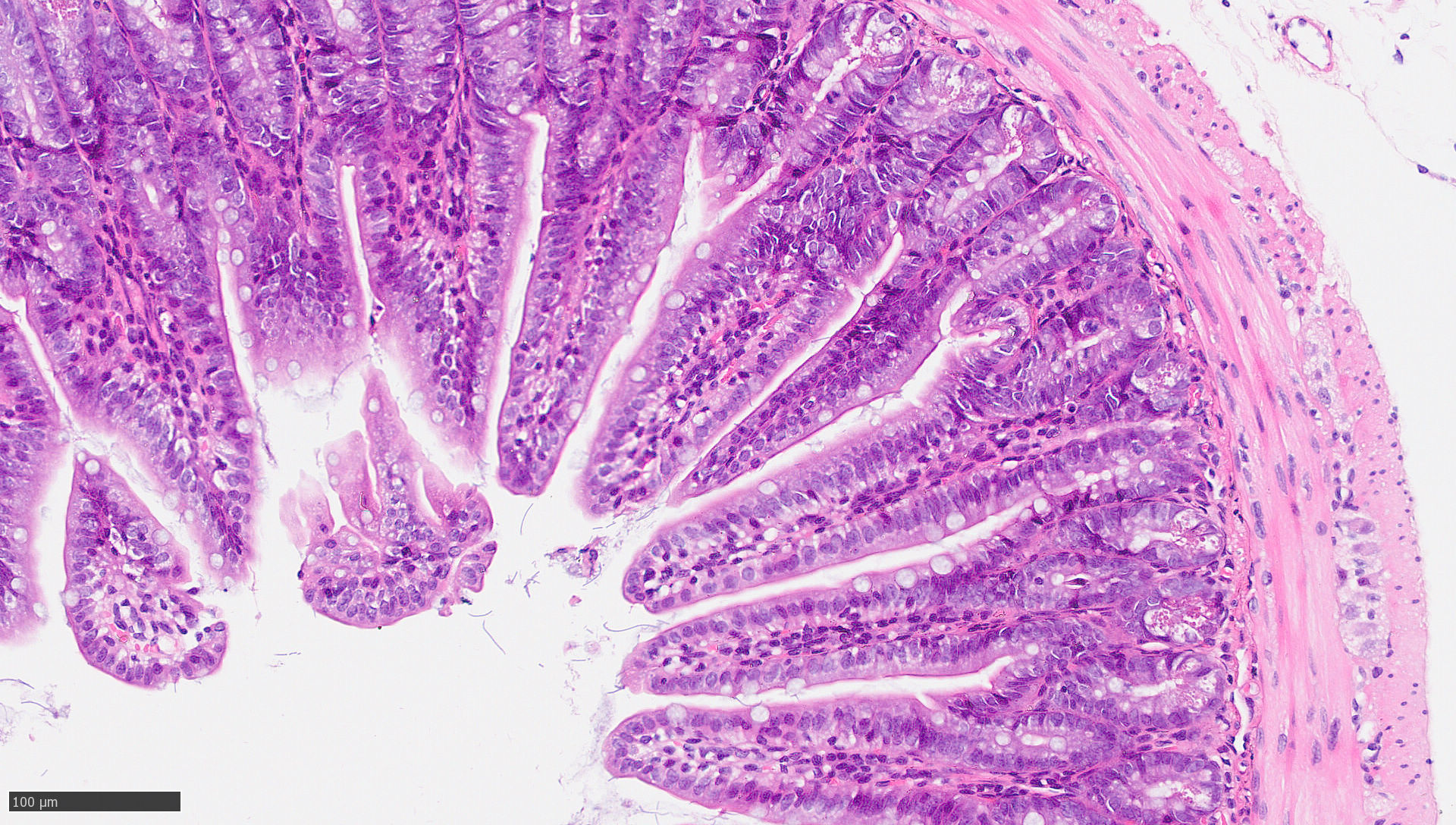

Supplement: Supplementary file 1 [file DataSheet_1.zip › Figure 5/Fig.5 Other files/full scans of Immunohistochemical staining of intestine/CLP (5).jpg]

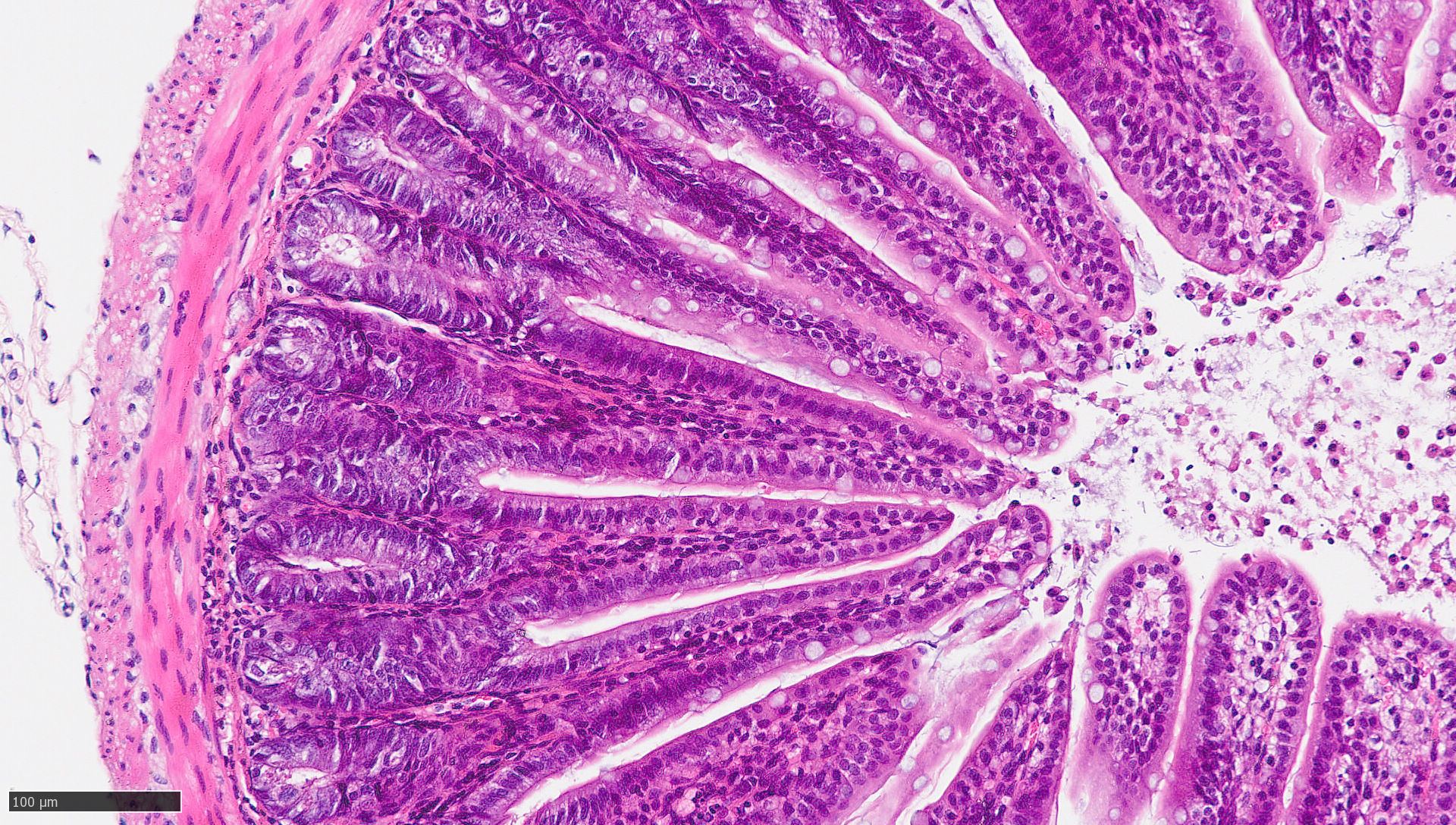

Supplement: Supplementary file 1 [file DataSheet_1.zip › Figure 5/Fig.5 Other files/full scans of Immunohistochemical staining of intestine/DFMO (6).jpg]

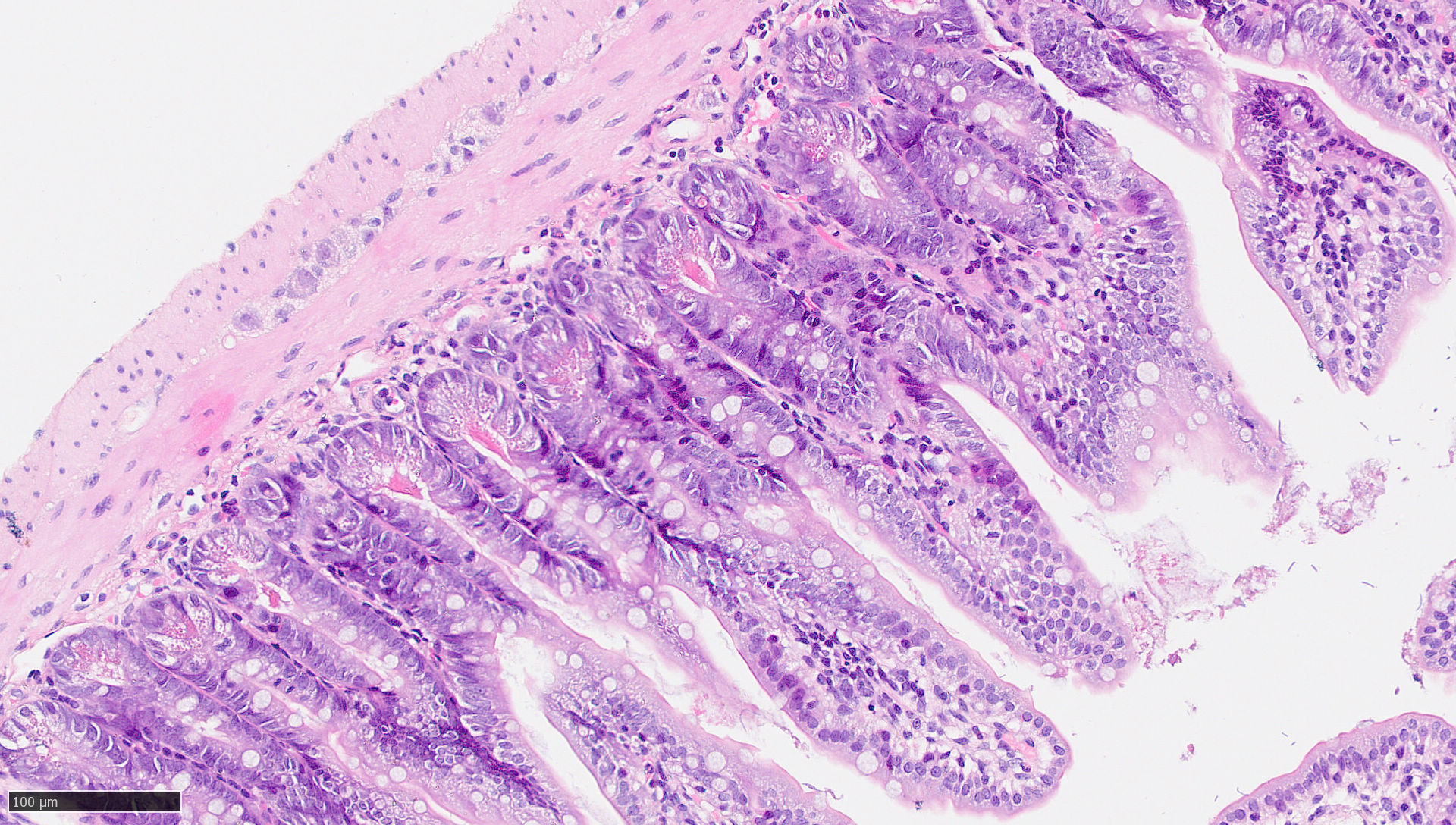

Supplement: Supplementary file 1 [file DataSheet_1.zip › Figure 5/Fig.5 Other files/full scans of Immunohistochemical staining of intestine/DFMO (1).jpg]

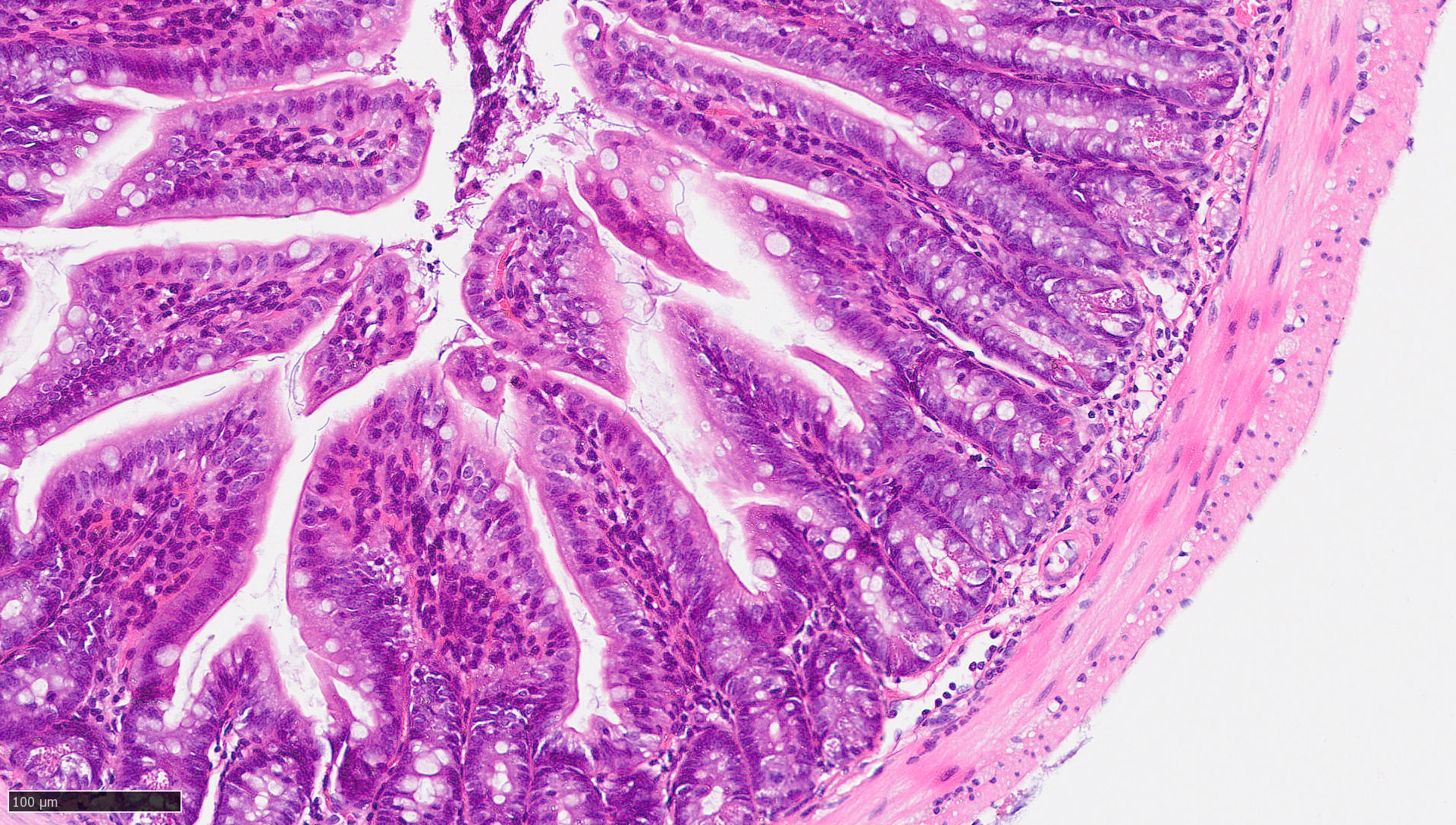

Supplement: Supplementary file 1 [file DataSheet_1.zip › Figure 5/Fig.5 Other files/full scans of Immunohistochemical staining of intestine/CLP (2).jpg]

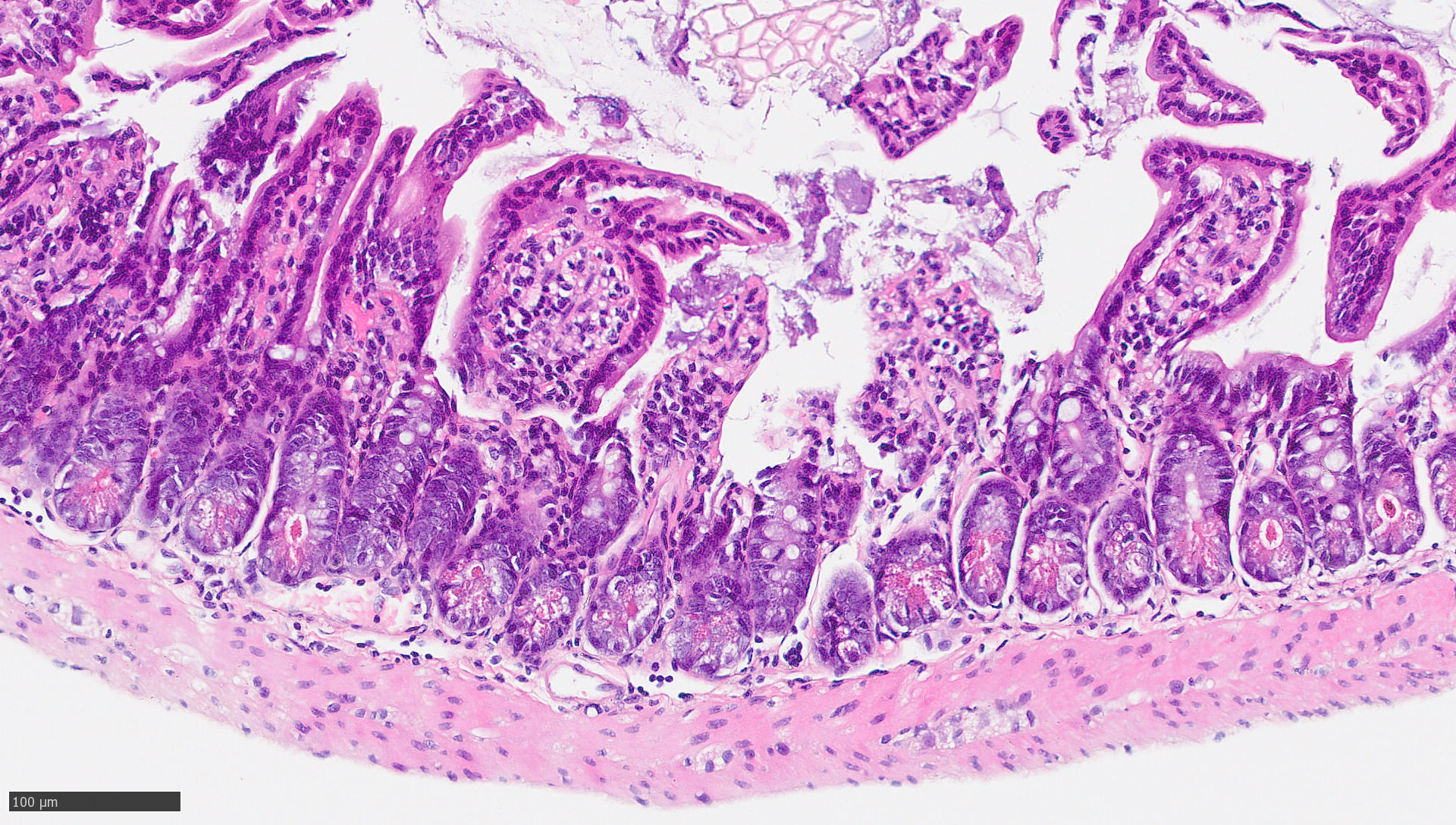

Supplement: Supplementary file 1 [file DataSheet_1.zip › Figure 5/Fig.5 Other files/full scans of Immunohistochemical staining of intestine/DEA (2).jpg]

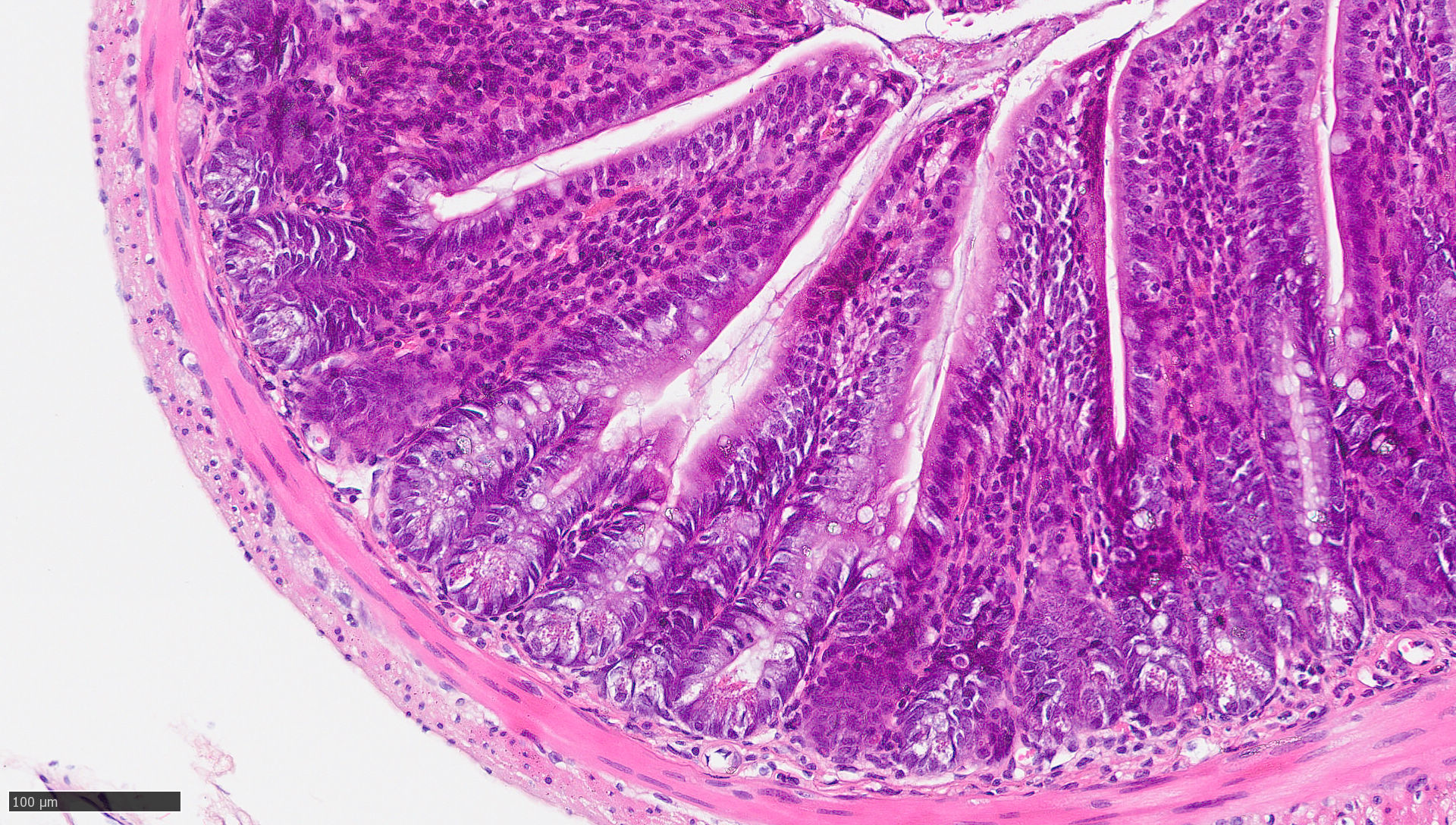

Supplement: Supplementary file 1 [file DataSheet_1.zip › Figure 5/Fig.5 Other files/full scans of Immunohistochemical staining of intestine/DEA (3).jpg]

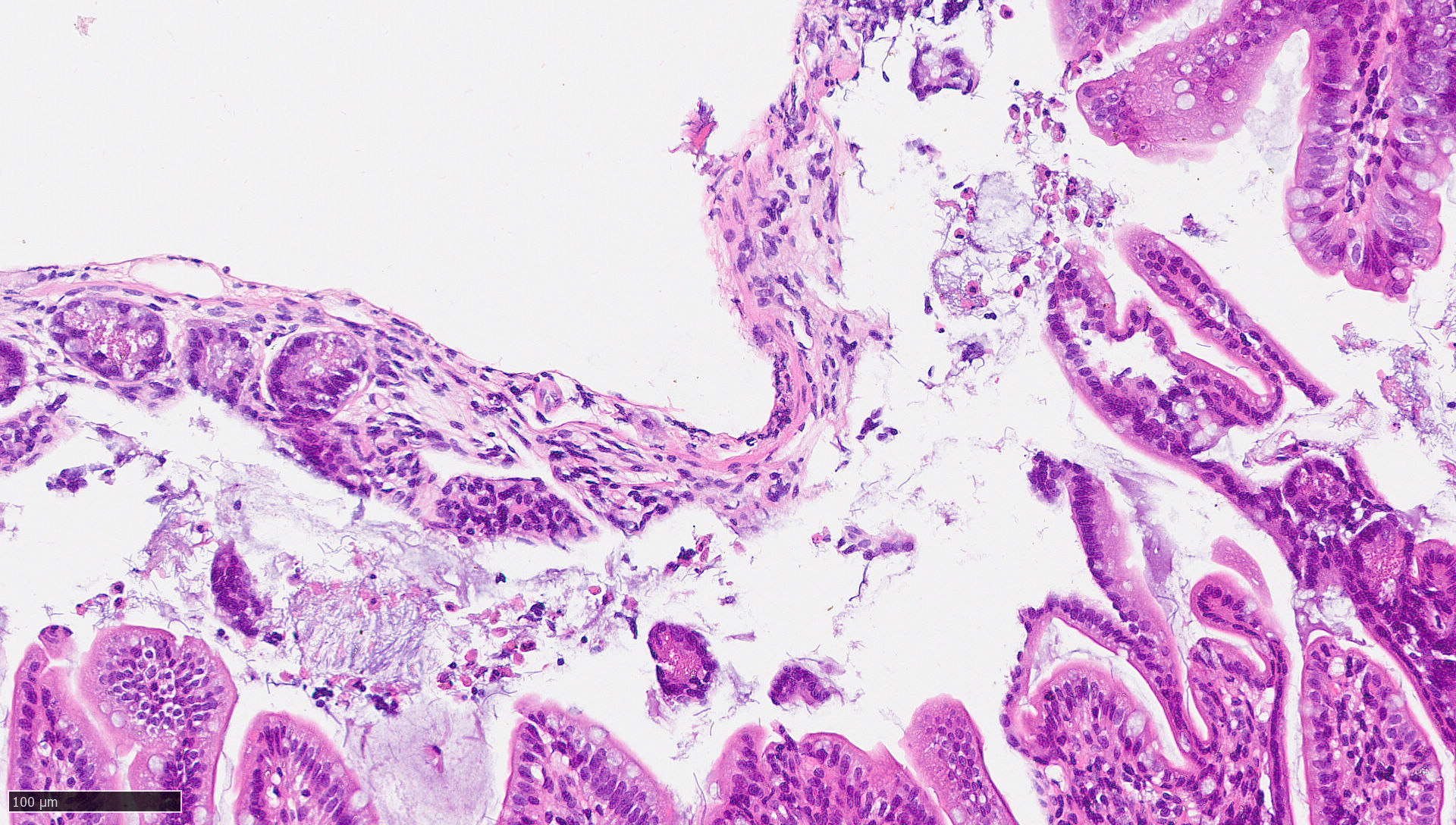

Supplement: Supplementary file 1 [file DataSheet_1.zip › Figure 5/Fig.5 Other files/full scans of Immunohistochemical staining of intestine/CLP (3).jpg]

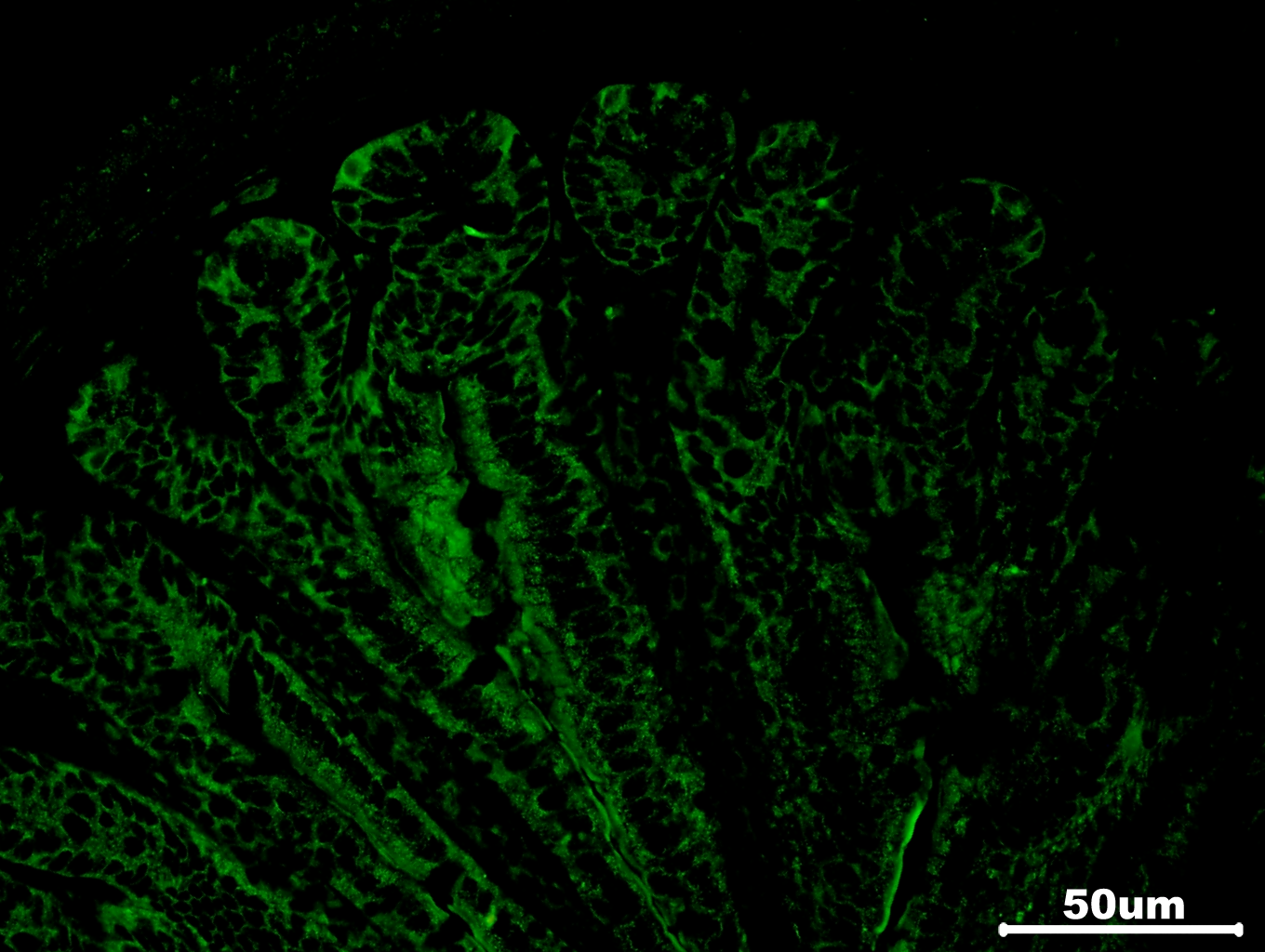

Supplement: Supplementary file 3 [file DataSheet_3.zip › A19-2-200-1-CD86.tif]

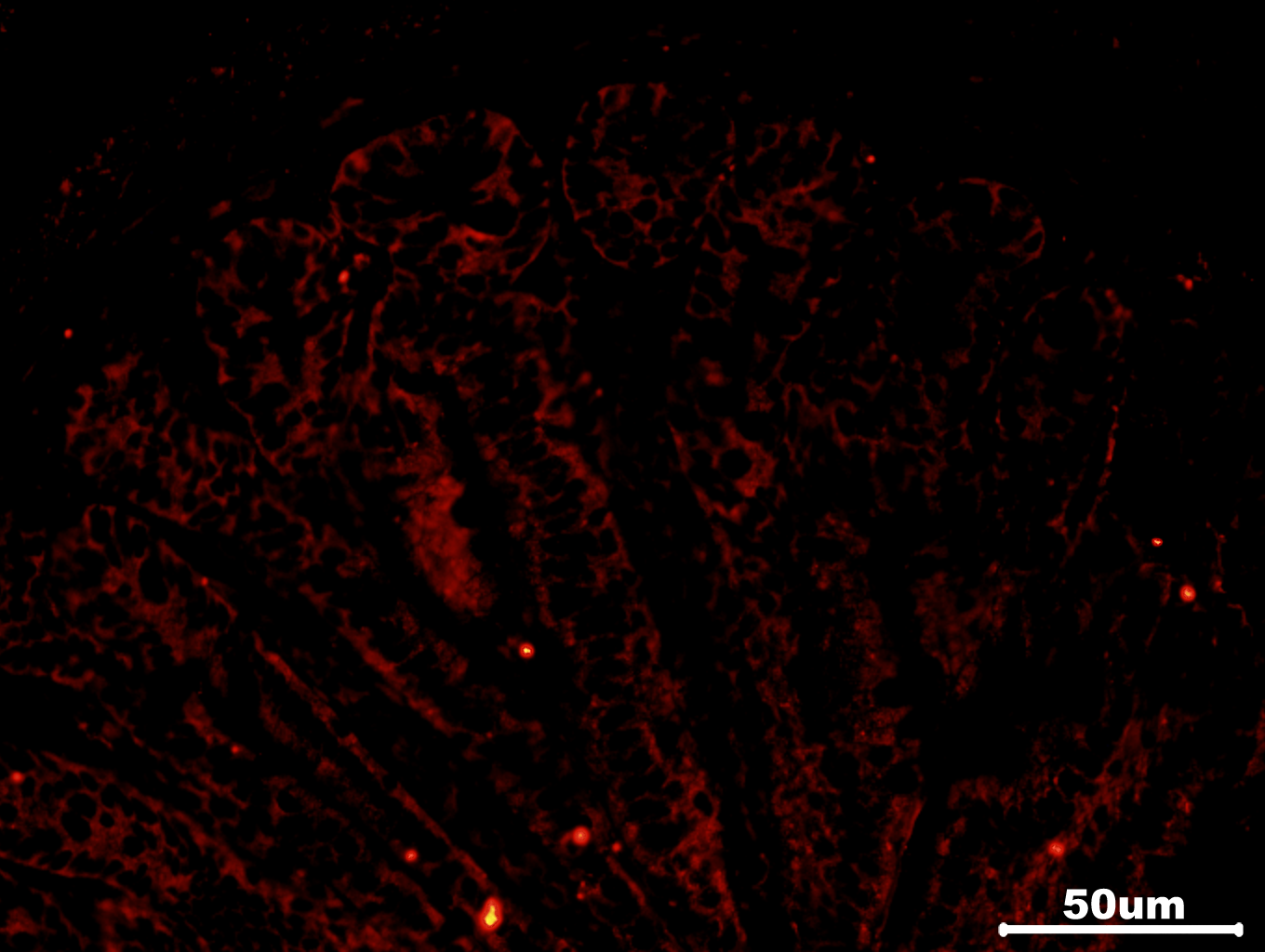

Supplement: Supplementary file 3 [file DataSheet_3.zip › A19-2-200-1-CD206.tif]

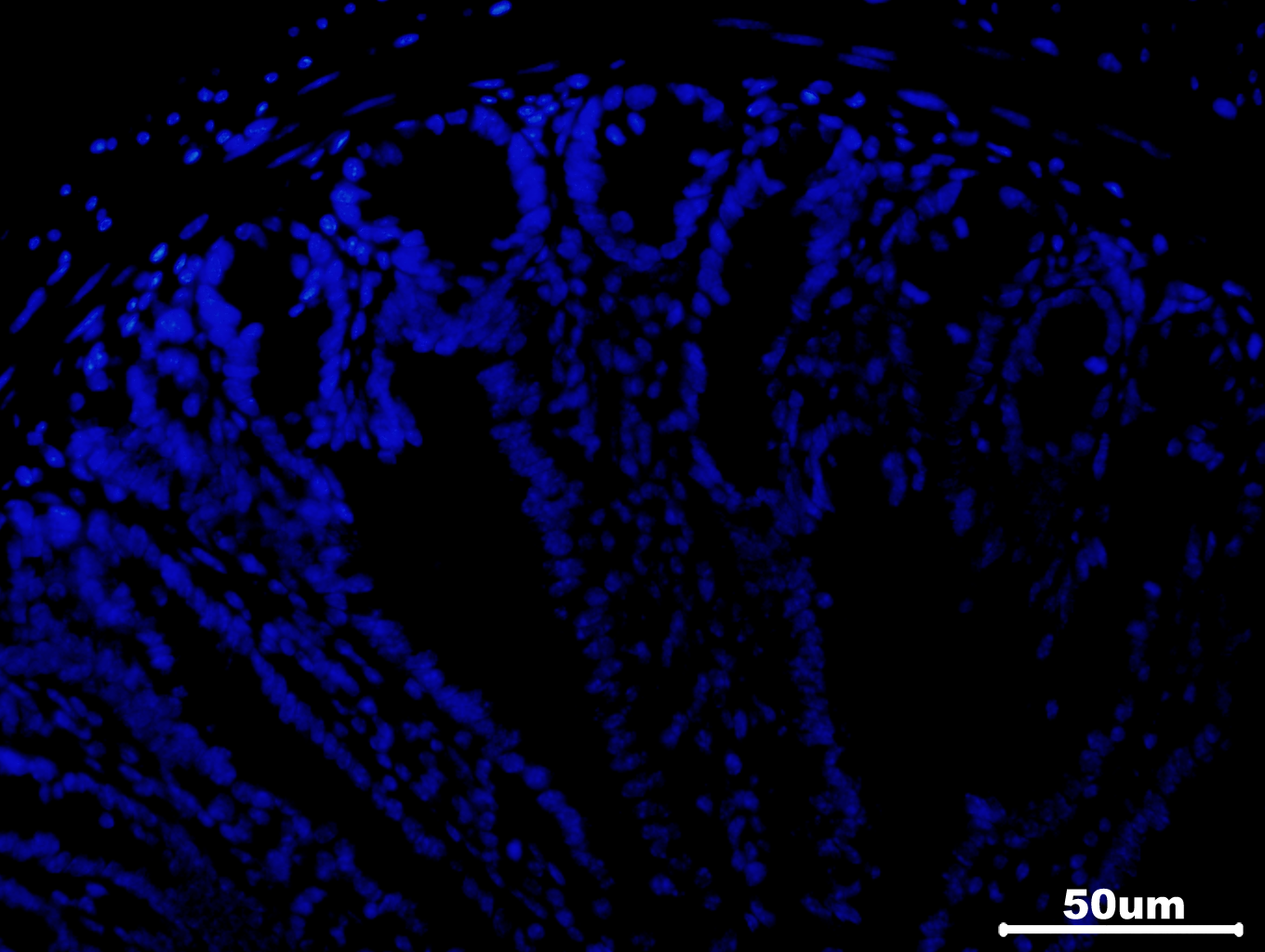

Supplement: Supplementary file 3 [file DataSheet_3.zip › A19-2-200-1-DAPI.tif]

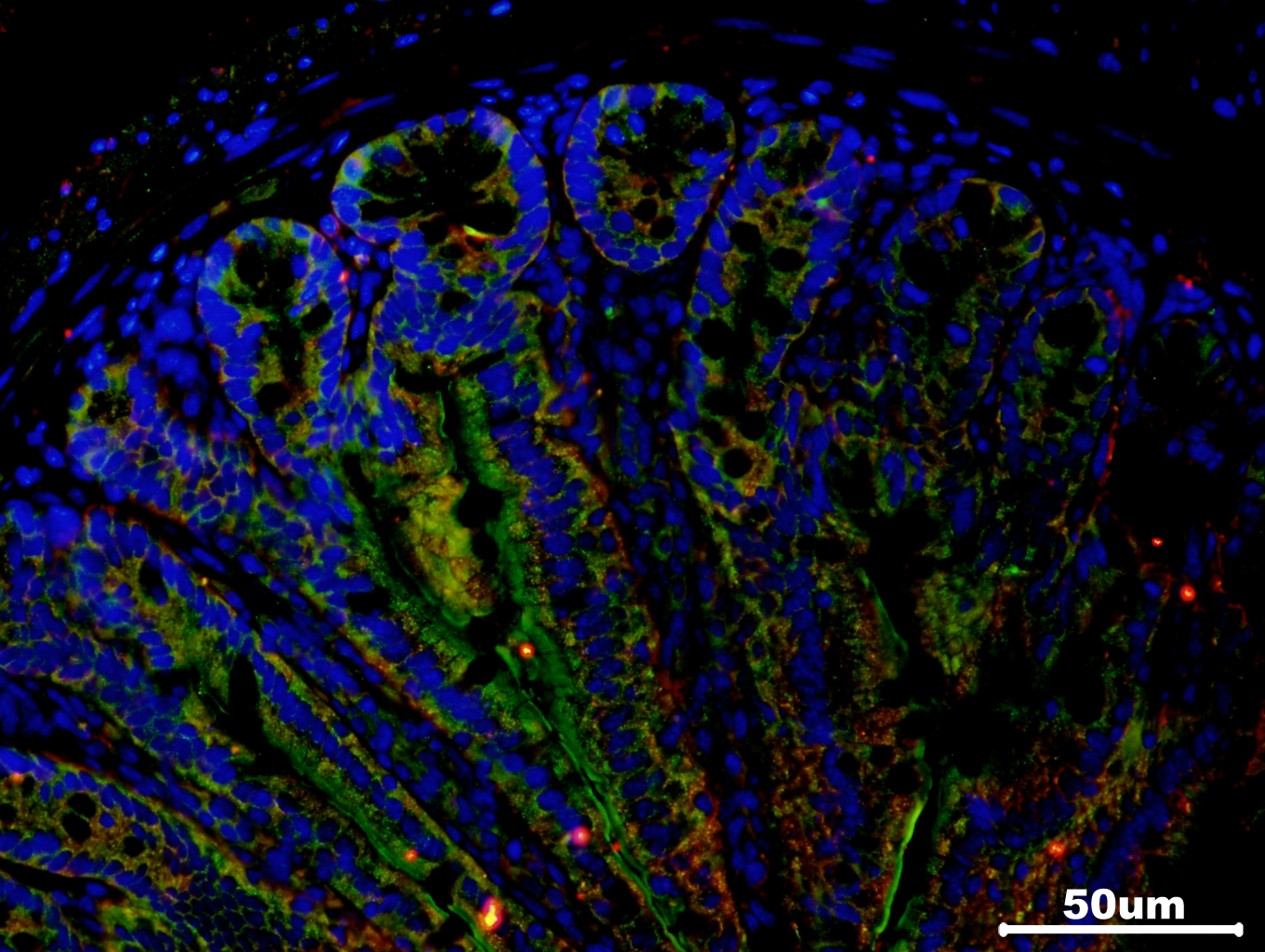

Supplement: Supplementary file 3 [file DataSheet_3.zip › A19-2-200-1-merge.tif]

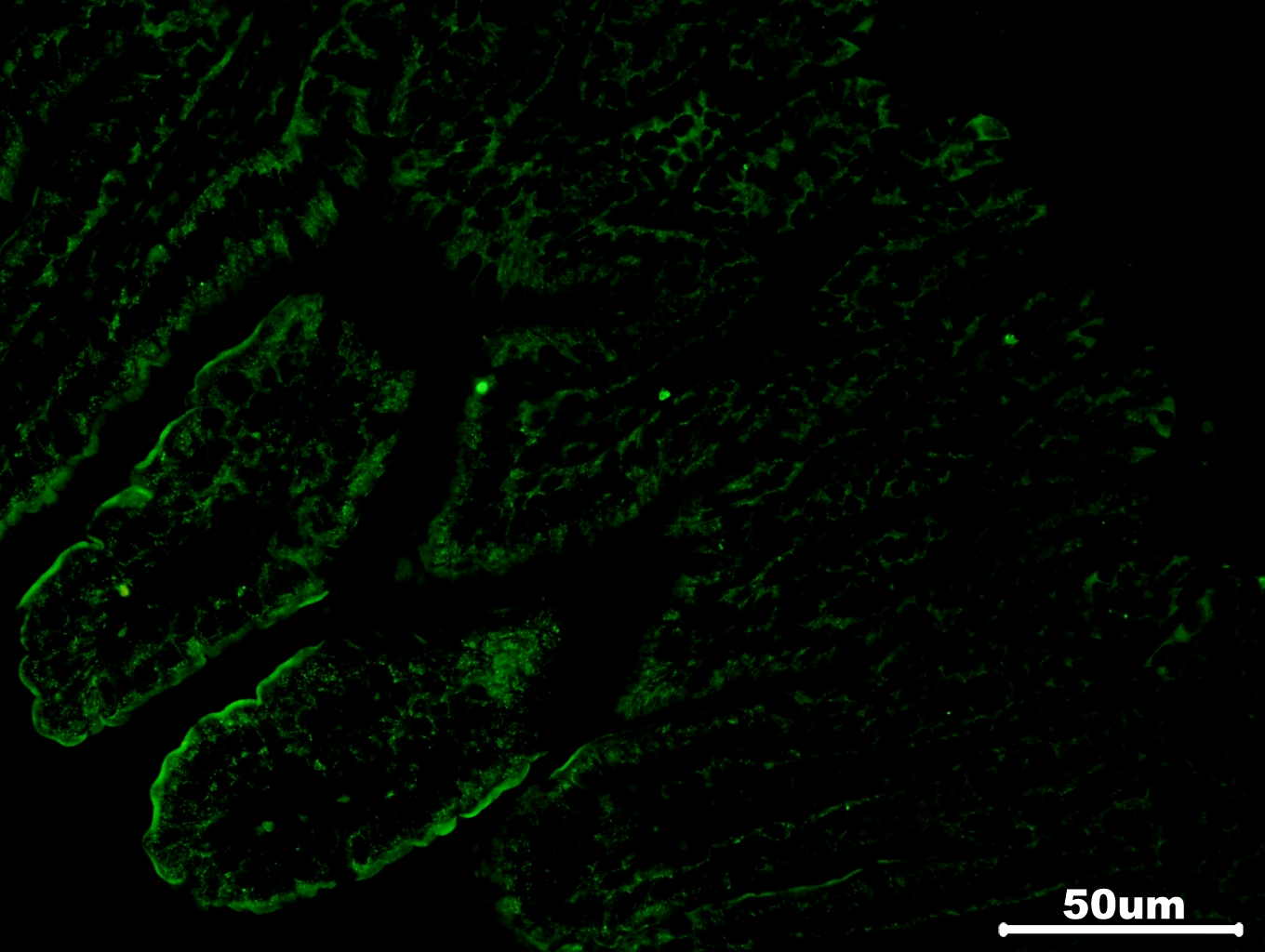

Supplement: Supplementary file 3 [file DataSheet_3.zip › A19-2-200-2-CD86.tif]

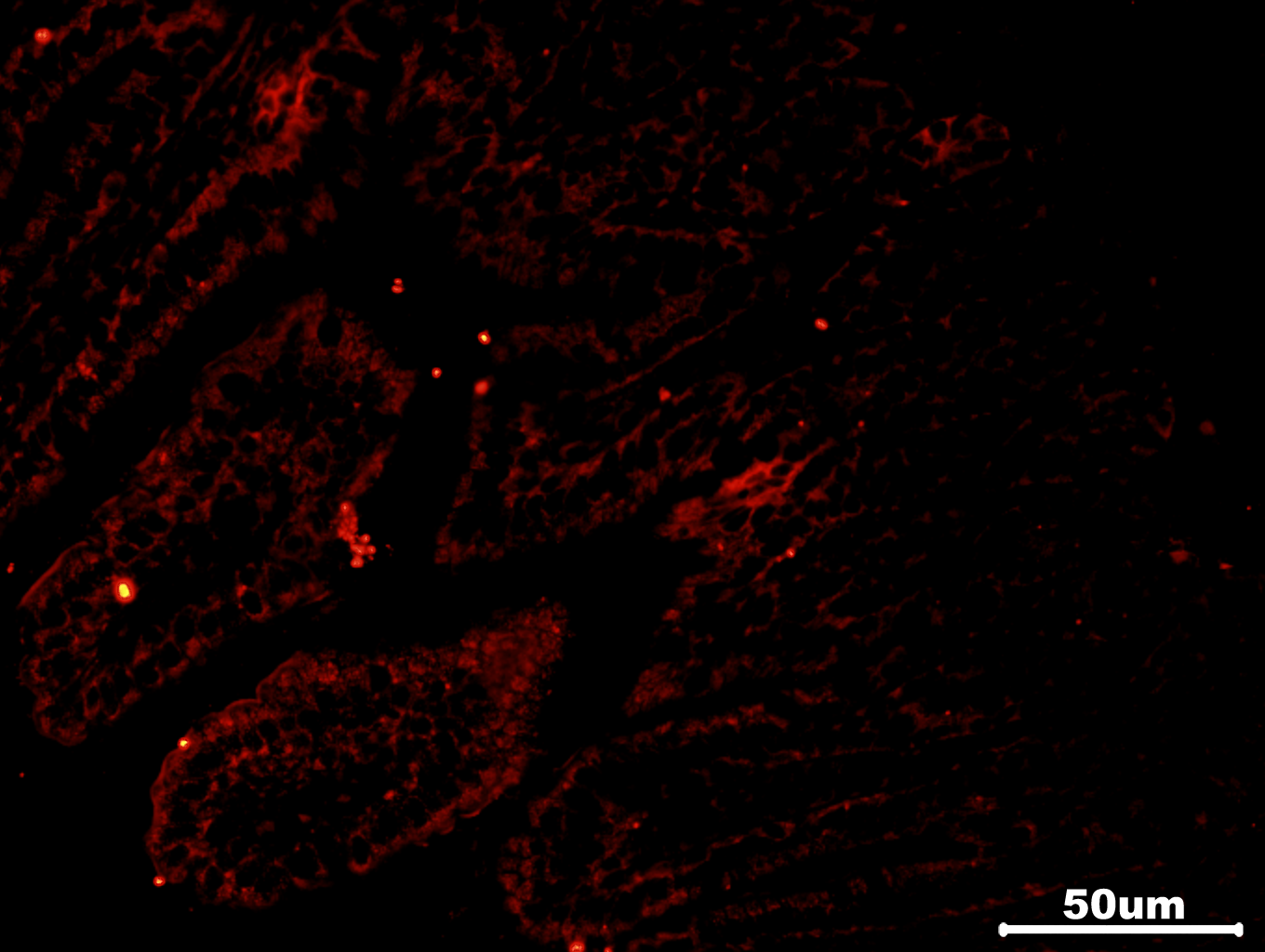

Supplement: Supplementary file 3 [file DataSheet_3.zip › A19-2-200-2-CD206.tif]

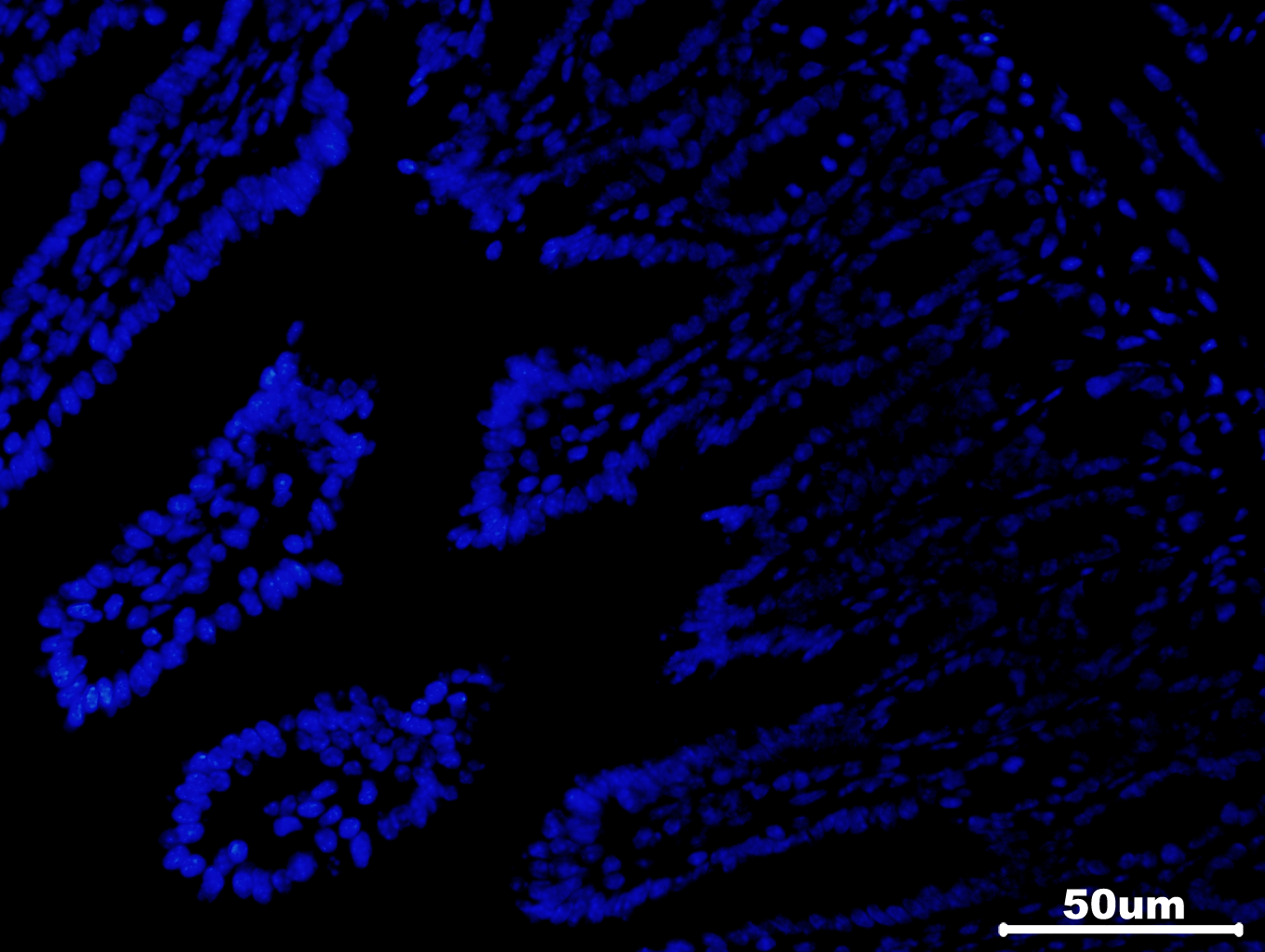

Supplement: Supplementary file 3 [file DataSheet_3.zip › A19-2-200-2-DAPI.tif]

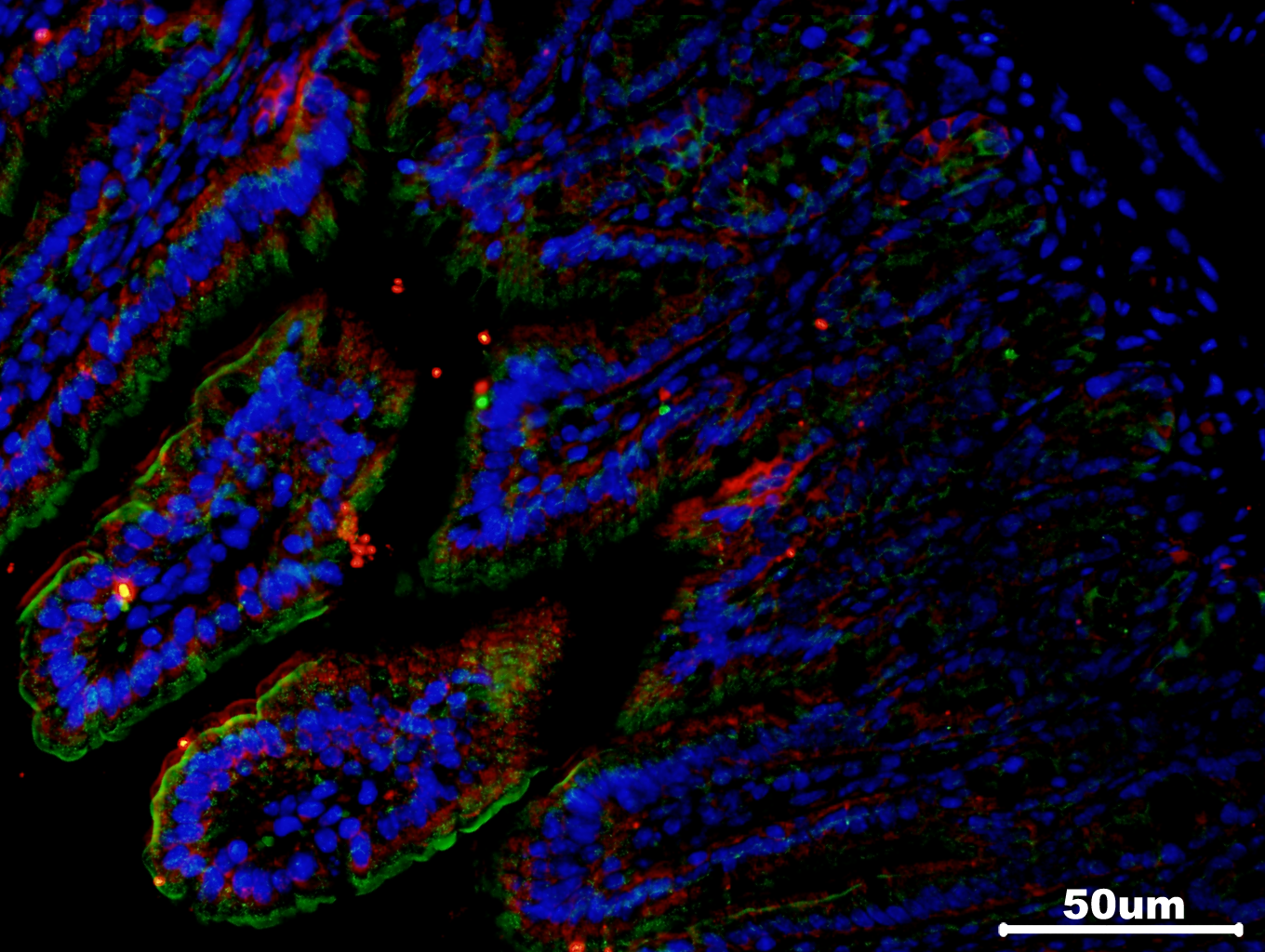

Supplement: Supplementary file 3 [file DataSheet_3.zip › A19-2-200-2-merge.tif]

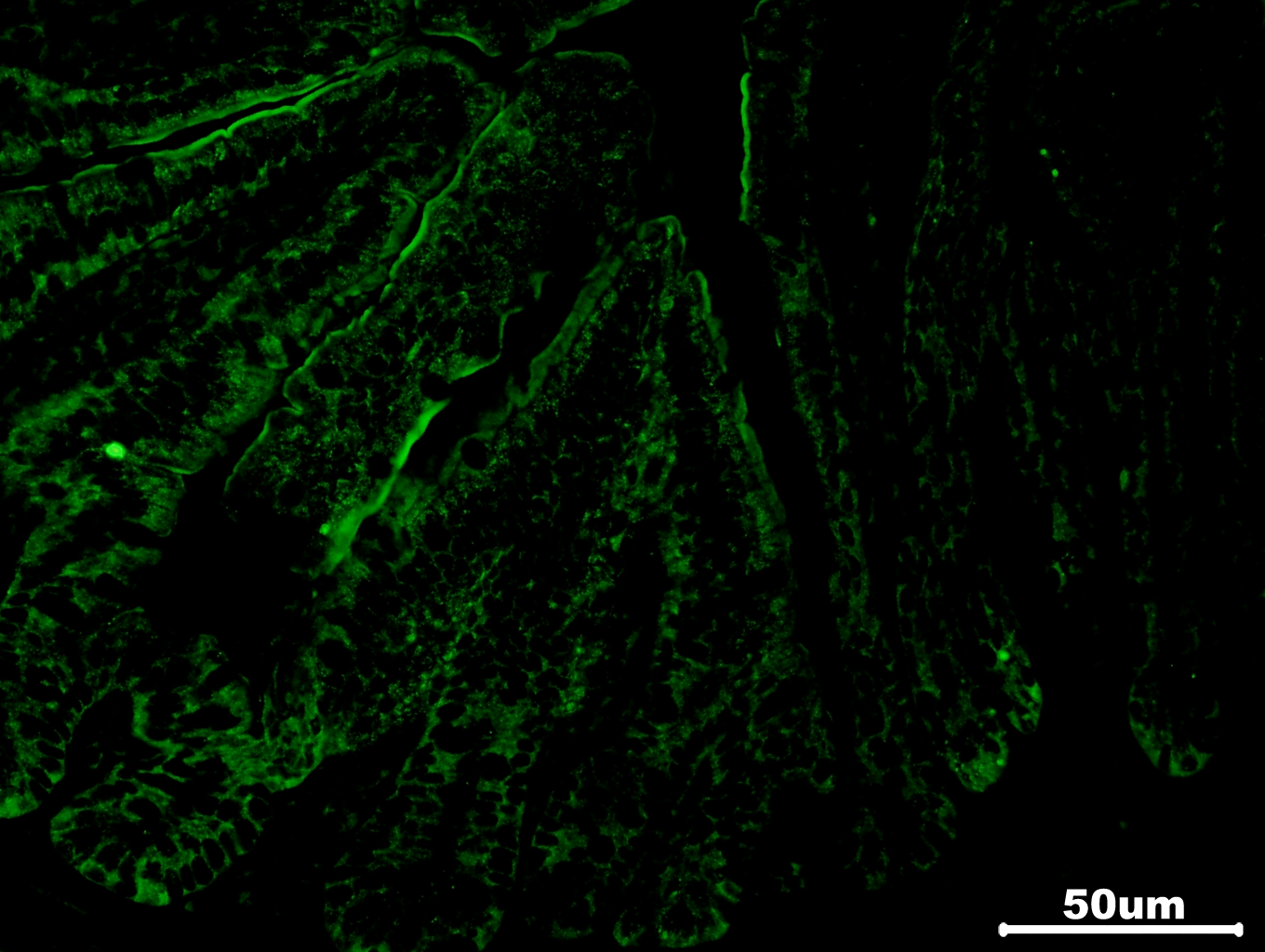

Supplement: Supplementary file 3 [file DataSheet_3.zip › A19-2-200-3-CD86.tif]

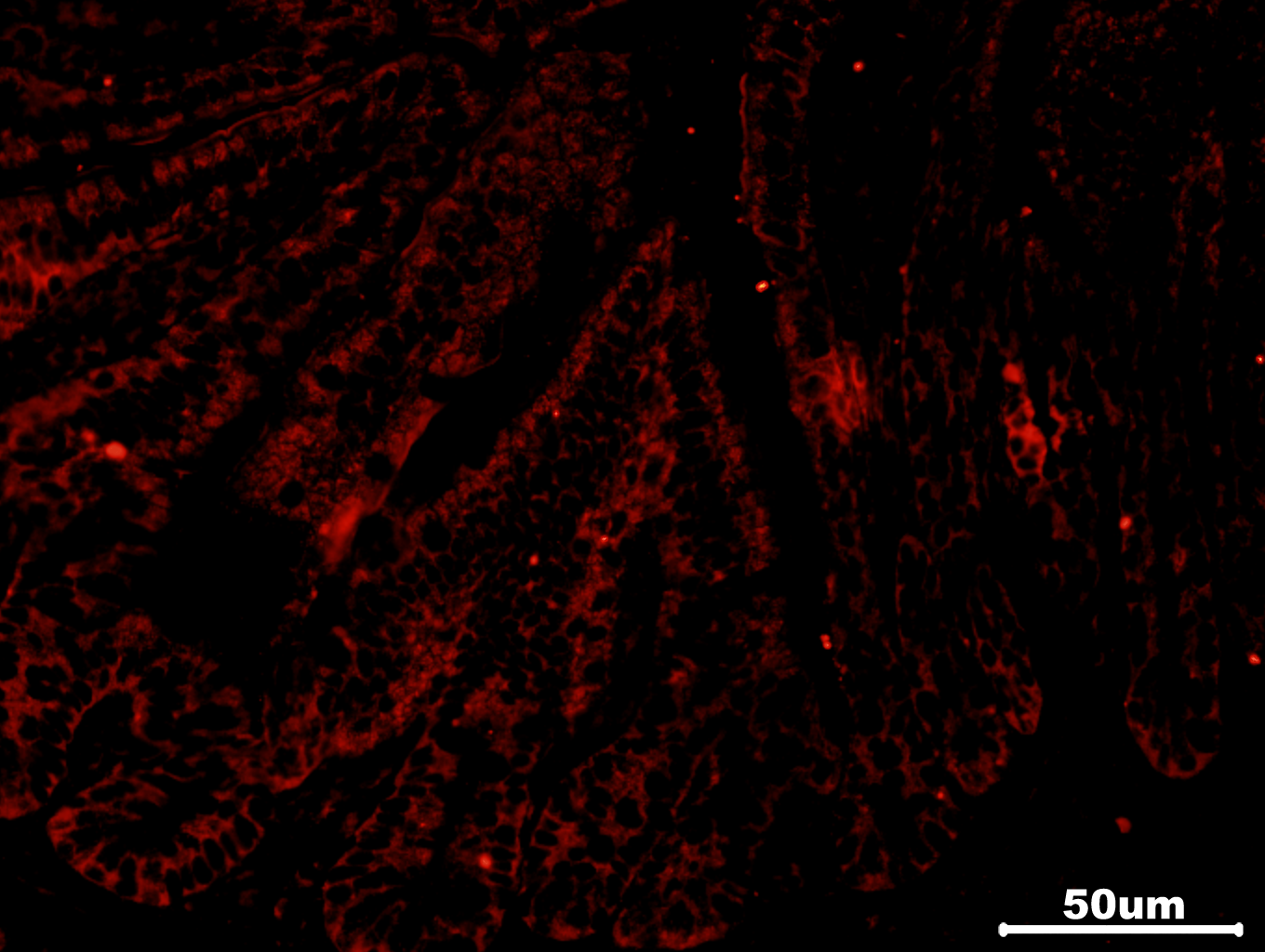

Supplement: Supplementary file 3 [file DataSheet_3.zip › A19-2-200-3-CD206.tif]

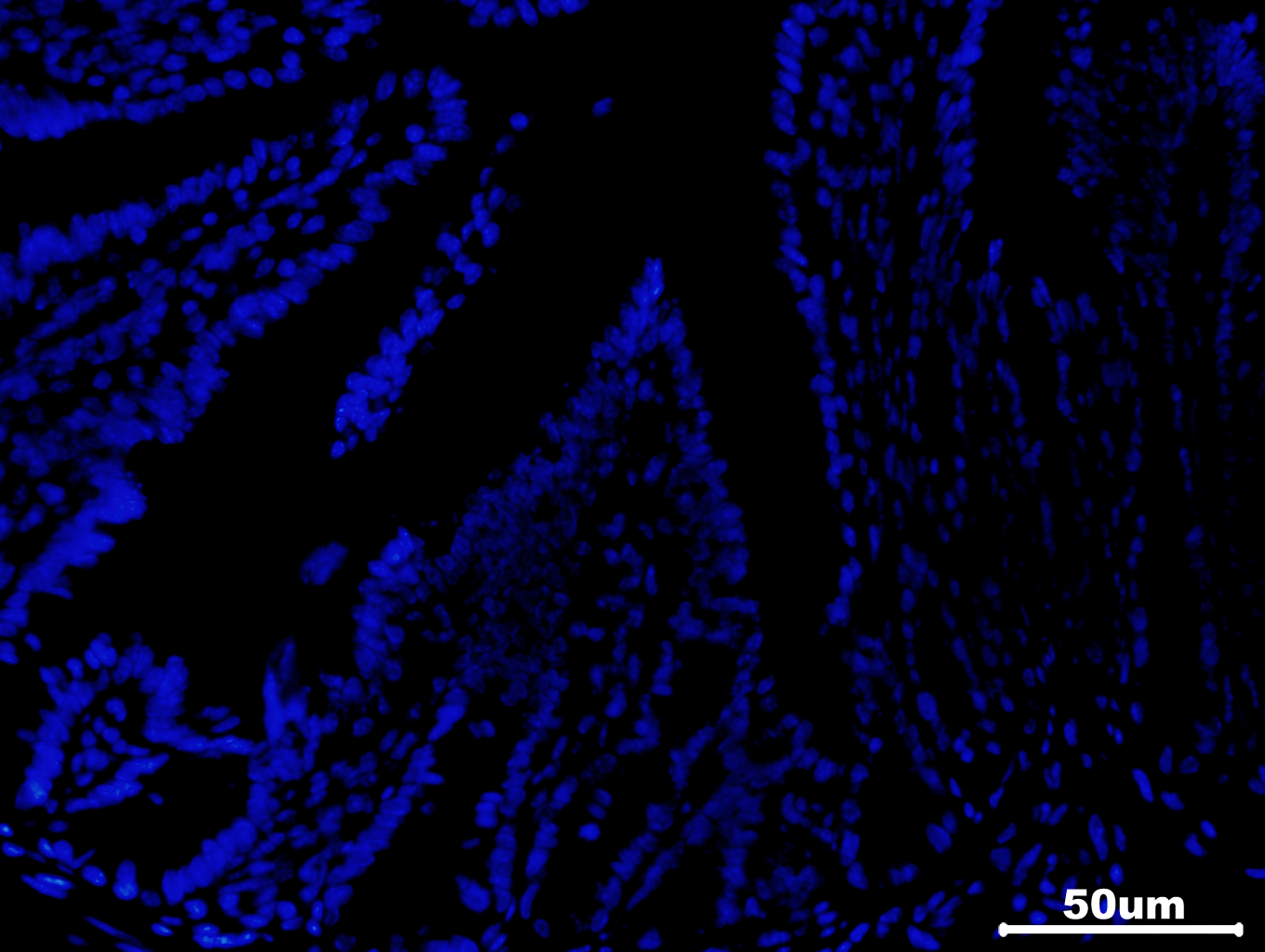

Supplement: Supplementary file 3 [file DataSheet_3.zip › A19-2-200-3-DAPI.tif]

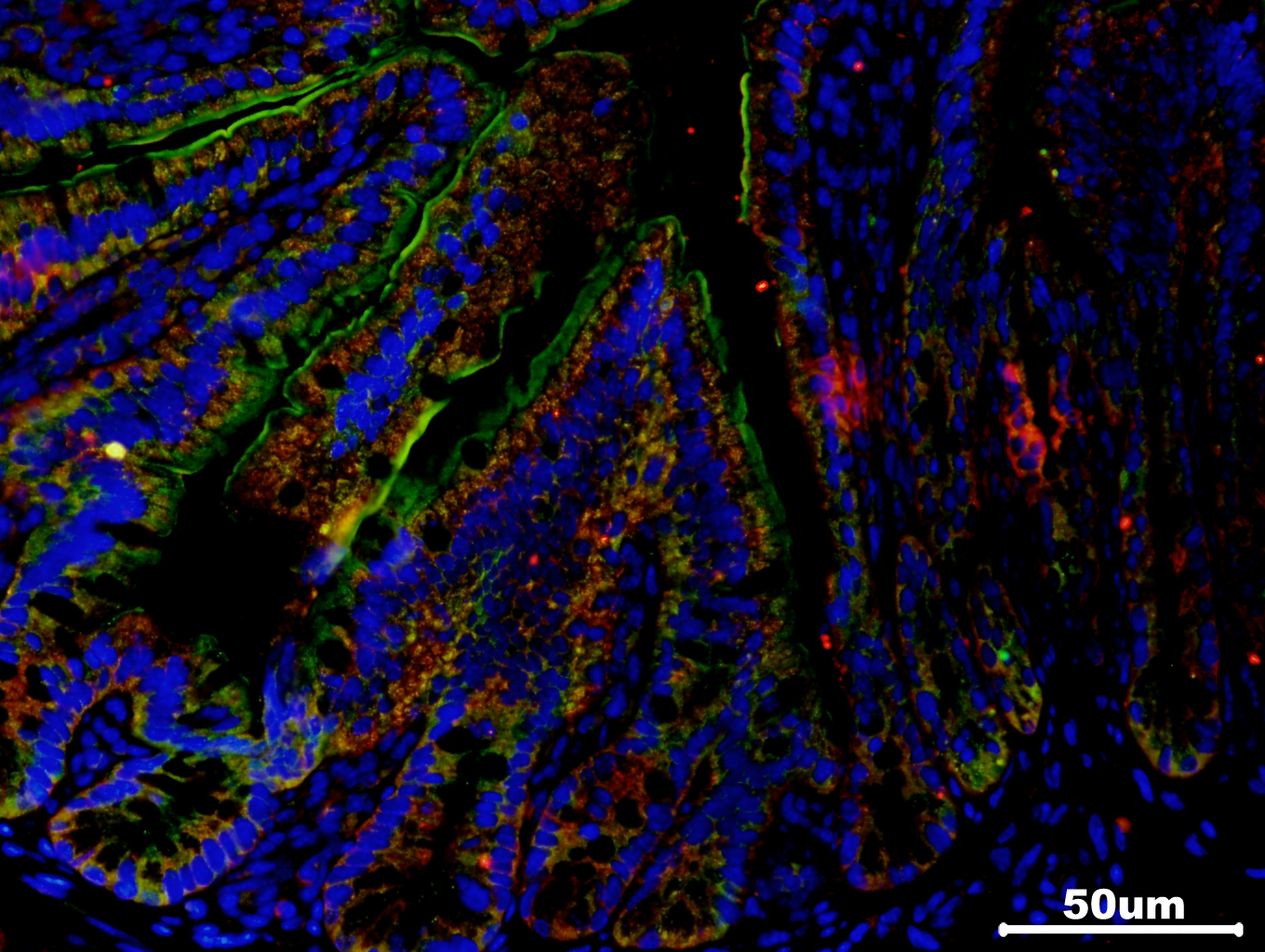

Supplement: Supplementary file 3 [file DataSheet_3.zip › A19-2-200-3-merge.tif]

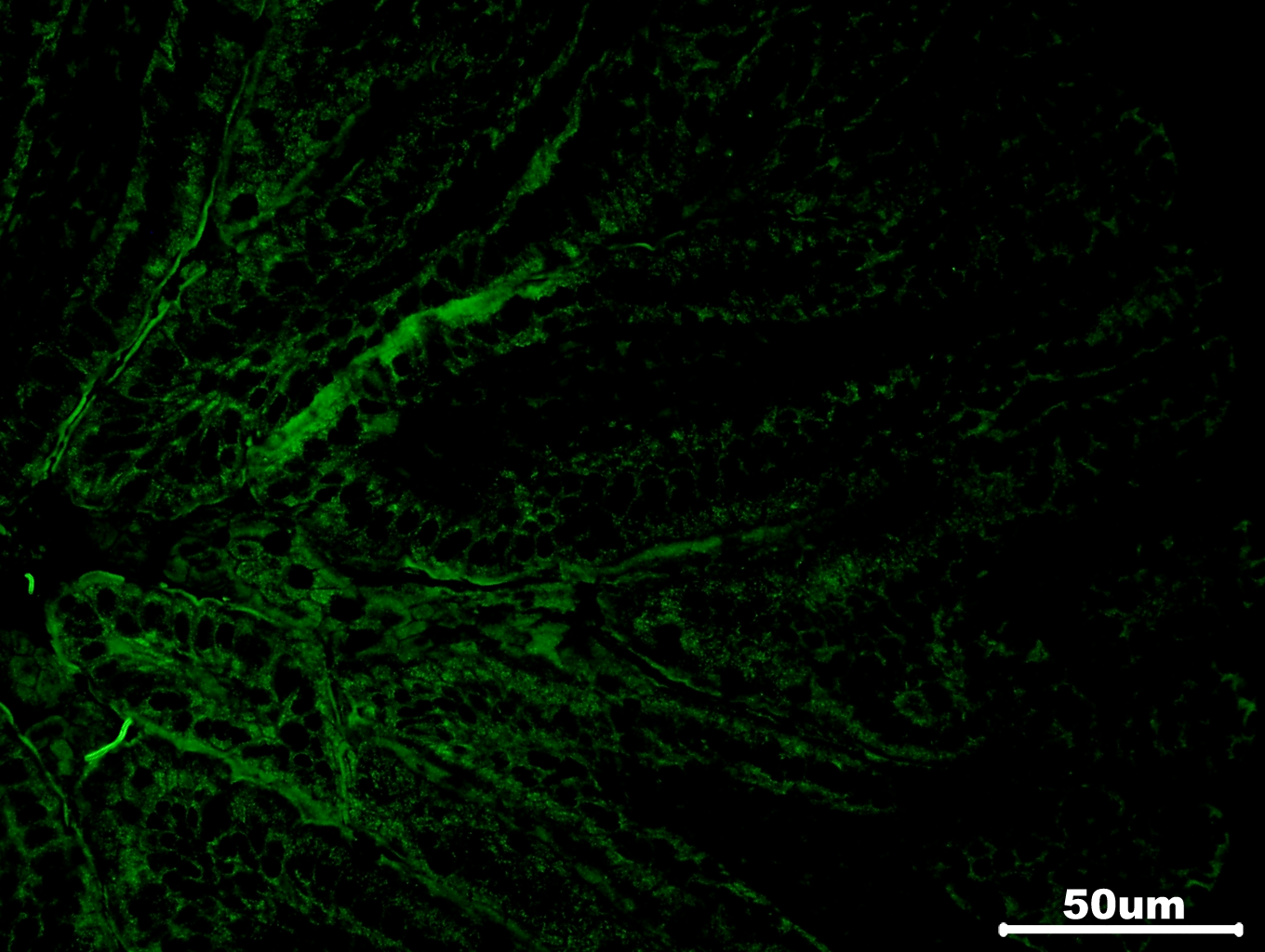

Supplement: Supplementary file 3 [file DataSheet_3.zip › A20-1-200-1-CD86.tif]

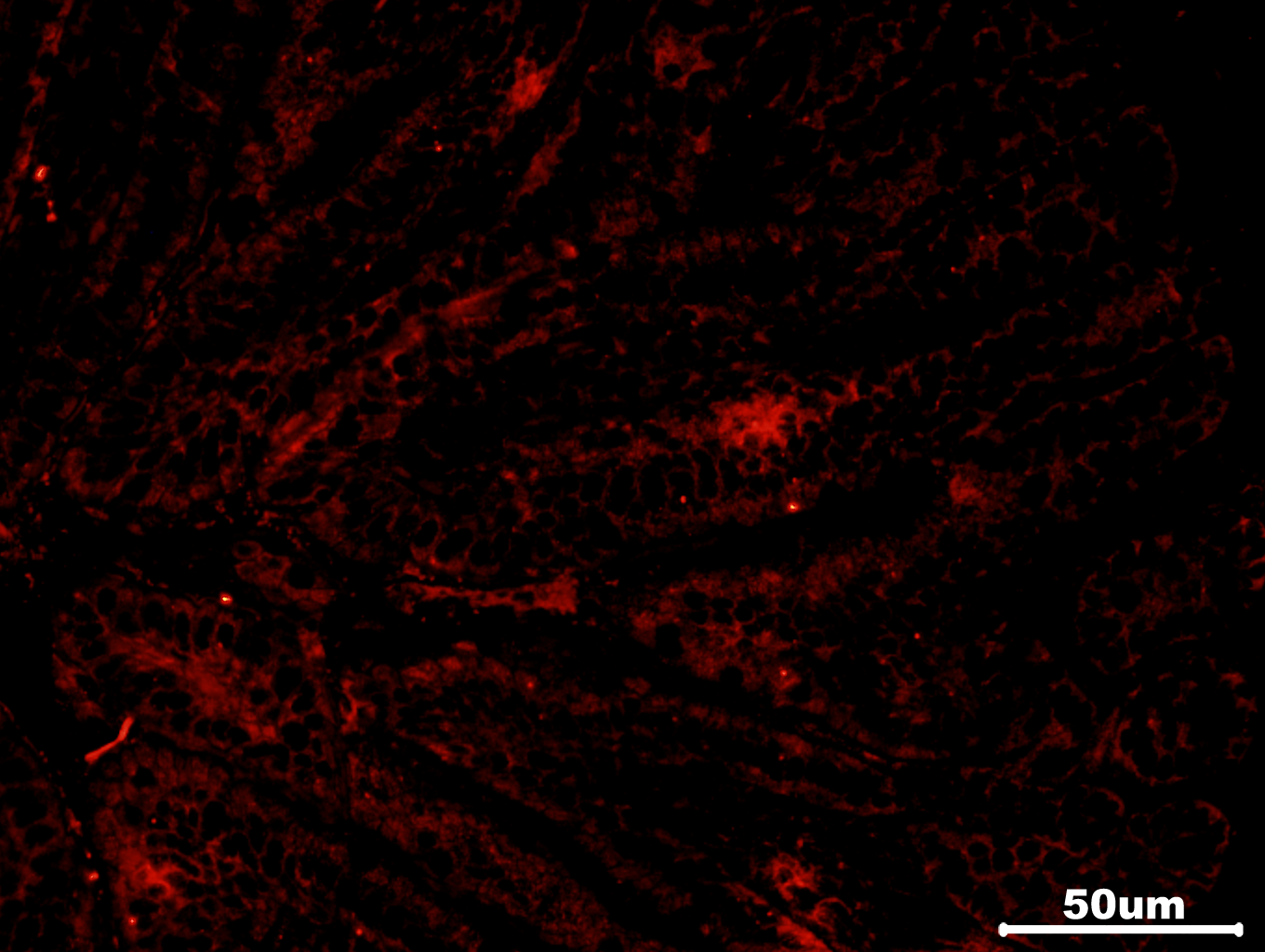

Supplement: Supplementary file 3 [file DataSheet_3.zip › A20-1-200-1-CD206.tif]

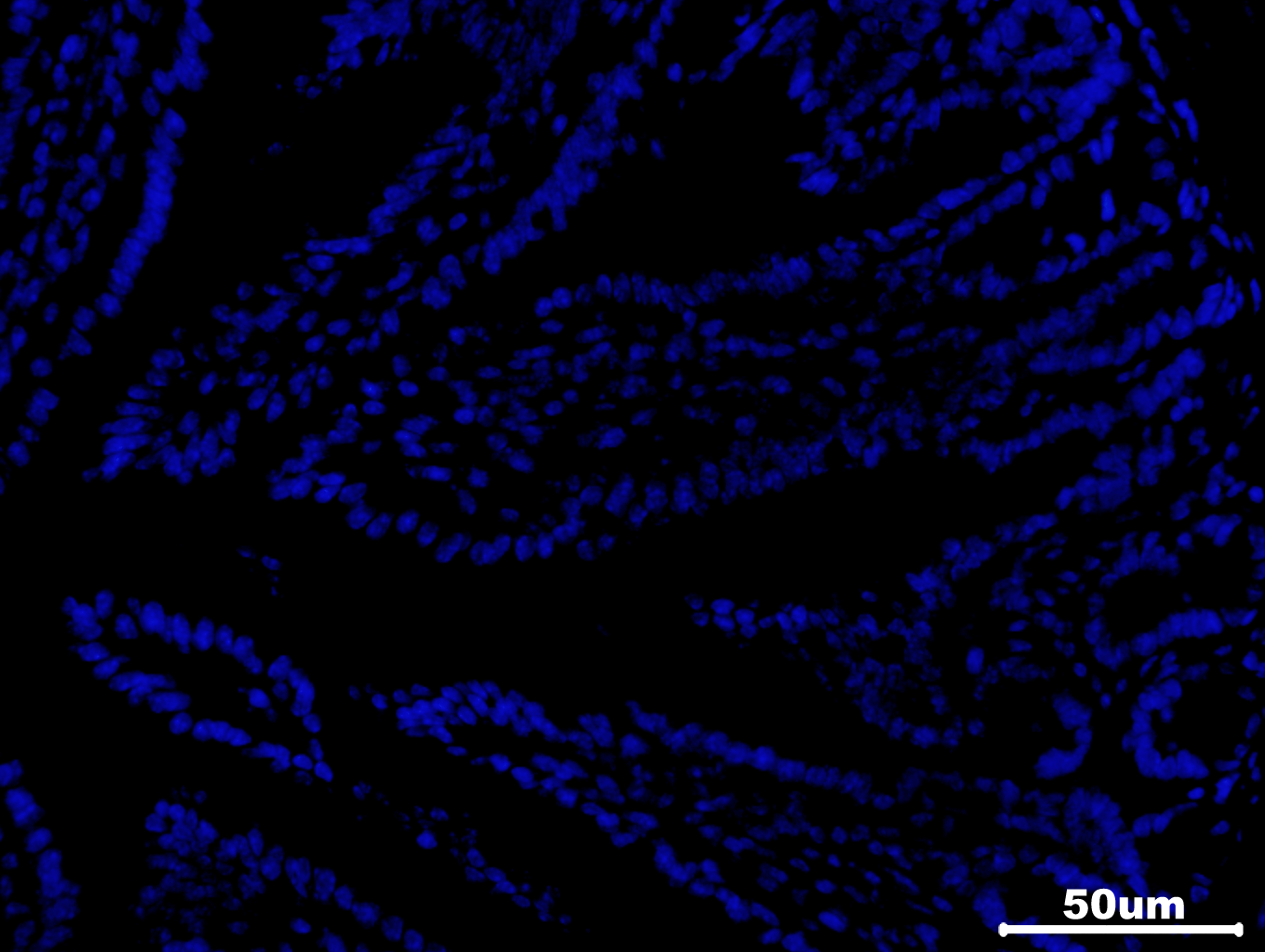

Supplement: Supplementary file 3 [file DataSheet_3.zip › A20-1-200-1-DAPI.tif]

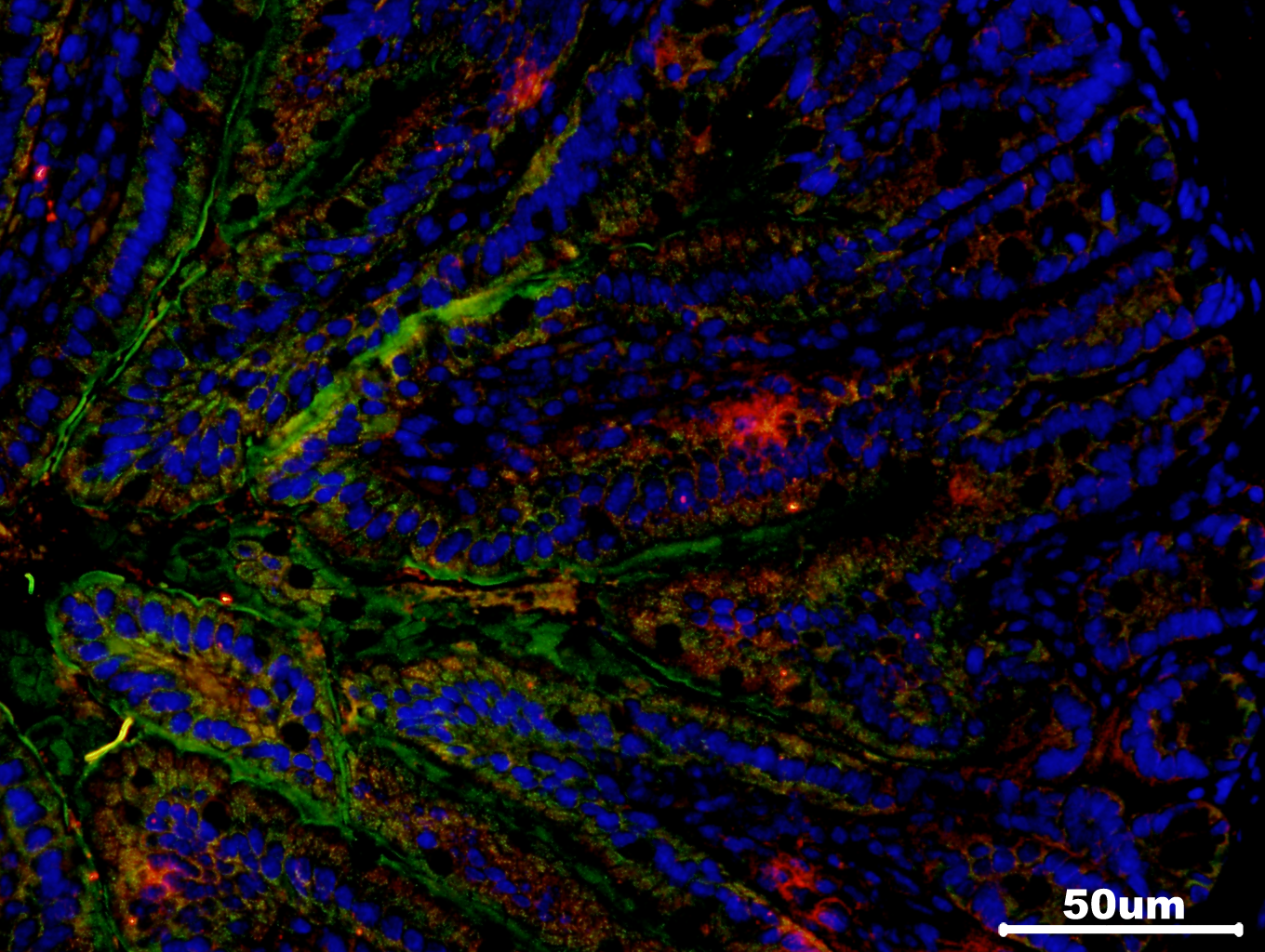

Supplement: Supplementary file 3 [file DataSheet_3.zip › A20-1-200-1-merge.tif]

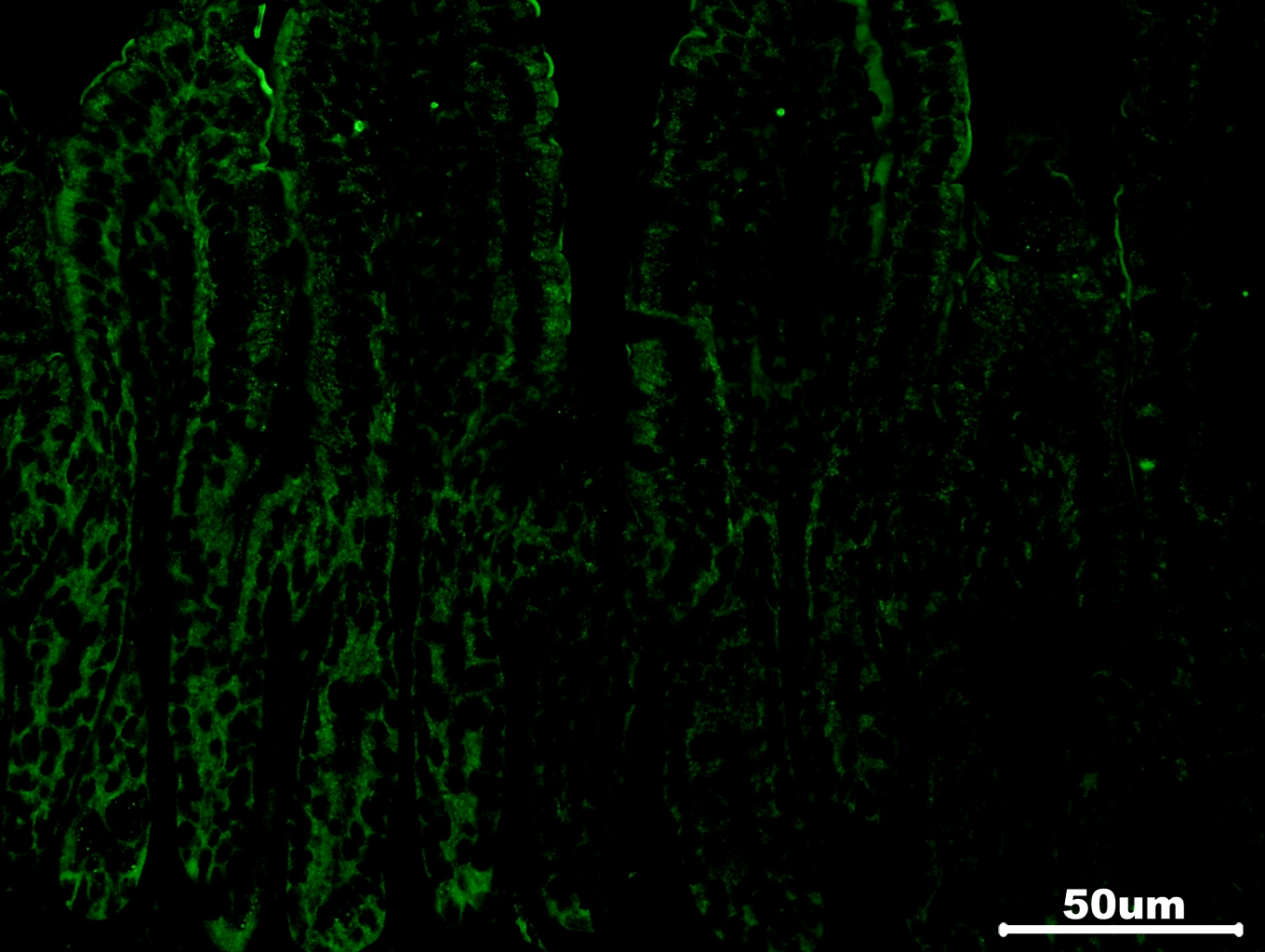

Supplement: Supplementary file 3 [file DataSheet_3.zip › A20-1-200-2-CD86.tif]

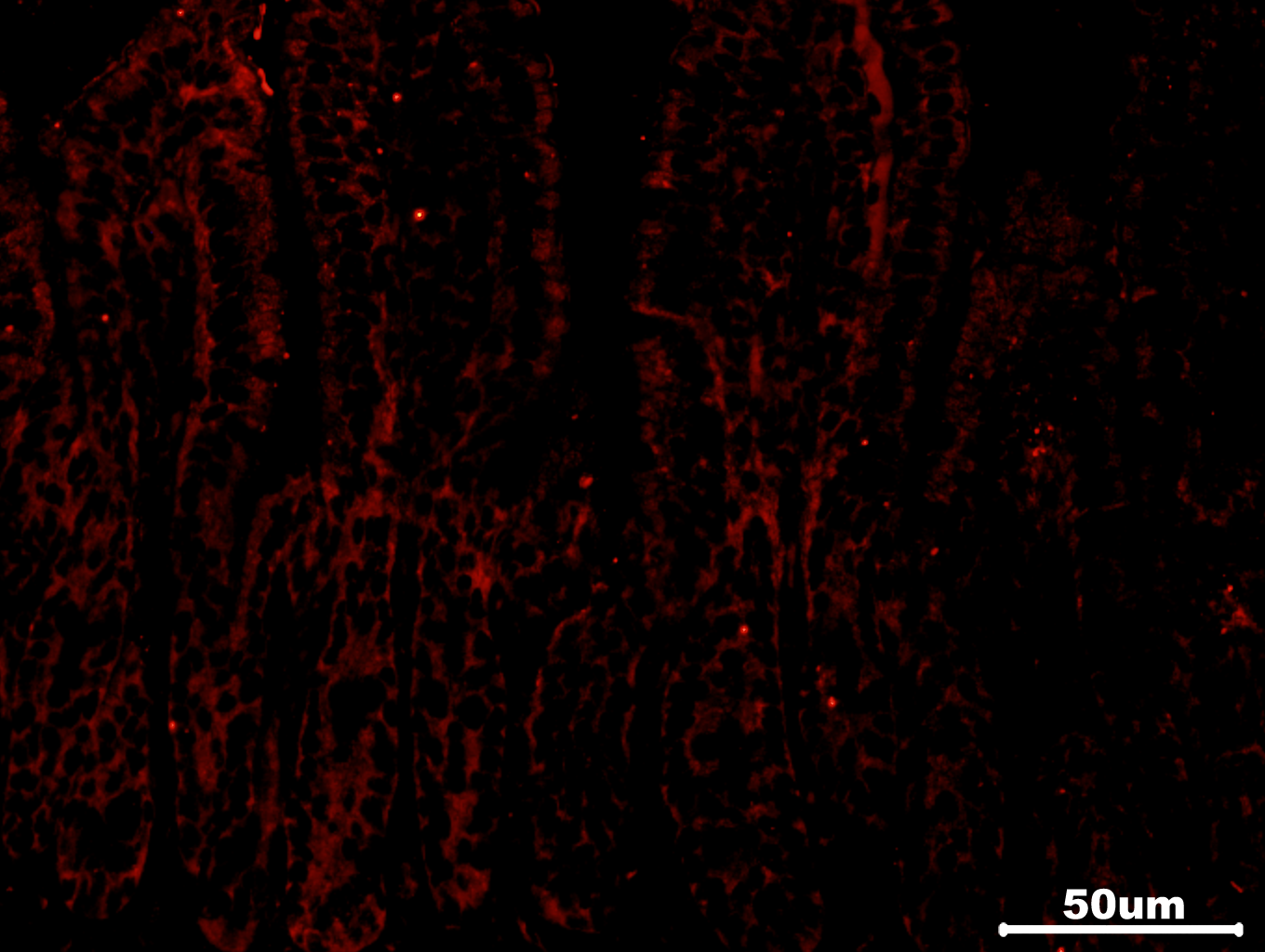

Supplement: Supplementary file 3 [file DataSheet_3.zip › A20-1-200-2-CD206.tif]

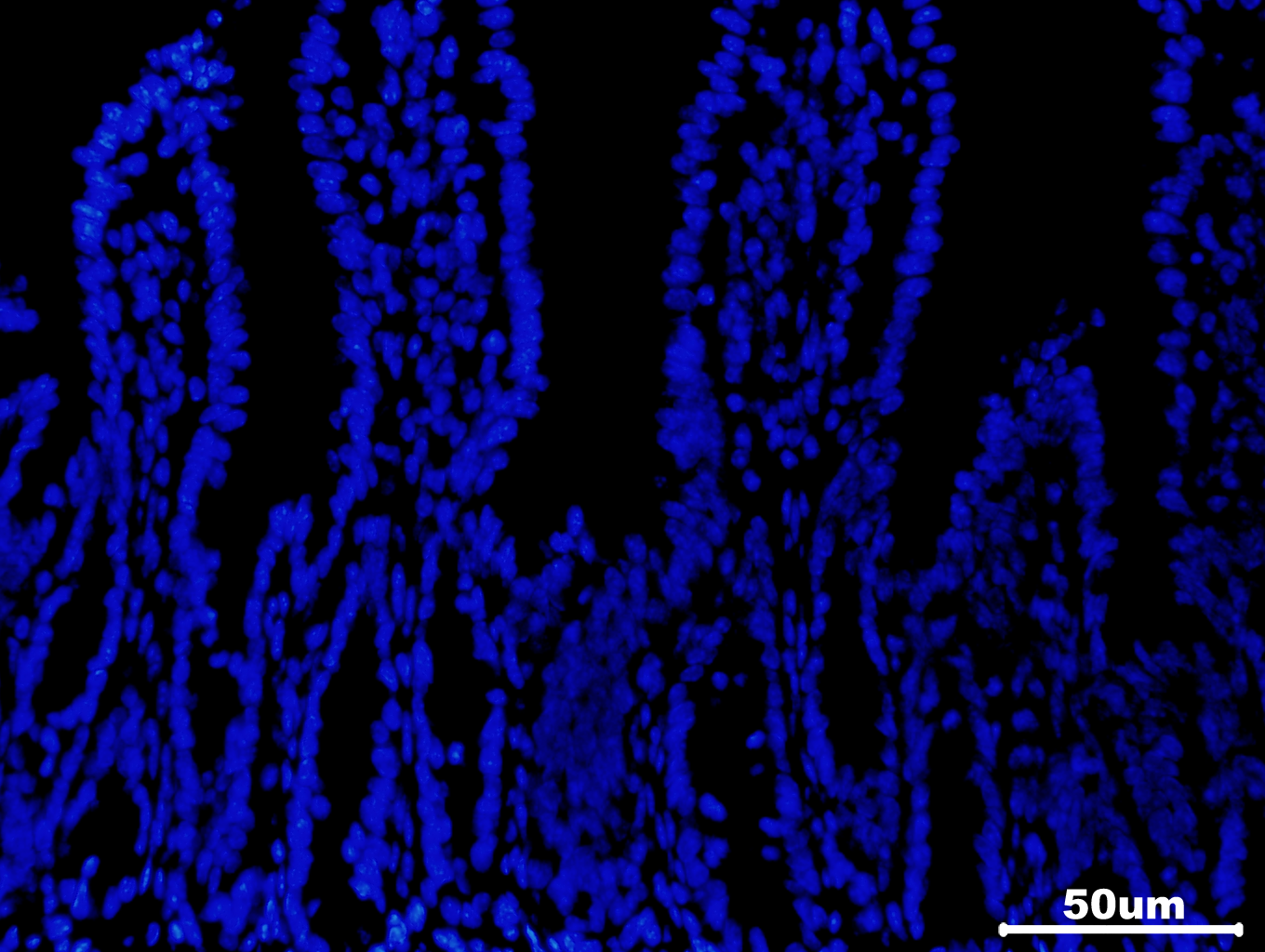

Supplement: Supplementary file 3 [file DataSheet_3.zip › A20-1-200-2-DAPI.tif]

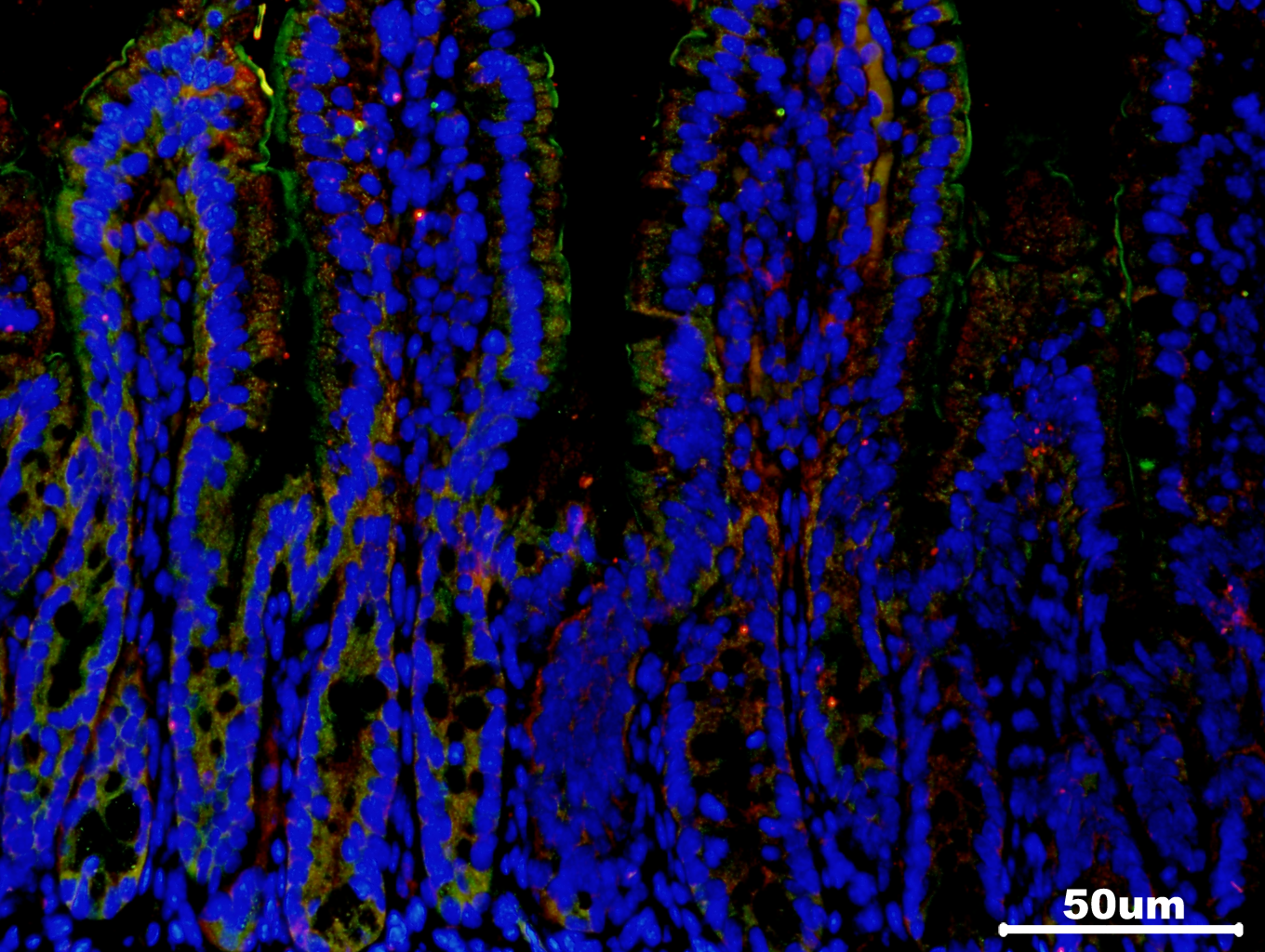

Supplement: Supplementary file 3 [file DataSheet_3.zip › A20-1-200-2-merge.tif]

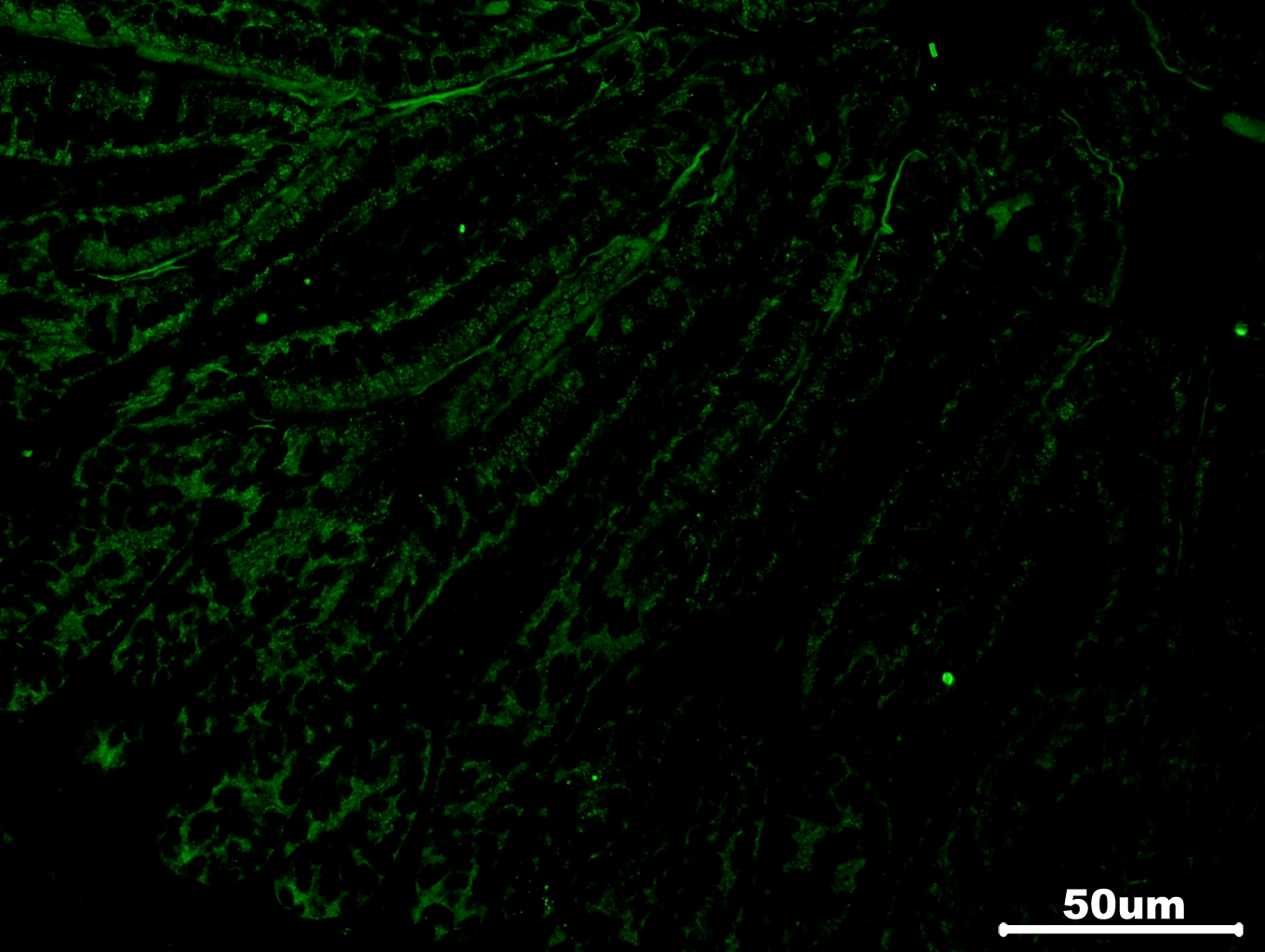

Supplement: Supplementary file 3 [file DataSheet_3.zip › A20-1-200-3-CD86.tif]

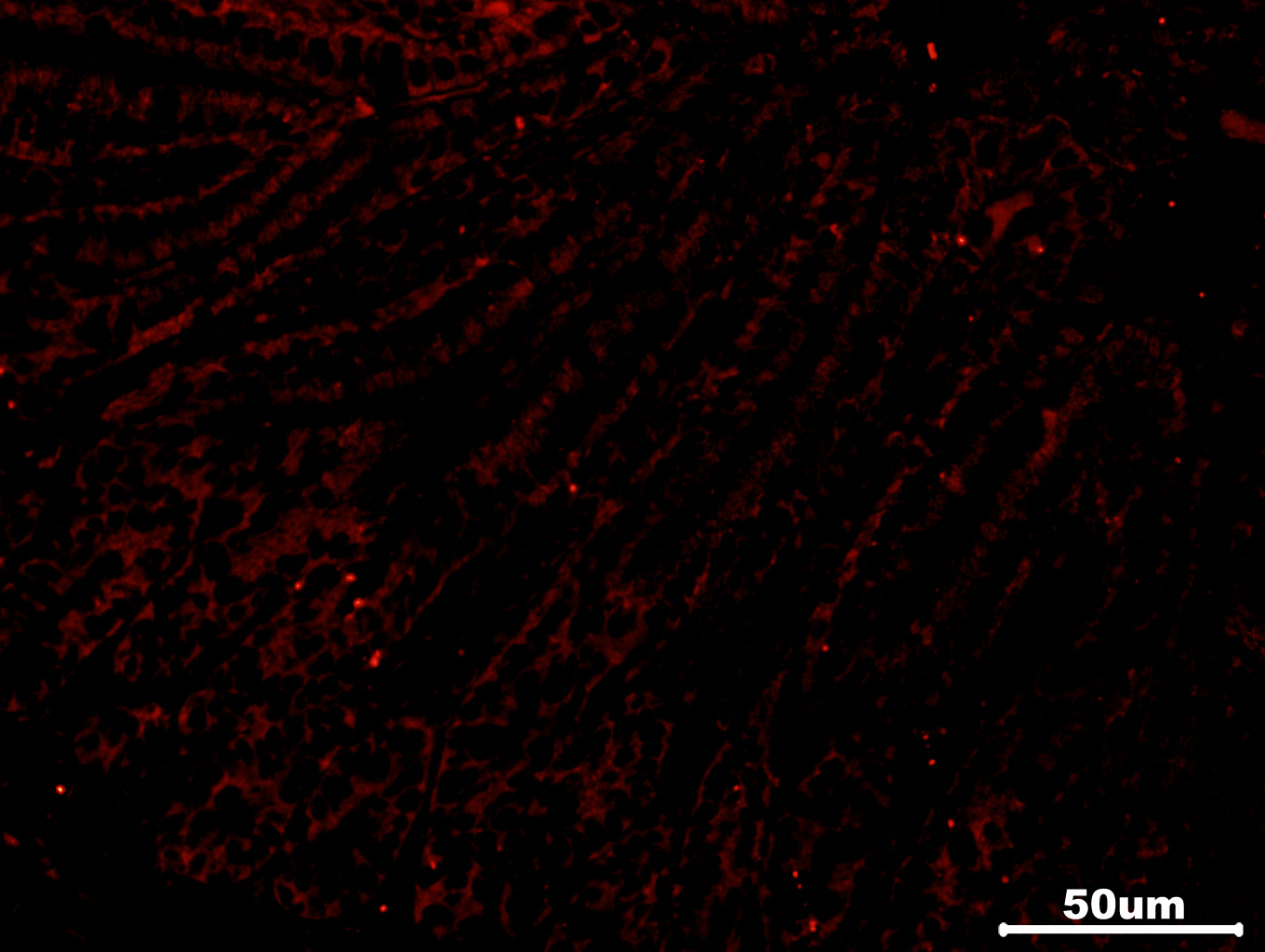

Supplement: Supplementary file 3 [file DataSheet_3.zip › A20-1-200-3-CD206.tif]

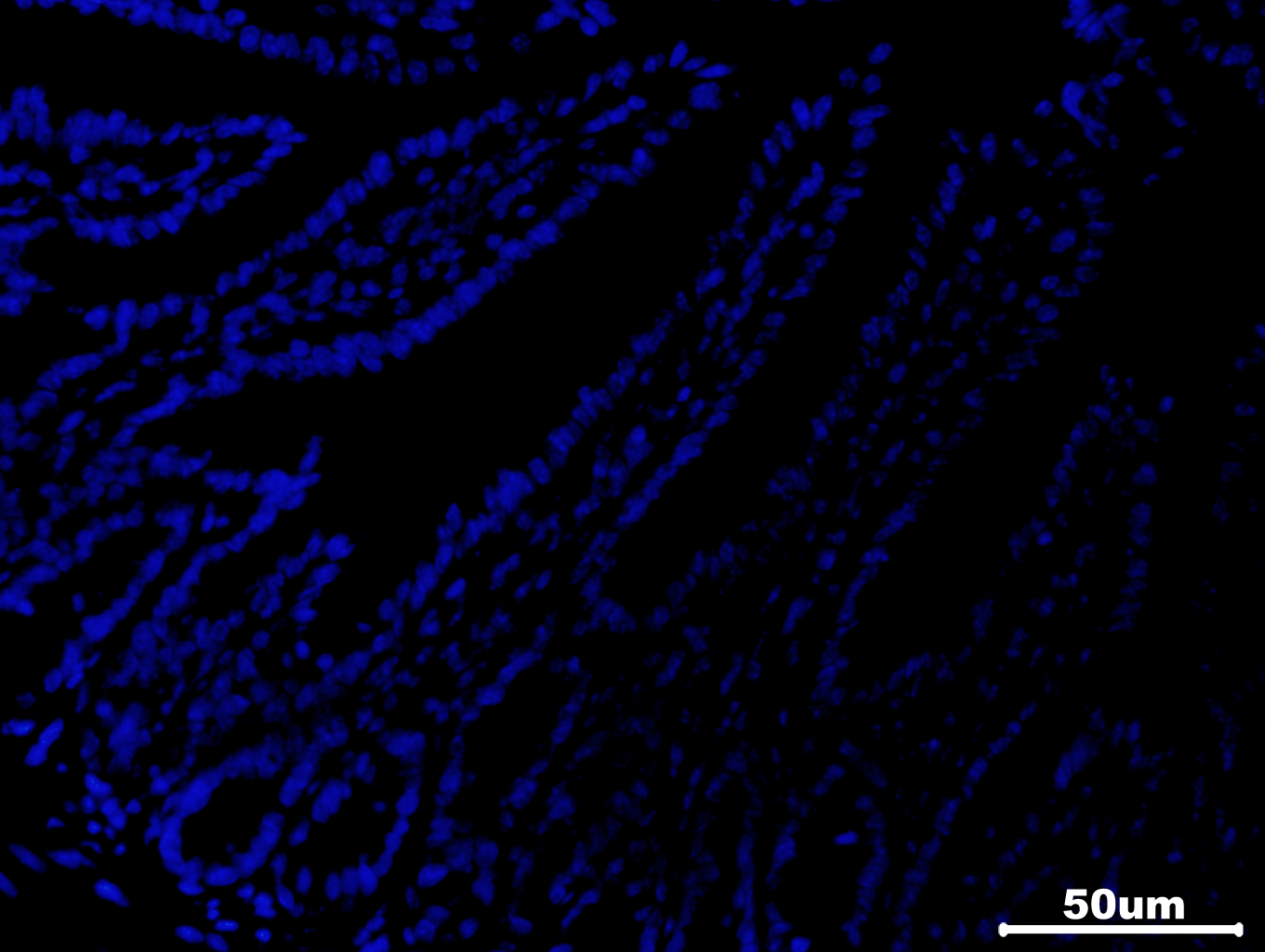

Supplement: Supplementary file 3 [file DataSheet_3.zip › A20-1-200-3-DAPI.tif]

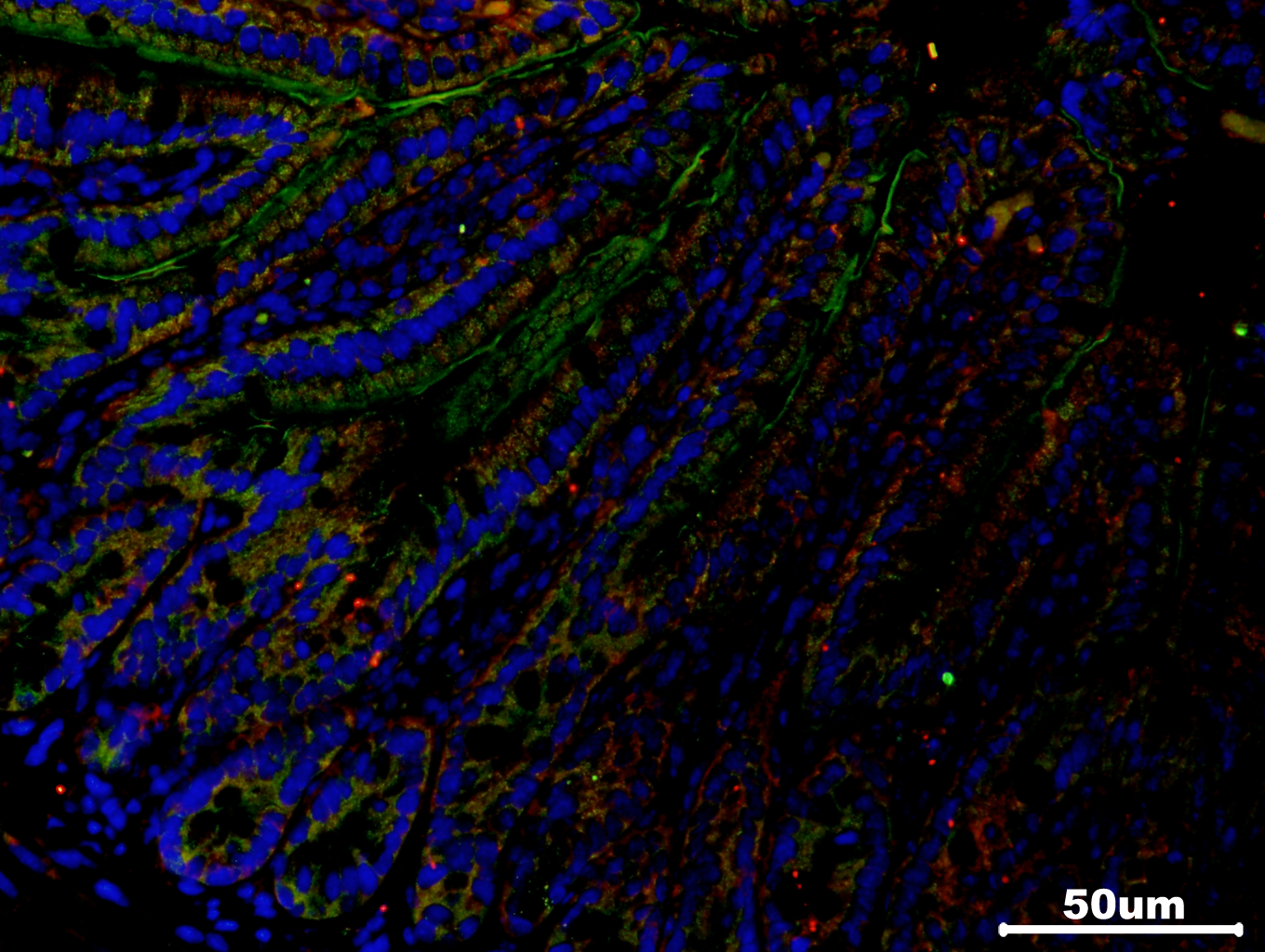

Supplement: Supplementary file 3 [file DataSheet_3.zip › A20-1-200-3-merge.tif]

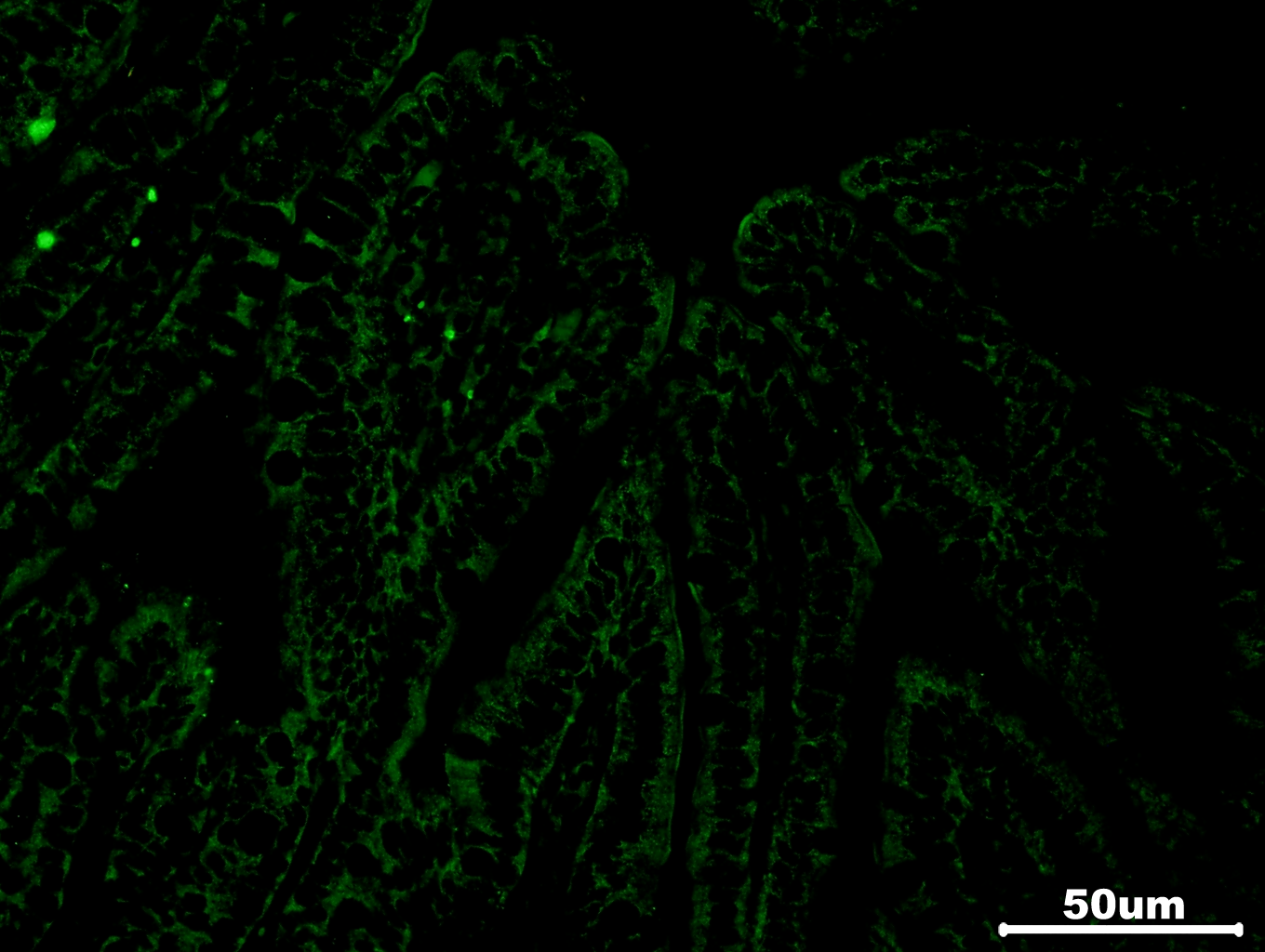

Supplement: Supplementary file 4 [file DataSheet_4.zip › E31-1-200-1-CD86.tif]

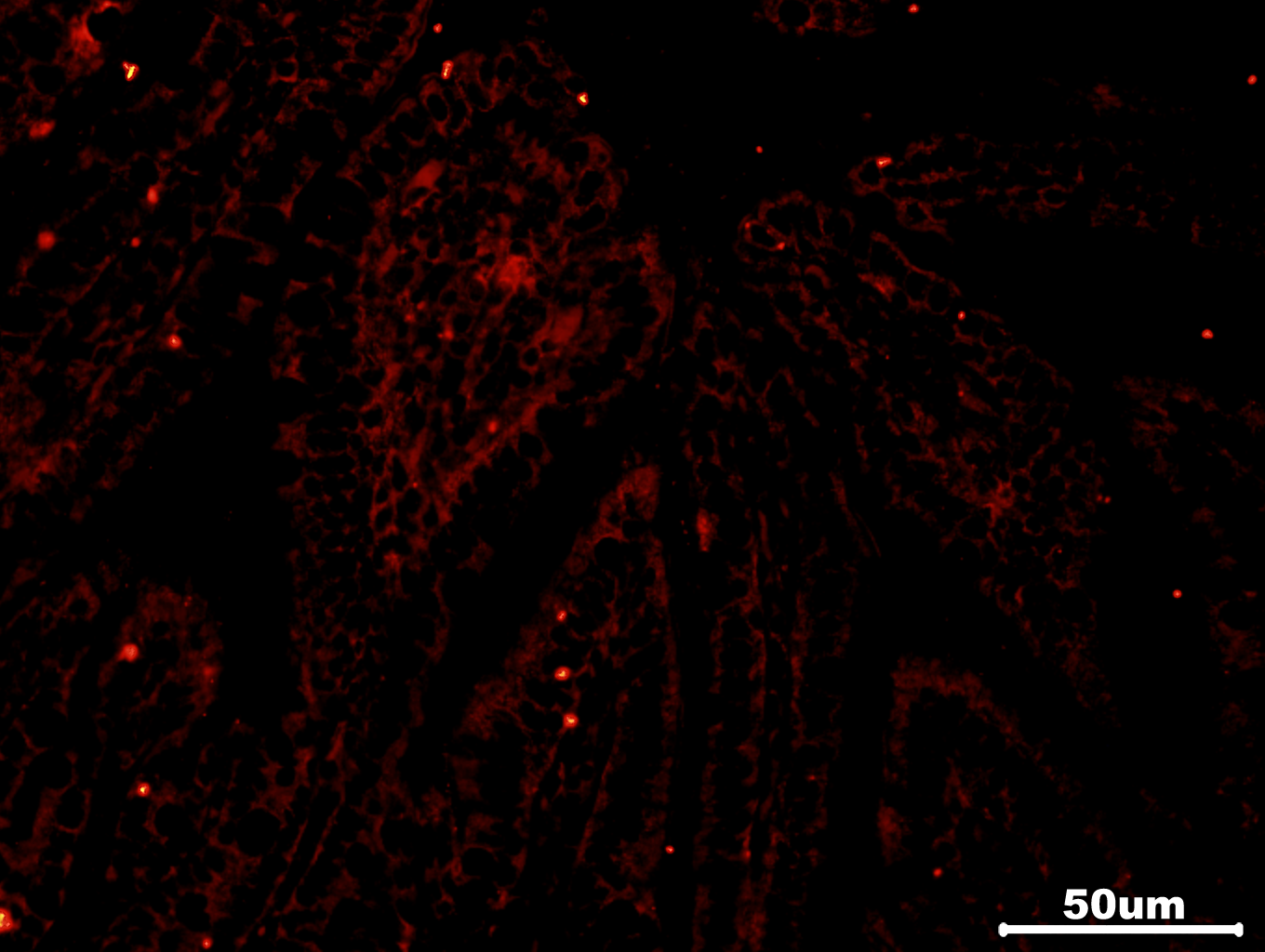

Supplement: Supplementary file 4 [file DataSheet_4.zip › E31-1-200-1-CD206.tif]

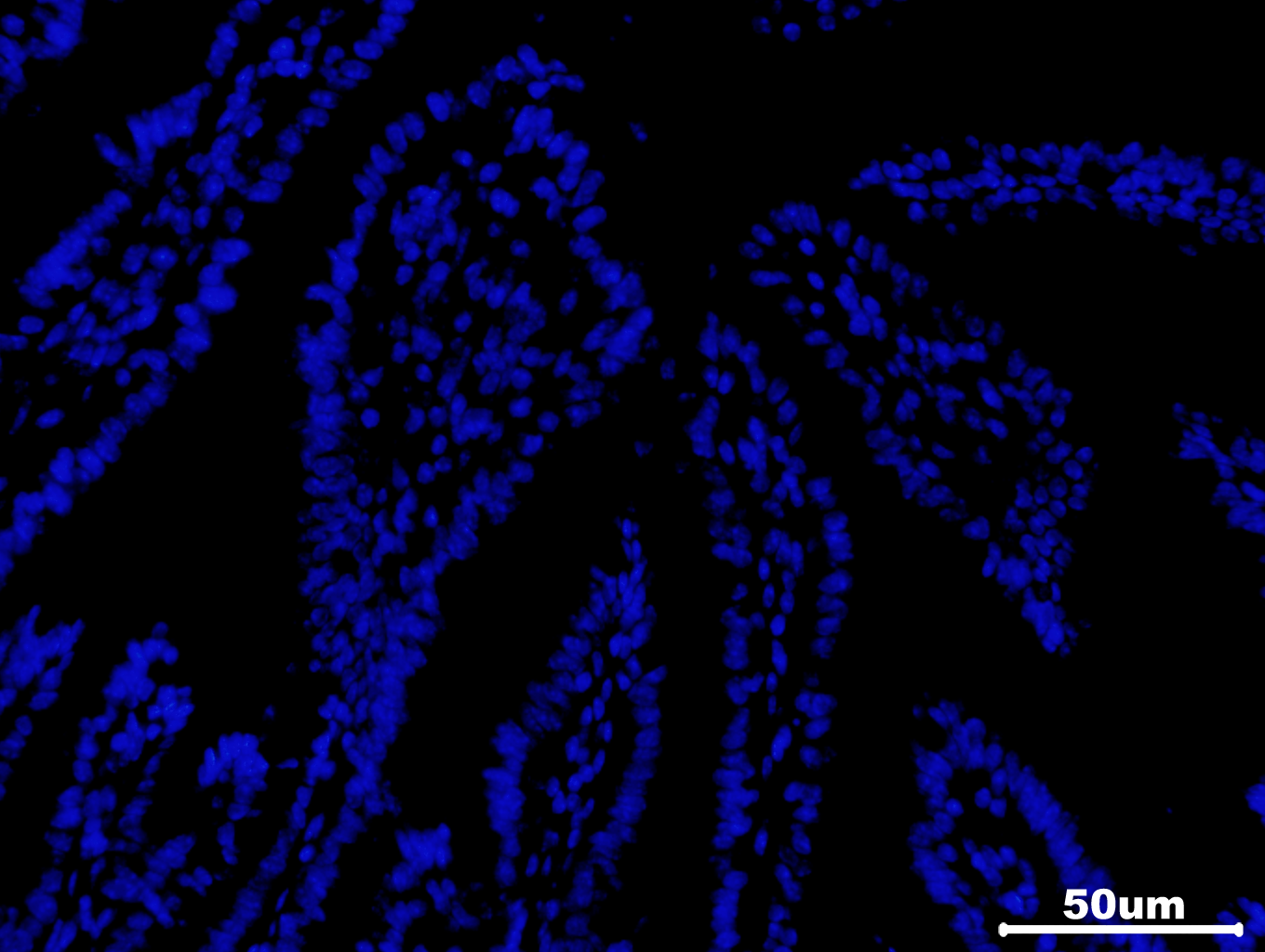

Supplement: Supplementary file 4 [file DataSheet_4.zip › E31-1-200-1-DAPI.tif]

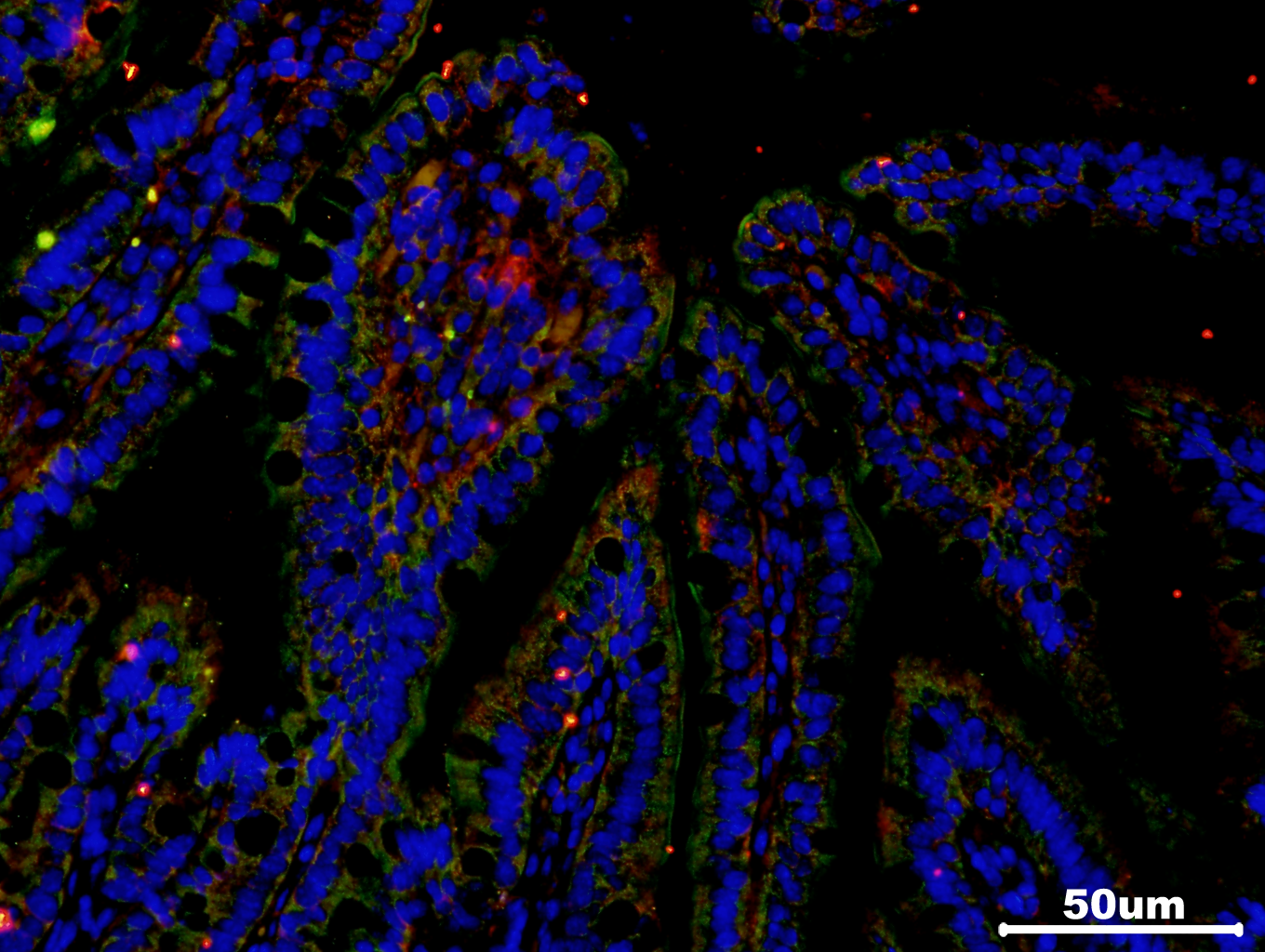

Supplement: Supplementary file 4 [file DataSheet_4.zip › E31-1-200-1-merge.tif]

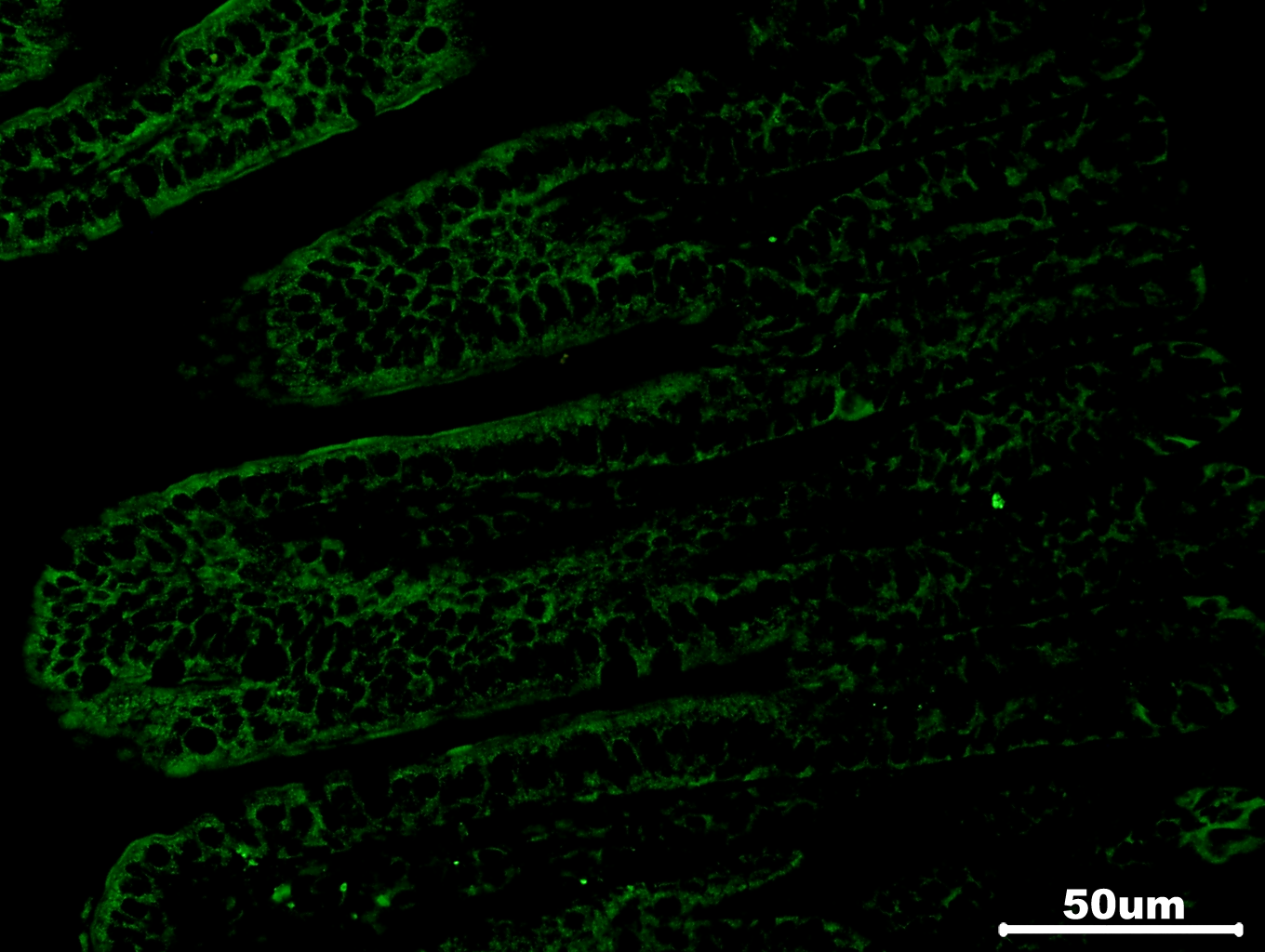

Supplement: Supplementary file 4 [file DataSheet_4.zip › E31-1-200-2-CD86.tif]

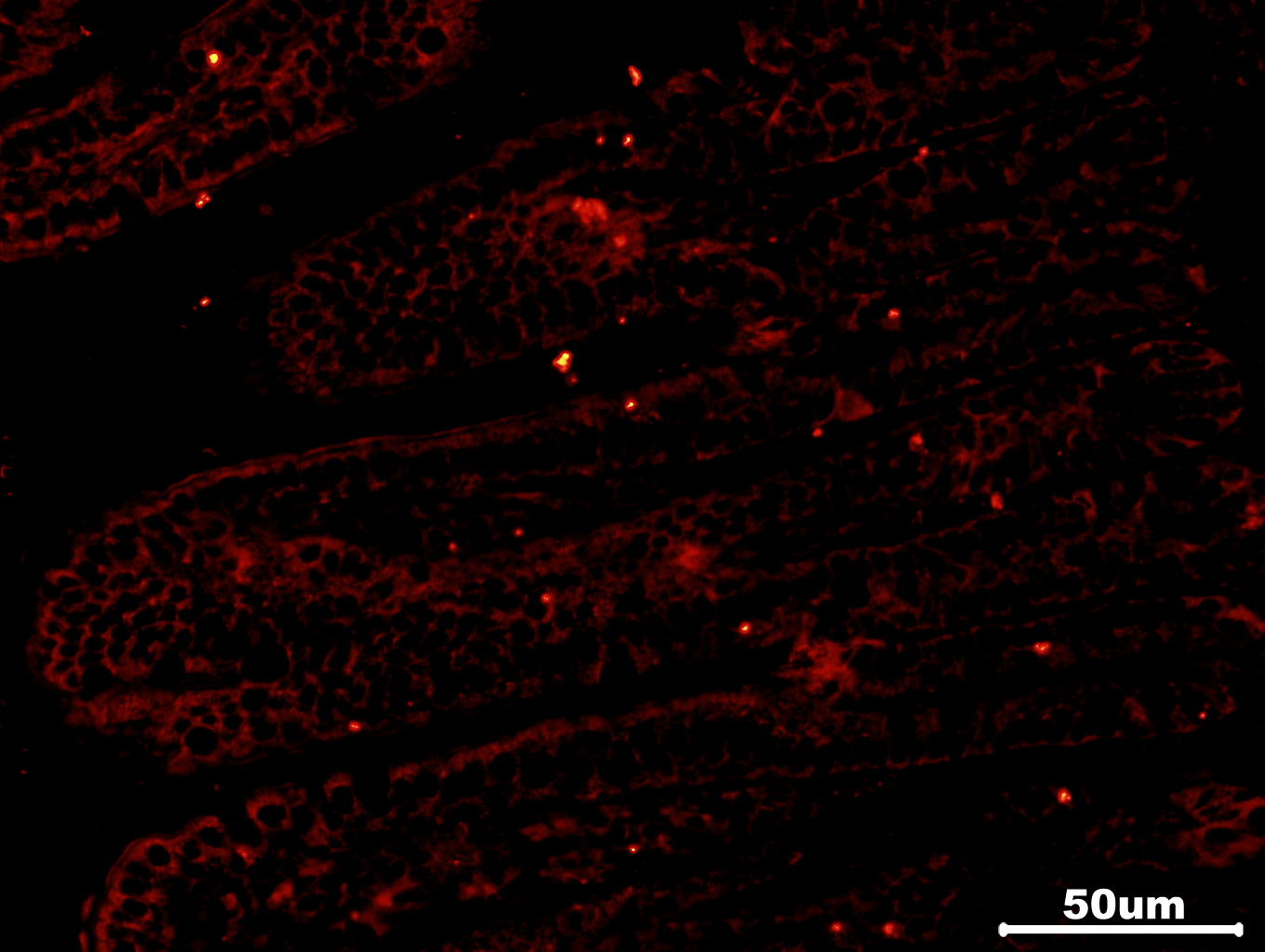

Supplement: Supplementary file 4 [file DataSheet_4.zip › E31-1-200-2-CD206.tif]

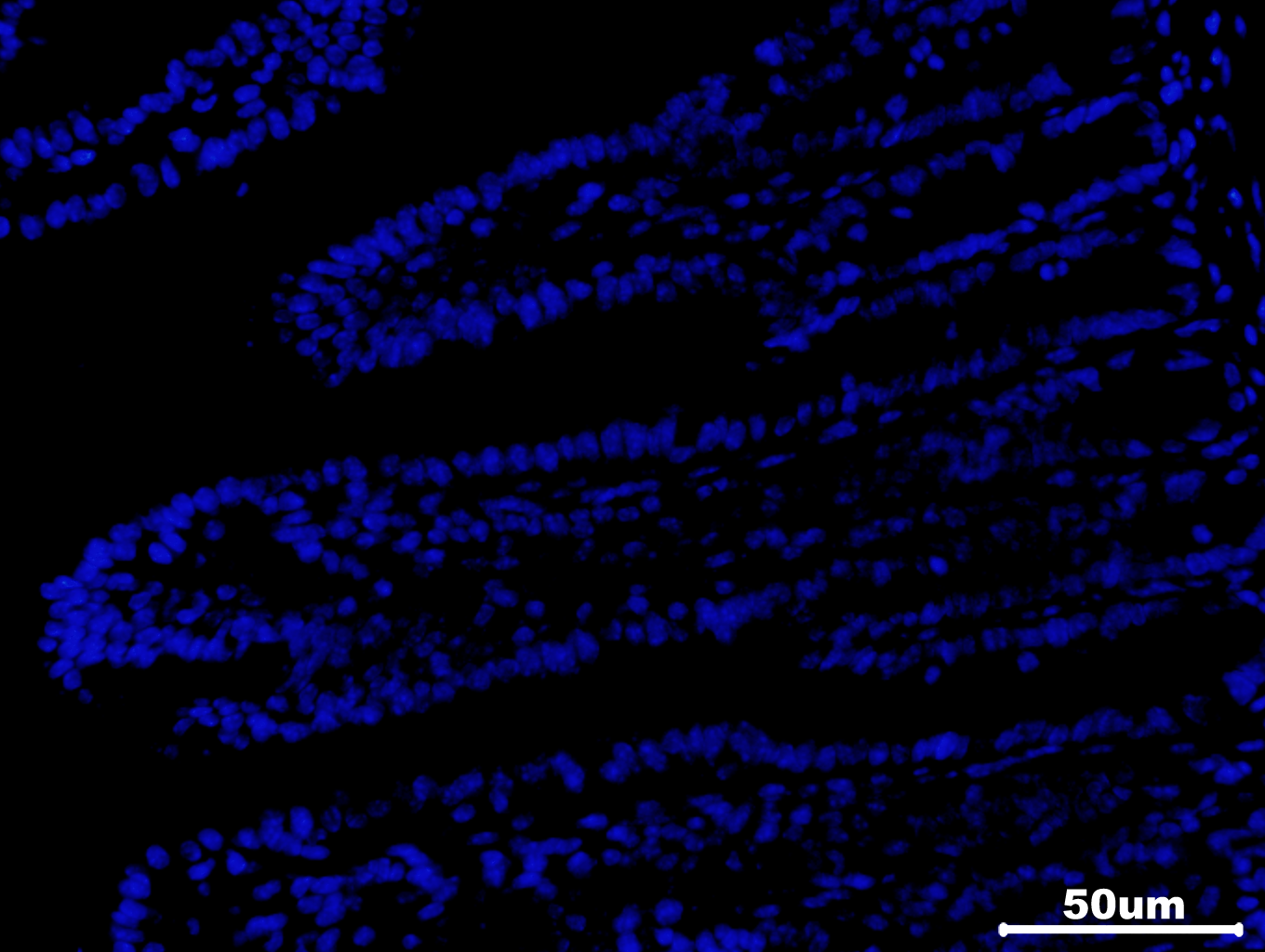

Supplement: Supplementary file 4 [file DataSheet_4.zip › E31-1-200-2-DAPI.tif]

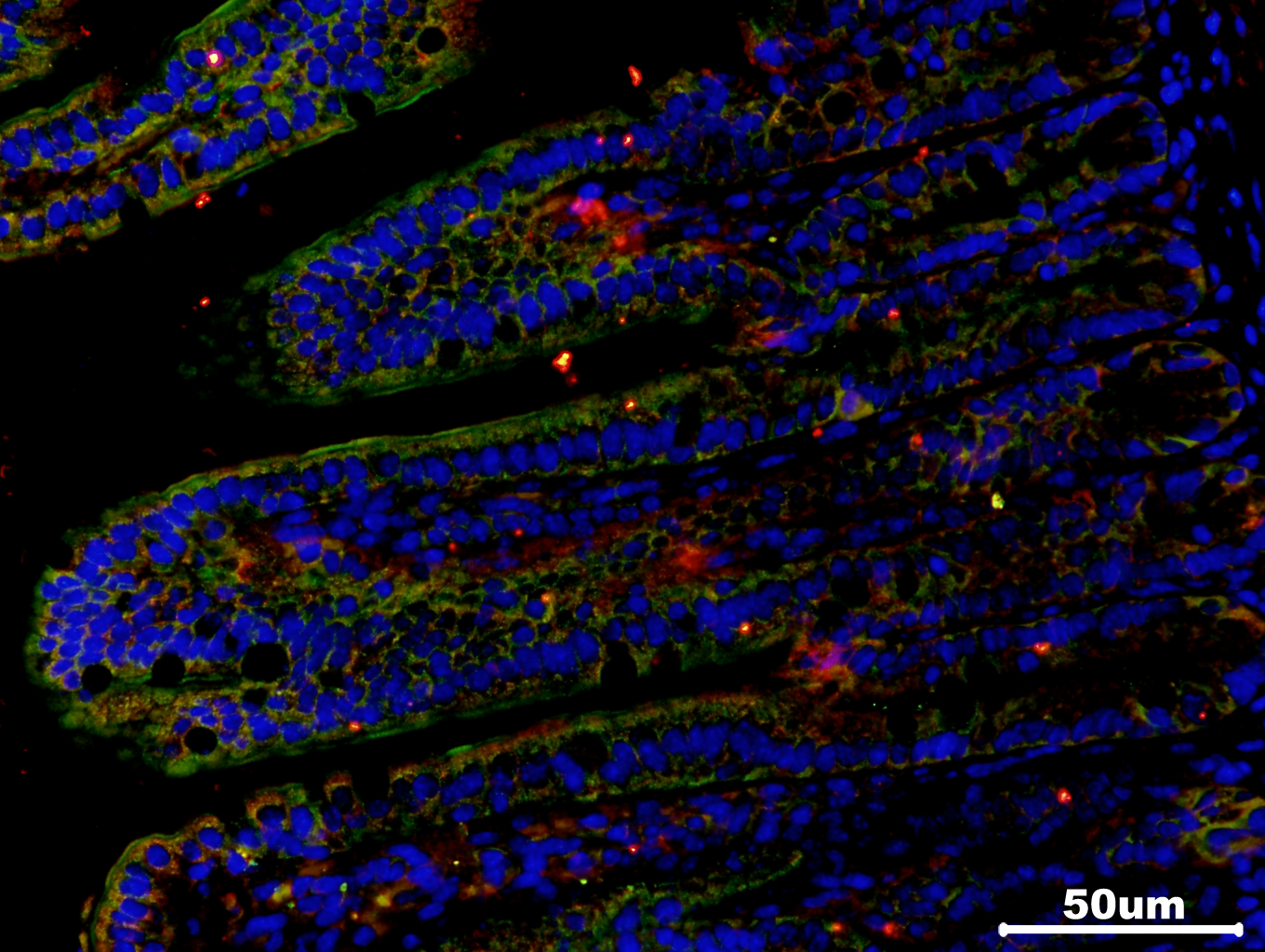

Supplement: Supplementary file 4 [file DataSheet_4.zip › E31-1-200-2-merge.tif]

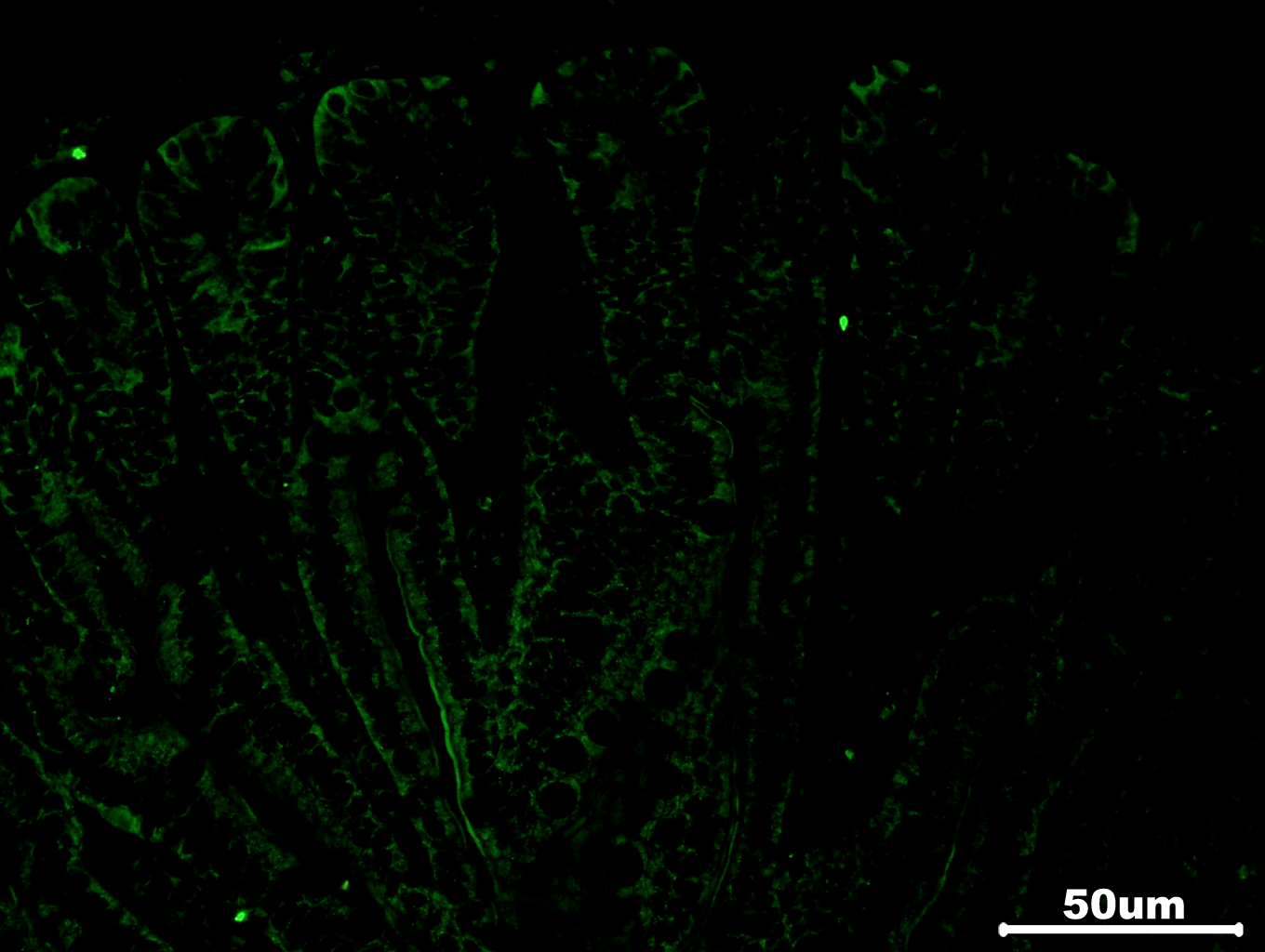

Supplement: Supplementary file 4 [file DataSheet_4.zip › E31-1-200-3-CD86.tif]

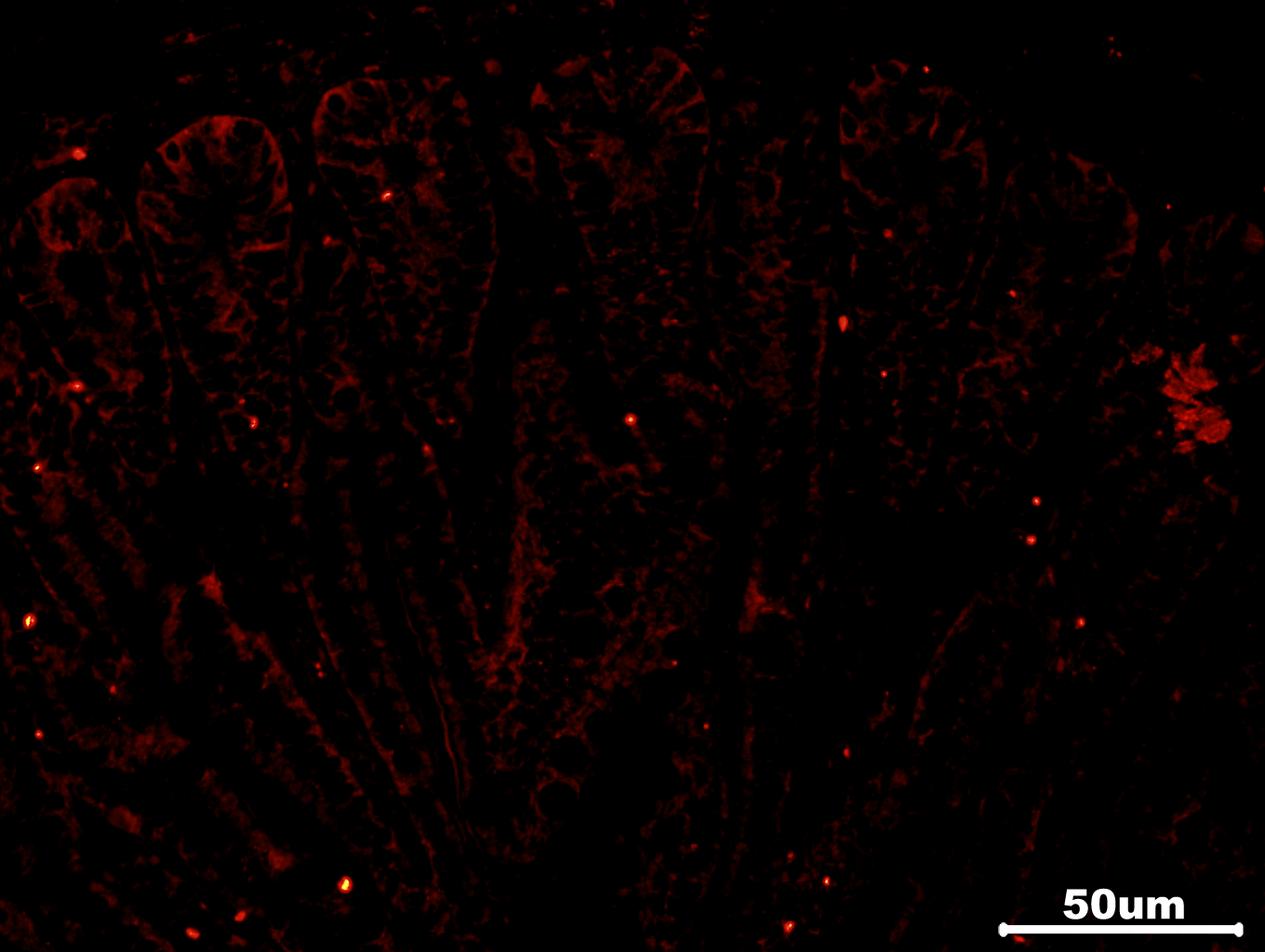

Supplement: Supplementary file 4 [file DataSheet_4.zip › E31-1-200-3-CD206.tif]

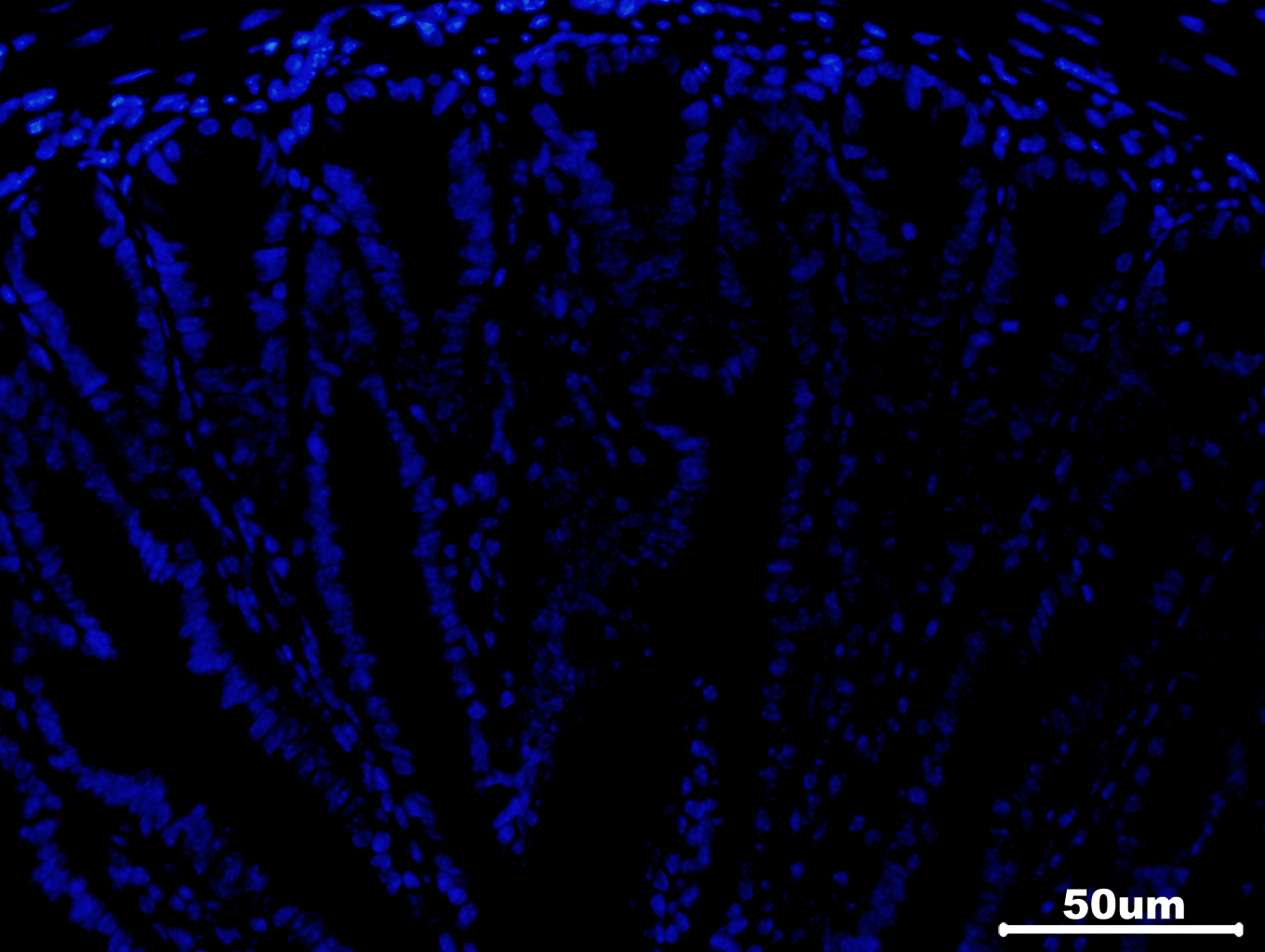

Supplement: Supplementary file 4 [file DataSheet_4.zip › E31-1-200-3-DAPI.tif]

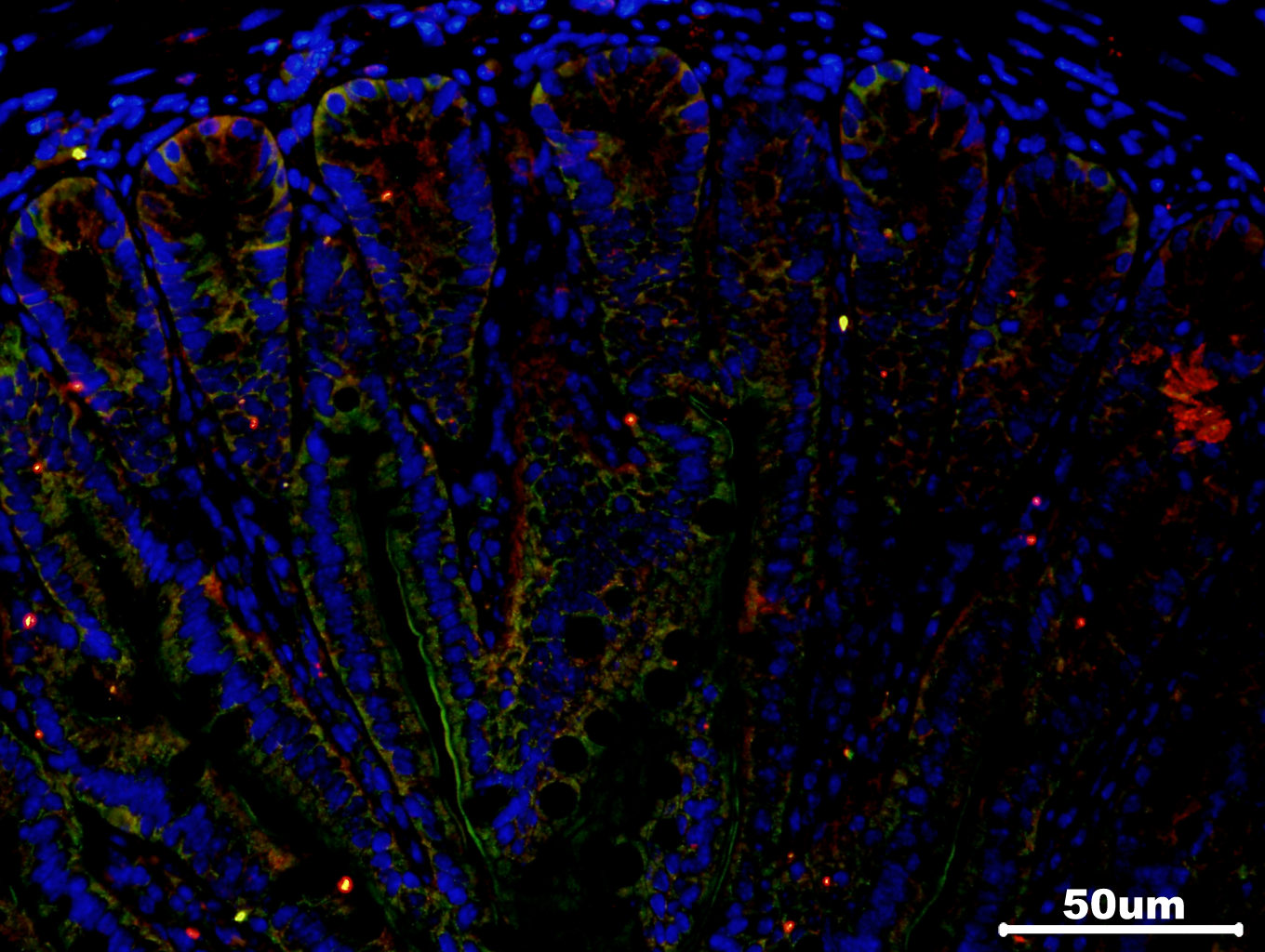

Supplement: Supplementary file 4 [file DataSheet_4.zip › E31-1-200-3-merge.tif]

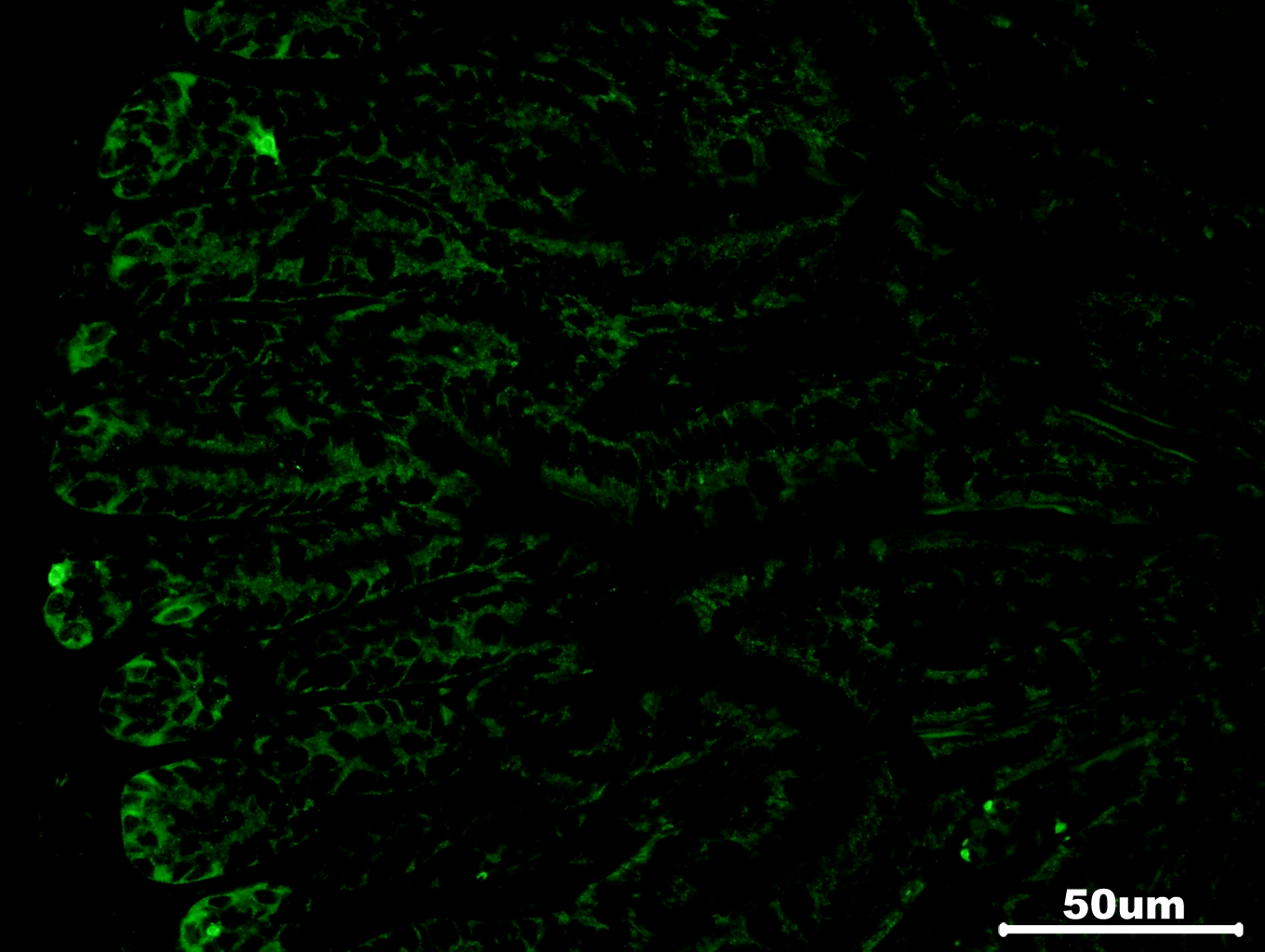

Supplement: Supplementary file 4 [file DataSheet_4.zip › E31-2-200-1-CD86.tif]

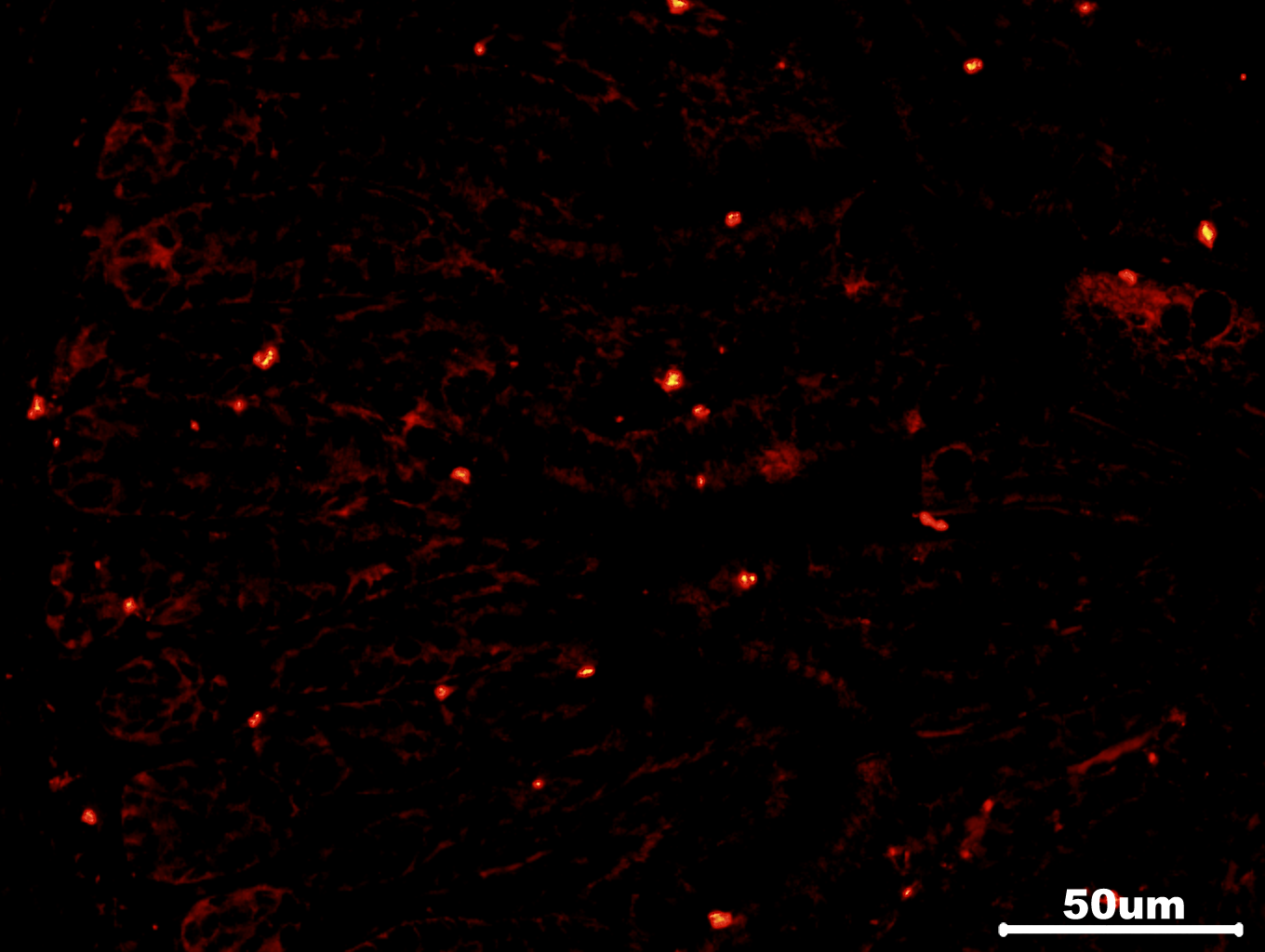

Supplement: Supplementary file 4 [file DataSheet_4.zip › E31-2-200-1-CD206.tif]

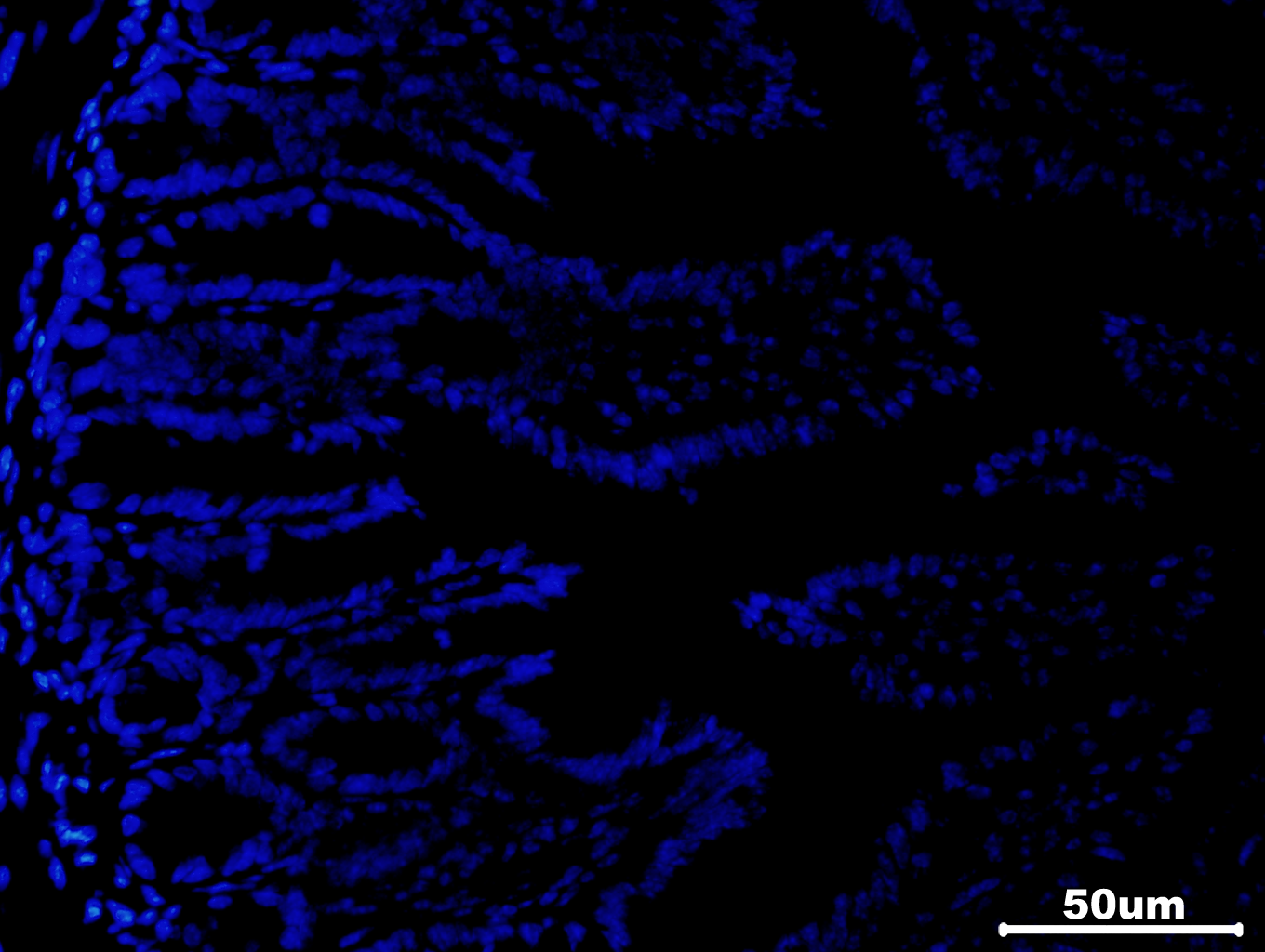

Supplement: Supplementary file 4 [file DataSheet_4.zip › E31-2-200-1-DAPI.tif]

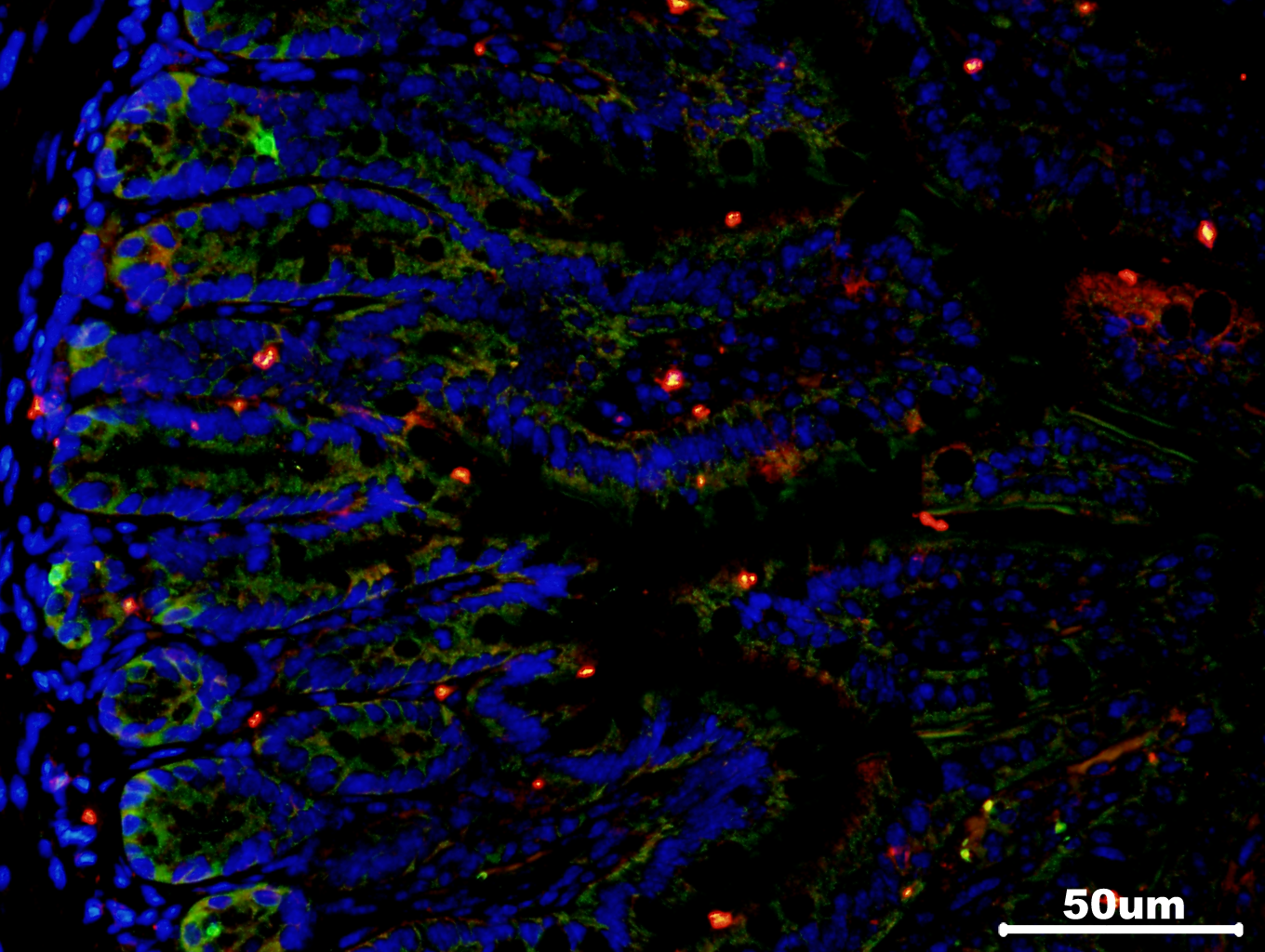

Supplement: Supplementary file 4 [file DataSheet_4.zip › E31-2-200-1-merge.tif]

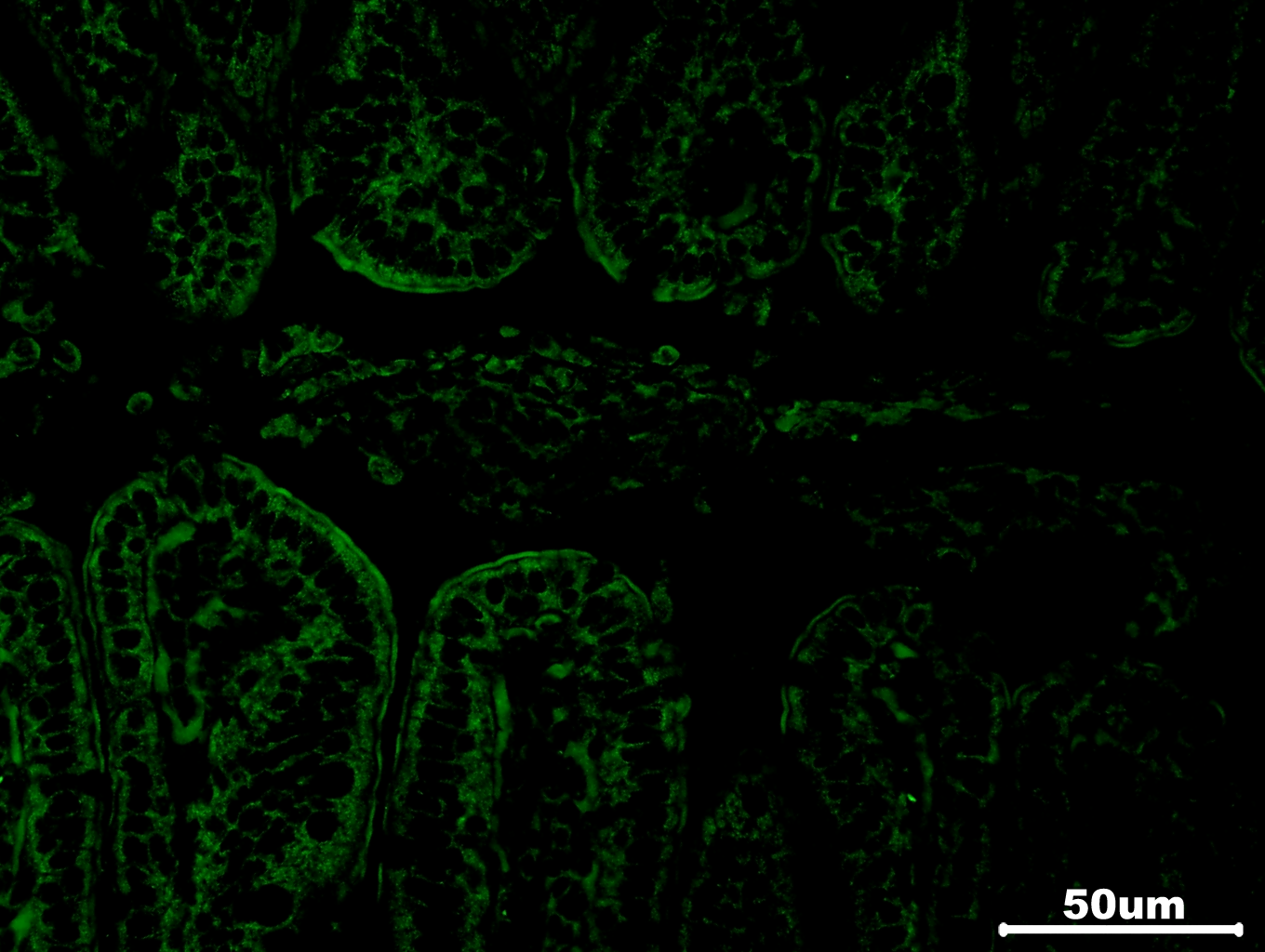

Supplement: Supplementary file 4 [file DataSheet_4.zip › E31-2-200-2-CD86.tif]

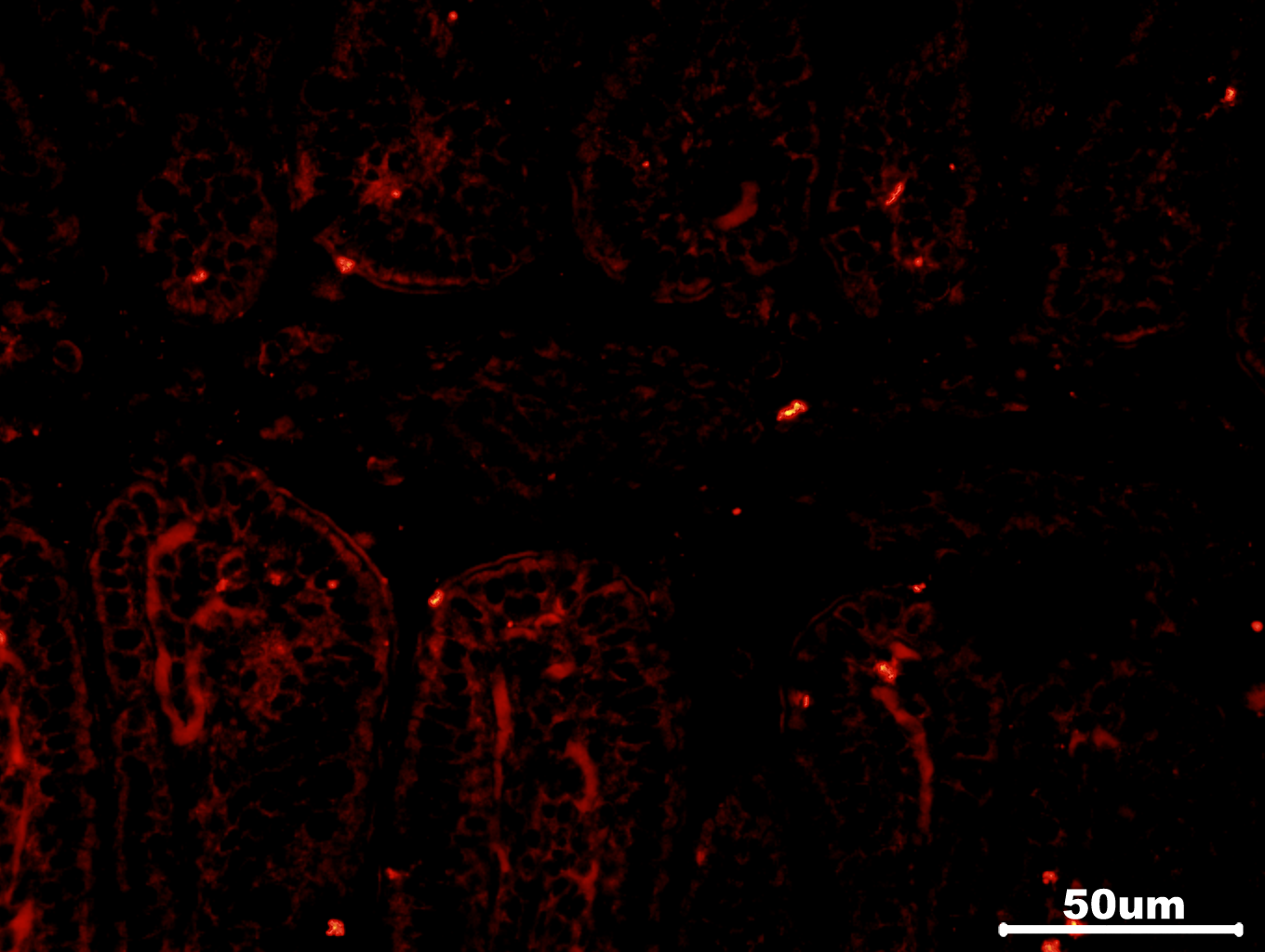

Supplement: Supplementary file 4 [file DataSheet_4.zip › E31-2-200-2-CD206.tif]

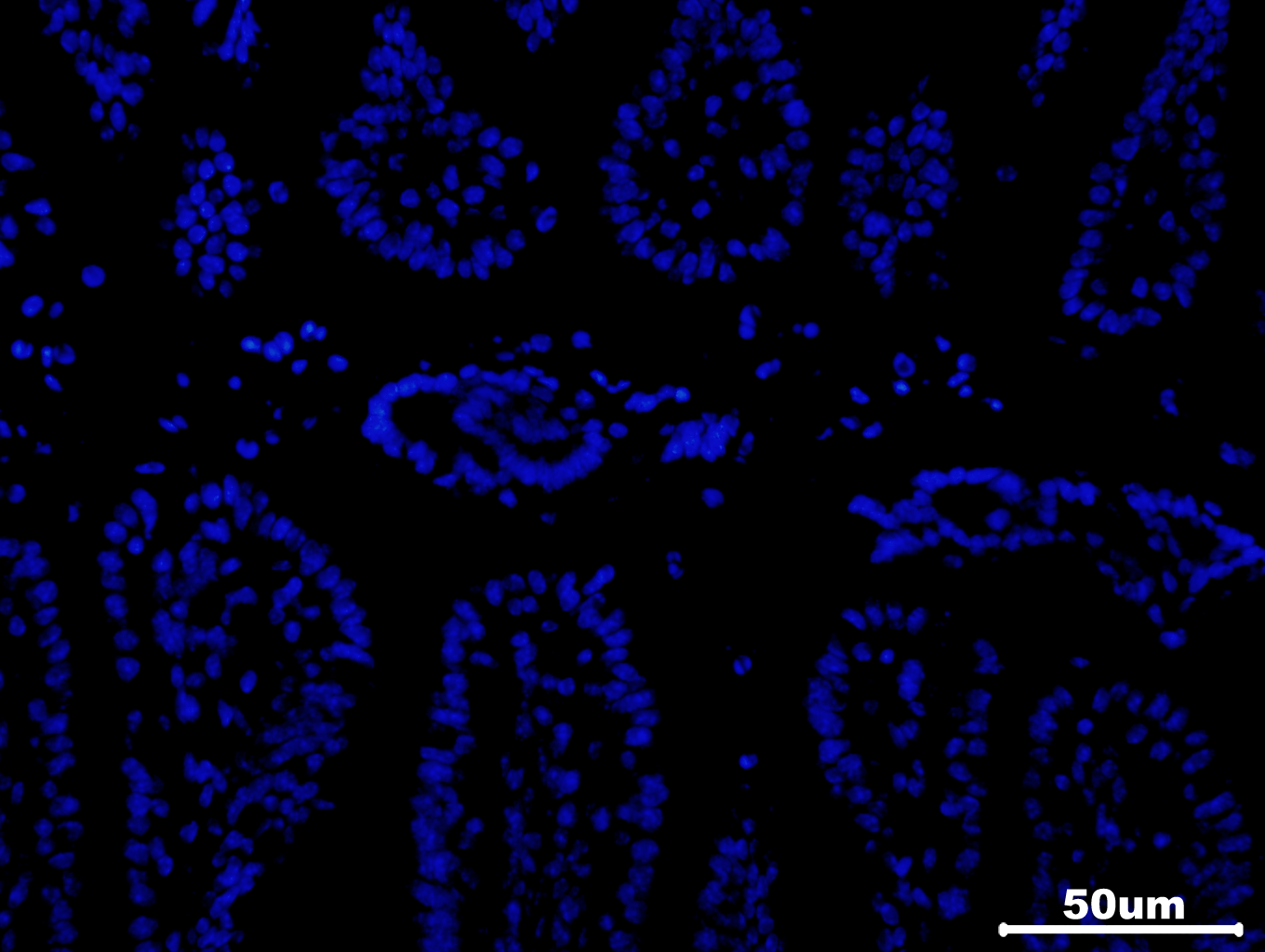

Supplement: Supplementary file 4 [file DataSheet_4.zip › E31-2-200-2-DAPI.tif]

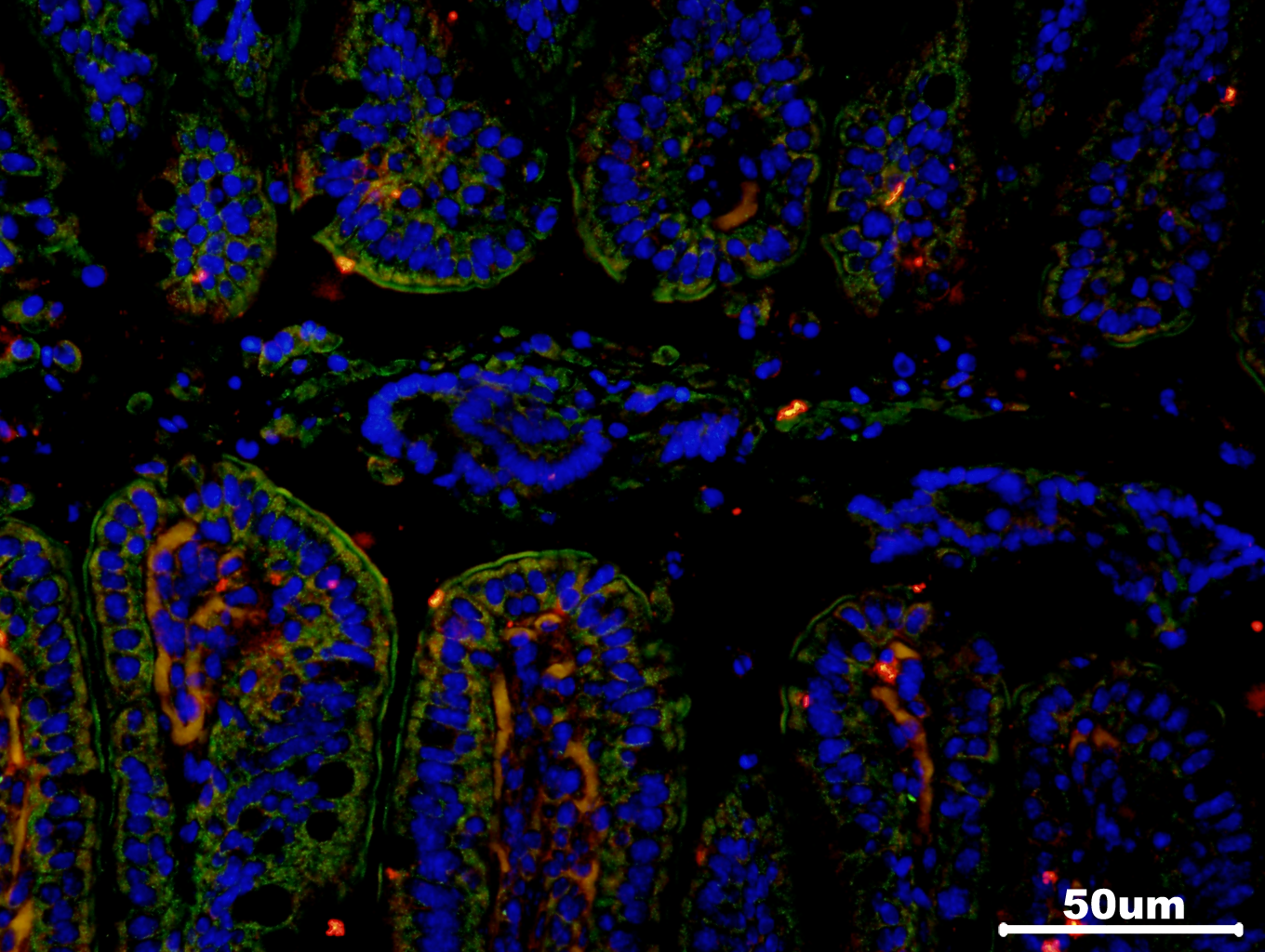

Supplement: Supplementary file 4 [file DataSheet_4.zip › E31-2-200-2-merge.tif]

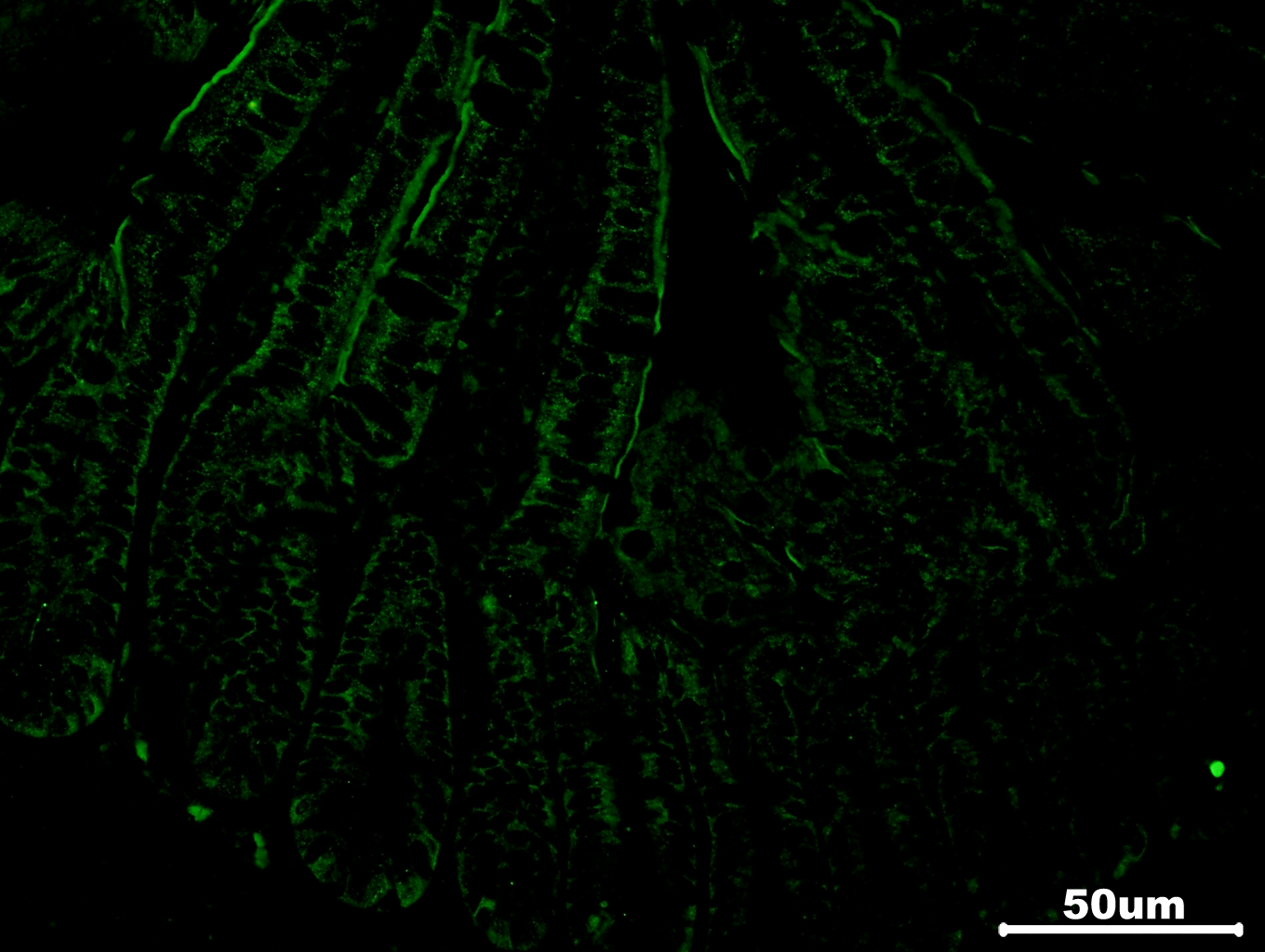

Supplement: Supplementary file 5 [file DataSheet_5.zip › B22-1-200-1-CD86.tif]

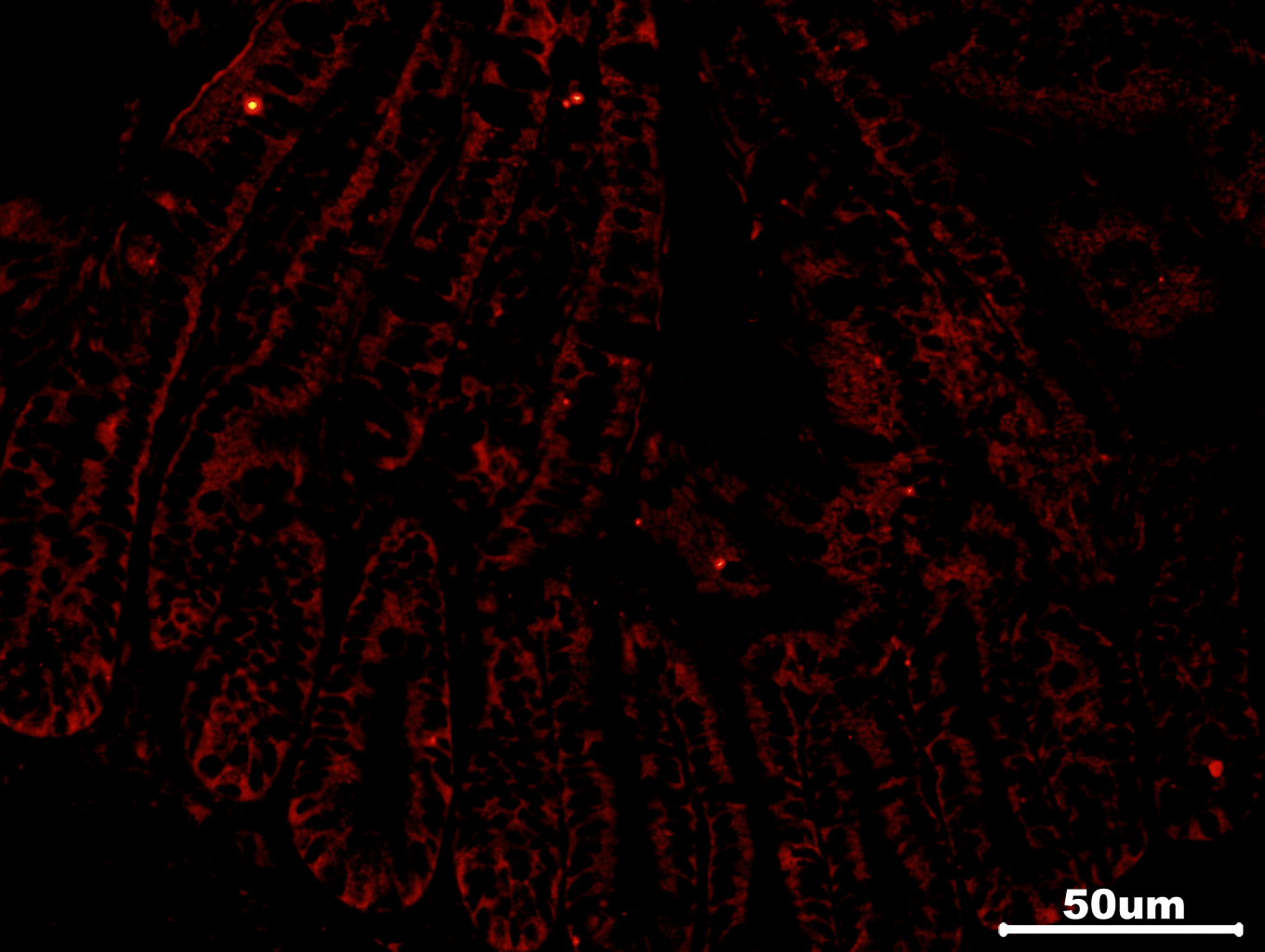

Supplement: Supplementary file 5 [file DataSheet_5.zip › B22-1-200-1-CD206.tif]

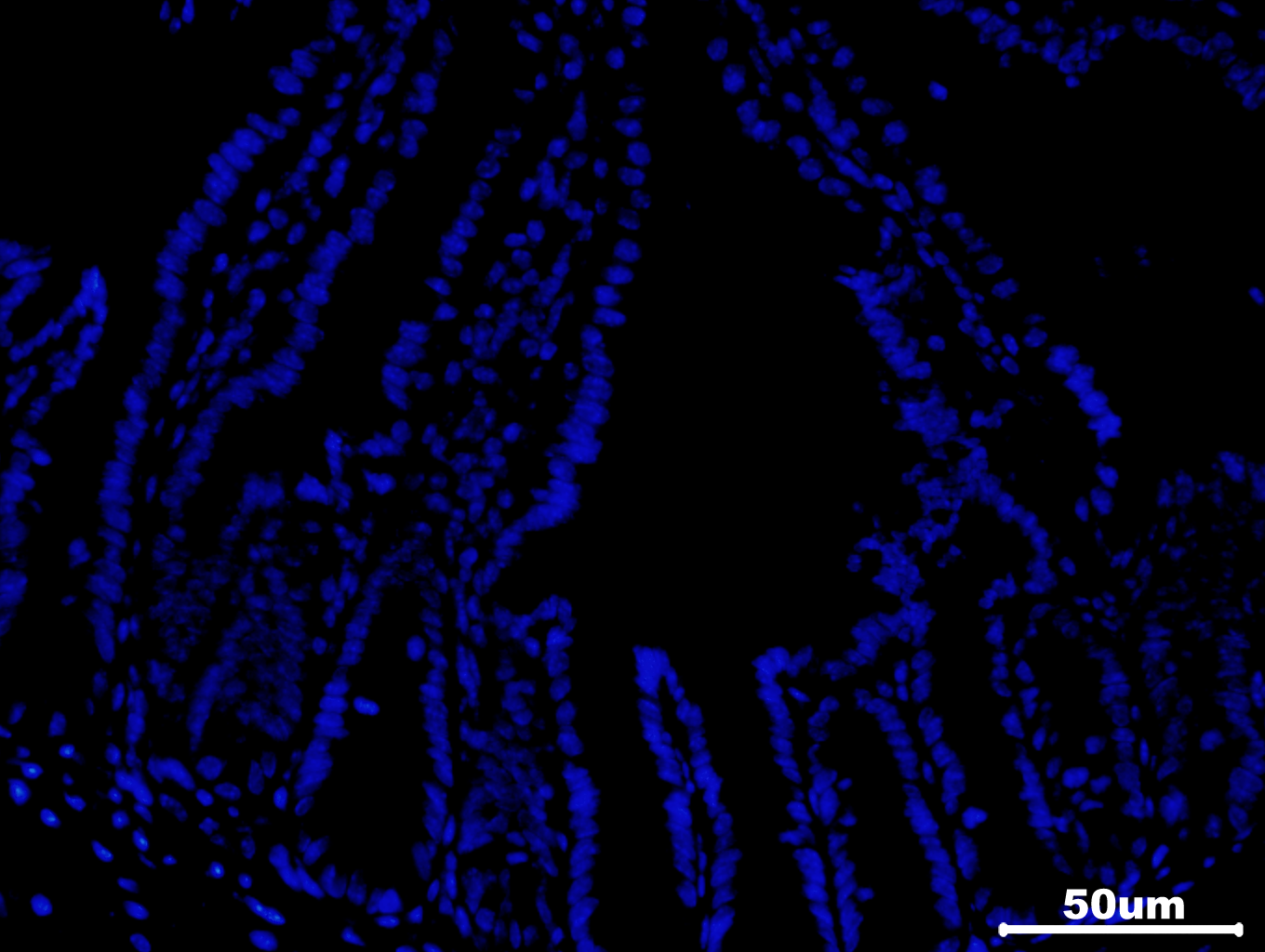

Supplement: Supplementary file 5 [file DataSheet_5.zip › B22-1-200-1-DAPI.tif]

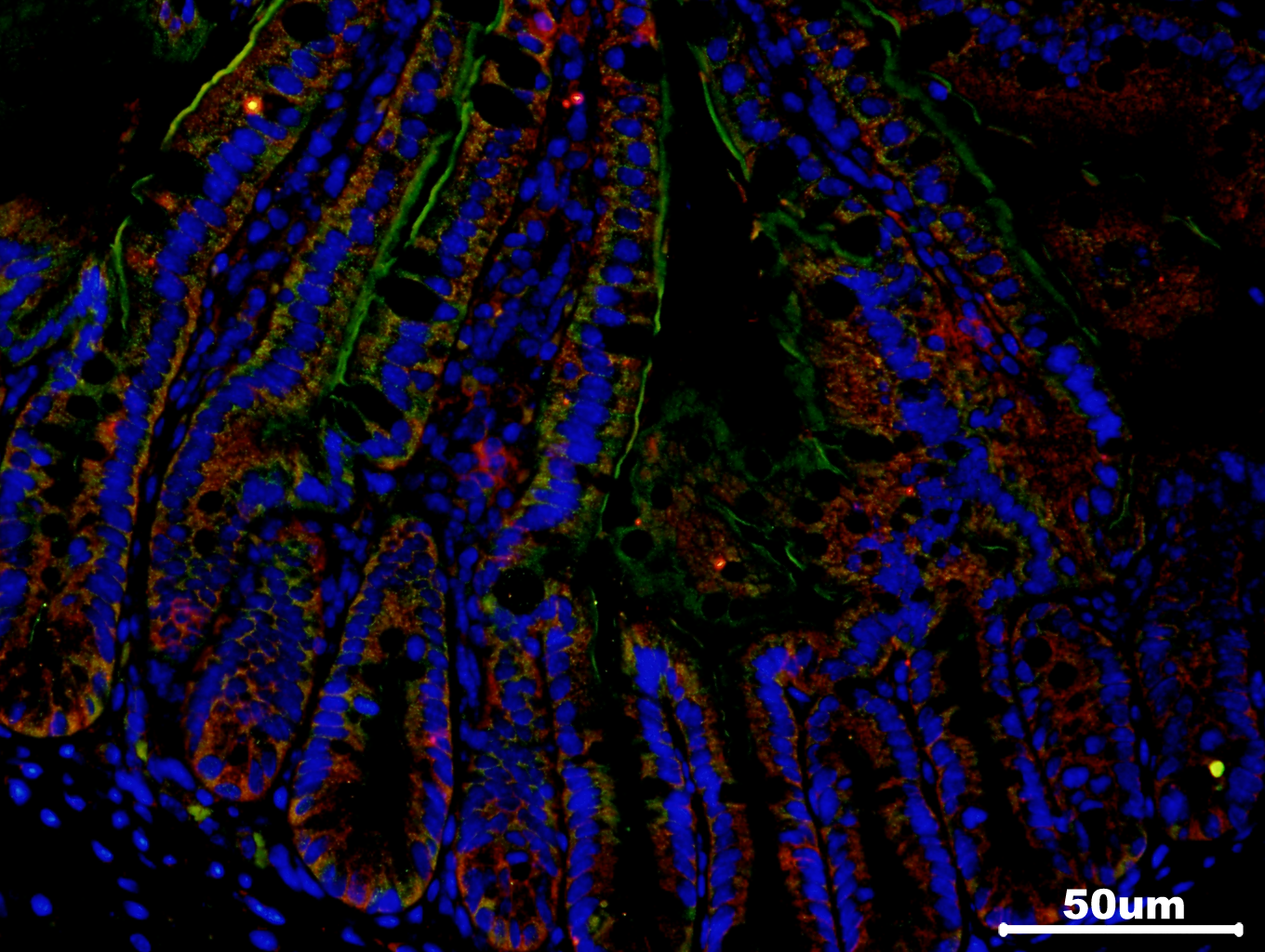

Supplement: Supplementary file 5 [file DataSheet_5.zip › B22-1-200-1-merge.tif]

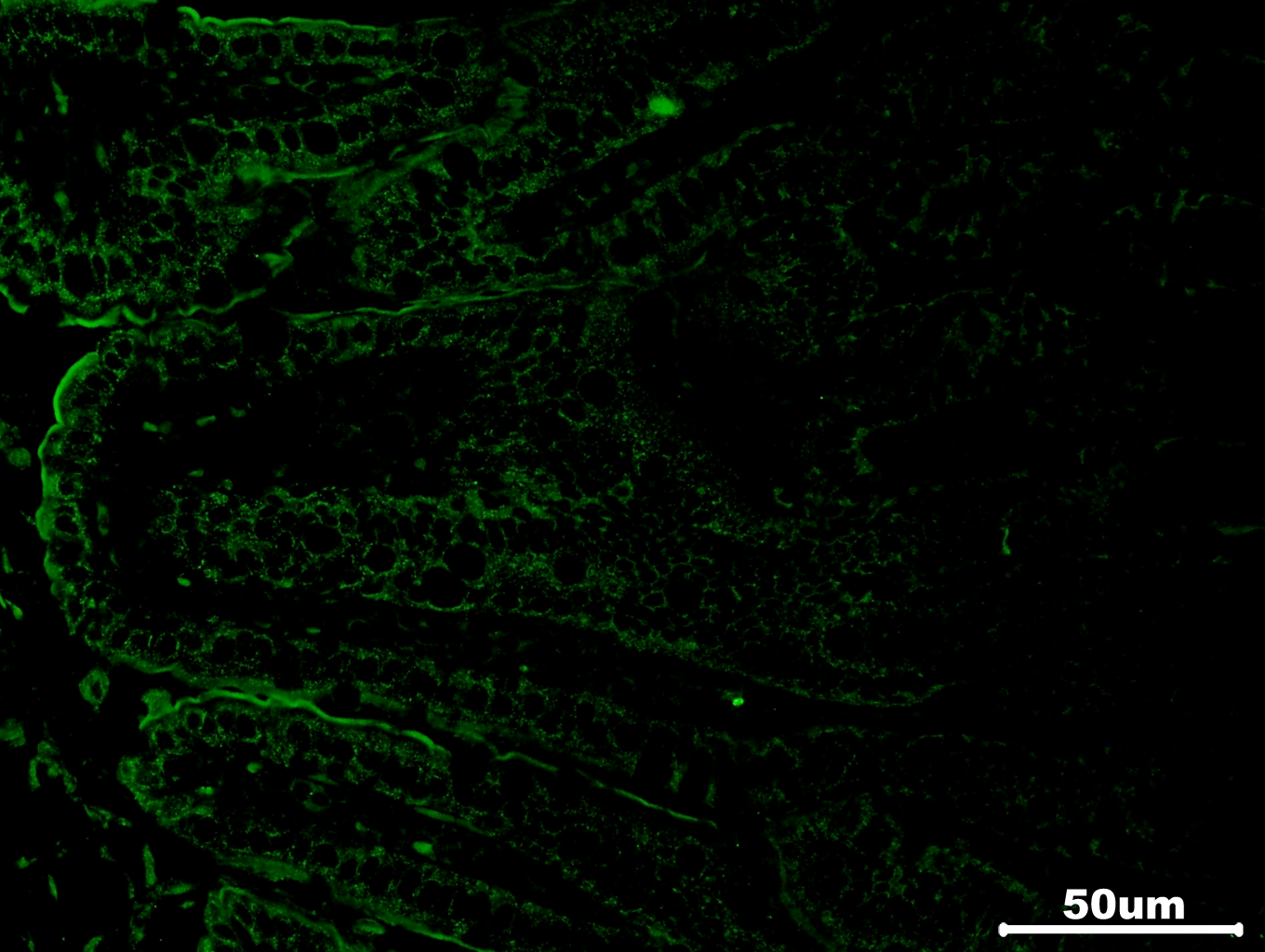

Supplement: Supplementary file 5 [file DataSheet_5.zip › B22-1-200-2-CD86.tif]

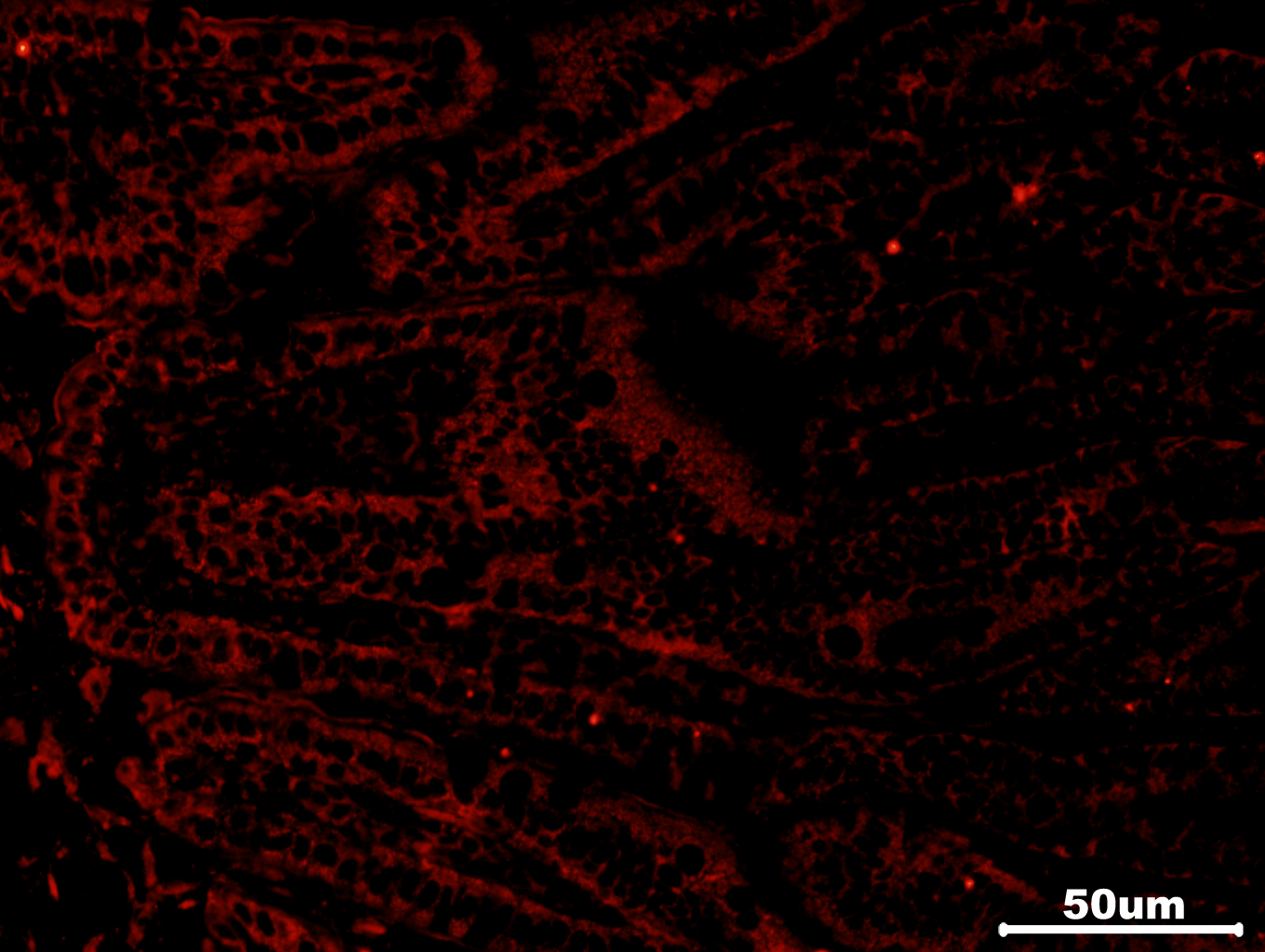

Supplement: Supplementary file 5 [file DataSheet_5.zip › B22-1-200-2-CD206.tif]

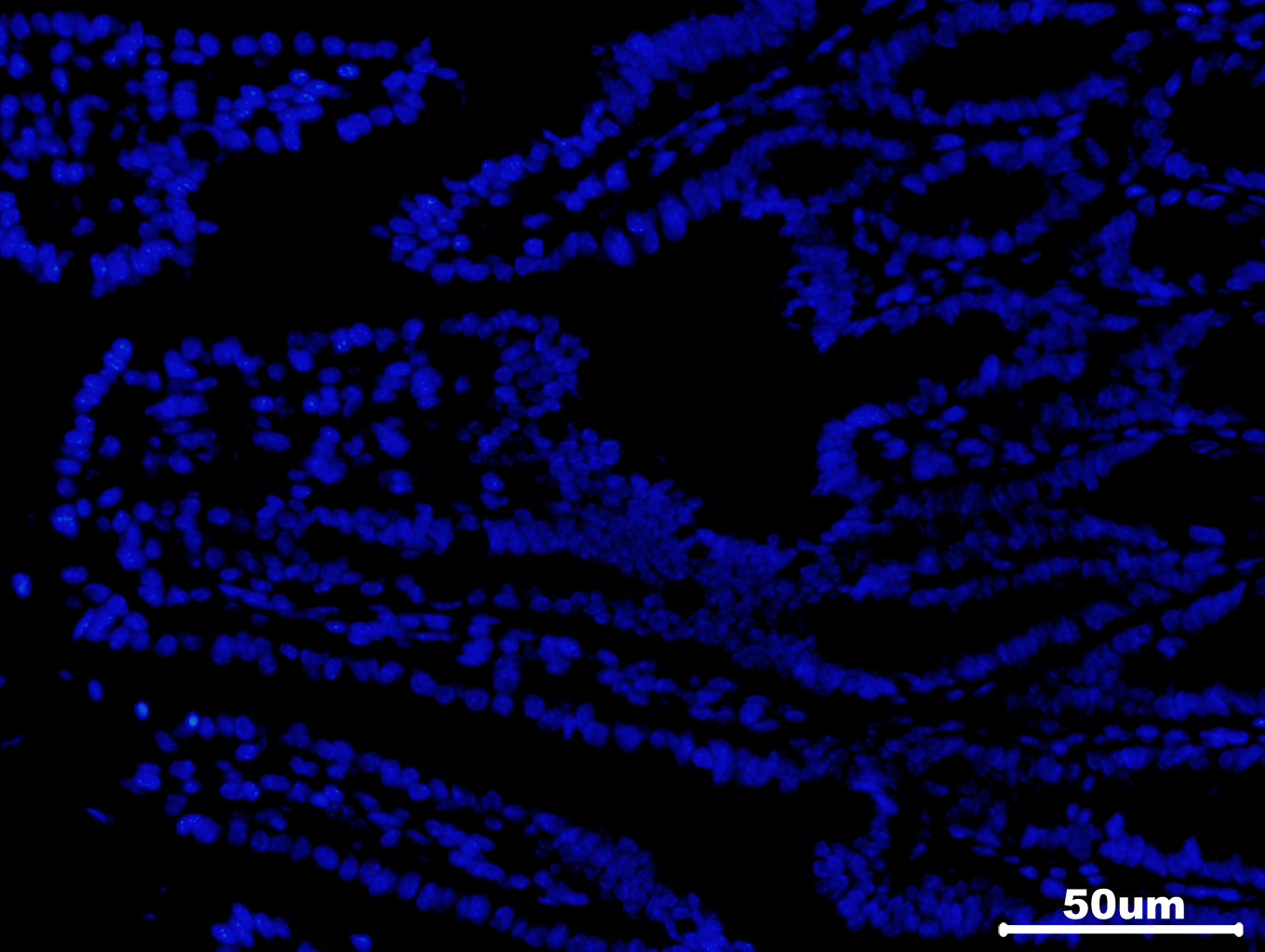

Supplement: Supplementary file 5 [file DataSheet_5.zip › B22-1-200-2-DAPI.tif]

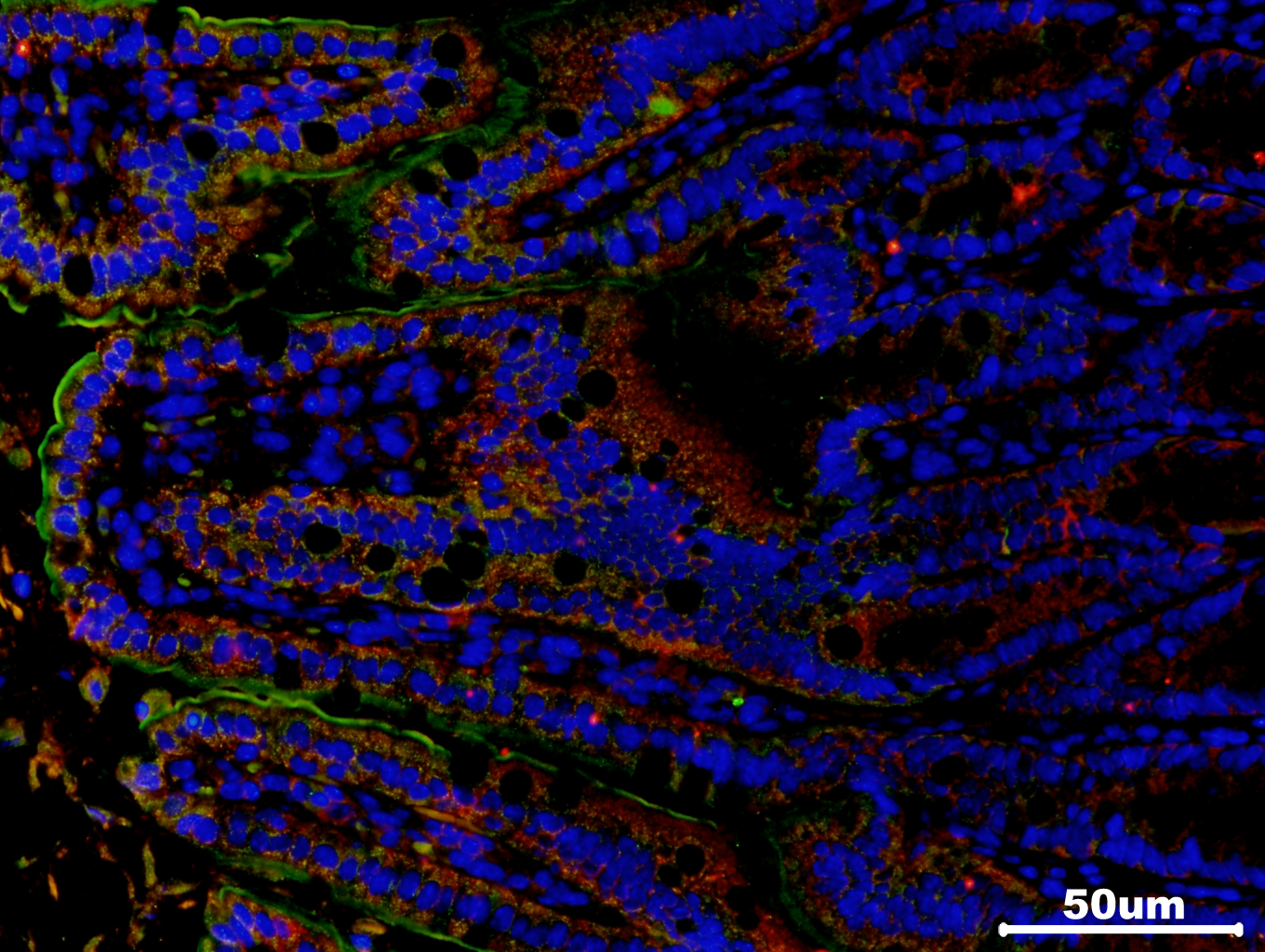

Supplement: Supplementary file 5 [file DataSheet_5.zip › B22-1-200-2-merge.tif]

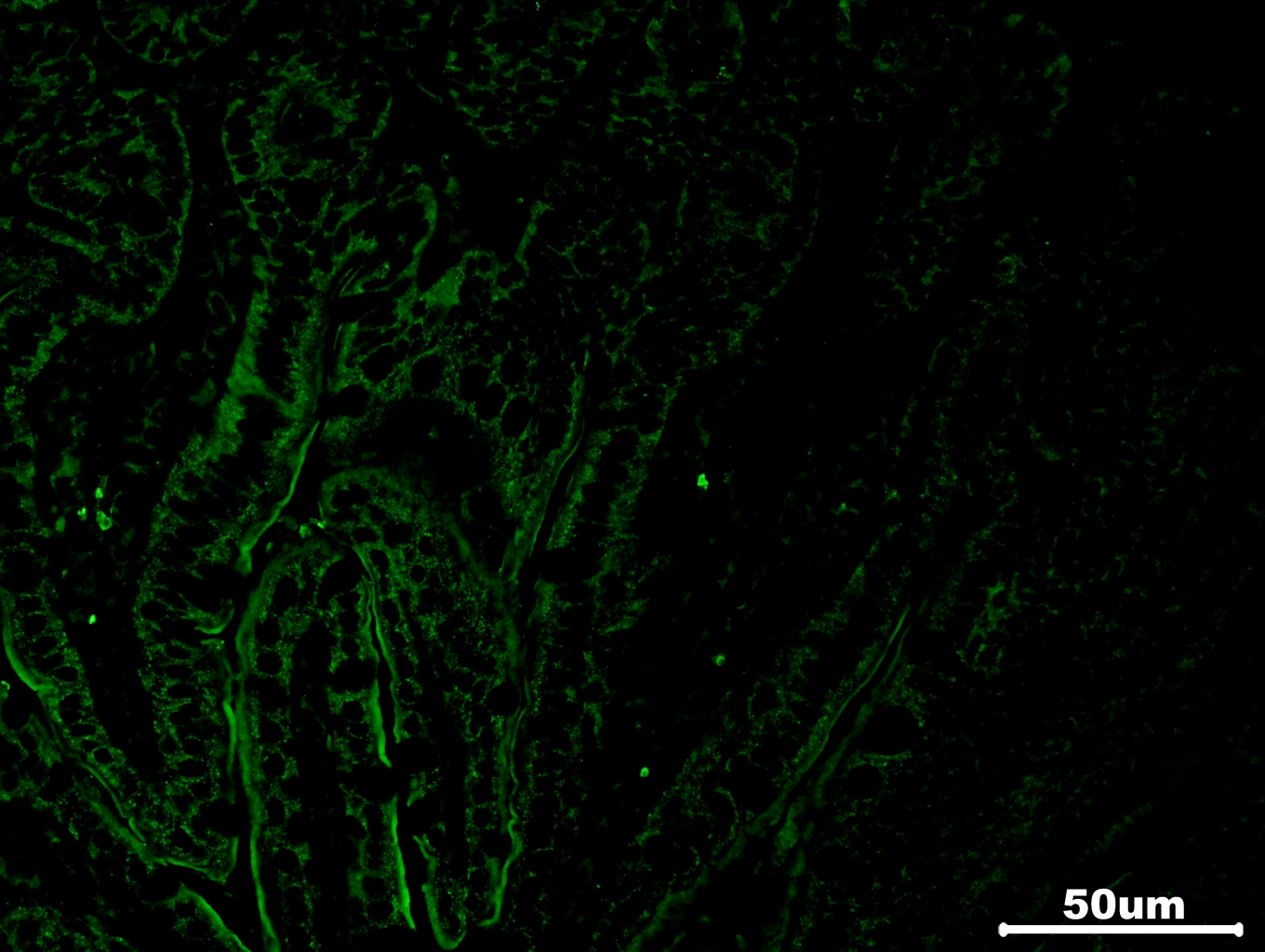

Supplement: Supplementary file 5 [file DataSheet_5.zip › B22-1-200-3-CD86.tif]

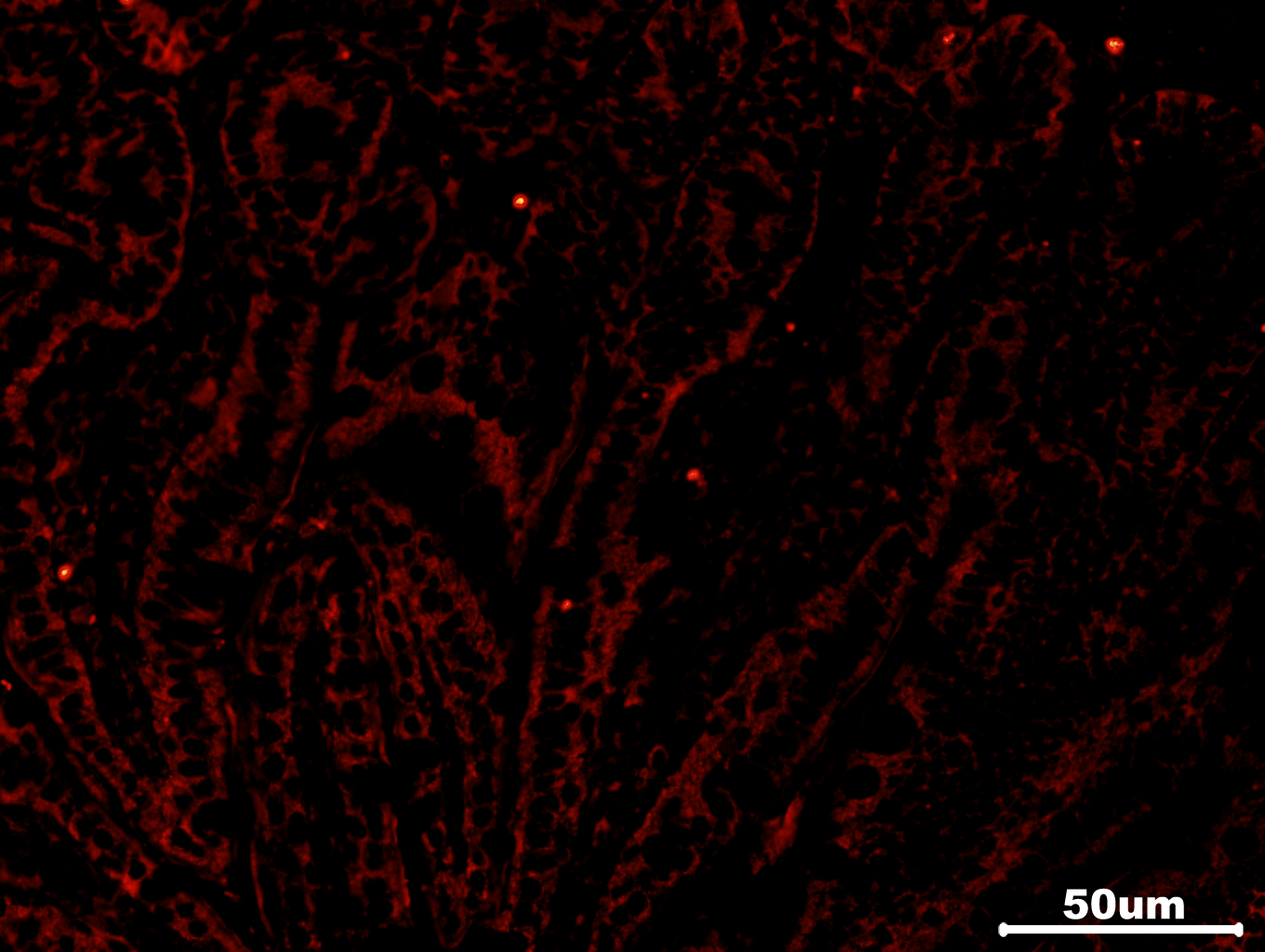

Supplement: Supplementary file 5 [file DataSheet_5.zip › B22-1-200-3-CD206.tif]

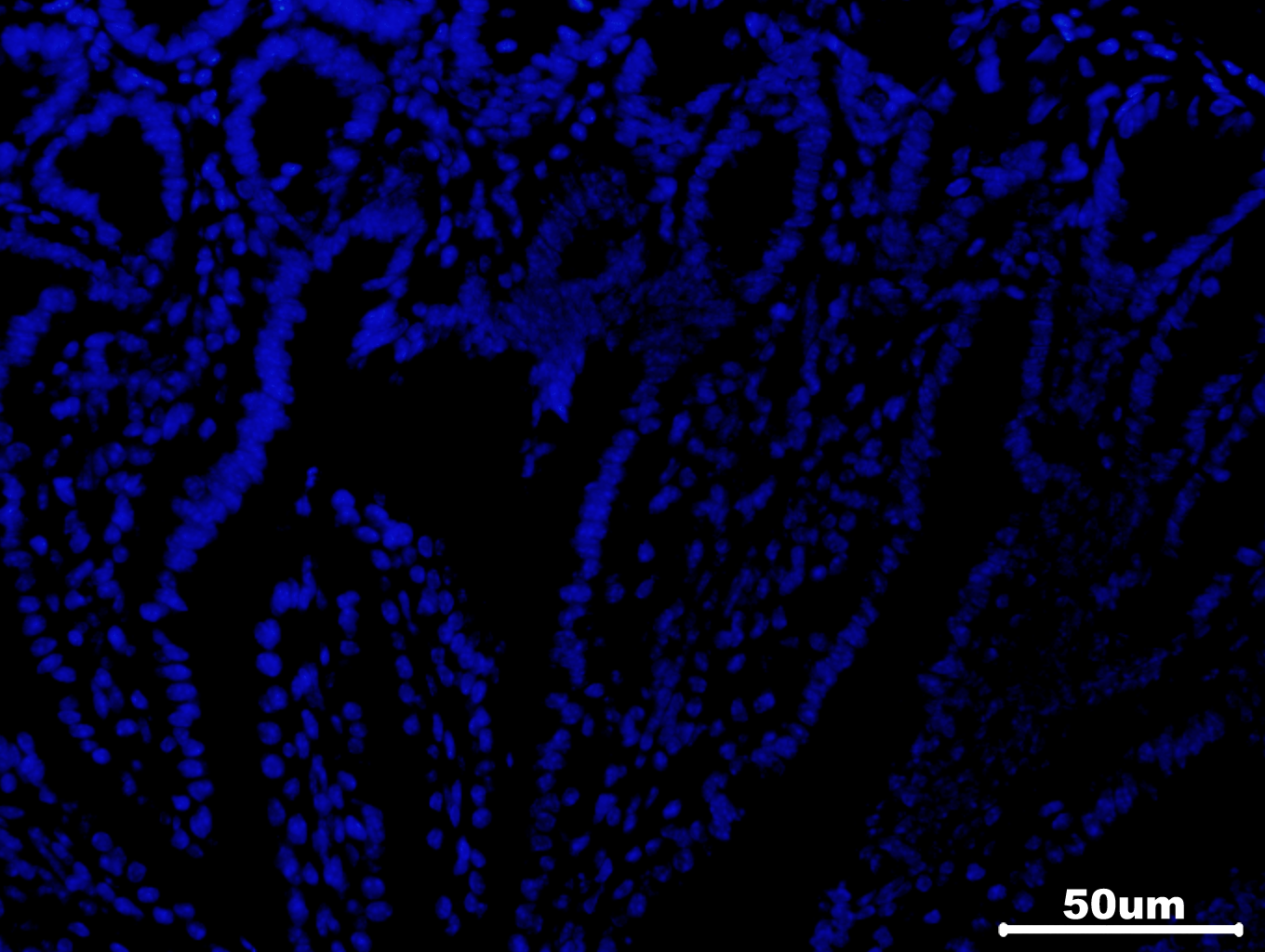

Supplement: Supplementary file 5 [file DataSheet_5.zip › B22-1-200-3-DAPI.tif]

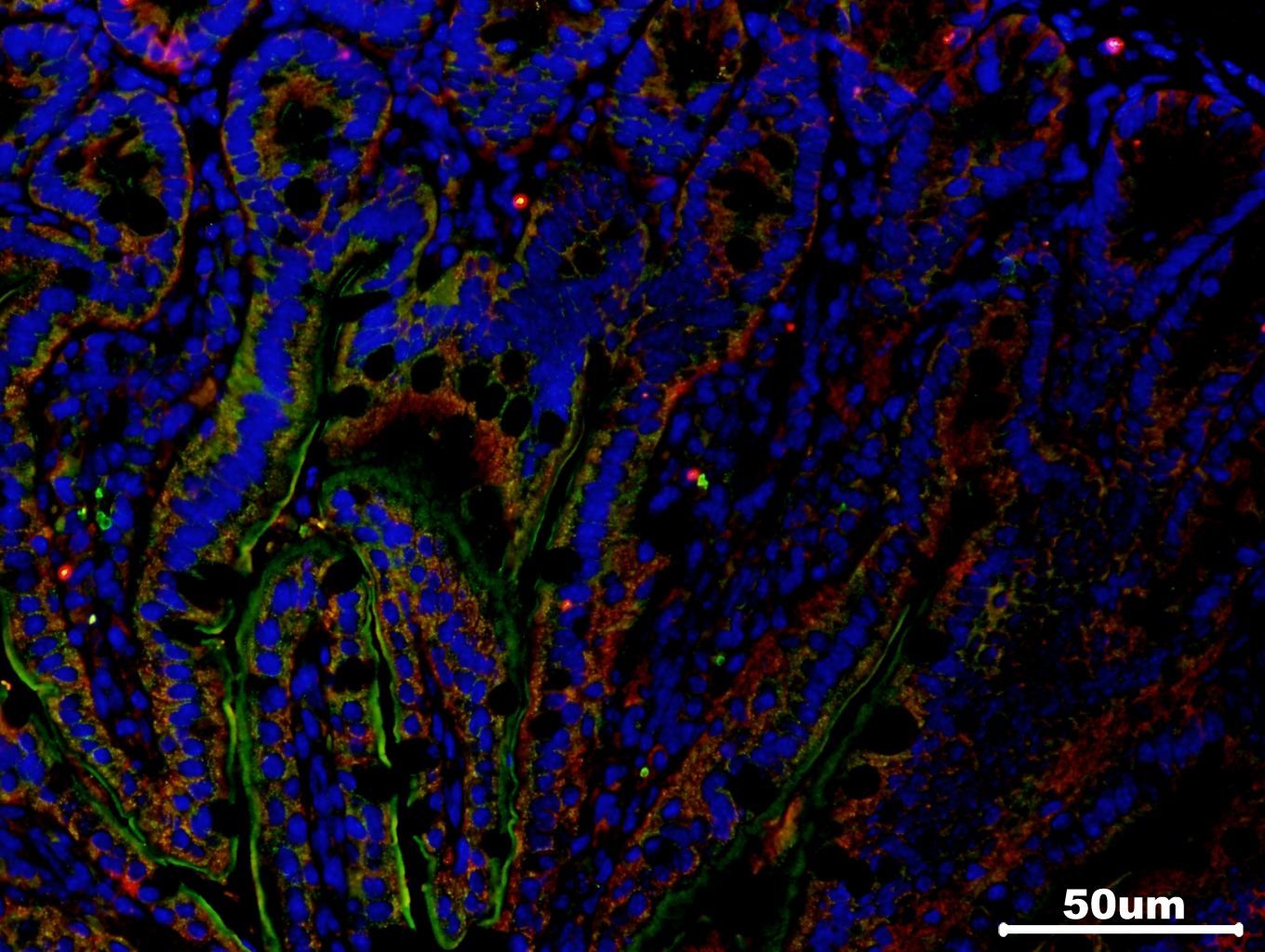

Supplement: Supplementary file 5 [file DataSheet_5.zip › B22-1-200-3-merge.tif]

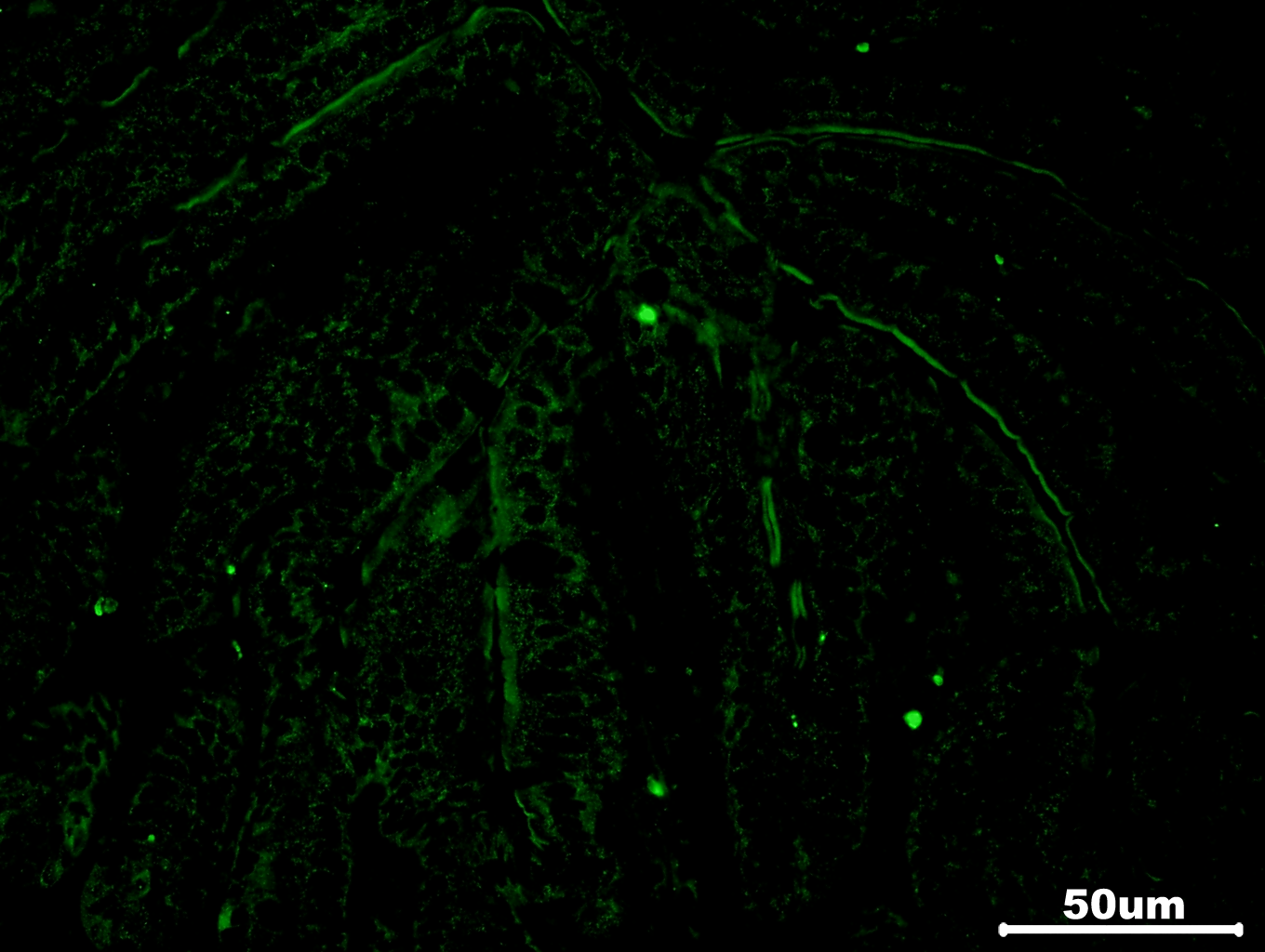

Supplement: Supplementary file 5 [file DataSheet_5.zip › B22-2-200-1-CD86.tif]

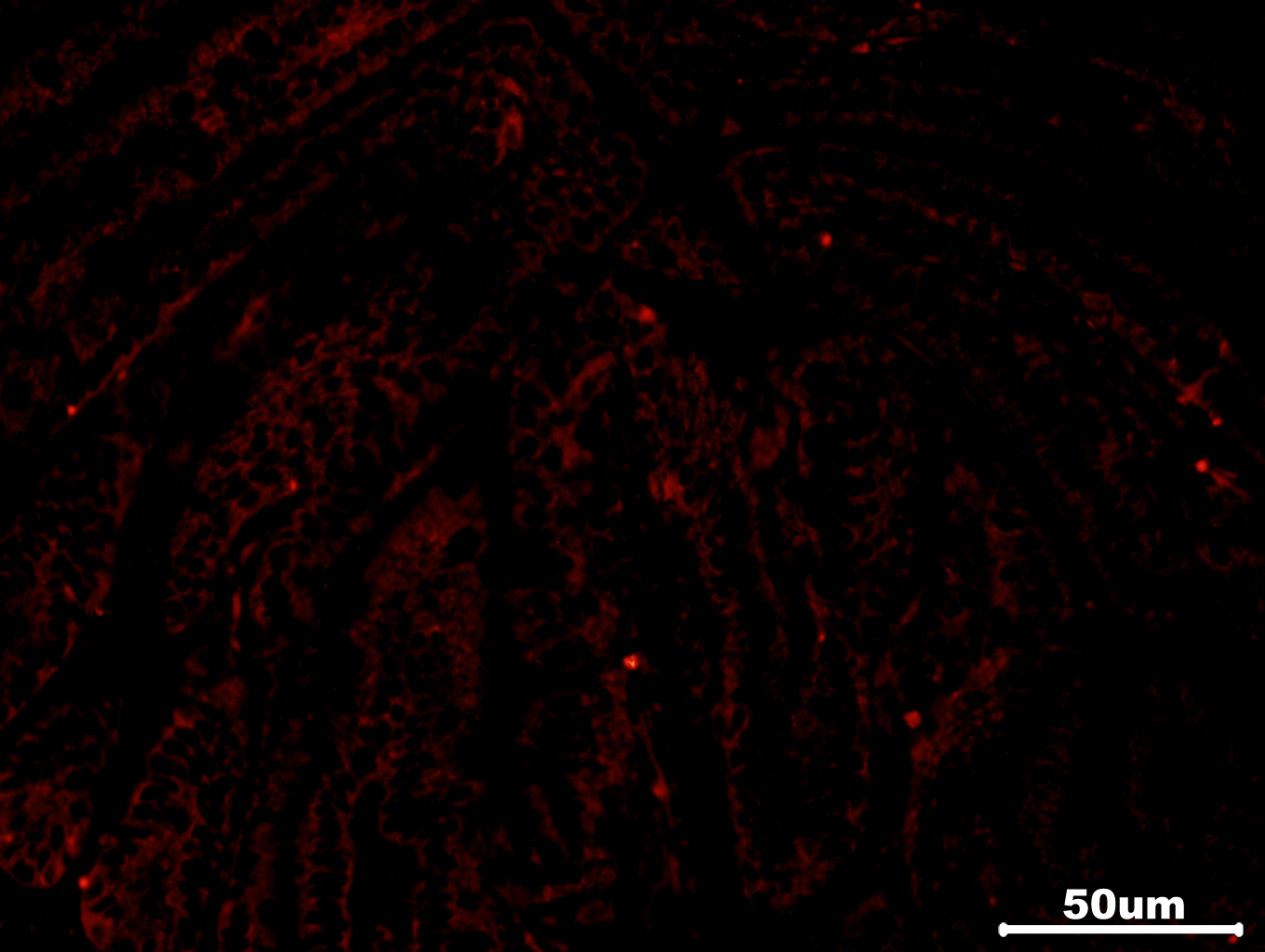

Supplement: Supplementary file 5 [file DataSheet_5.zip › B22-2-200-1-CD206.tif]

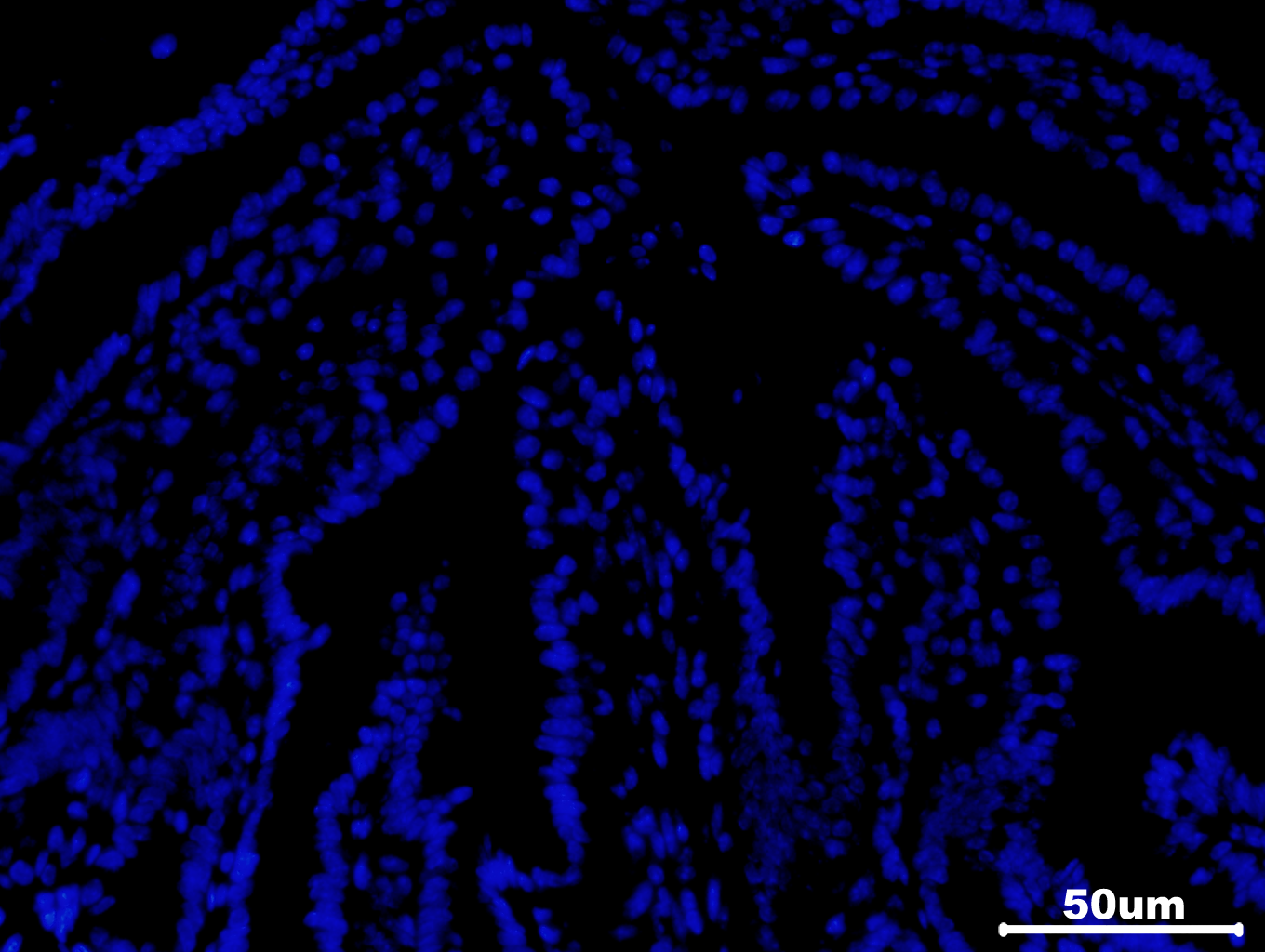

Supplement: Supplementary file 5 [file DataSheet_5.zip › B22-2-200-1-DAPI.tif]

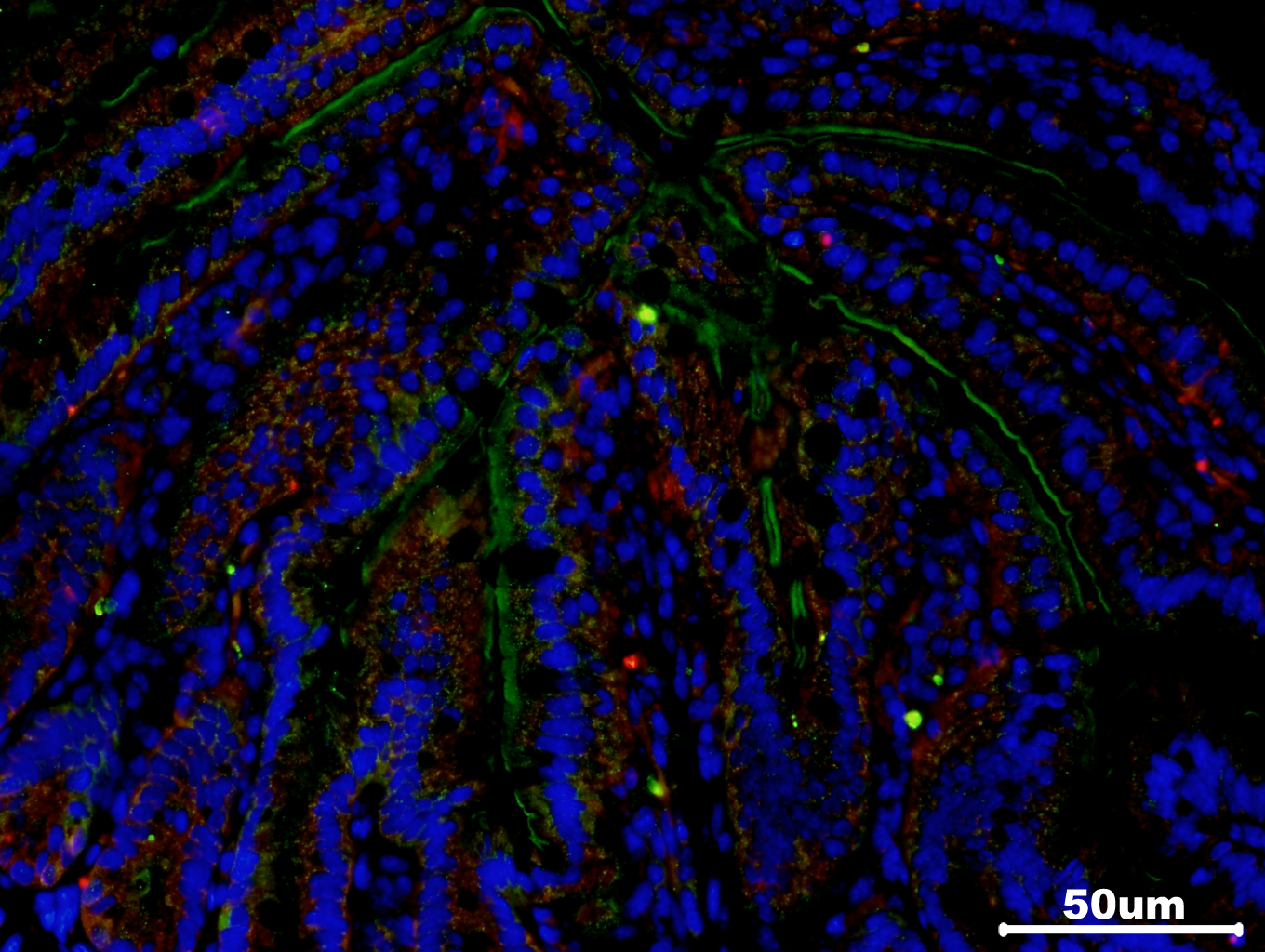

Supplement: Supplementary file 5 [file DataSheet_5.zip › B22-2-200-1-merge.tif]

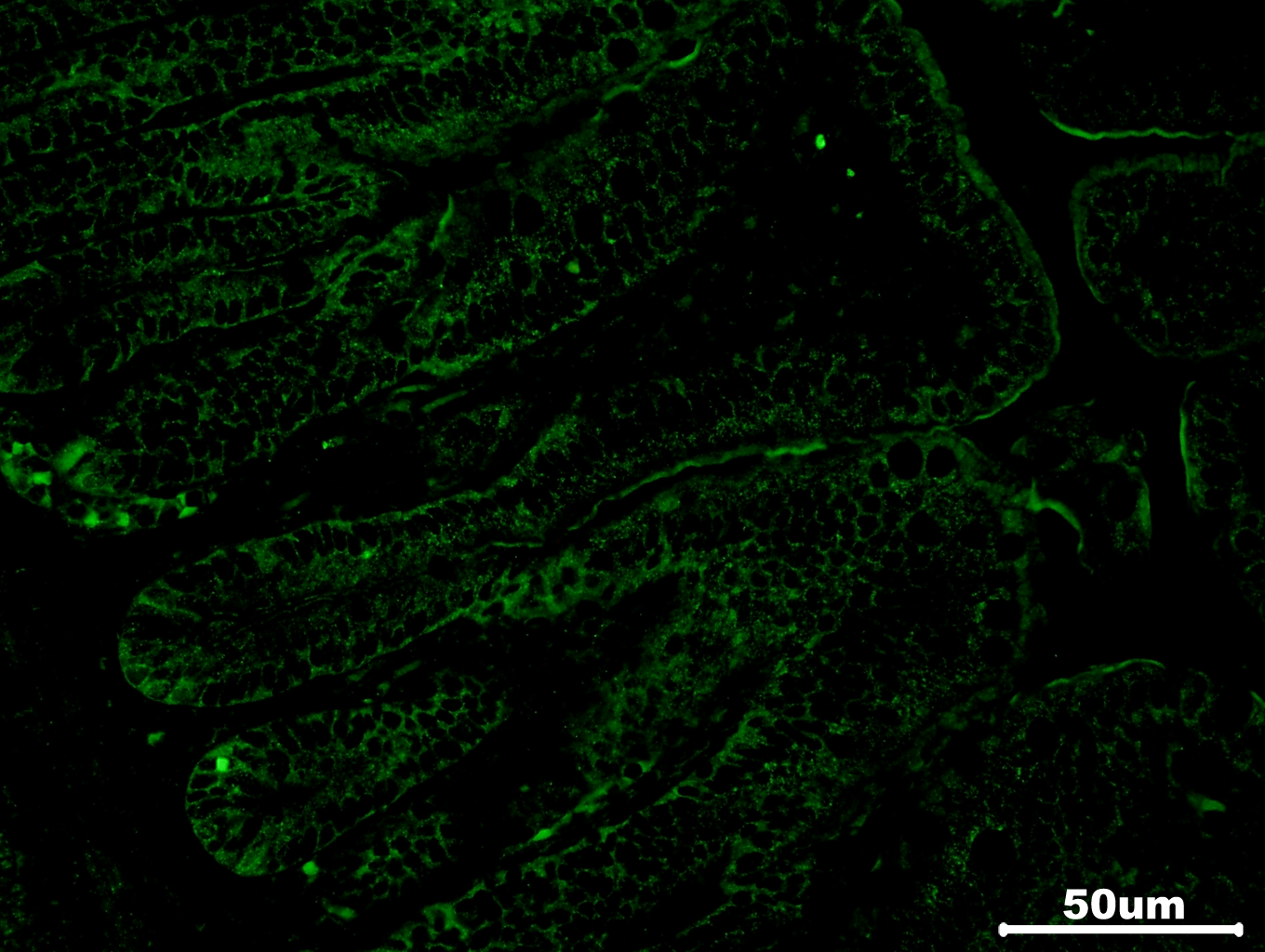

Supplement: Supplementary file 5 [file DataSheet_5.zip › B22-2-200-2-CD86.tif]

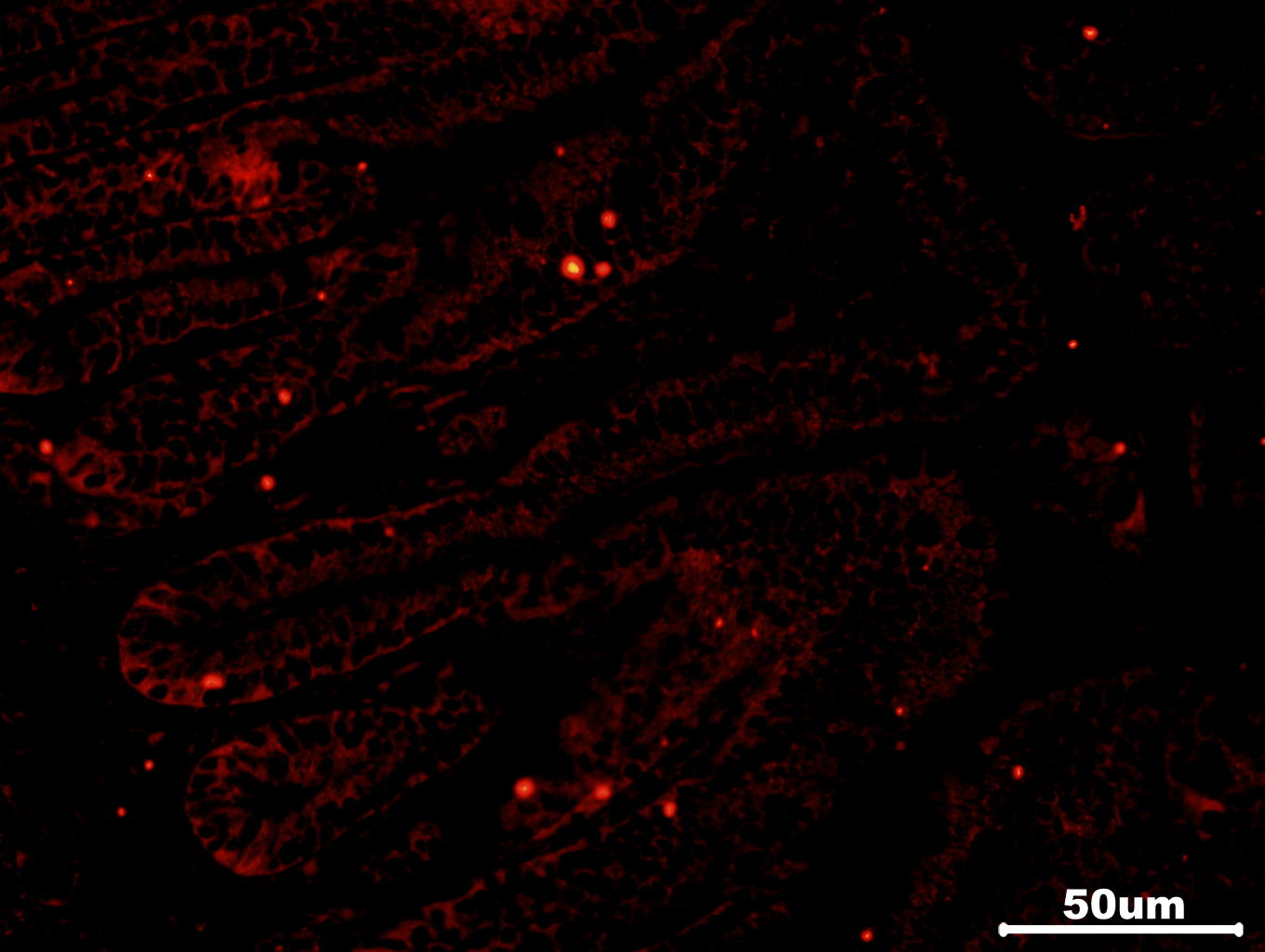

Supplement: Supplementary file 5 [file DataSheet_5.zip › B22-2-200-2-CD206.tif]

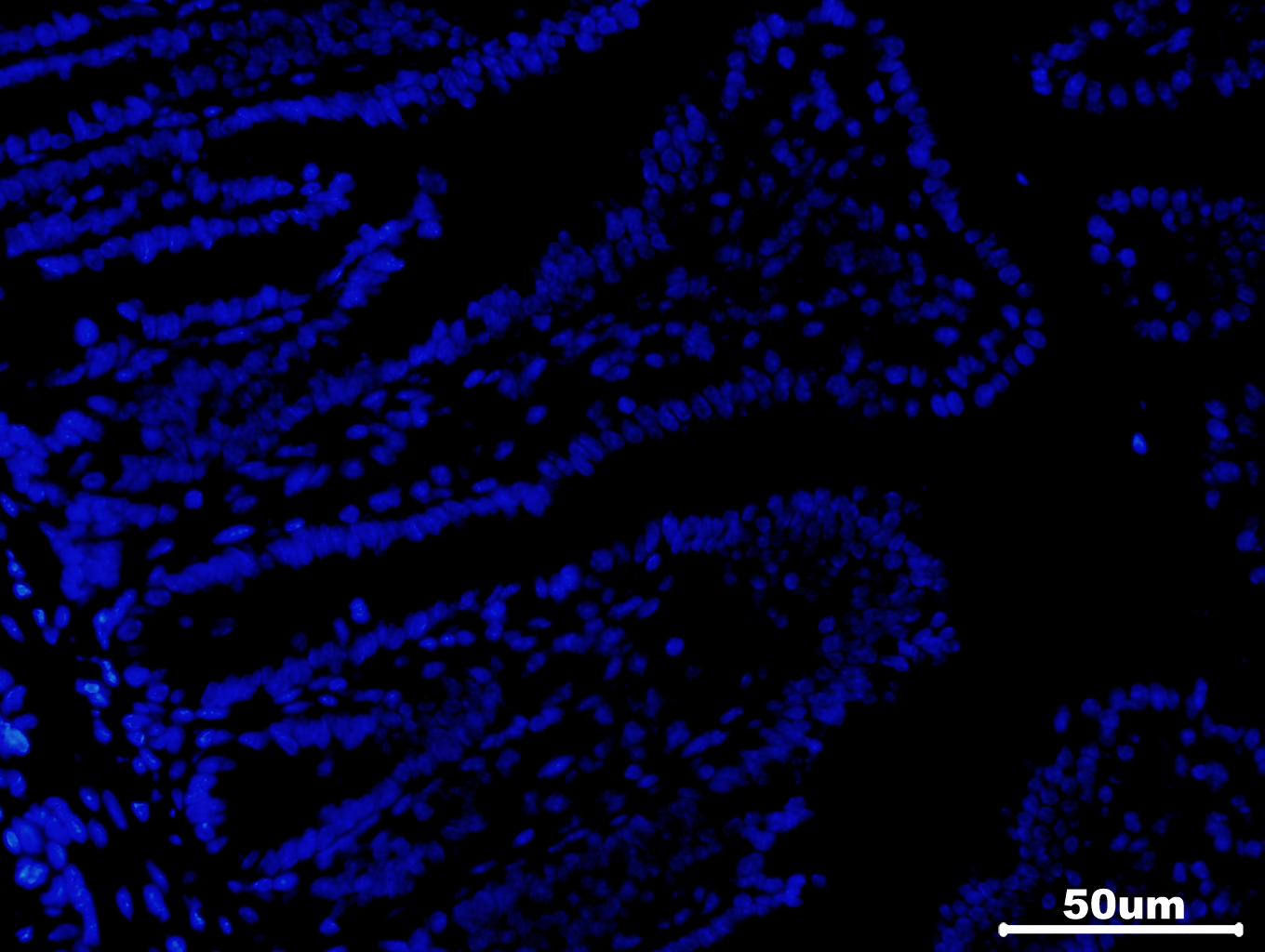

Supplement: Supplementary file 5 [file DataSheet_5.zip › B22-2-200-2-DAPI.tif]

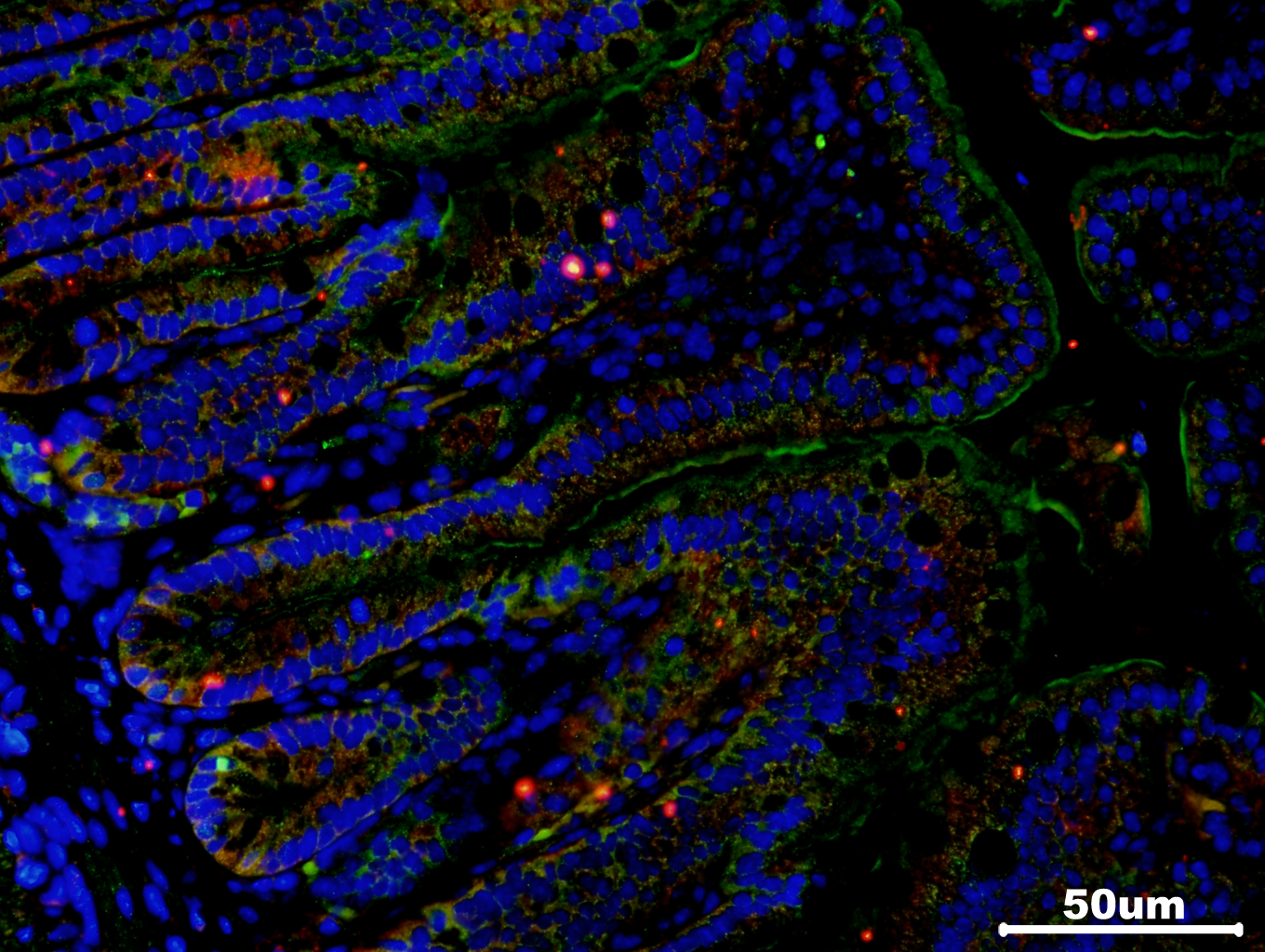

Supplement: Supplementary file 5 [file DataSheet_5.zip › B22-2-200-2-merge.tif]

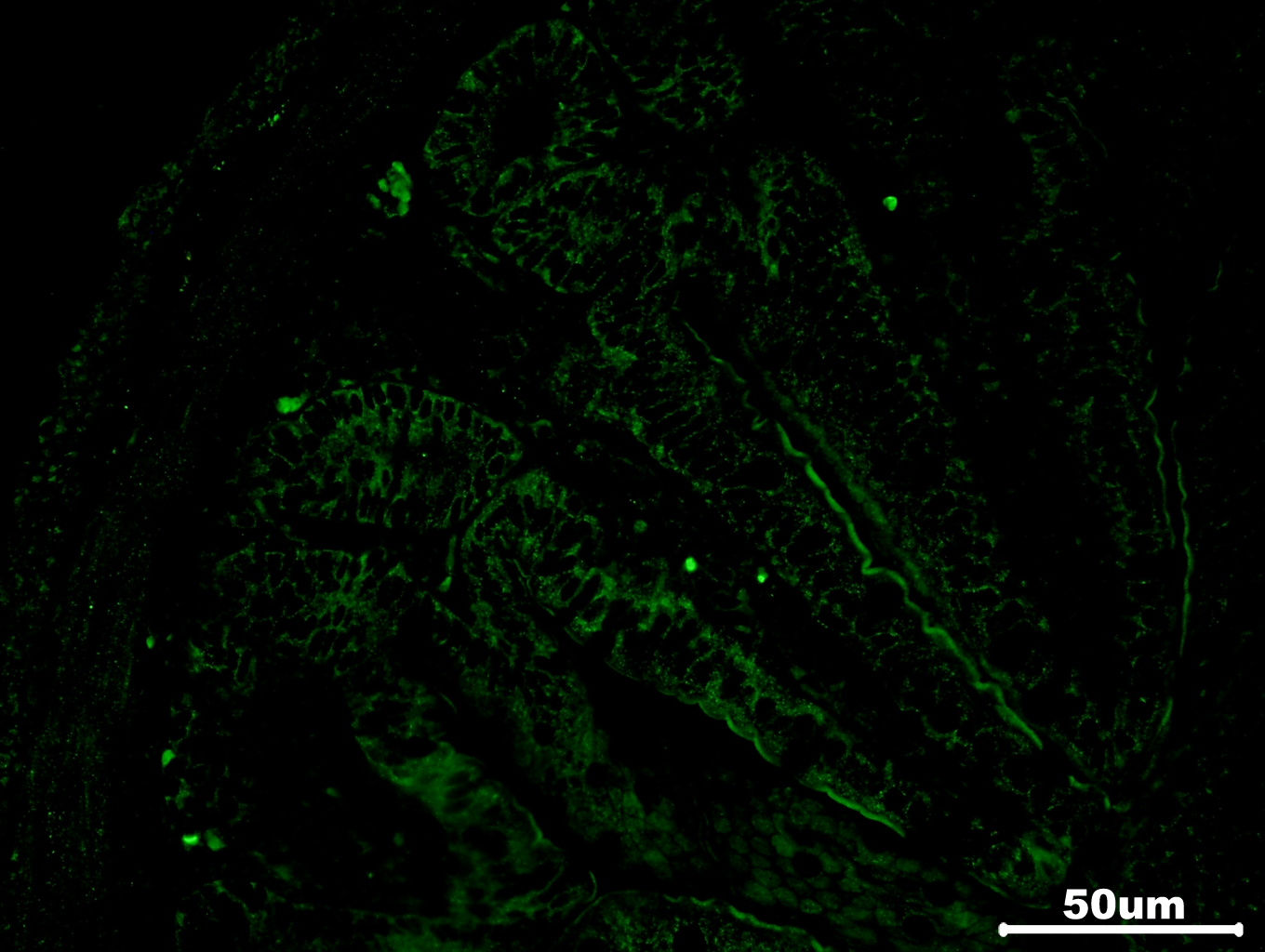

Supplement: Supplementary file 5 [file DataSheet_5.zip › B22-2-200-3-CD86.tif]

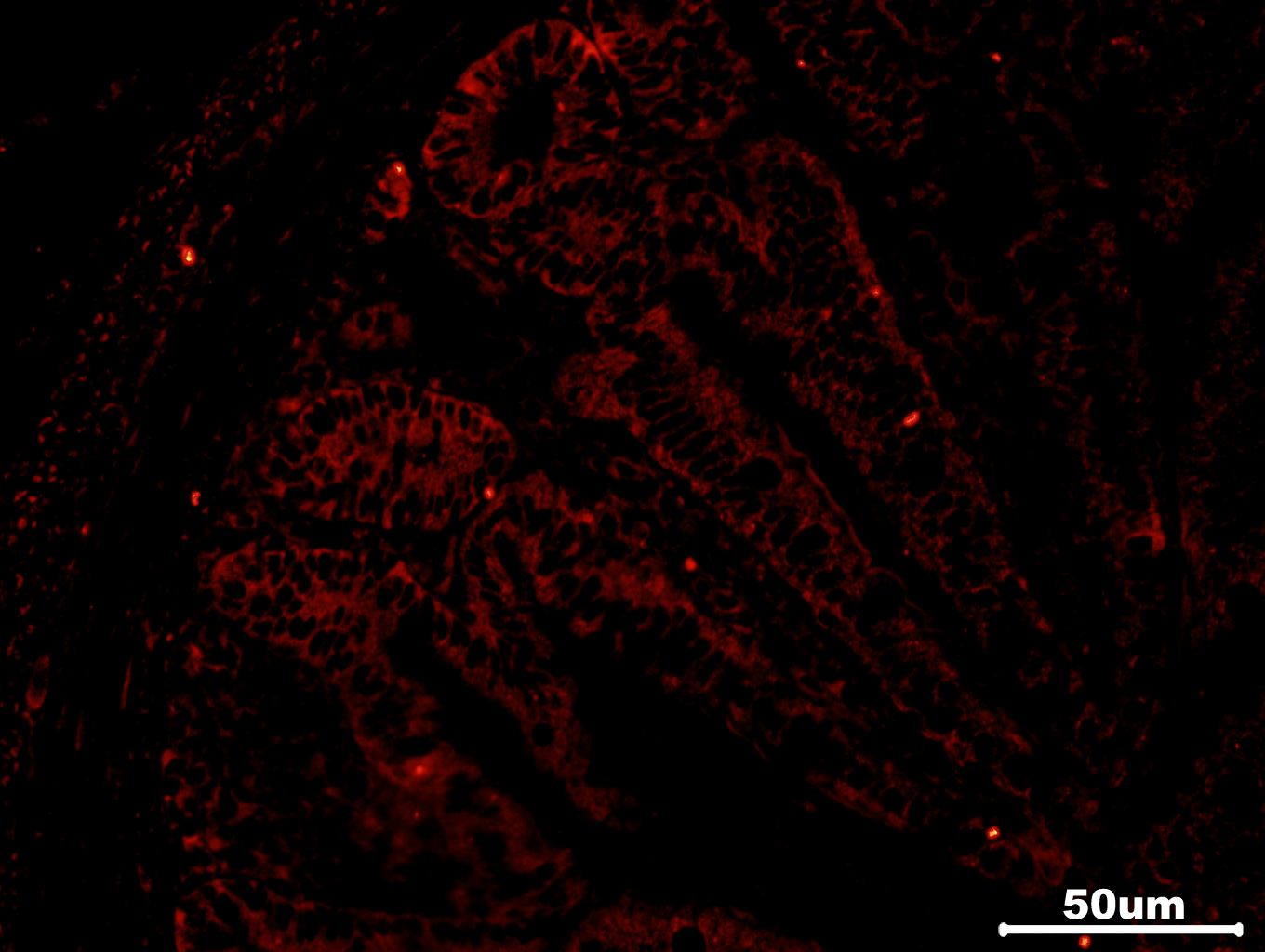

Supplement: Supplementary file 5 [file DataSheet_5.zip › B22-2-200-3-CD206.tif]

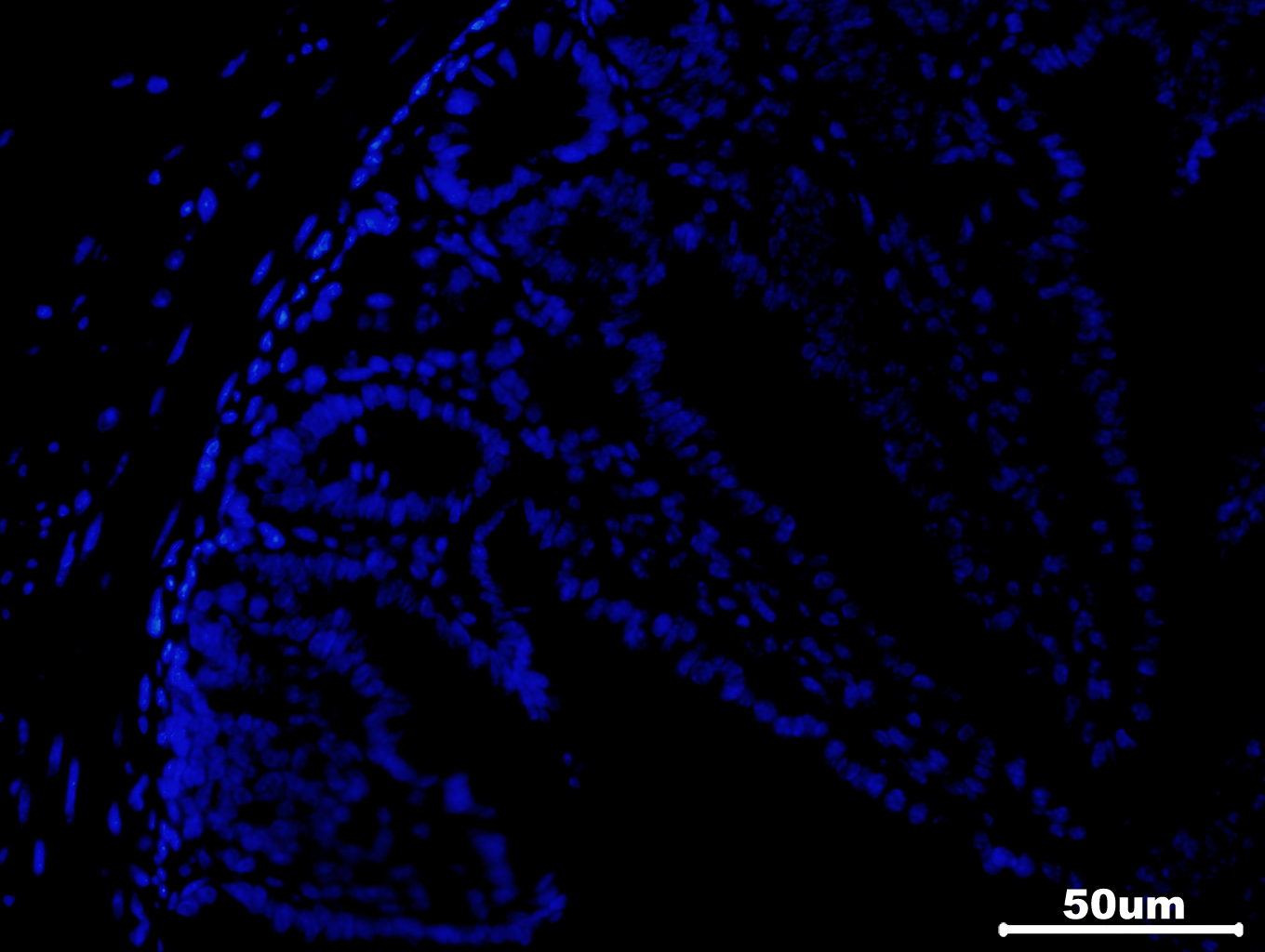

Supplement: Supplementary file 5 [file DataSheet_5.zip › B22-2-200-3-DAPI.tif]

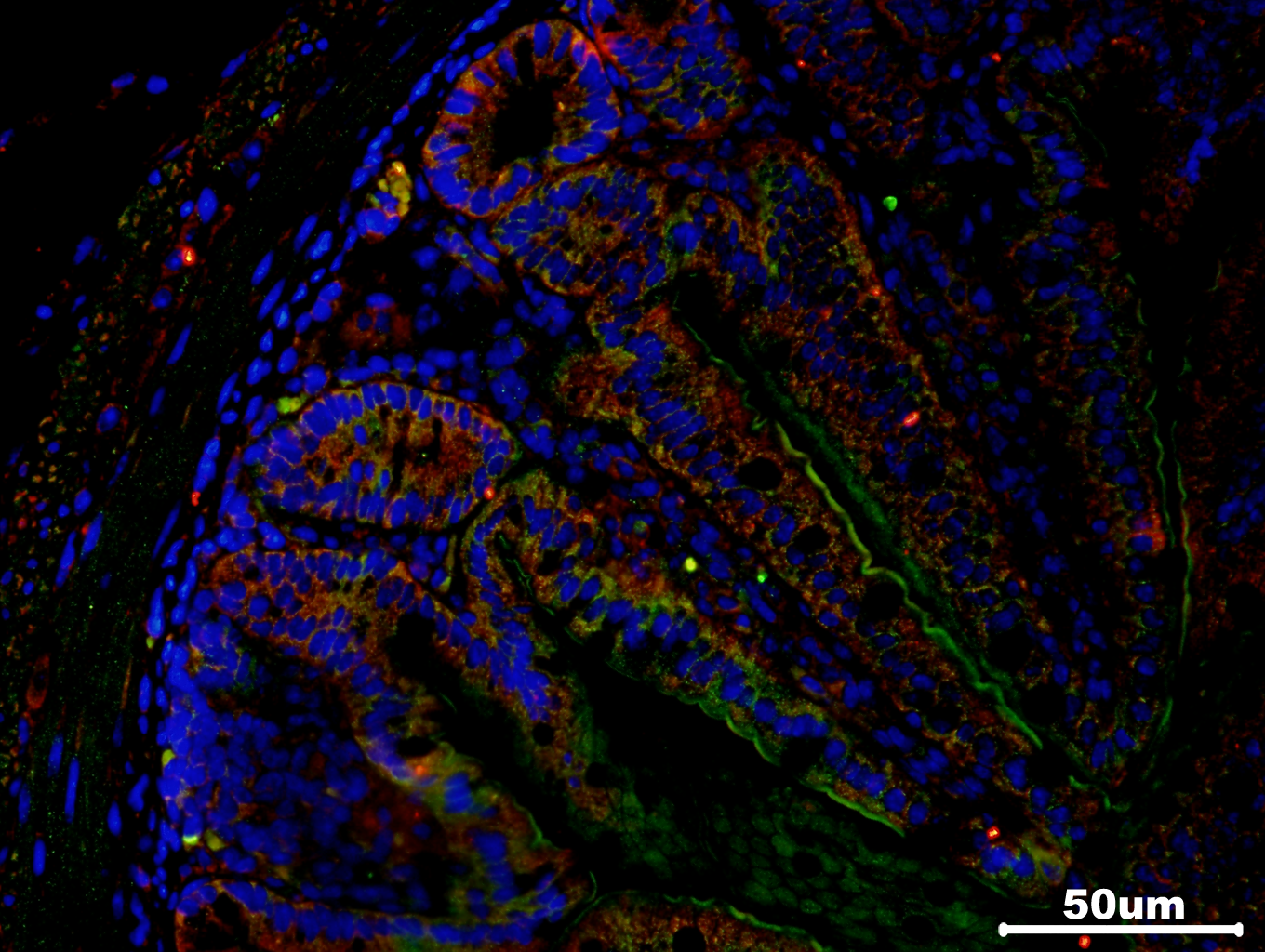

Supplement: Supplementary file 5 [file DataSheet_5.zip › B22-2-200-3-merge.tif]

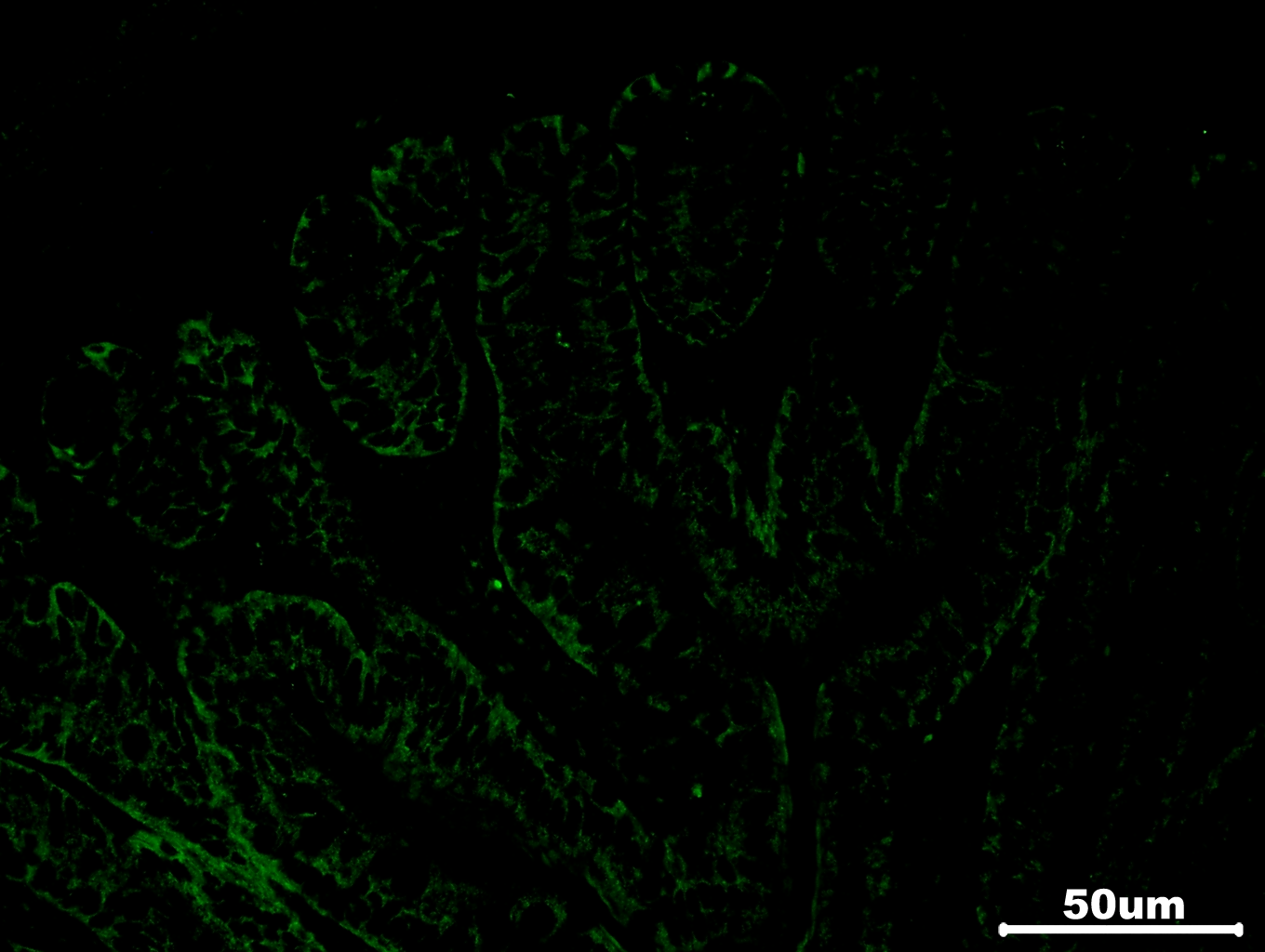

Supplement: Supplementary file 5 [file DataSheet_5.zip › B23-1-200-1-CD86.tif]

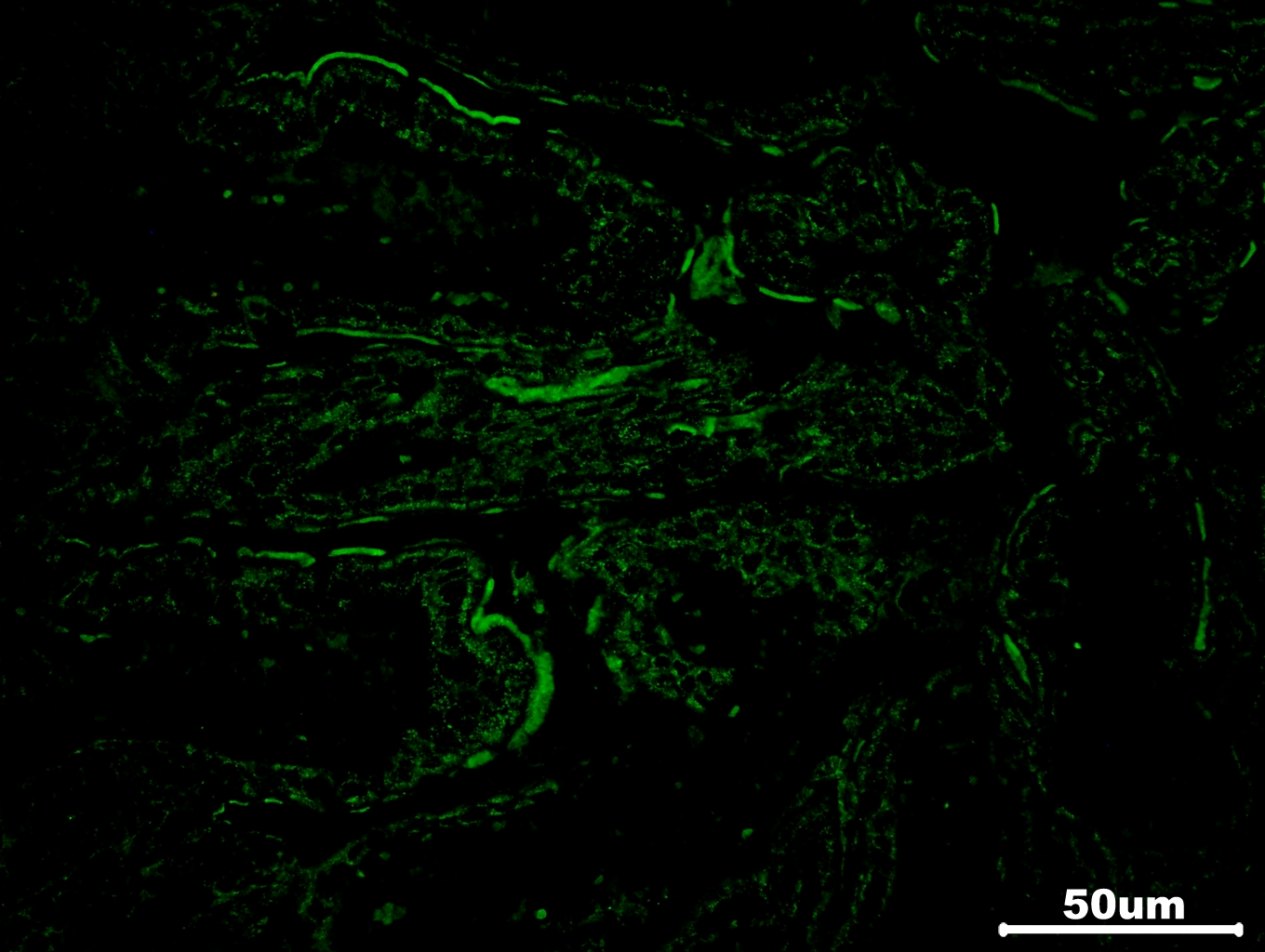

Supplement: Supplementary file 6 [file DataSheet_6.zip › C25-1-200-1-CD86.tif]

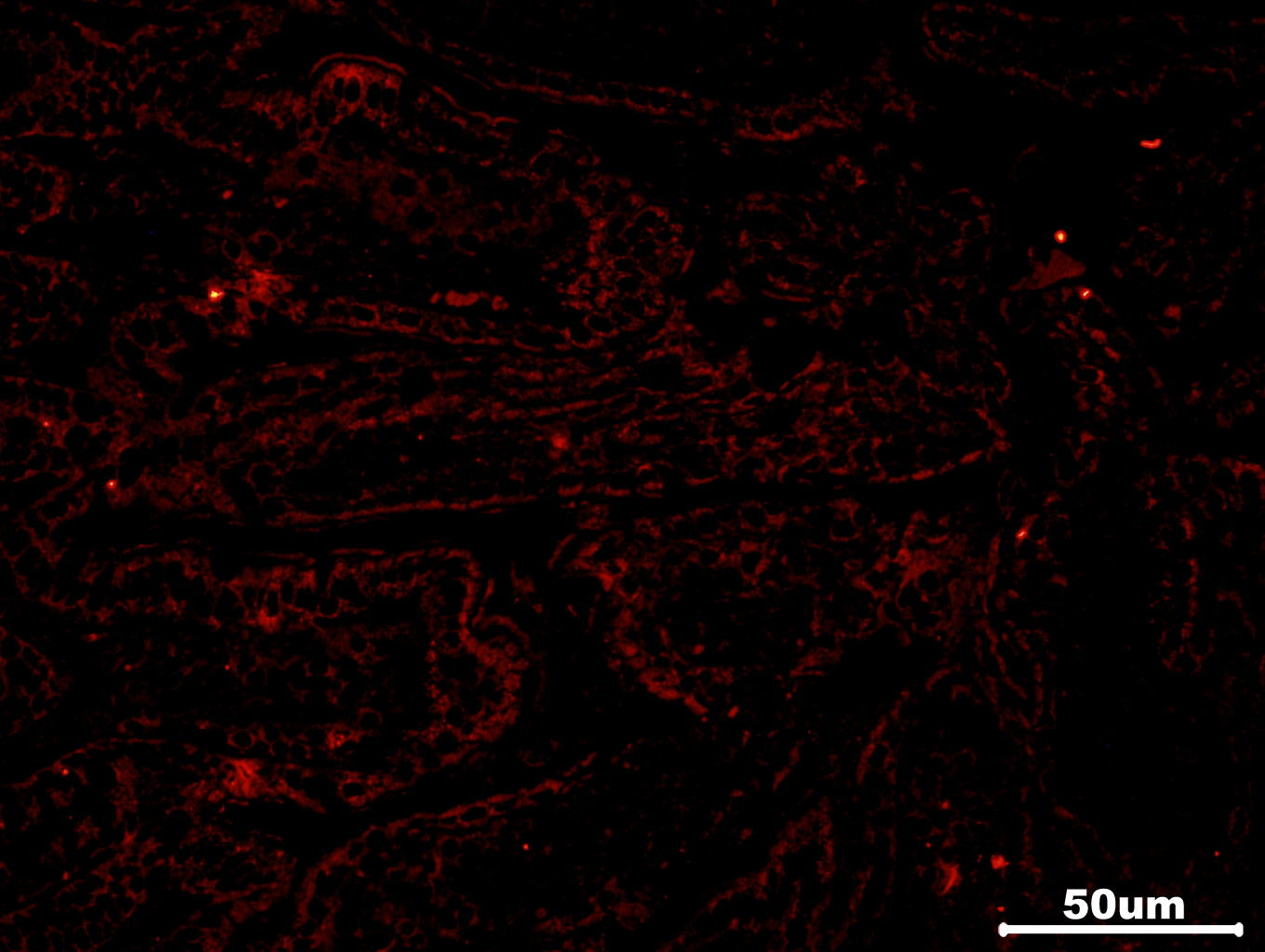

Supplement: Supplementary file 6 [file DataSheet_6.zip › C25-1-200-1-CD206.tif]

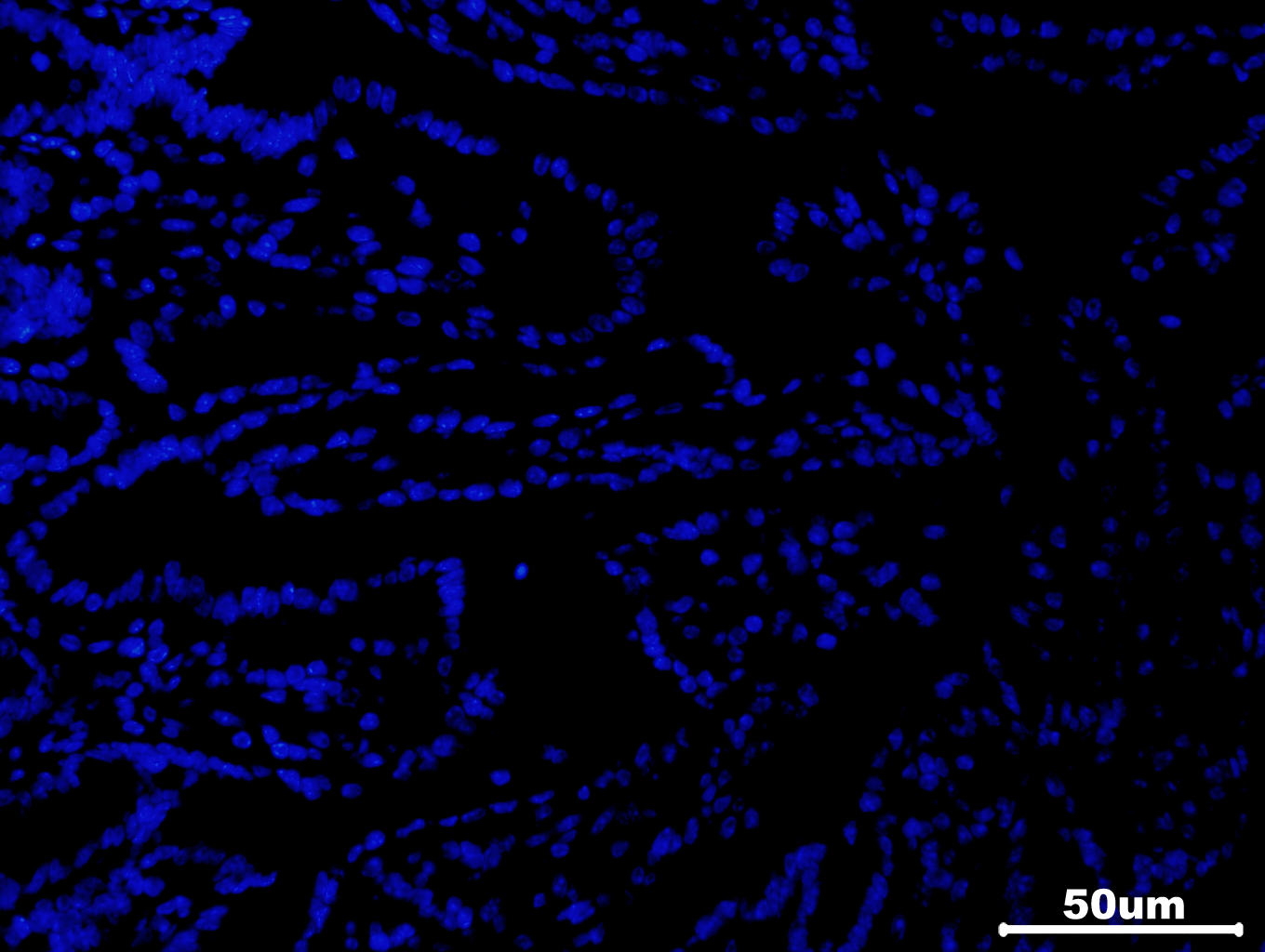

Supplement: Supplementary file 6 [file DataSheet_6.zip › C25-1-200-1-DAPI.tif]

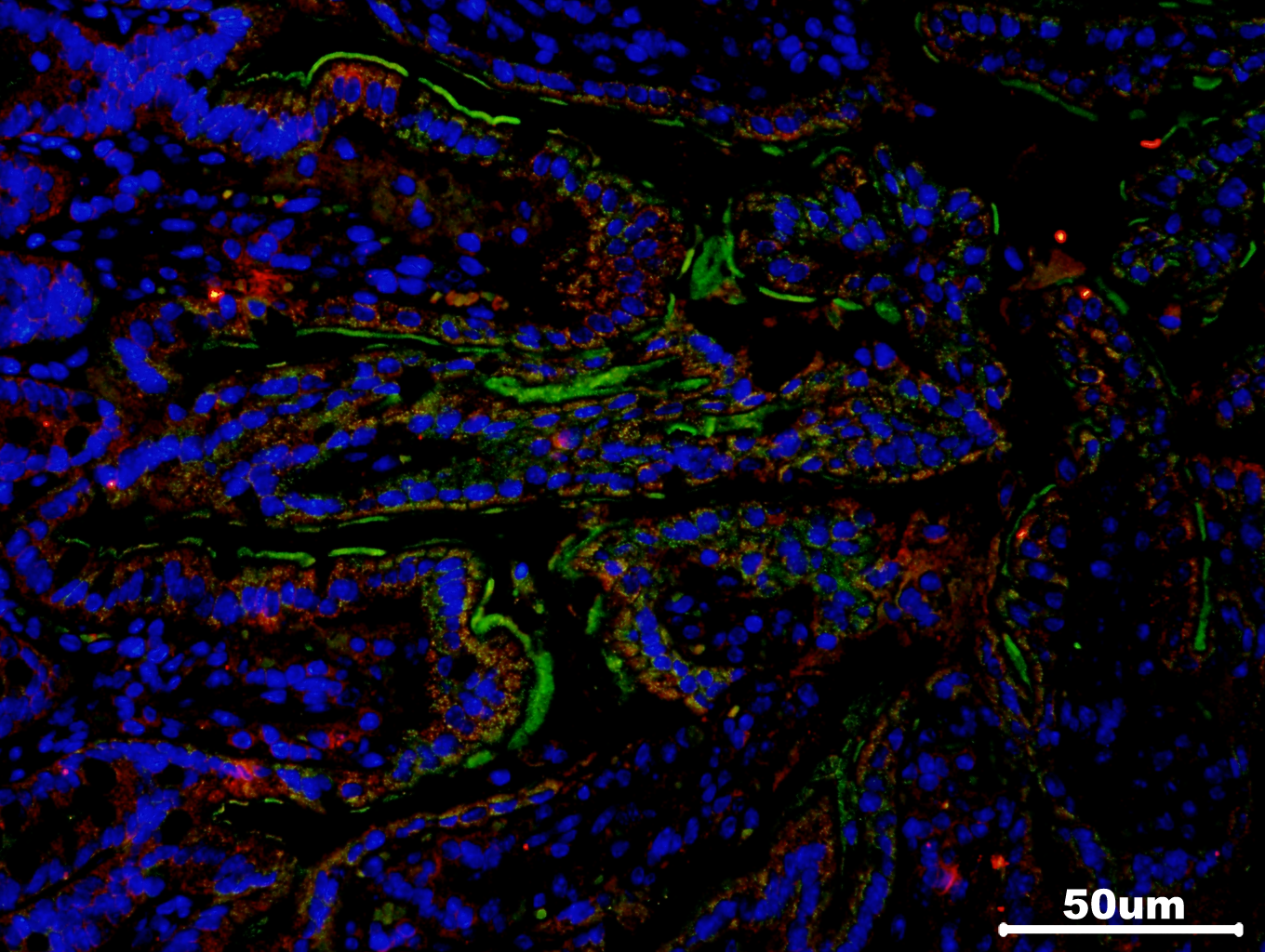

Supplement: Supplementary file 6 [file DataSheet_6.zip › C25-1-200-1-merge.tif]

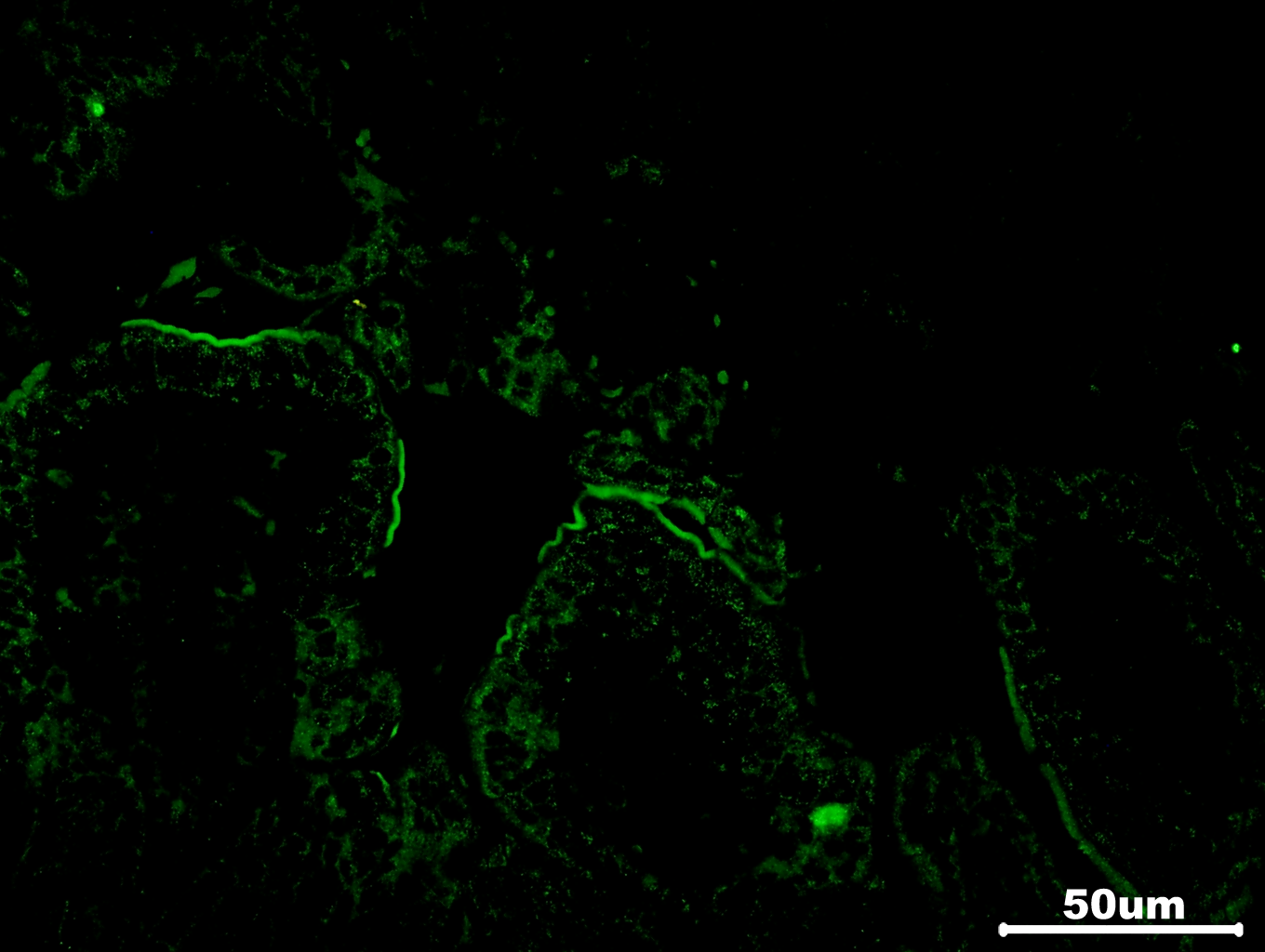

Supplement: Supplementary file 6 [file DataSheet_6.zip › C25-1-200-2-CD86.tif]

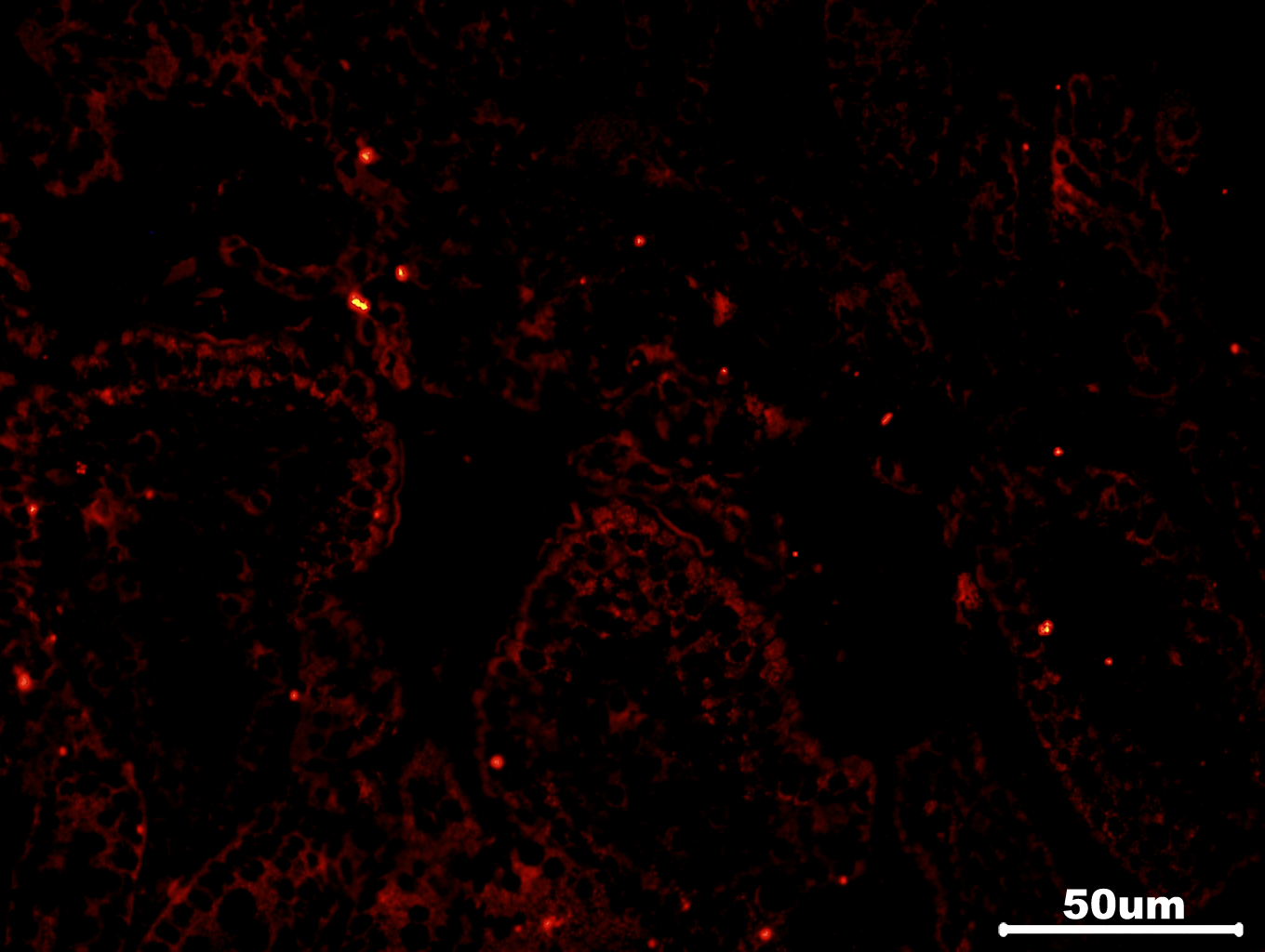

Supplement: Supplementary file 6 [file DataSheet_6.zip › C25-1-200-2-CD206.tif]

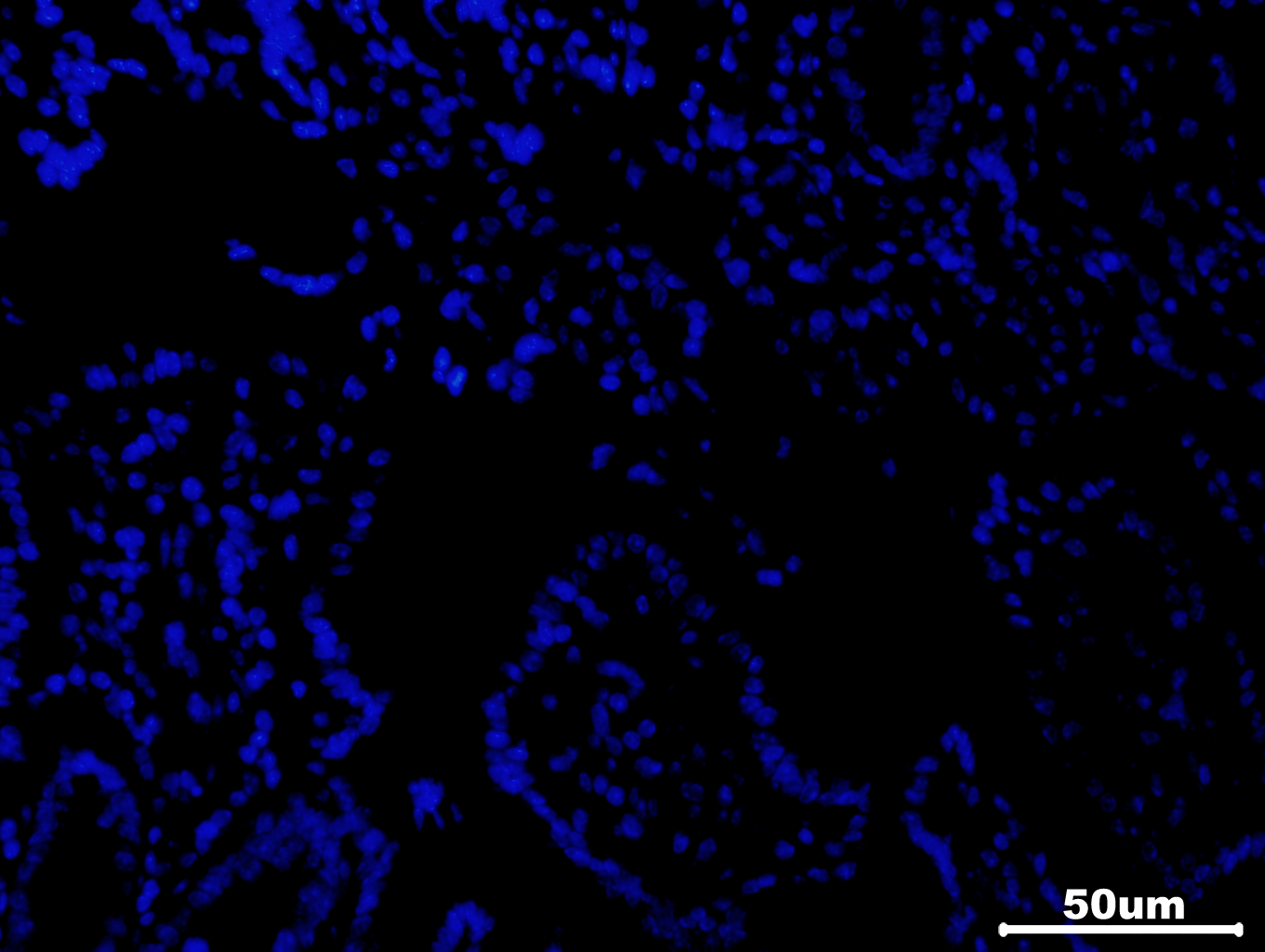

Supplement: Supplementary file 6 [file DataSheet_6.zip › C25-1-200-2-DAPI.tif]

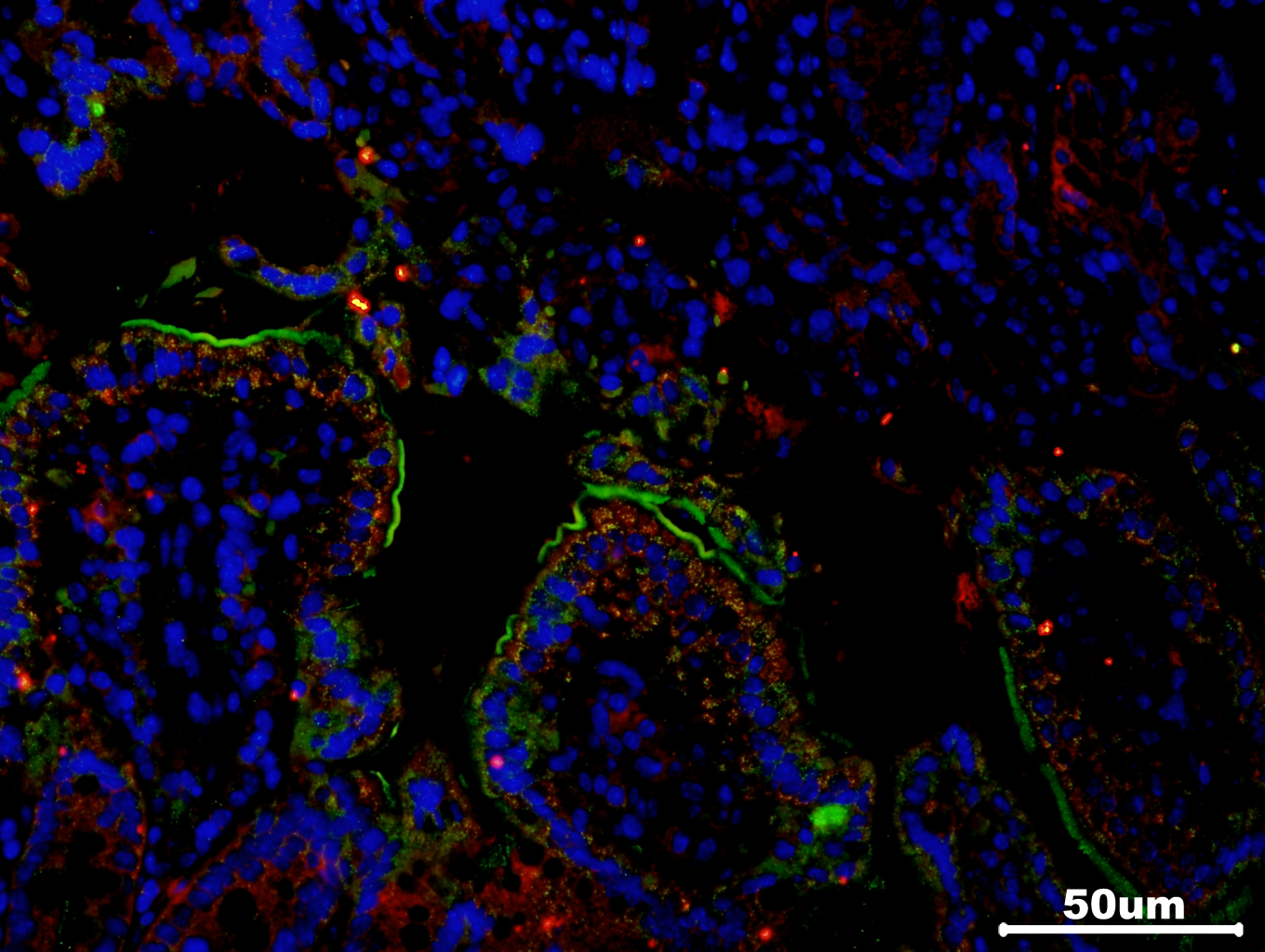

Supplement: Supplementary file 6 [file DataSheet_6.zip › C25-1-200-2-merge.tif]

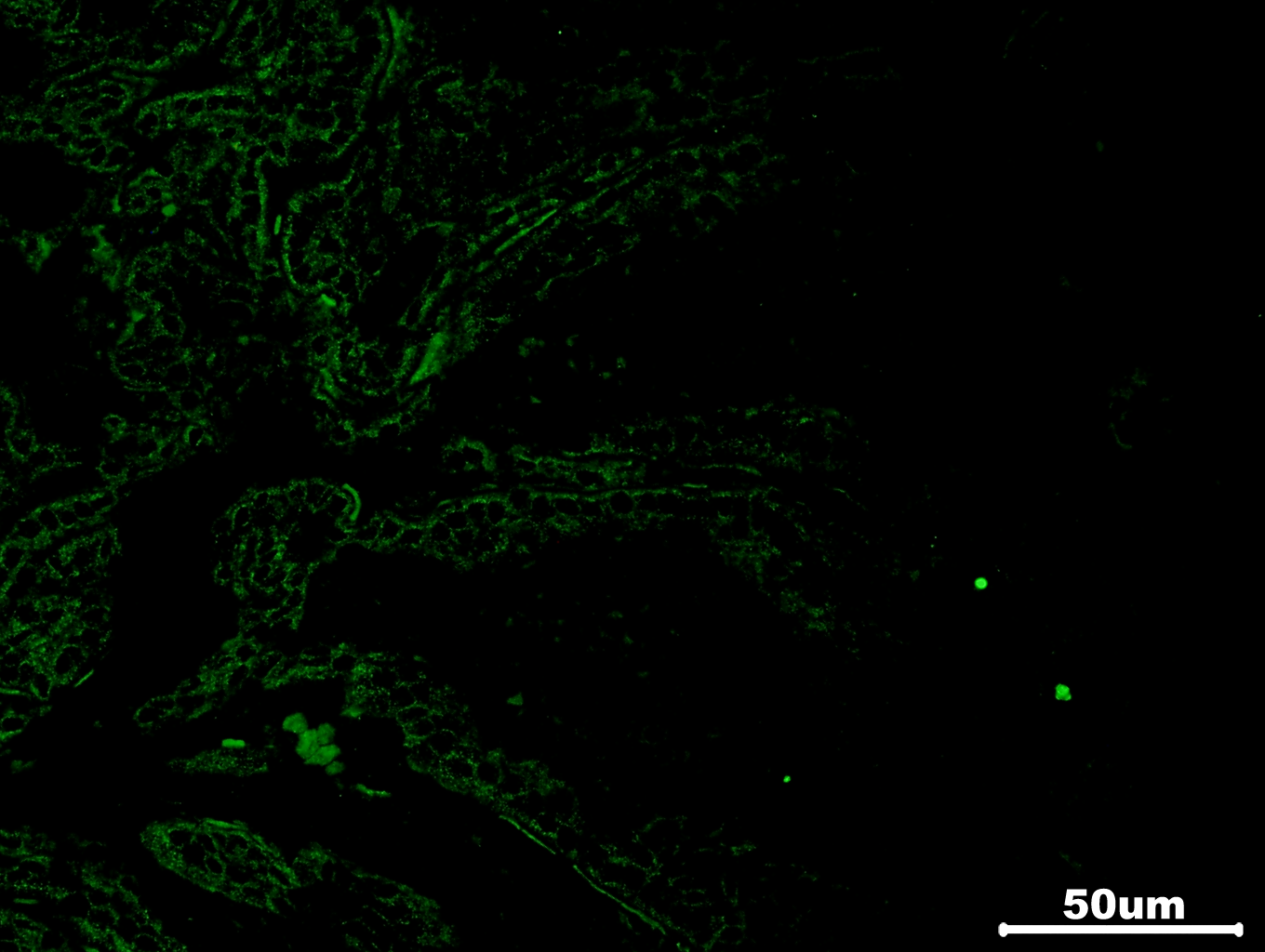

Supplement: Supplementary file 6 [file DataSheet_6.zip › C25-1-200-3-CD86.tif]

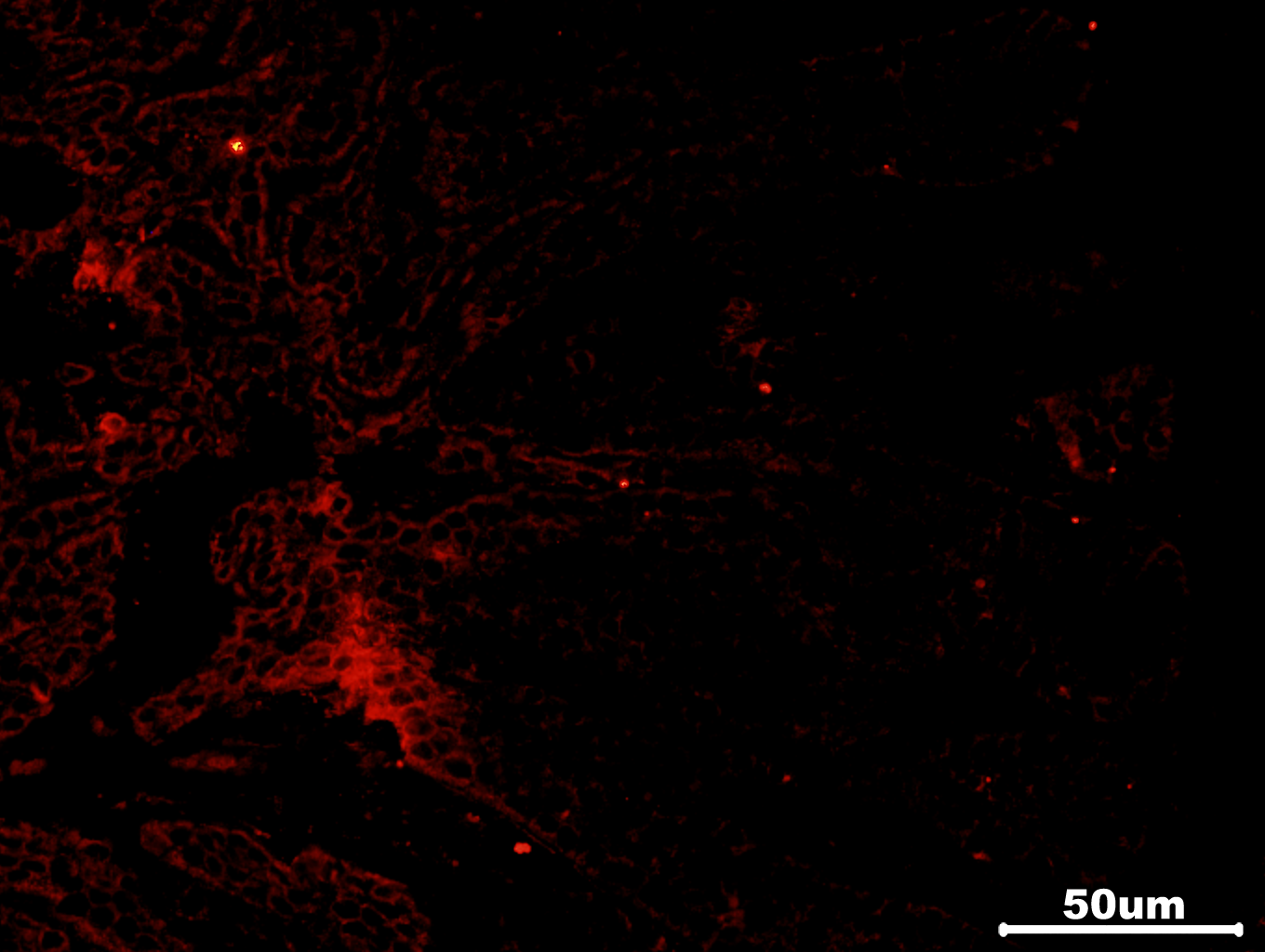

Supplement: Supplementary file 6 [file DataSheet_6.zip › C25-1-200-3-CD206.tif]

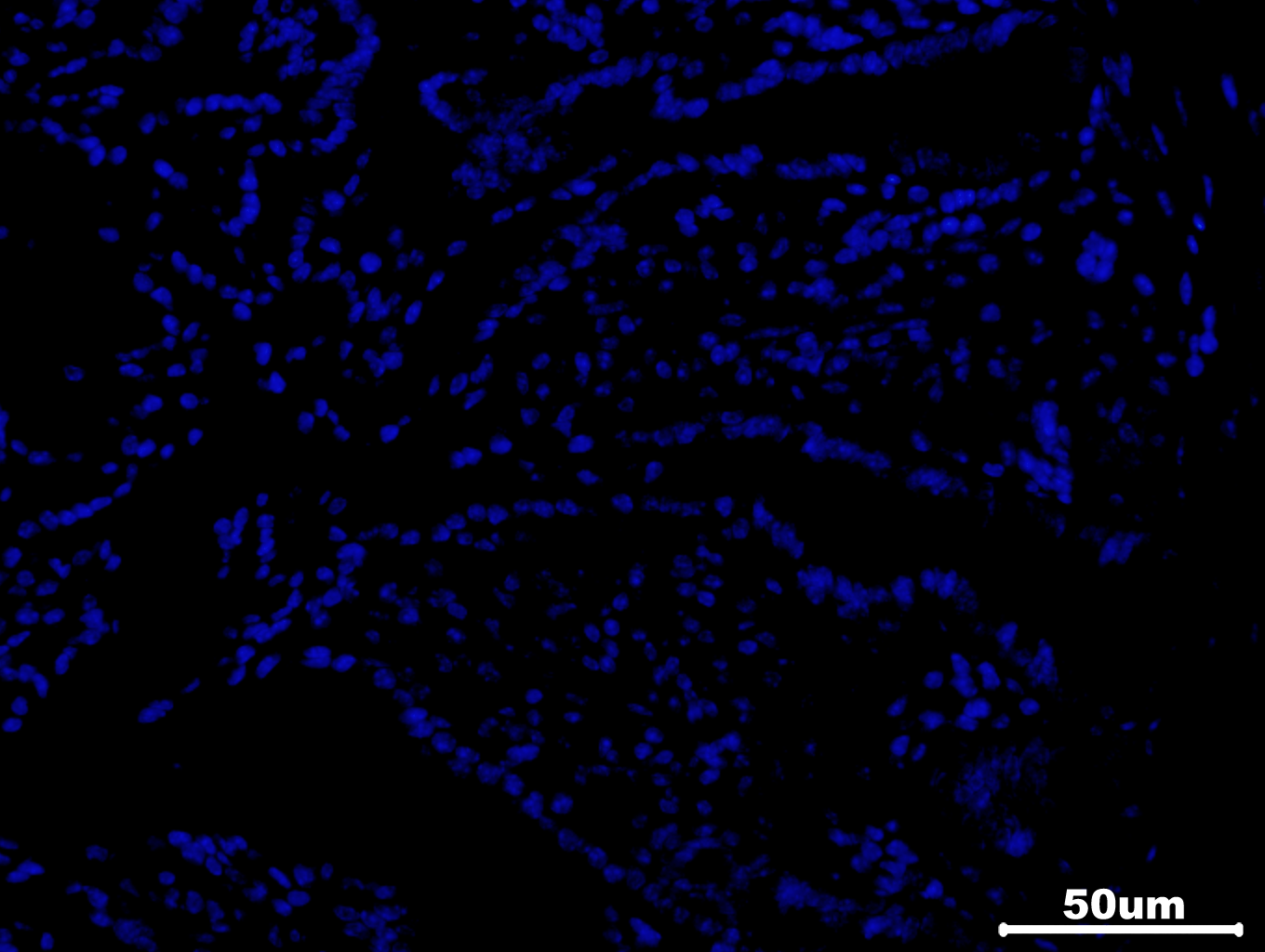

Supplement: Supplementary file 6 [file DataSheet_6.zip › C25-1-200-3-DAPI.tif]

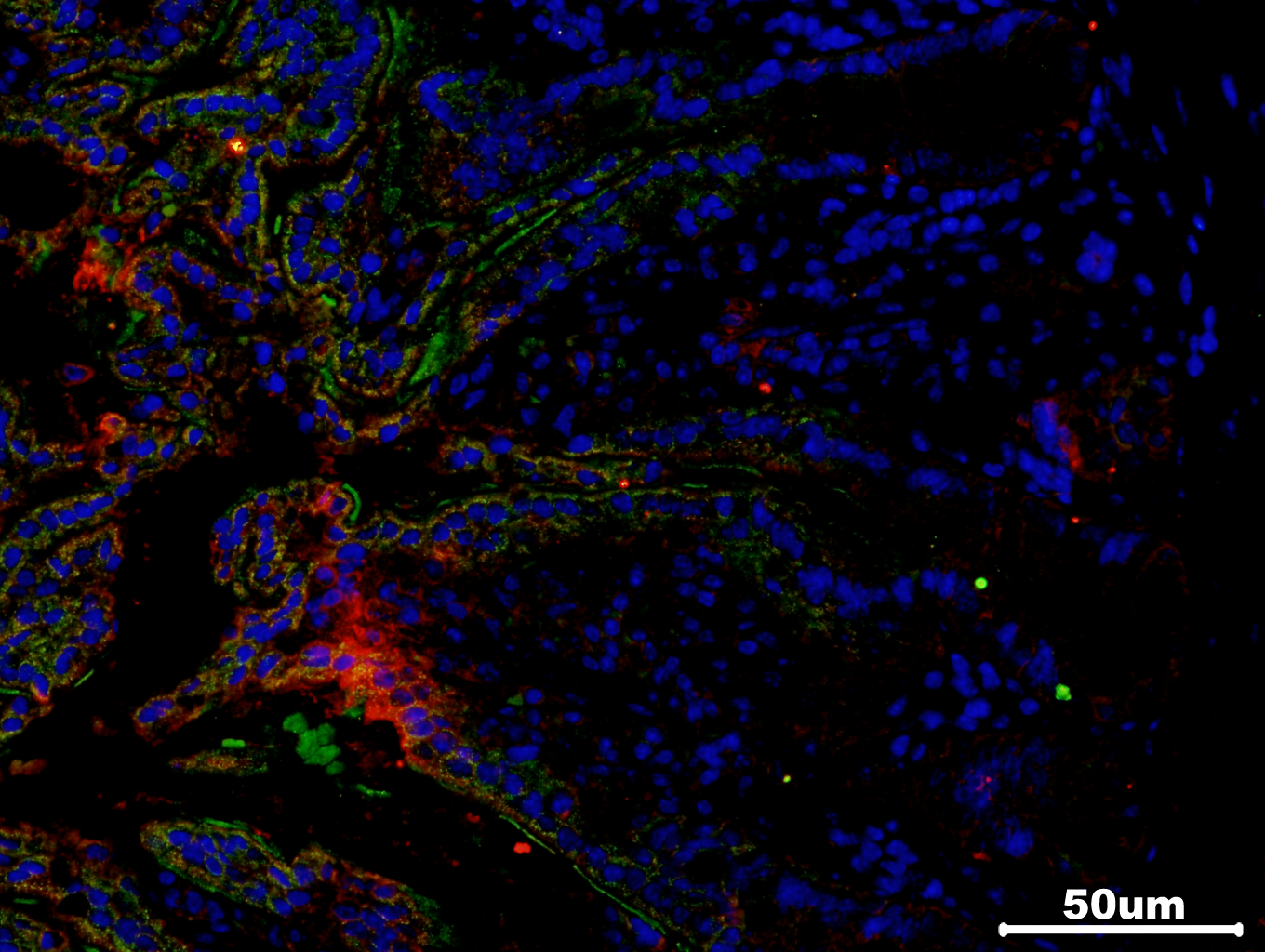

Supplement: Supplementary file 6 [file DataSheet_6.zip › C25-1-200-3-merge.tif]

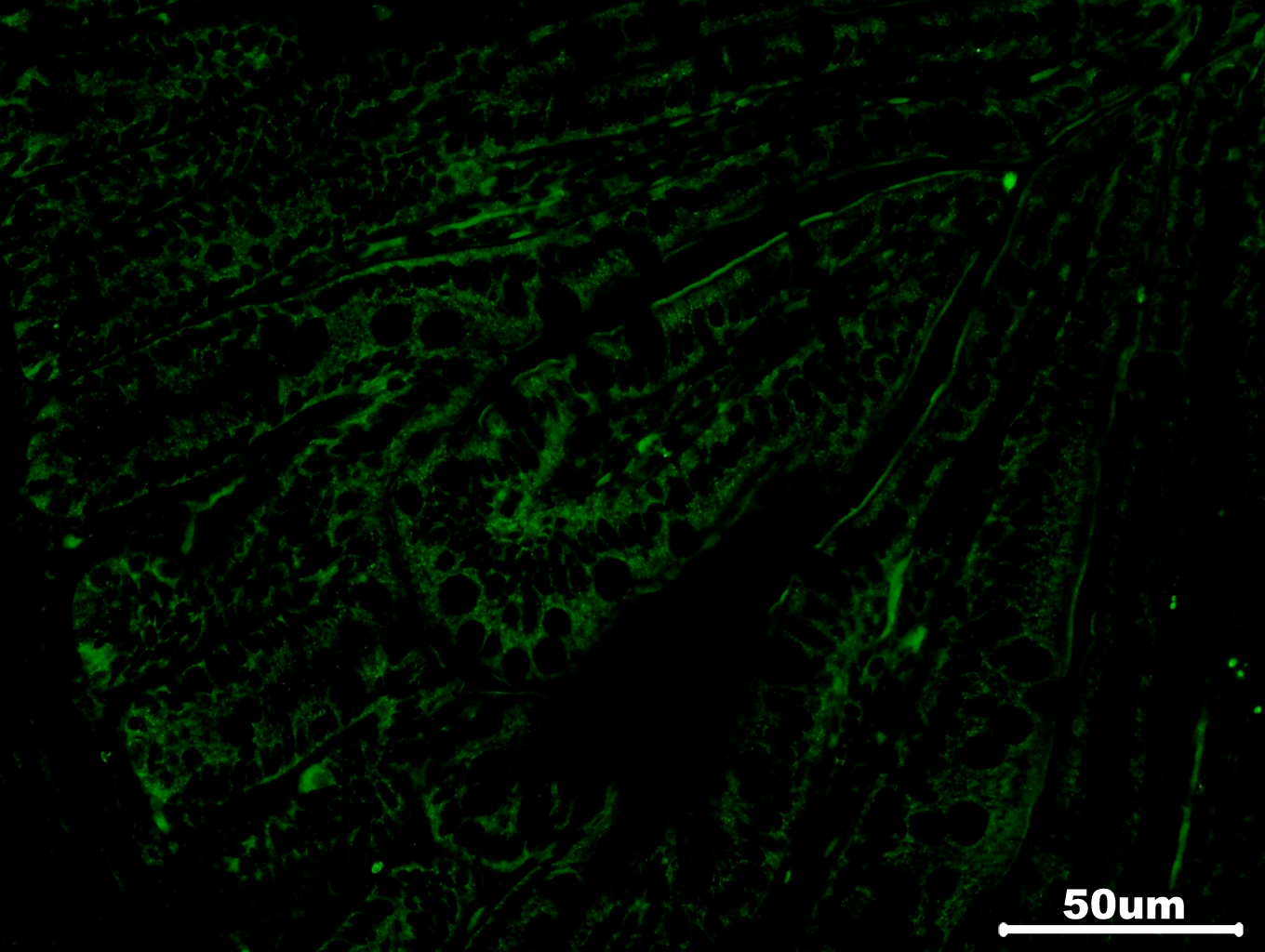

Supplement: Supplementary file 6 [file DataSheet_6.zip › C26-1-200-1-CD86.tif]
