# Supplementary figures and images for: Mechanism of electro-acupuncture in alleviating intestinal injury in septic mice via polyamine-related M2-macrophage polarization (part 2 of 3)
Source: Front Immunol. 2024 Apr 22;15:1373876. doi: 10.3389/fimmu.2024.1373876 (PMC11075497; doi:10.3389/fimmu.2024.1373876)

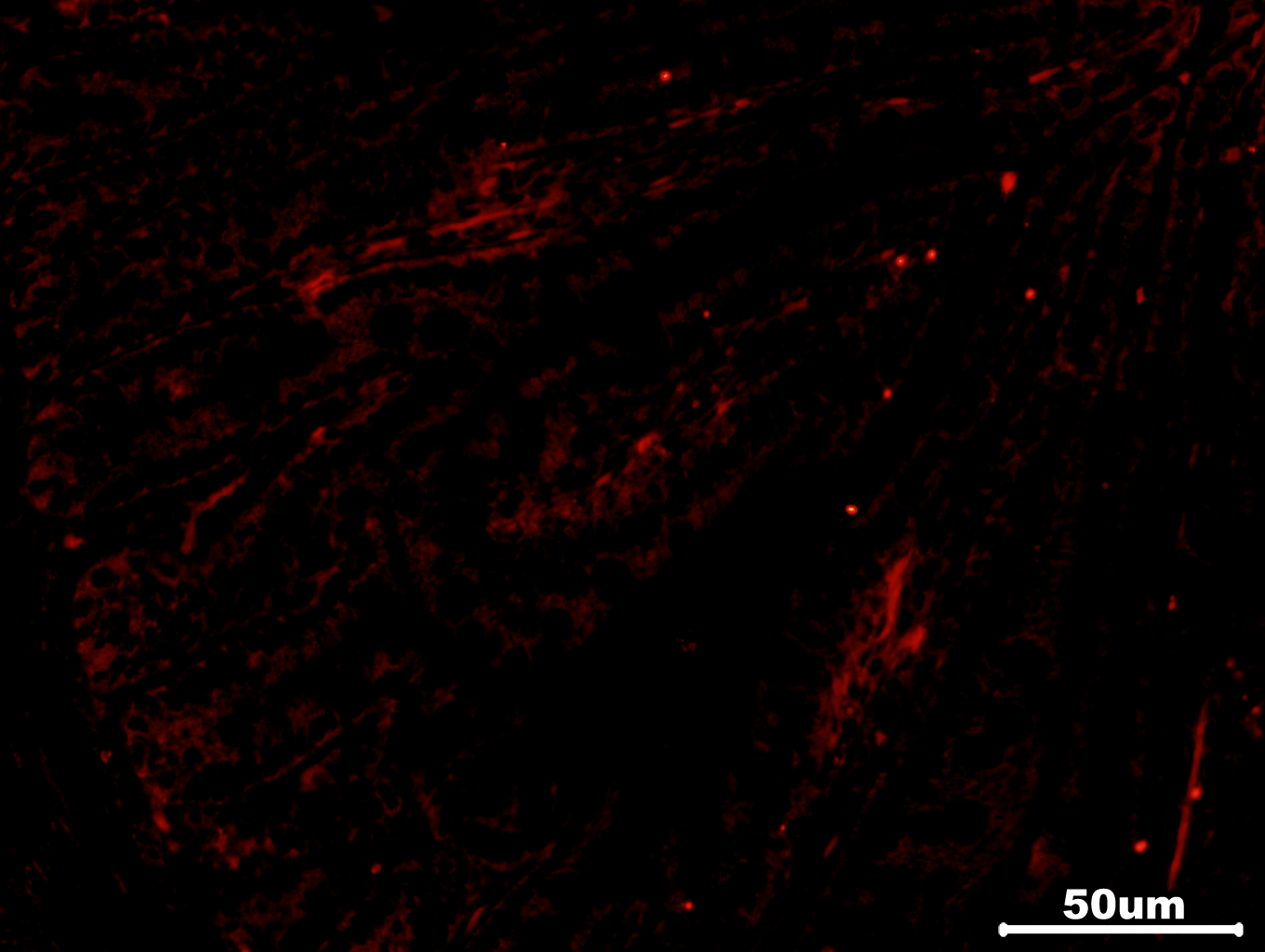

Supplement: Supplementary file 6 [file DataSheet_6.zip › C26-1-200-1-CD206.tif]

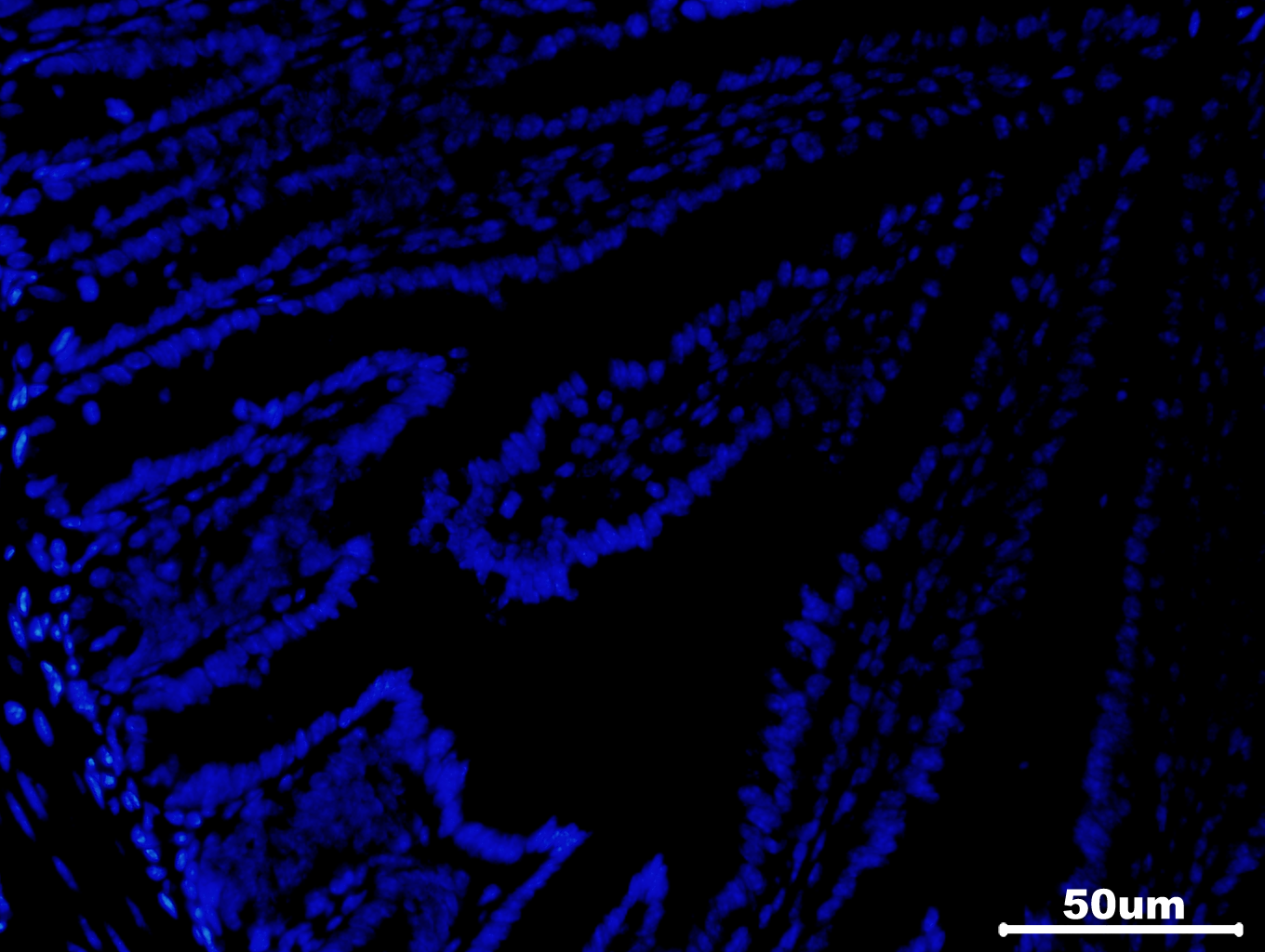

Supplement: Supplementary file 6 [file DataSheet_6.zip › C26-1-200-1-DAPI.tif]

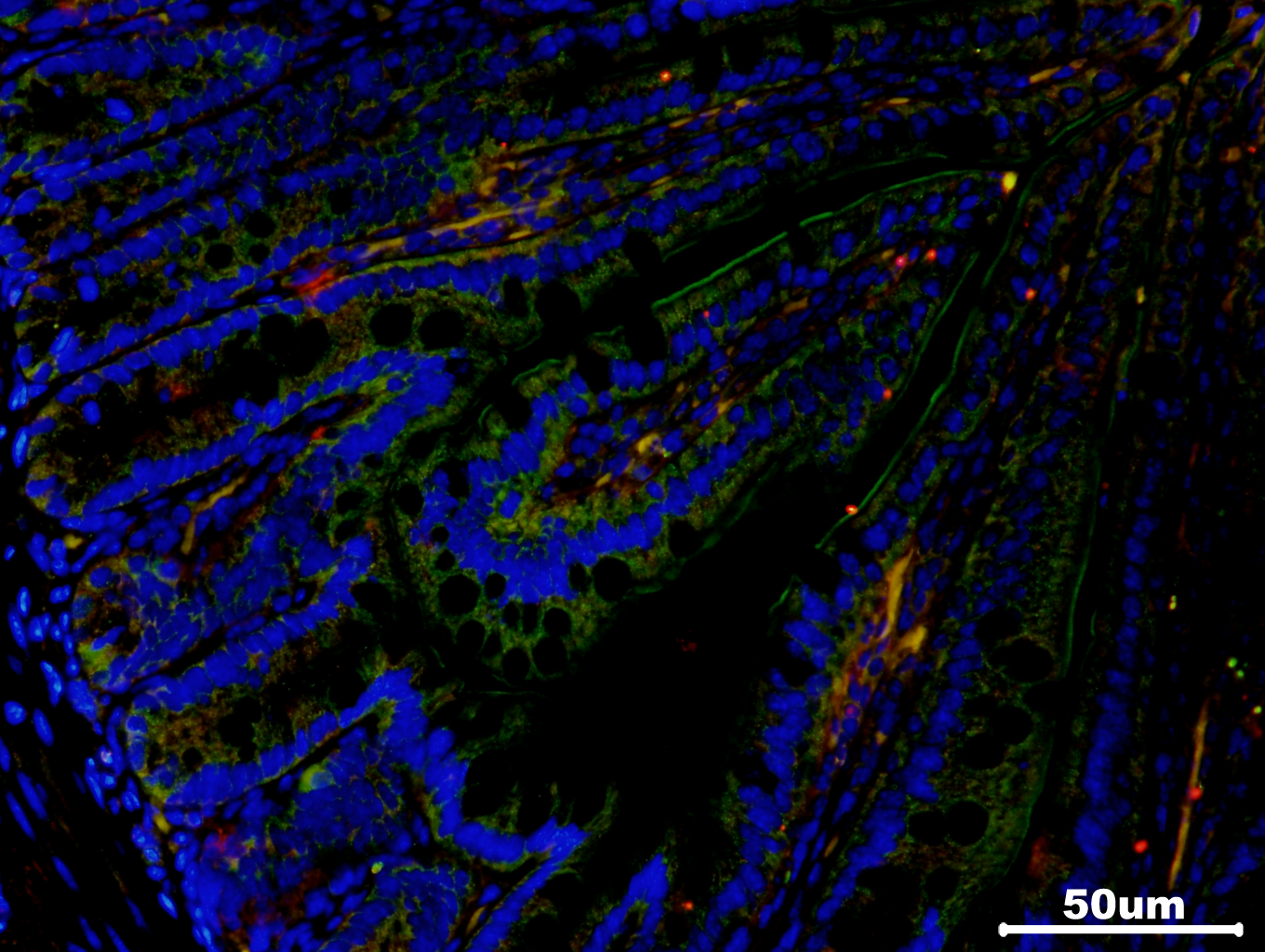

Supplement: Supplementary file 6 [file DataSheet_6.zip › C26-1-200-1-merge.tif]

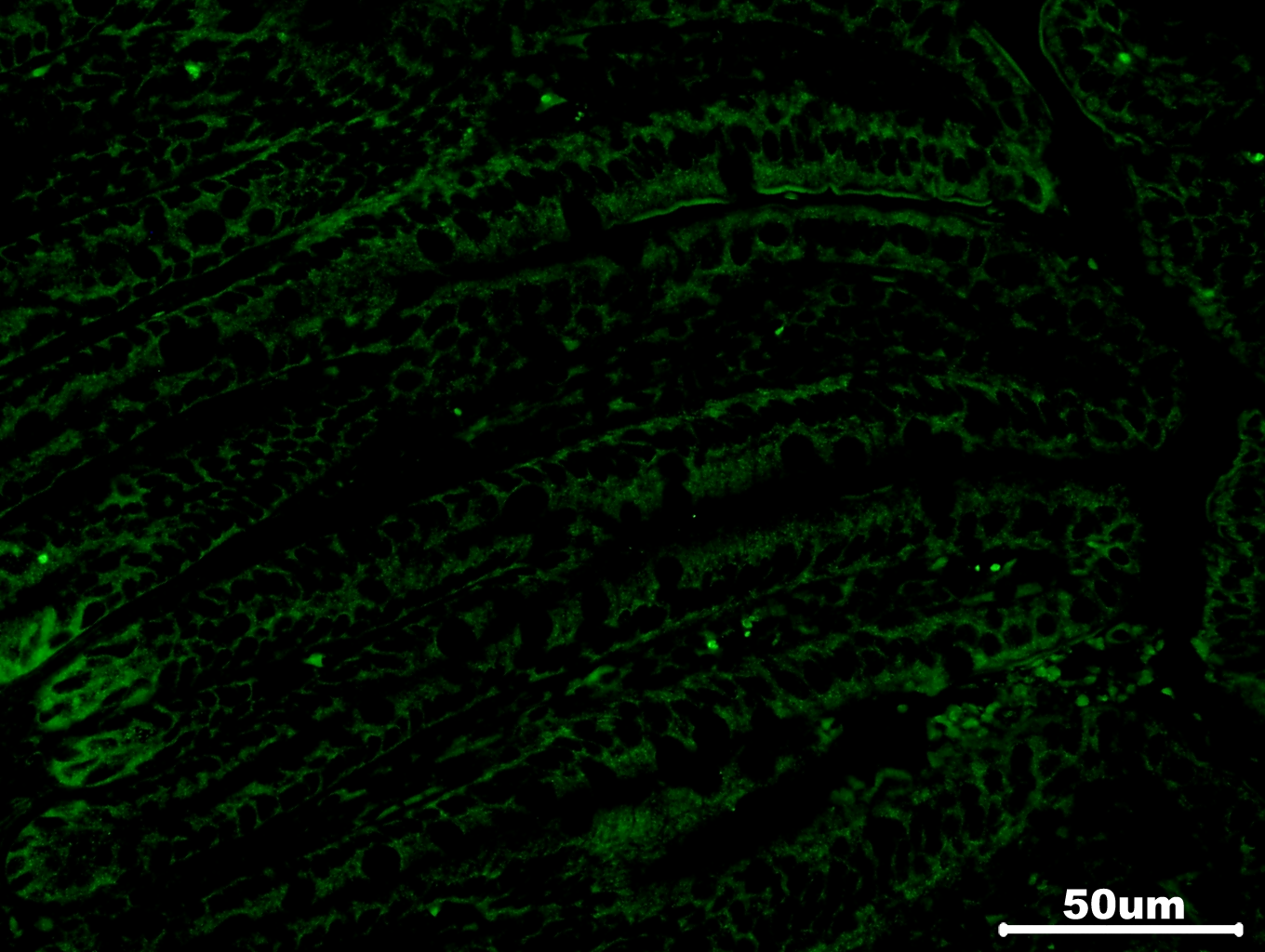

Supplement: Supplementary file 6 [file DataSheet_6.zip › C26-1-200-2-CD86.tif]

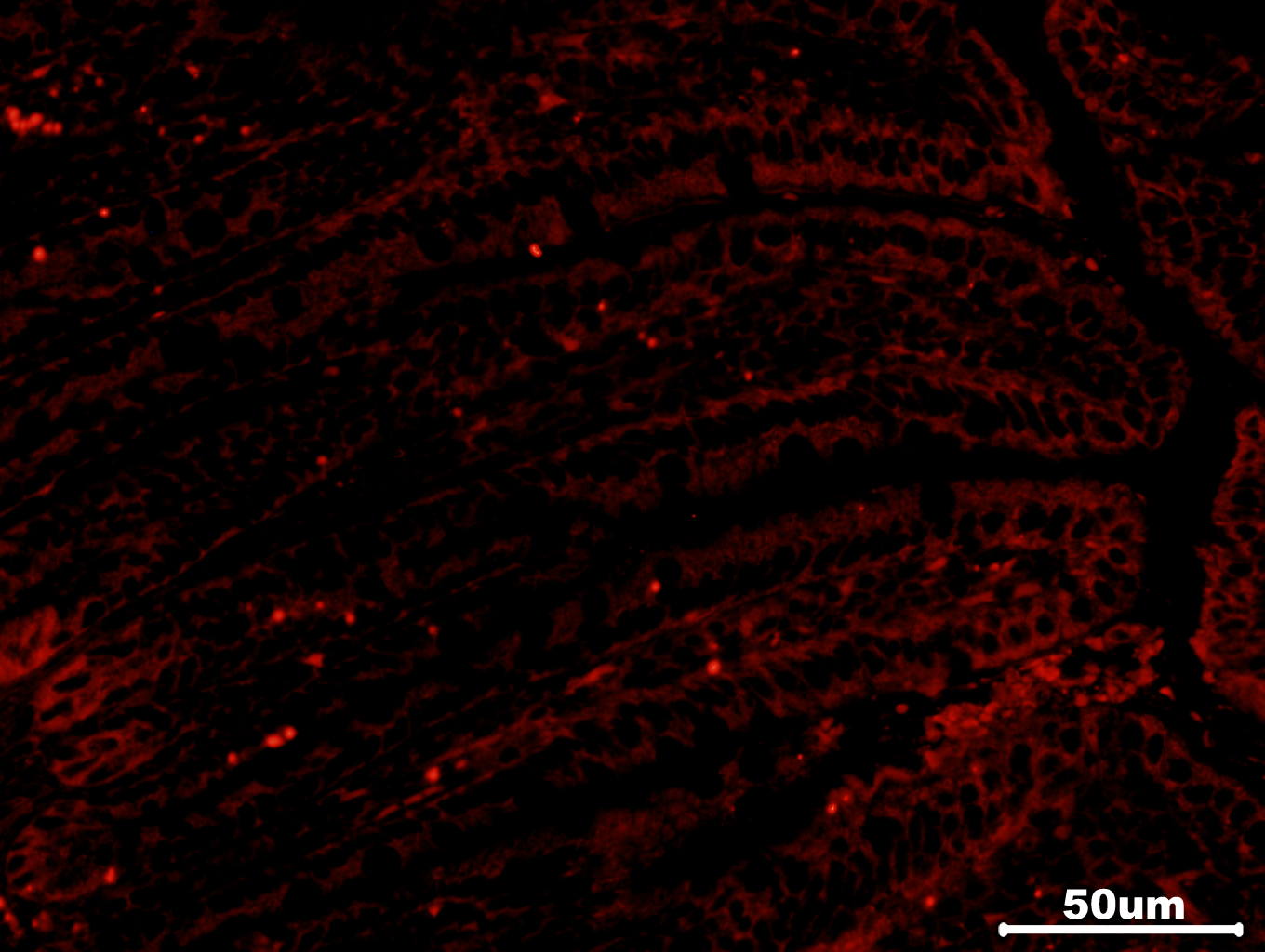

Supplement: Supplementary file 6 [file DataSheet_6.zip › C26-1-200-2-CD206.tif]

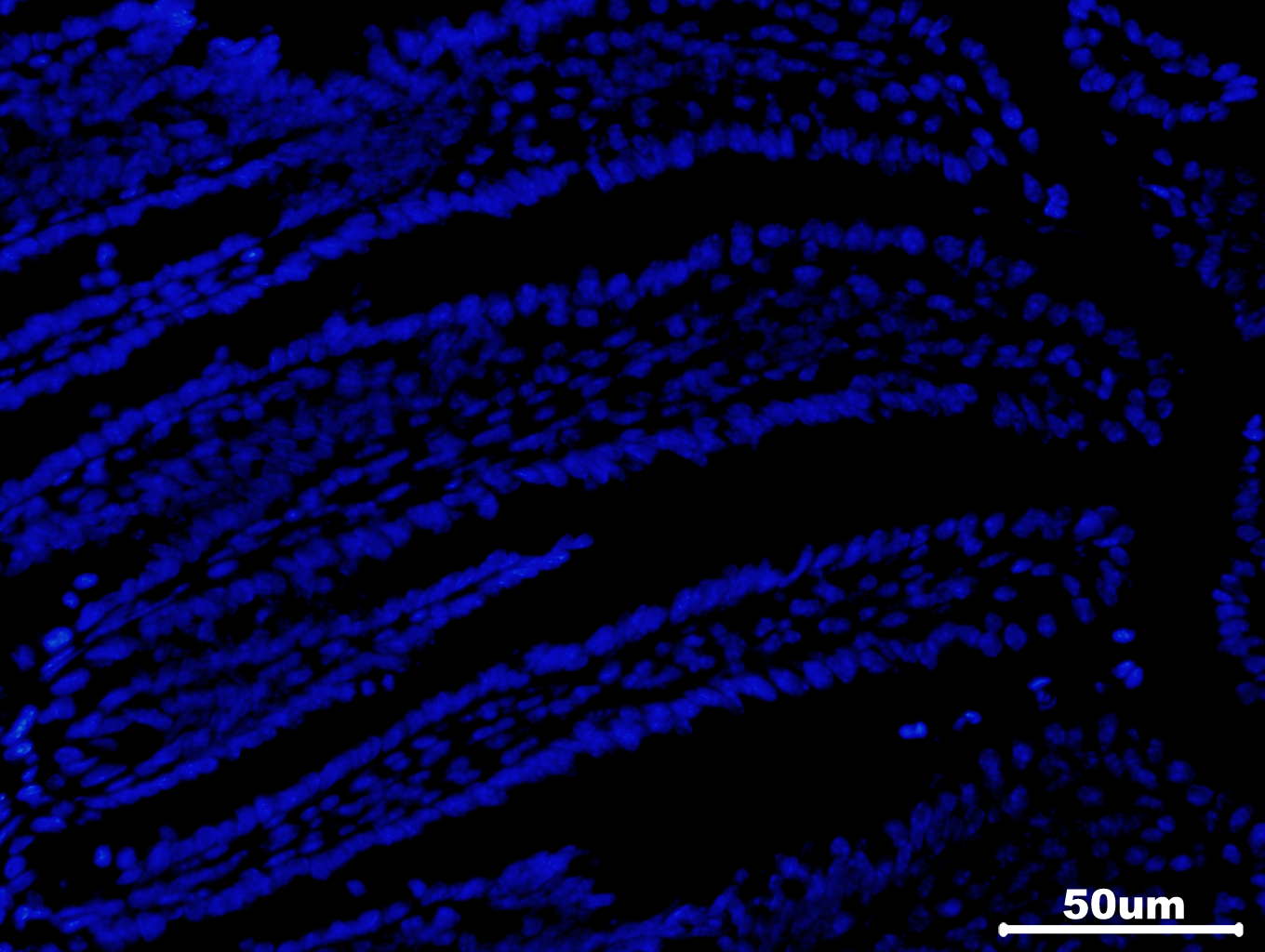

Supplement: Supplementary file 6 [file DataSheet_6.zip › C26-1-200-2-DAPI.tif]

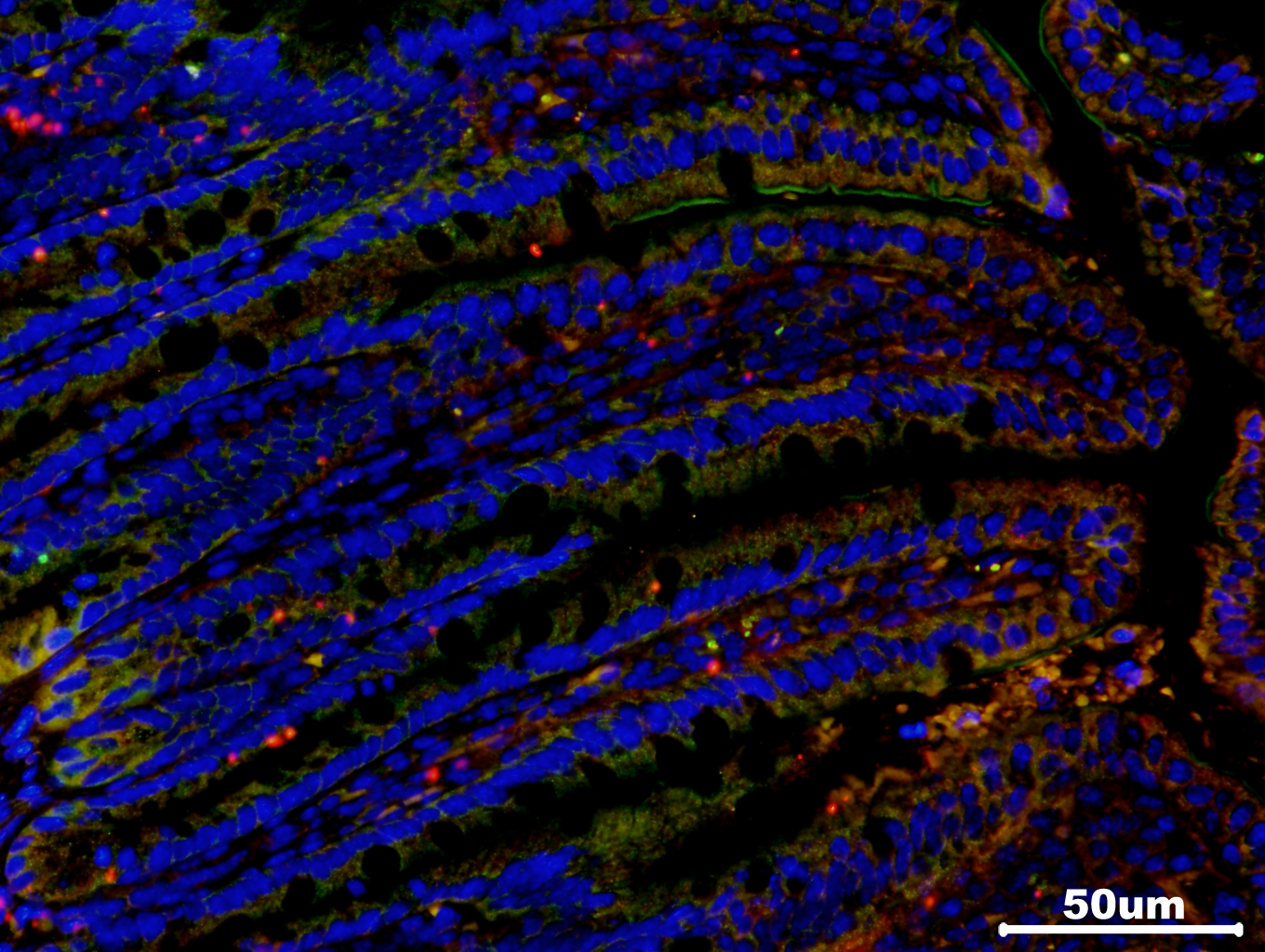

Supplement: Supplementary file 6 [file DataSheet_6.zip › C26-1-200-2-merge.tif]

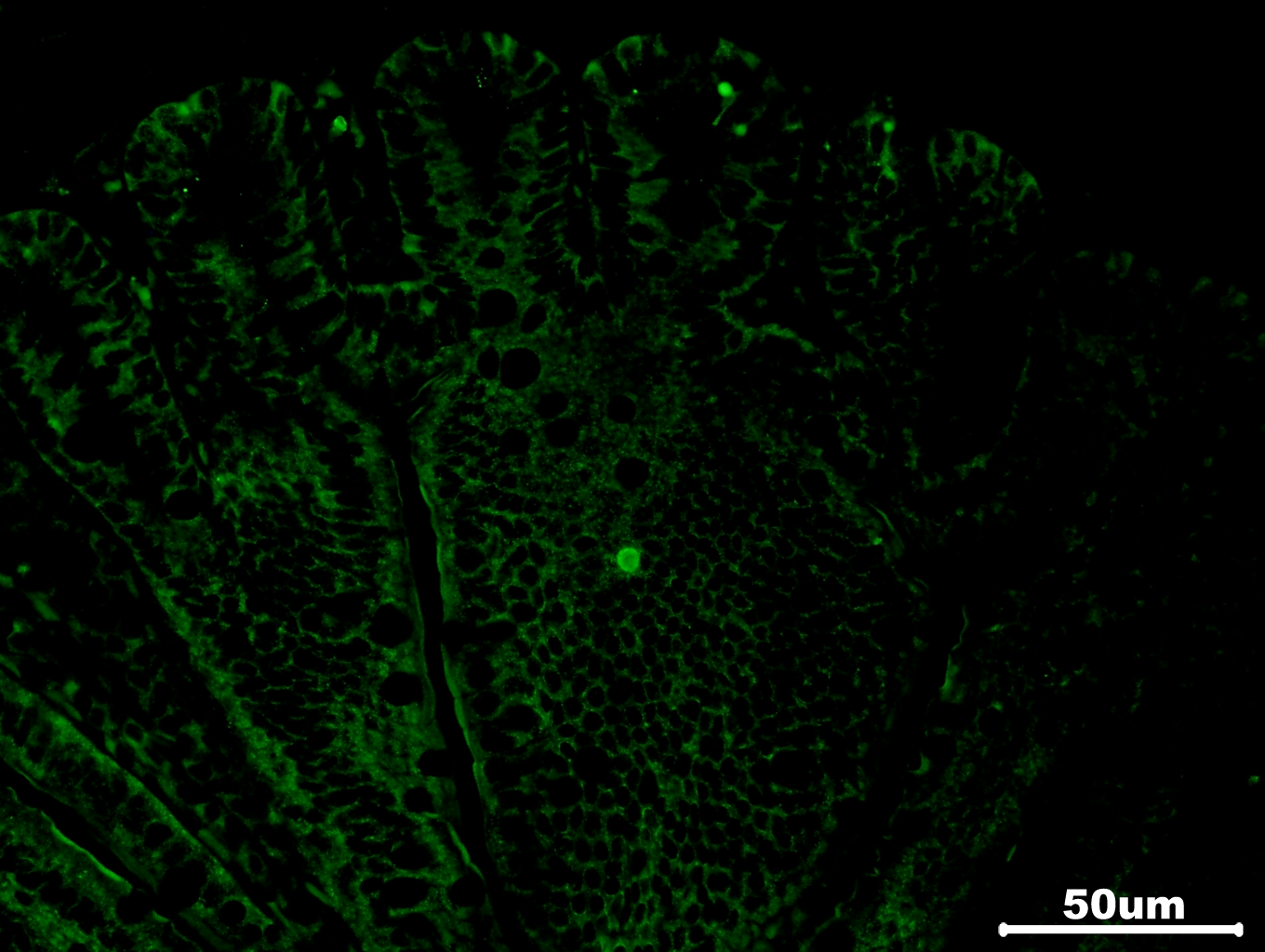

Supplement: Supplementary file 6 [file DataSheet_6.zip › C26-1-200-3-CD86.tif]

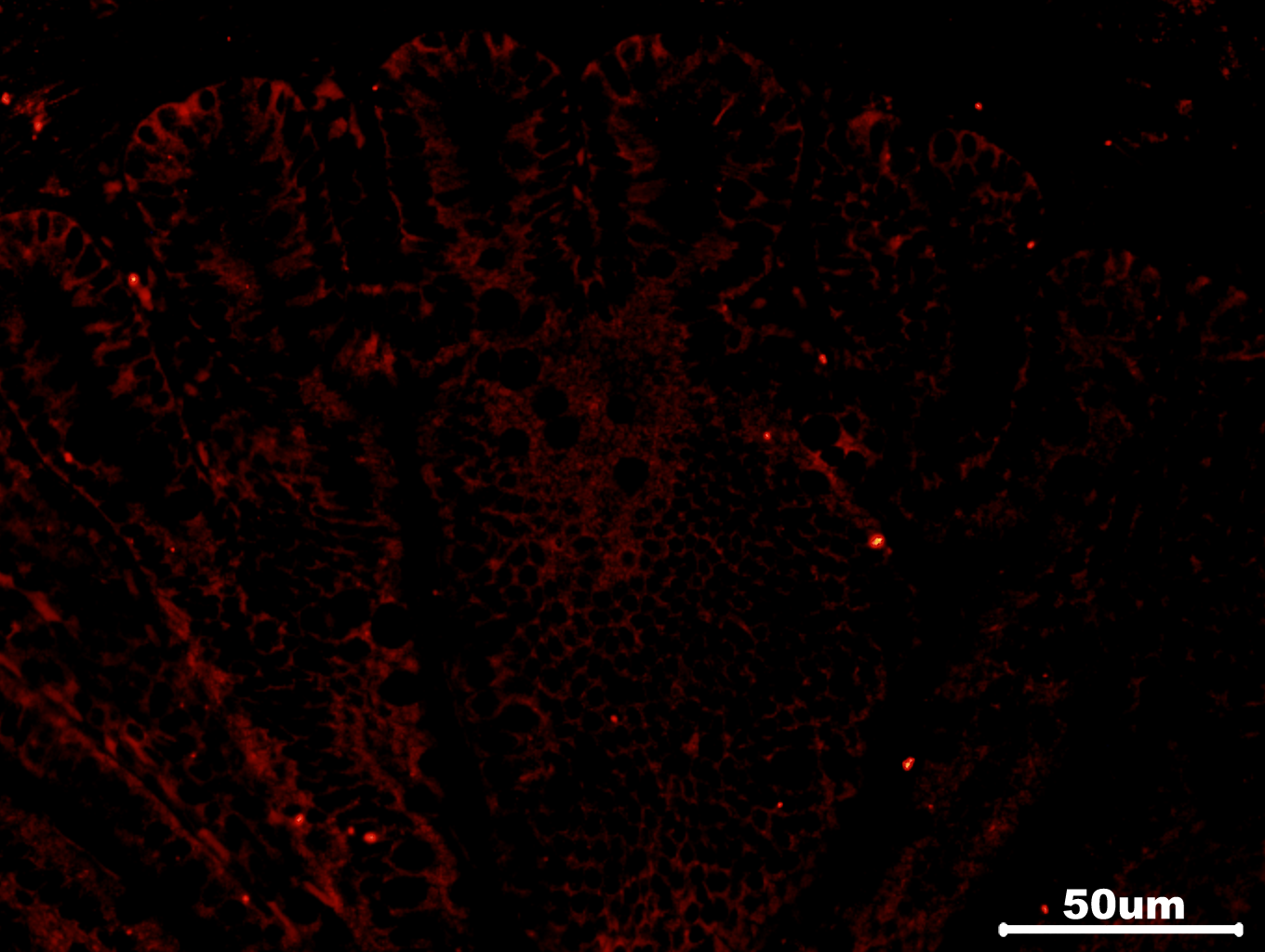

Supplement: Supplementary file 6 [file DataSheet_6.zip › C26-1-200-3-CD206.tif]

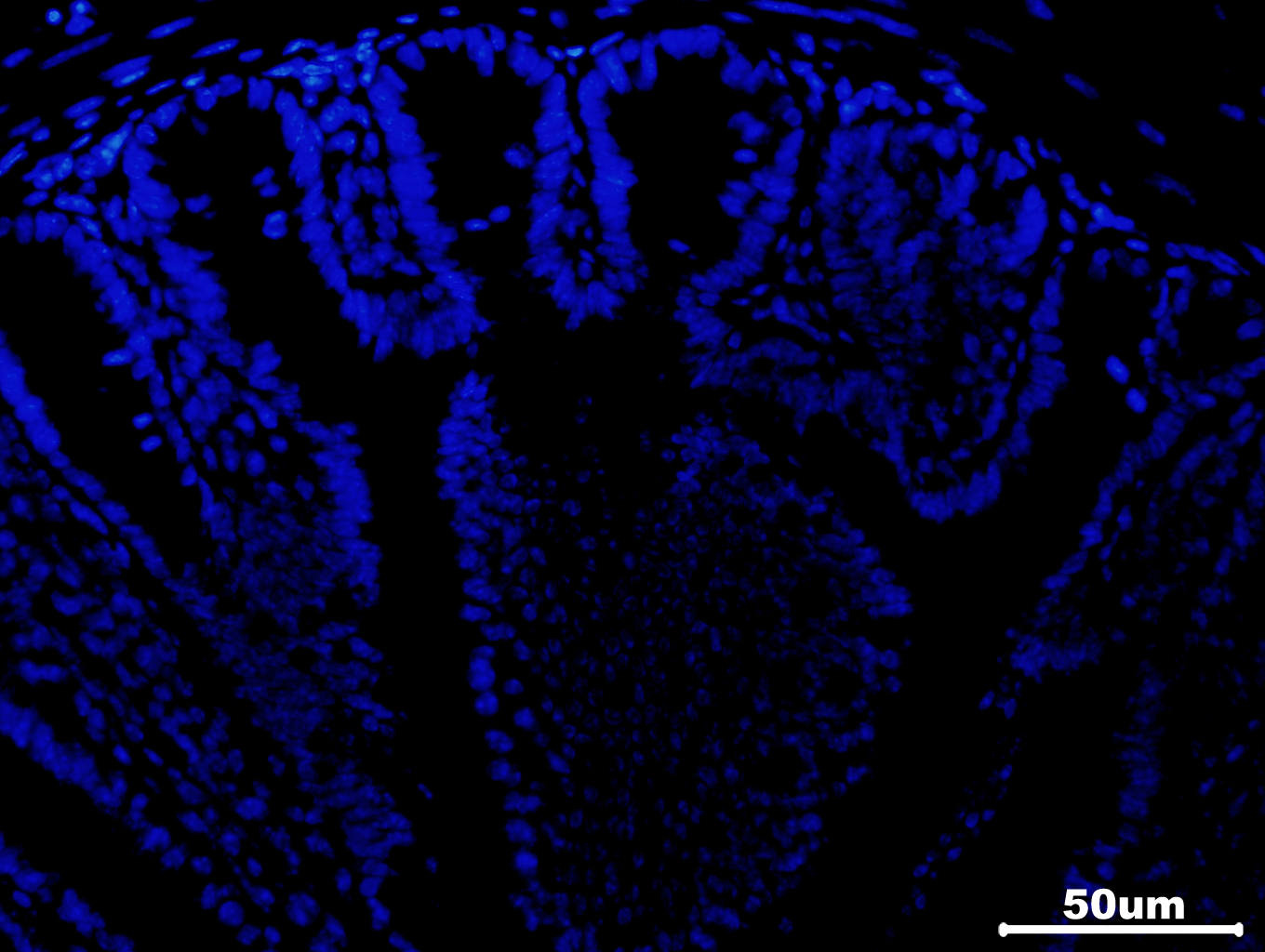

Supplement: Supplementary file 6 [file DataSheet_6.zip › C26-1-200-3-DAPI.tif]

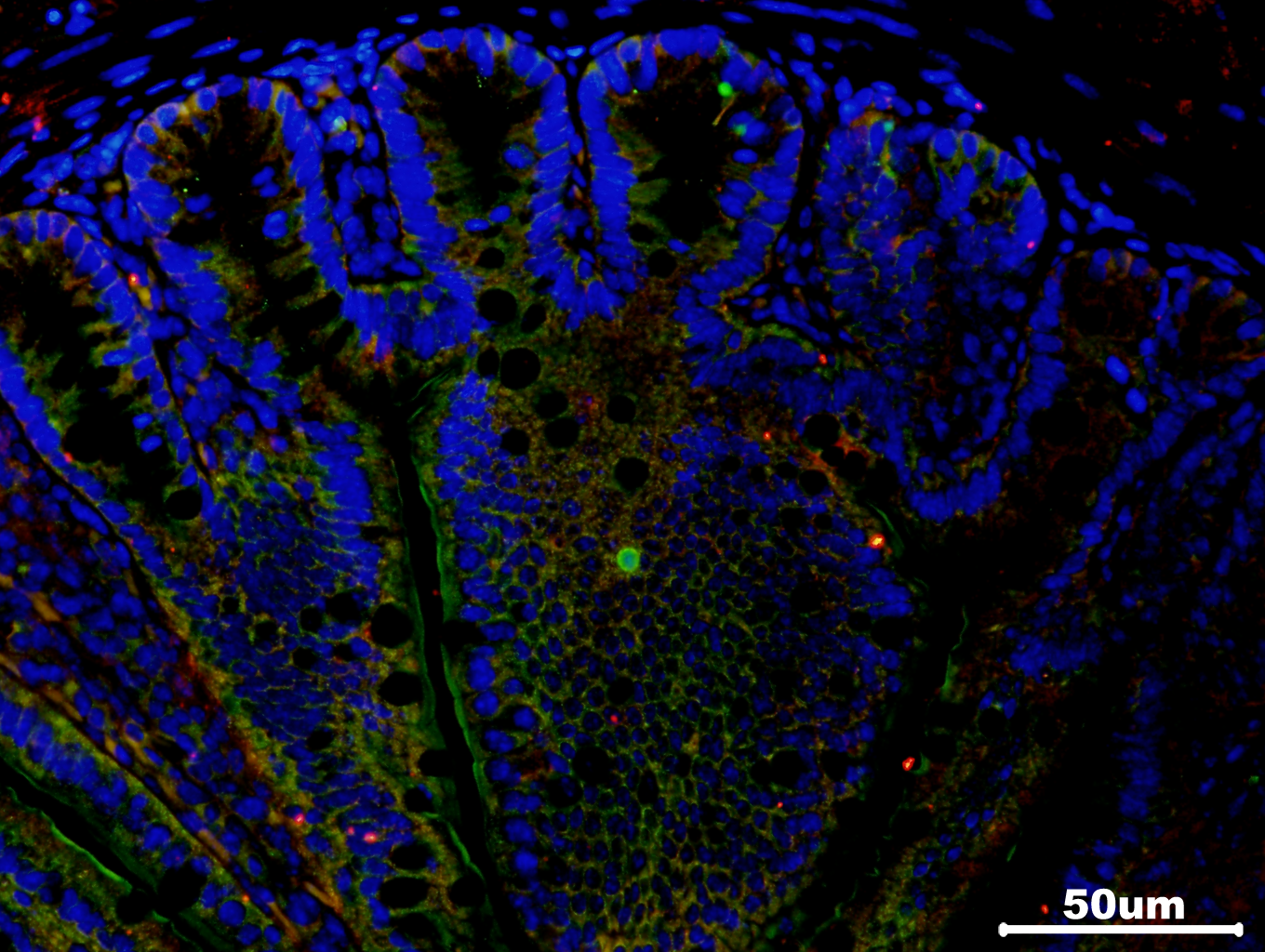

Supplement: Supplementary file 6 [file DataSheet_6.zip › C26-1-200-3-merge.tif]

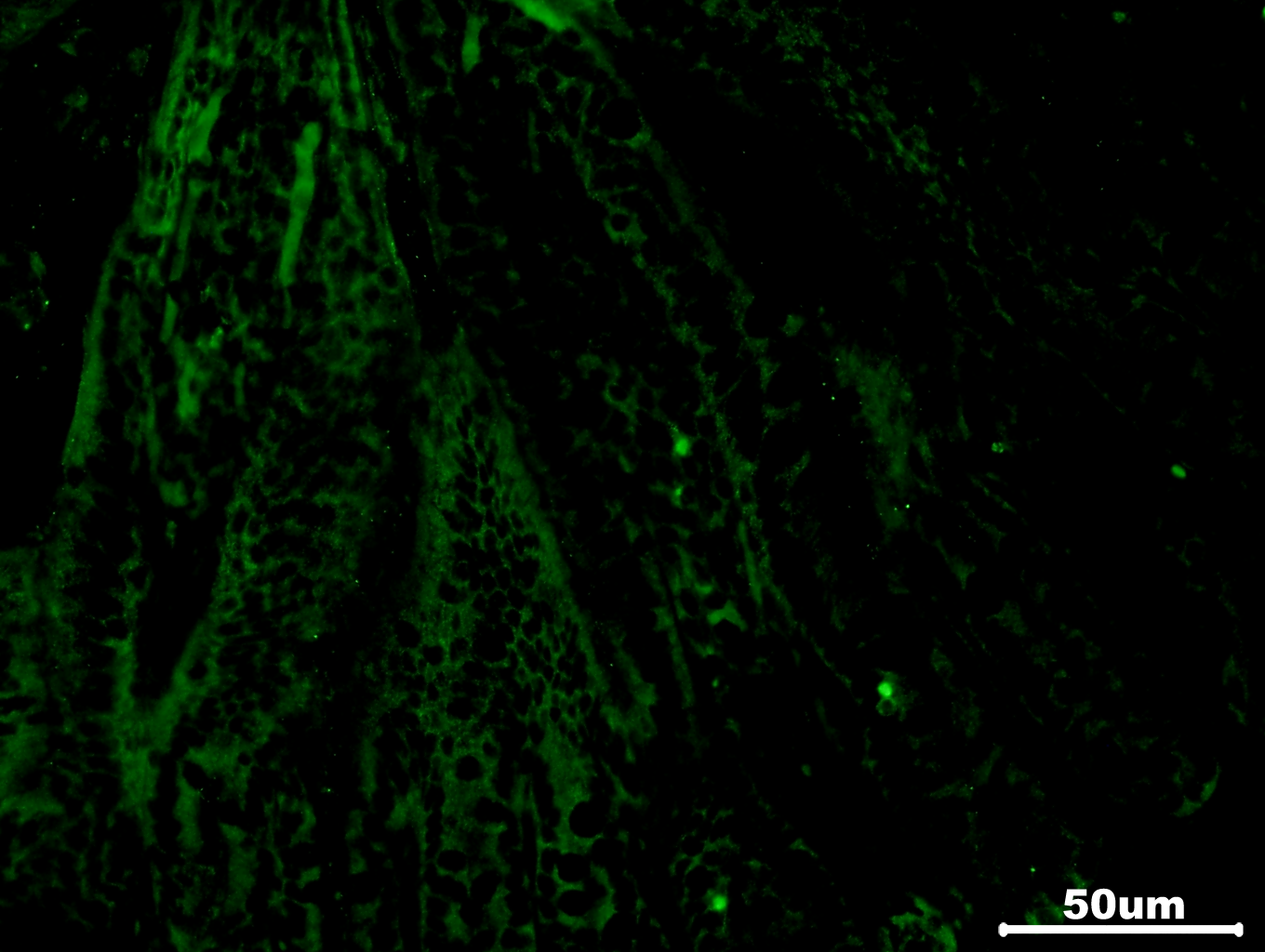

Supplement: Supplementary file 6 [file DataSheet_6.zip › C26-2-200-1-CD86.tif]

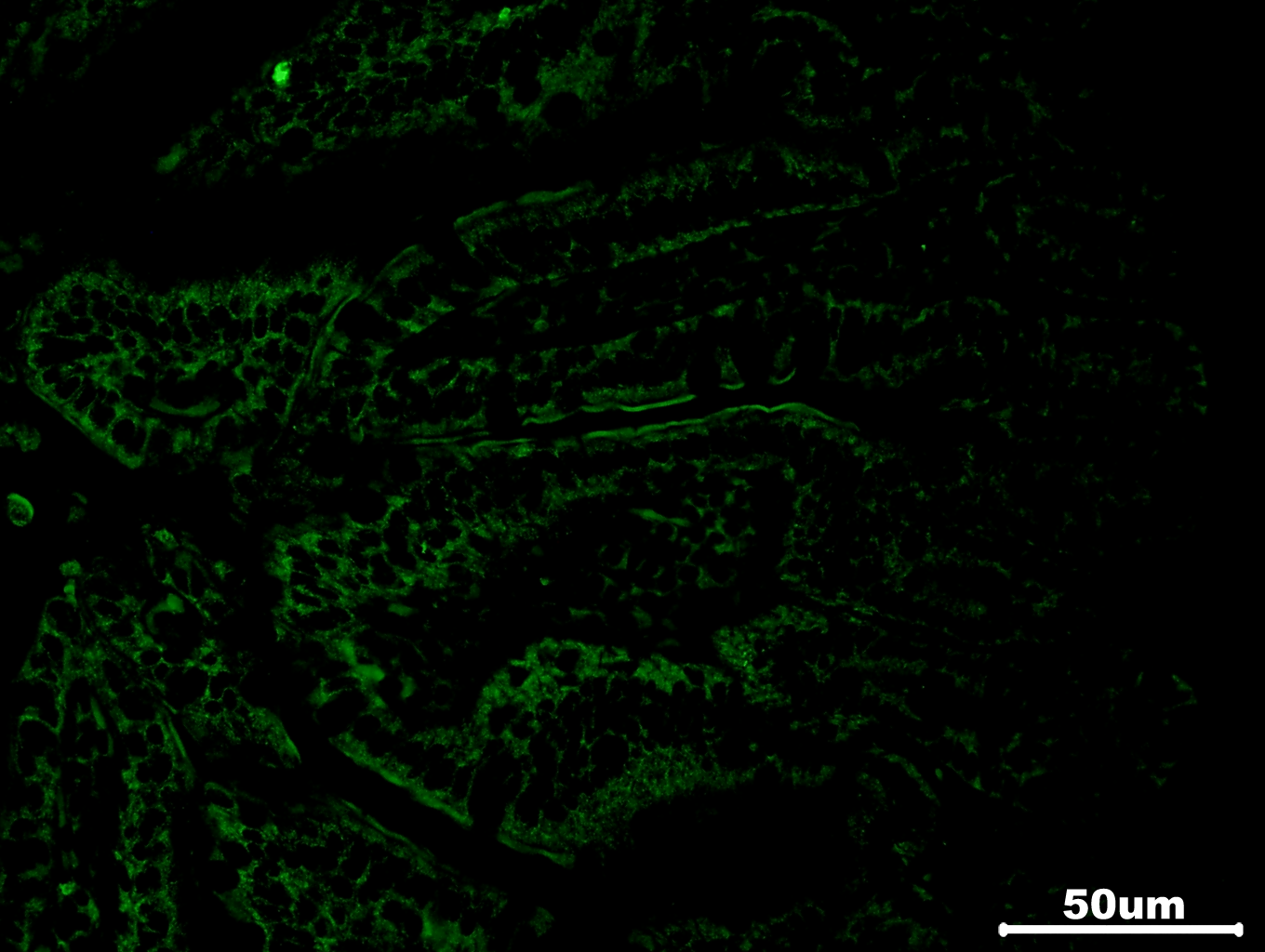

Supplement: Supplementary file 8 [file DataSheet_8.zip › E31-2-200-3-CD86.tif]

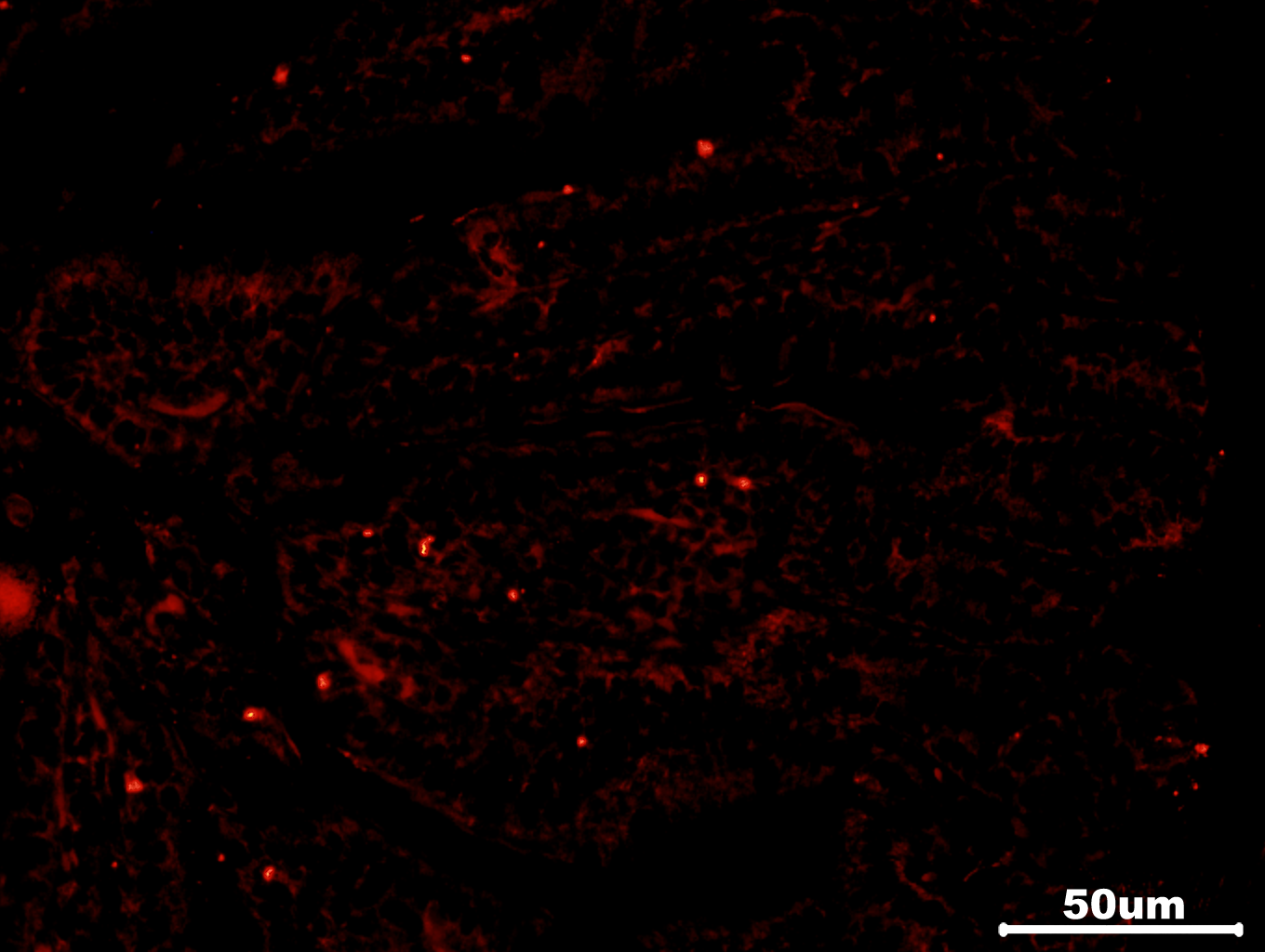

Supplement: Supplementary file 8 [file DataSheet_8.zip › E31-2-200-3-CD206.tif]

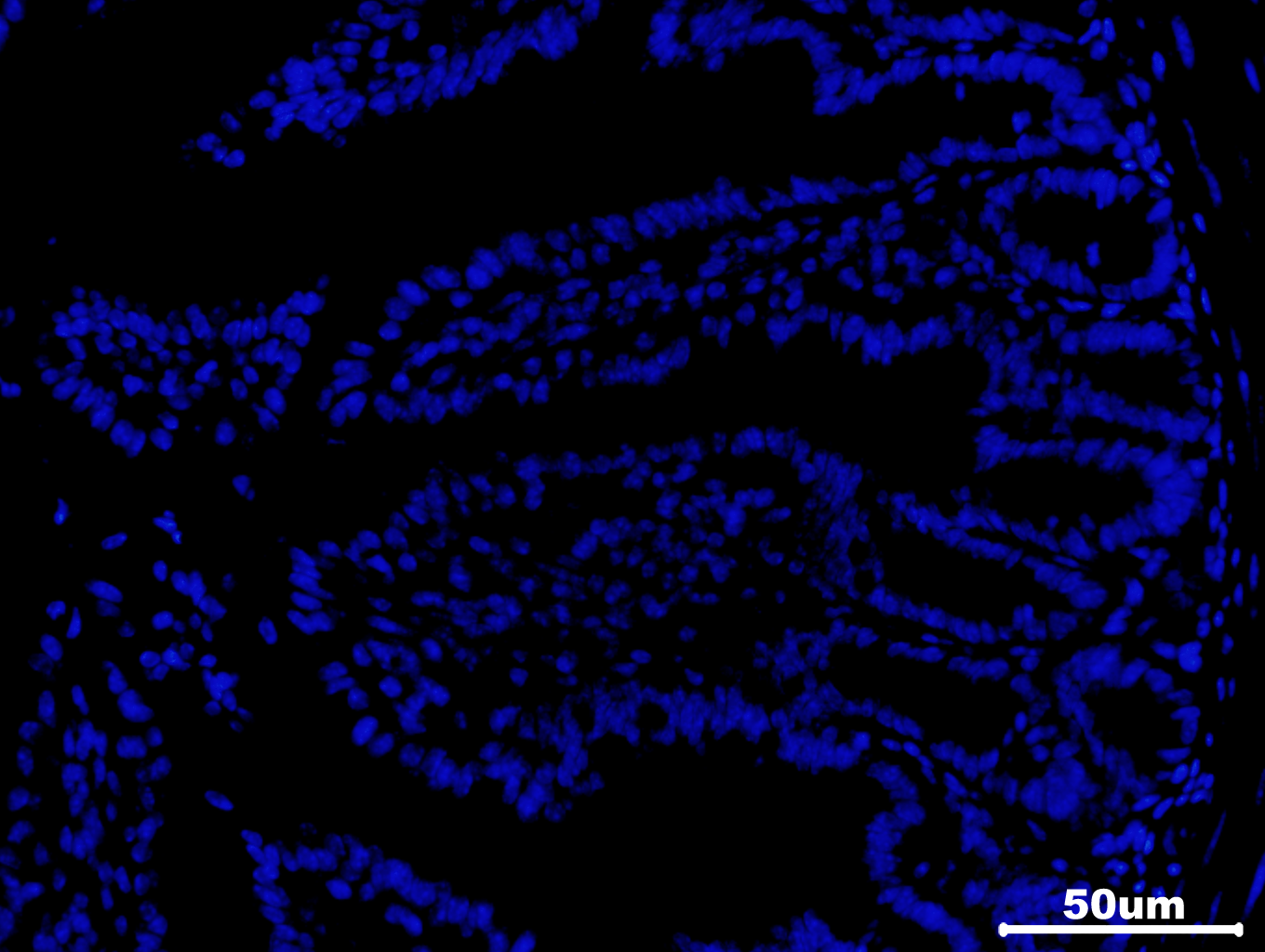

Supplement: Supplementary file 8 [file DataSheet_8.zip › E31-2-200-3-DAPI.tif]

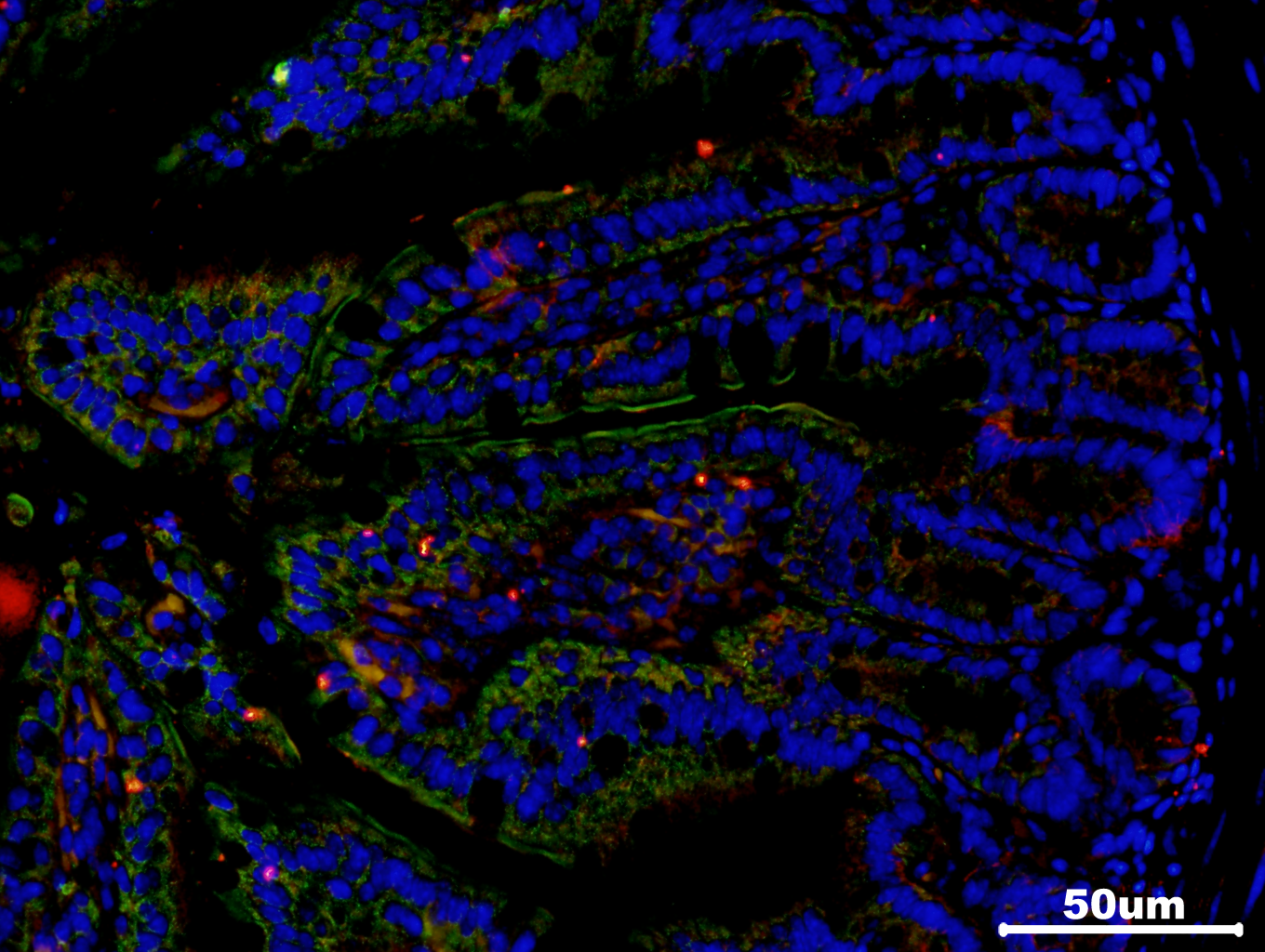

Supplement: Supplementary file 8 [file DataSheet_8.zip › E31-2-200-3-merge.tif]

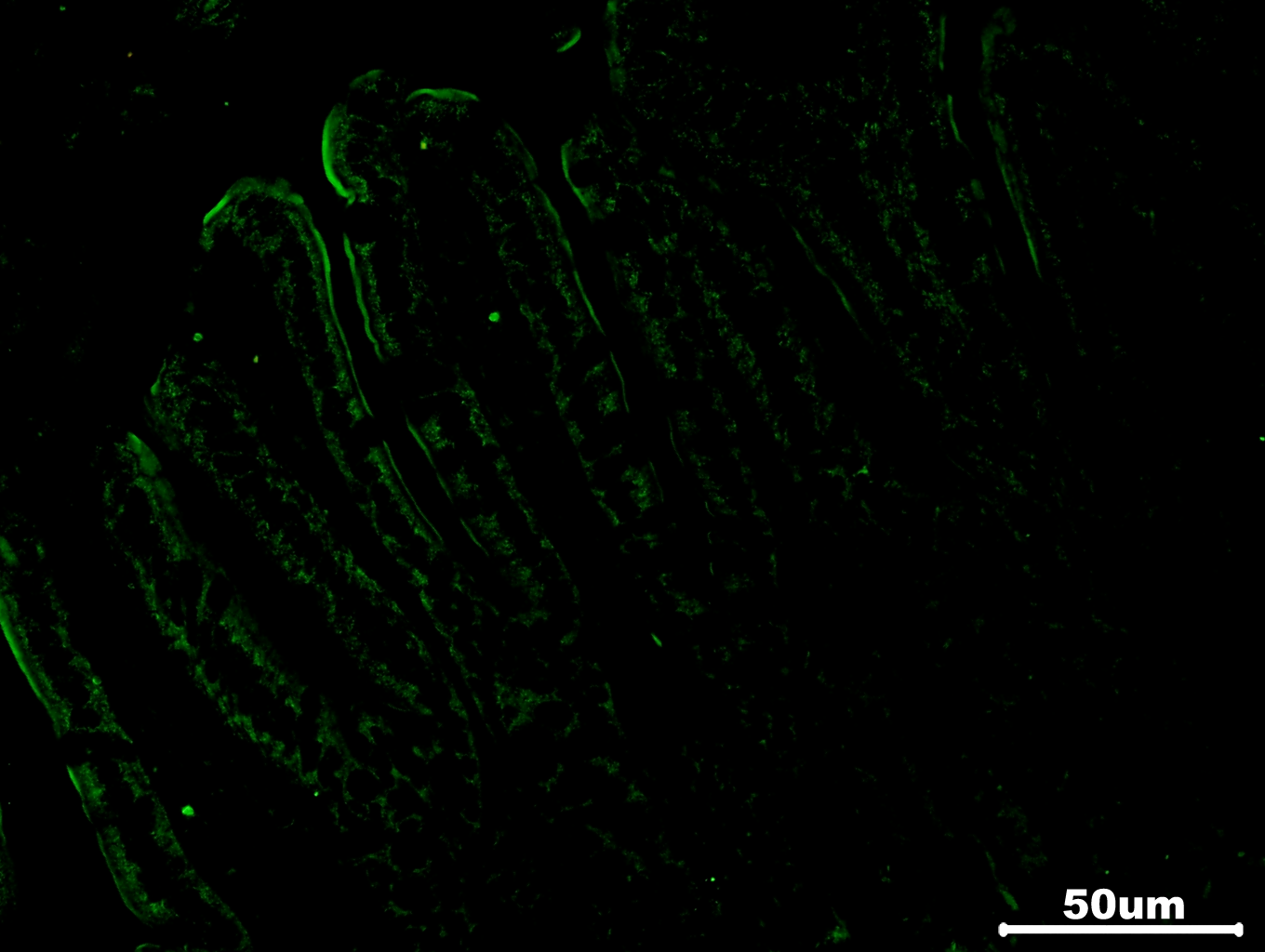

Supplement: Supplementary file 8 [file DataSheet_8.zip › E32-1-200-1-CD86.tif]

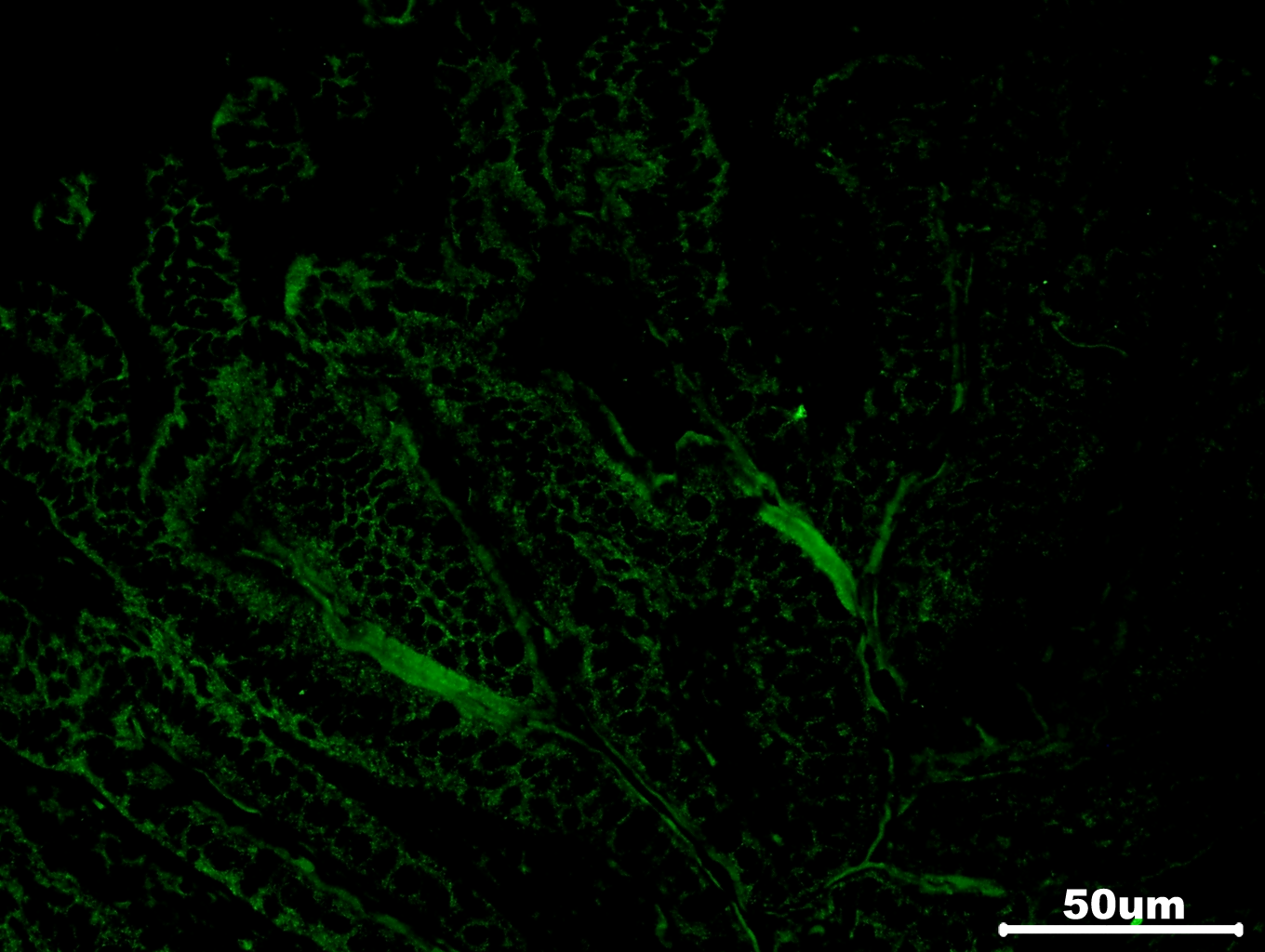

Supplement: Supplementary file 9 [file DataSheet_9.zip › D28-1-200-1-CD86.tif]

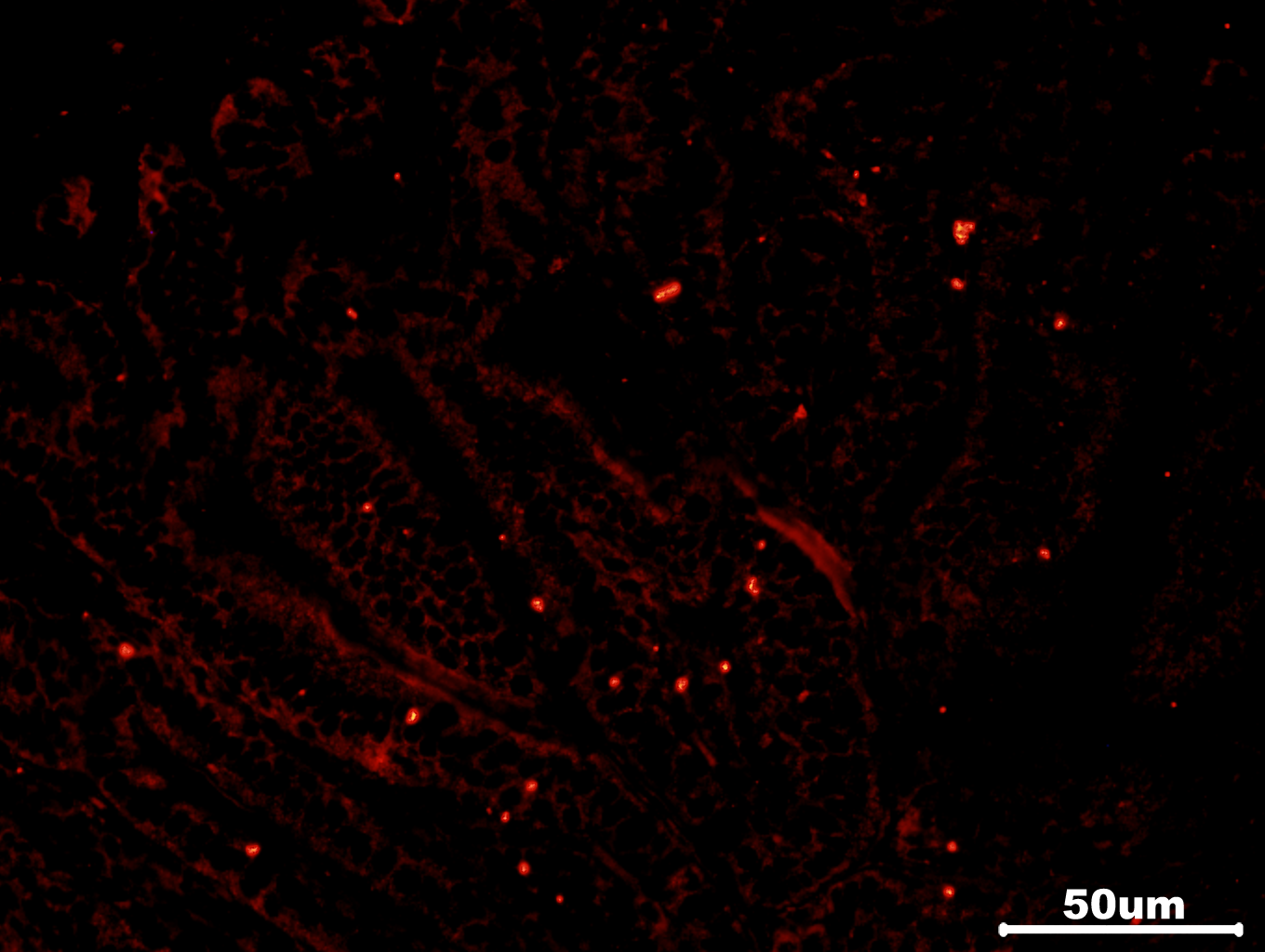

Supplement: Supplementary file 9 [file DataSheet_9.zip › D28-1-200-1-CD206.tif]

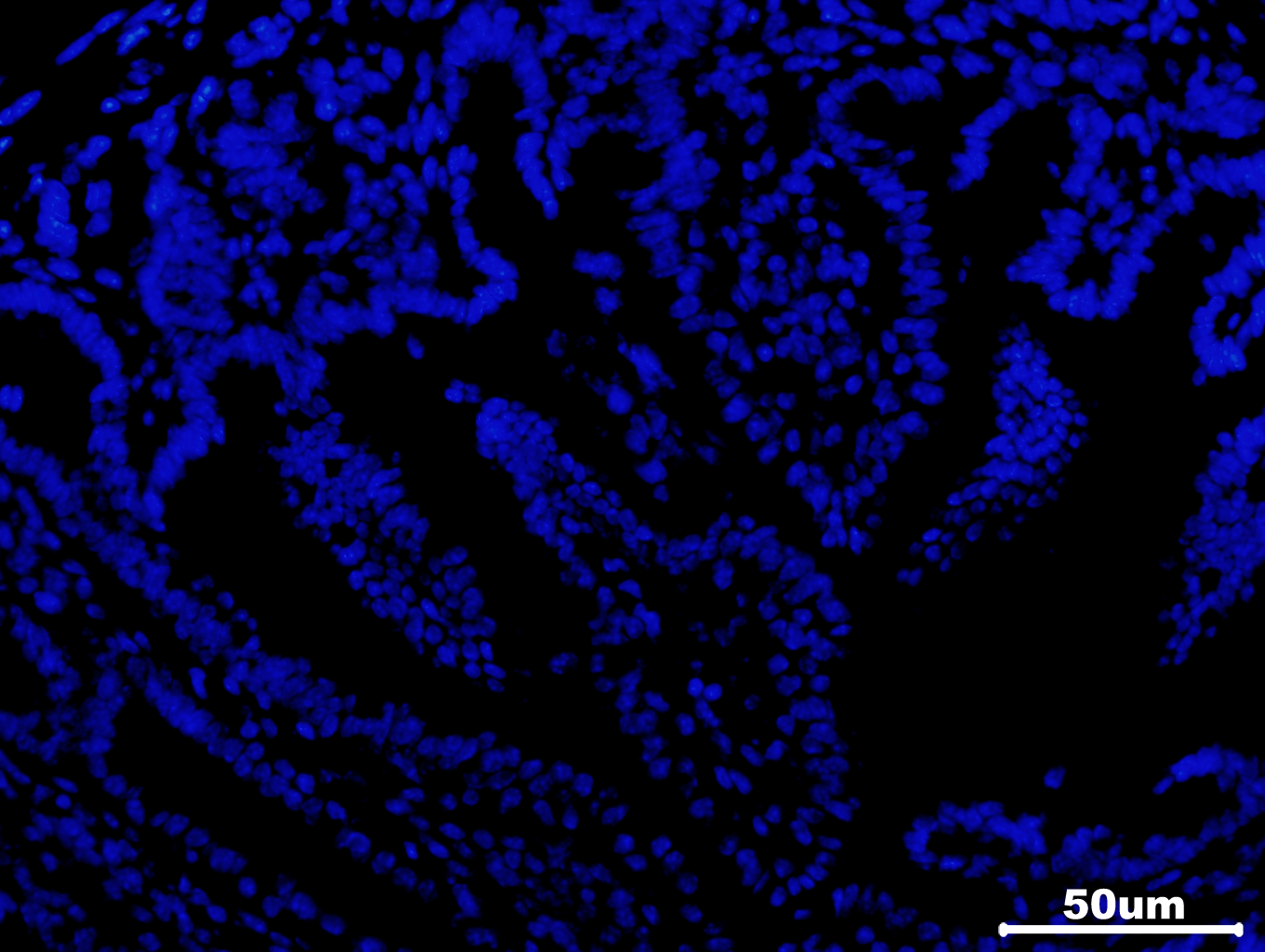

Supplement: Supplementary file 9 [file DataSheet_9.zip › D28-1-200-1-DAPI.tif]

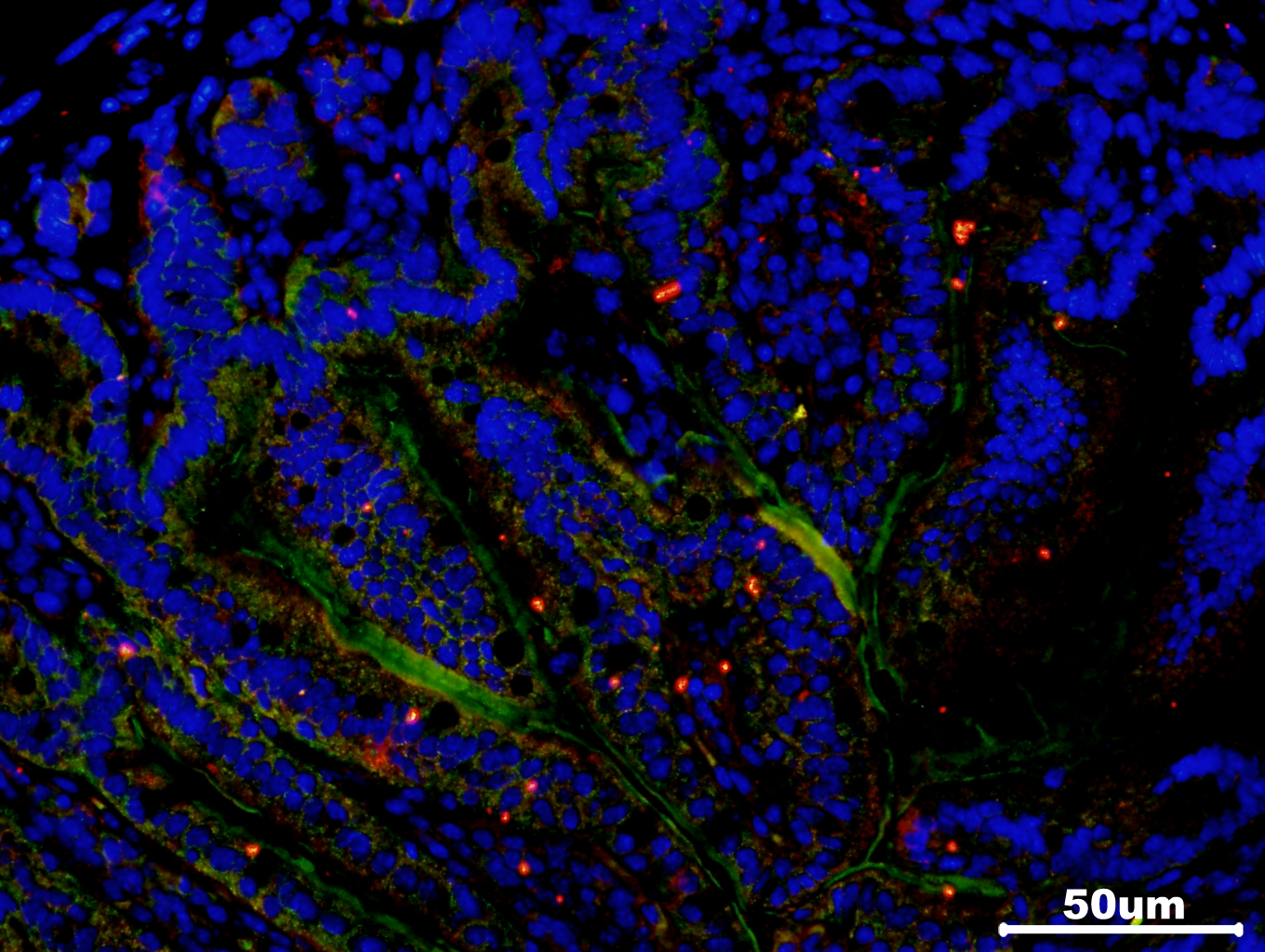

Supplement: Supplementary file 9 [file DataSheet_9.zip › D28-1-200-1-merge.tif]

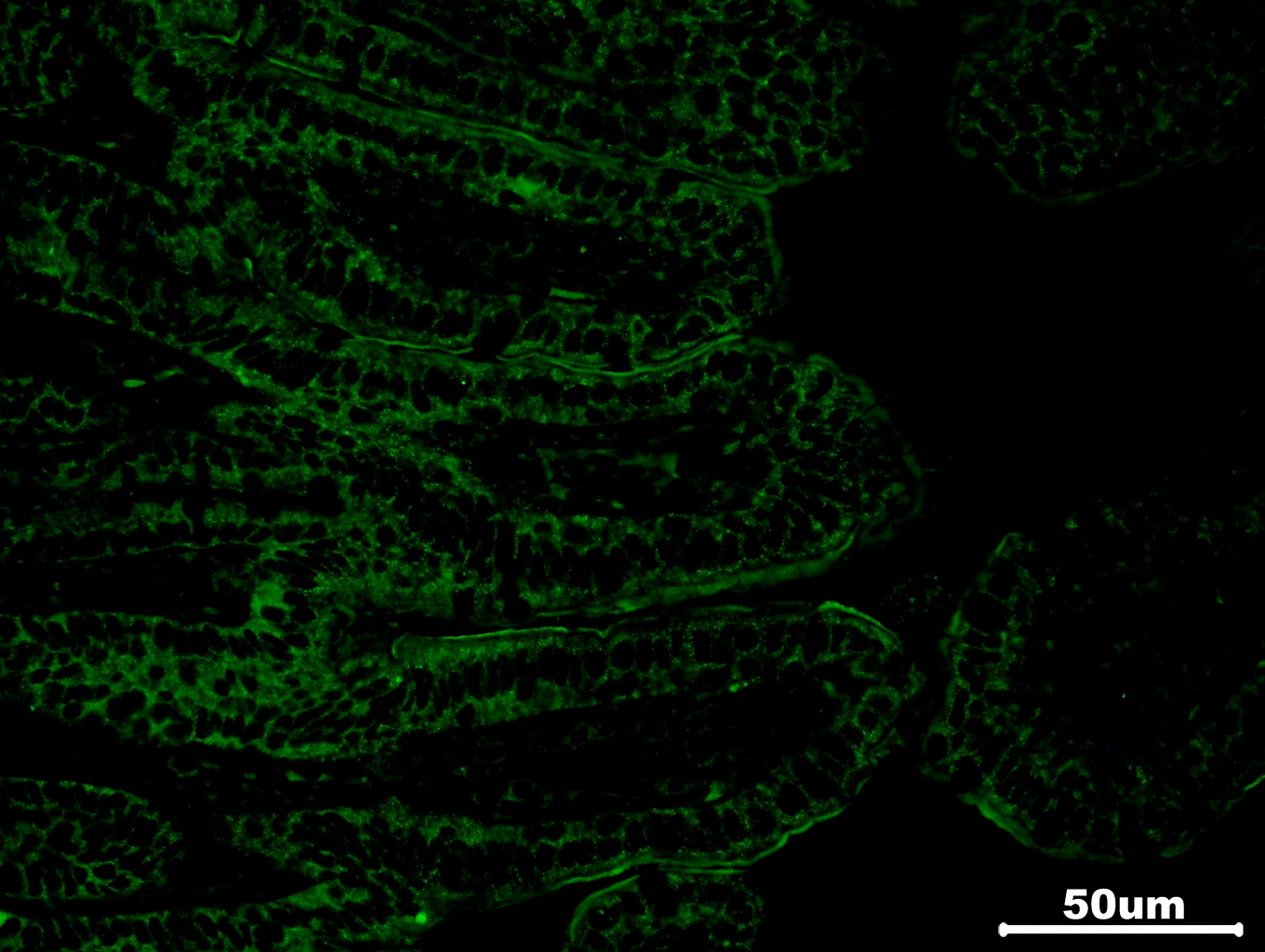

Supplement: Supplementary file 9 [file DataSheet_9.zip › D28-1-200-2-CD86.tif]

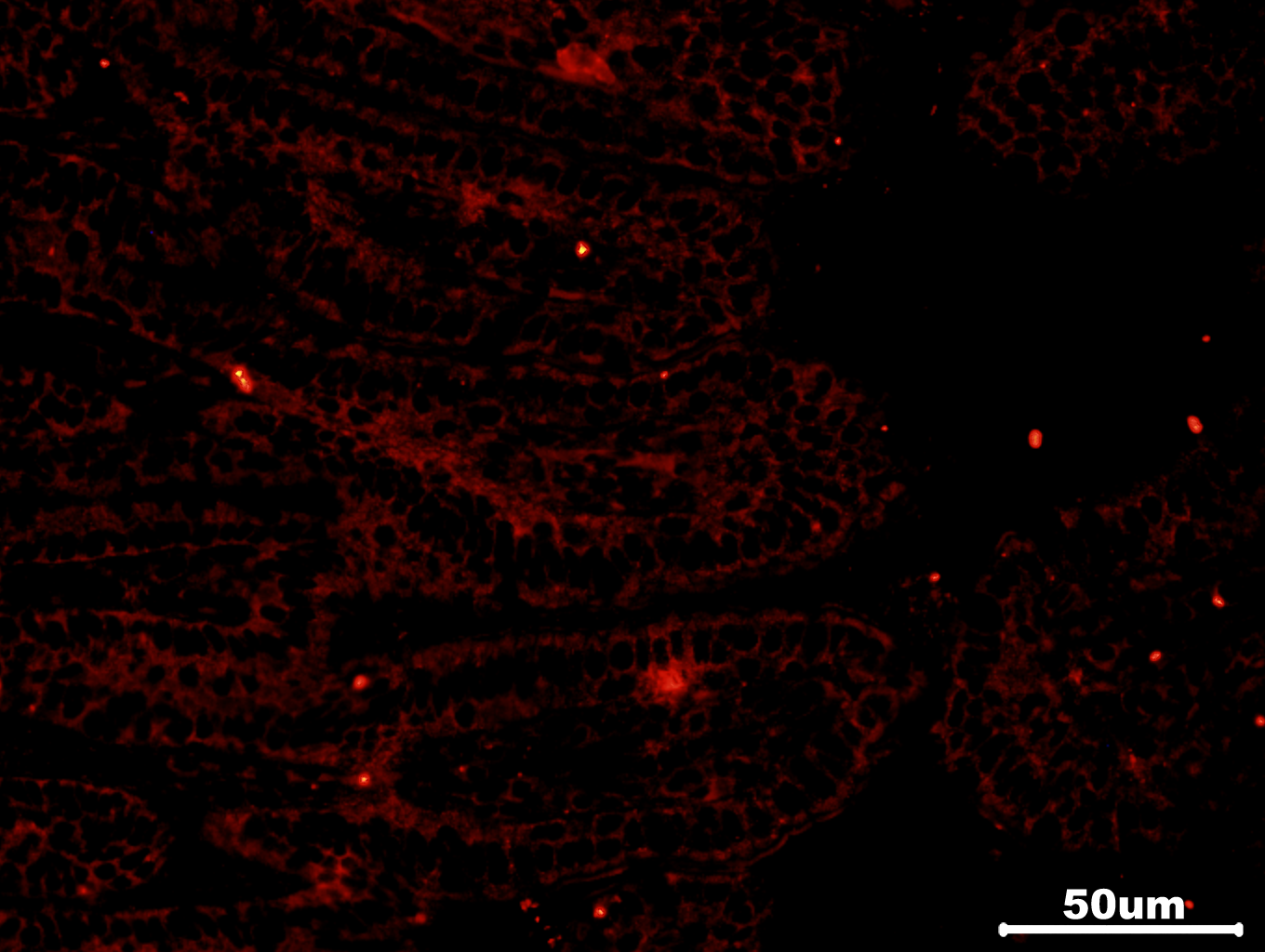

Supplement: Supplementary file 9 [file DataSheet_9.zip › D28-1-200-2-CD206.tif]

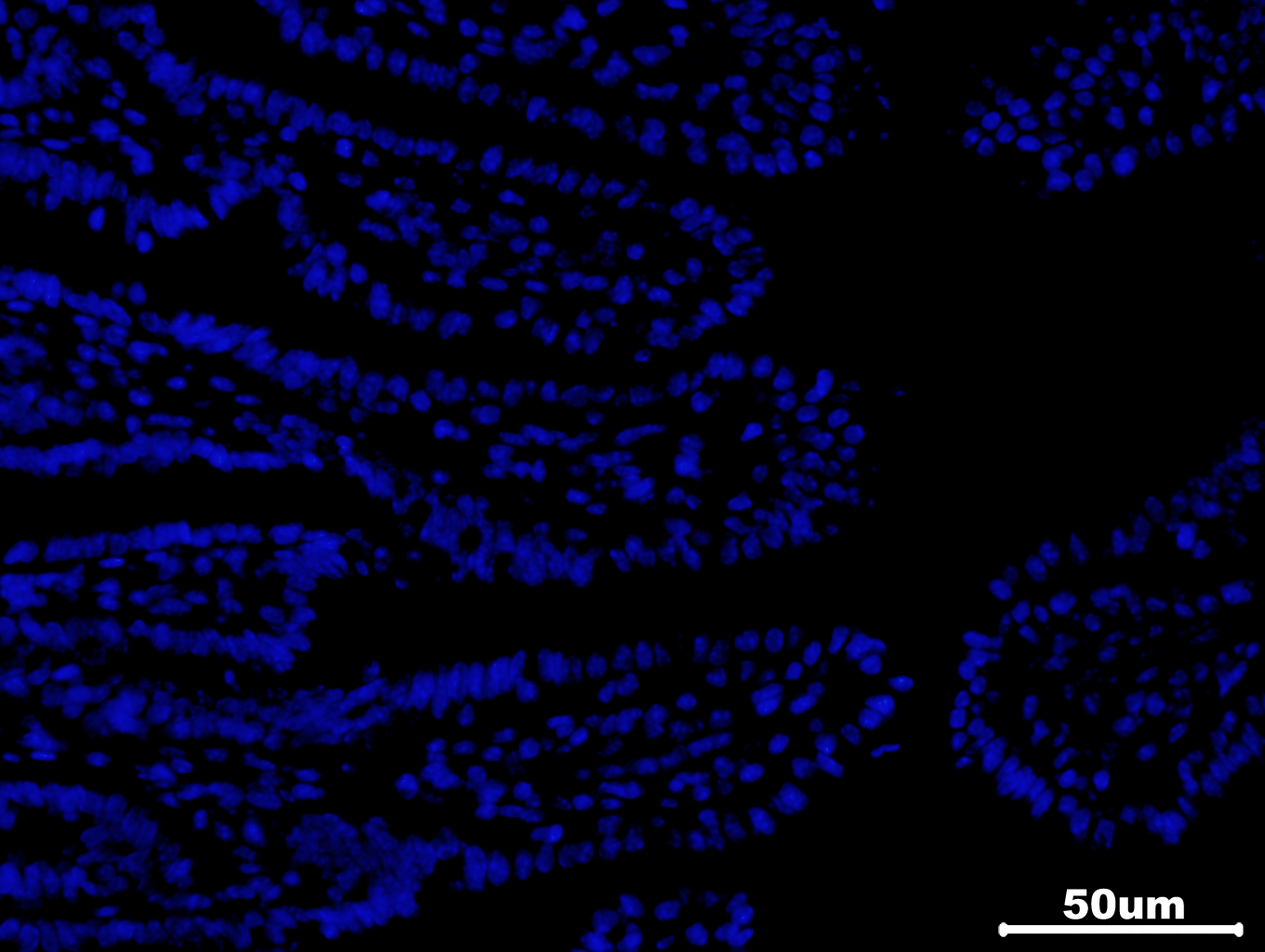

Supplement: Supplementary file 9 [file DataSheet_9.zip › D28-1-200-2-DAPI.tif]

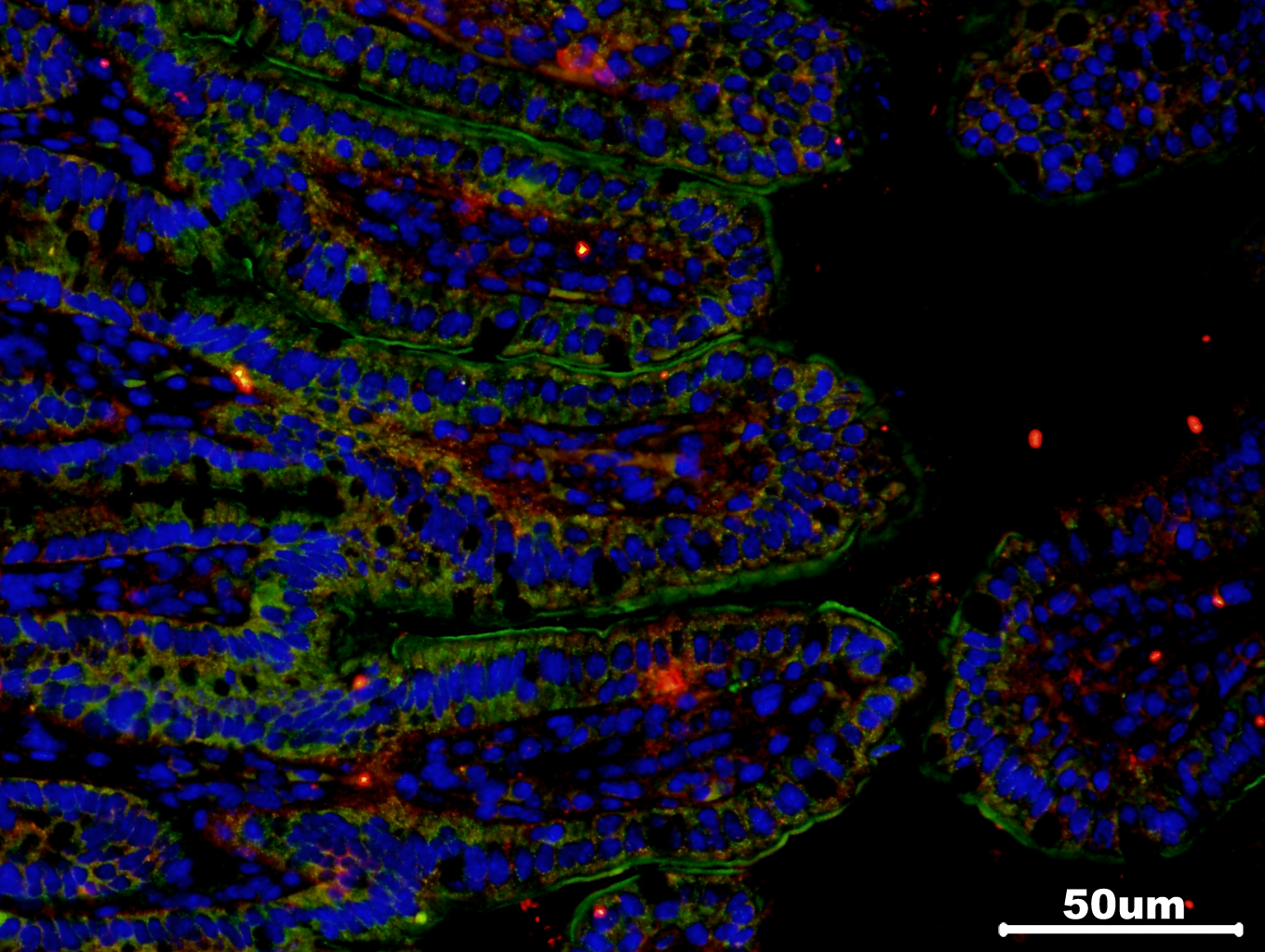

Supplement: Supplementary file 9 [file DataSheet_9.zip › D28-1-200-2-merge.tif]

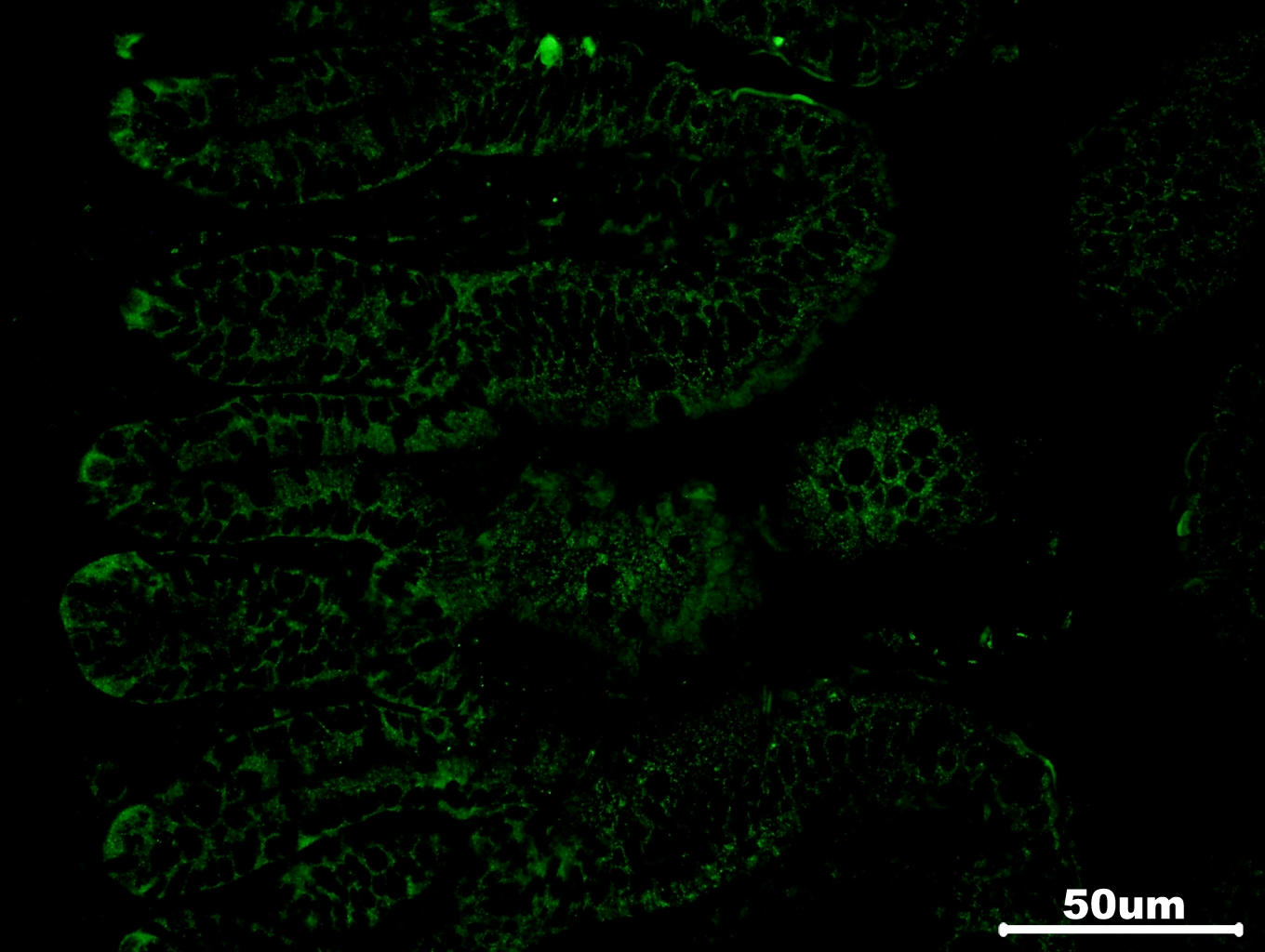

Supplement: Supplementary file 9 [file DataSheet_9.zip › D28-1-200-3-CD86.tif]

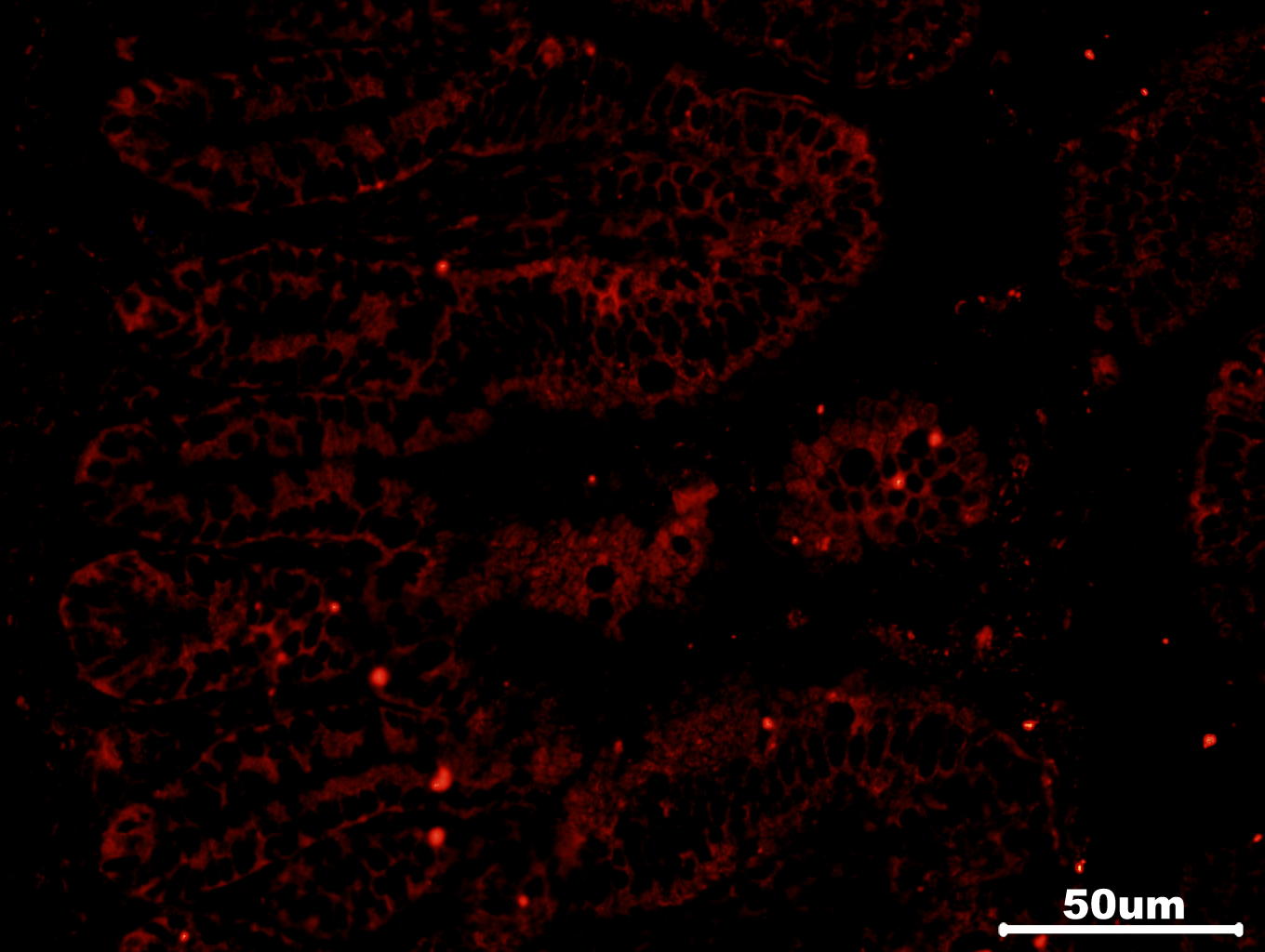

Supplement: Supplementary file 9 [file DataSheet_9.zip › D28-1-200-3-CD206.tif]

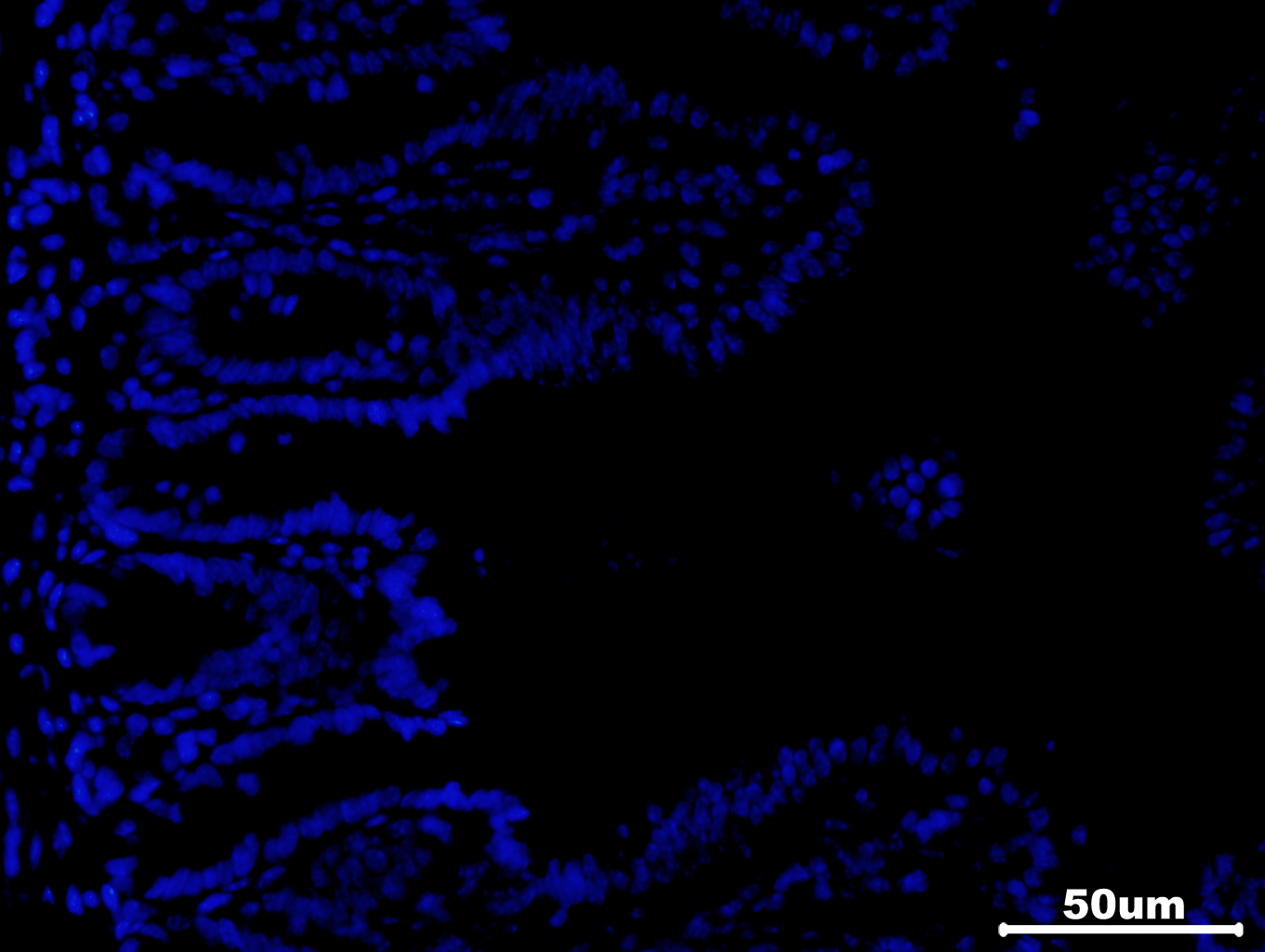

Supplement: Supplementary file 9 [file DataSheet_9.zip › D28-1-200-3-DAPI.tif]

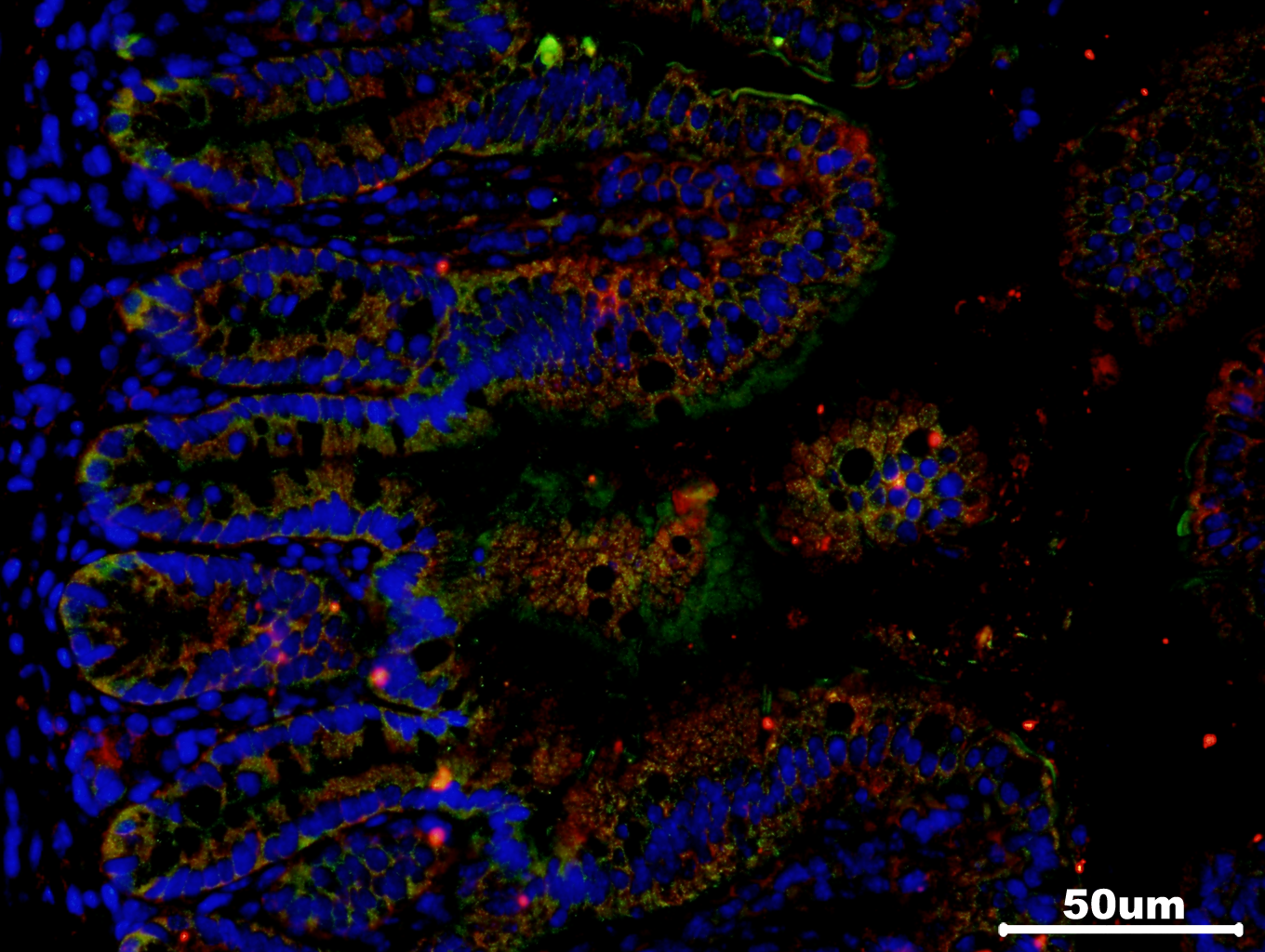

Supplement: Supplementary file 9 [file DataSheet_9.zip › D28-1-200-3-merge.tif]

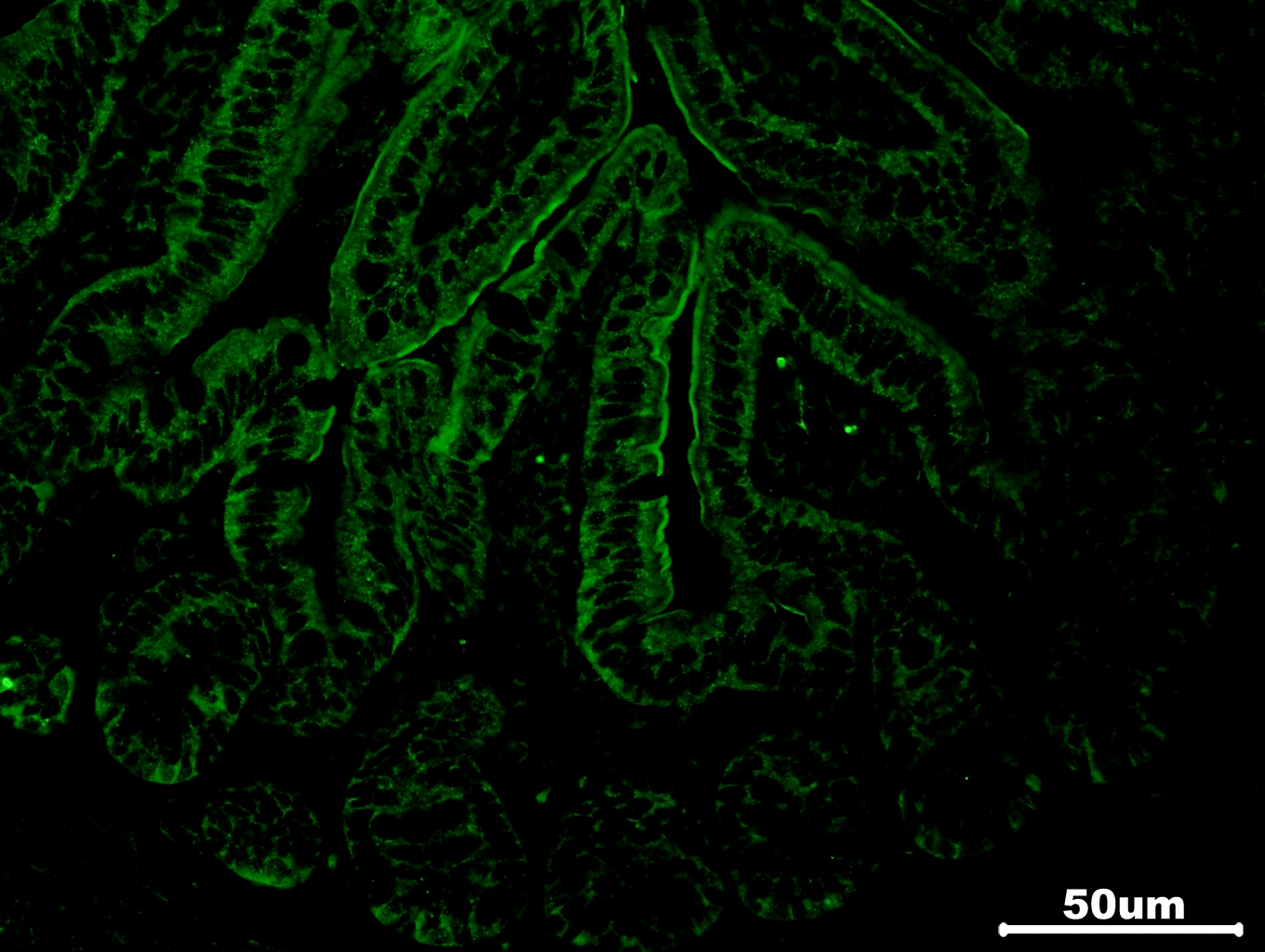

Supplement: Supplementary file 9 [file DataSheet_9.zip › D28-2-200-1-CD86.tif]

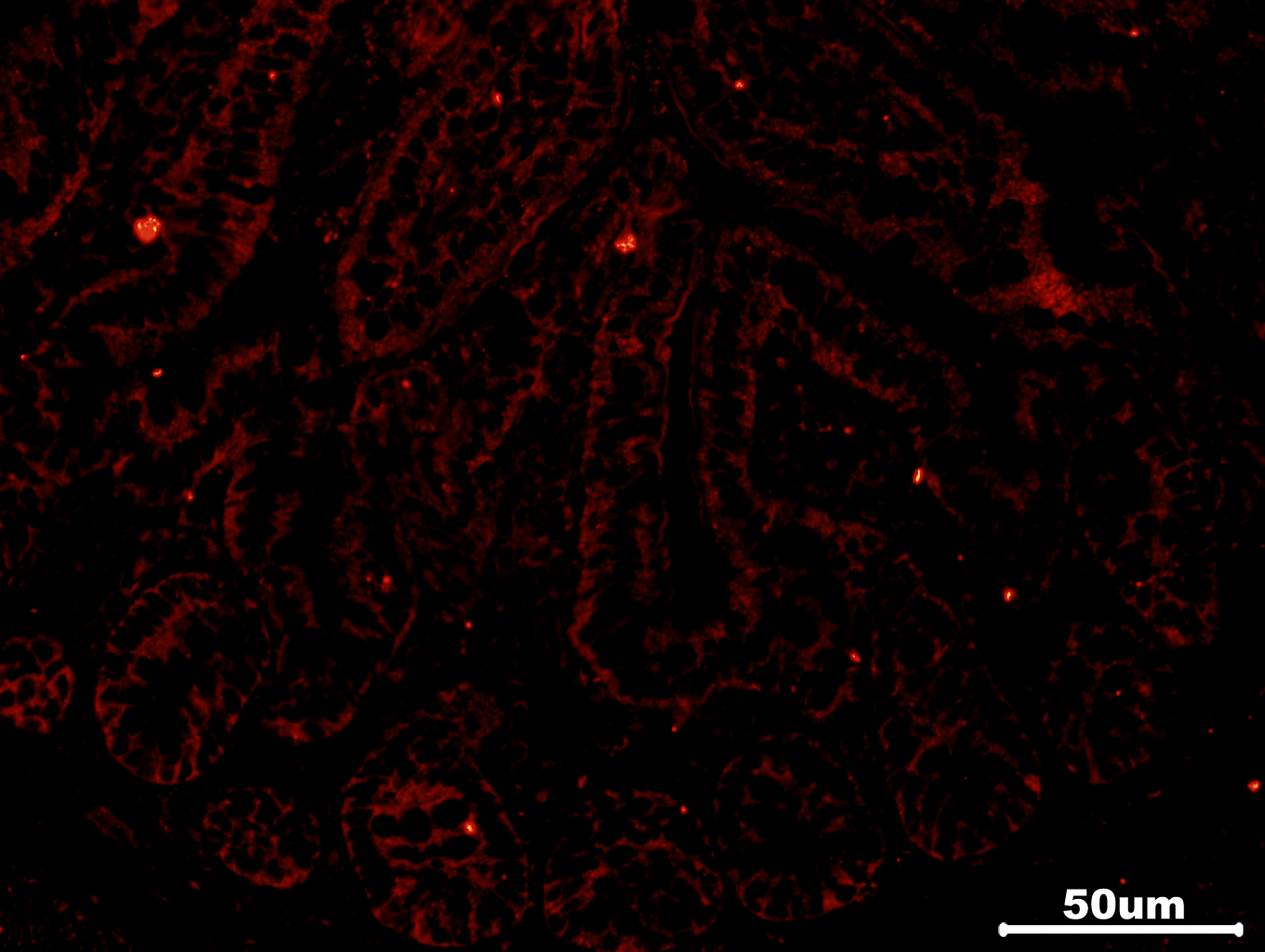

Supplement: Supplementary file 9 [file DataSheet_9.zip › D28-2-200-1-CD206.tif]

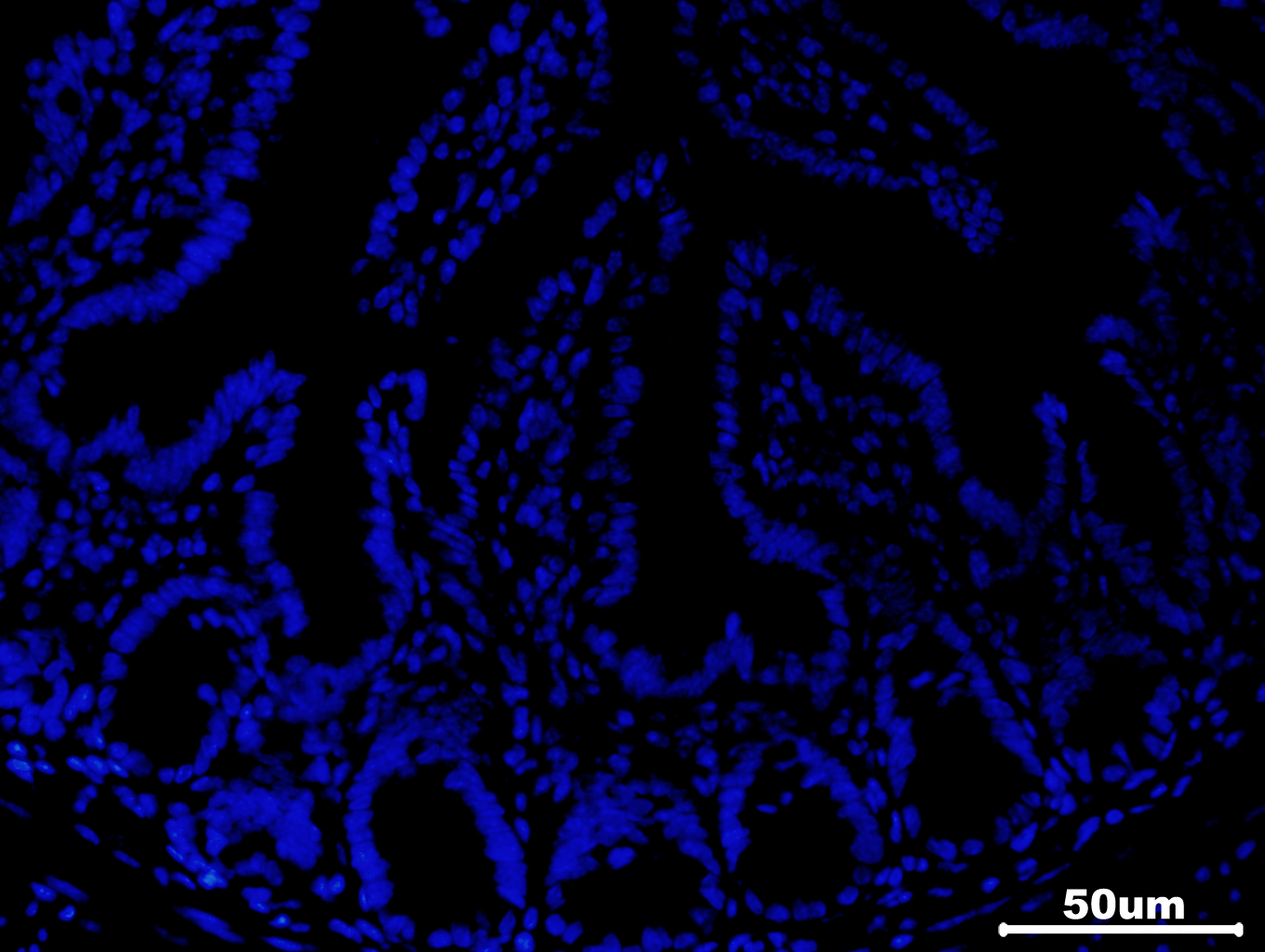

Supplement: Supplementary file 9 [file DataSheet_9.zip › D28-2-200-1-DAPI.tif]

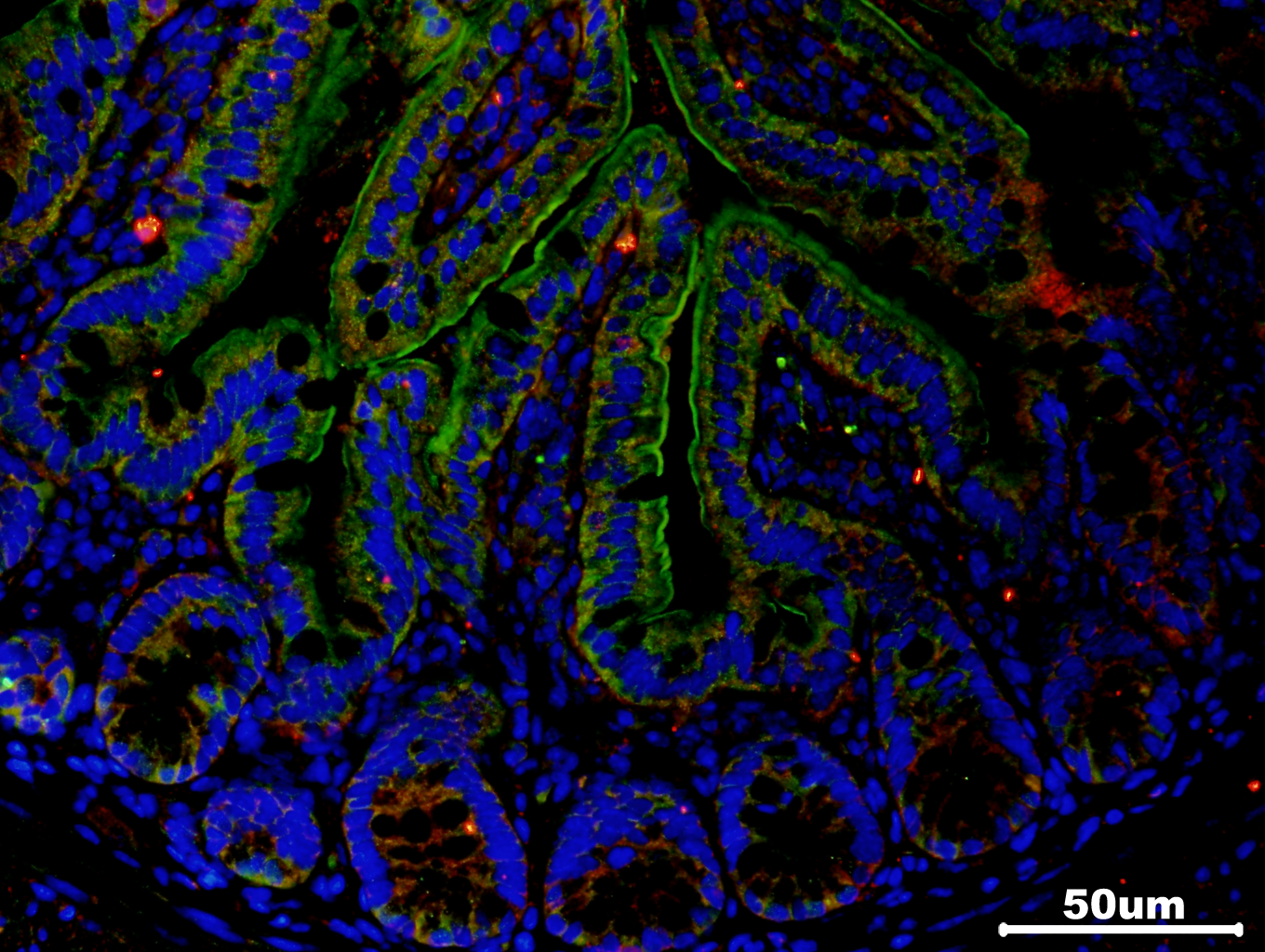

Supplement: Supplementary file 9 [file DataSheet_9.zip › D28-2-200-1-merge.tif]

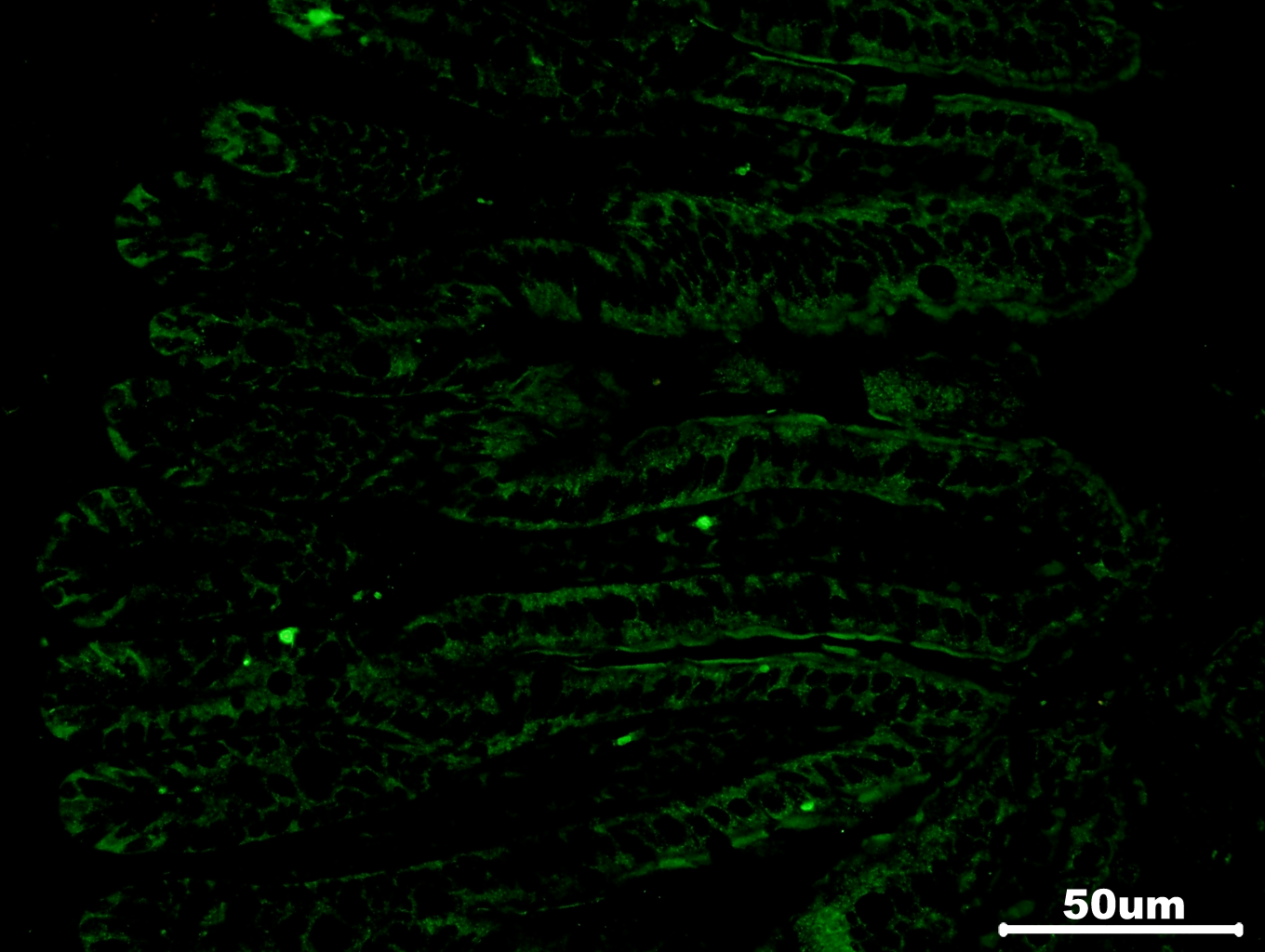

Supplement: Supplementary file 9 [file DataSheet_9.zip › D28-2-200-2-CD86.tif]

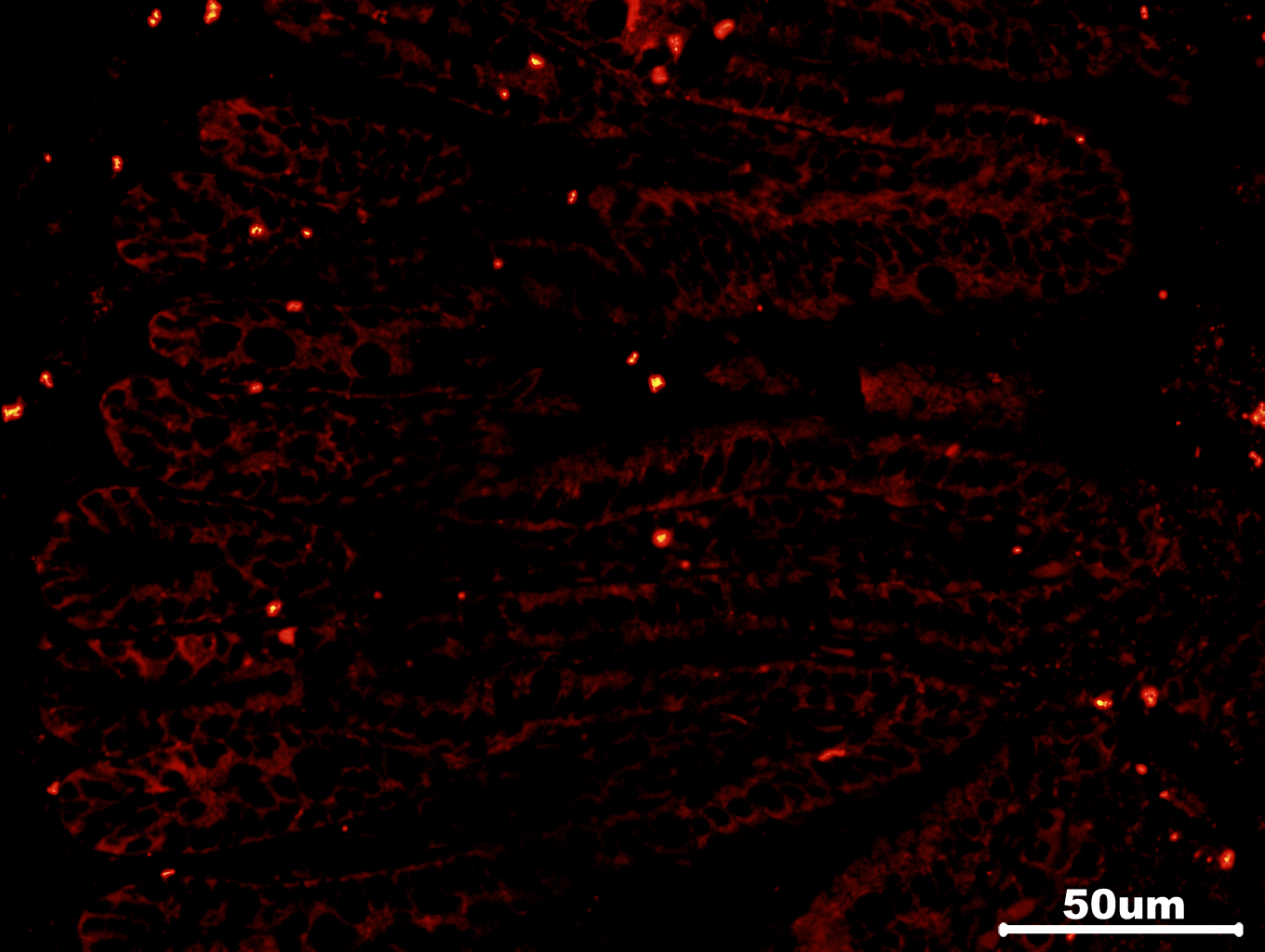

Supplement: Supplementary file 9 [file DataSheet_9.zip › D28-2-200-2-CD206.tif]

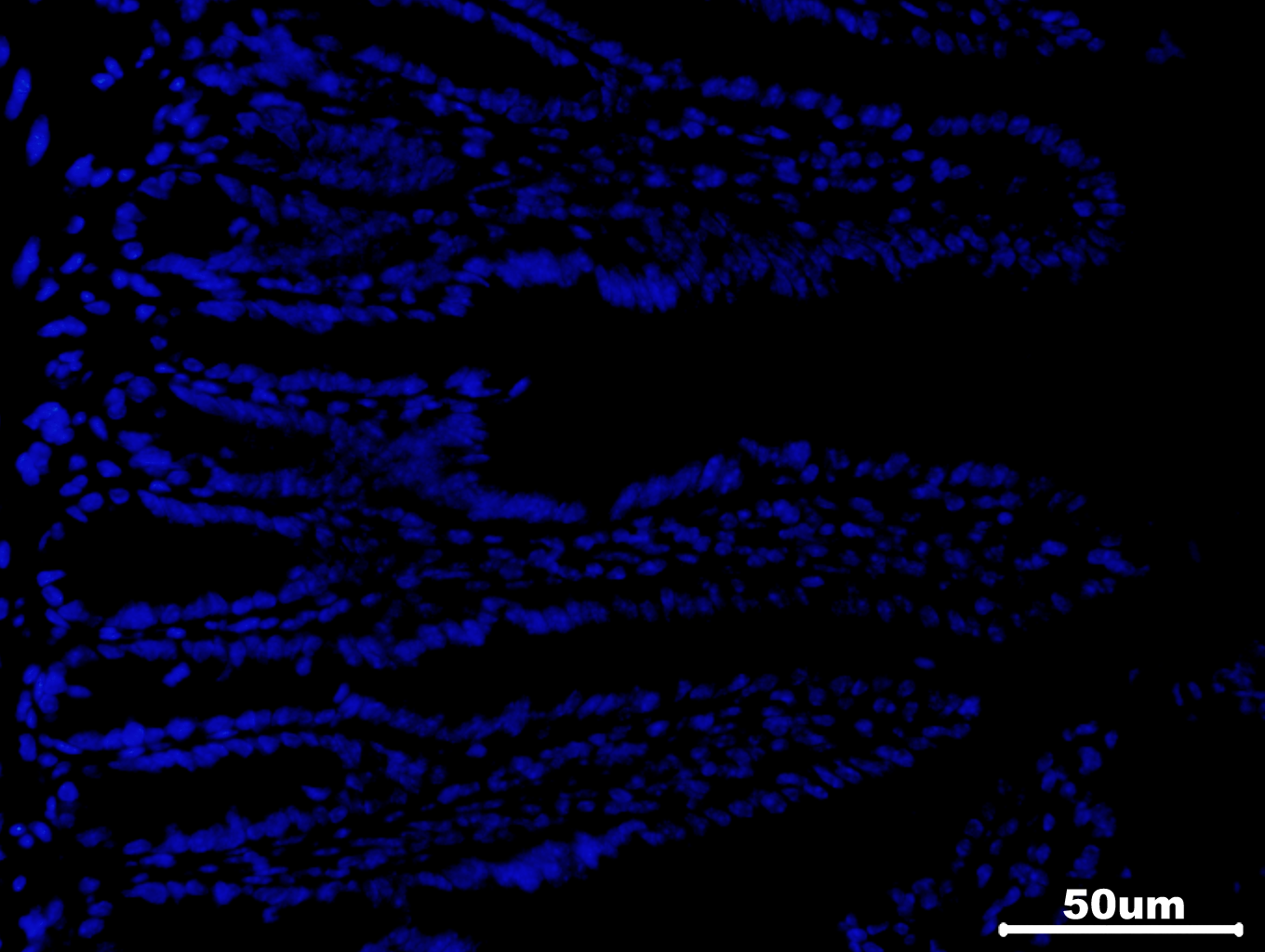

Supplement: Supplementary file 9 [file DataSheet_9.zip › D28-2-200-2-DAPI.tif]

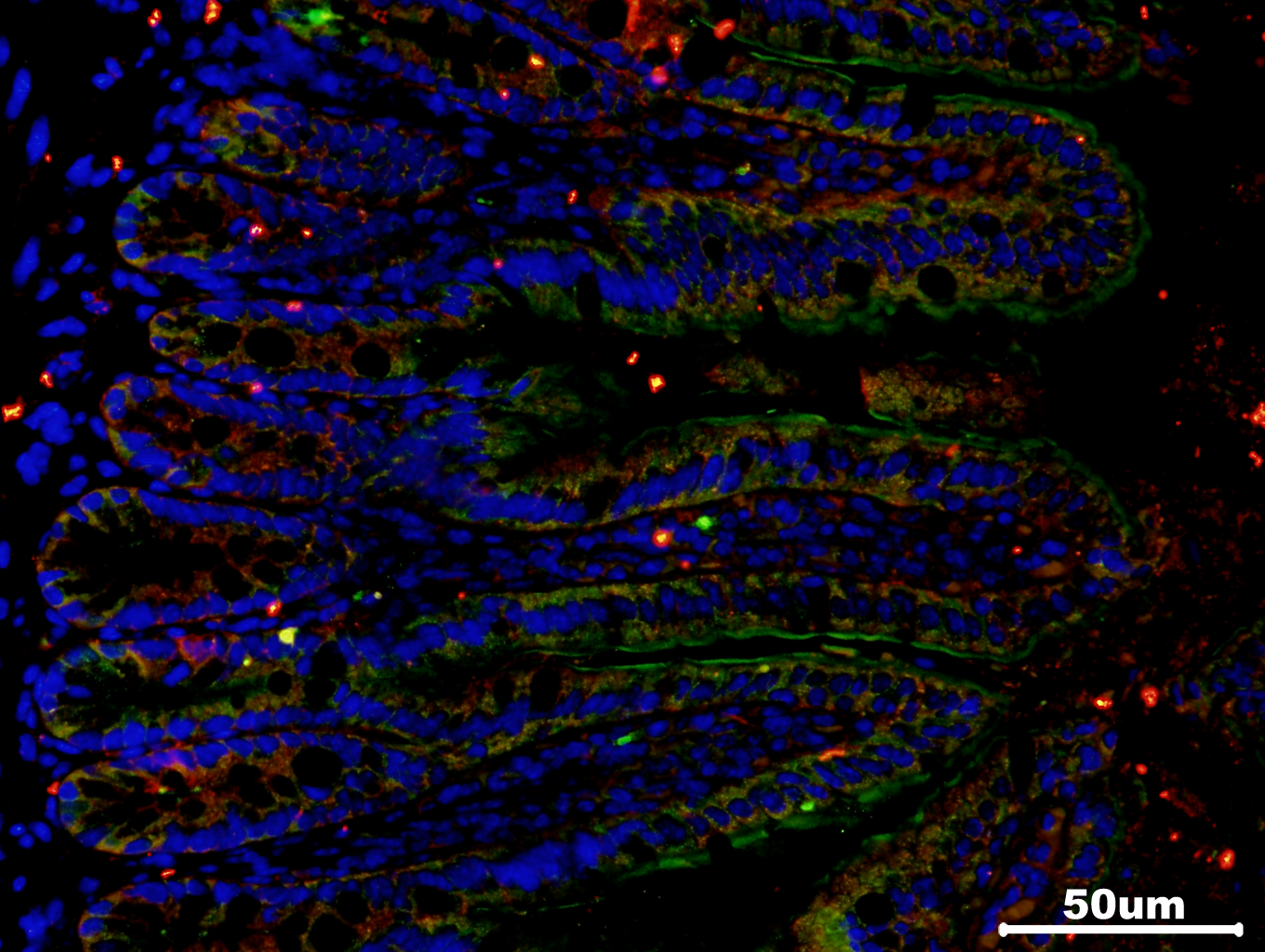

Supplement: Supplementary file 9 [file DataSheet_9.zip › D28-2-200-2-merge.tif]

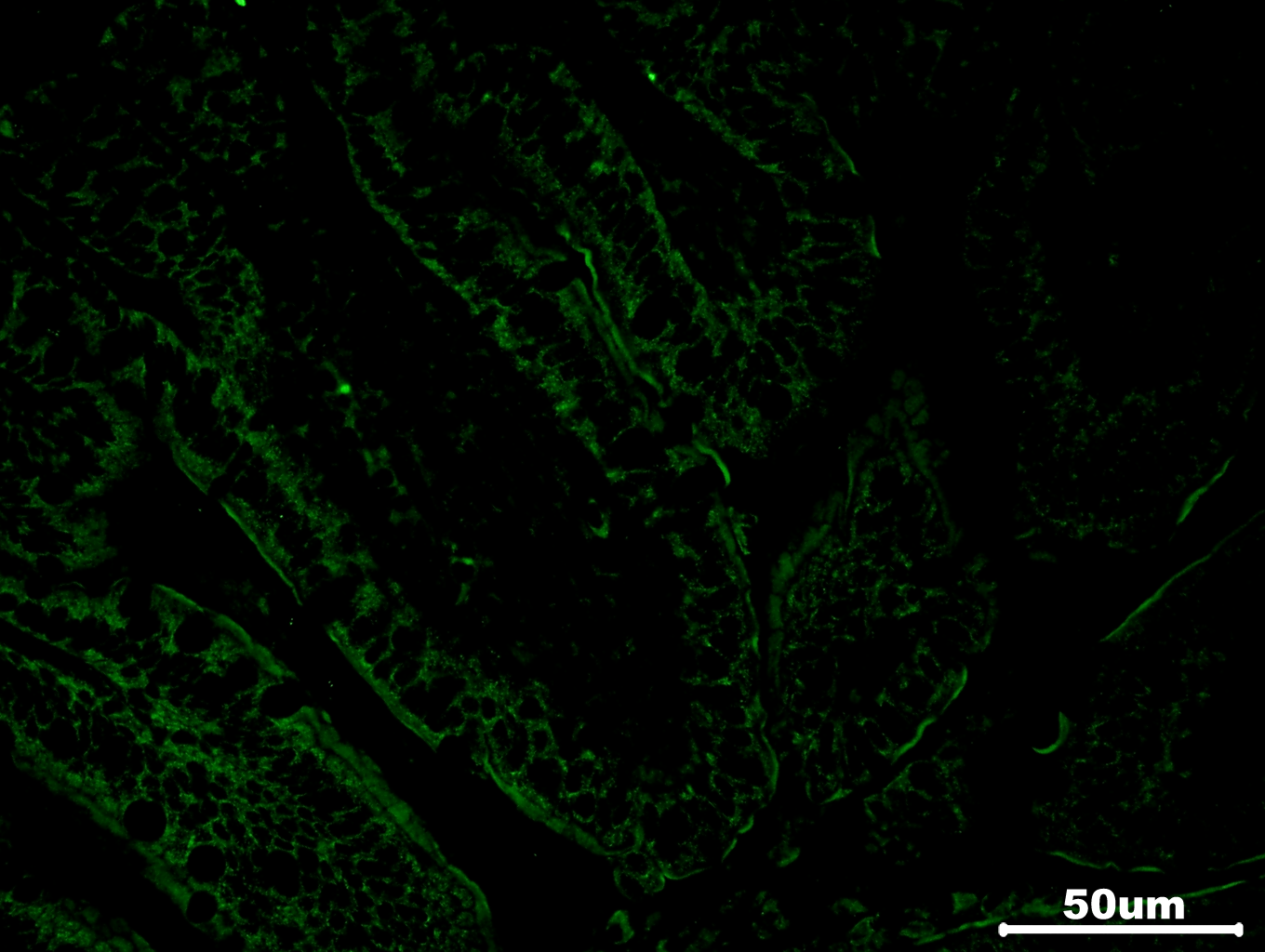

Supplement: Supplementary file 9 [file DataSheet_9.zip › D28-2-200-3-CD86.tif]

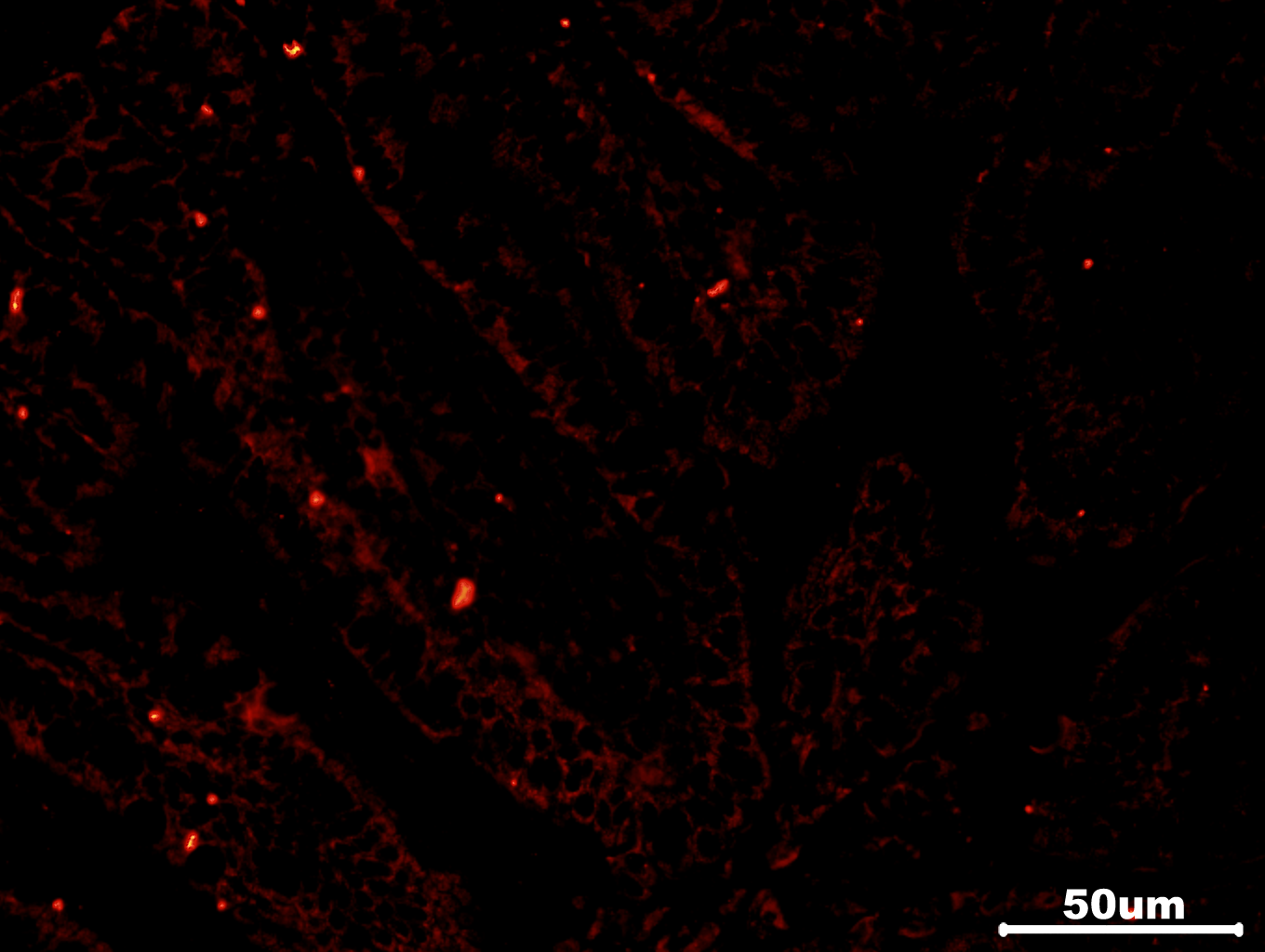

Supplement: Supplementary file 9 [file DataSheet_9.zip › D28-2-200-3-CD206.tif]

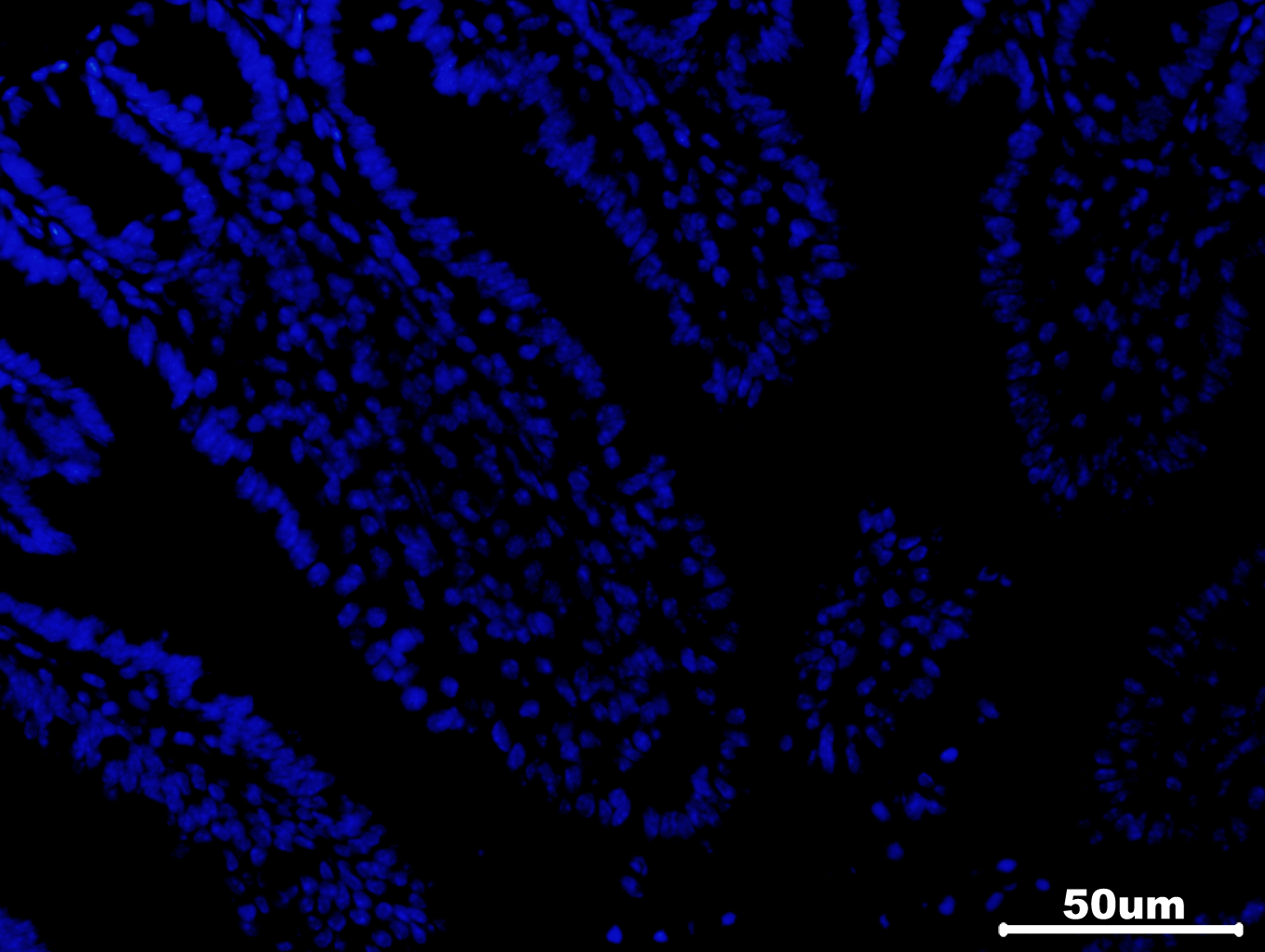

Supplement: Supplementary file 9 [file DataSheet_9.zip › D28-2-200-3-DAPI.tif]

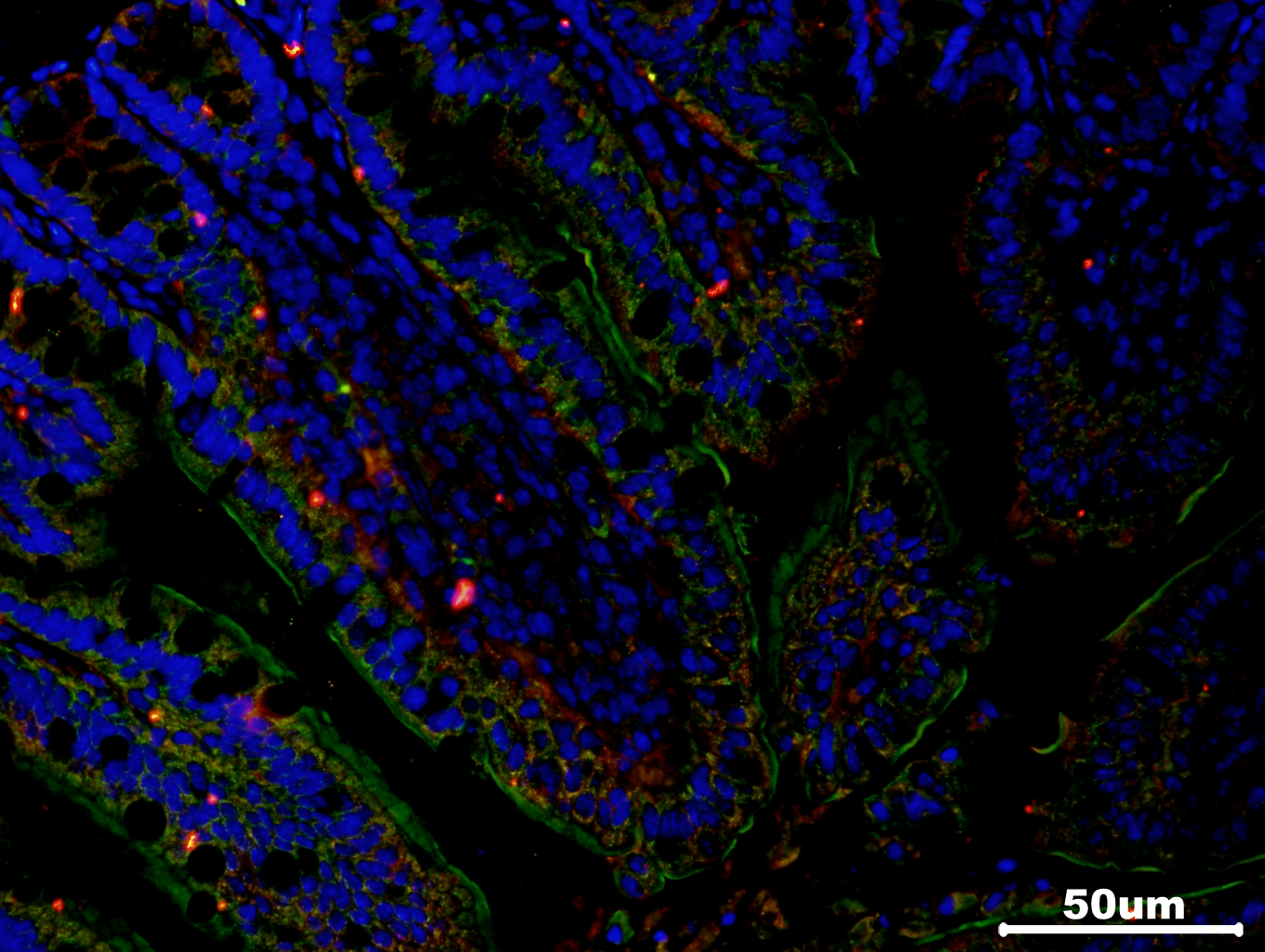

Supplement: Supplementary file 9 [file DataSheet_9.zip › D28-2-200-3-merge.tif]

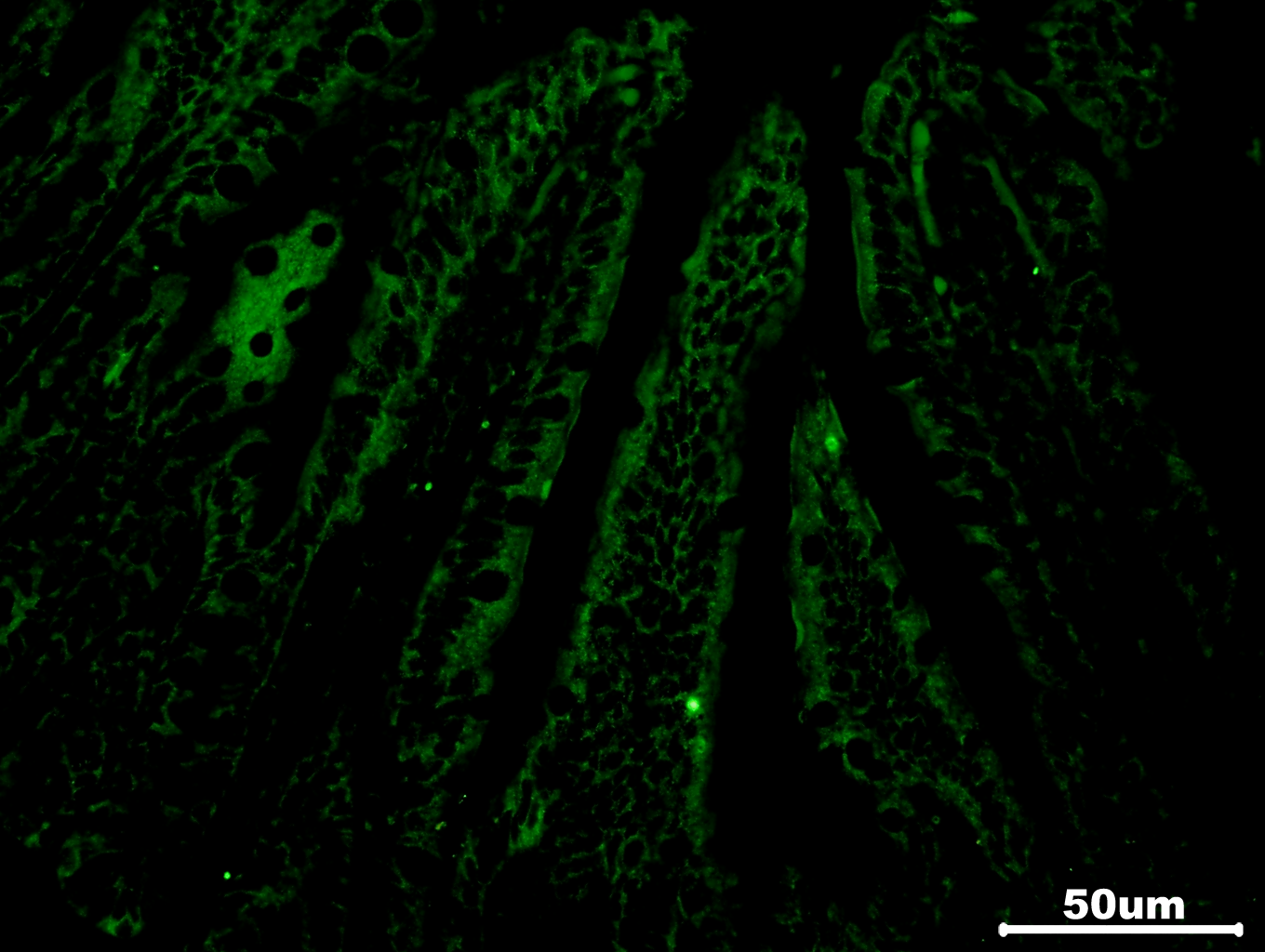

Supplement: Supplementary file 9 [file DataSheet_9.zip › D29-1-200-1-CD86.tif]

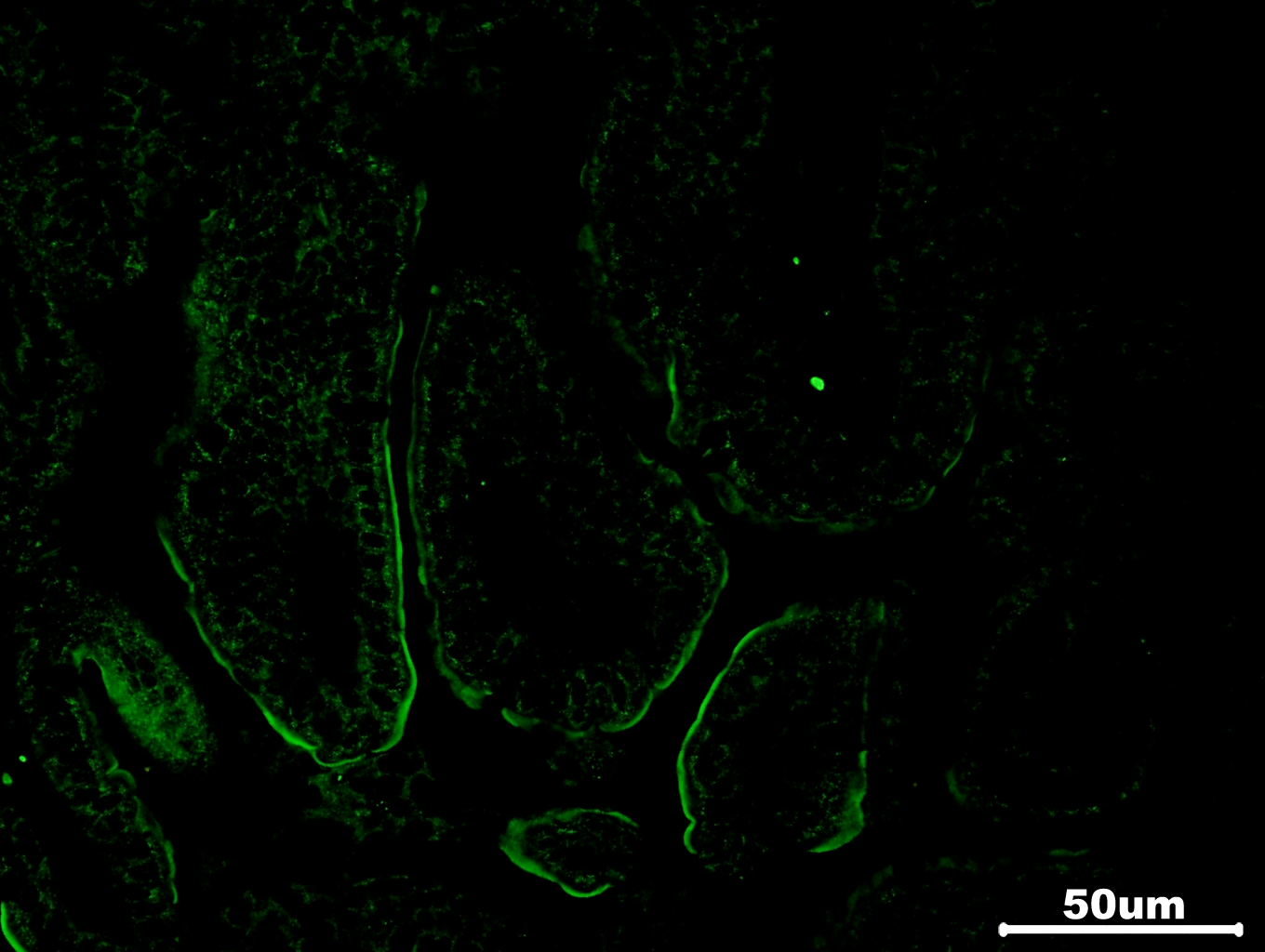

Supplement: Supplementary file 10 [file DataSheet_10.zip › F34-1-200-1-CD86.tif]

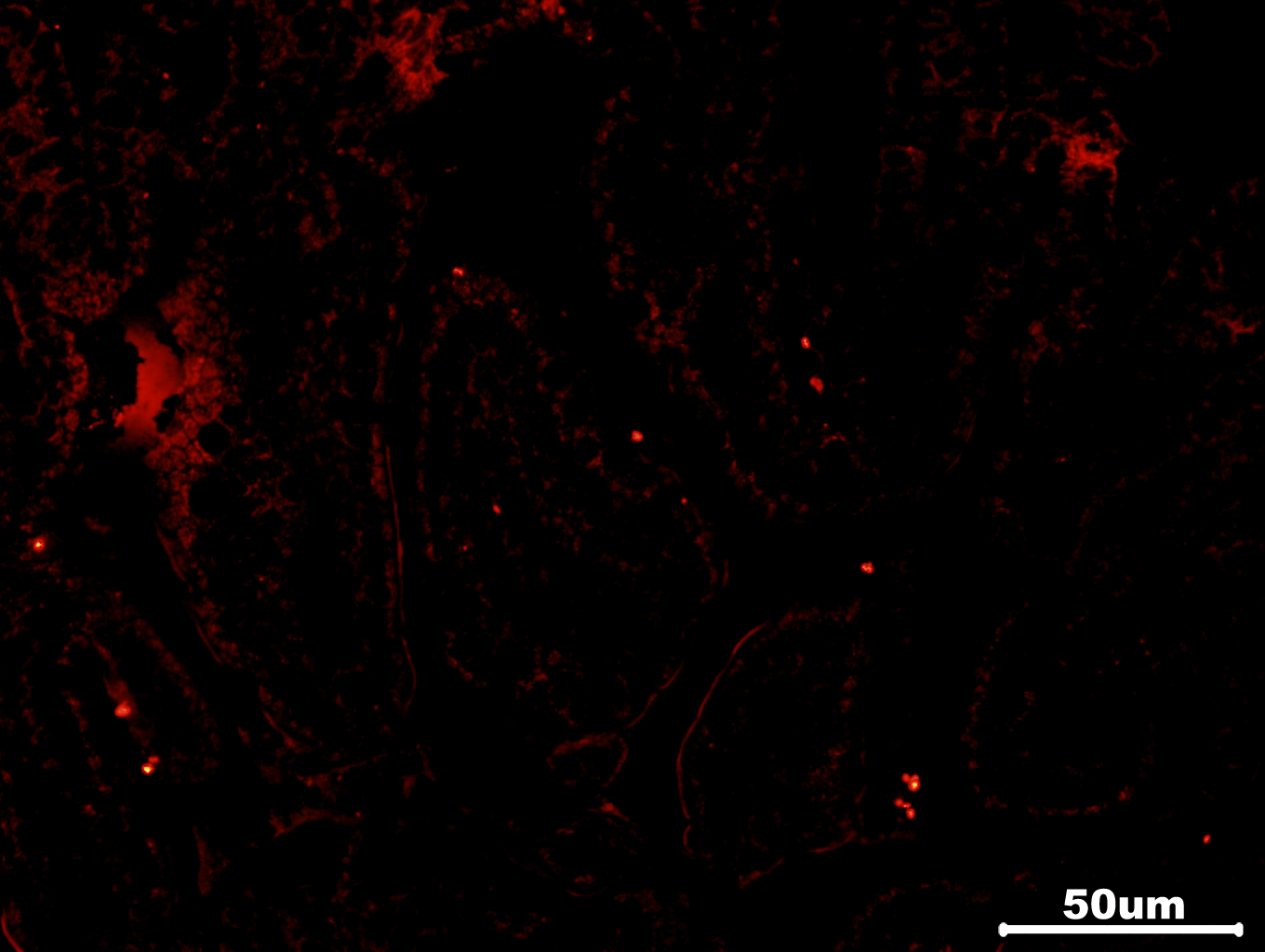

Supplement: Supplementary file 10 [file DataSheet_10.zip › F34-1-200-1-CD206.tif]

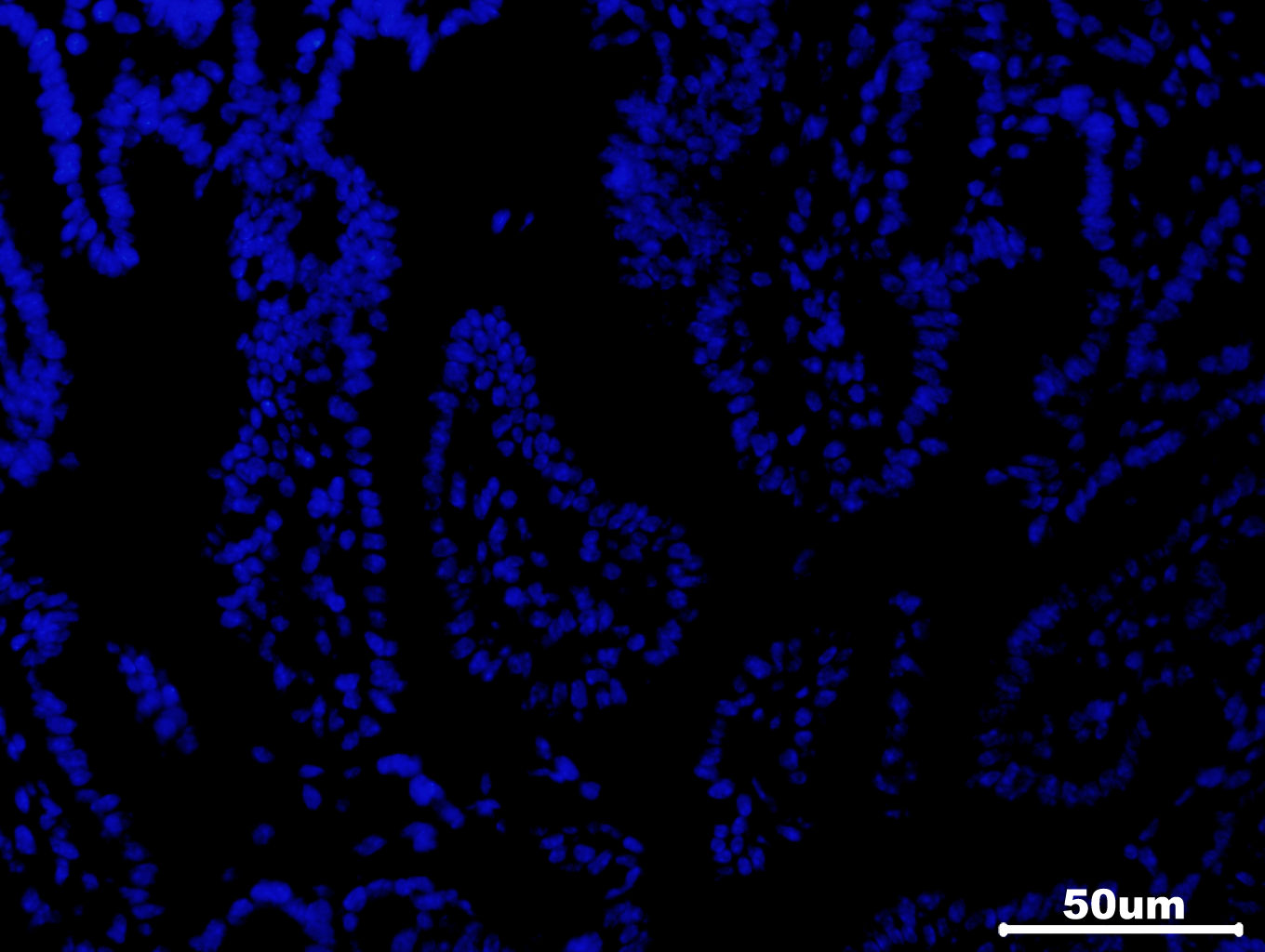

Supplement: Supplementary file 10 [file DataSheet_10.zip › F34-1-200-1-DAPI.tif]

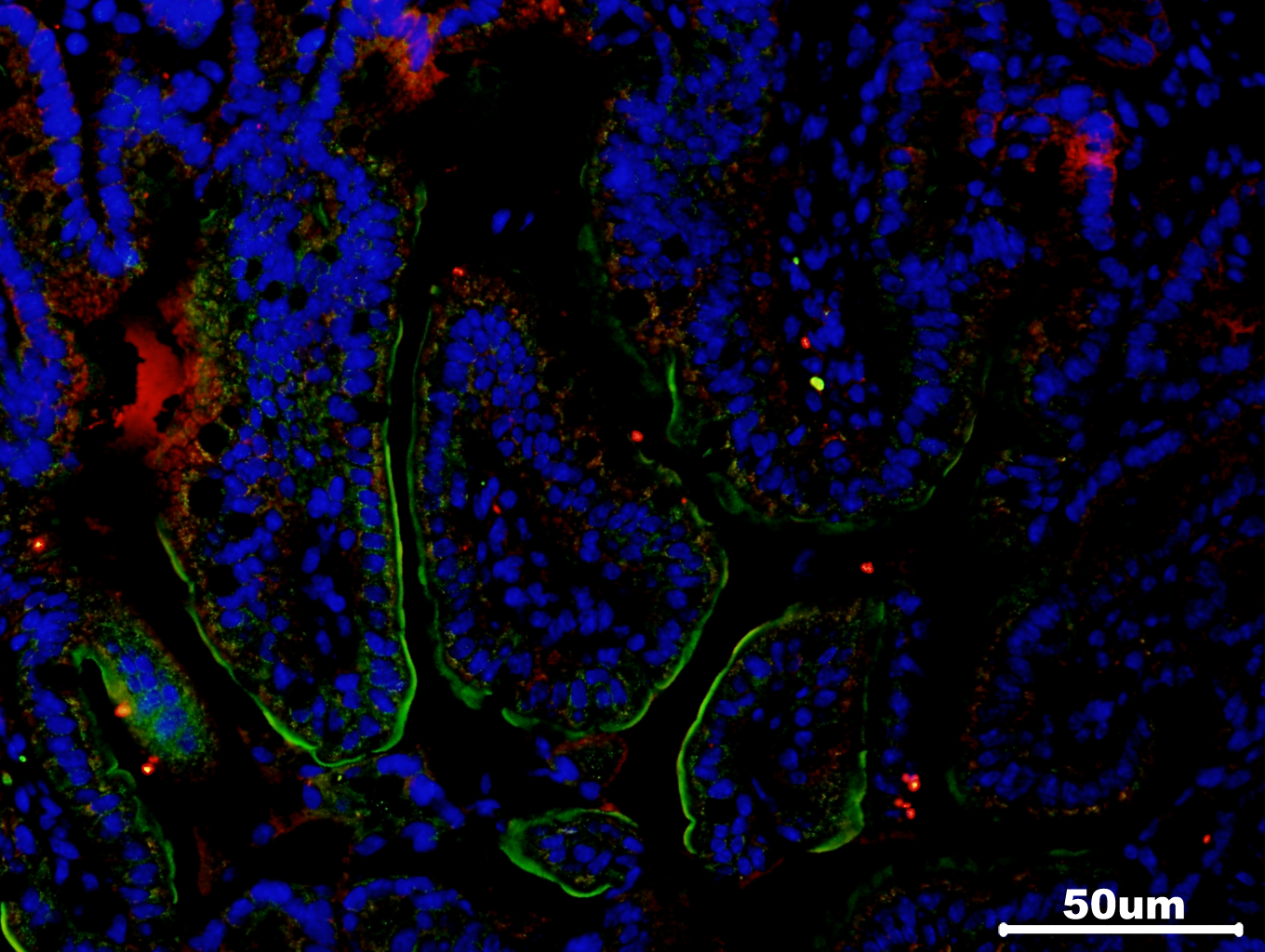

Supplement: Supplementary file 10 [file DataSheet_10.zip › F34-1-200-1-merge.tif]

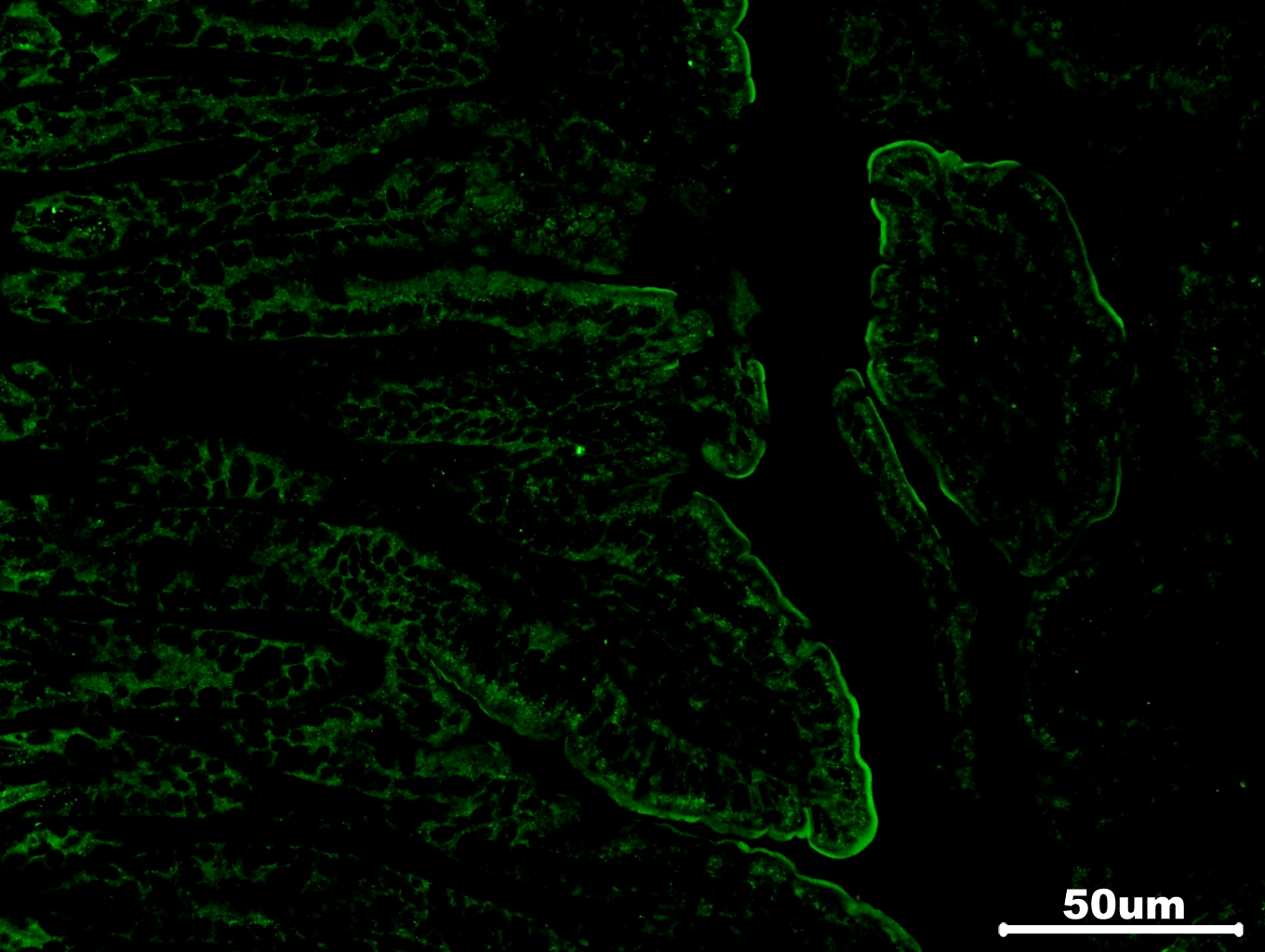

Supplement: Supplementary file 10 [file DataSheet_10.zip › F34-1-200-2-CD86.tif]

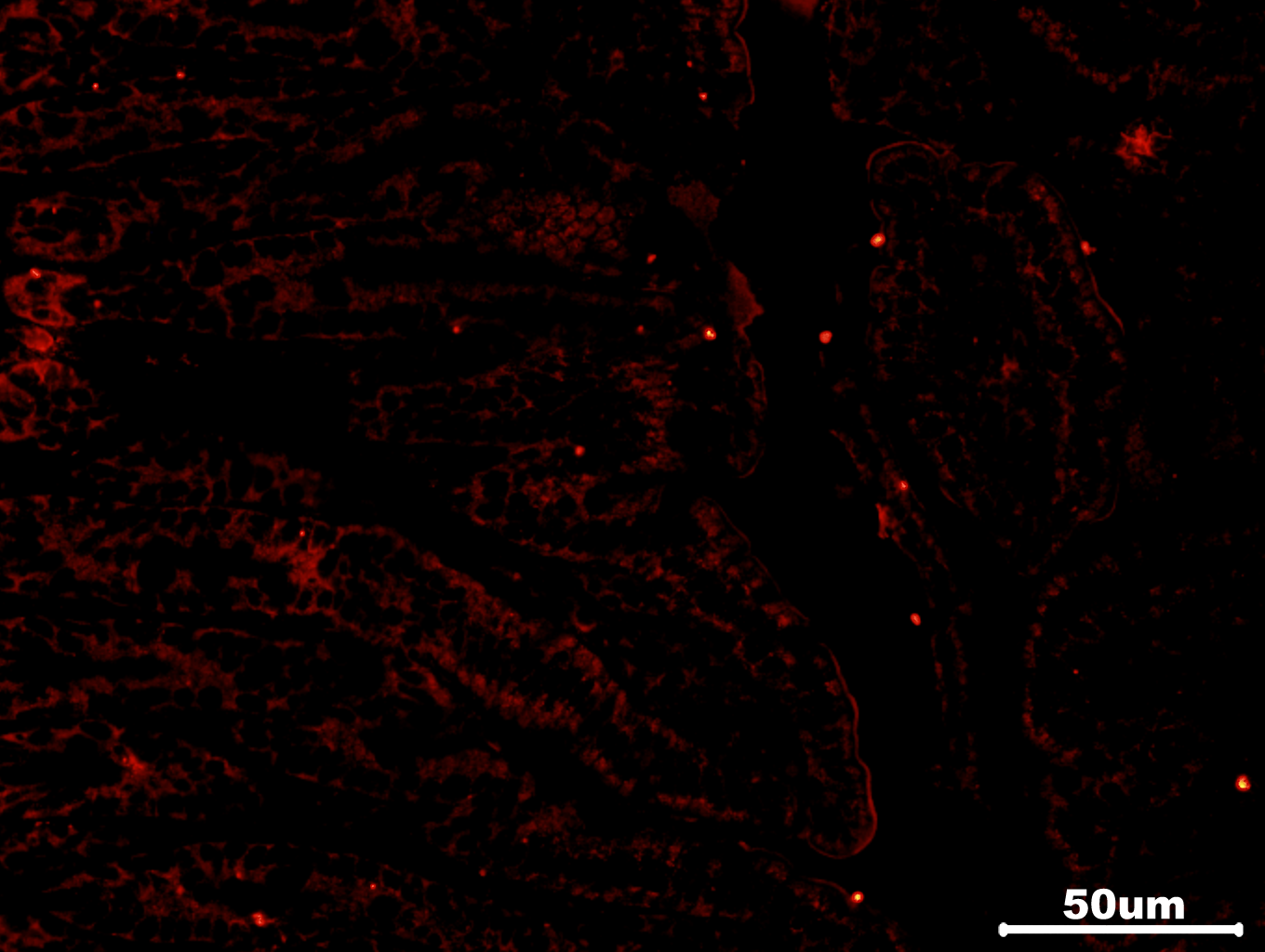

Supplement: Supplementary file 10 [file DataSheet_10.zip › F34-1-200-2-CD206.tif]

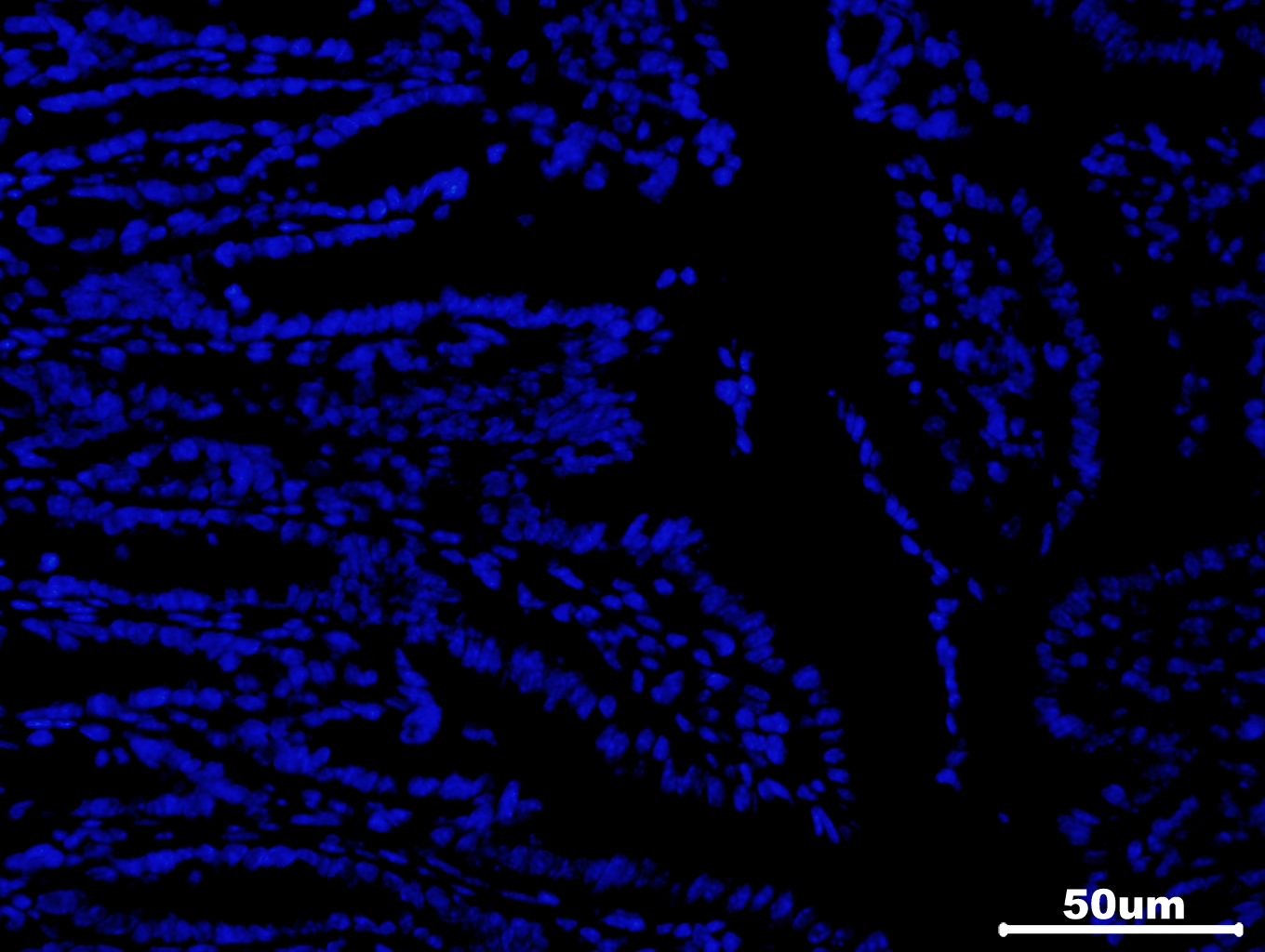

Supplement: Supplementary file 10 [file DataSheet_10.zip › F34-1-200-2-DAPI.tif]

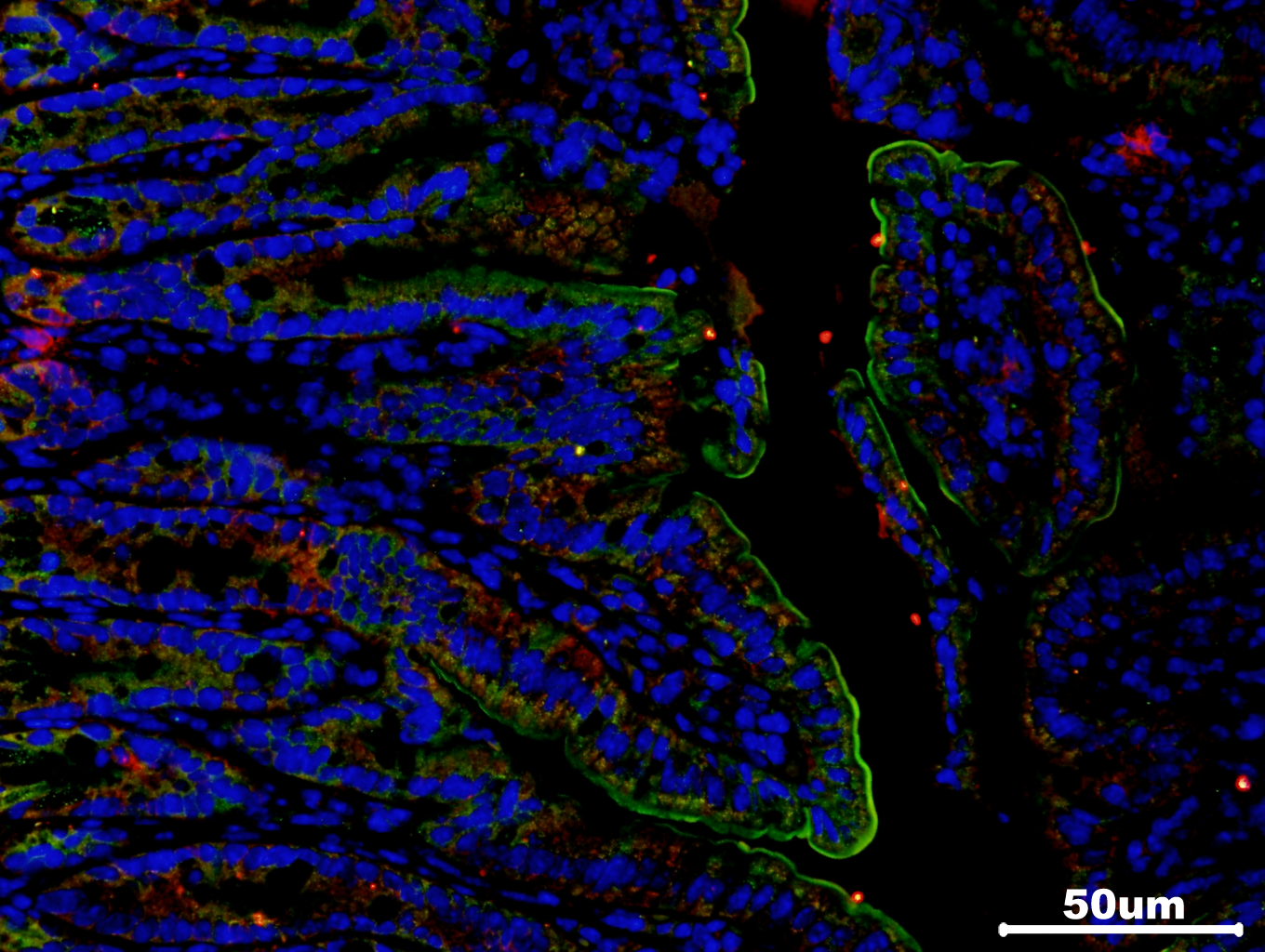

Supplement: Supplementary file 10 [file DataSheet_10.zip › F34-1-200-2-merge.tif]

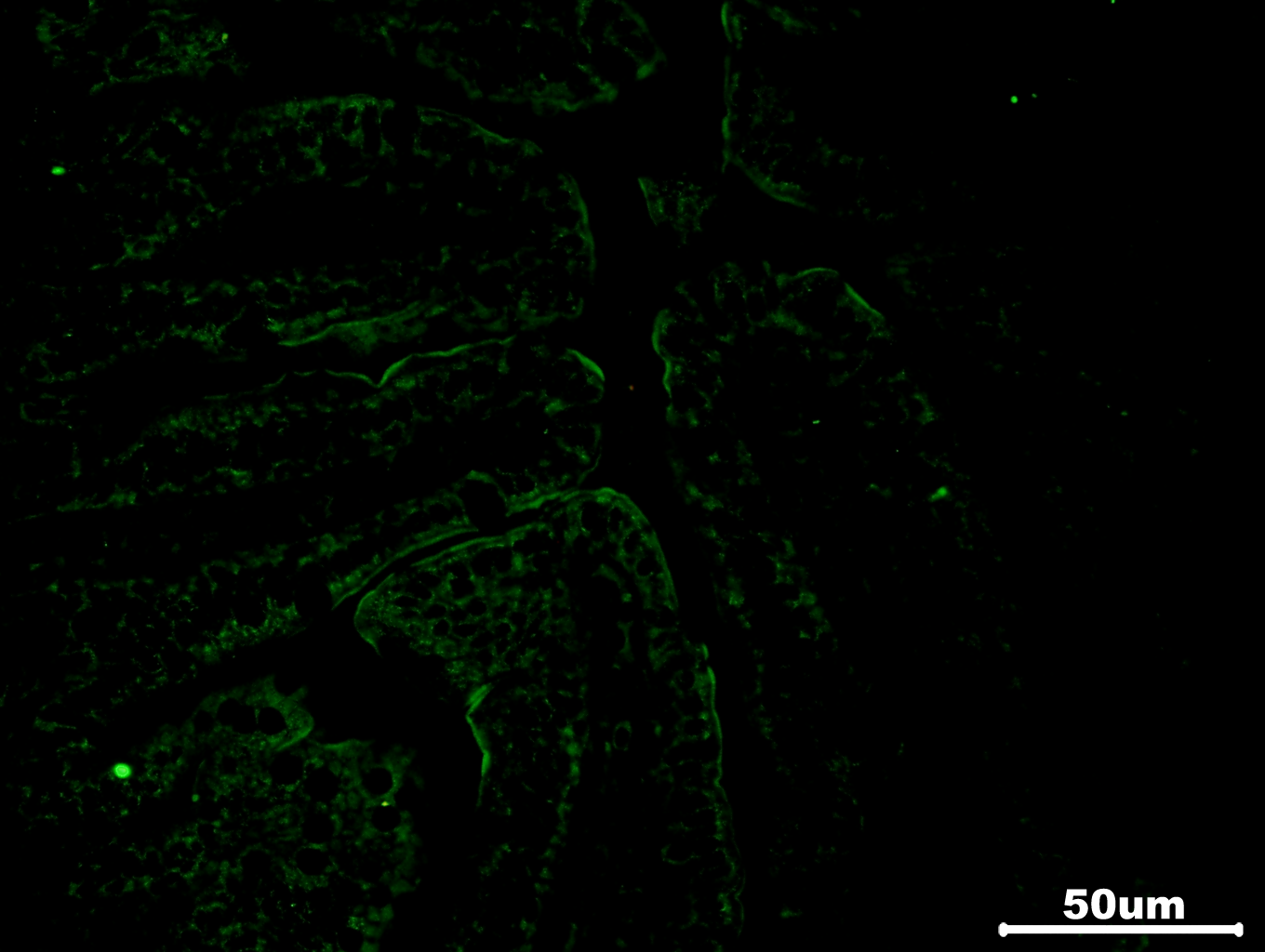

Supplement: Supplementary file 10 [file DataSheet_10.zip › F34-1-200-3-CD86.tif]

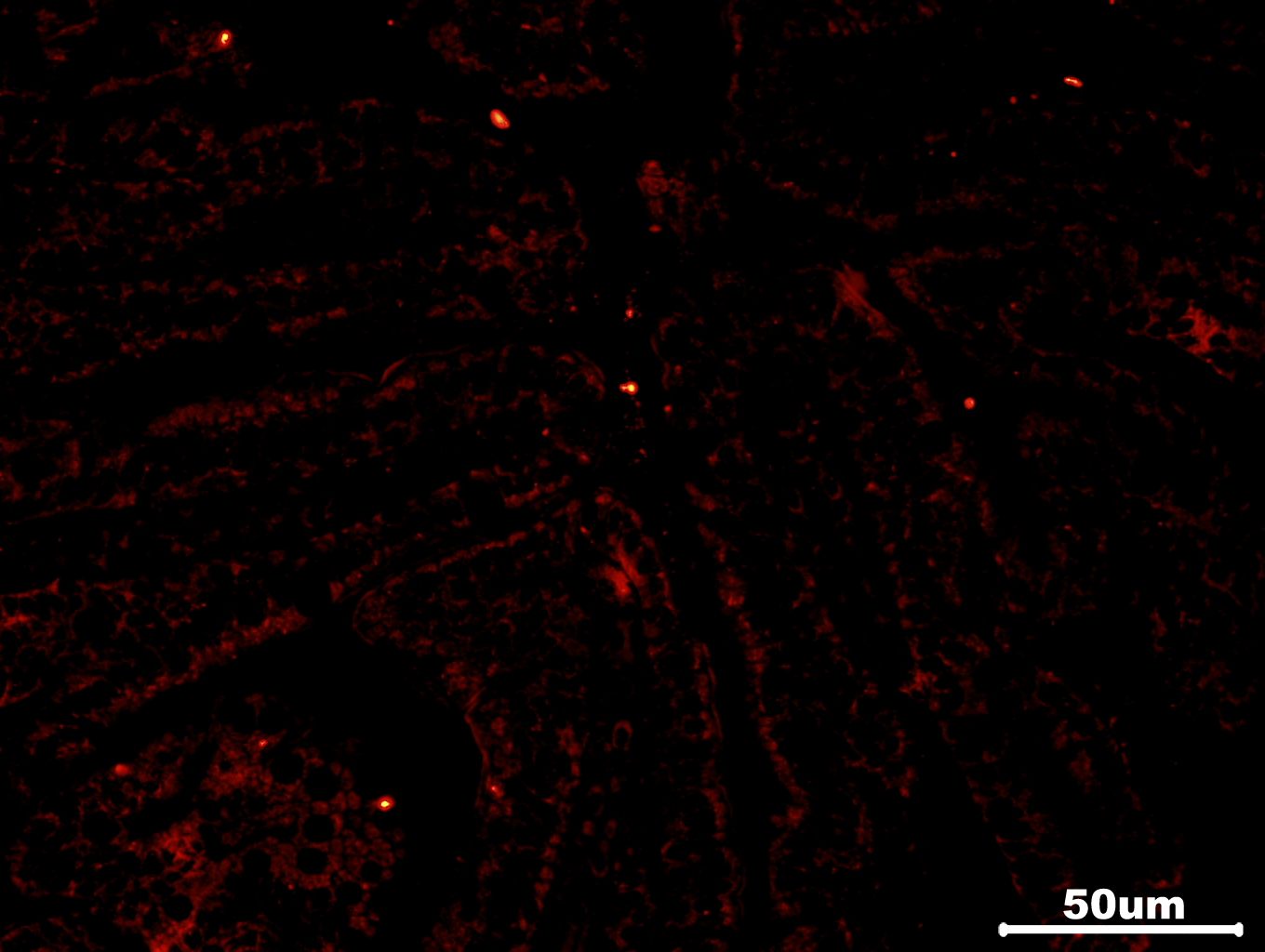

Supplement: Supplementary file 10 [file DataSheet_10.zip › F34-1-200-3-CD206.tif]

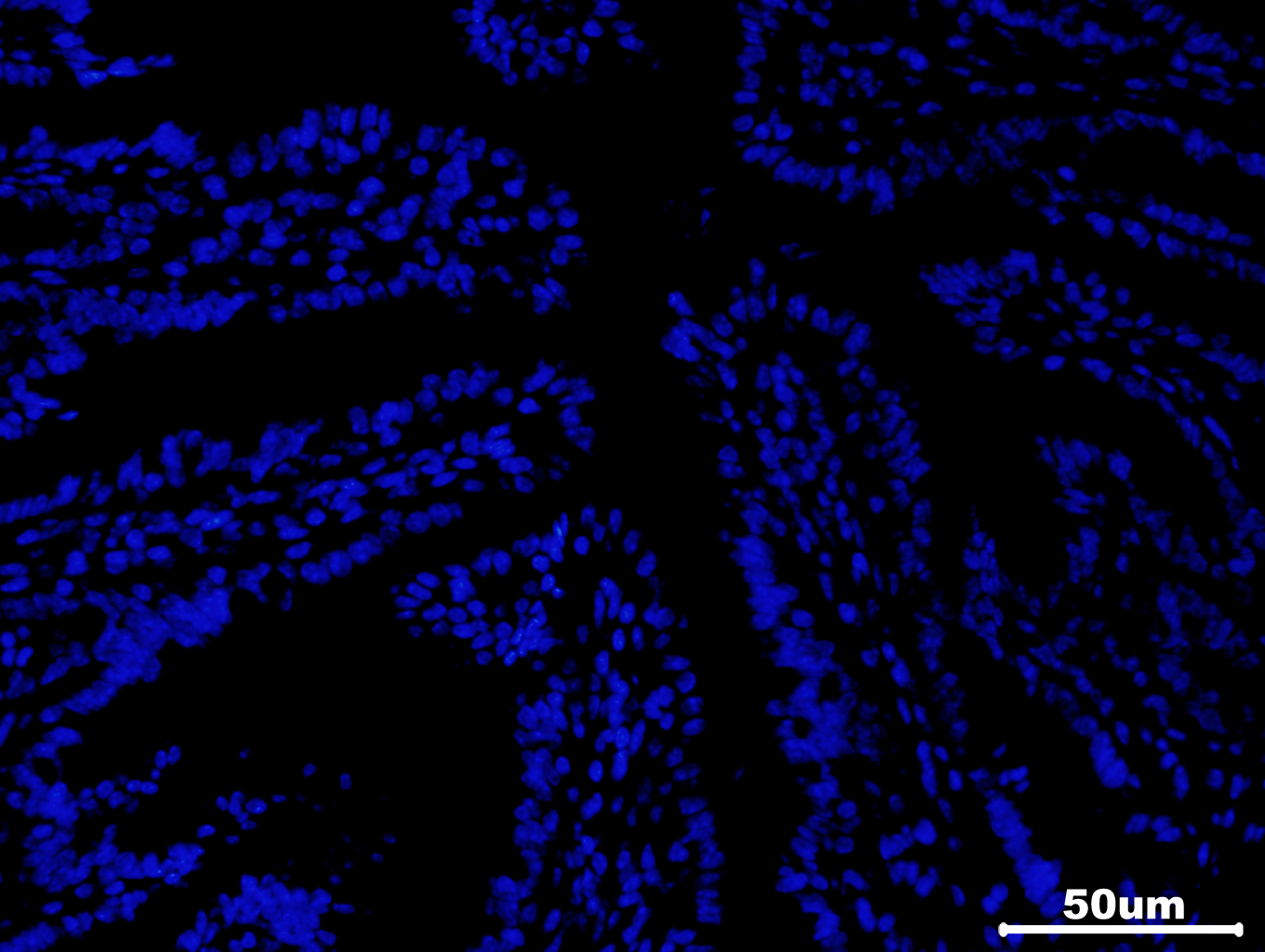

Supplement: Supplementary file 10 [file DataSheet_10.zip › F34-1-200-3-DAPI.tif]

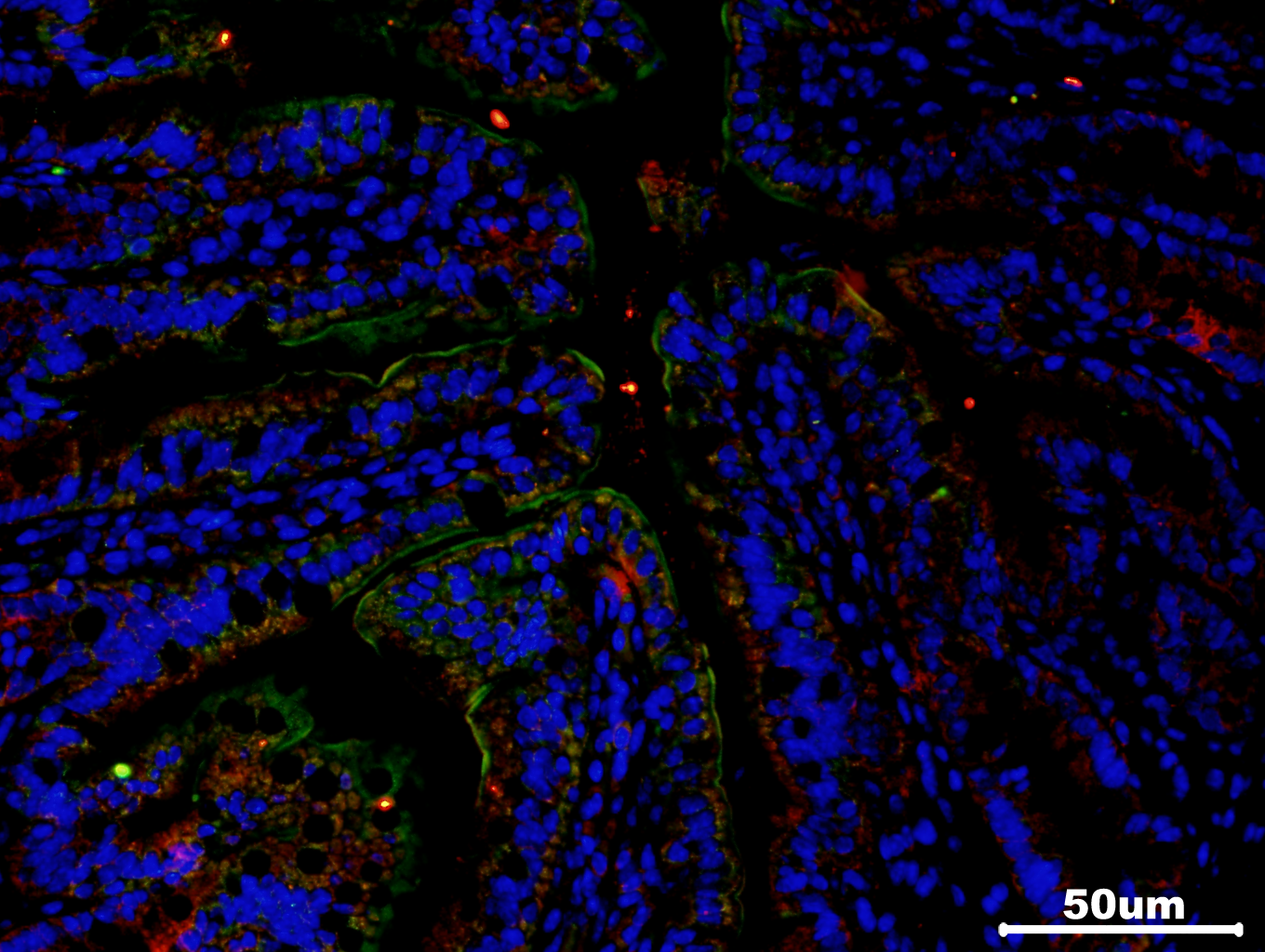

Supplement: Supplementary file 10 [file DataSheet_10.zip › F34-1-200-3-merge.tif]

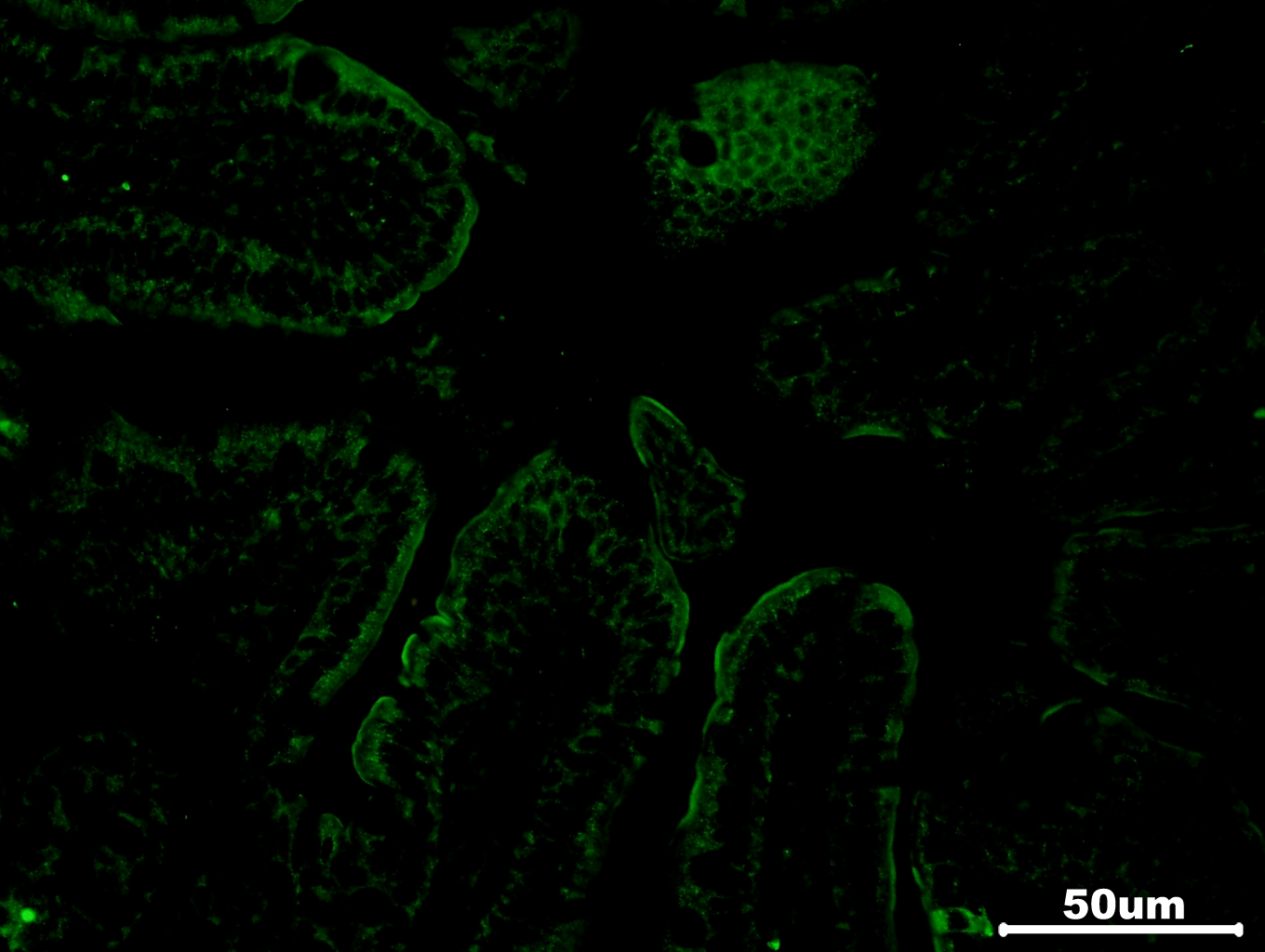

Supplement: Supplementary file 10 [file DataSheet_10.zip › F34-2-200-1-CD86.tif]

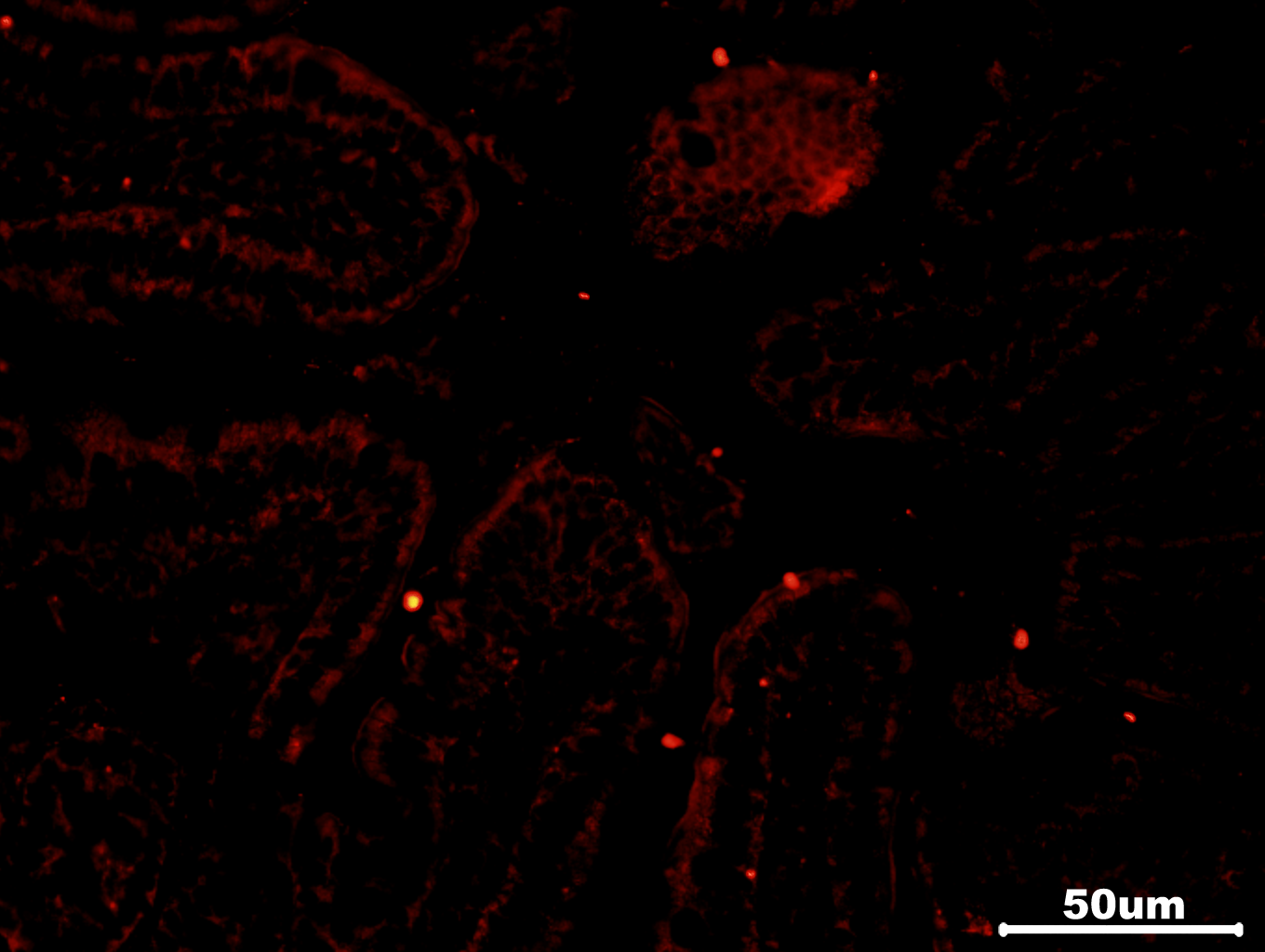

Supplement: Supplementary file 10 [file DataSheet_10.zip › F34-2-200-1-CD206.tif]

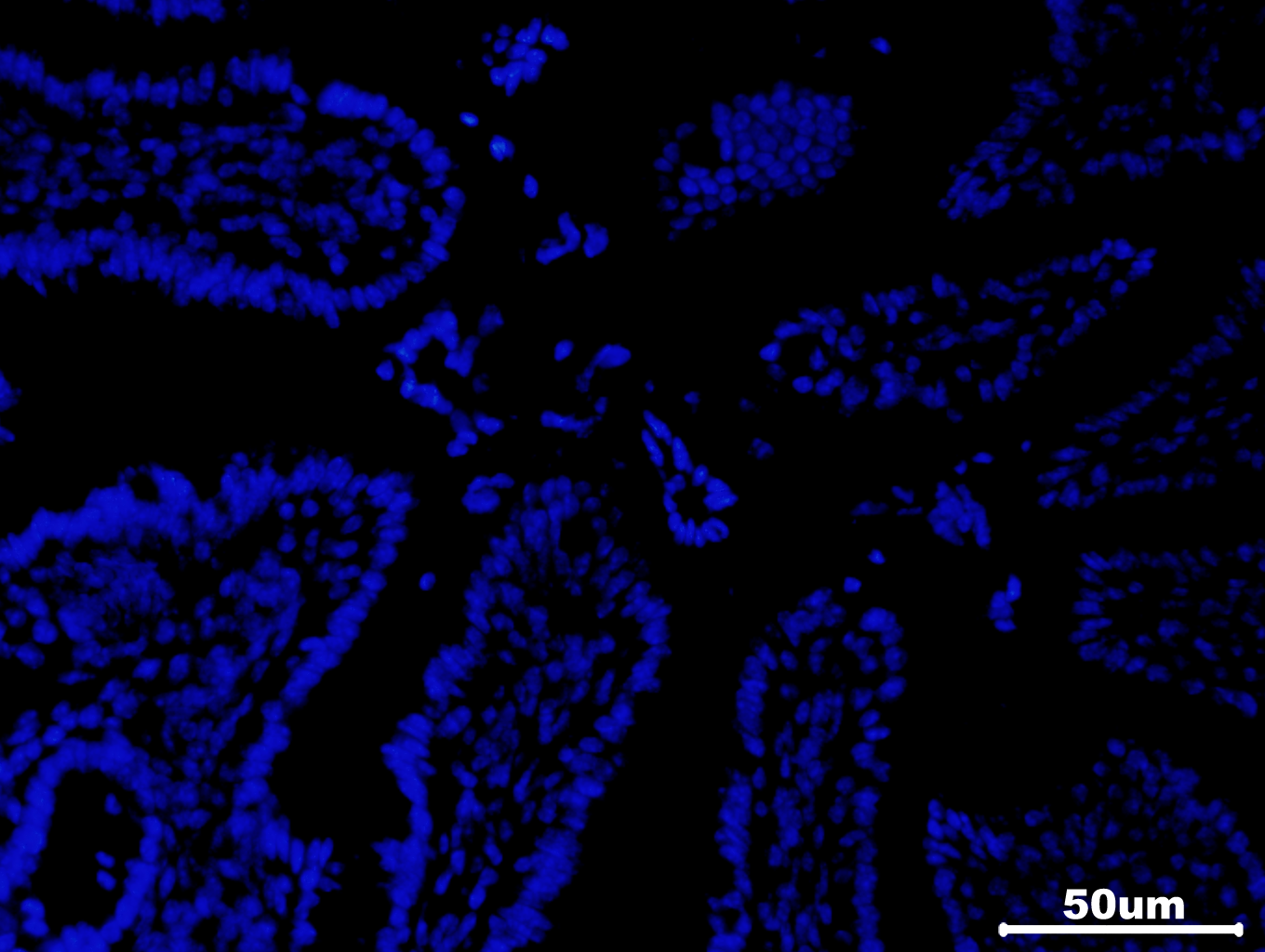

Supplement: Supplementary file 10 [file DataSheet_10.zip › F34-2-200-1-DAPI.tif]

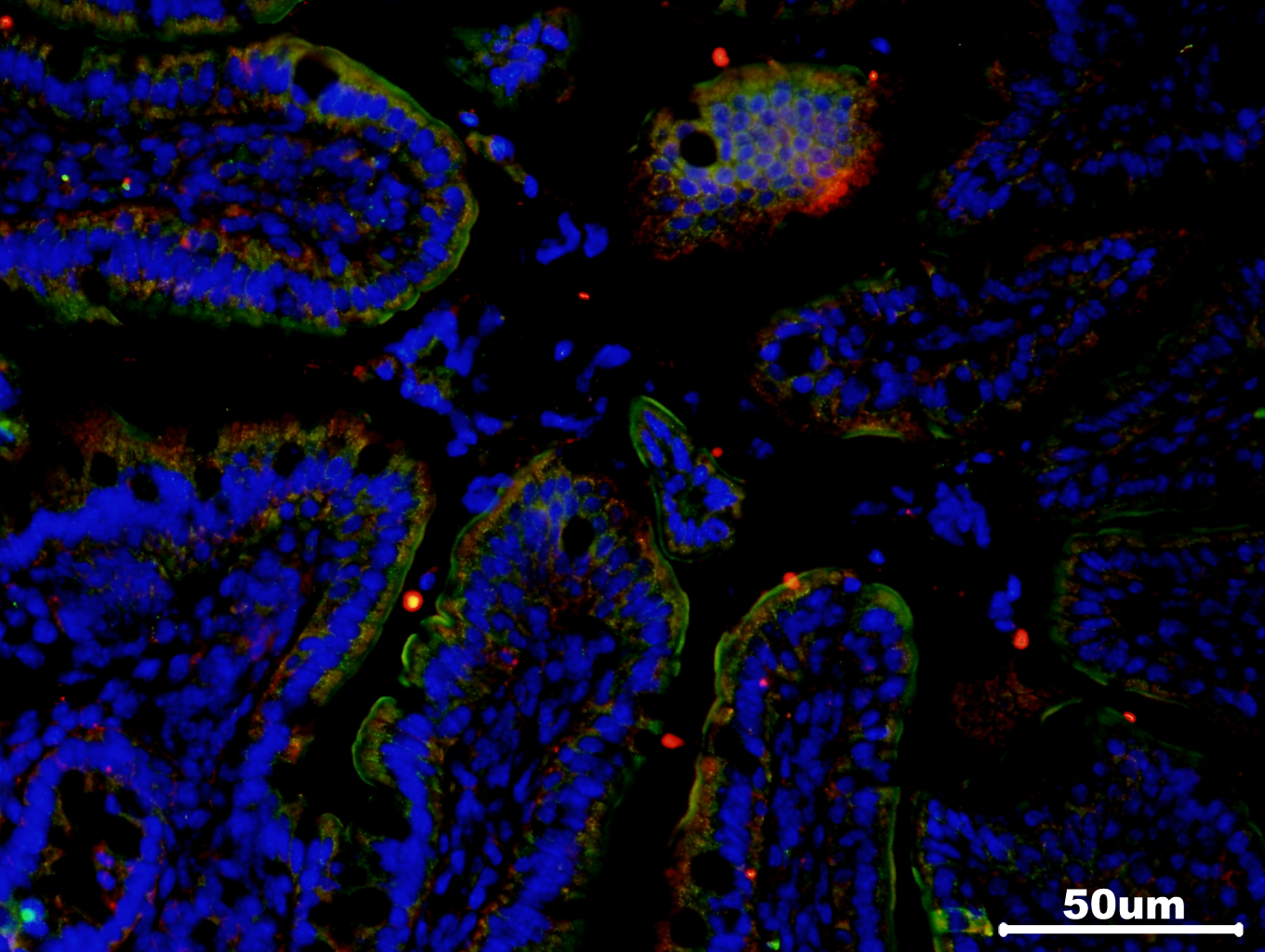

Supplement: Supplementary file 10 [file DataSheet_10.zip › F34-2-200-1-merge.tif]

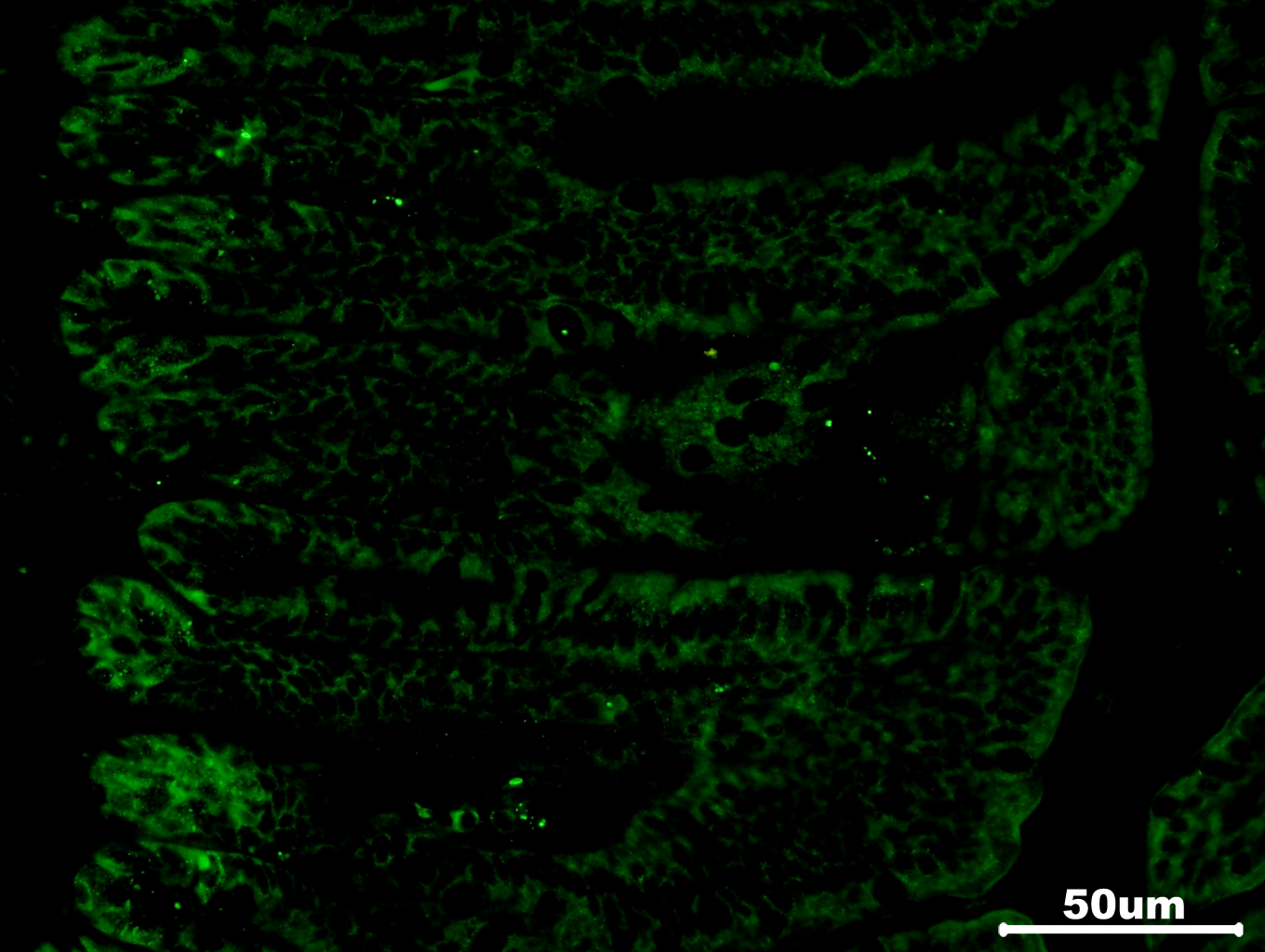

Supplement: Supplementary file 10 [file DataSheet_10.zip › F34-2-200-2-CD86.tif]

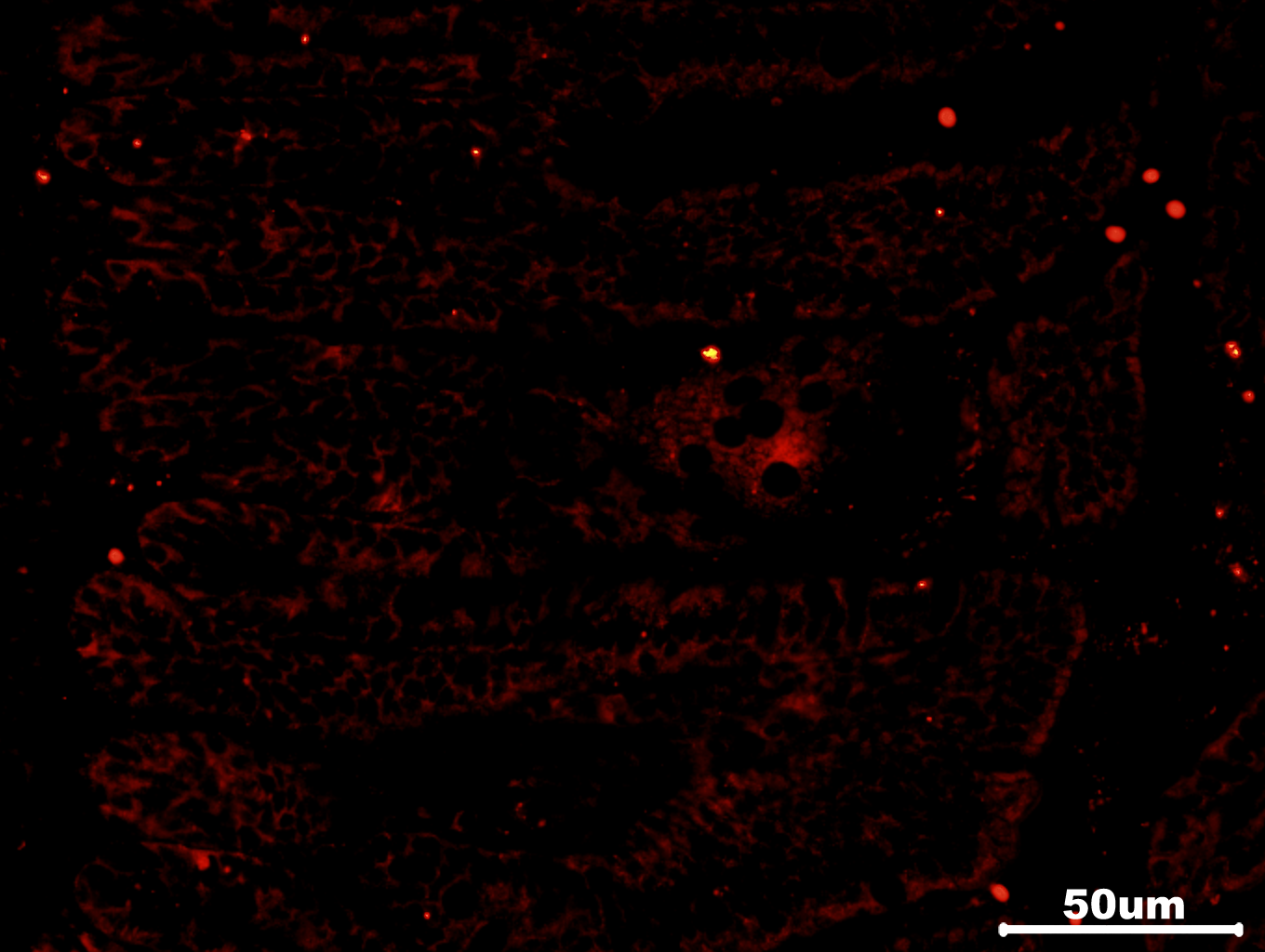

Supplement: Supplementary file 10 [file DataSheet_10.zip › F34-2-200-2-CD206.tif]

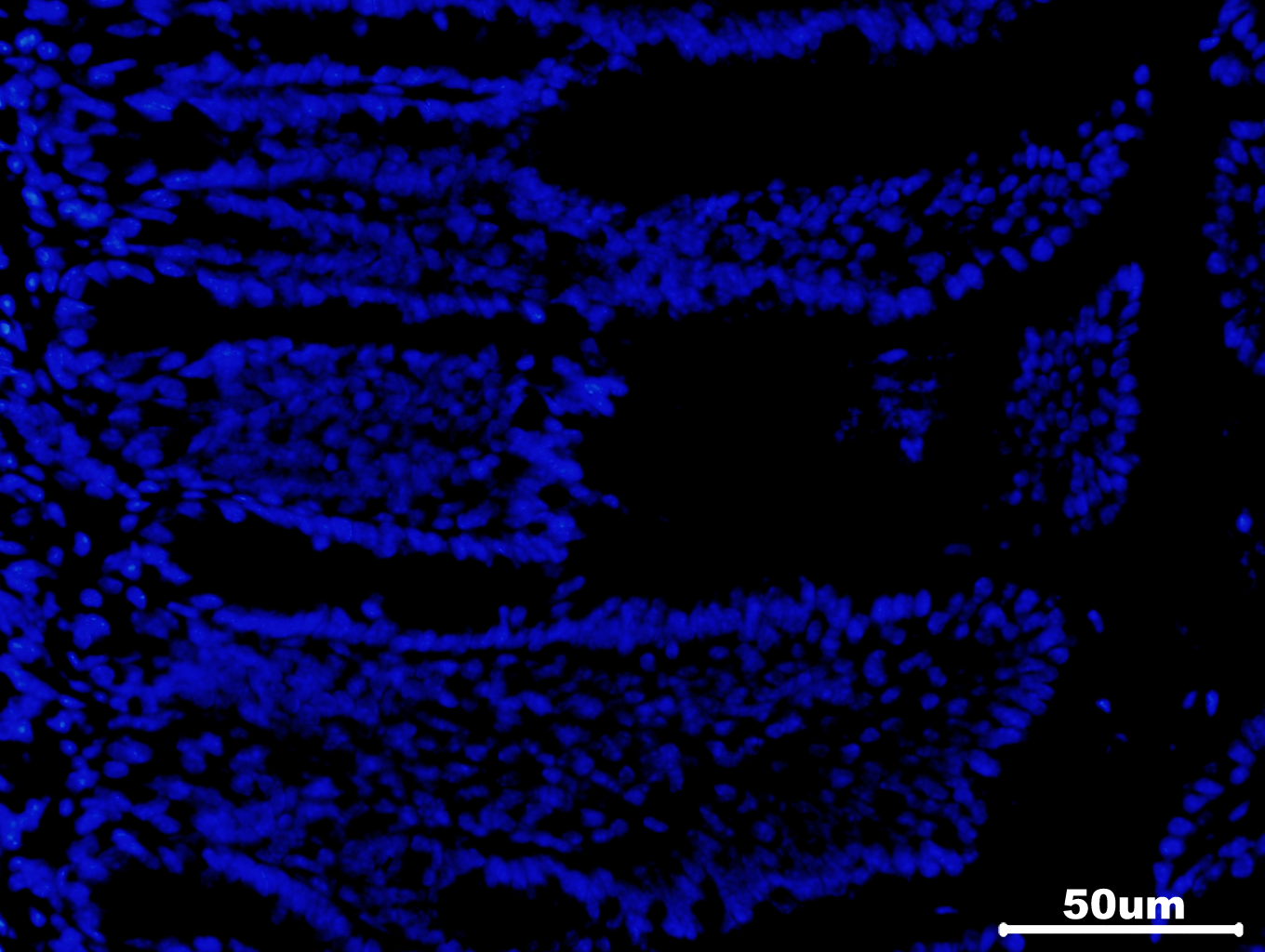

Supplement: Supplementary file 10 [file DataSheet_10.zip › F34-2-200-2-DAPI.tif]

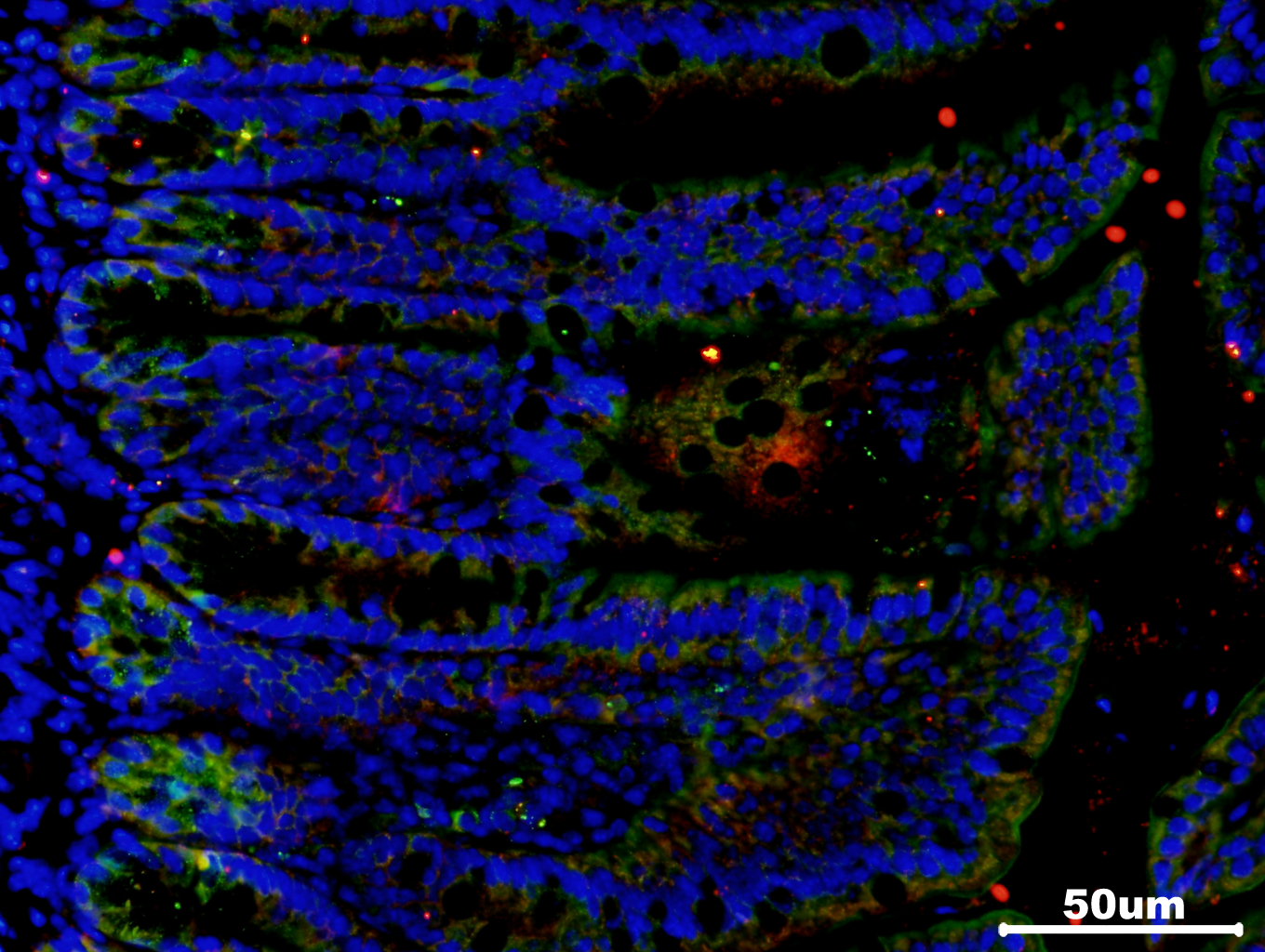

Supplement: Supplementary file 10 [file DataSheet_10.zip › F34-2-200-2-merge.tif]

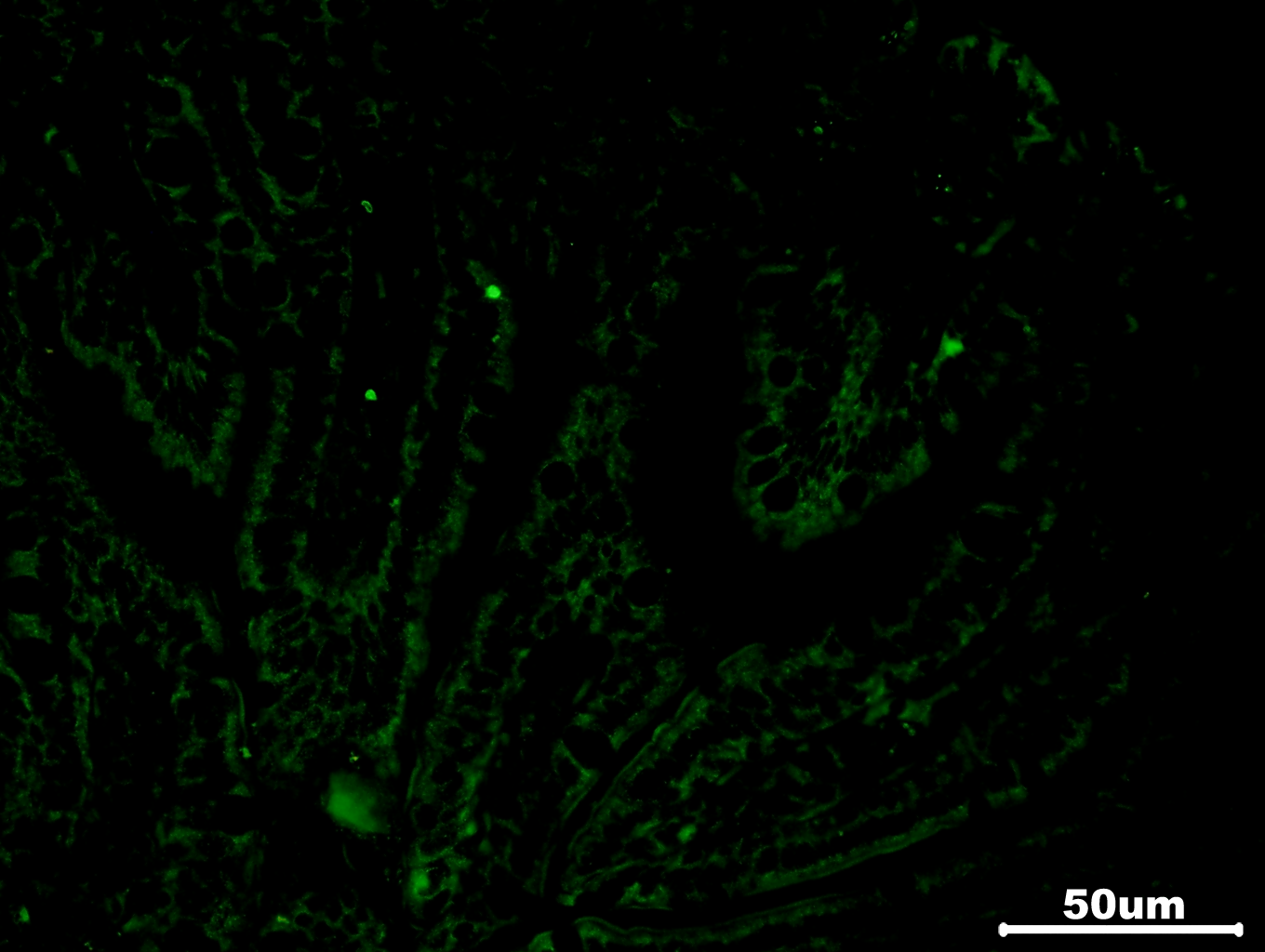

Supplement: Supplementary file 10 [file DataSheet_10.zip › F34-2-200-3-CD86.tif]

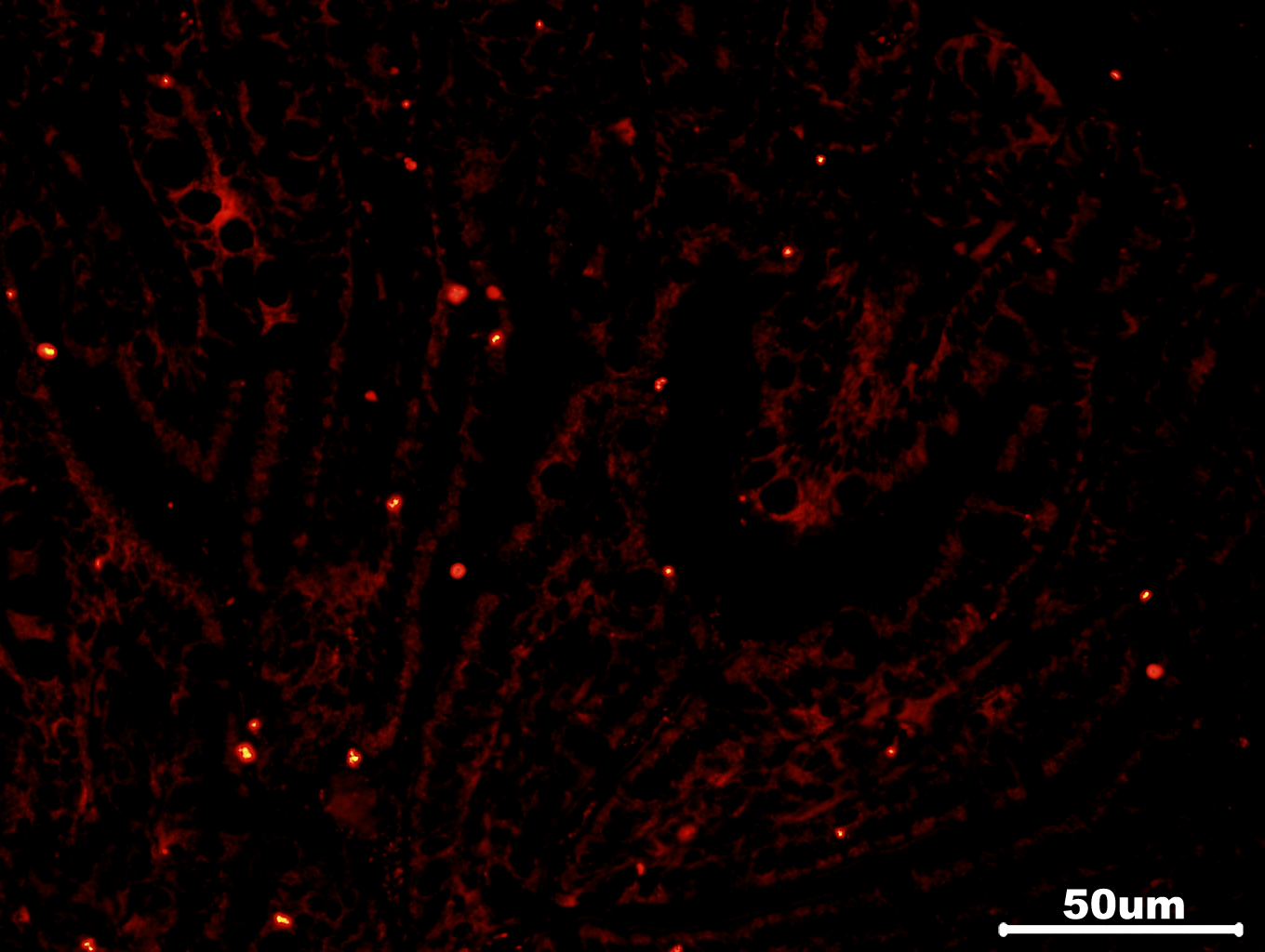

Supplement: Supplementary file 10 [file DataSheet_10.zip › F34-2-200-3-CD206.tif]

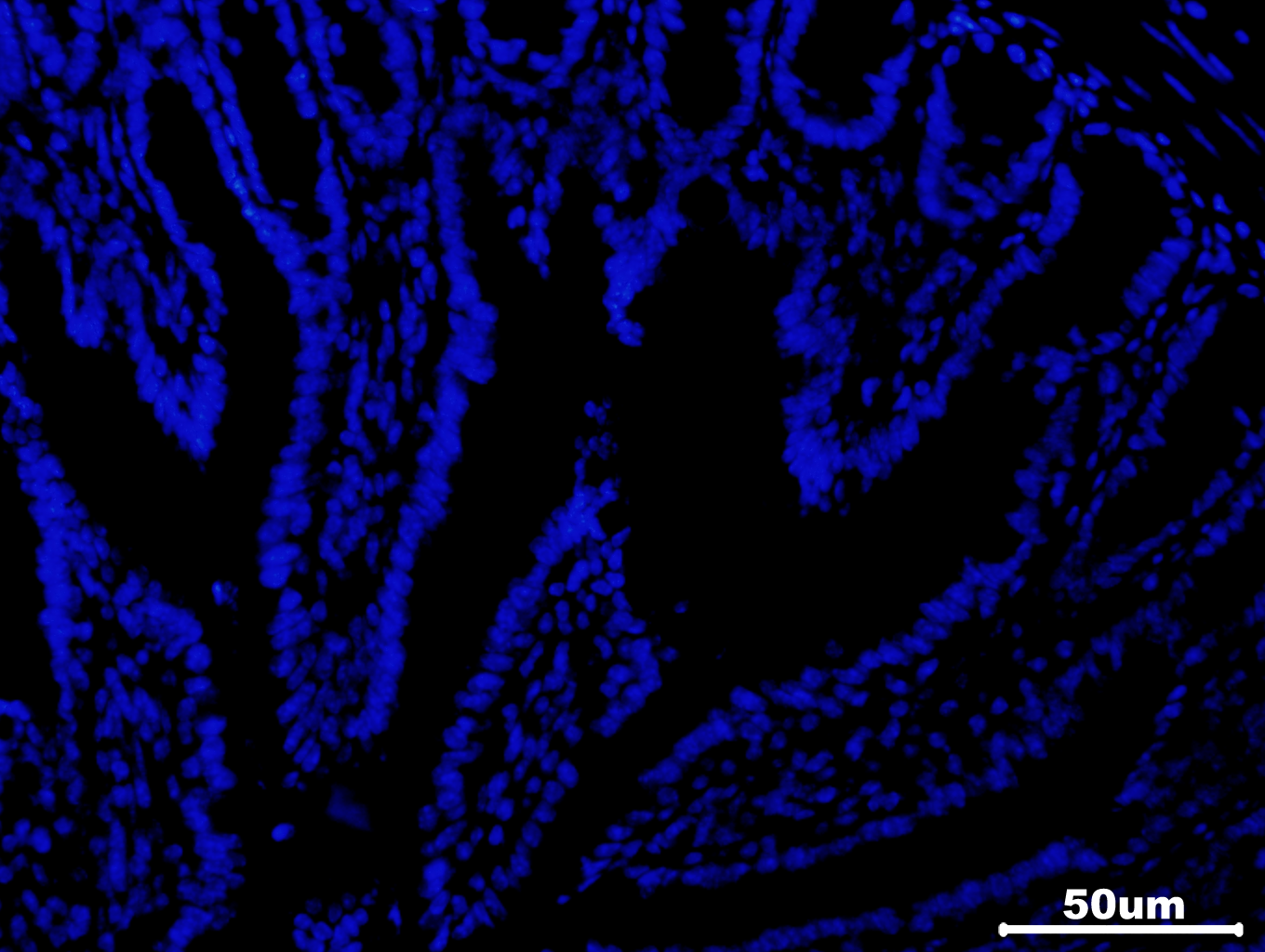

Supplement: Supplementary file 10 [file DataSheet_10.zip › F34-2-200-3-DAPI.tif]

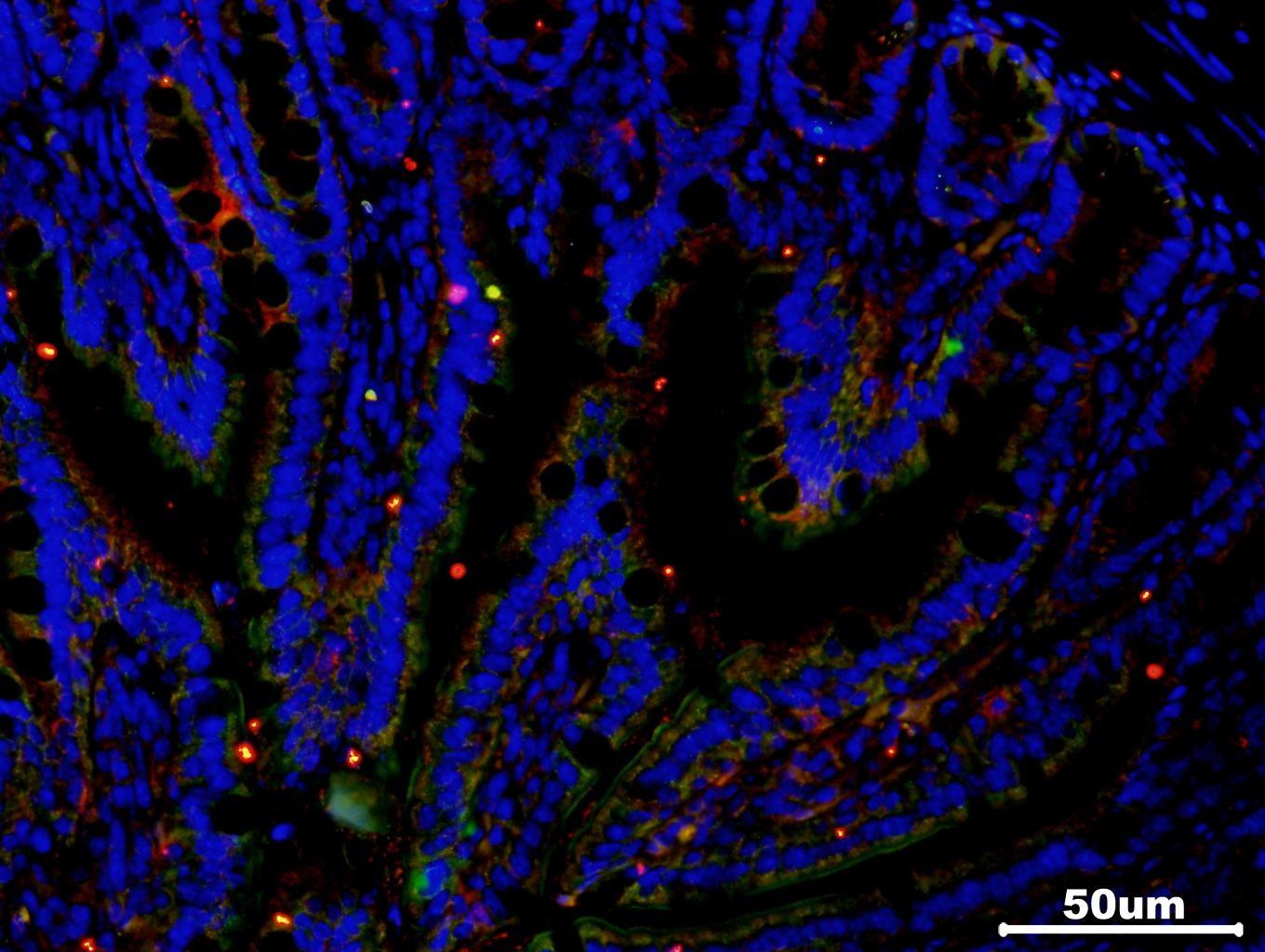

Supplement: Supplementary file 10 [file DataSheet_10.zip › F34-2-200-3-merge.tif]

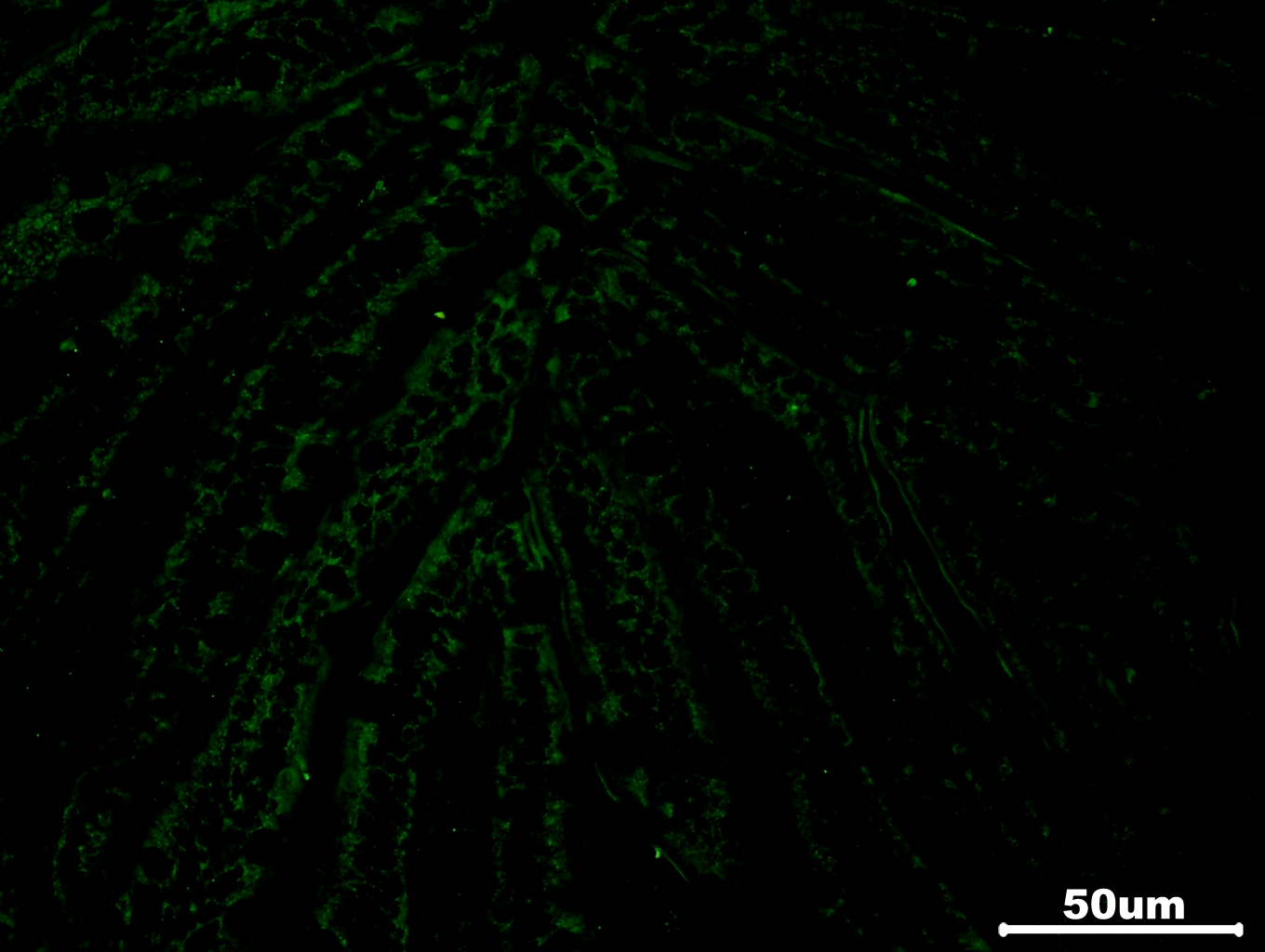

Supplement: Supplementary file 10 [file DataSheet_10.zip › F35-1-200-1-CD86.tif]

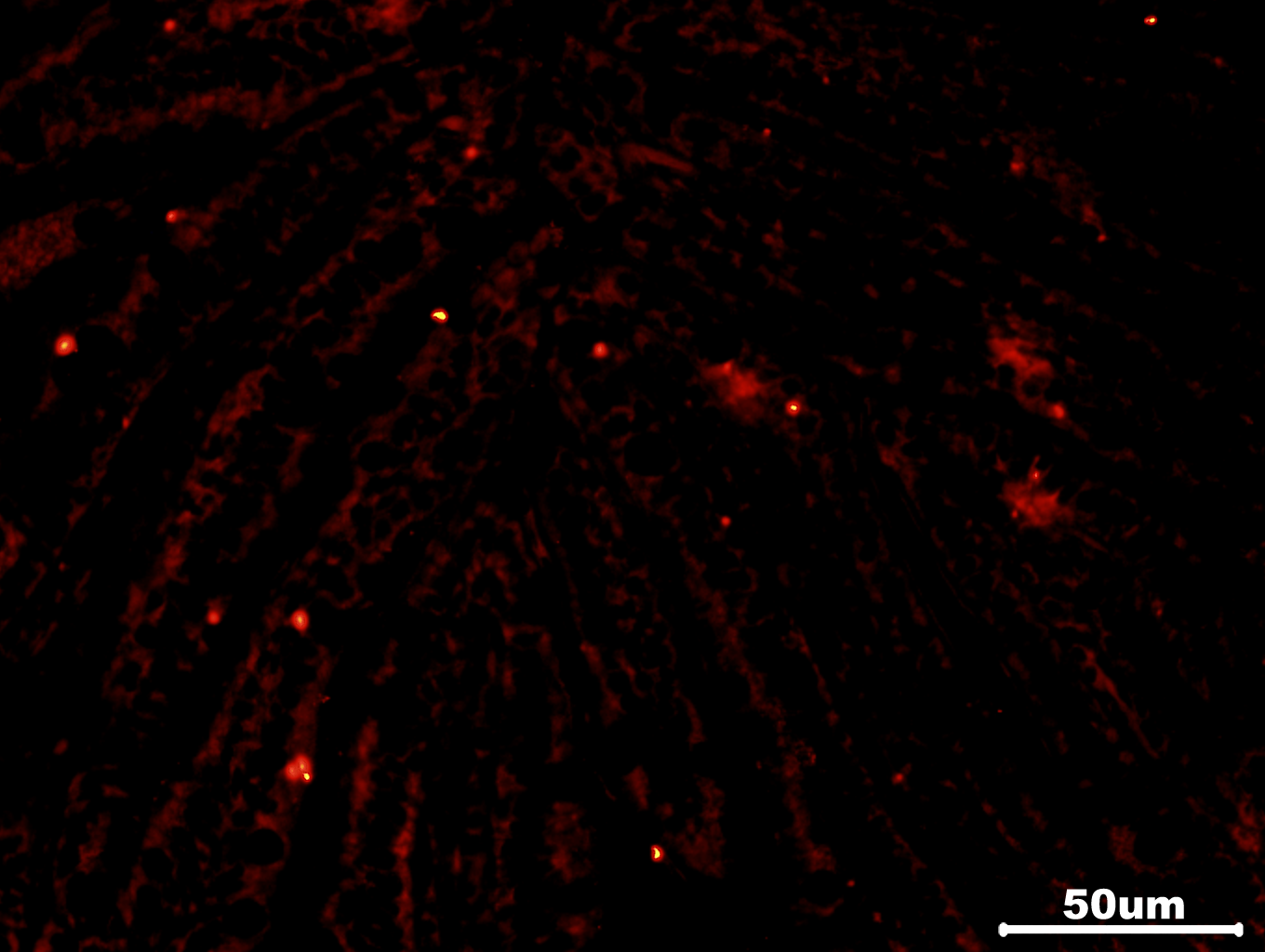

Supplement: Supplementary file 11 [file DataSheet_11.zip › F35-1-200-1-CD206.tif]

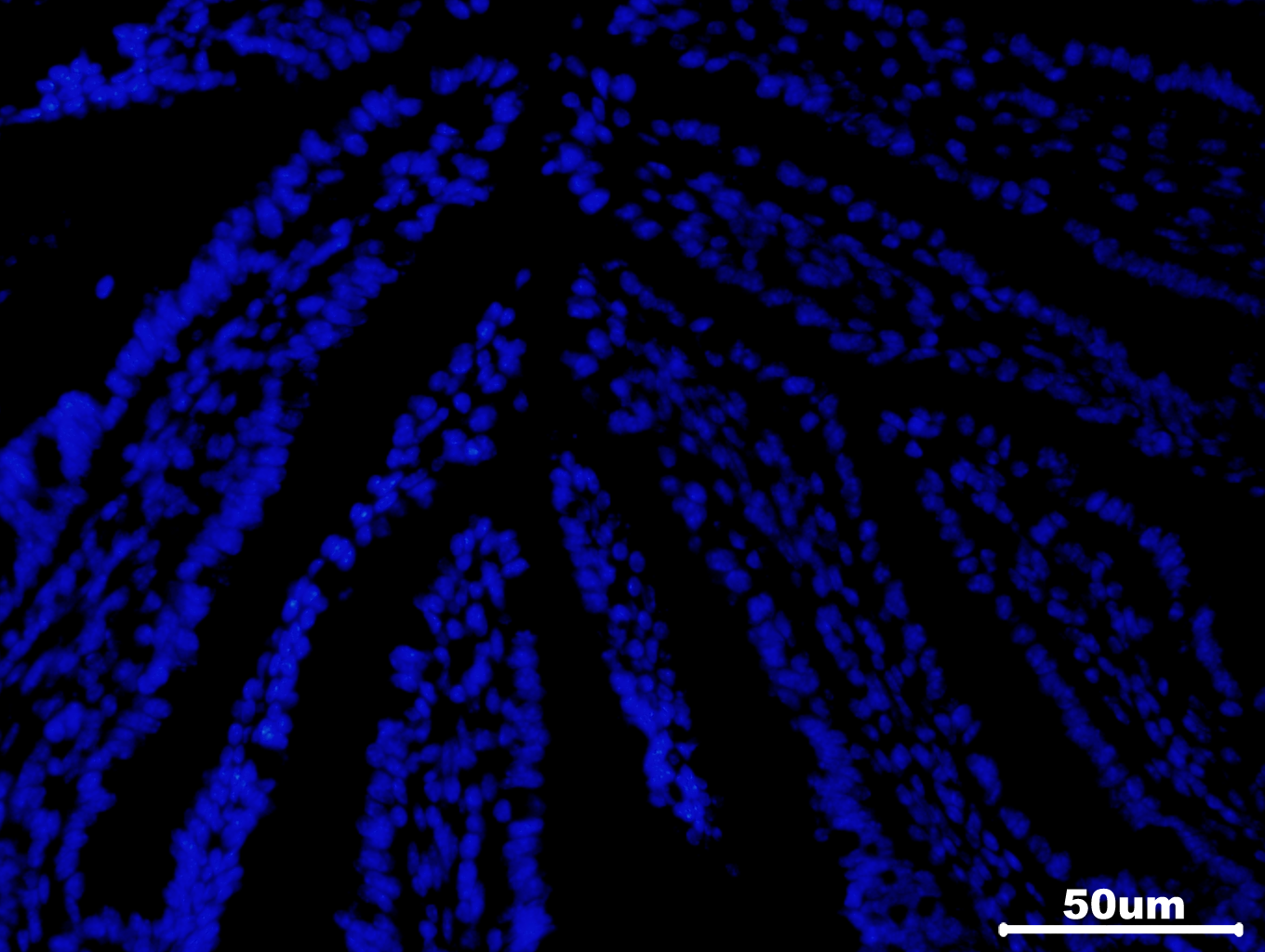

Supplement: Supplementary file 11 [file DataSheet_11.zip › F35-1-200-1-DAPI.tif]

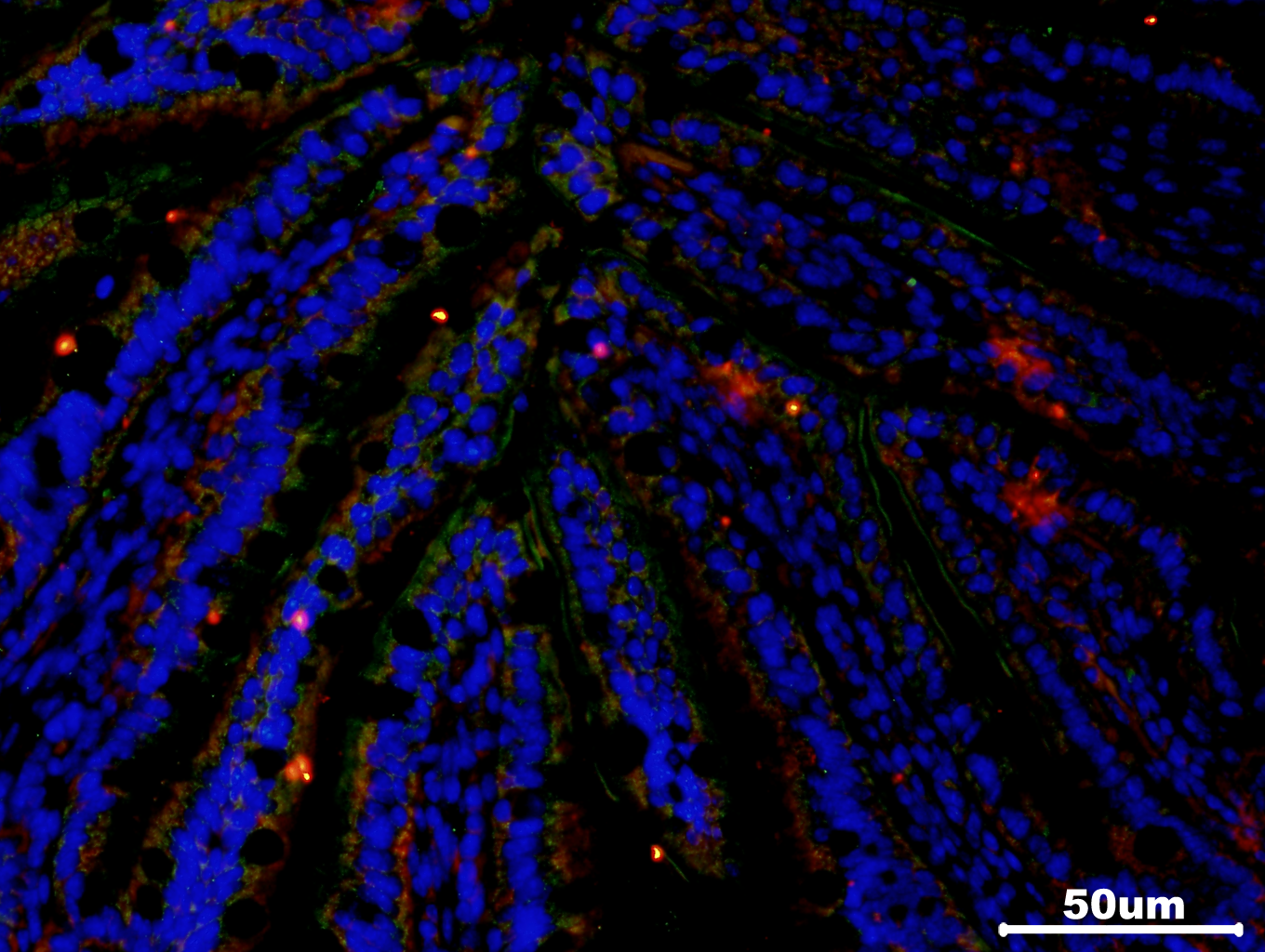

Supplement: Supplementary file 11 [file DataSheet_11.zip › F35-1-200-1-merge.tif]

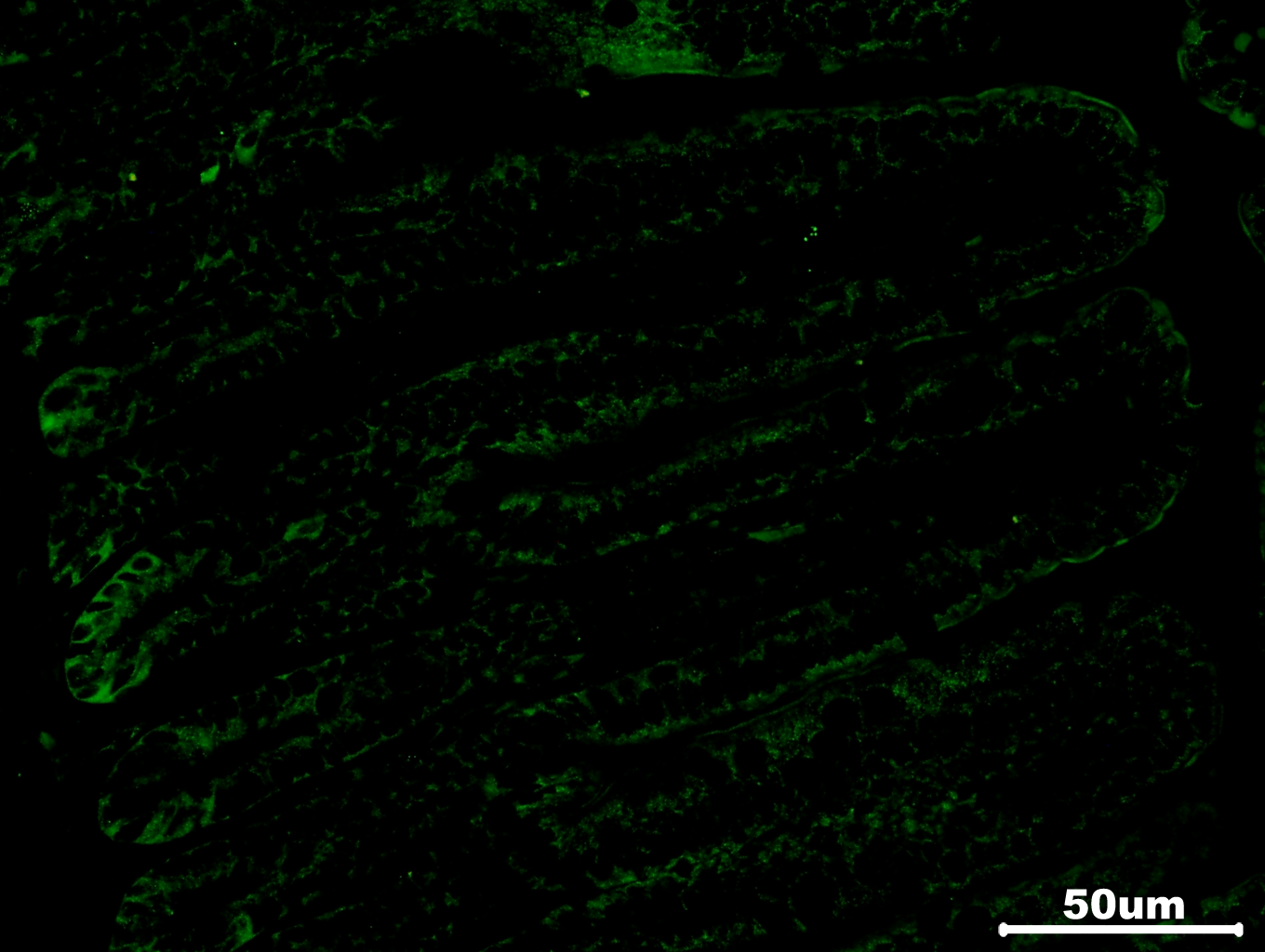

Supplement: Supplementary file 11 [file DataSheet_11.zip › F35-1-200-2-CD86.tif]

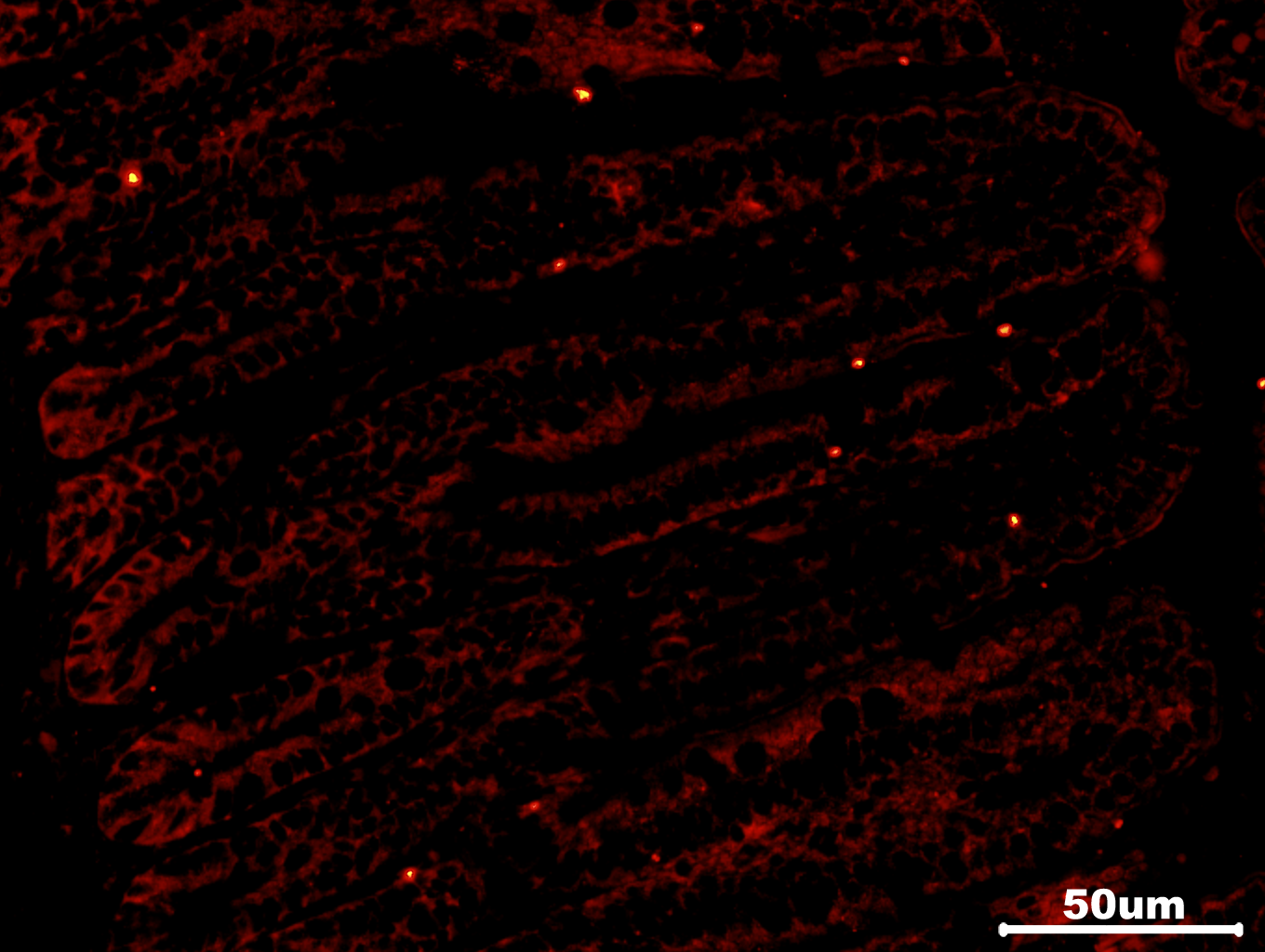

Supplement: Supplementary file 11 [file DataSheet_11.zip › F35-1-200-2-CD206.tif]

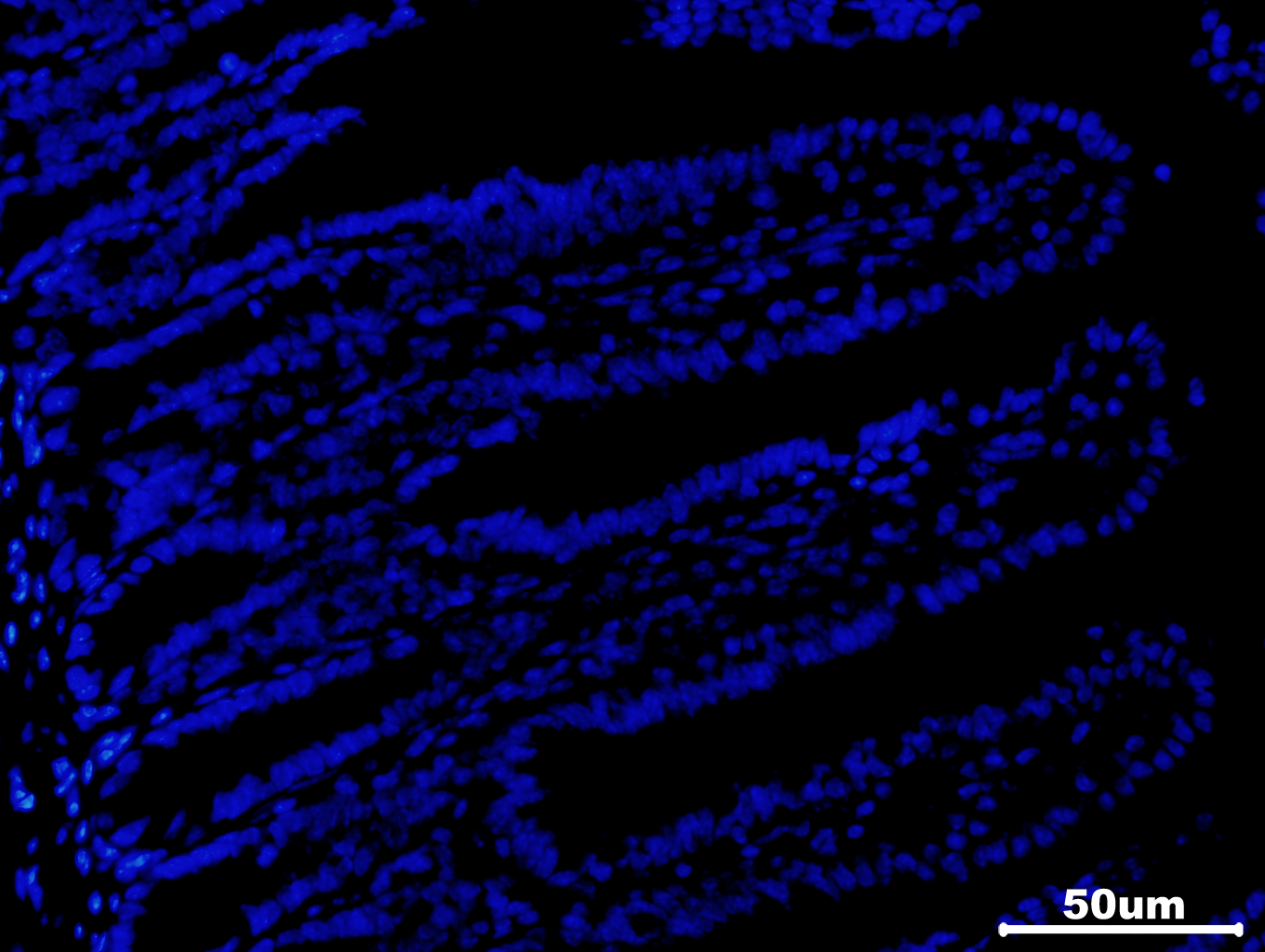

Supplement: Supplementary file 11 [file DataSheet_11.zip › F35-1-200-2-DAPI.tif]

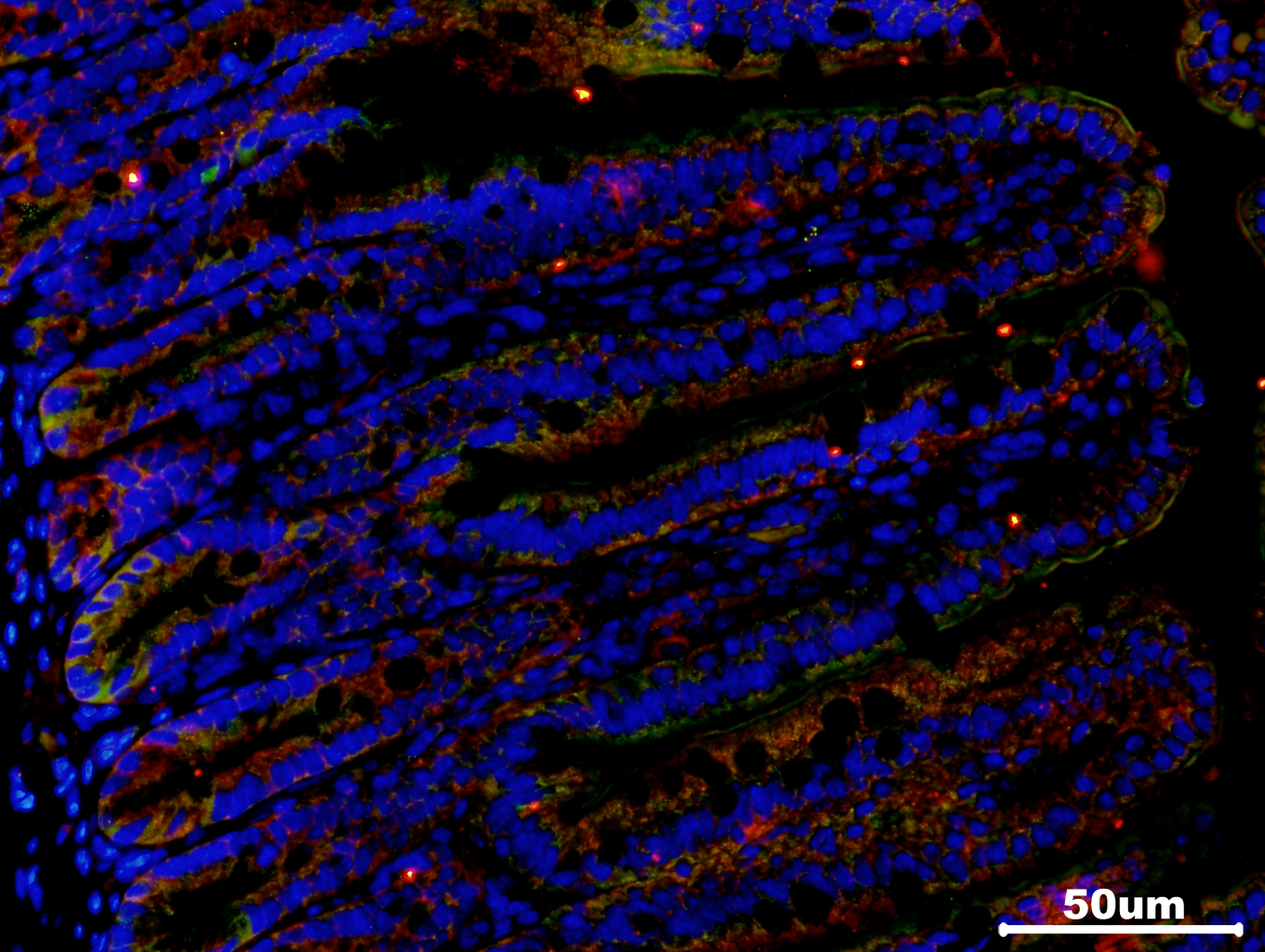

Supplement: Supplementary file 11 [file DataSheet_11.zip › F35-1-200-2-merge.tif]

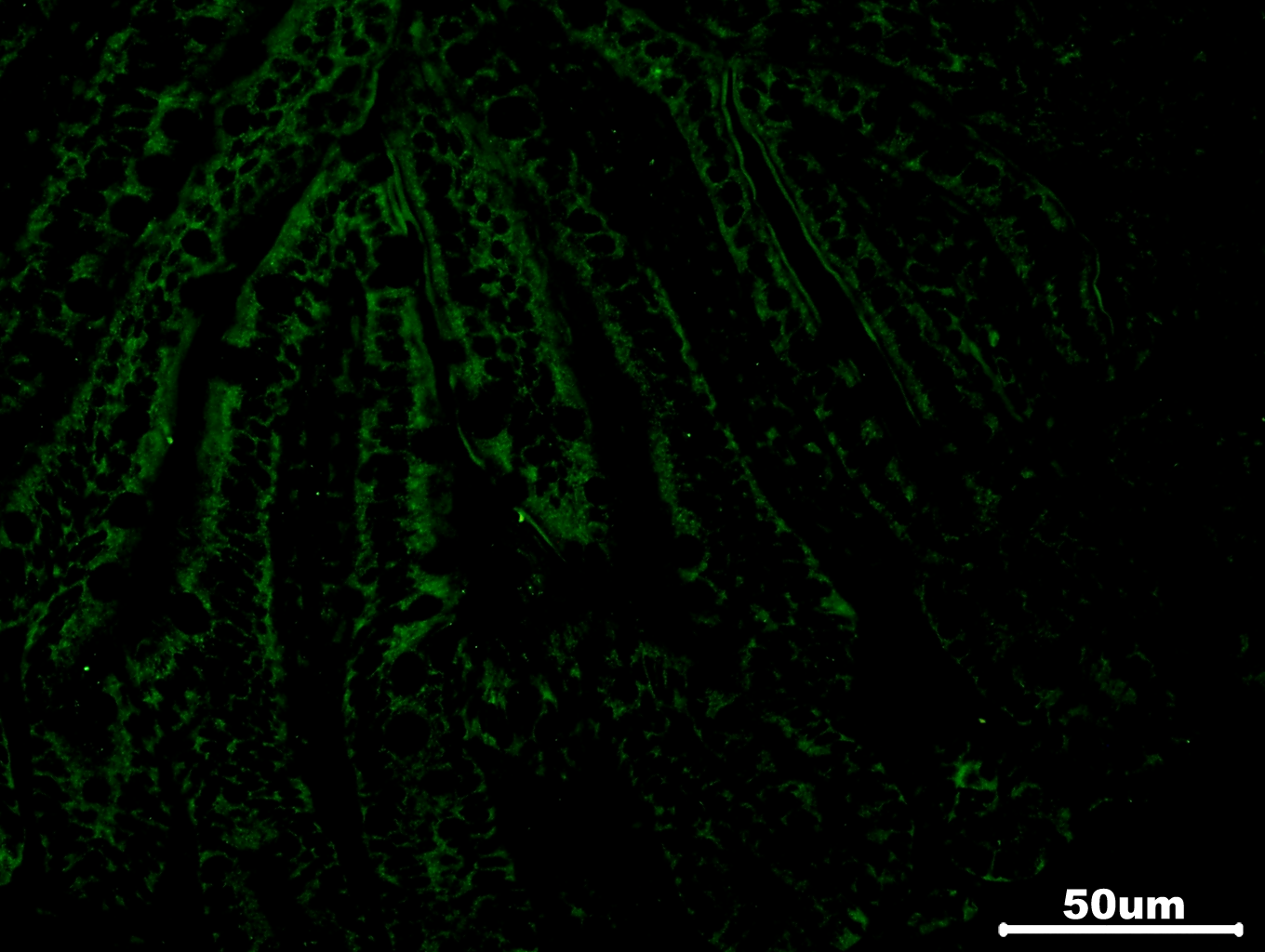

Supplement: Supplementary file 11 [file DataSheet_11.zip › F35-1-200-3-CD86.tif]

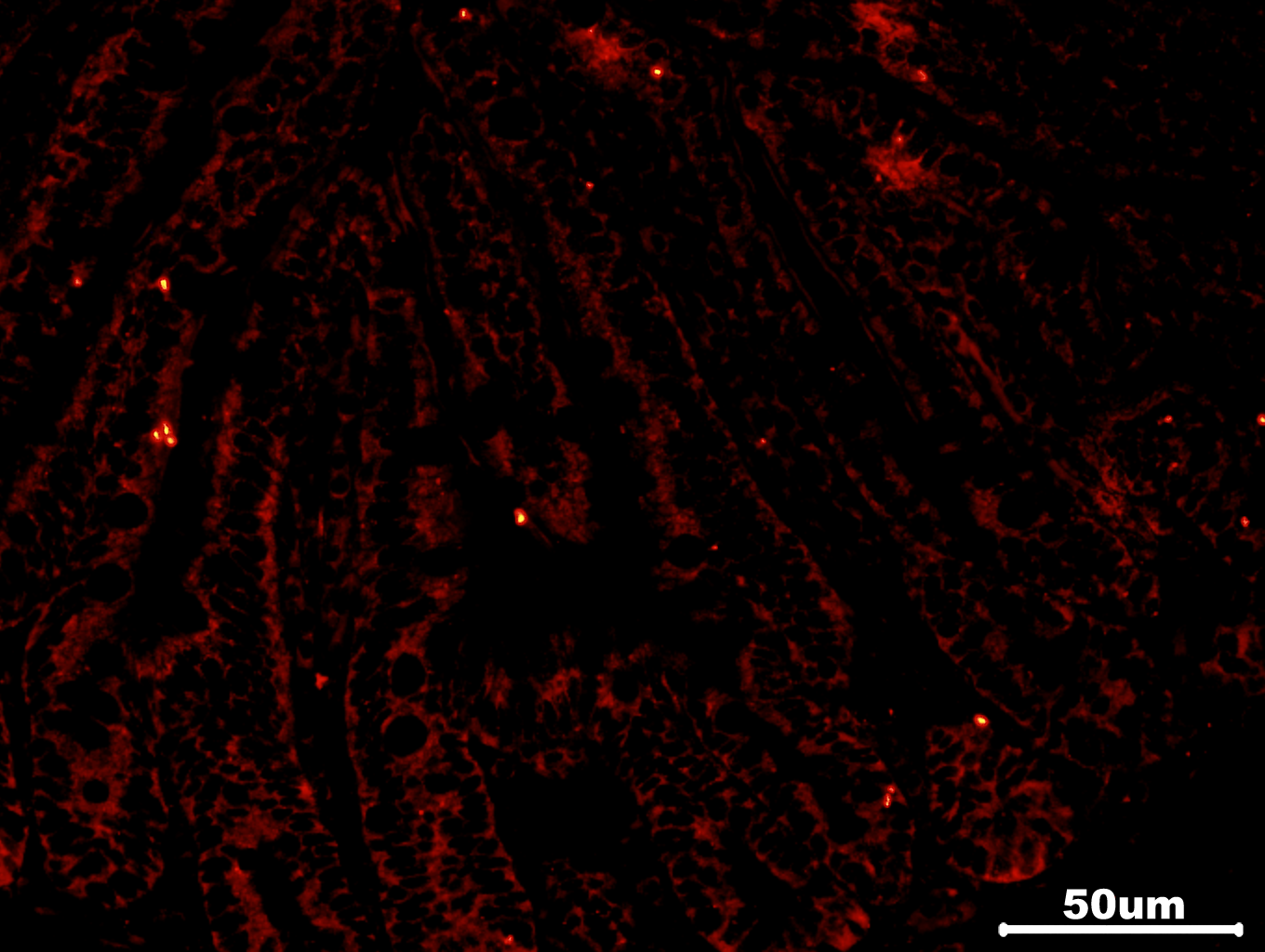

Supplement: Supplementary file 11 [file DataSheet_11.zip › F35-1-200-3-CD206.tif]

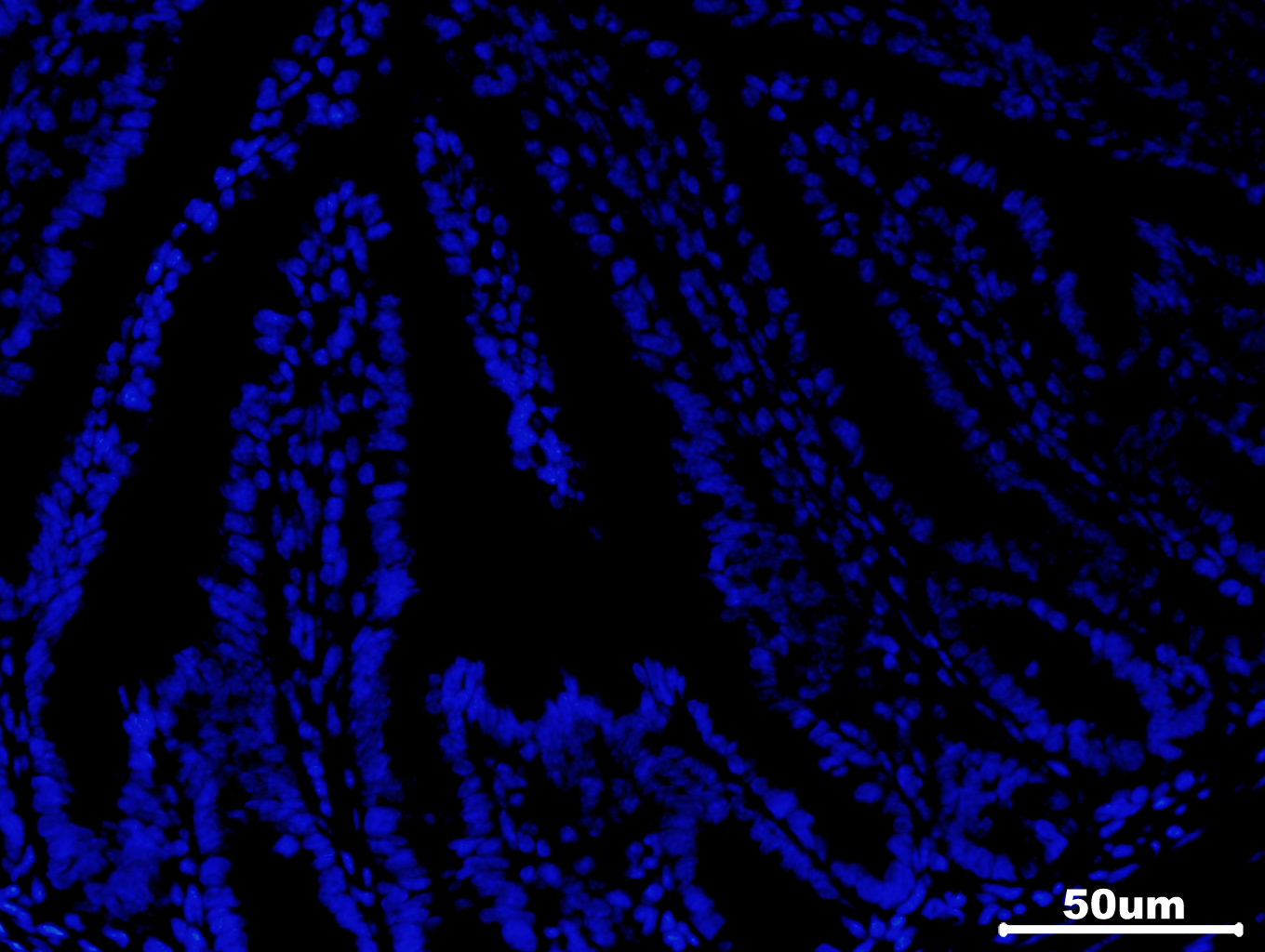

Supplement: Supplementary file 11 [file DataSheet_11.zip › F35-1-200-3-DAPI.tif]

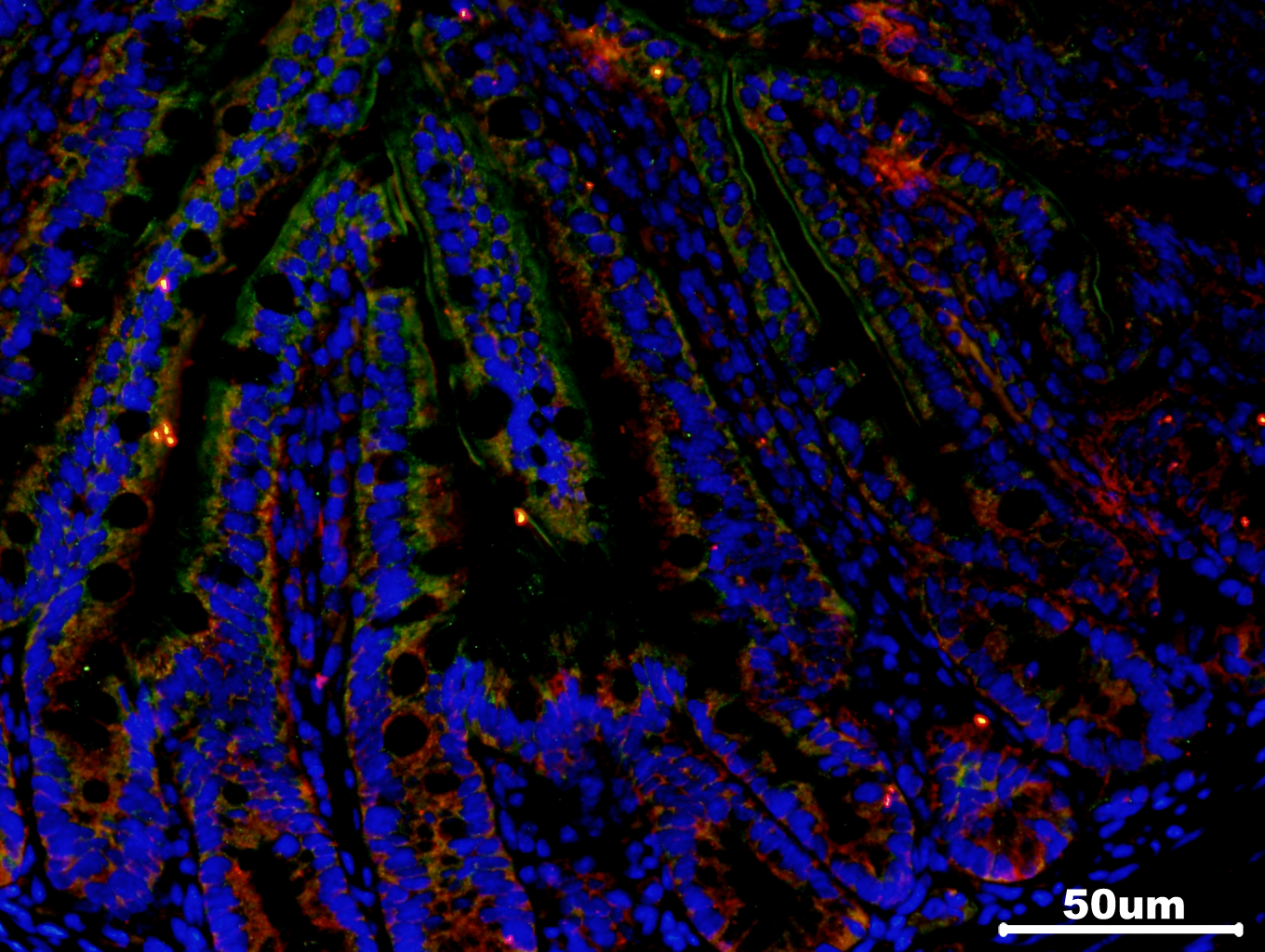

Supplement: Supplementary file 11 [file DataSheet_11.zip › F35-1-200-3-merge.tif]

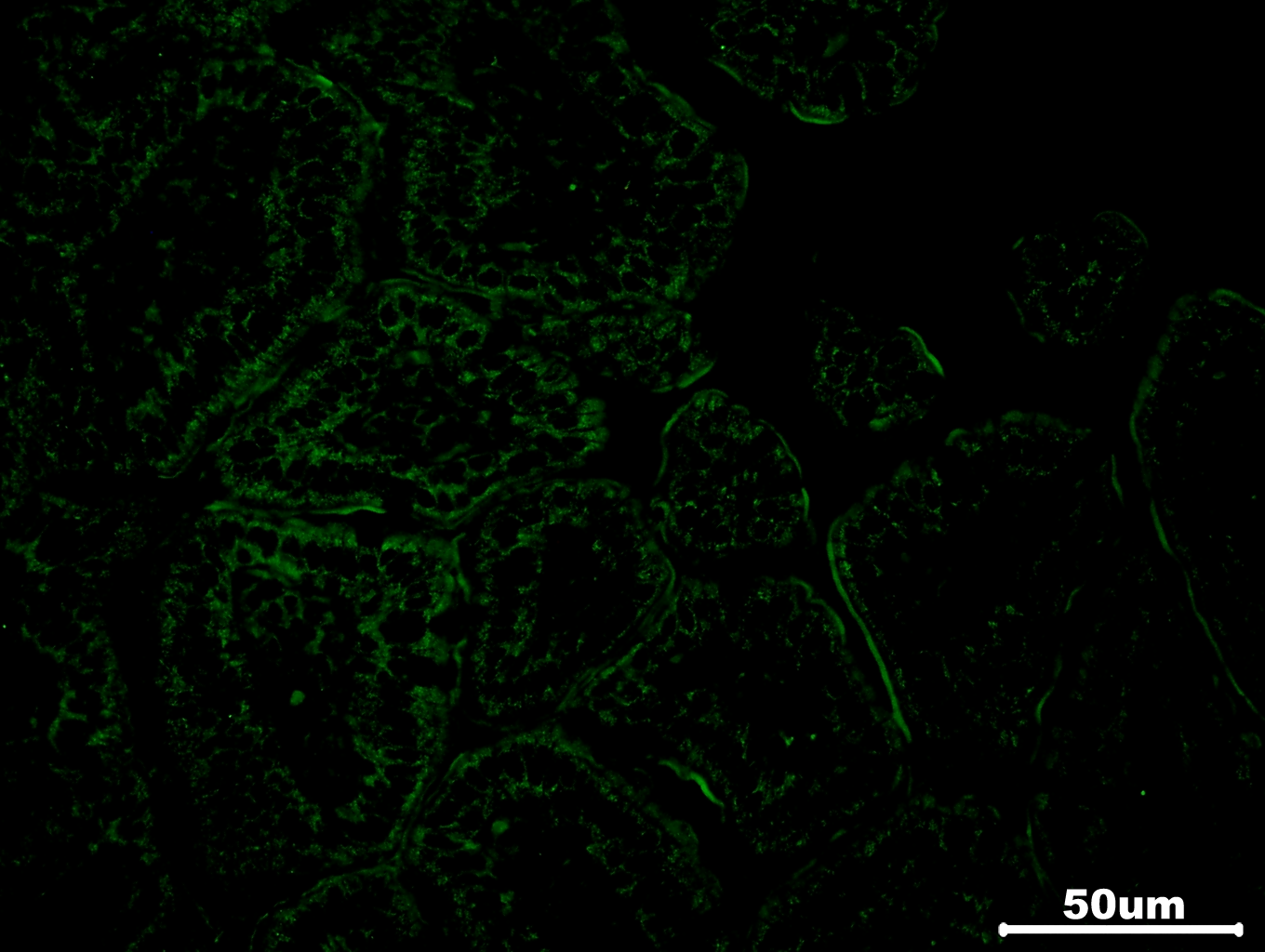

Supplement: Supplementary file 11 [file DataSheet_11.zip › F35-2-200-1-CD86.tif]

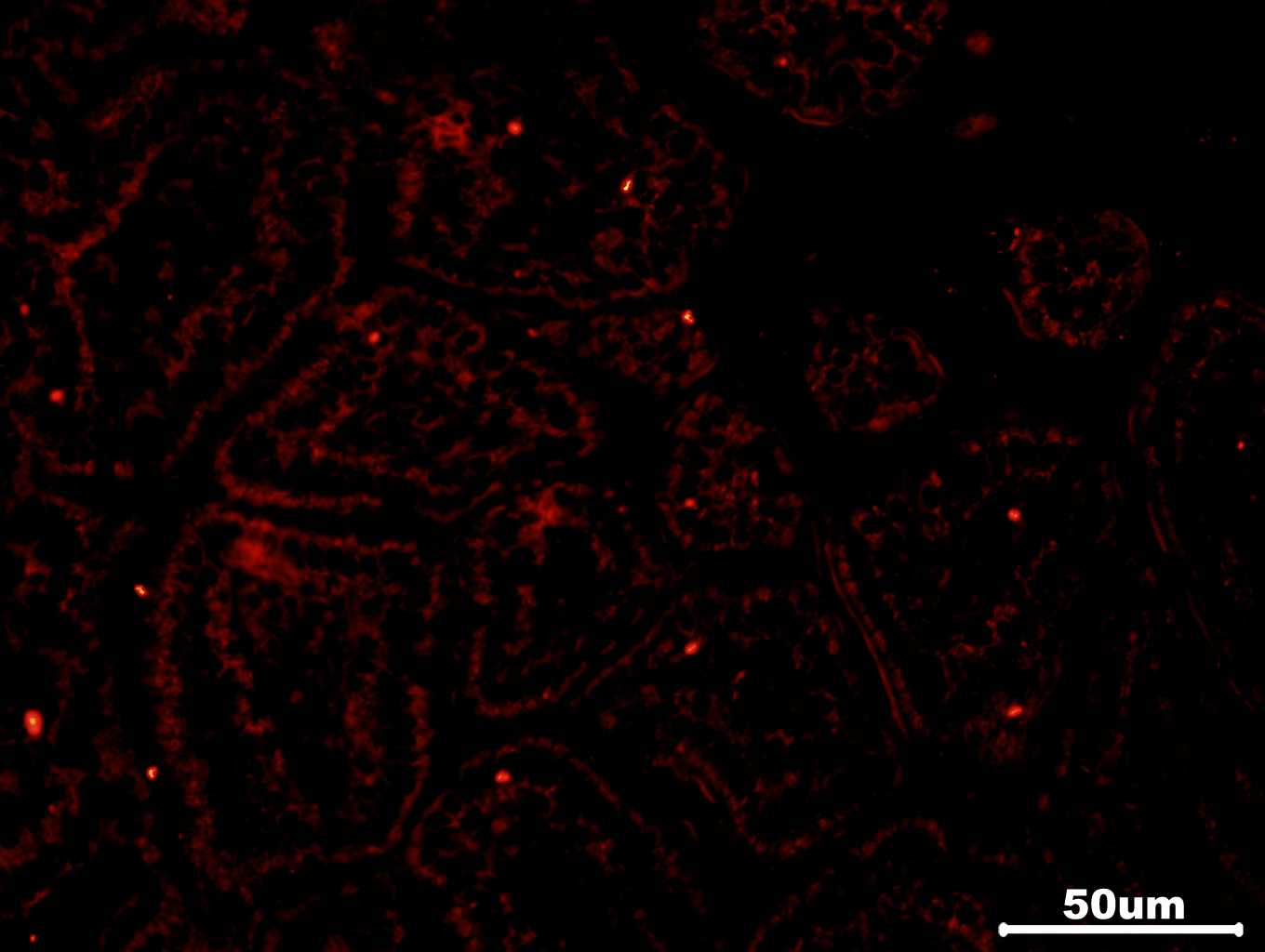

Supplement: Supplementary file 11 [file DataSheet_11.zip › F35-2-200-1-CD206.tif]

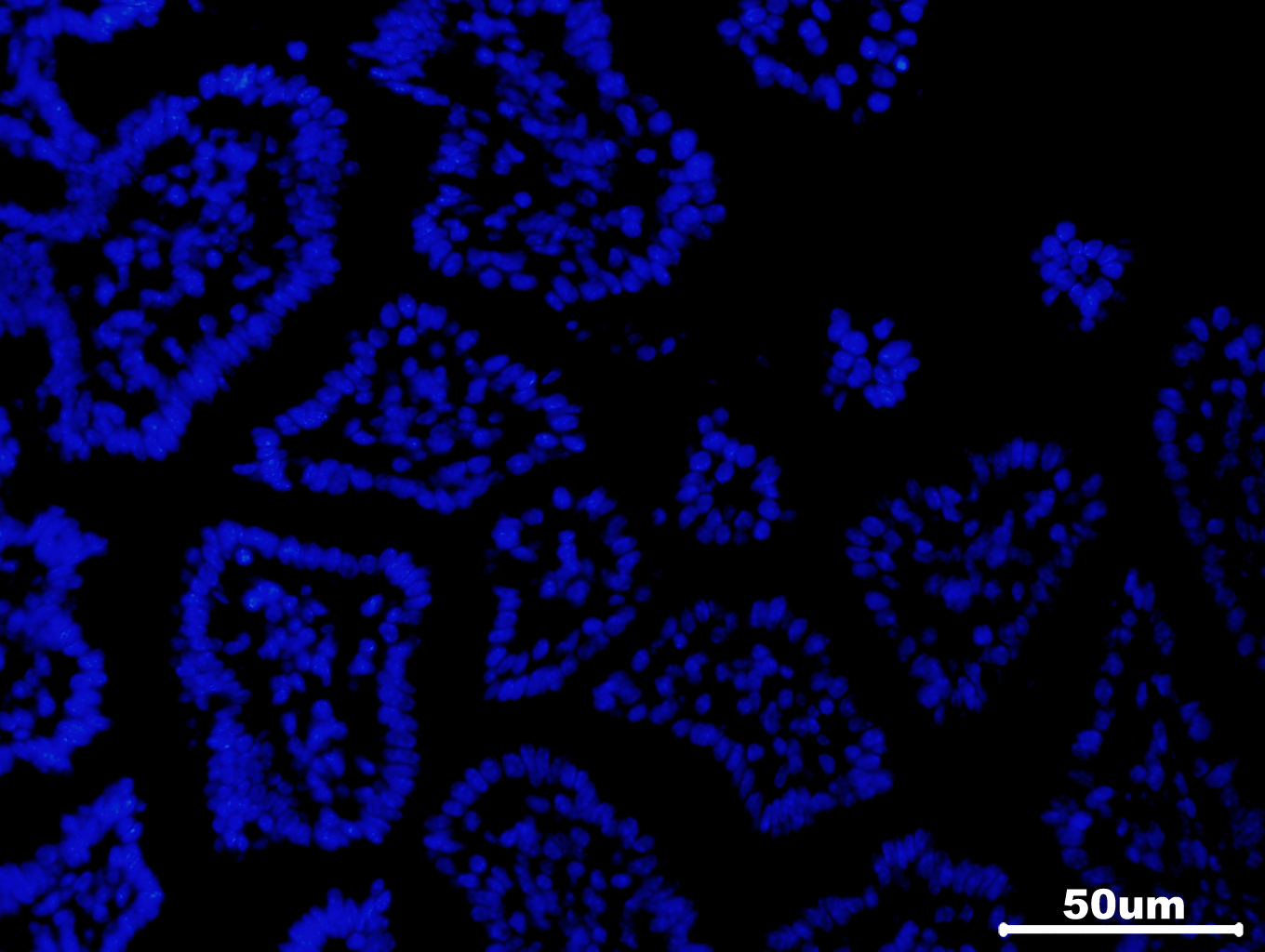

Supplement: Supplementary file 11 [file DataSheet_11.zip › F35-2-200-1-DAPI.tif]

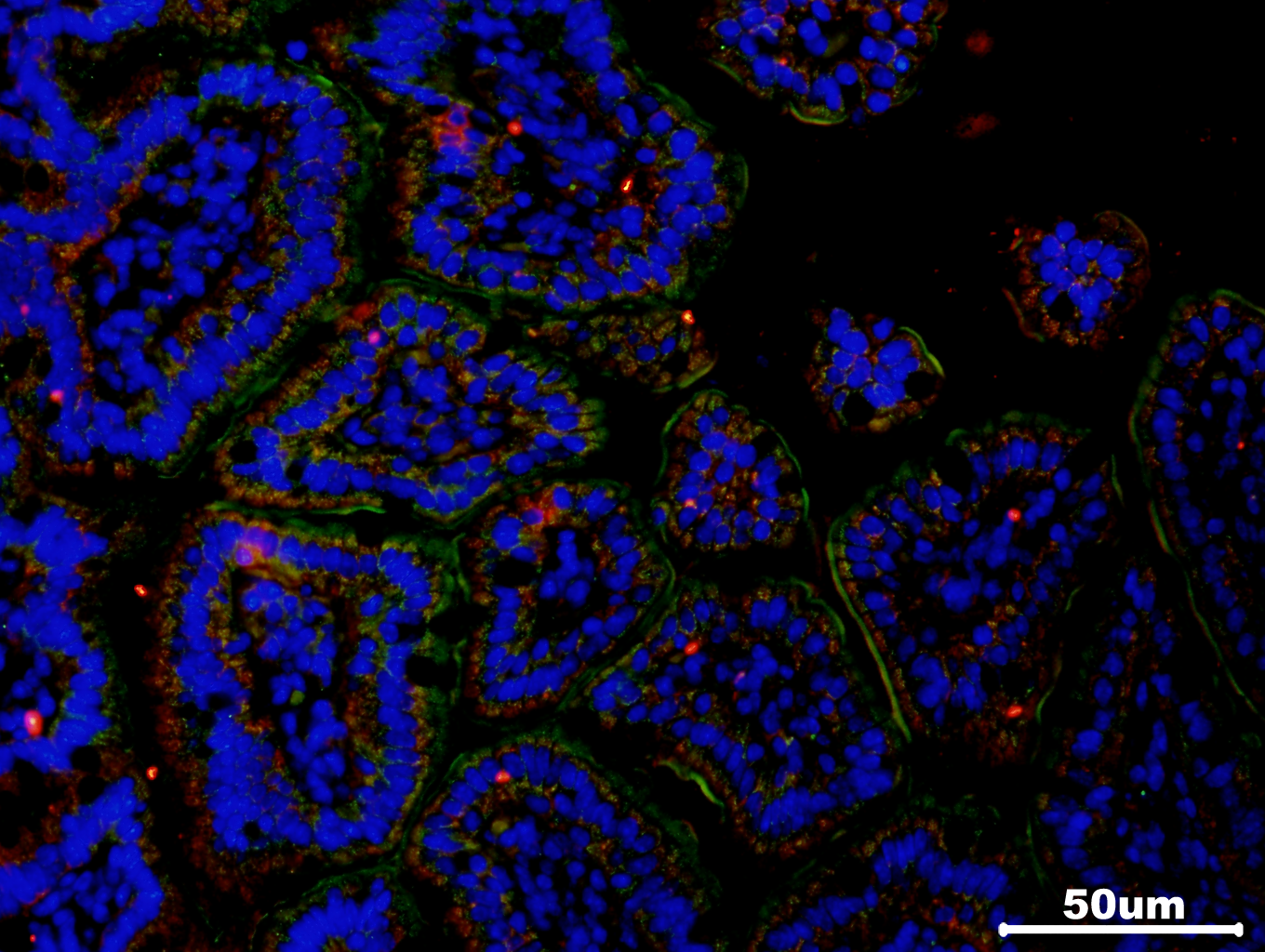

Supplement: Supplementary file 11 [file DataSheet_11.zip › F35-2-200-1-merge.tif]

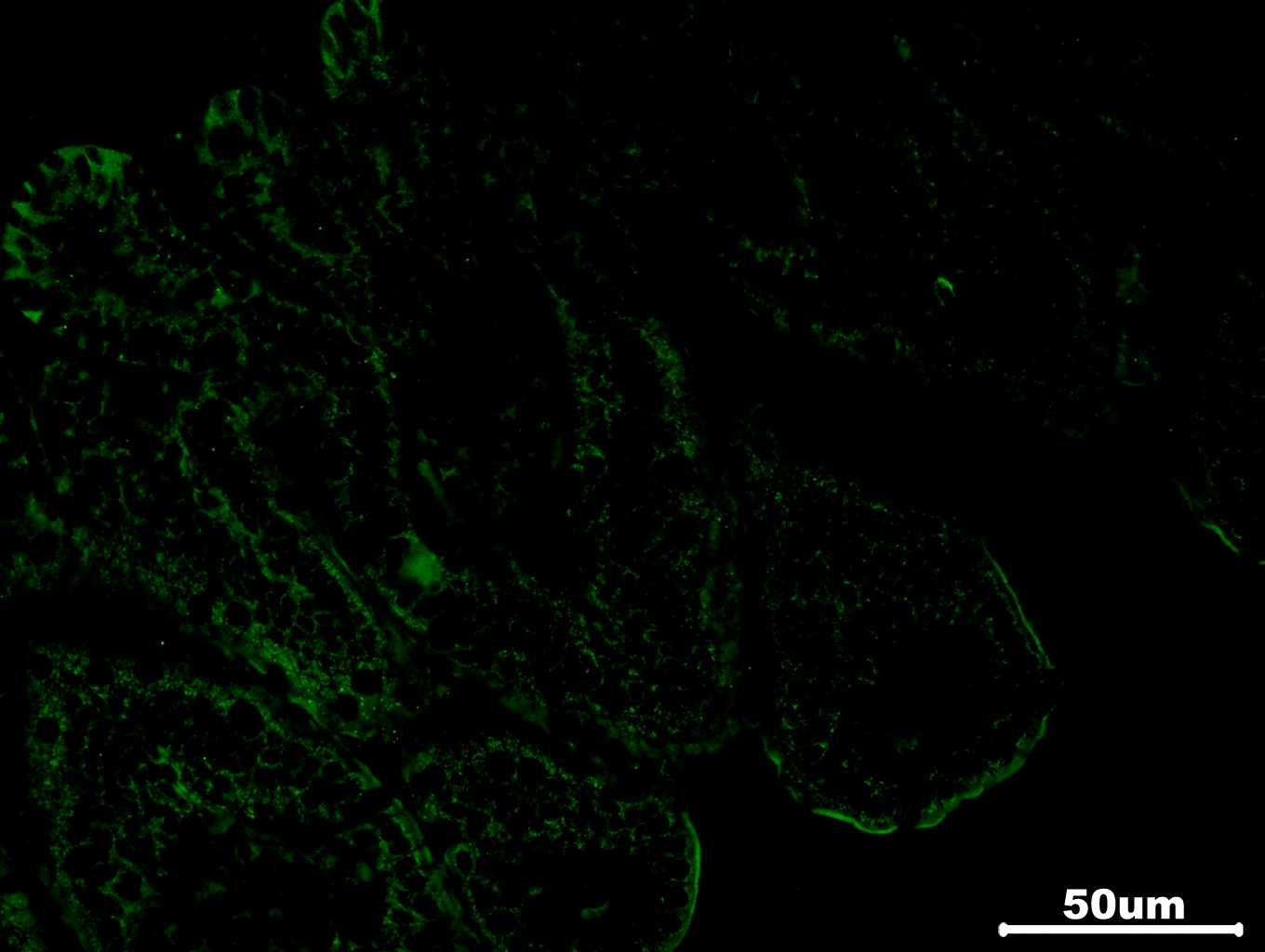

Supplement: Supplementary file 11 [file DataSheet_11.zip › F35-2-200-2-CD86.tif]

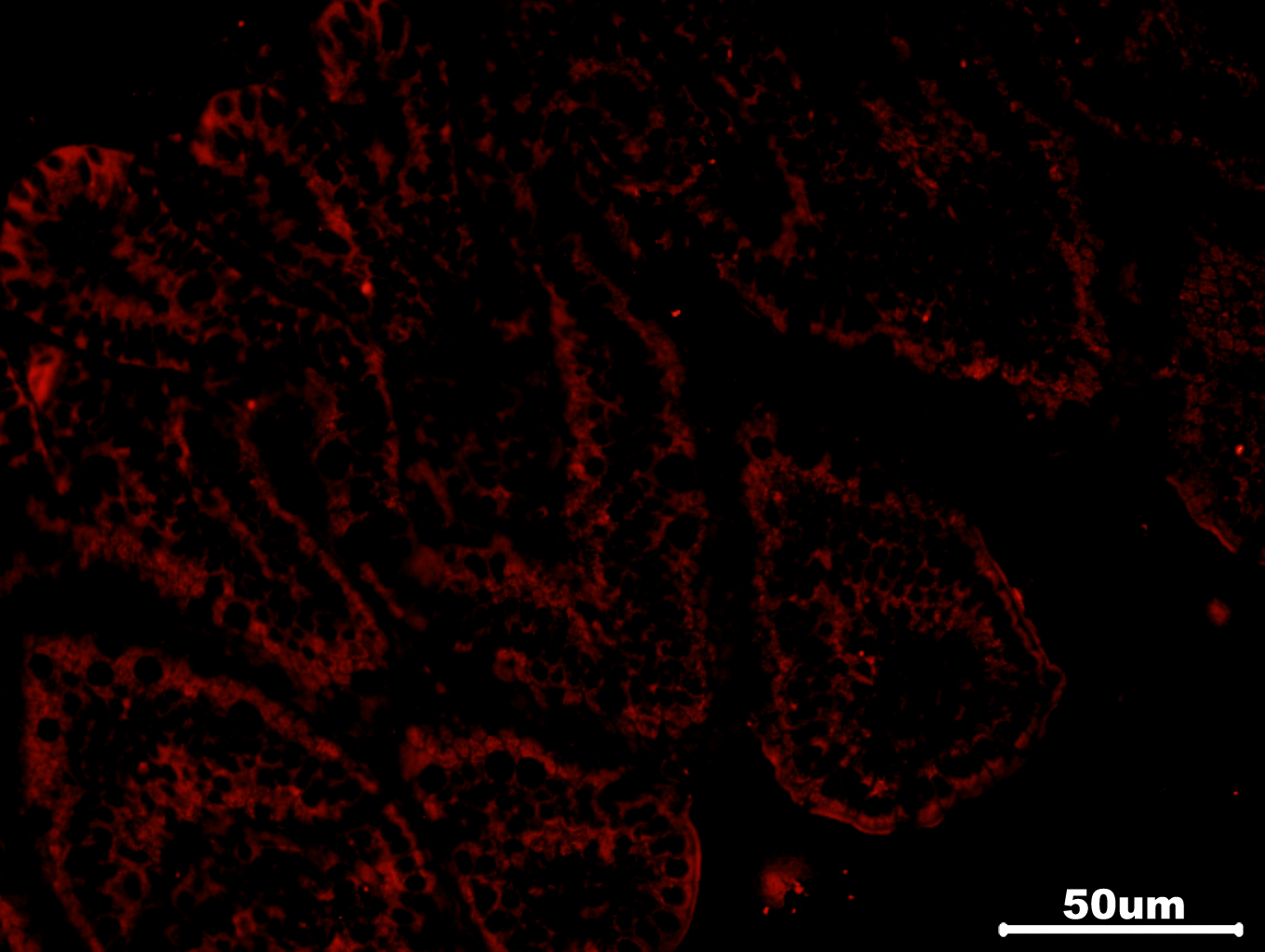

Supplement: Supplementary file 11 [file DataSheet_11.zip › F35-2-200-2-CD206.tif]

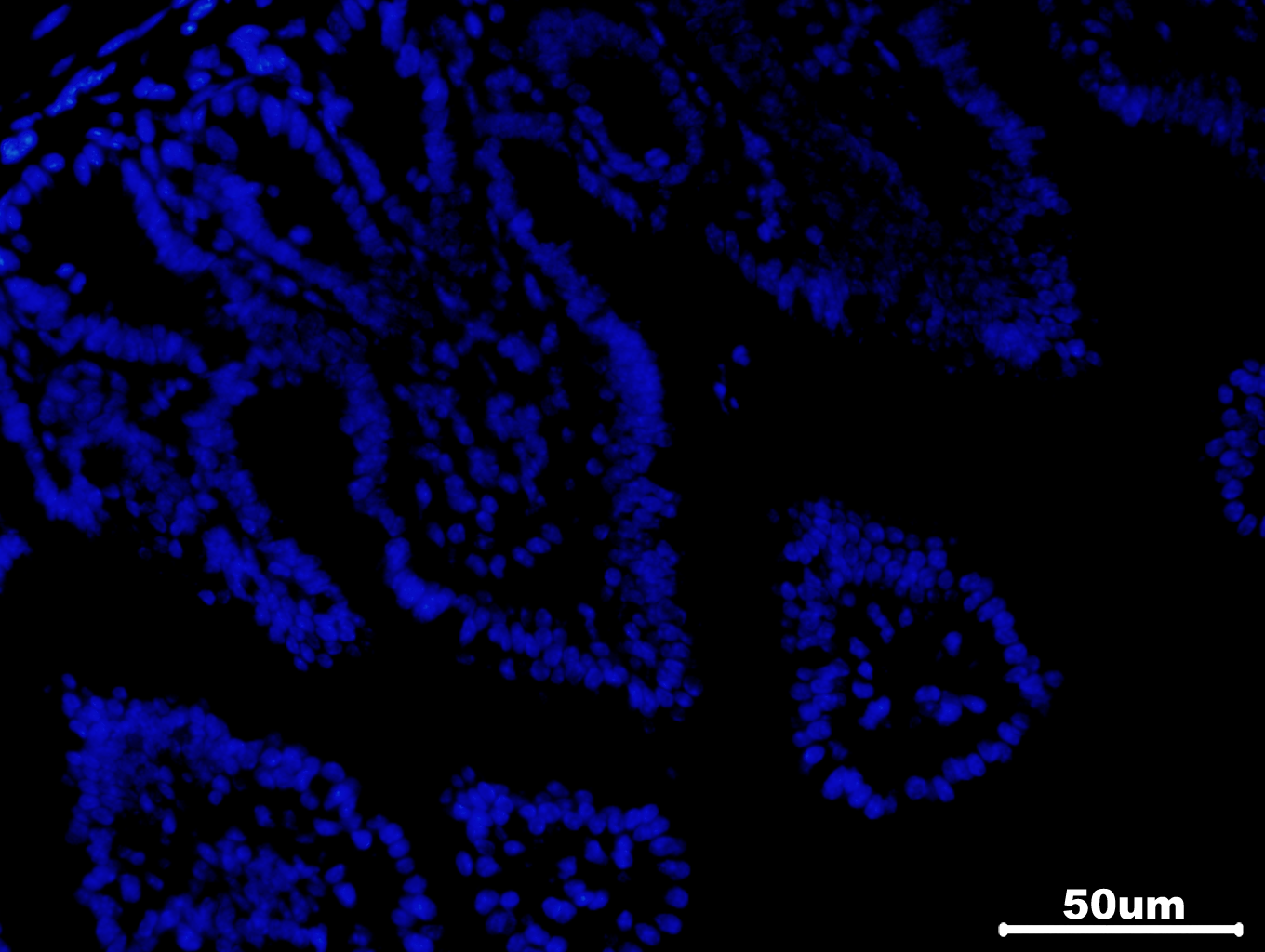

Supplement: Supplementary file 11 [file DataSheet_11.zip › F35-2-200-2-DAPI.tif]

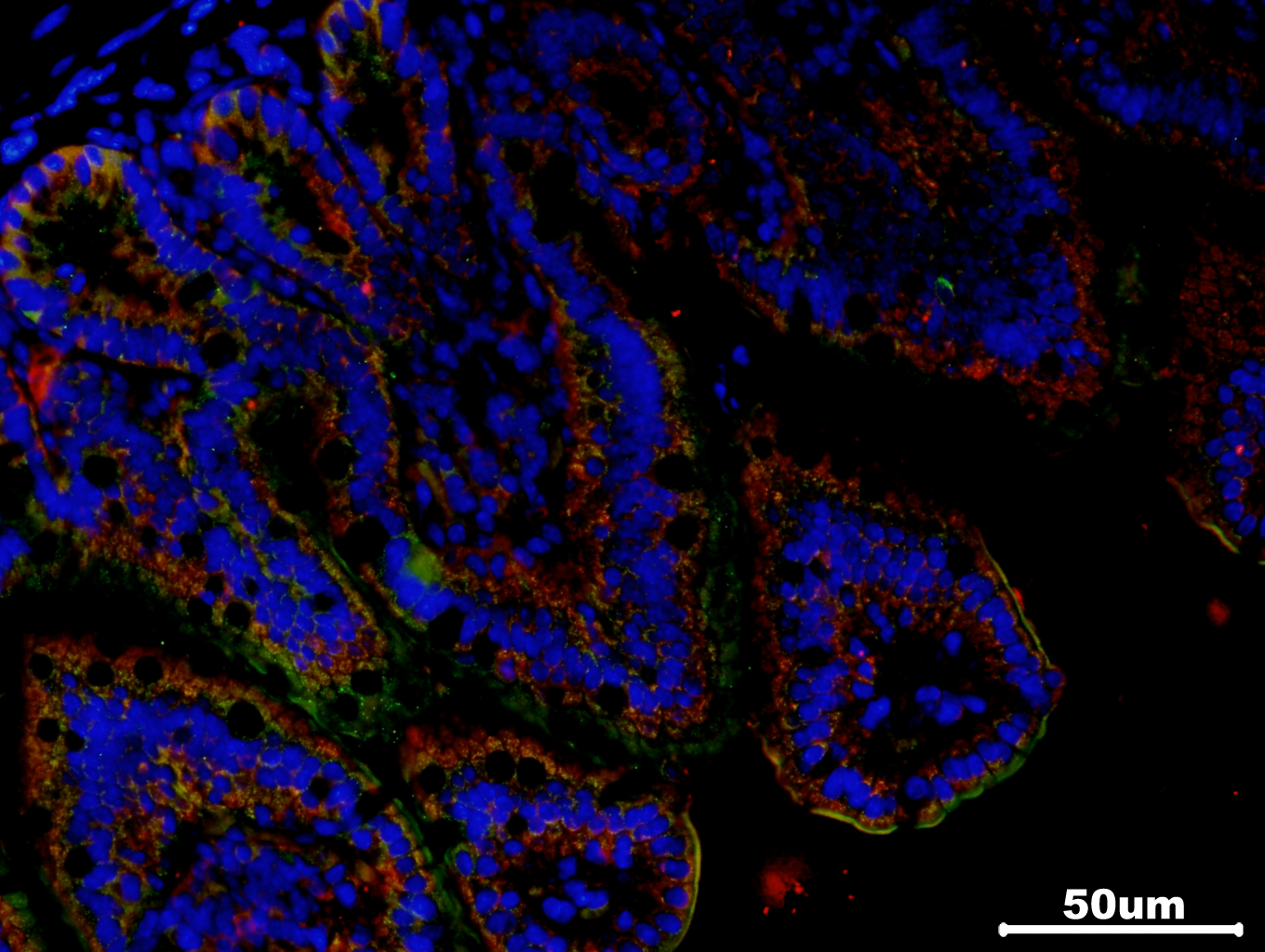

Supplement: Supplementary file 11 [file DataSheet_11.zip › F35-2-200-2-merge.tif]

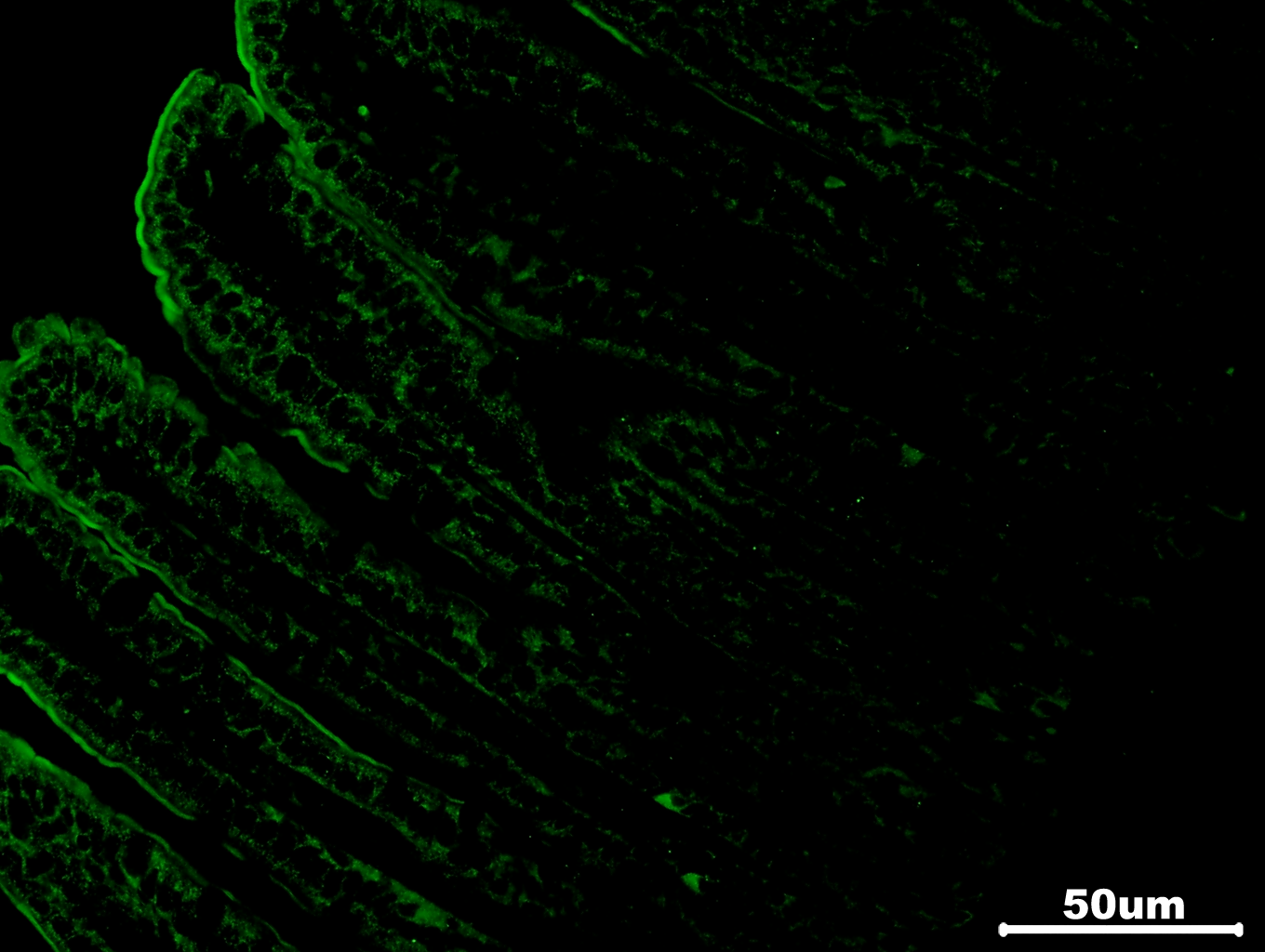

Supplement: Supplementary file 11 [file DataSheet_11.zip › F35-2-200-3-CD86.tif]

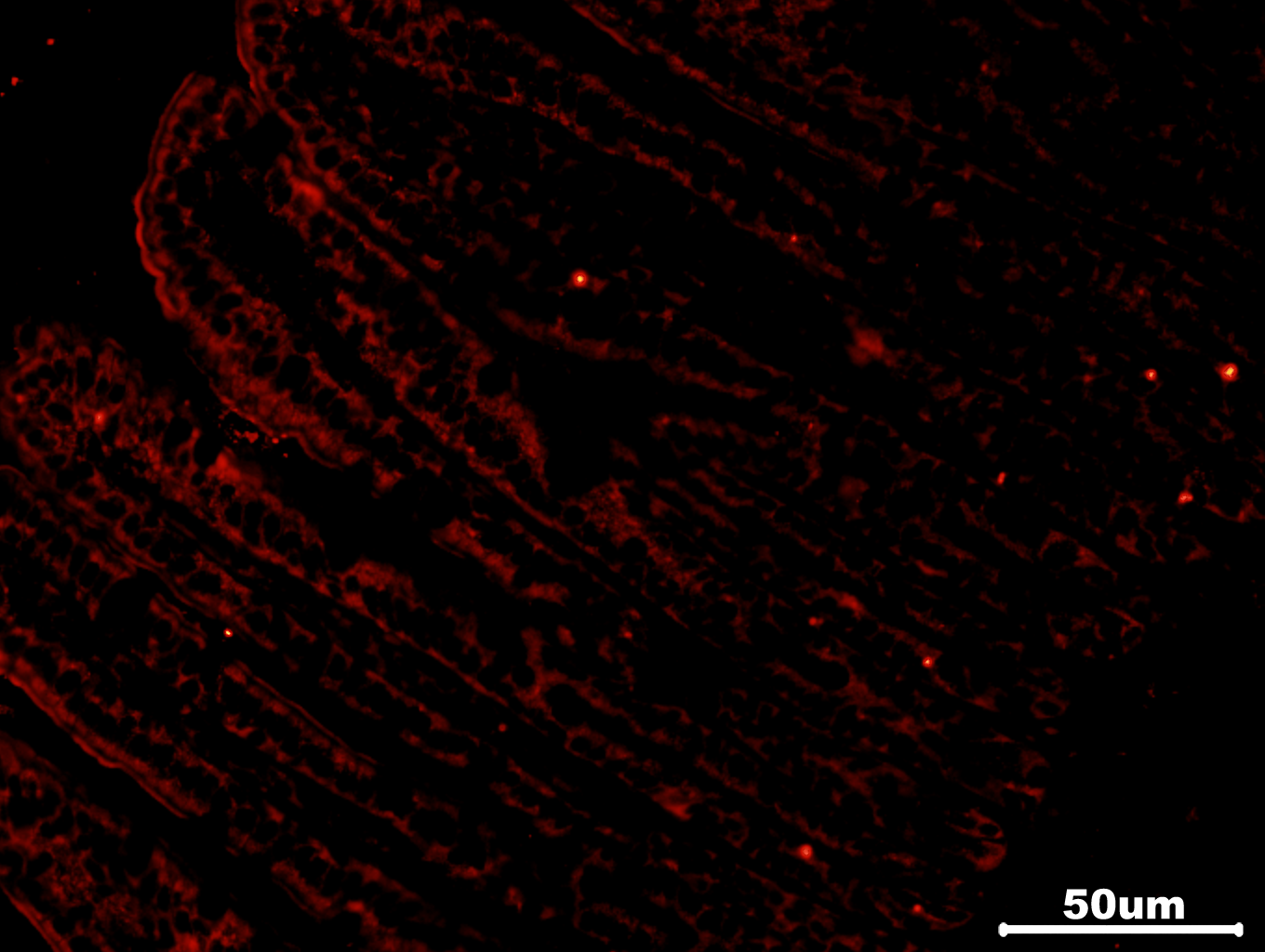

Supplement: Supplementary file 11 [file DataSheet_11.zip › F35-2-200-3-CD206.tif]

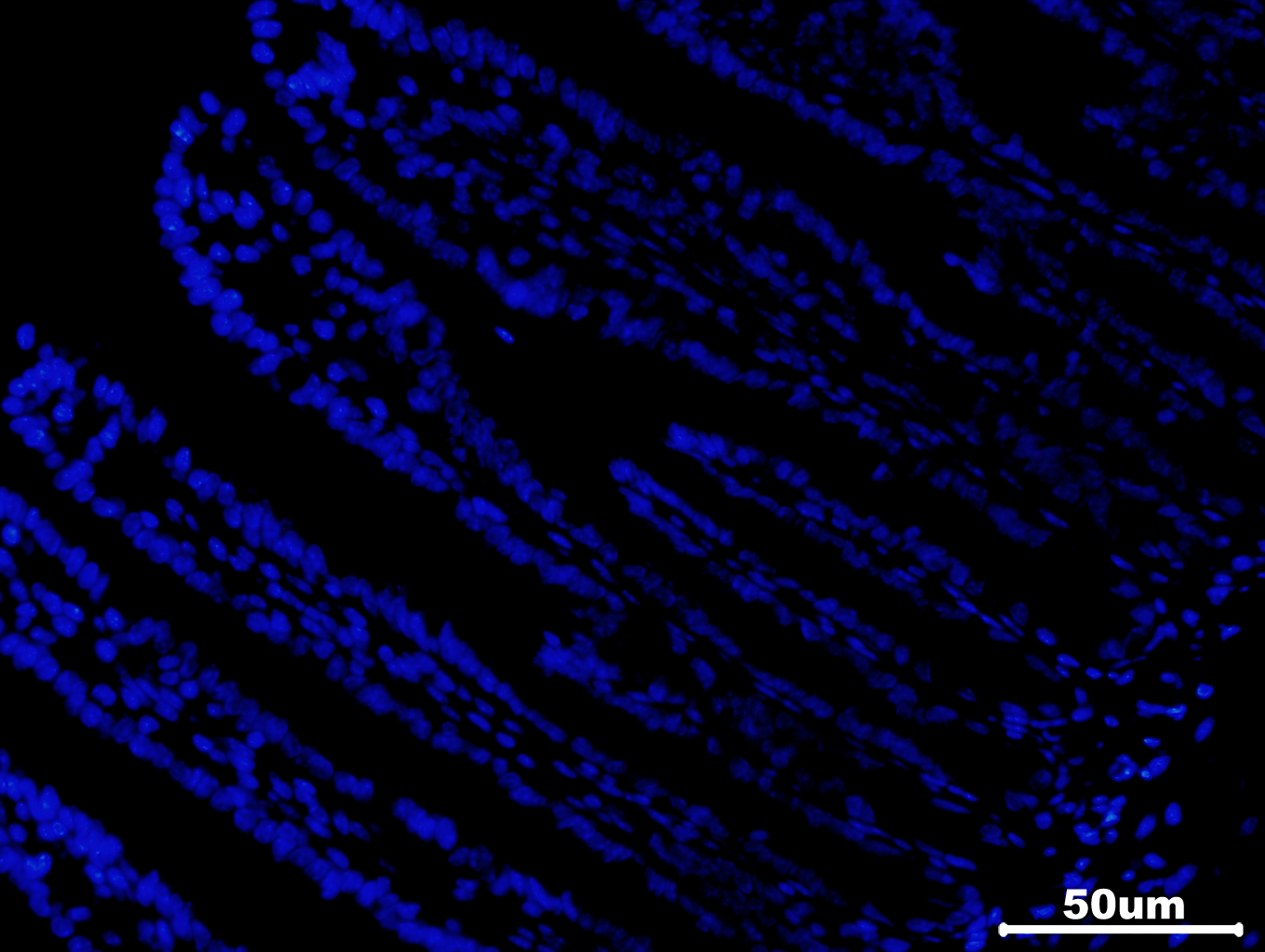

Supplement: Supplementary file 11 [file DataSheet_11.zip › F35-2-200-3-DAPI.tif]

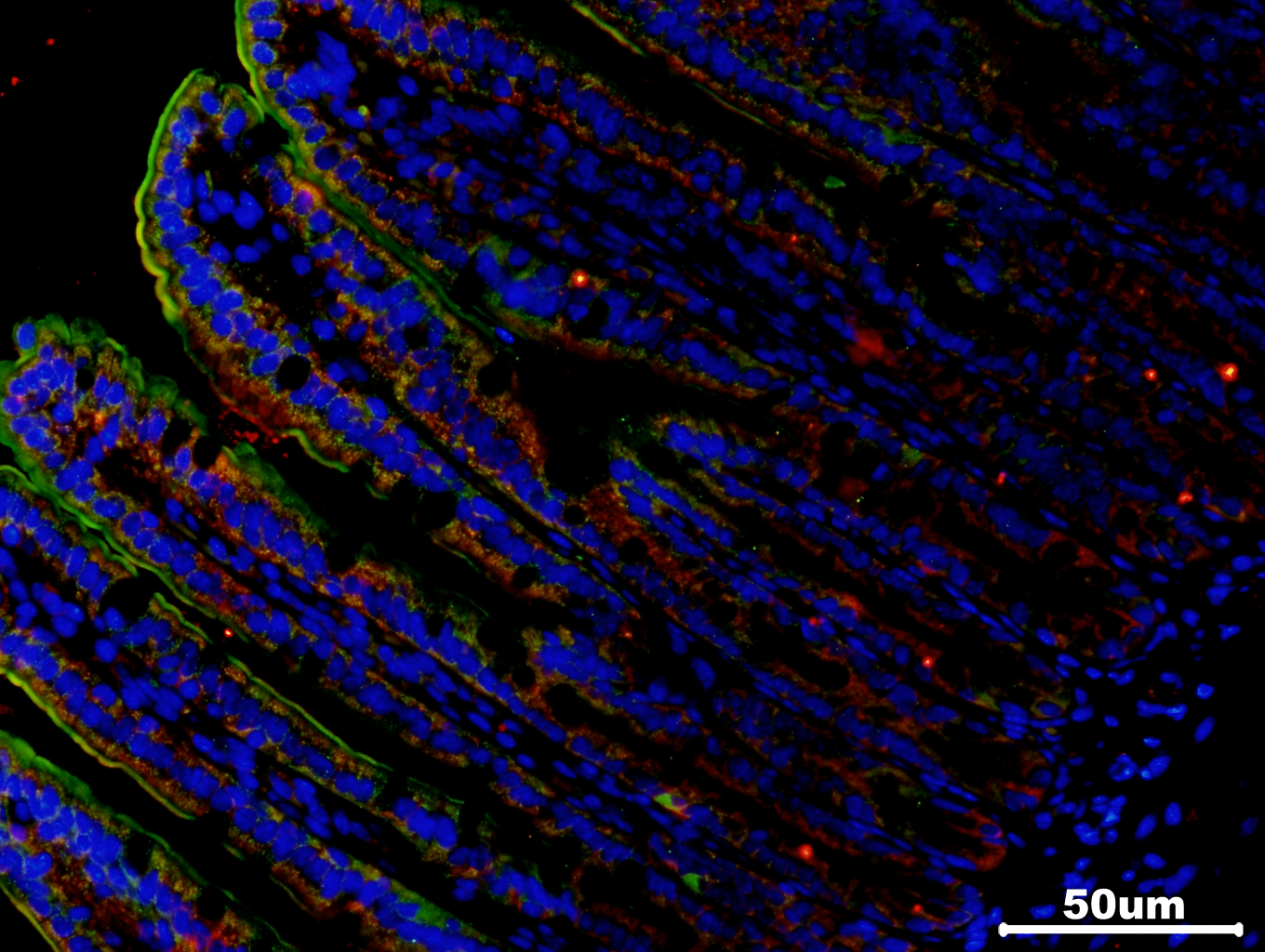

Supplement: Supplementary file 11 [file DataSheet_11.zip › F35-2-200-3-merge.tif]

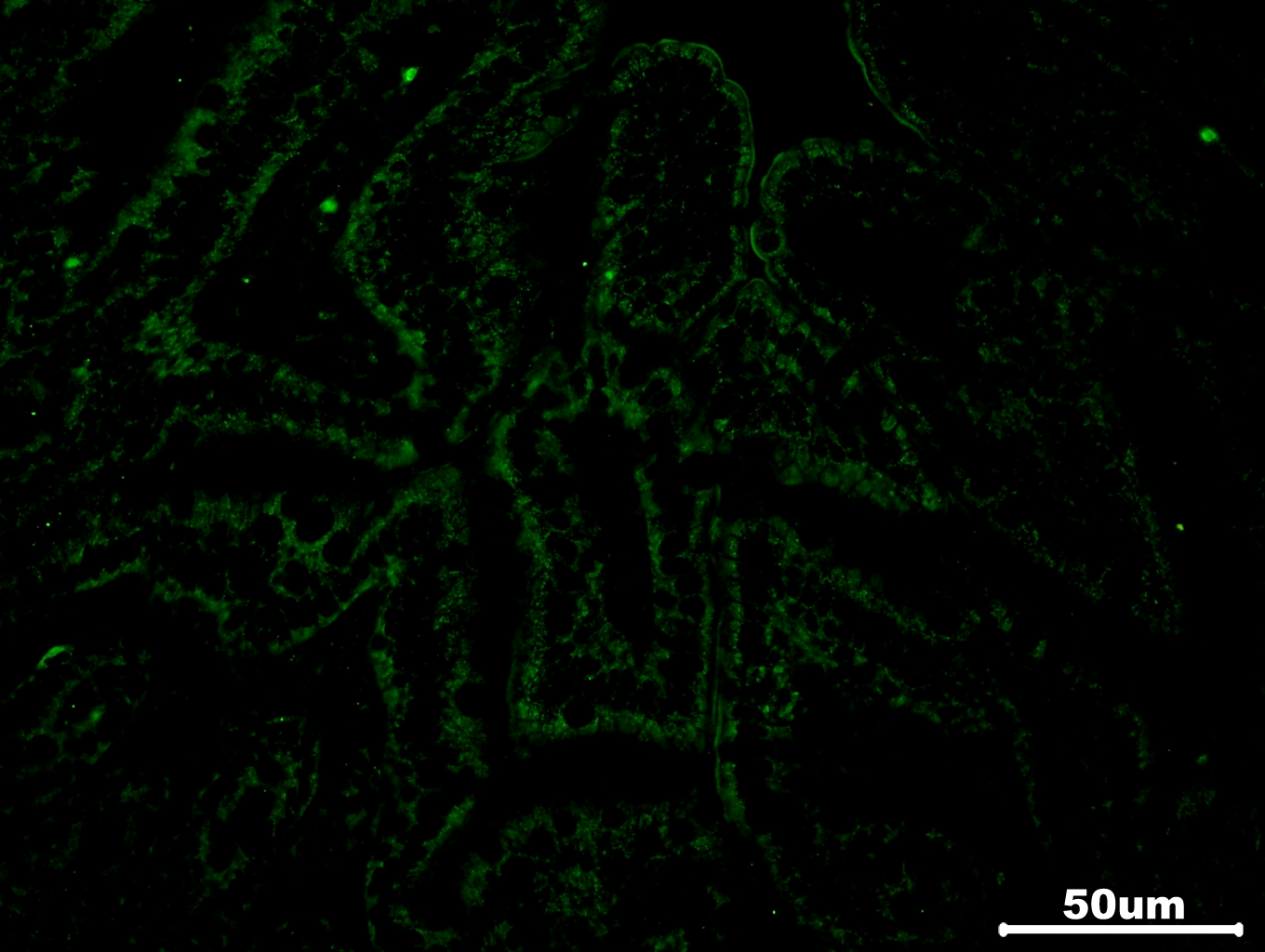

Supplement: Supplementary file 11 [file DataSheet_11.zip › F36-1-200-1-CD86.tif]

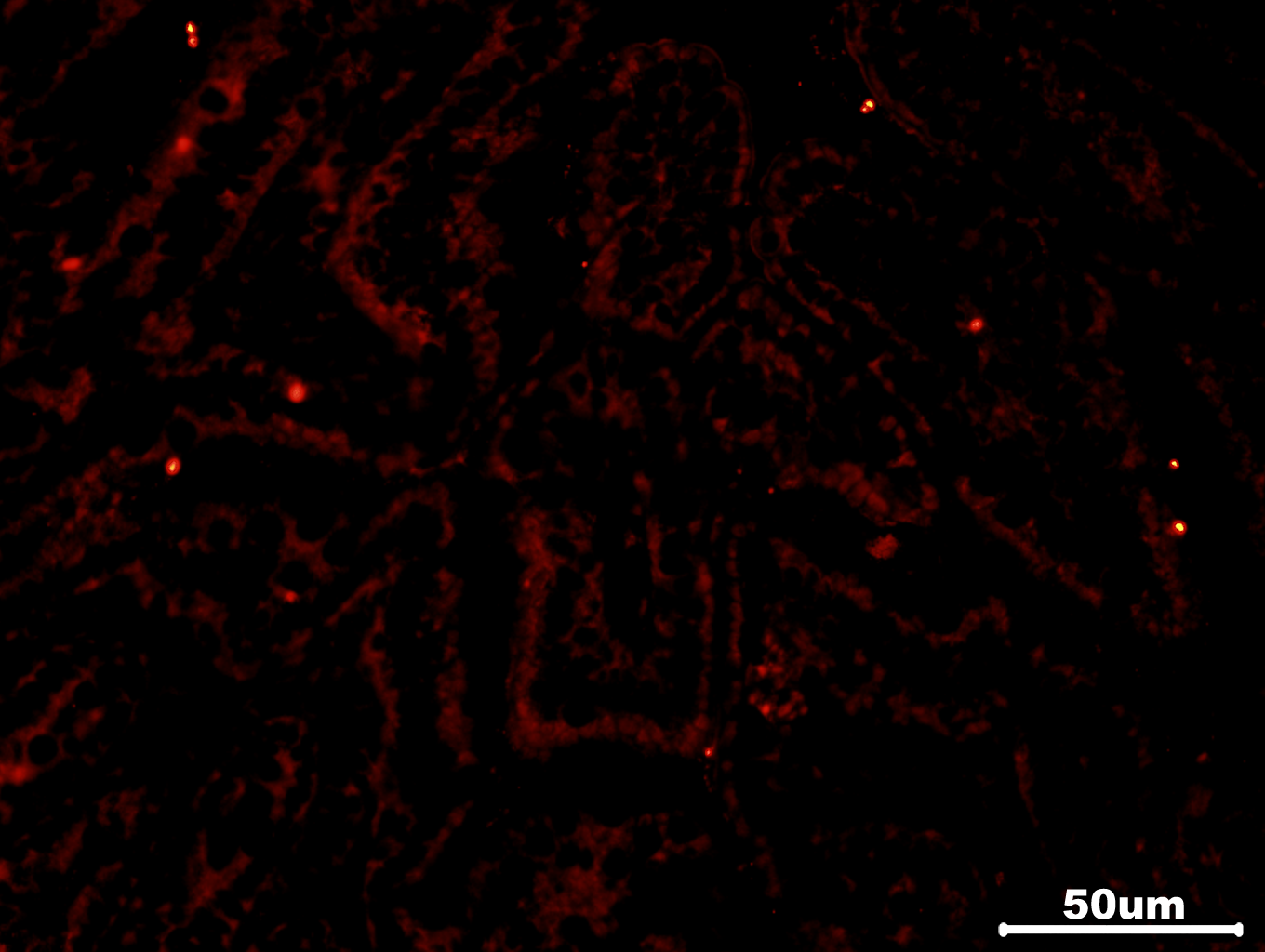

Supplement: Supplementary file 11 [file DataSheet_11.zip › F36-1-200-1-CD206.tif]

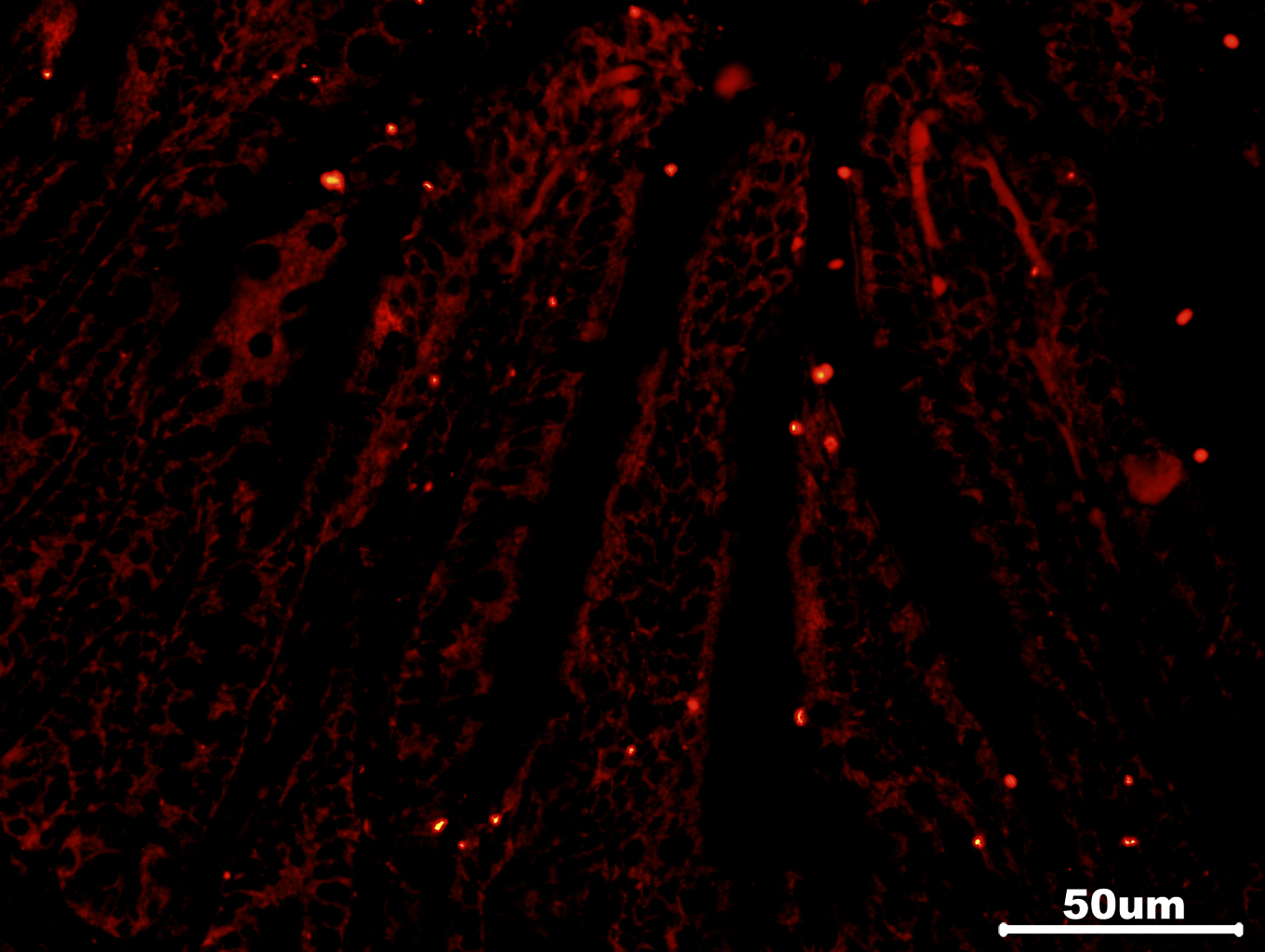

Supplement: Supplementary file 12 [file DataSheet_12.zip › D29-1-200-1-CD206.tif]

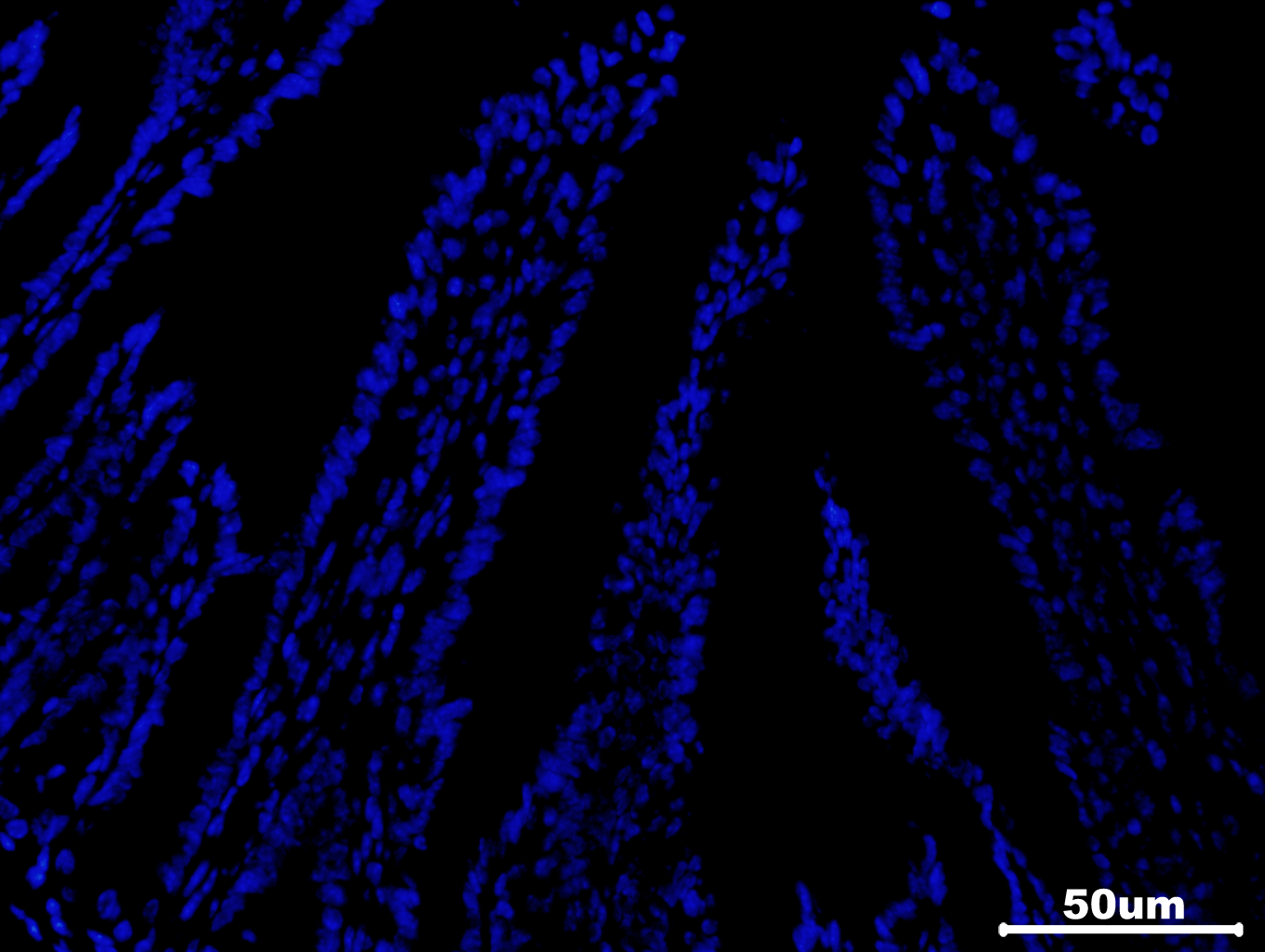

Supplement: Supplementary file 12 [file DataSheet_12.zip › D29-1-200-1-DAPI.tif]

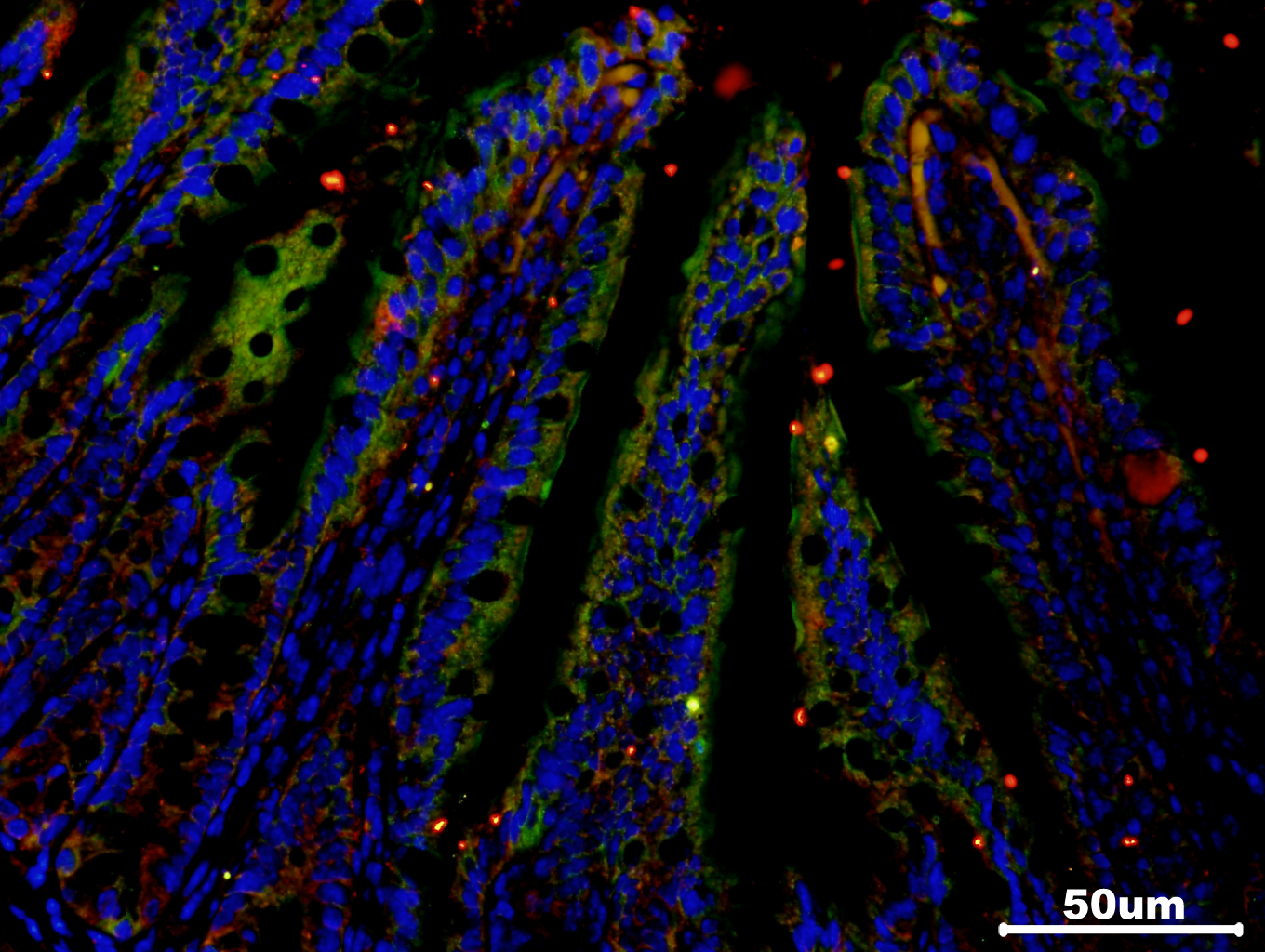

Supplement: Supplementary file 12 [file DataSheet_12.zip › D29-1-200-1-merge.tif]

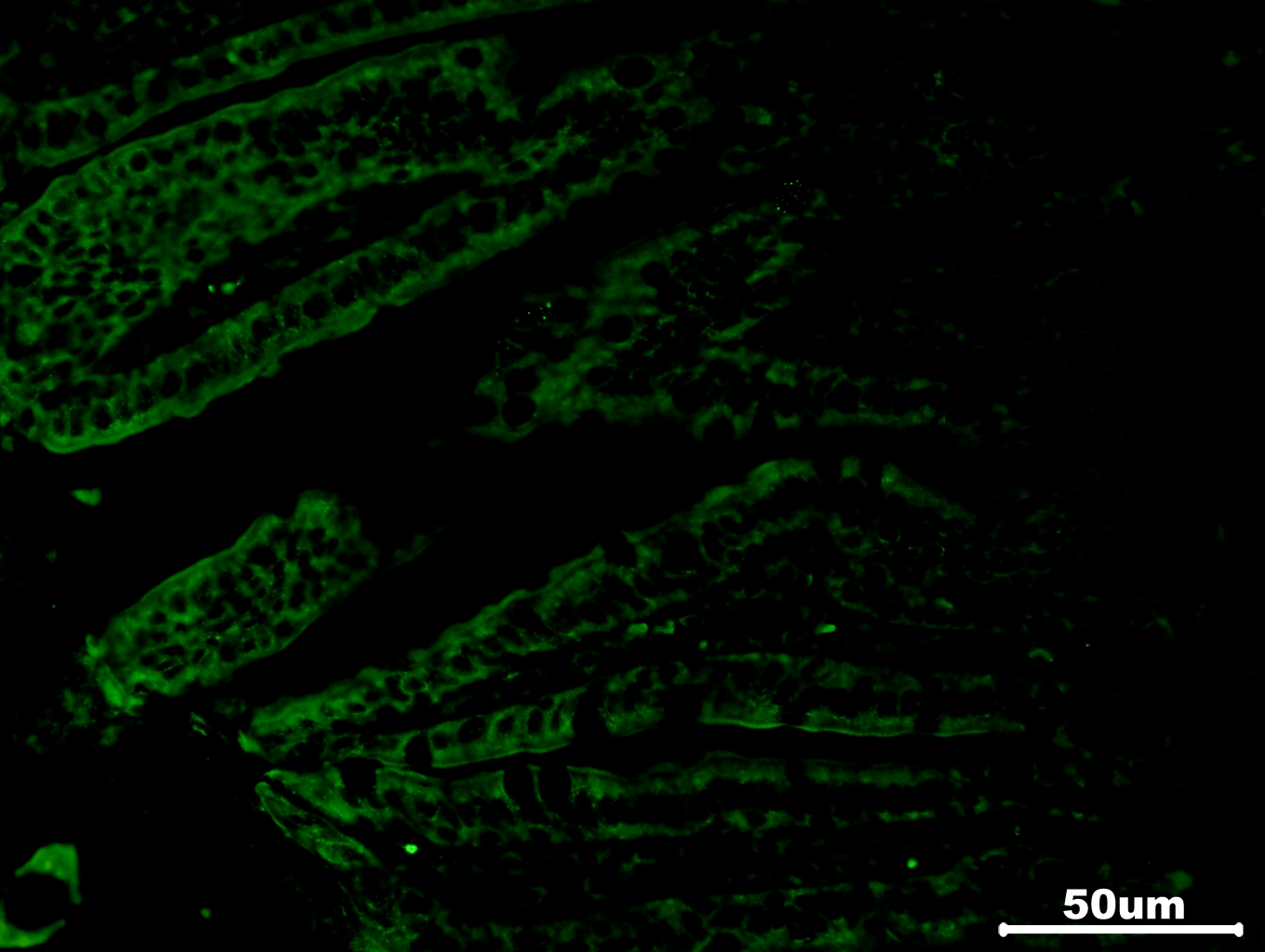

Supplement: Supplementary file 12 [file DataSheet_12.zip › D29-1-200-2-CD86.tif]

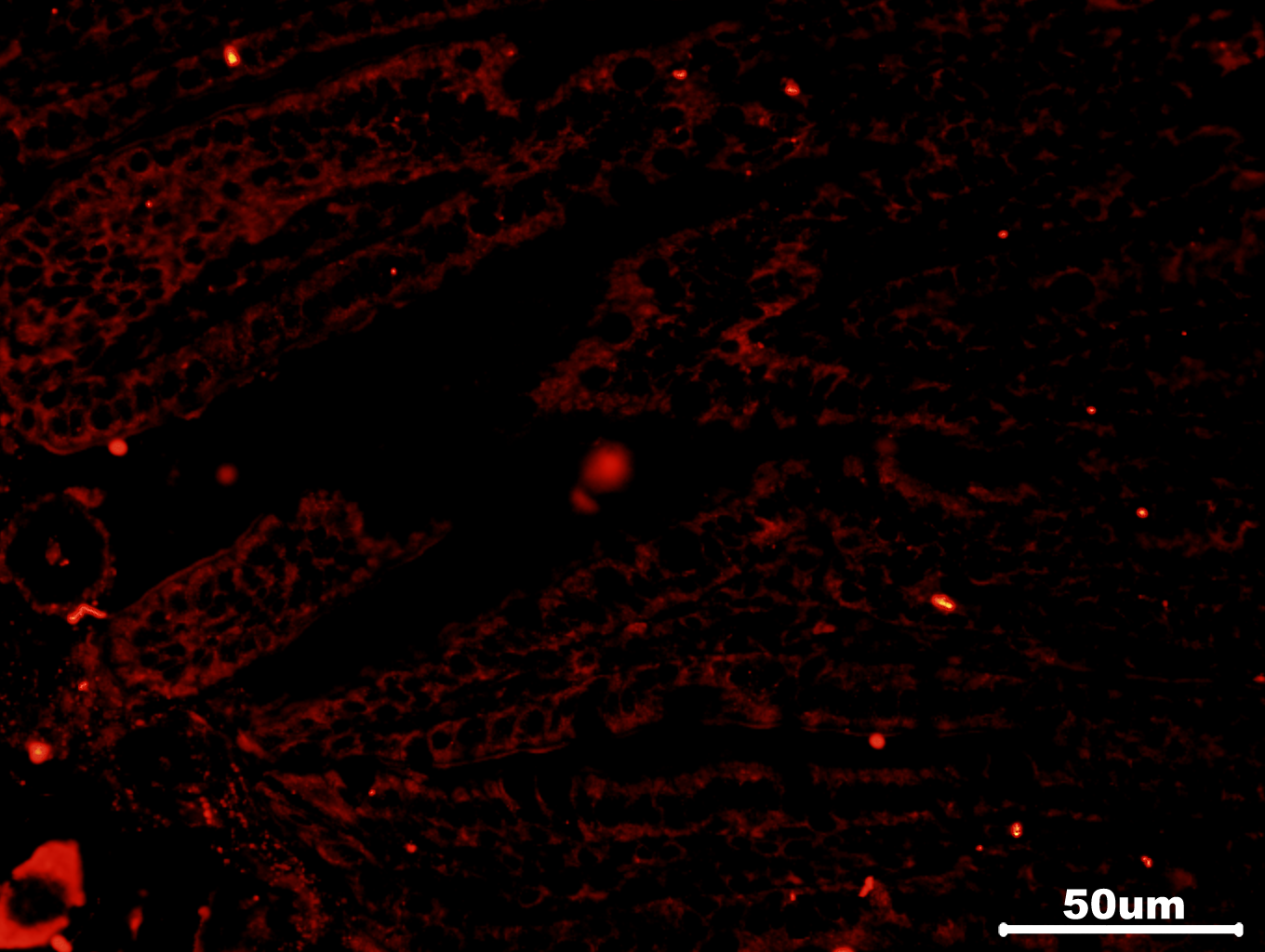

Supplement: Supplementary file 12 [file DataSheet_12.zip › D29-1-200-2-CD206.tif]

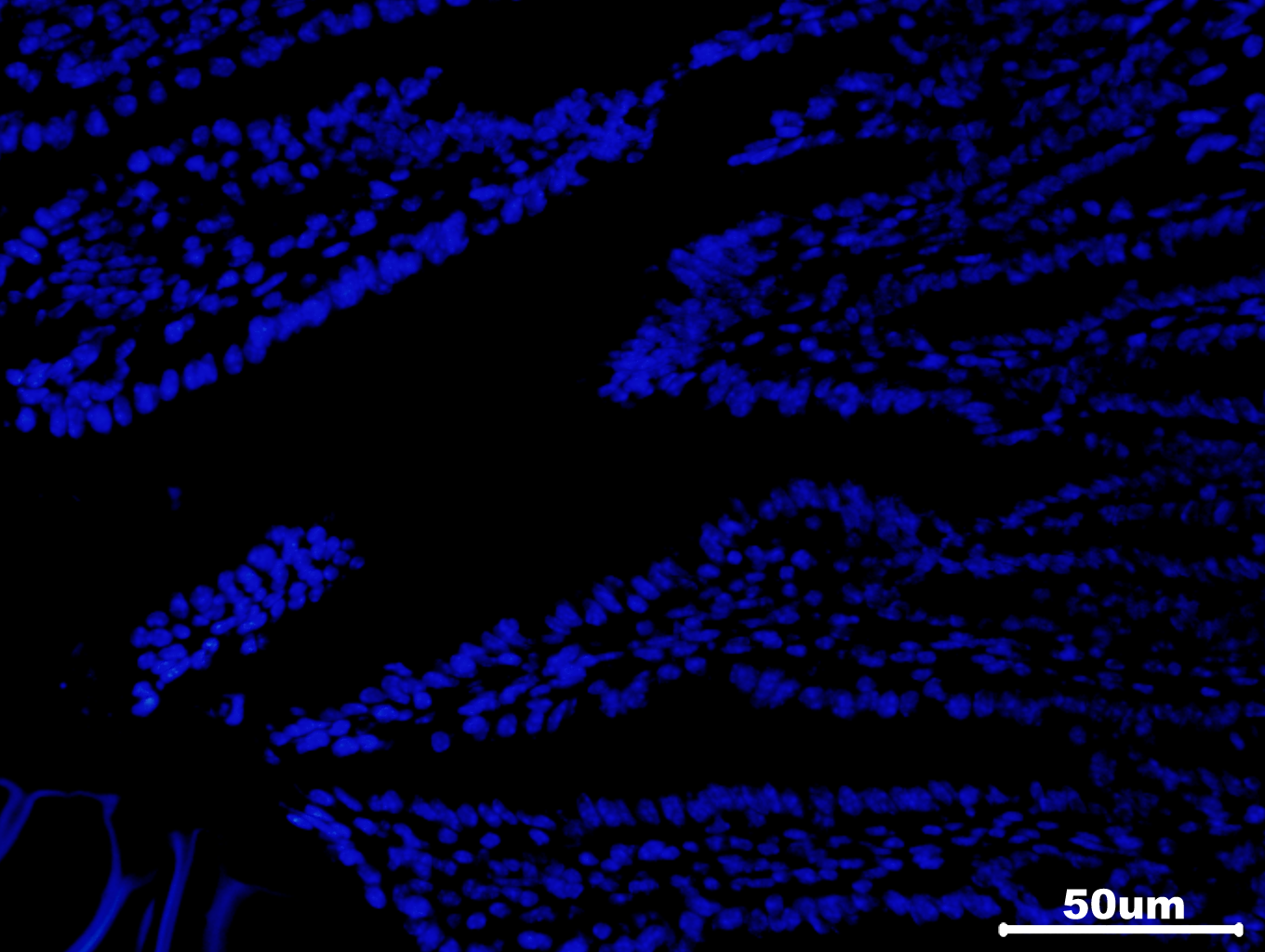

Supplement: Supplementary file 12 [file DataSheet_12.zip › D29-1-200-2-DAPI.tif]

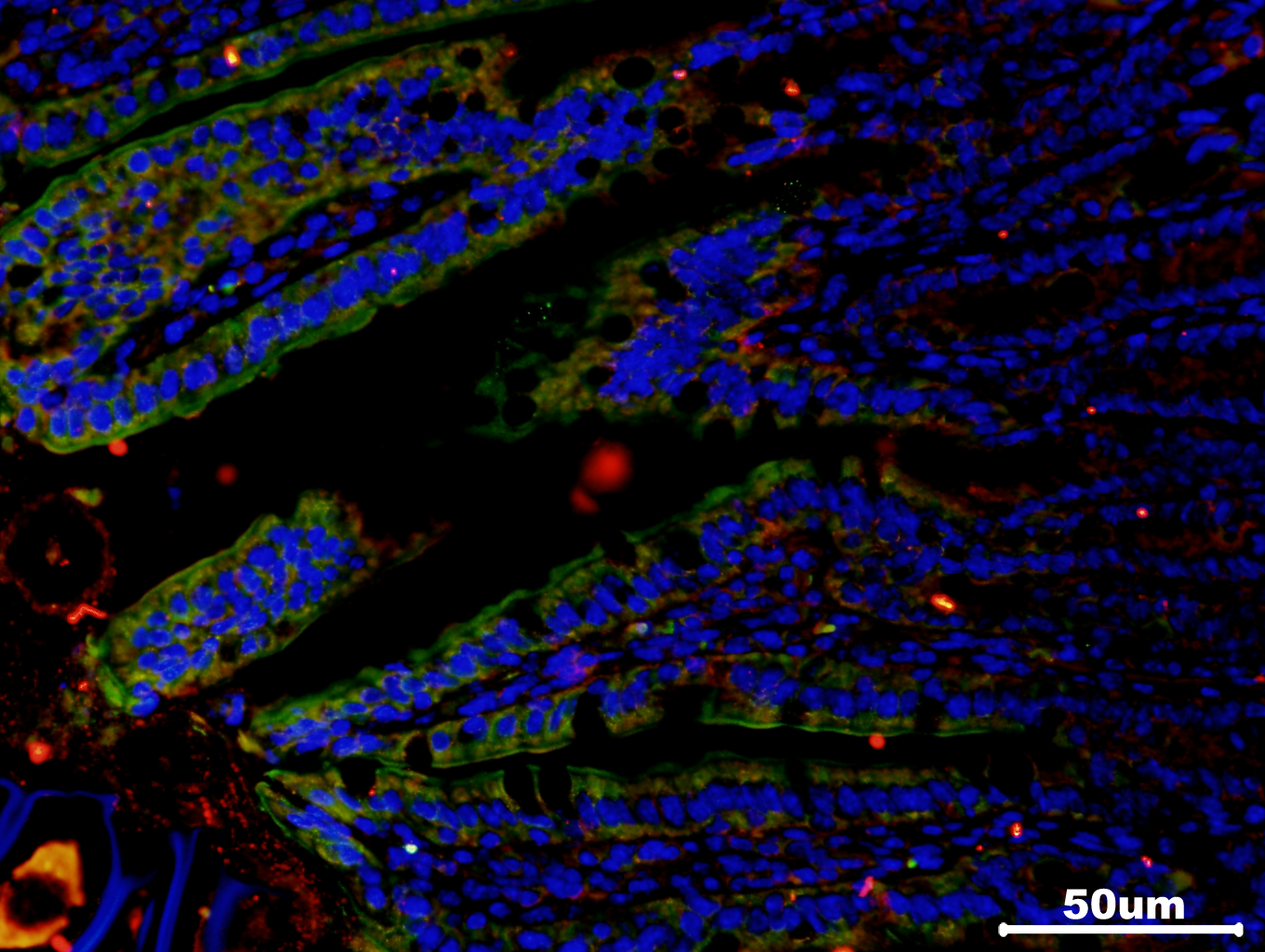

Supplement: Supplementary file 12 [file DataSheet_12.zip › D29-1-200-2-merge.tif]

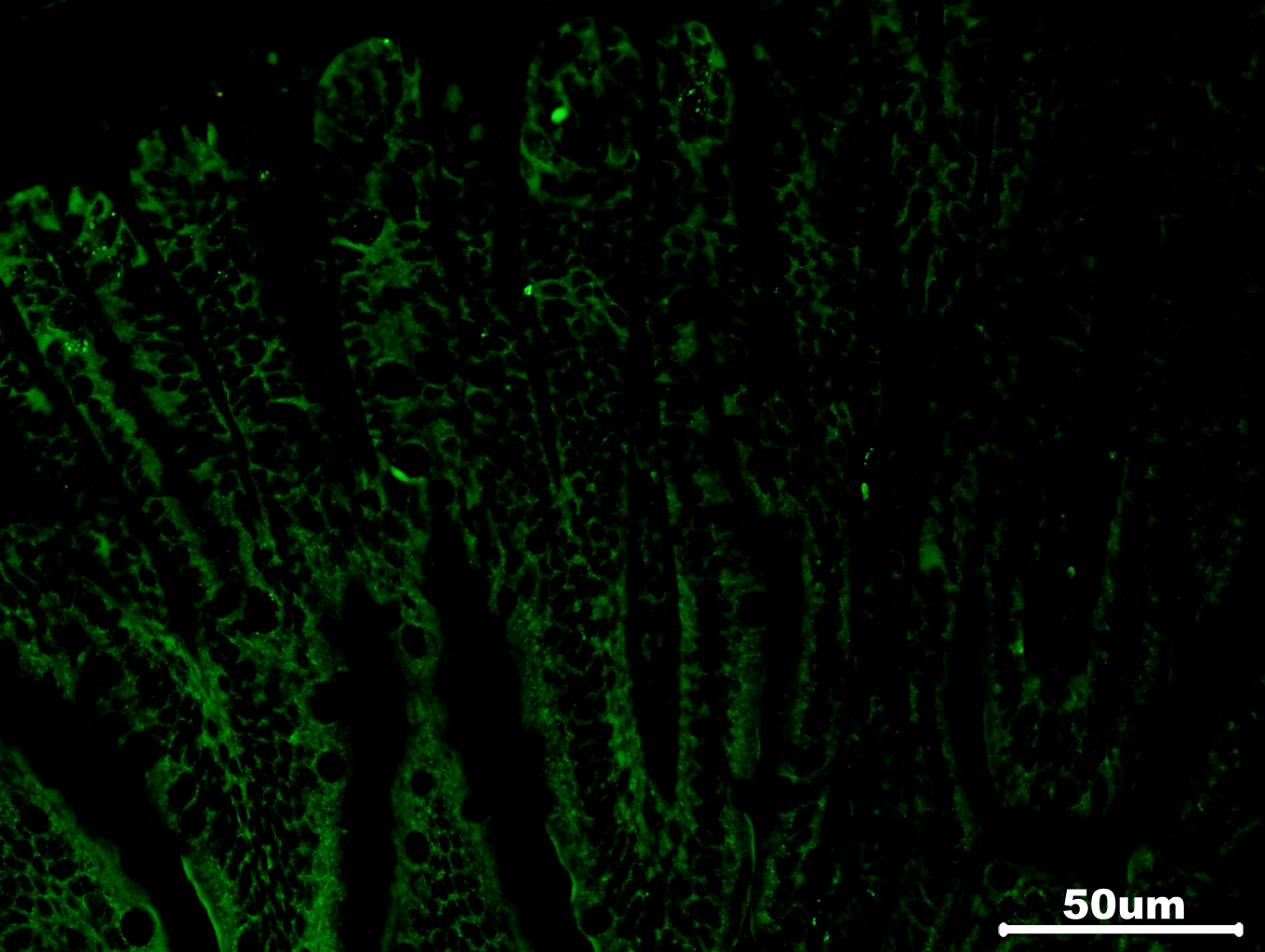

Supplement: Supplementary file 12 [file DataSheet_12.zip › D29-1-200-3-CD86.tif]
