# Supplementary figures and images for: Mechanism of electro-acupuncture in alleviating intestinal injury in septic mice via polyamine-related M2-macrophage polarization (part 3 of 3)
Source: Front Immunol. 2024 Apr 22;15:1373876. doi: 10.3389/fimmu.2024.1373876 (PMC11075497; doi:10.3389/fimmu.2024.1373876)

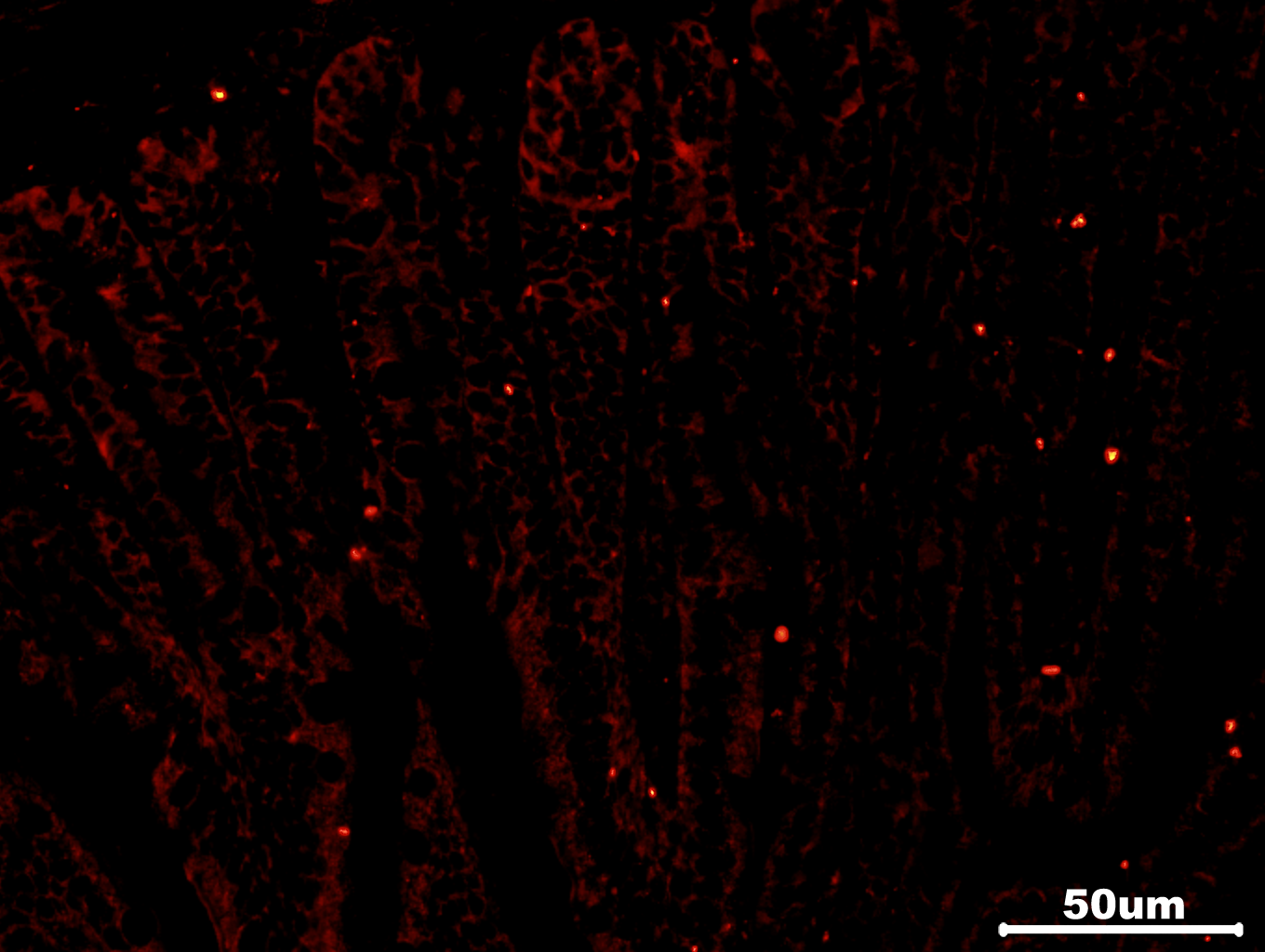

Supplement: Supplementary file 12 [file DataSheet_12.zip › D29-1-200-3-CD206.tif]

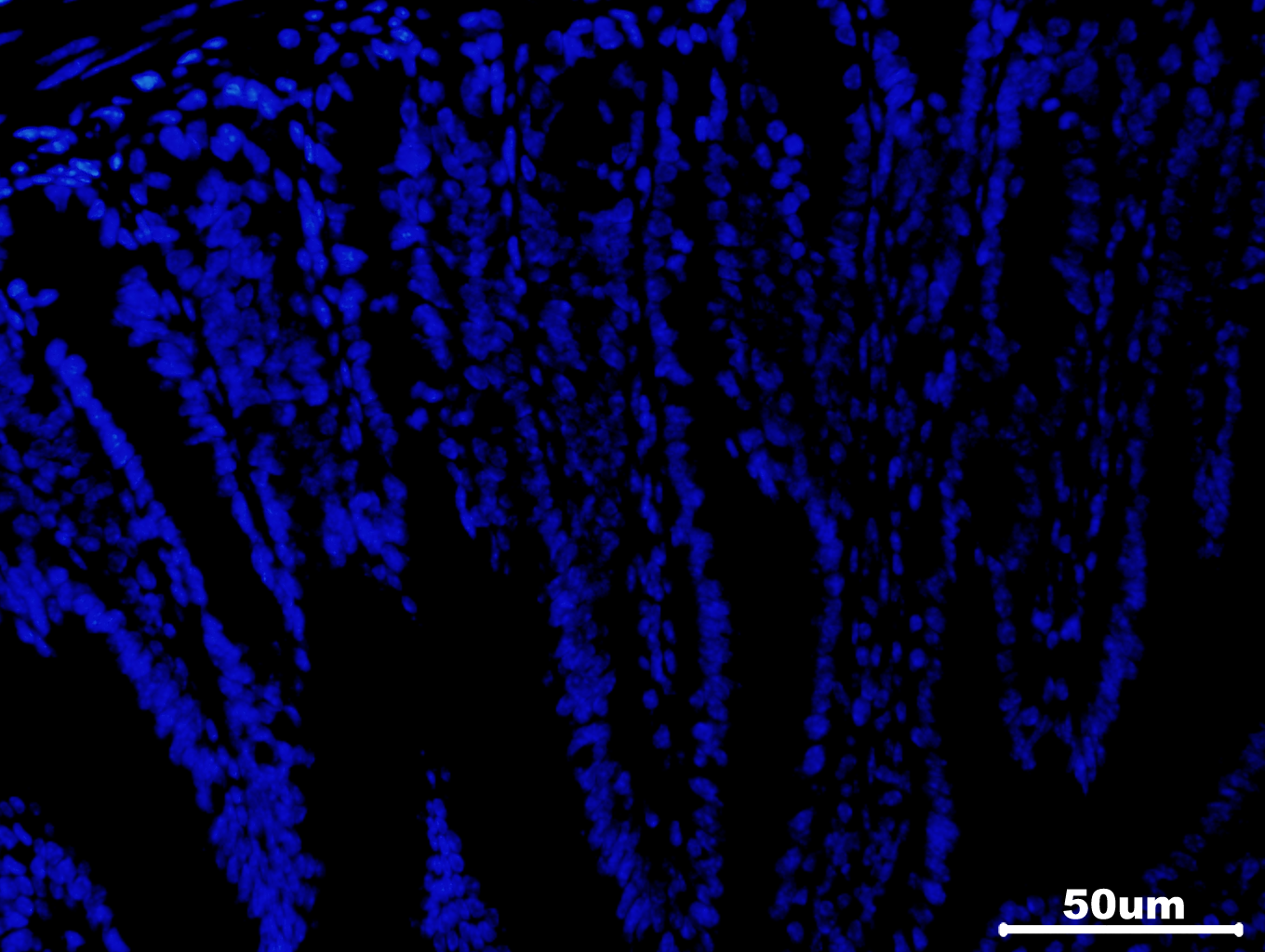

Supplement: Supplementary file 12 [file DataSheet_12.zip › D29-1-200-3-DAPI.tif]

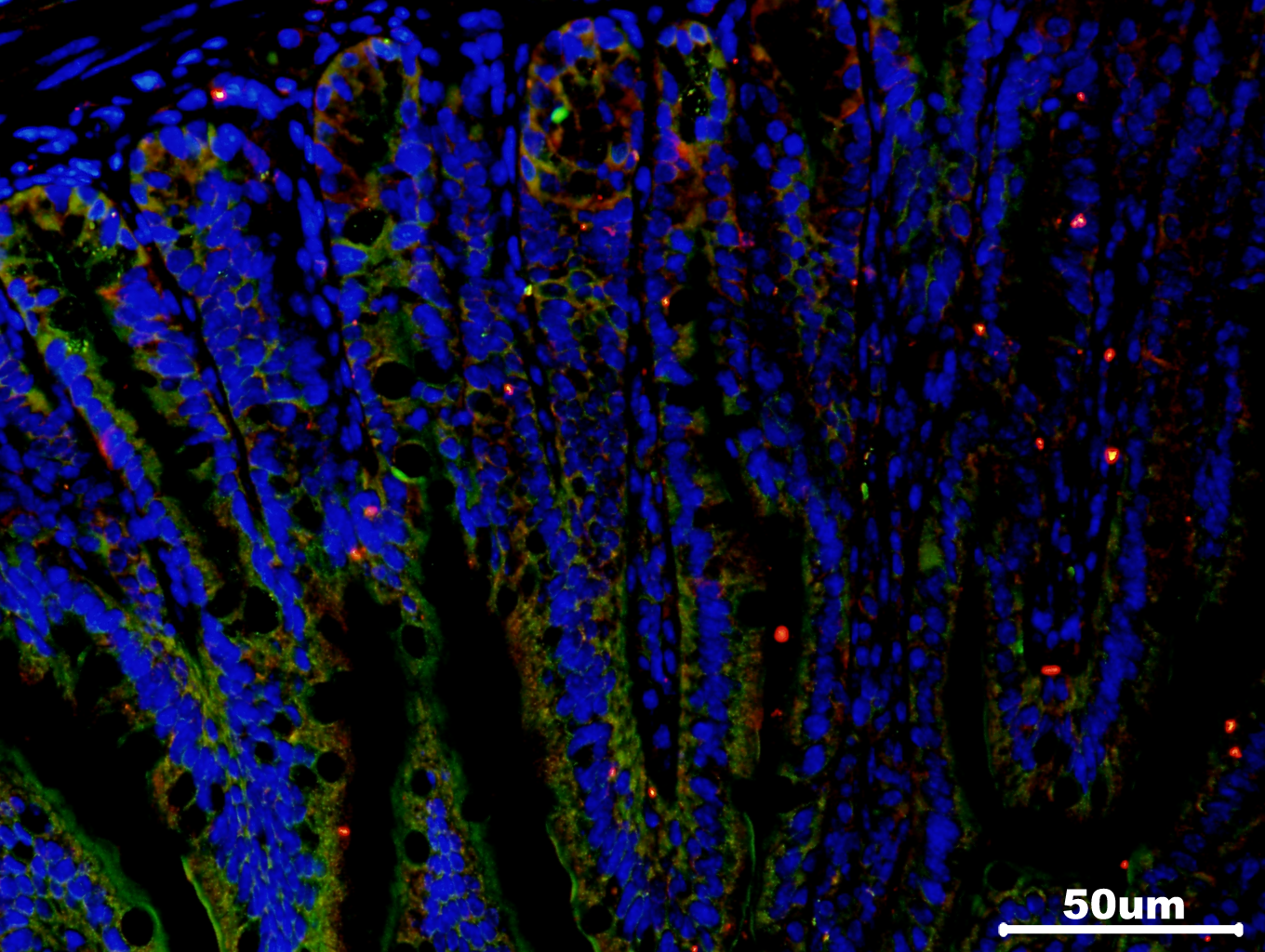

Supplement: Supplementary file 12 [file DataSheet_12.zip › D29-1-200-3-merge.tif]

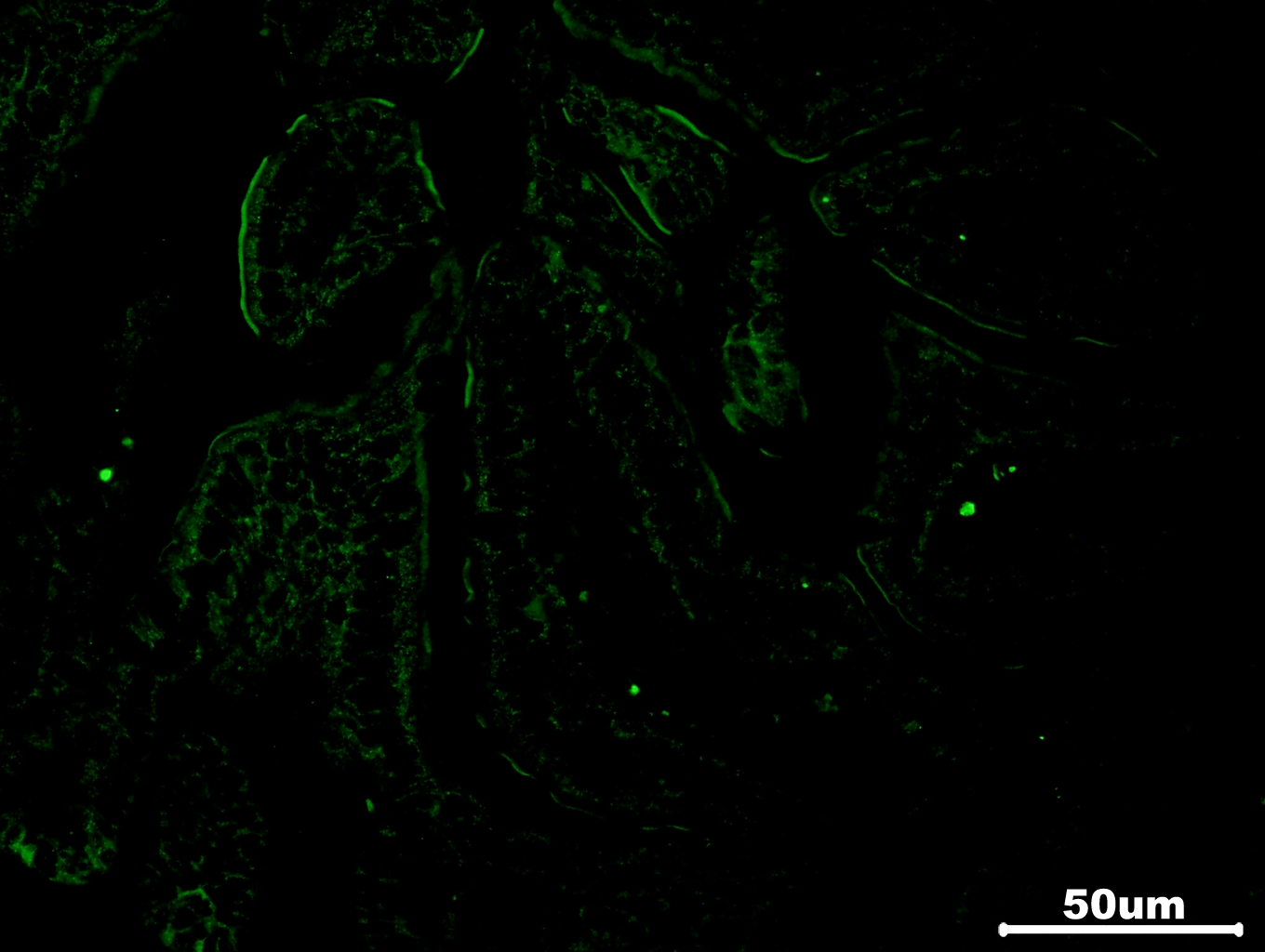

Supplement: Supplementary file 12 [file DataSheet_12.zip › D29-2-200-1-CD86.tif]

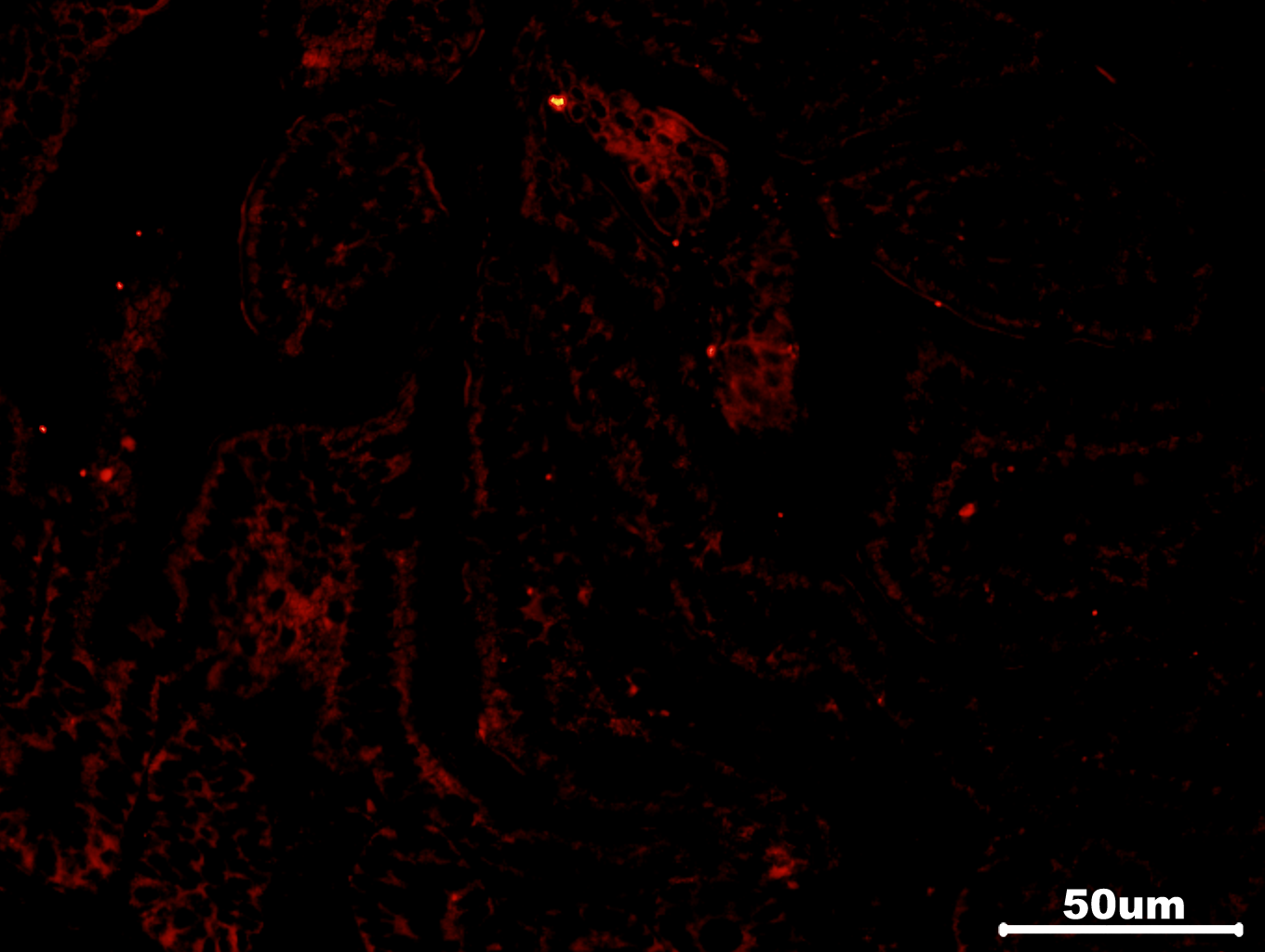

Supplement: Supplementary file 12 [file DataSheet_12.zip › D29-2-200-1-CD206.tif]

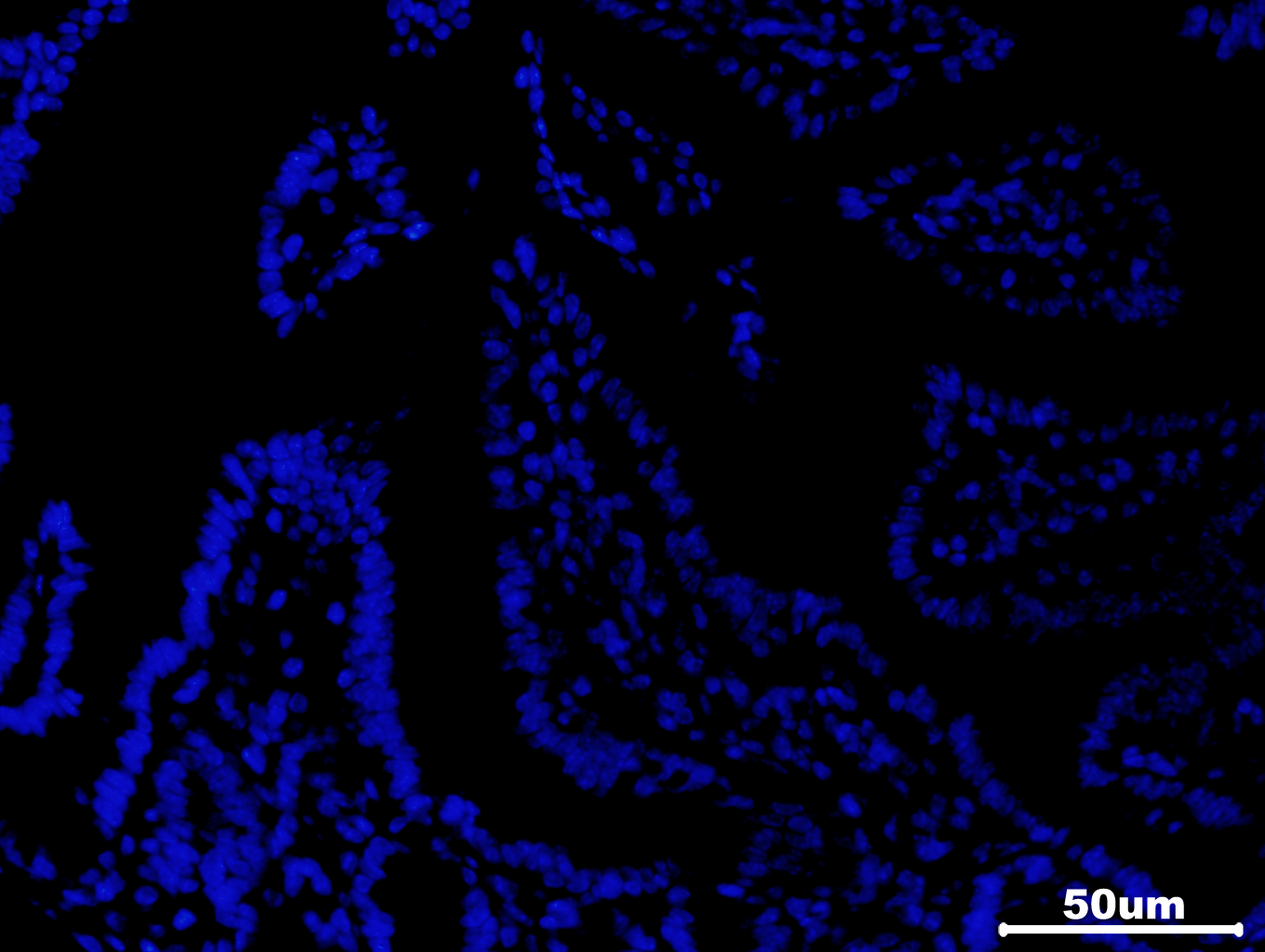

Supplement: Supplementary file 12 [file DataSheet_12.zip › D29-2-200-1-DAPI.tif]

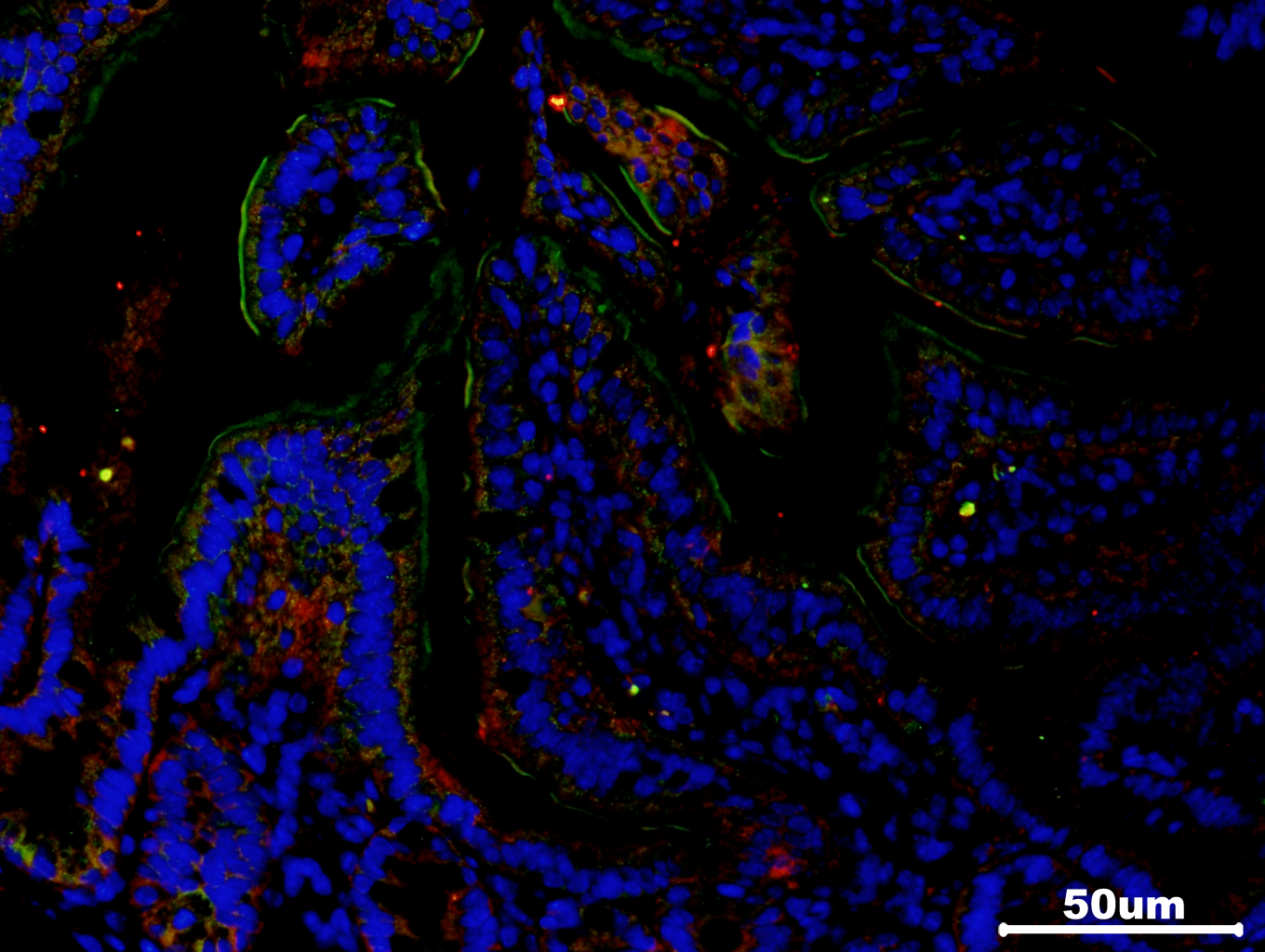

Supplement: Supplementary file 12 [file DataSheet_12.zip › D29-2-200-1-merge.tif]

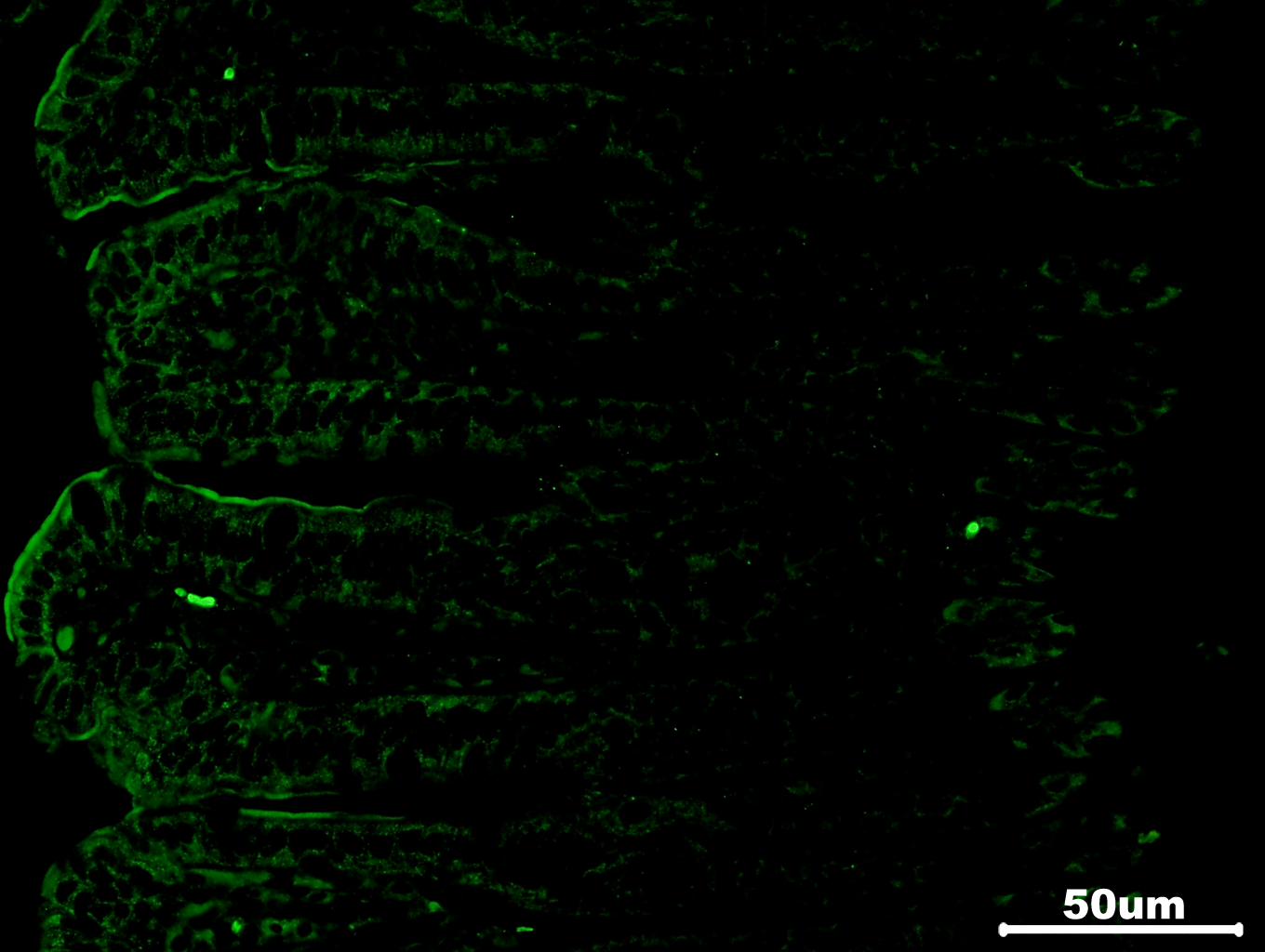

Supplement: Supplementary file 12 [file DataSheet_12.zip › D29-2-200-2-CD86.tif]

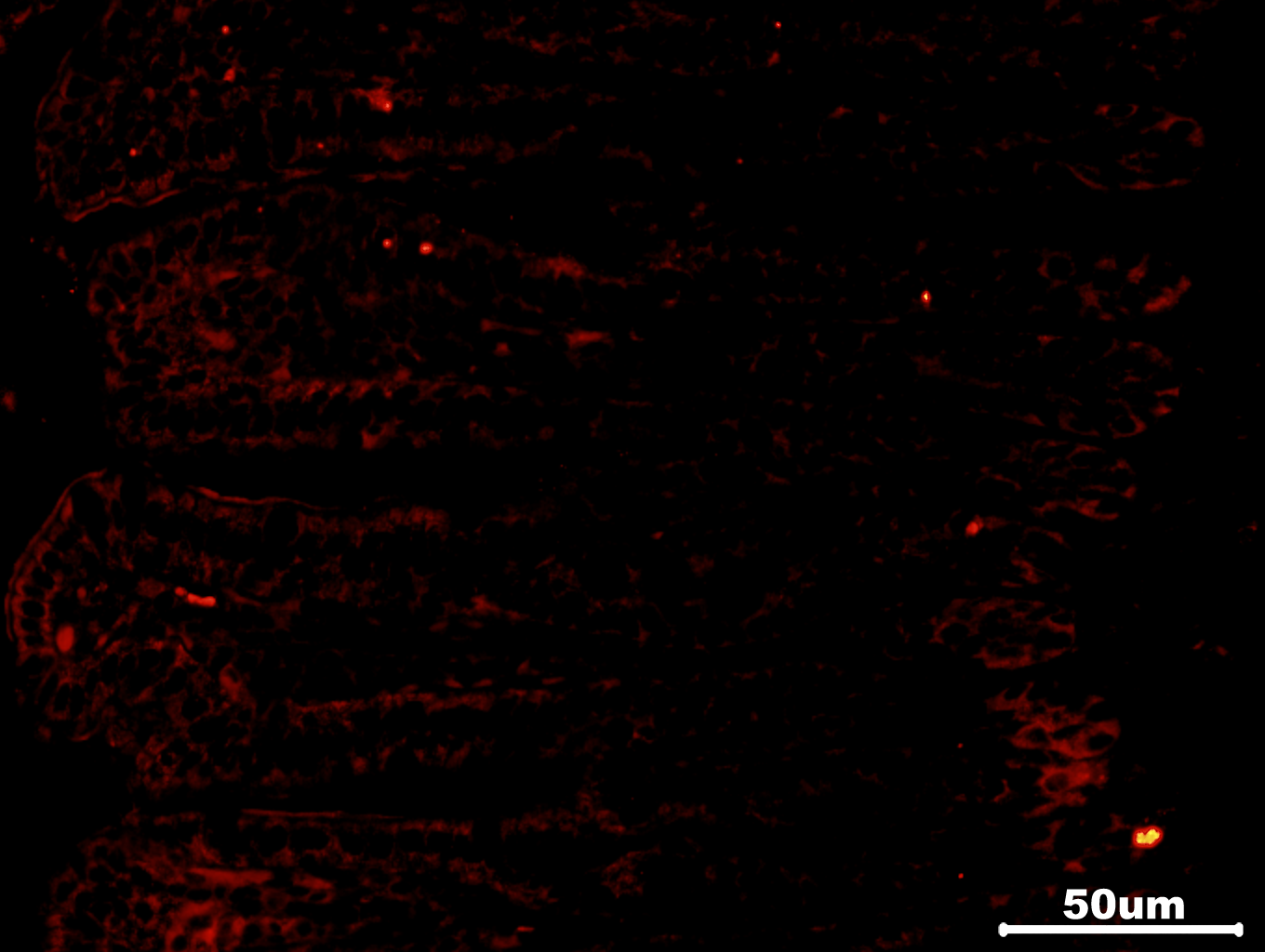

Supplement: Supplementary file 12 [file DataSheet_12.zip › D29-2-200-2-CD206.tif]

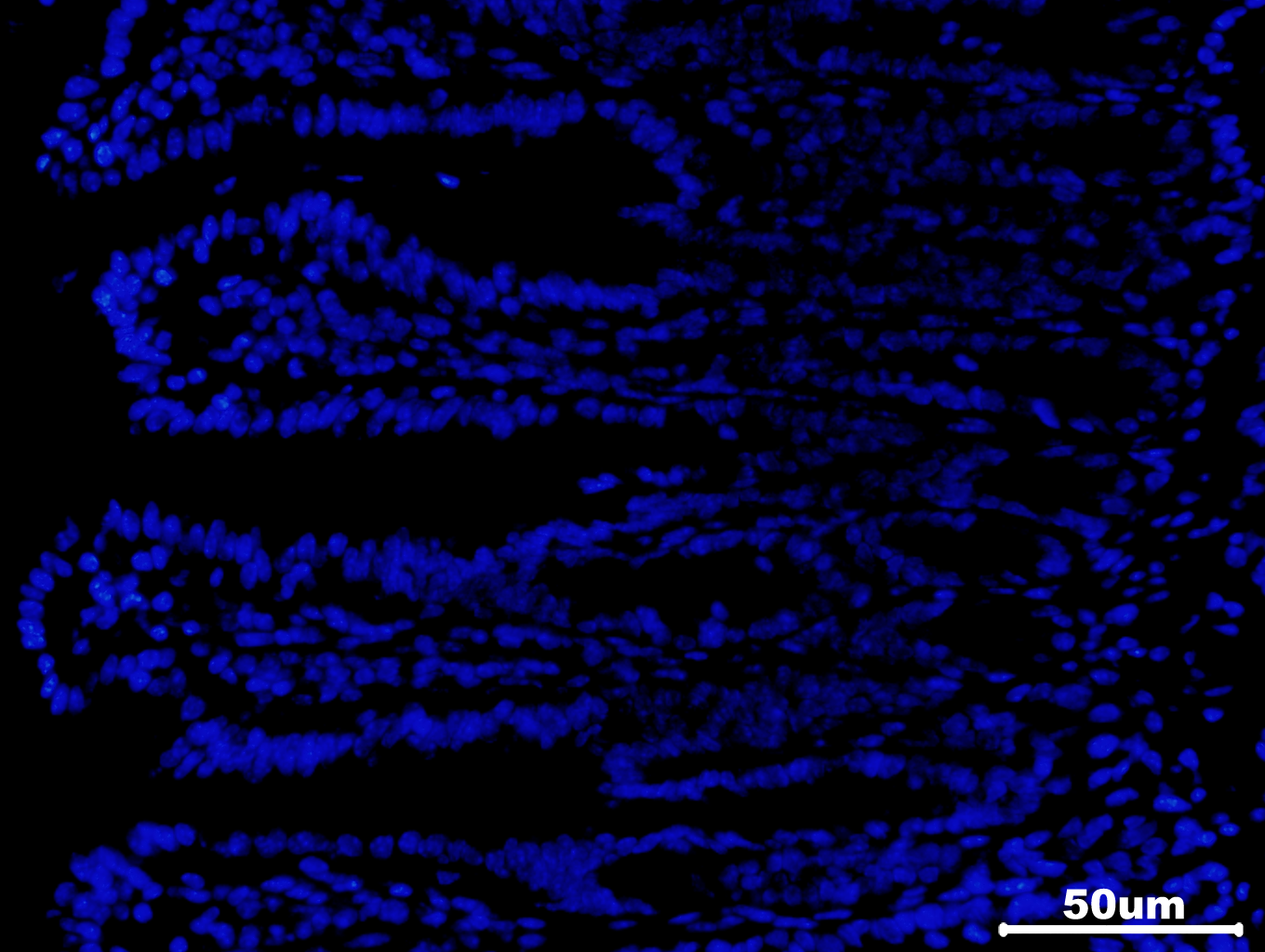

Supplement: Supplementary file 12 [file DataSheet_12.zip › D29-2-200-2-DAPI.tif]

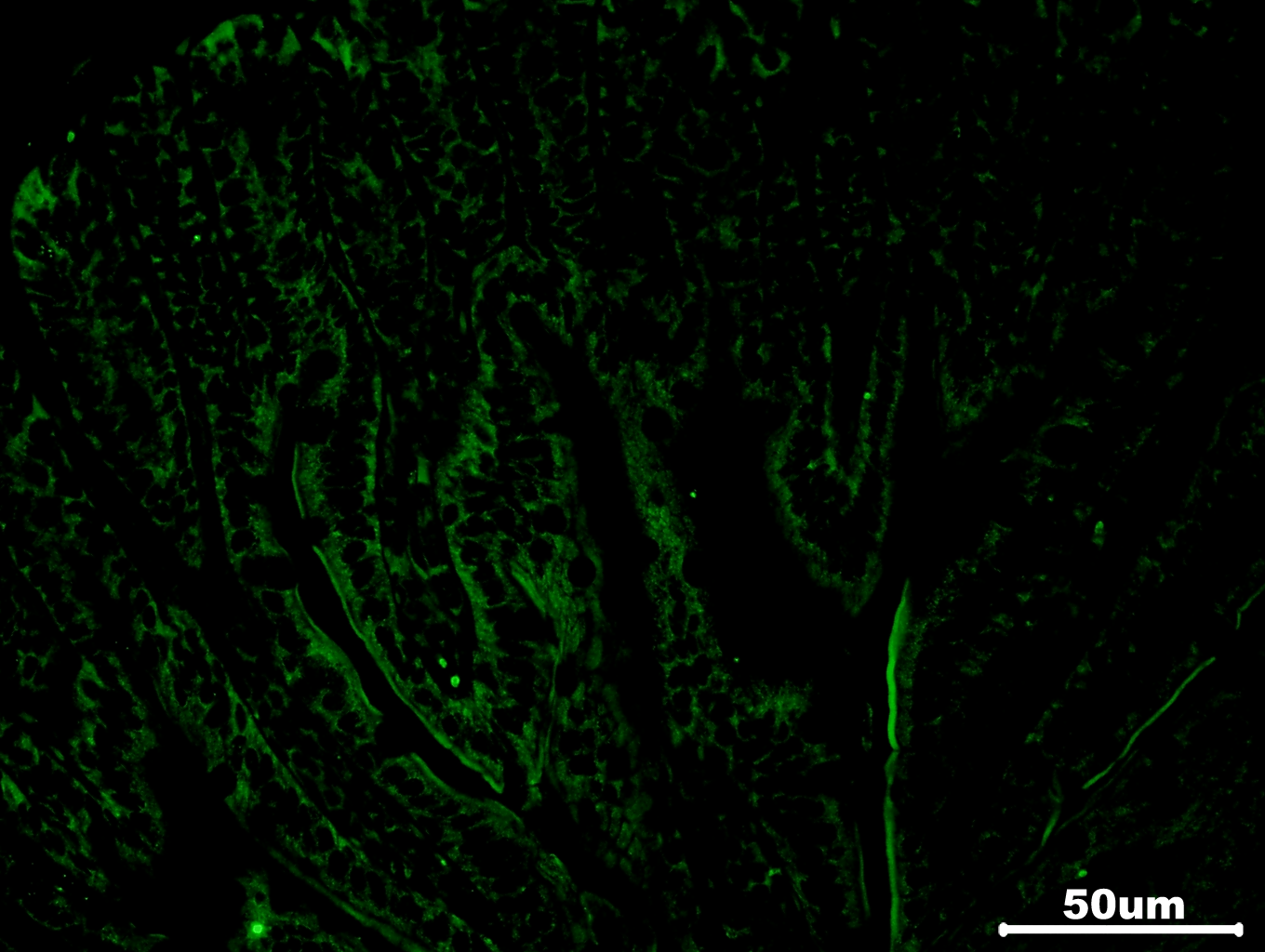

Supplement: Supplementary file 12 [file DataSheet_12.zip › D29-2-200-3-CD86.tif]

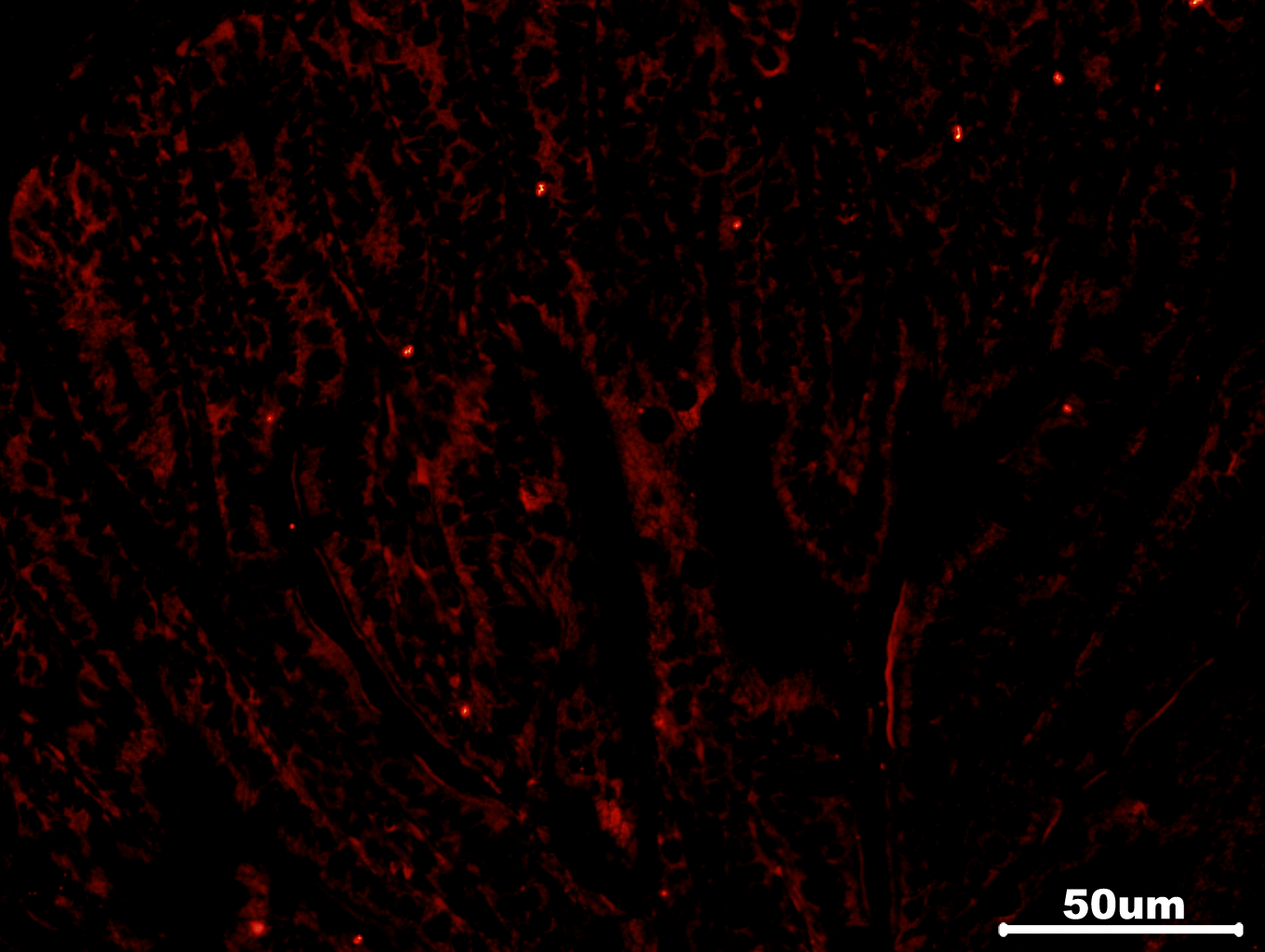

Supplement: Supplementary file 12 [file DataSheet_12.zip › D29-2-200-3-CD206.tif]

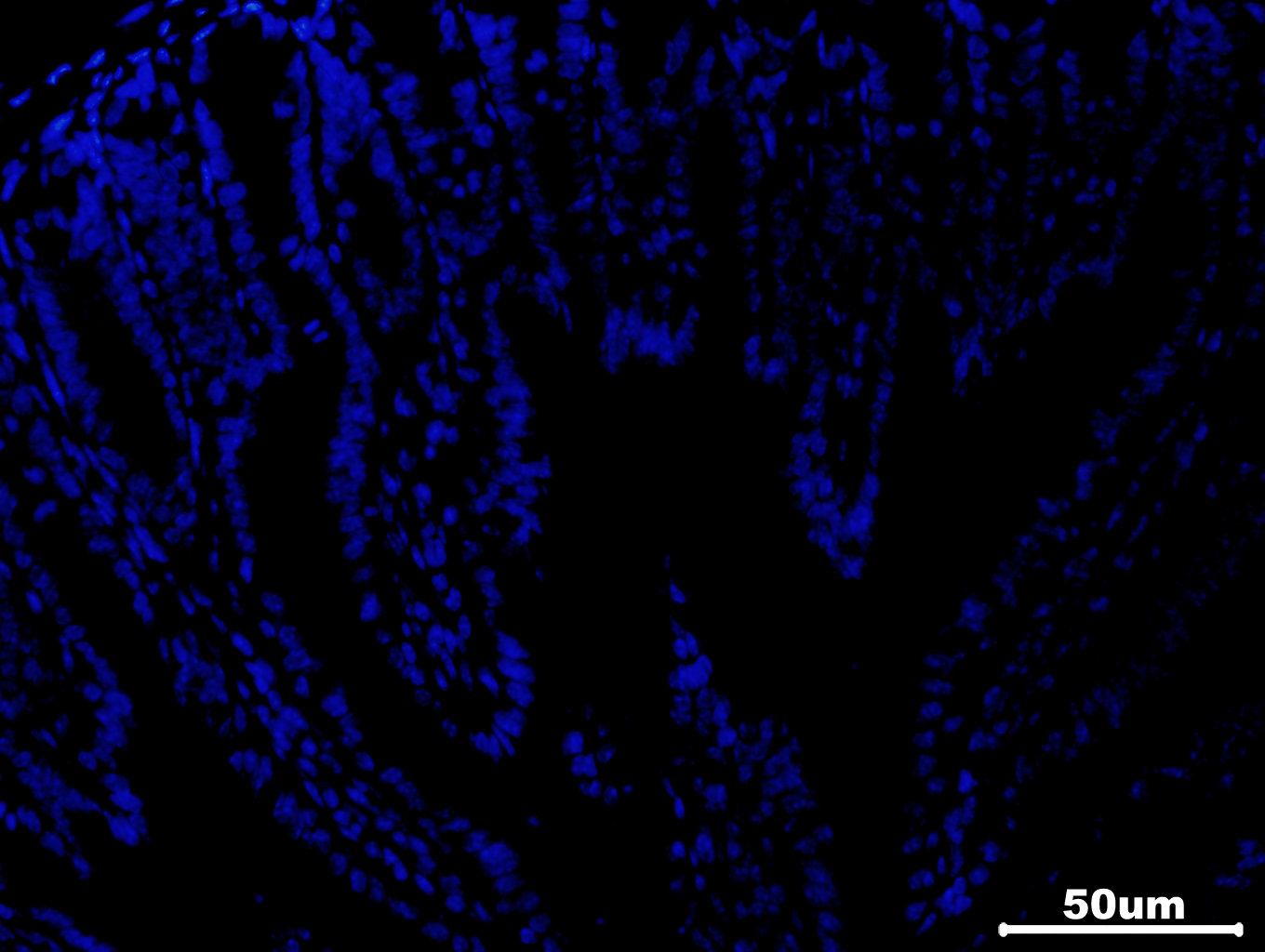

Supplement: Supplementary file 12 [file DataSheet_12.zip › D29-2-200-3-DAPI.tif]

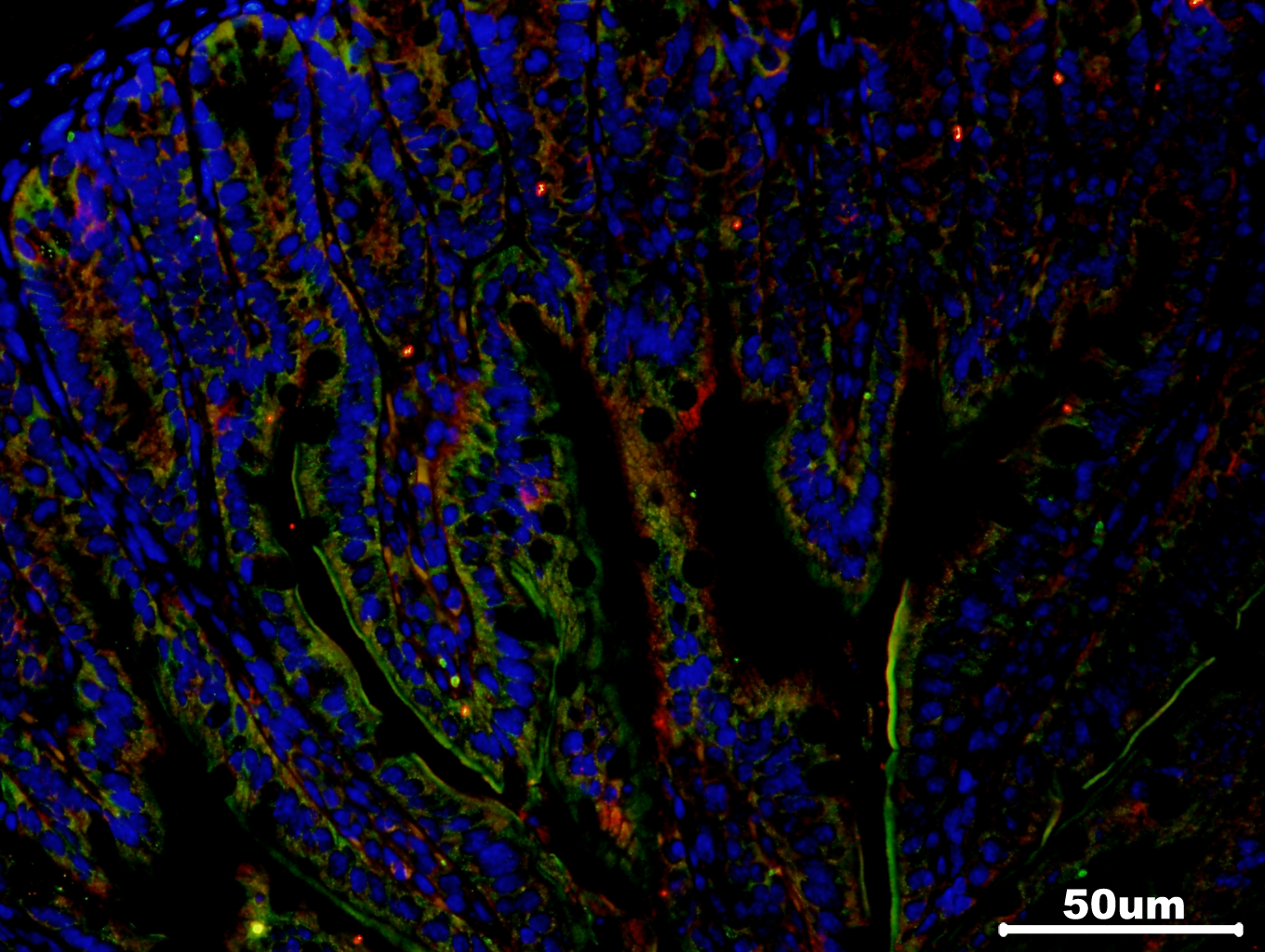

Supplement: Supplementary file 12 [file DataSheet_12.zip › D29-2-200-3-merge.tif]

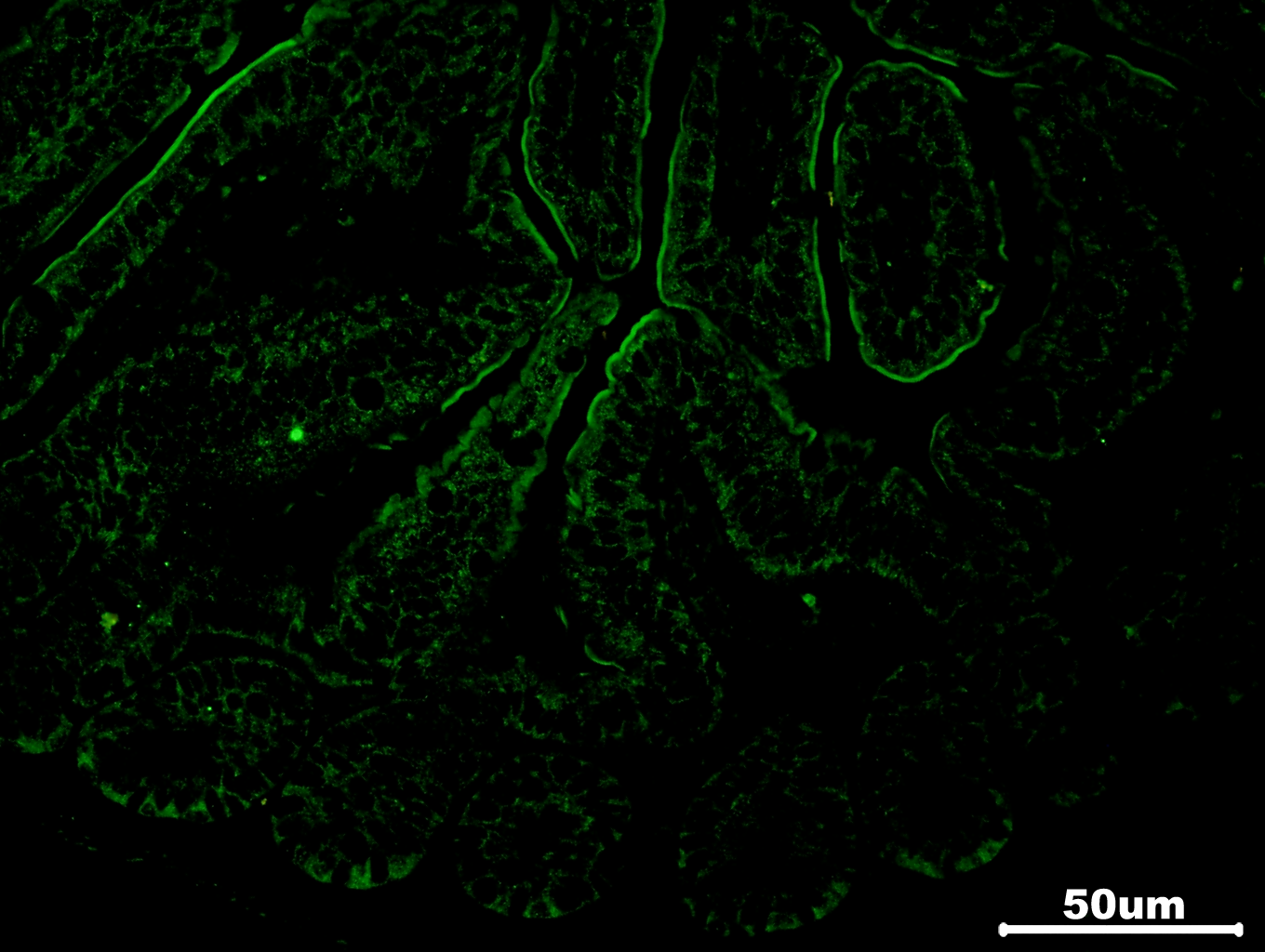

Supplement: Supplementary file 12 [file DataSheet_12.zip › D30-1-200-1-CD86.tif]

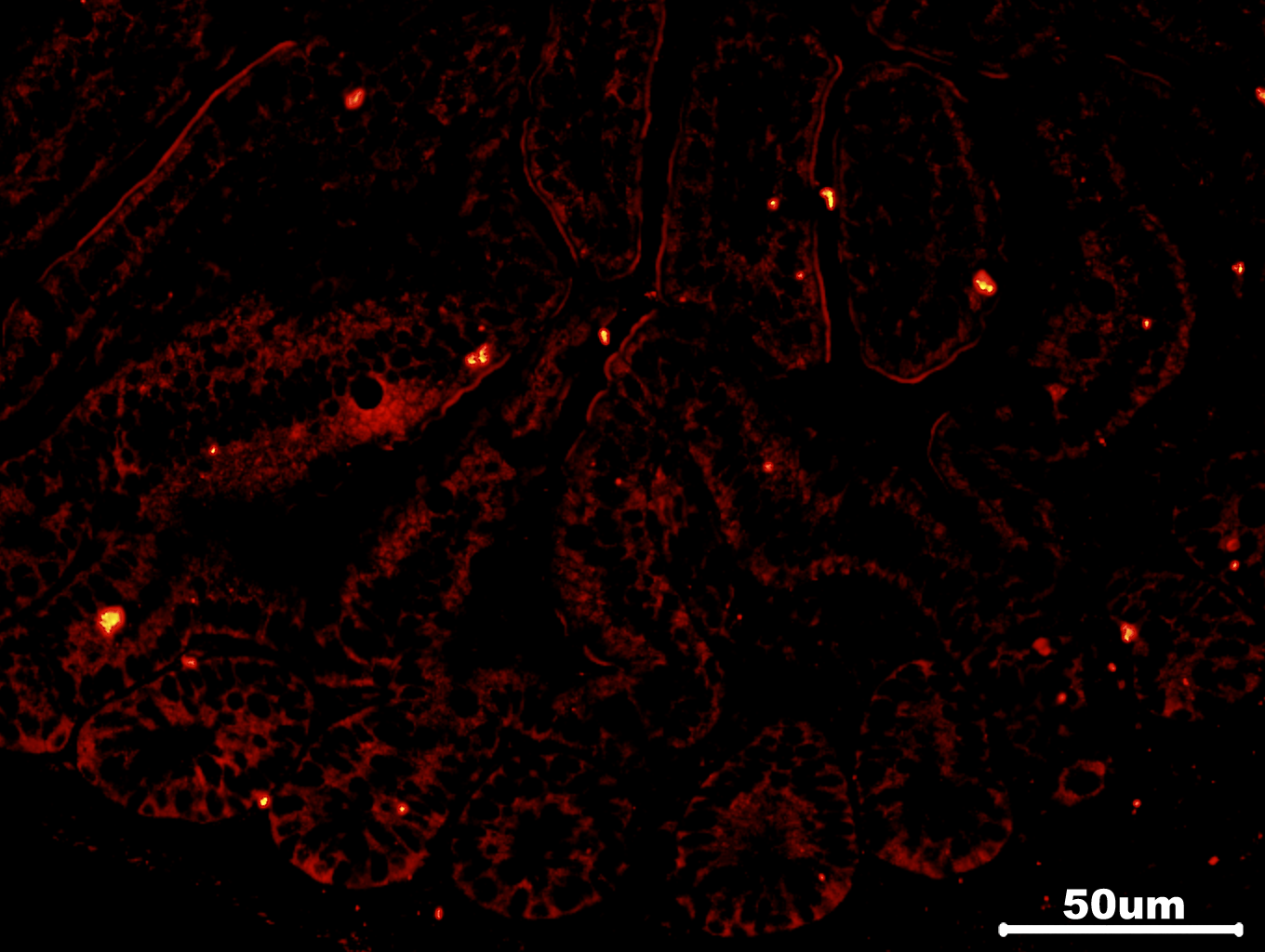

Supplement: Supplementary file 12 [file DataSheet_12.zip › D30-1-200-1-CD206.tif]

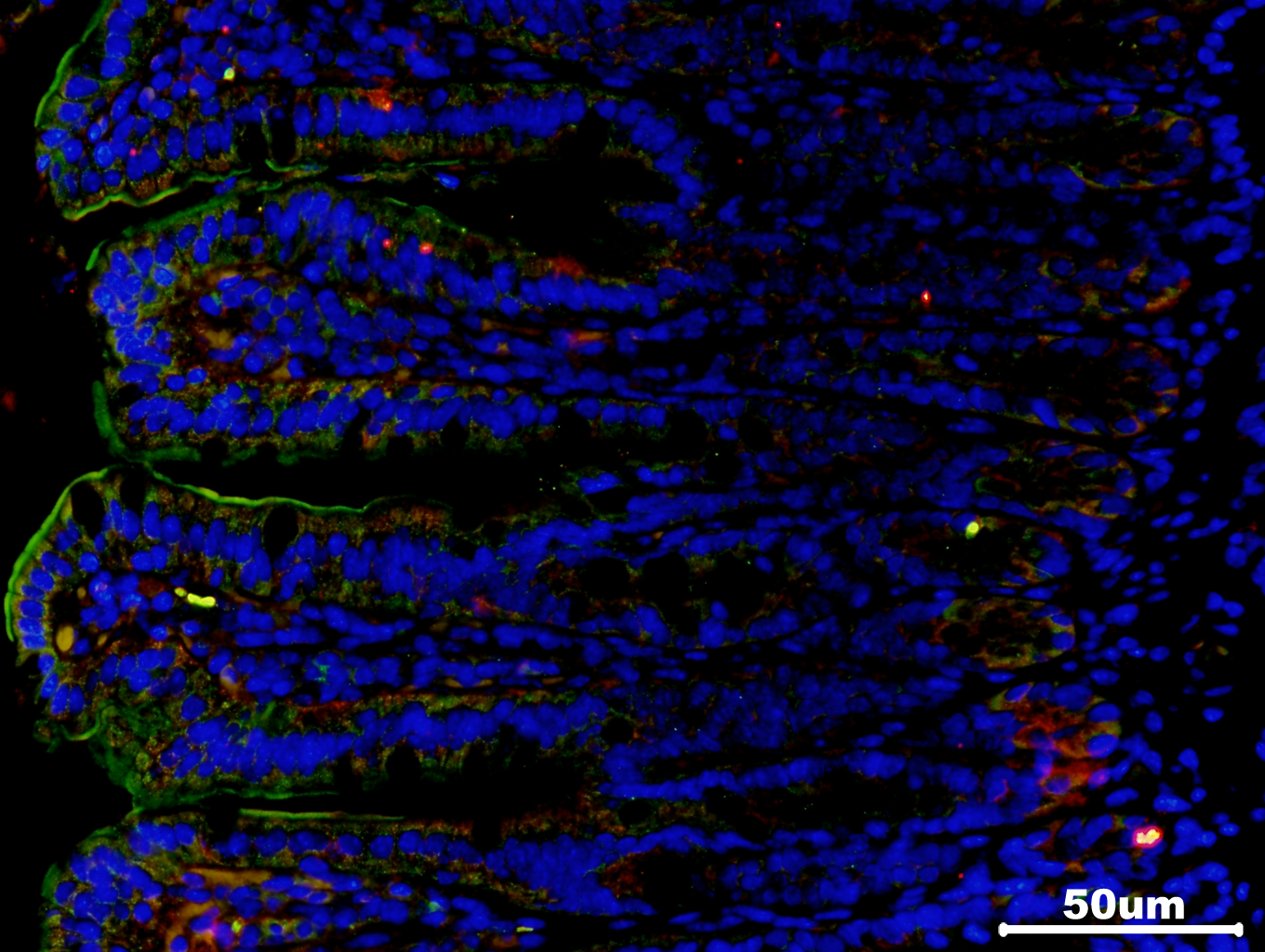

Supplement: Supplementary file 12 [file DataSheet_12.zip › D29-2-200-2-merge.tif]

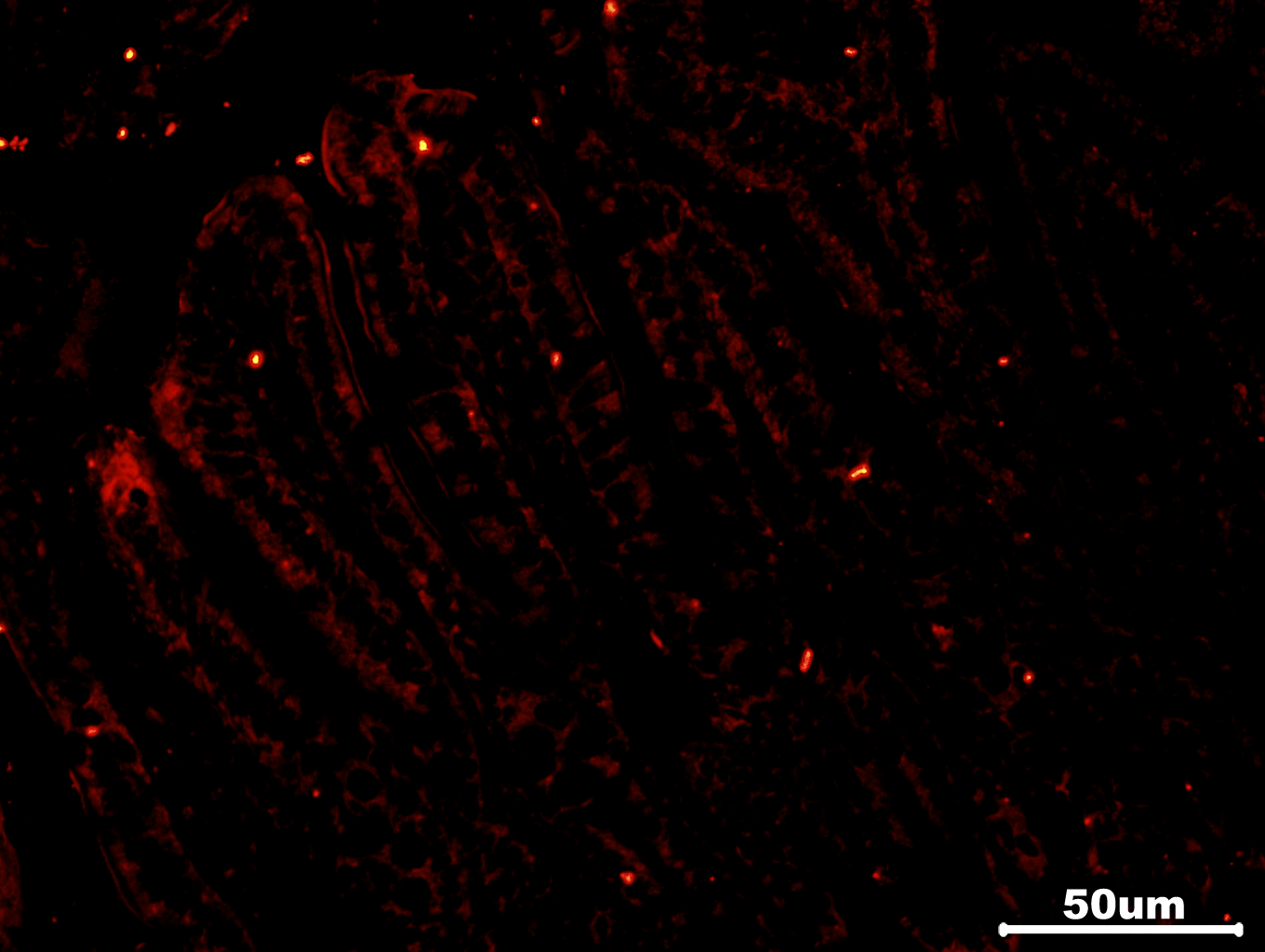

Supplement: Supplementary file 13 [file DataSheet_13.zip › E32-1-200-1-CD206.tif]

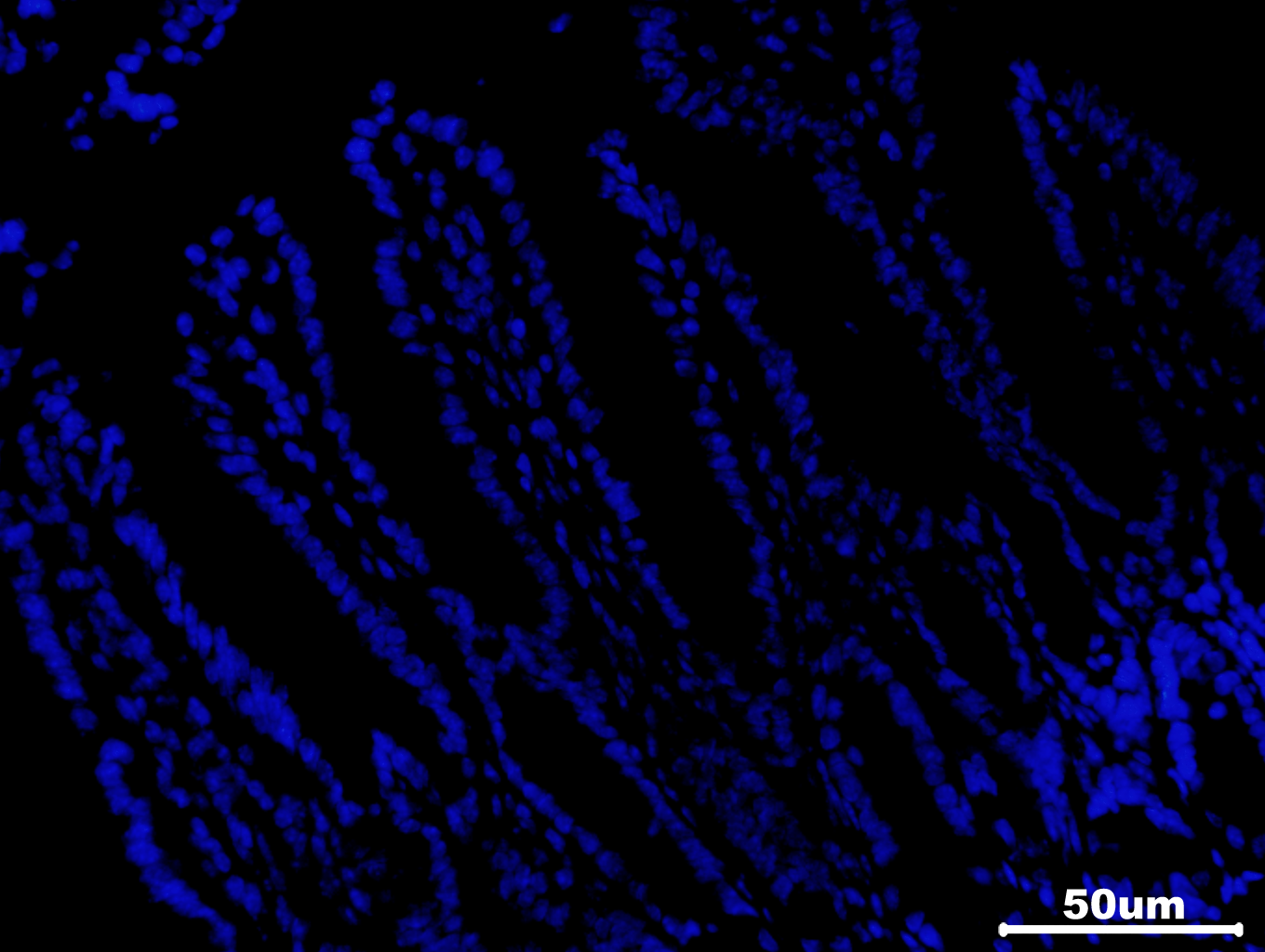

Supplement: Supplementary file 13 [file DataSheet_13.zip › E32-1-200-1-DAPI.tif]

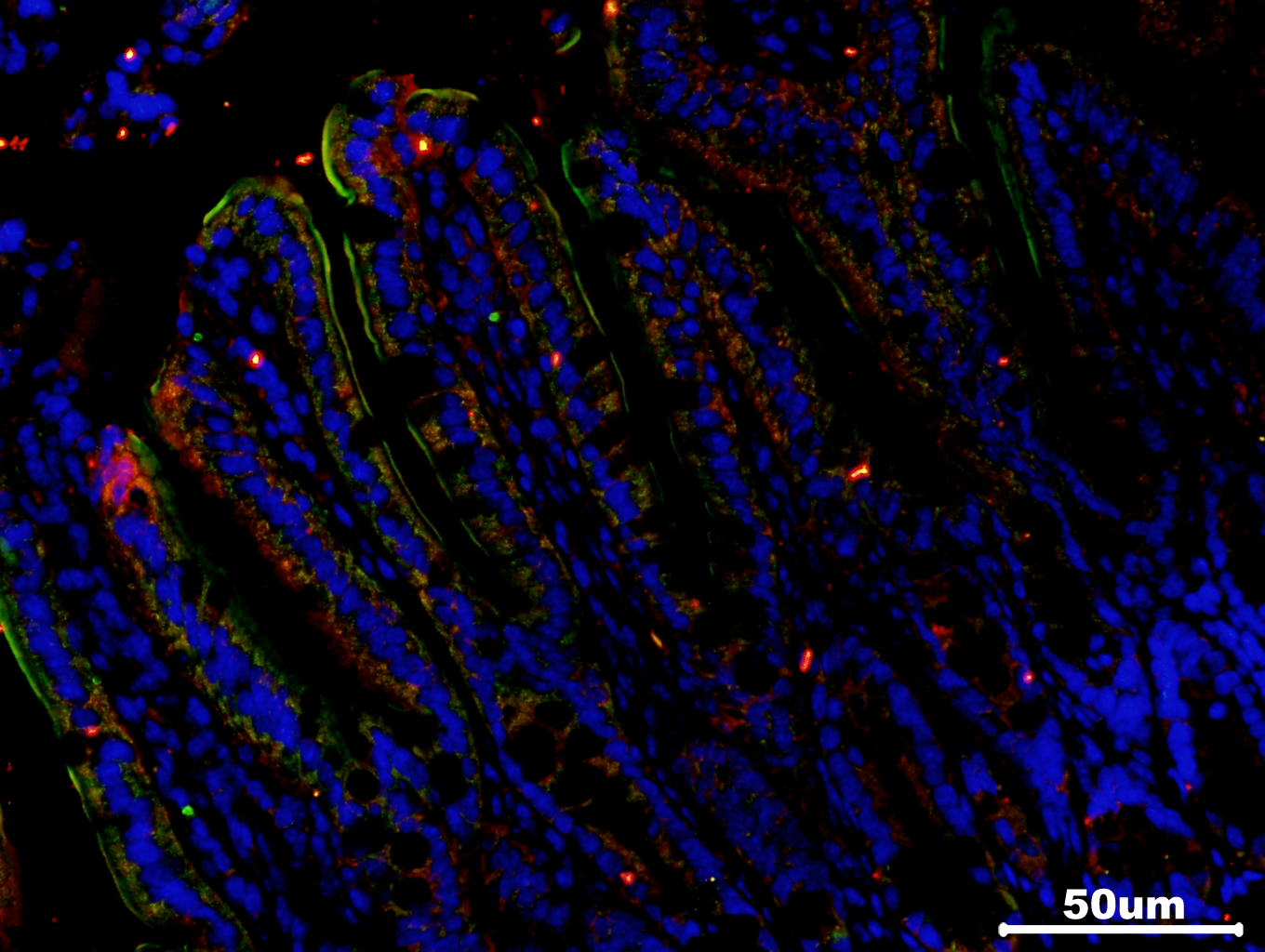

Supplement: Supplementary file 13 [file DataSheet_13.zip › E32-1-200-1-merge.tif]

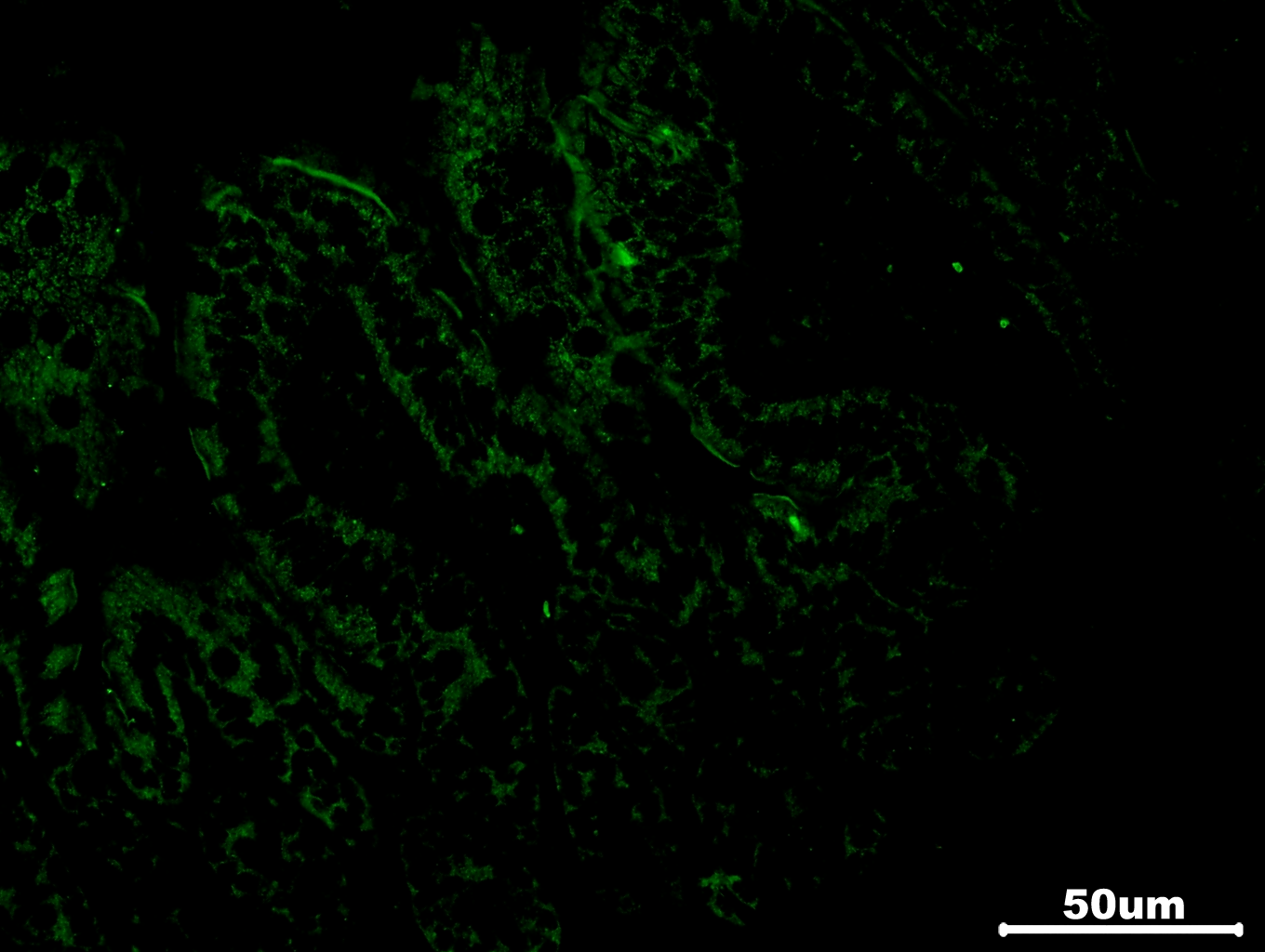

Supplement: Supplementary file 13 [file DataSheet_13.zip › E32-1-200-2-CD86.tif]

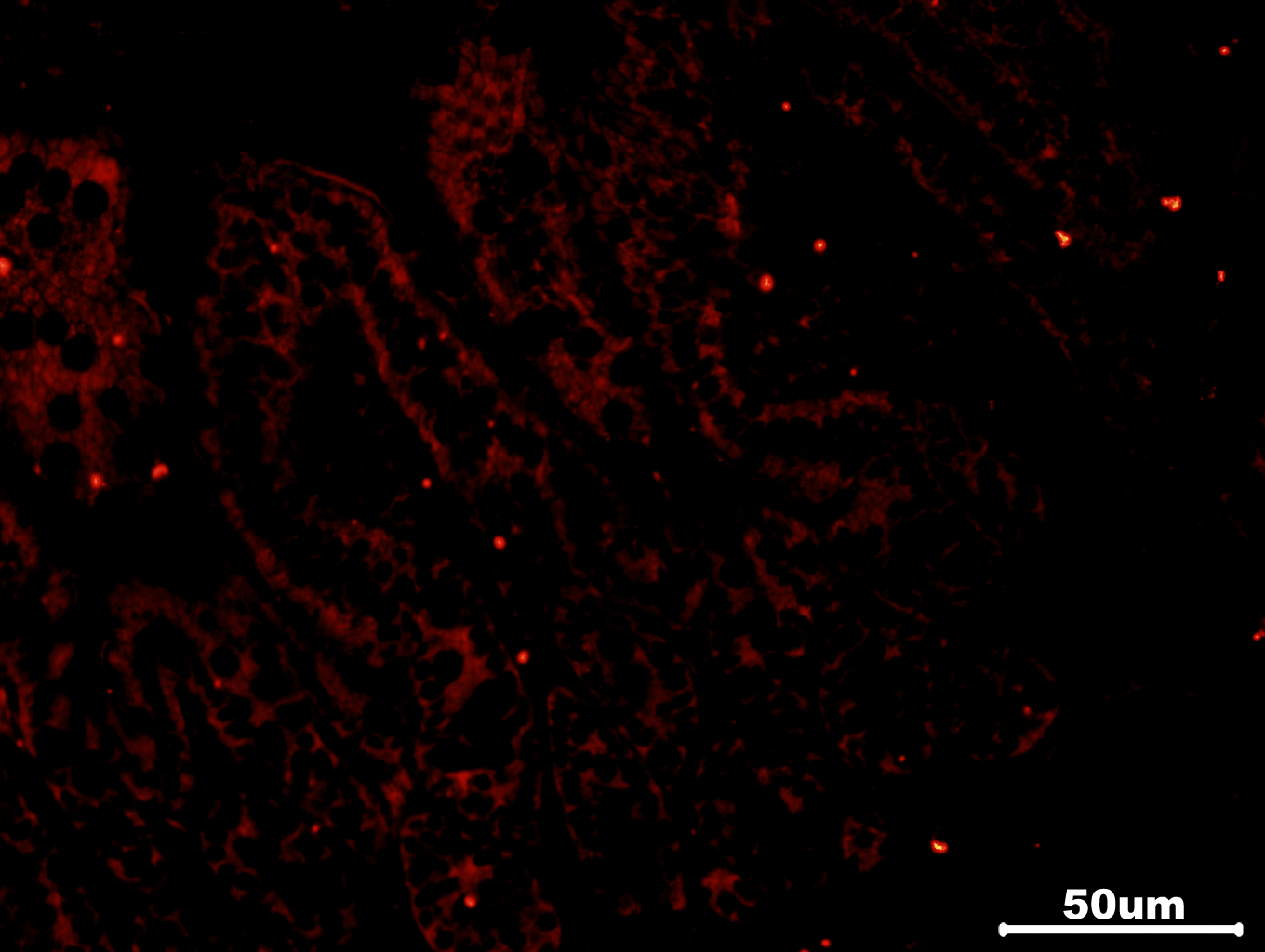

Supplement: Supplementary file 13 [file DataSheet_13.zip › E32-1-200-2-CD206.tif]

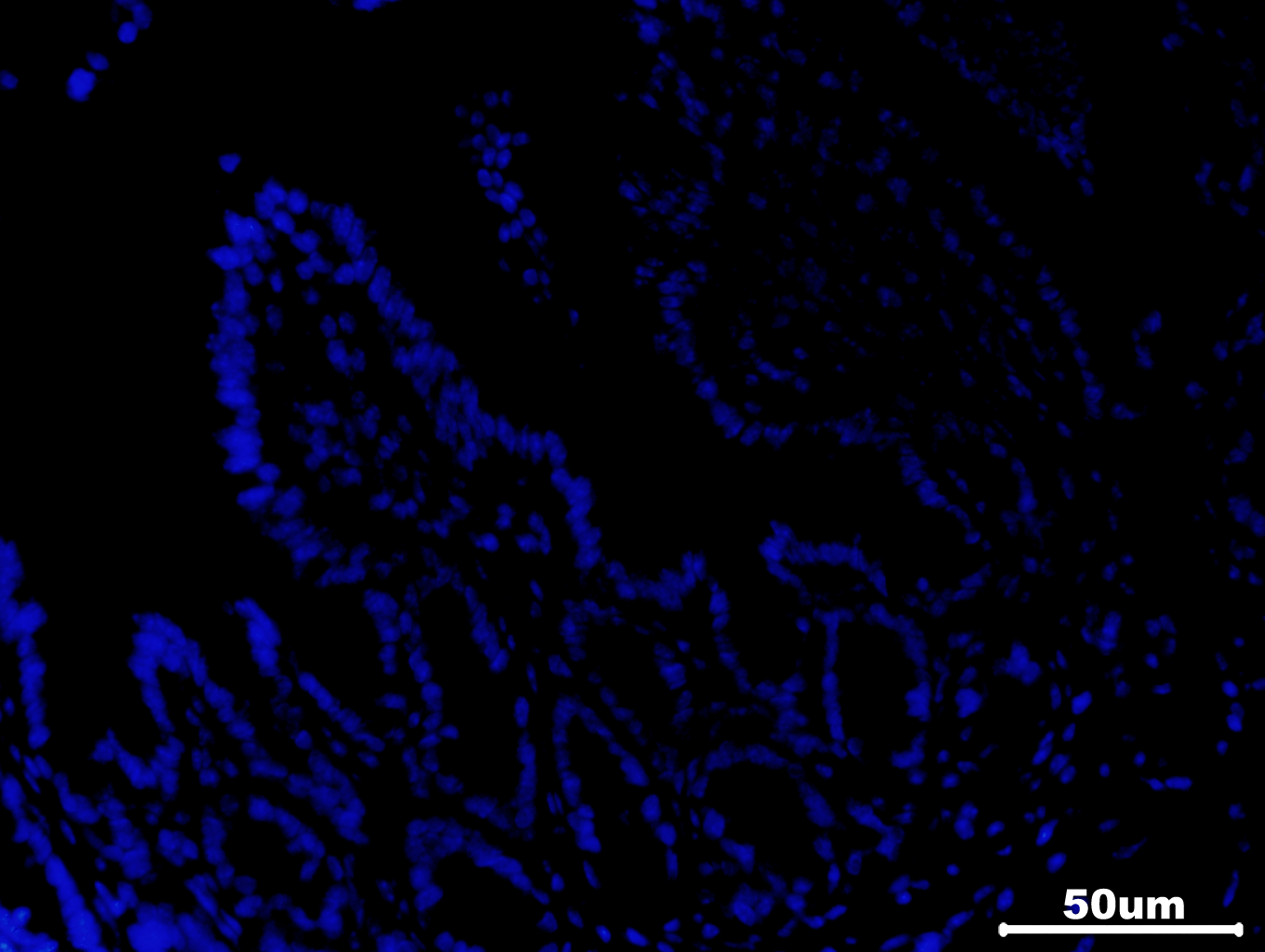

Supplement: Supplementary file 13 [file DataSheet_13.zip › E32-1-200-2-DAPI.tif]

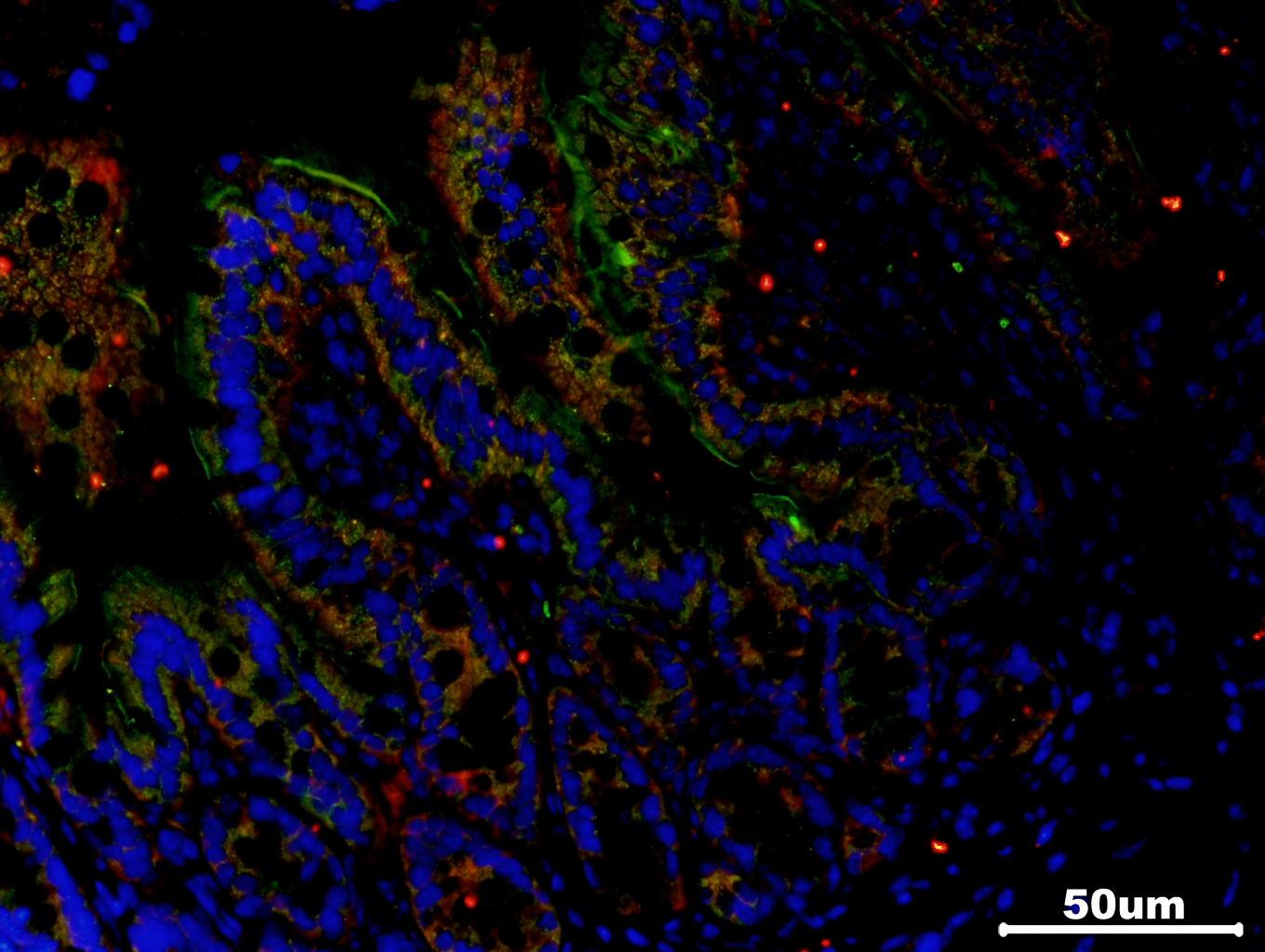

Supplement: Supplementary file 13 [file DataSheet_13.zip › E32-1-200-2-merge.tif]

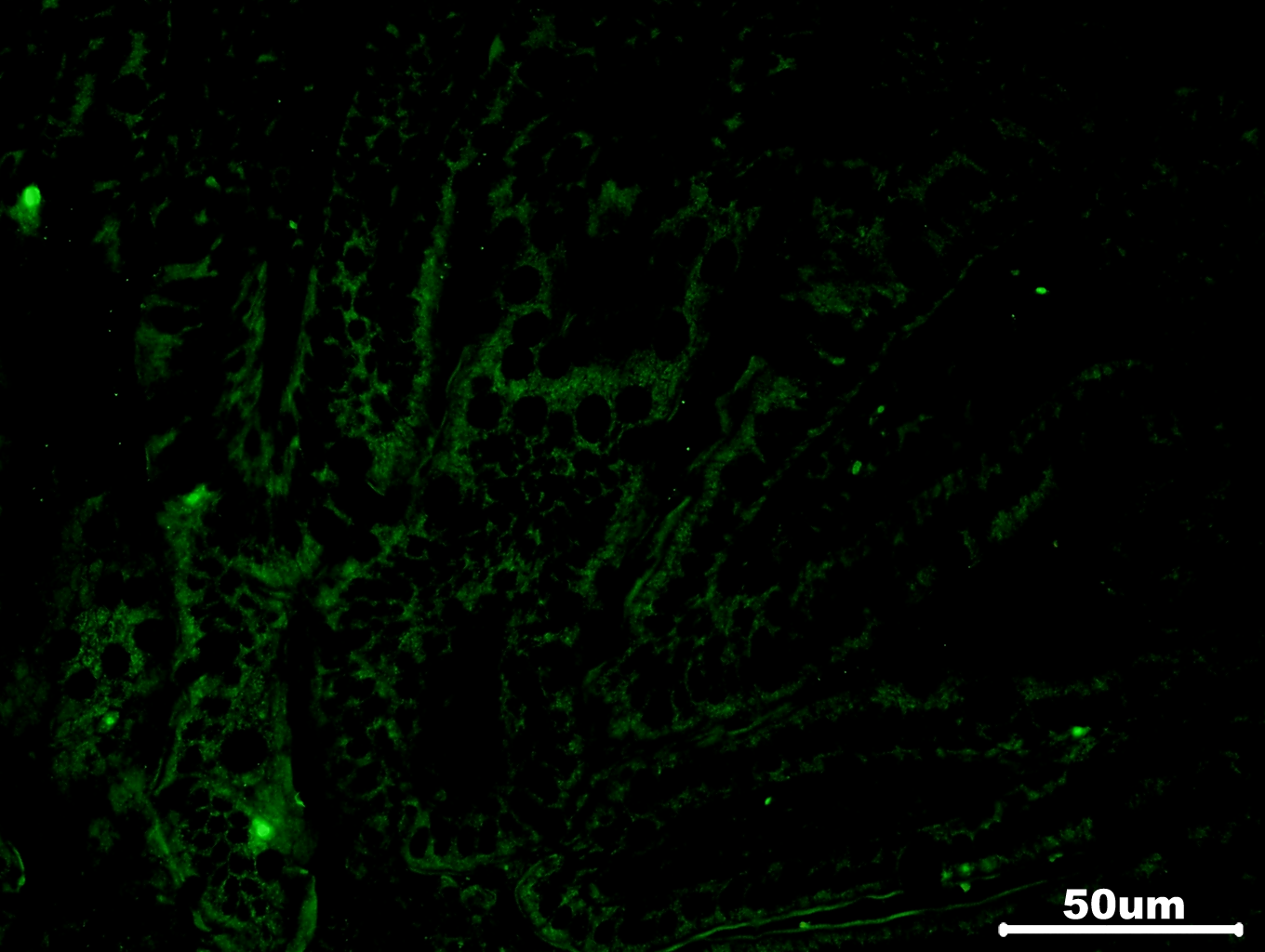

Supplement: Supplementary file 13 [file DataSheet_13.zip › E32-1-200-3-CD86.tif]

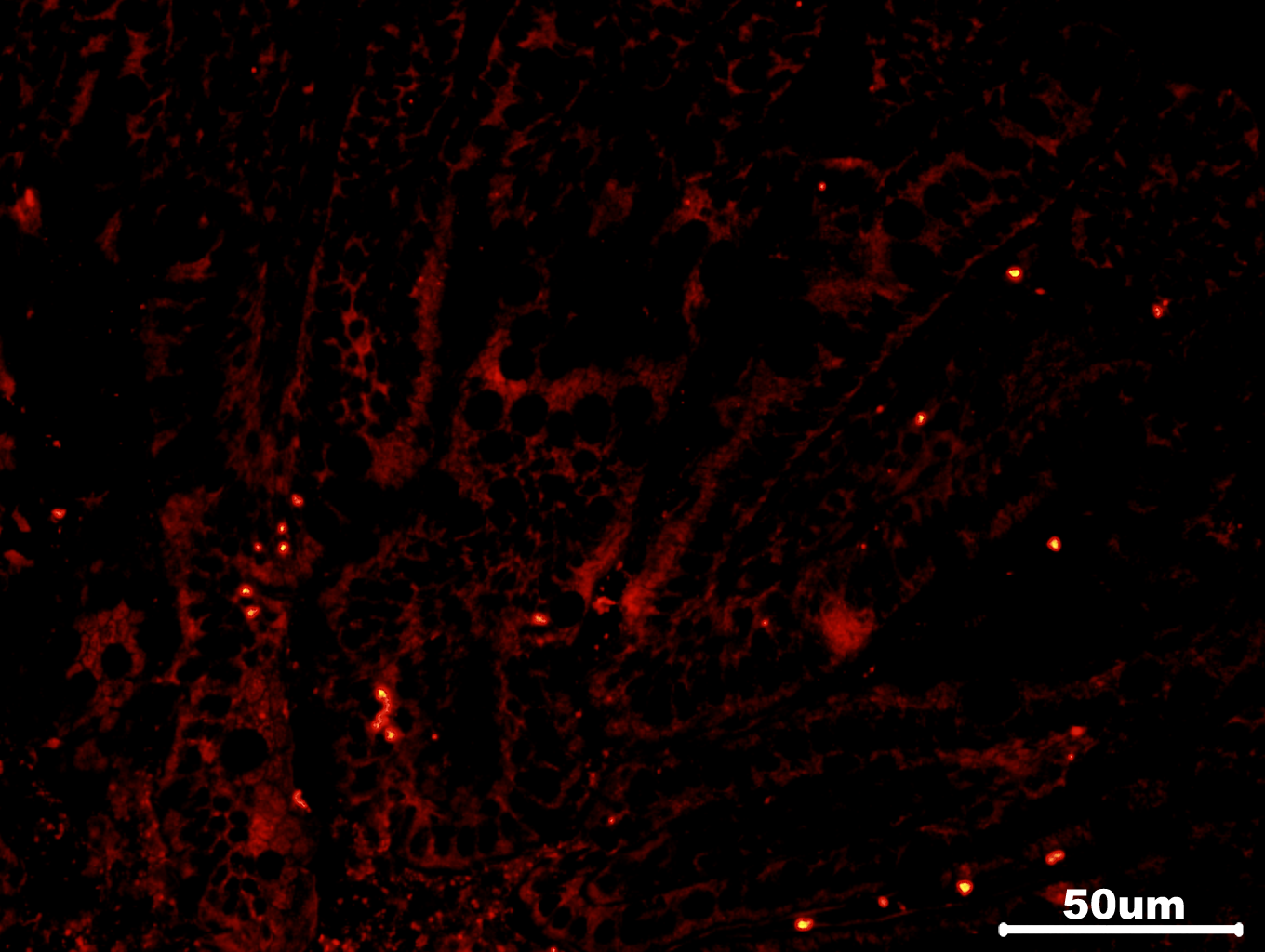

Supplement: Supplementary file 13 [file DataSheet_13.zip › E32-1-200-3-CD206.tif]

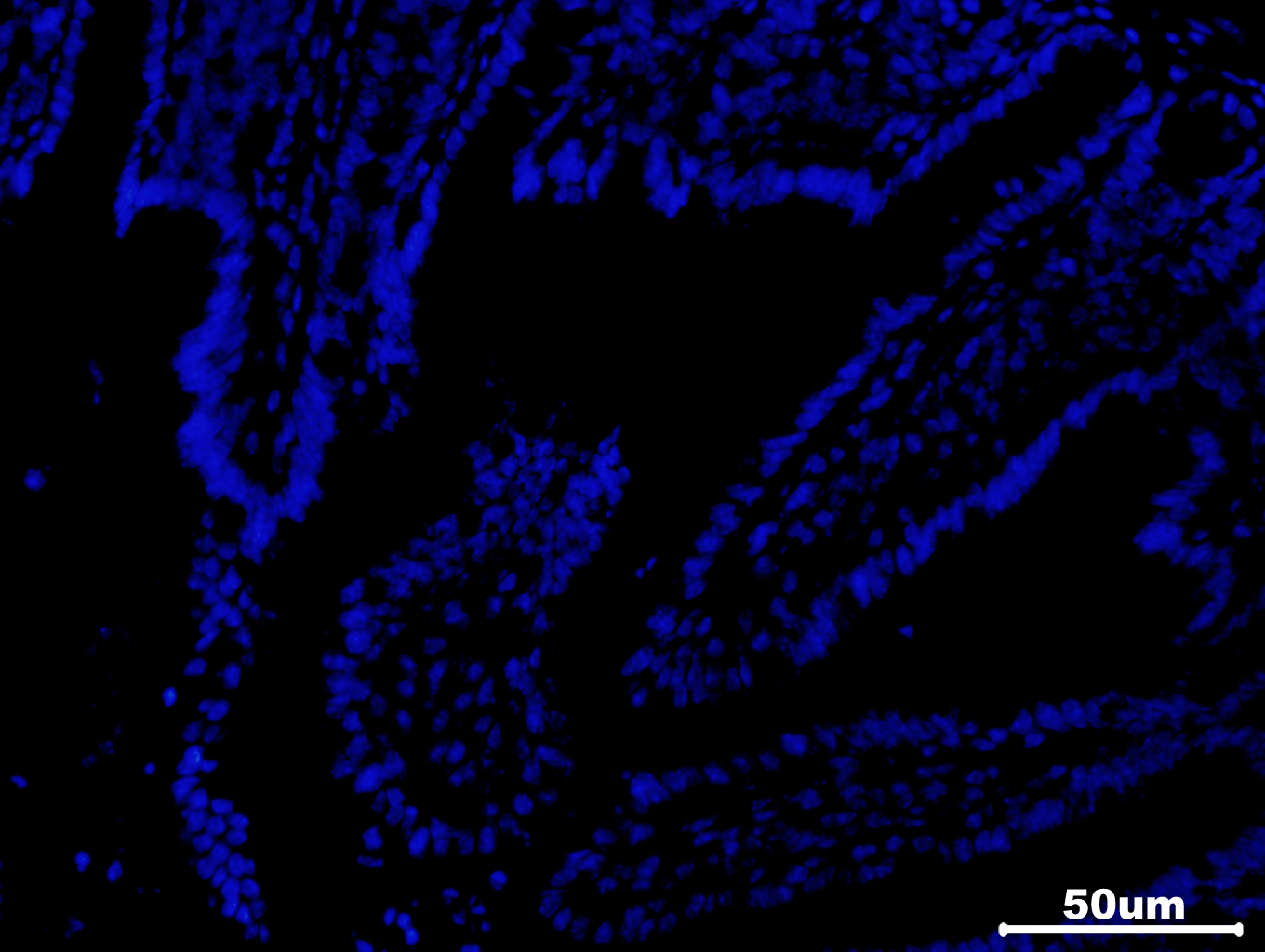

Supplement: Supplementary file 13 [file DataSheet_13.zip › E32-1-200-3-DAPI.tif]

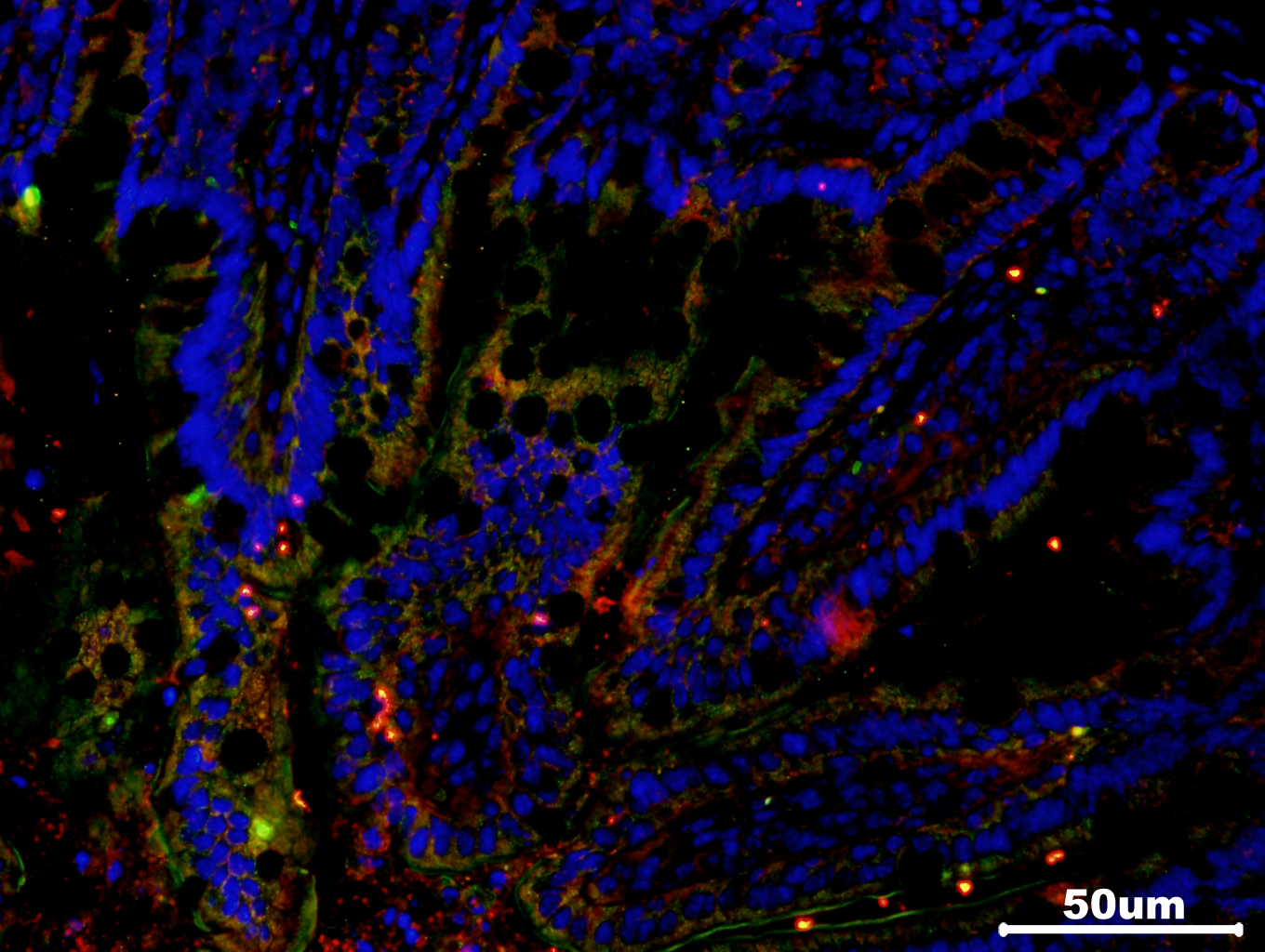

Supplement: Supplementary file 13 [file DataSheet_13.zip › E32-1-200-3-merge.tif]

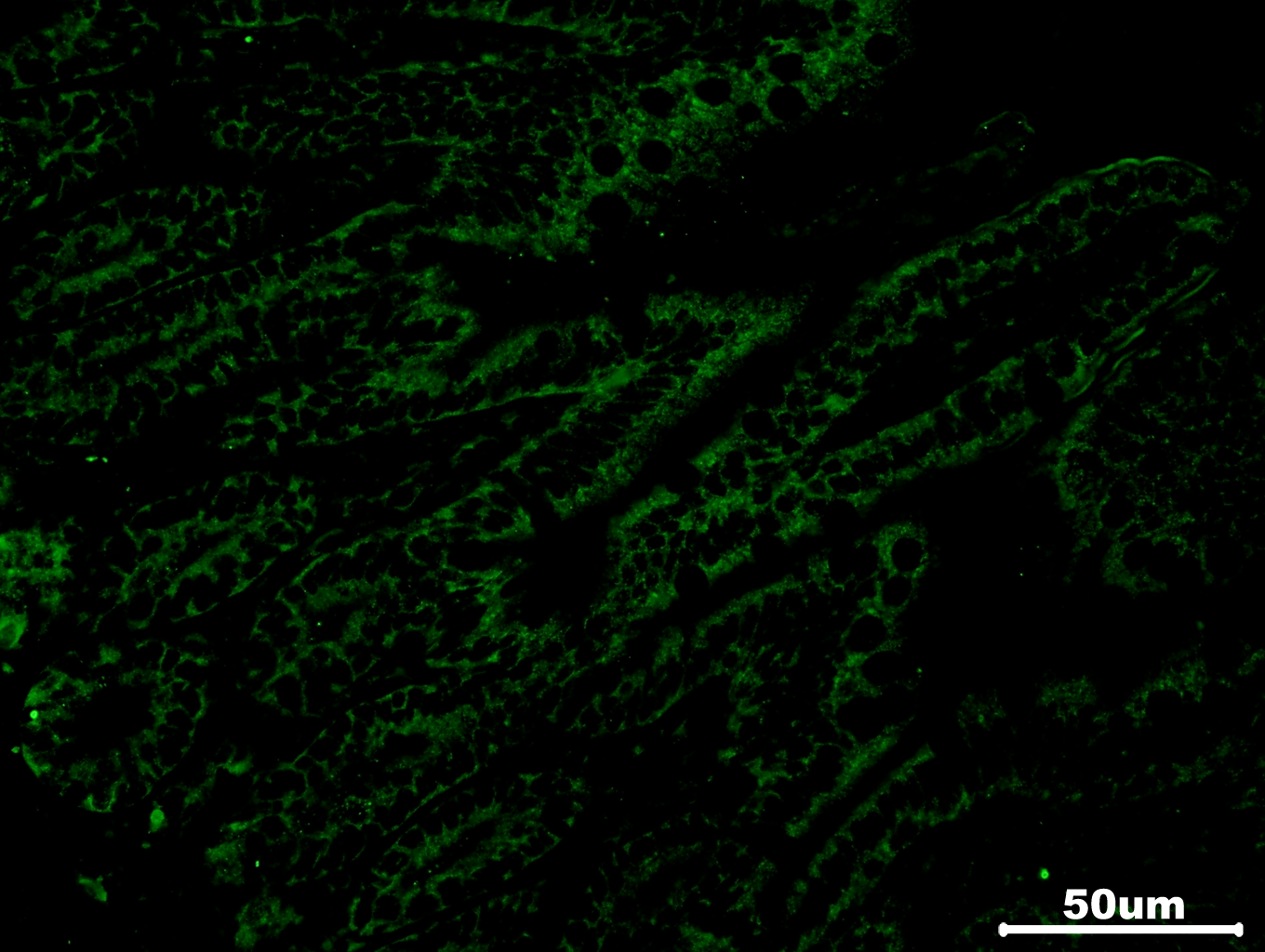

Supplement: Supplementary file 13 [file DataSheet_13.zip › E32-2-200-1-CD86.tif]

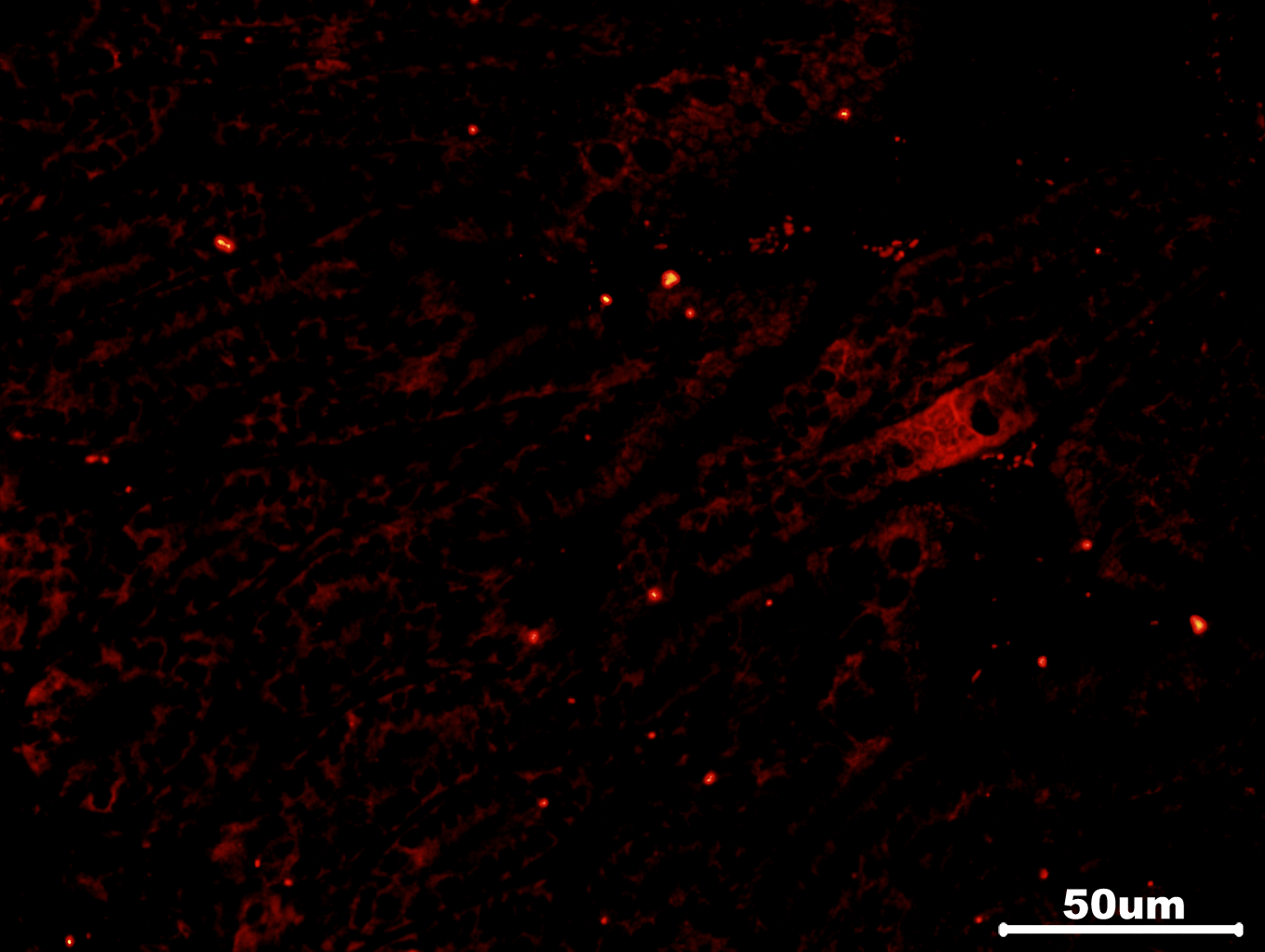

Supplement: Supplementary file 13 [file DataSheet_13.zip › E32-2-200-1-CD206.tif]

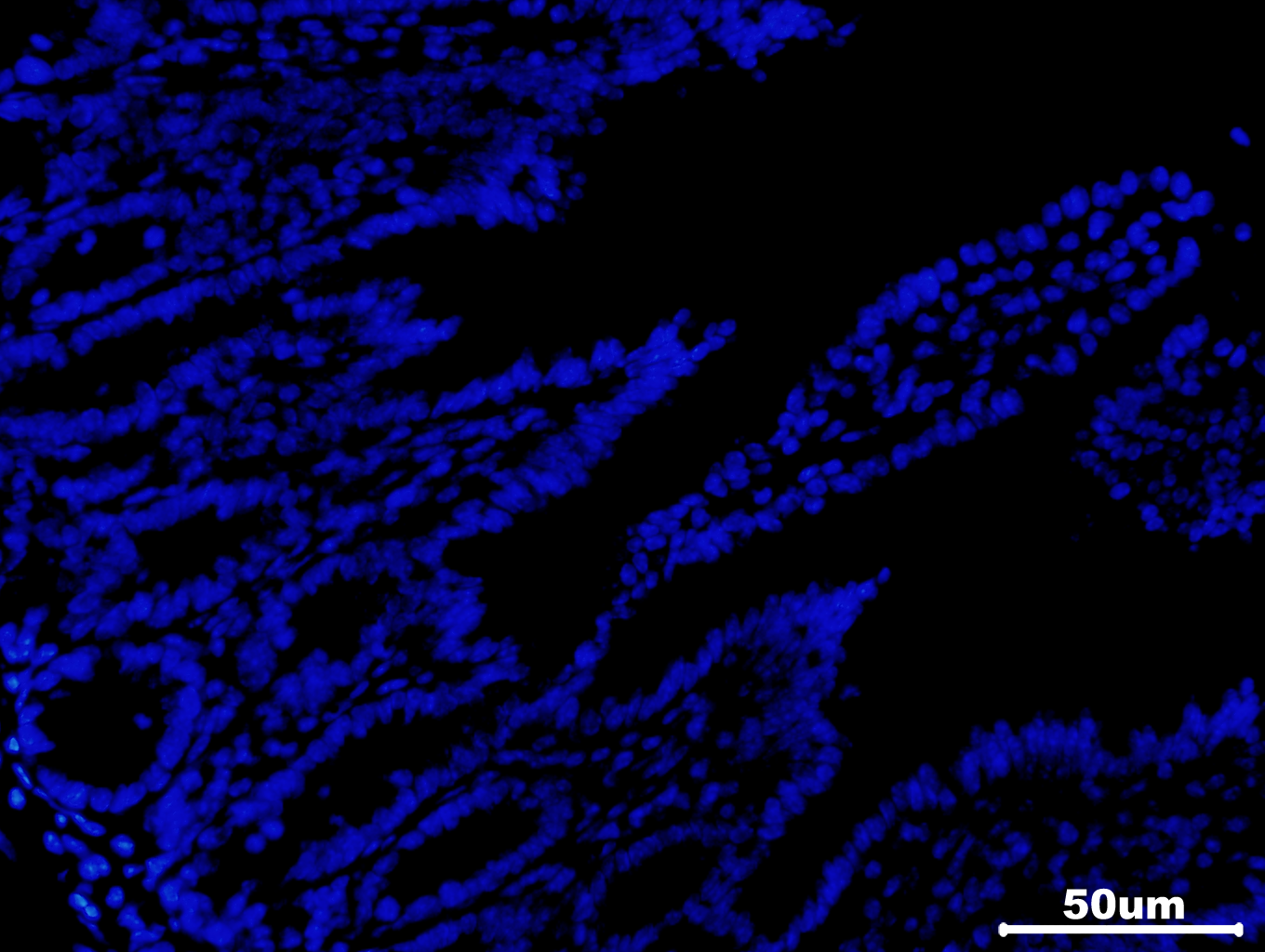

Supplement: Supplementary file 13 [file DataSheet_13.zip › E32-2-200-1-DAPI.tif]

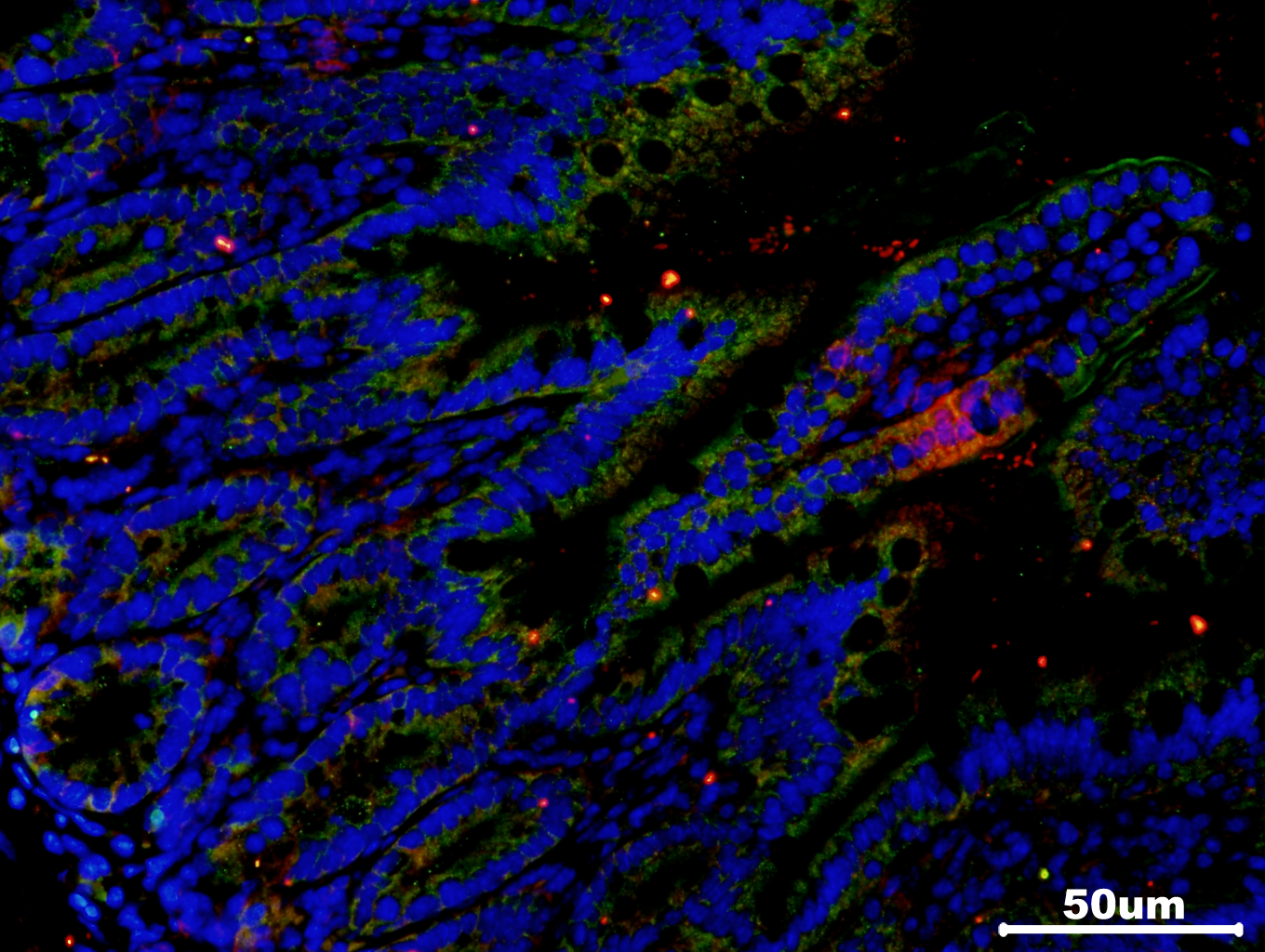

Supplement: Supplementary file 13 [file DataSheet_13.zip › E32-2-200-1-merge.tif]

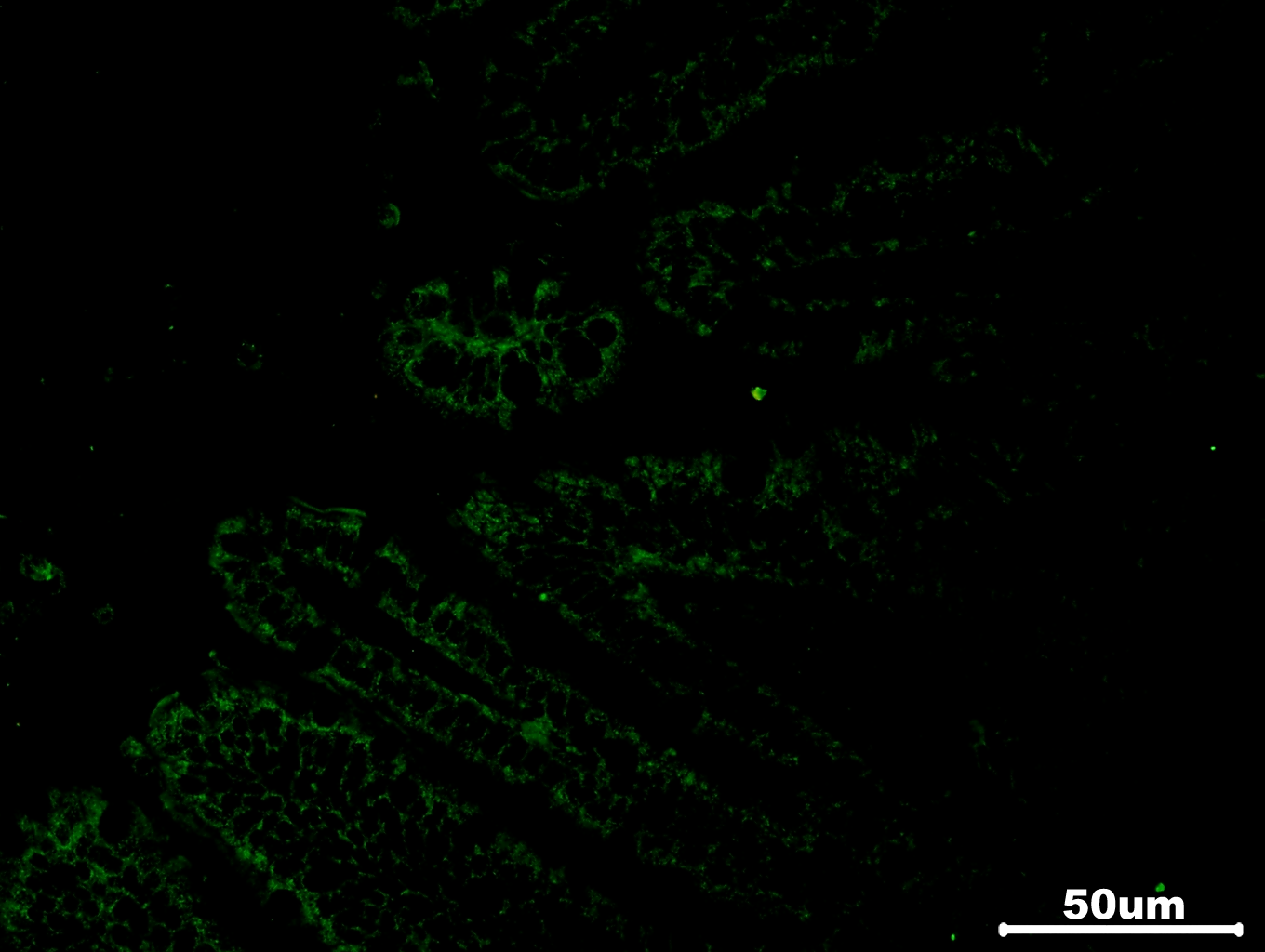

Supplement: Supplementary file 13 [file DataSheet_13.zip › E32-2-200-2-CD86.tif]

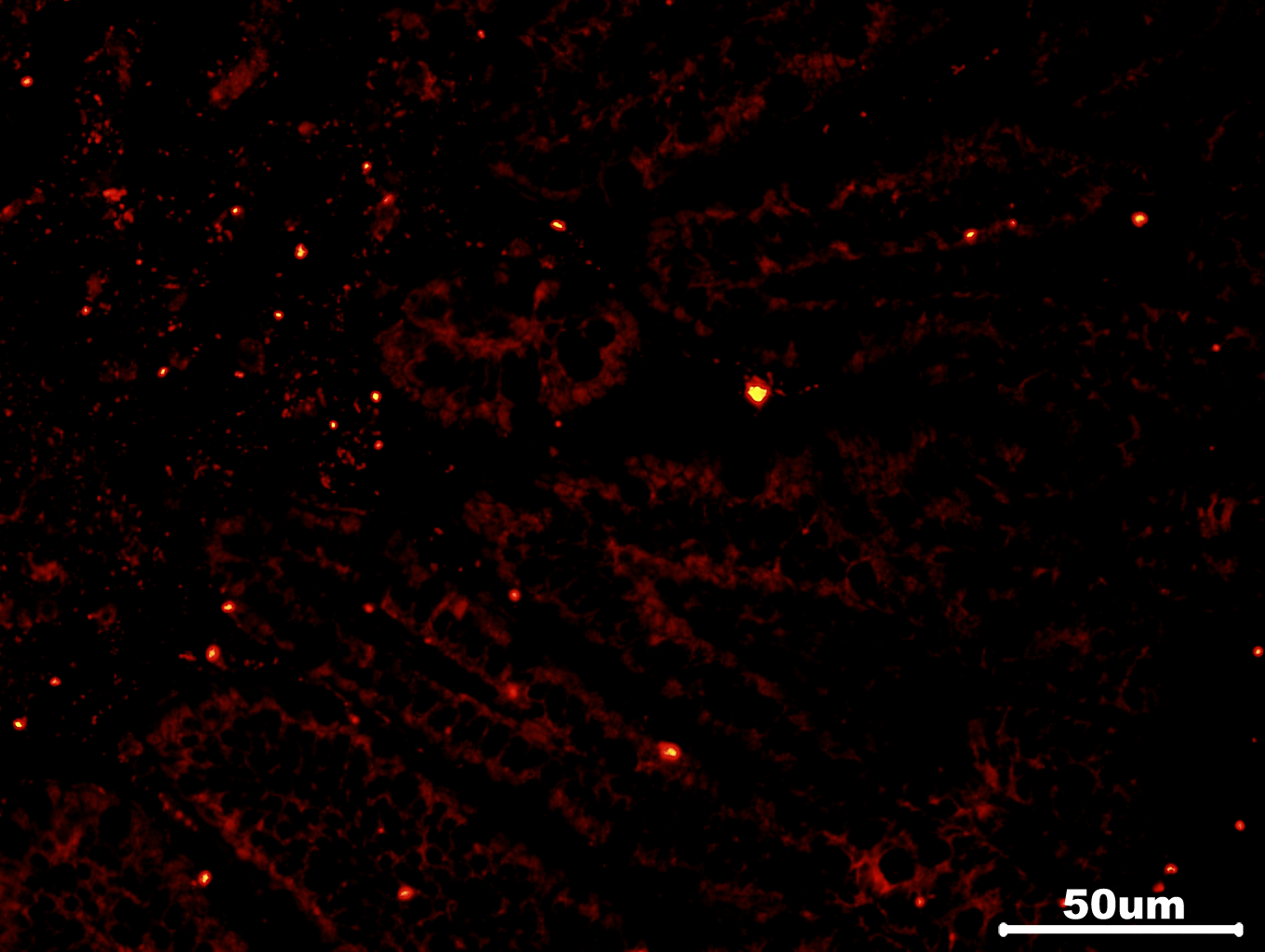

Supplement: Supplementary file 13 [file DataSheet_13.zip › E32-2-200-2-CD206.tif]

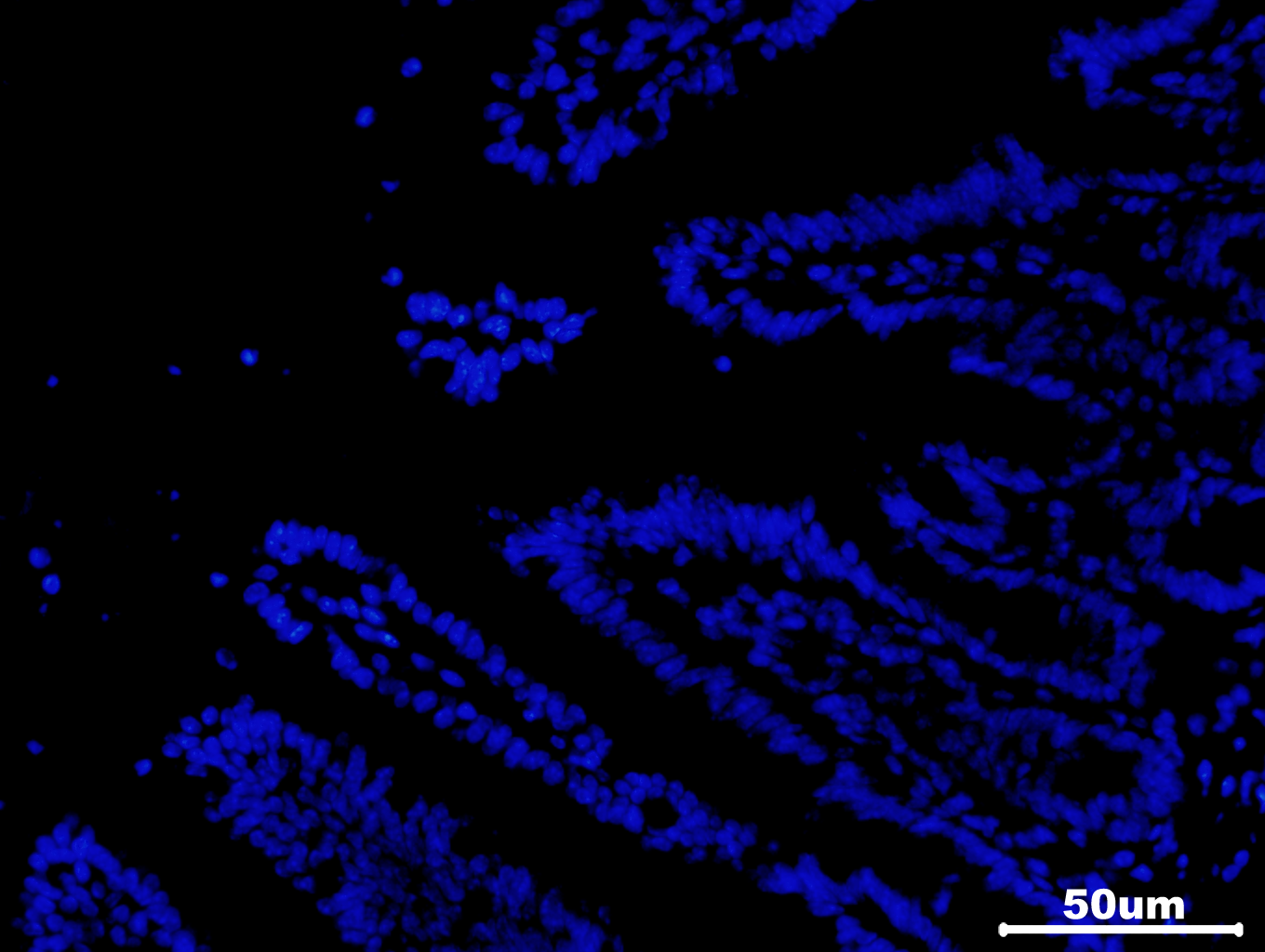

Supplement: Supplementary file 13 [file DataSheet_13.zip › E32-2-200-2-DAPI.tif]

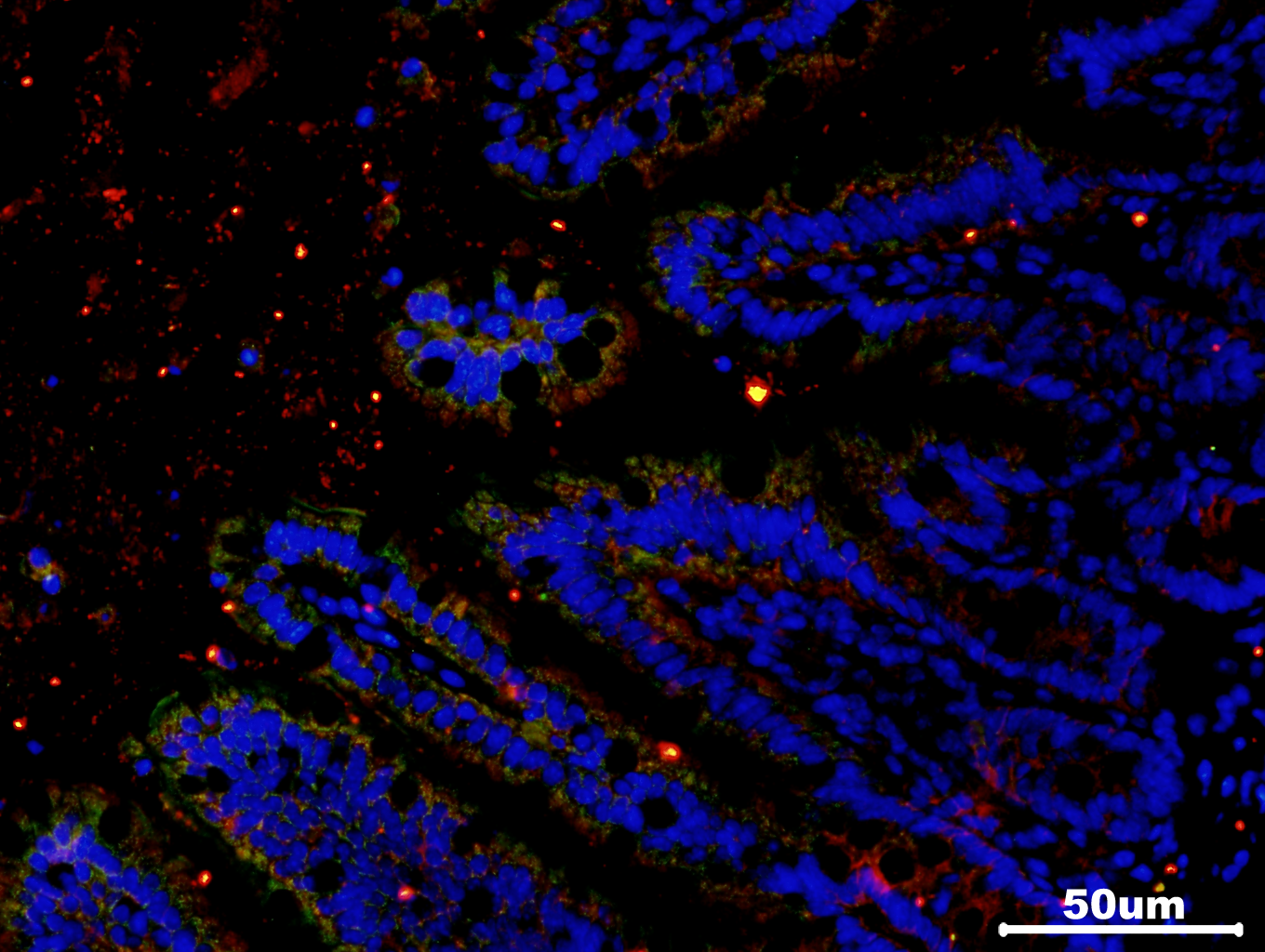

Supplement: Supplementary file 13 [file DataSheet_13.zip › E32-2-200-2-merge.tif]

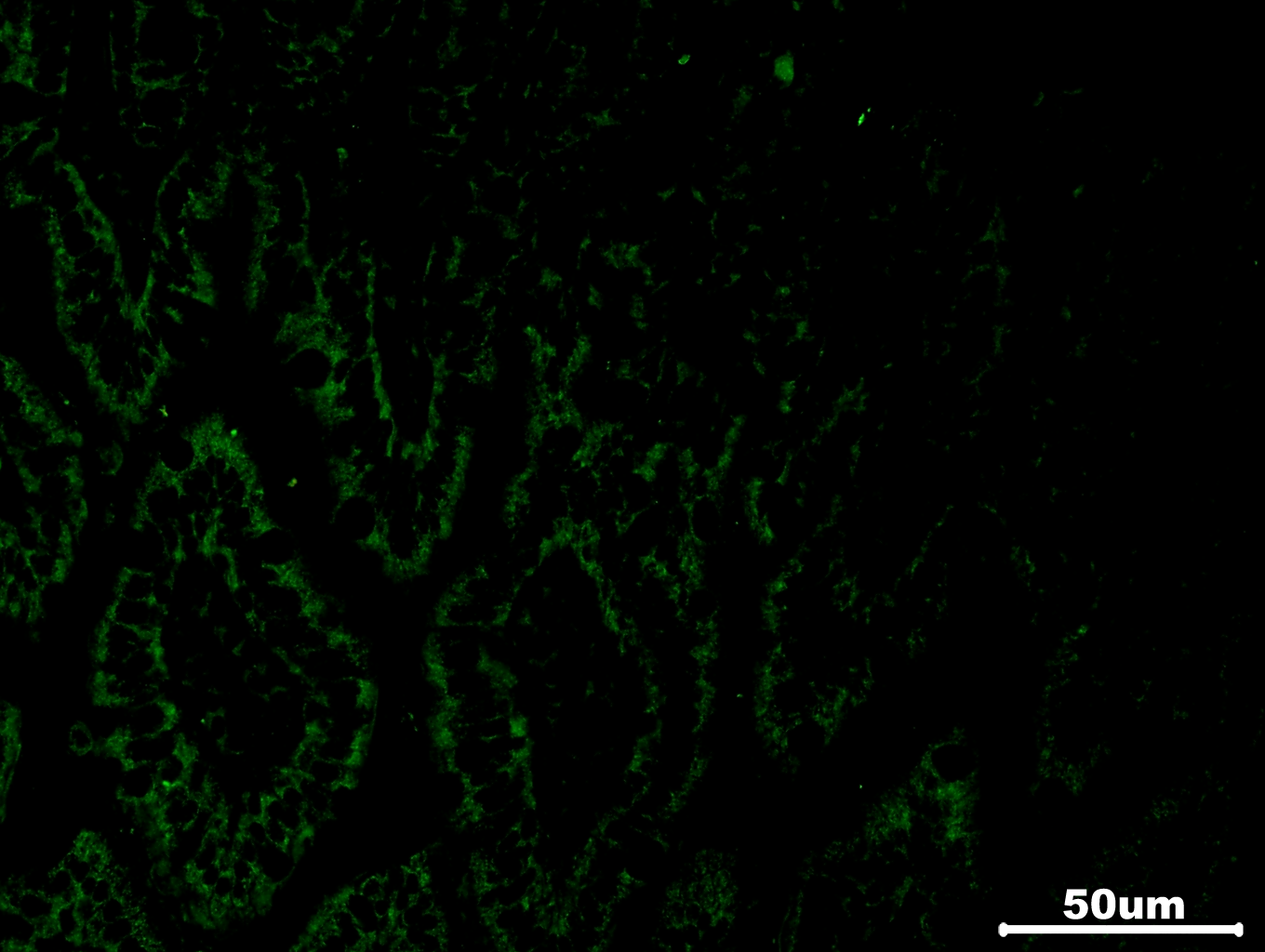

Supplement: Supplementary file 13 [file DataSheet_13.zip › E32-2-200-3-CD86.tif]

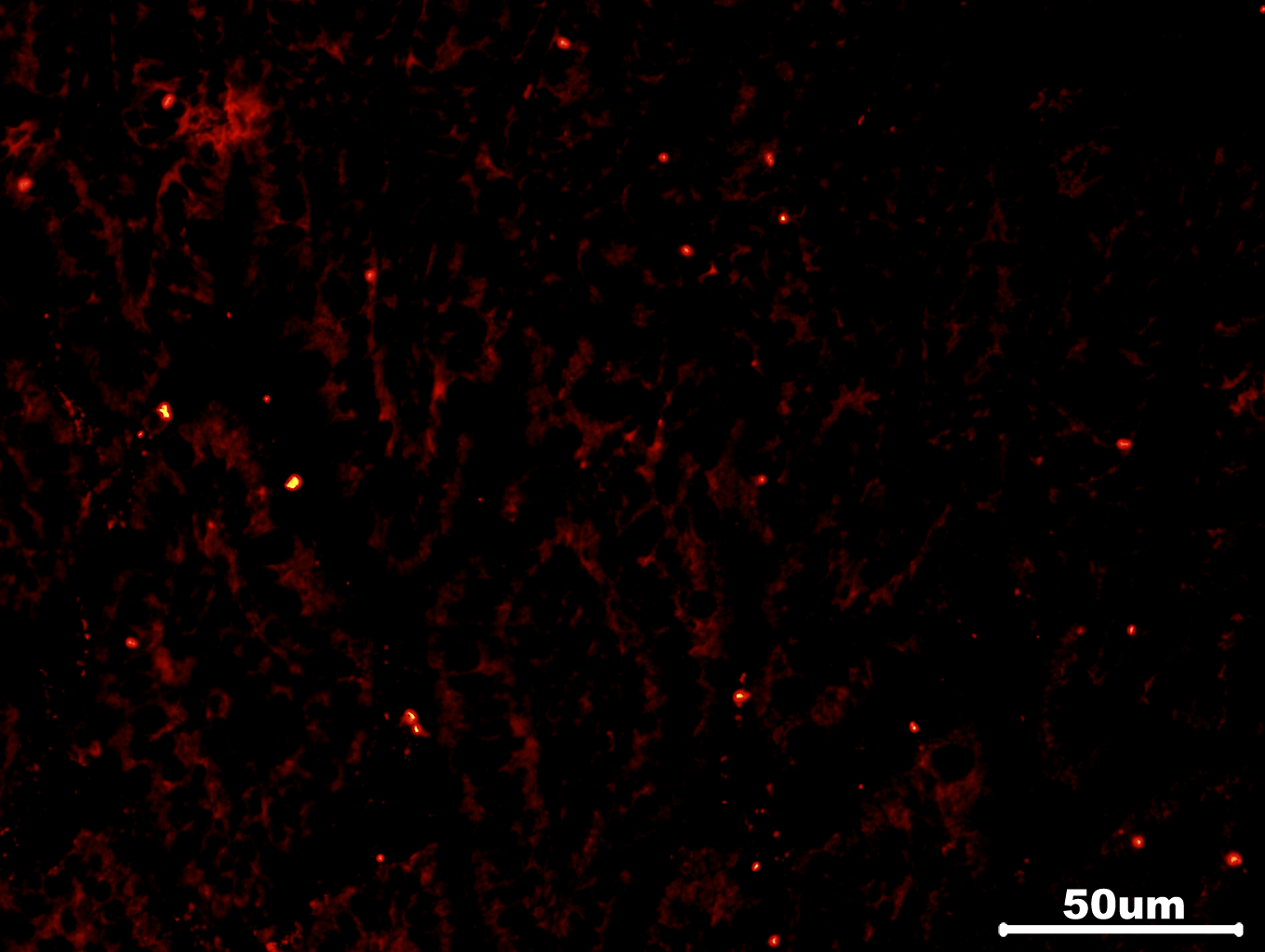

Supplement: Supplementary file 13 [file DataSheet_13.zip › E32-2-200-3-CD206.tif]

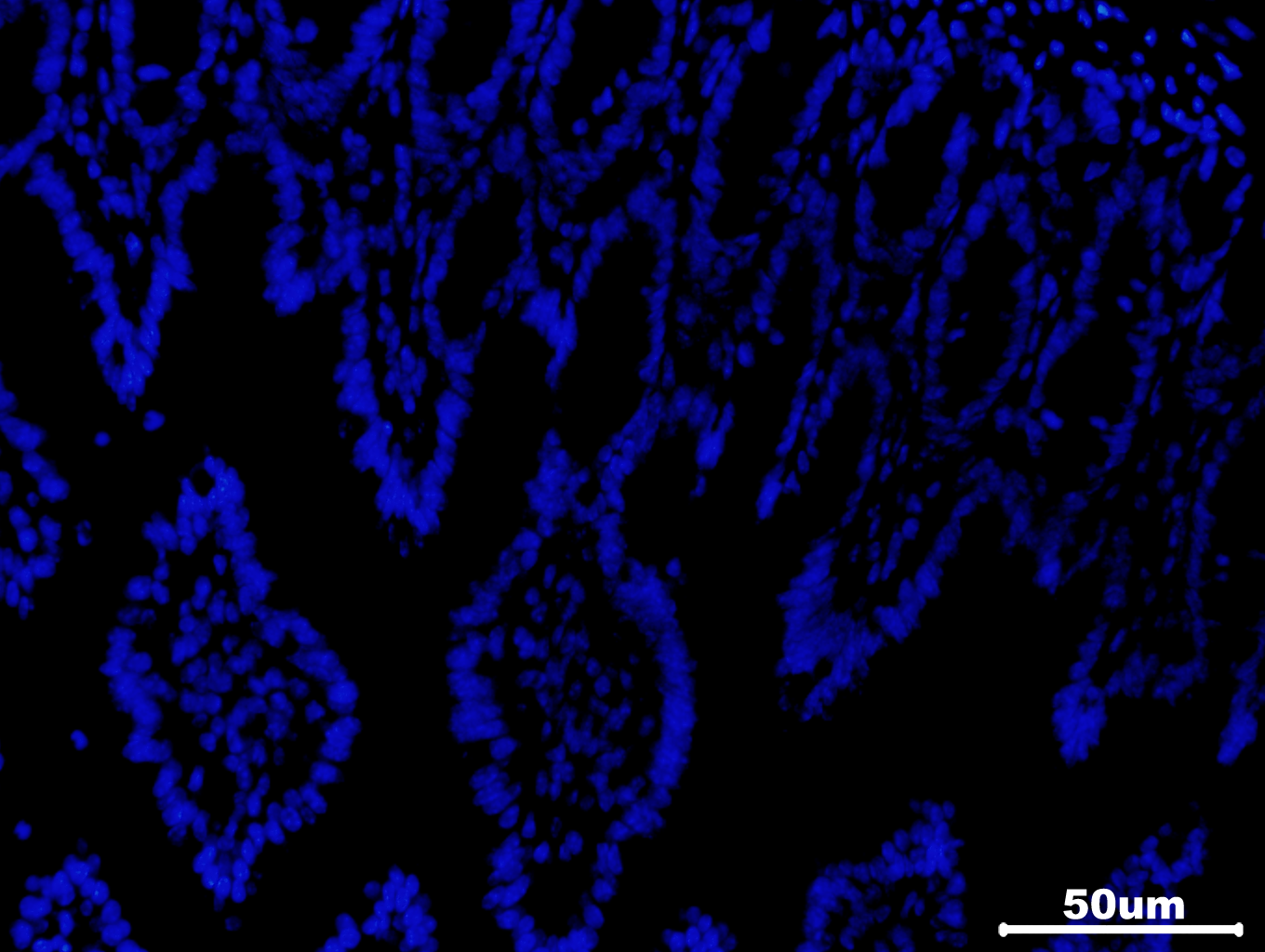

Supplement: Supplementary file 13 [file DataSheet_13.zip › E32-2-200-3-DAPI.tif]

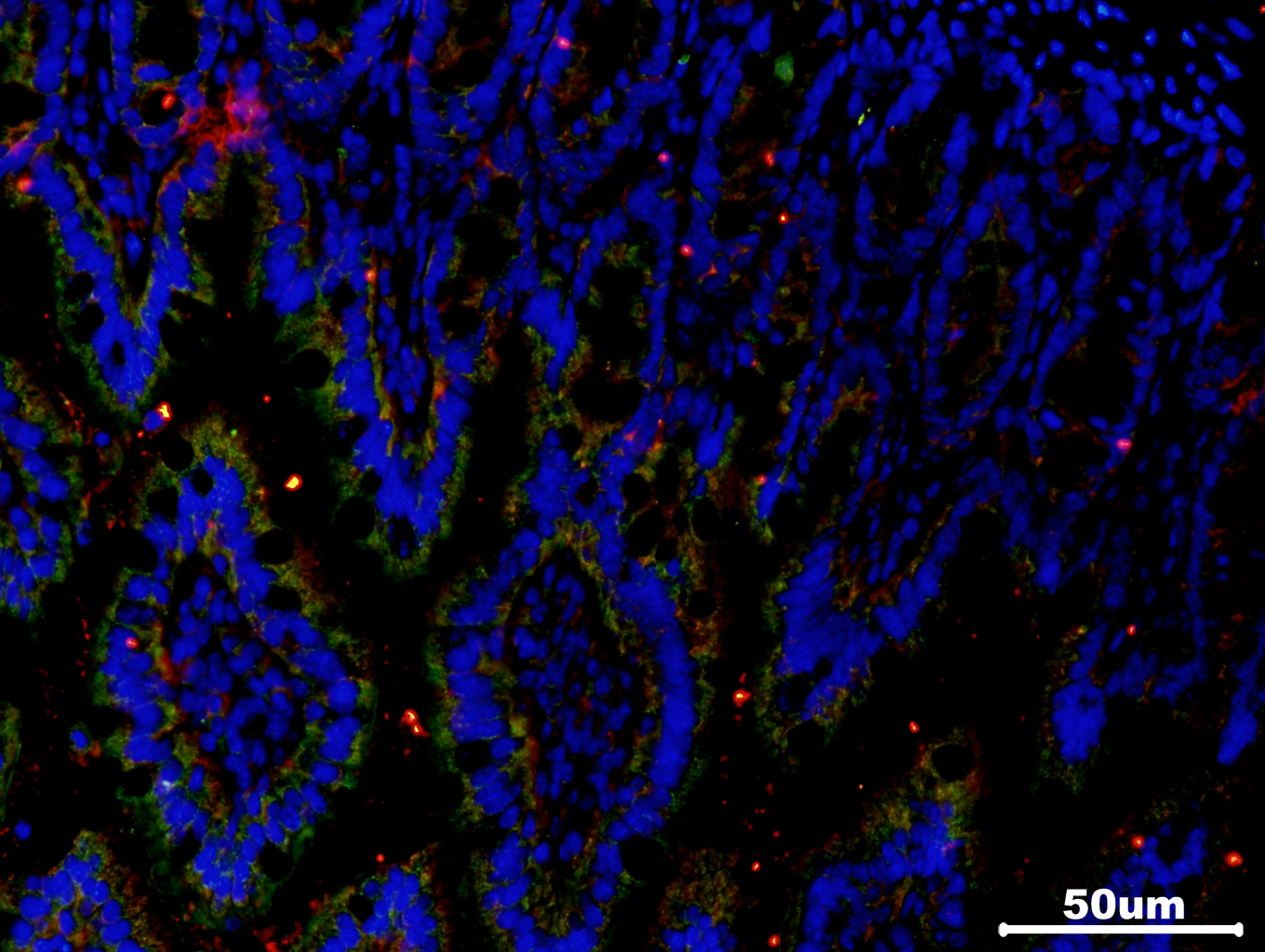

Supplement: Supplementary file 13 [file DataSheet_13.zip › E32-2-200-3-merge.tif]

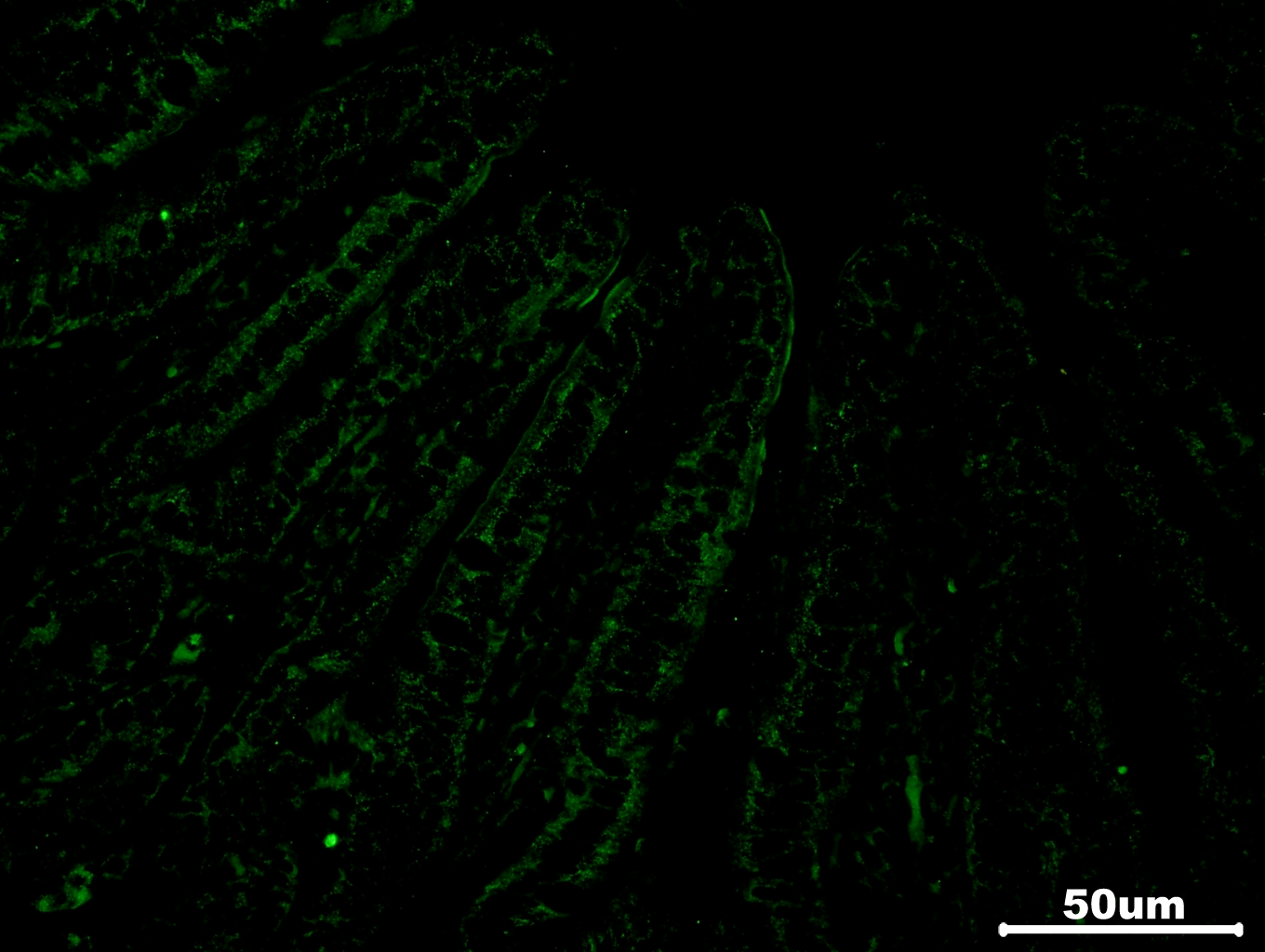

Supplement: Supplementary file 13 [file DataSheet_13.zip › E33-1-200-1-CD86.tif]

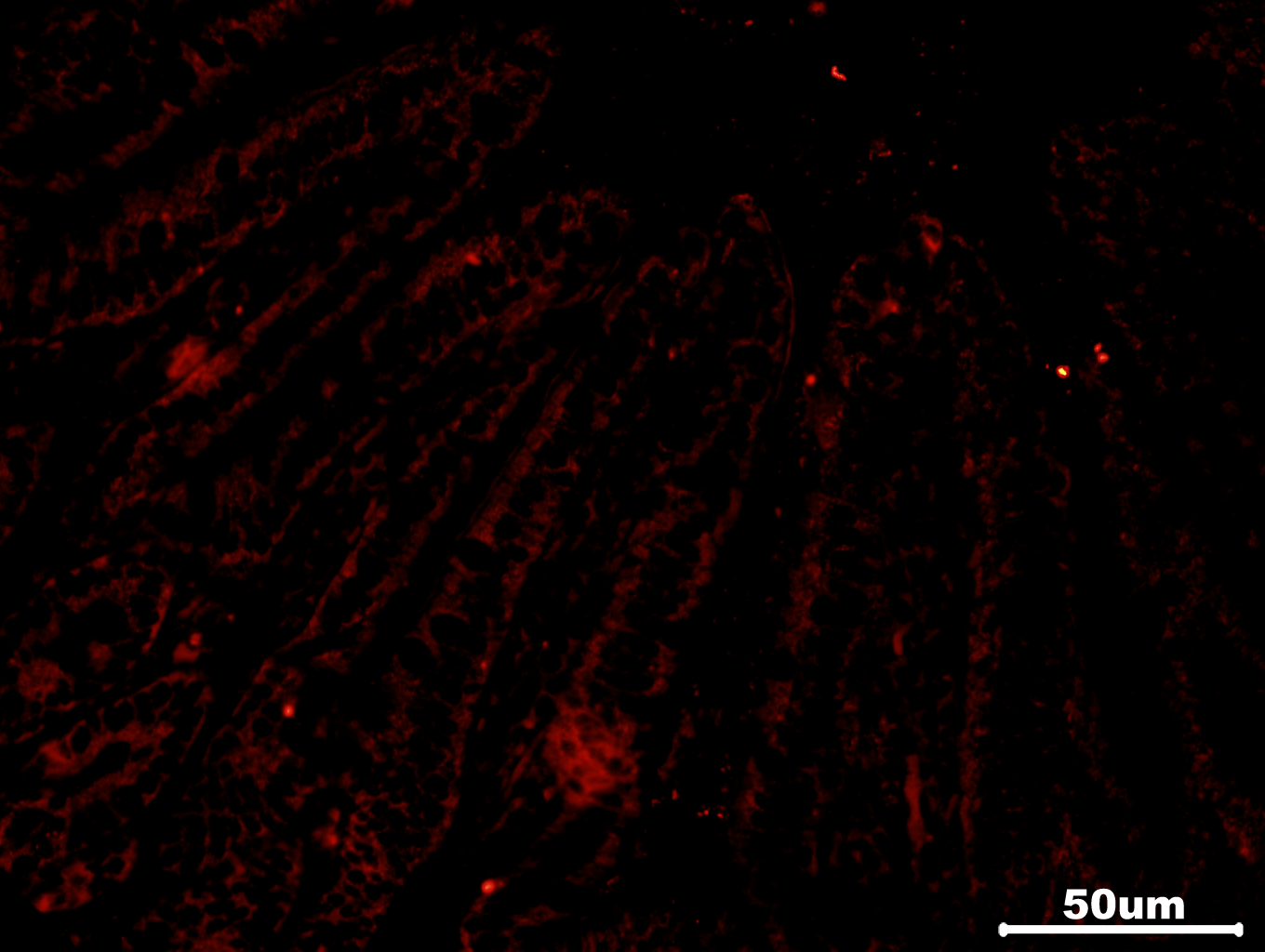

Supplement: Supplementary file 13 [file DataSheet_13.zip › E33-1-200-1-CD206.tif]

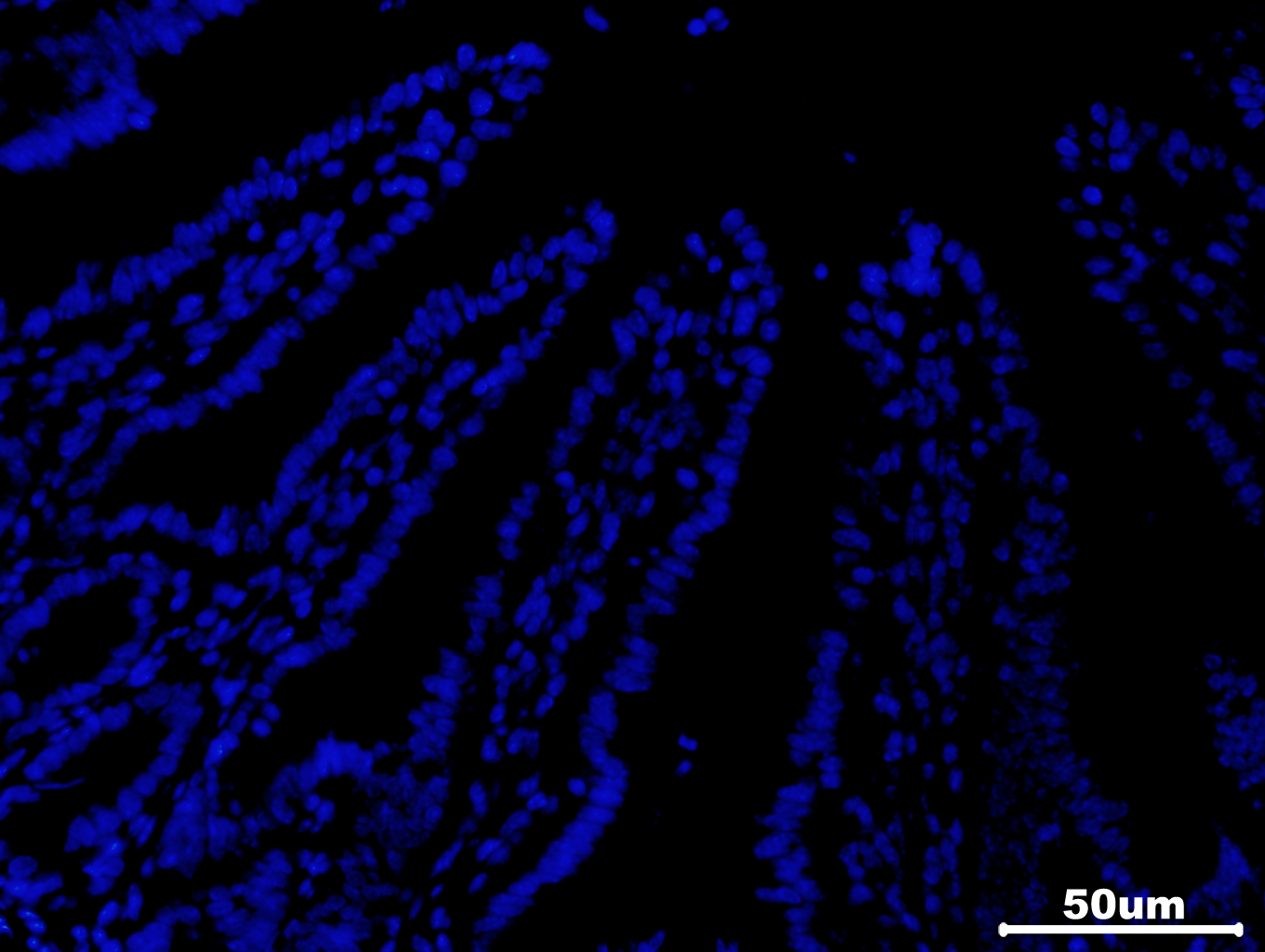

Supplement: Supplementary file 14 [file DataSheet_14.zip › E33-1-200-1-DAPI.tif]

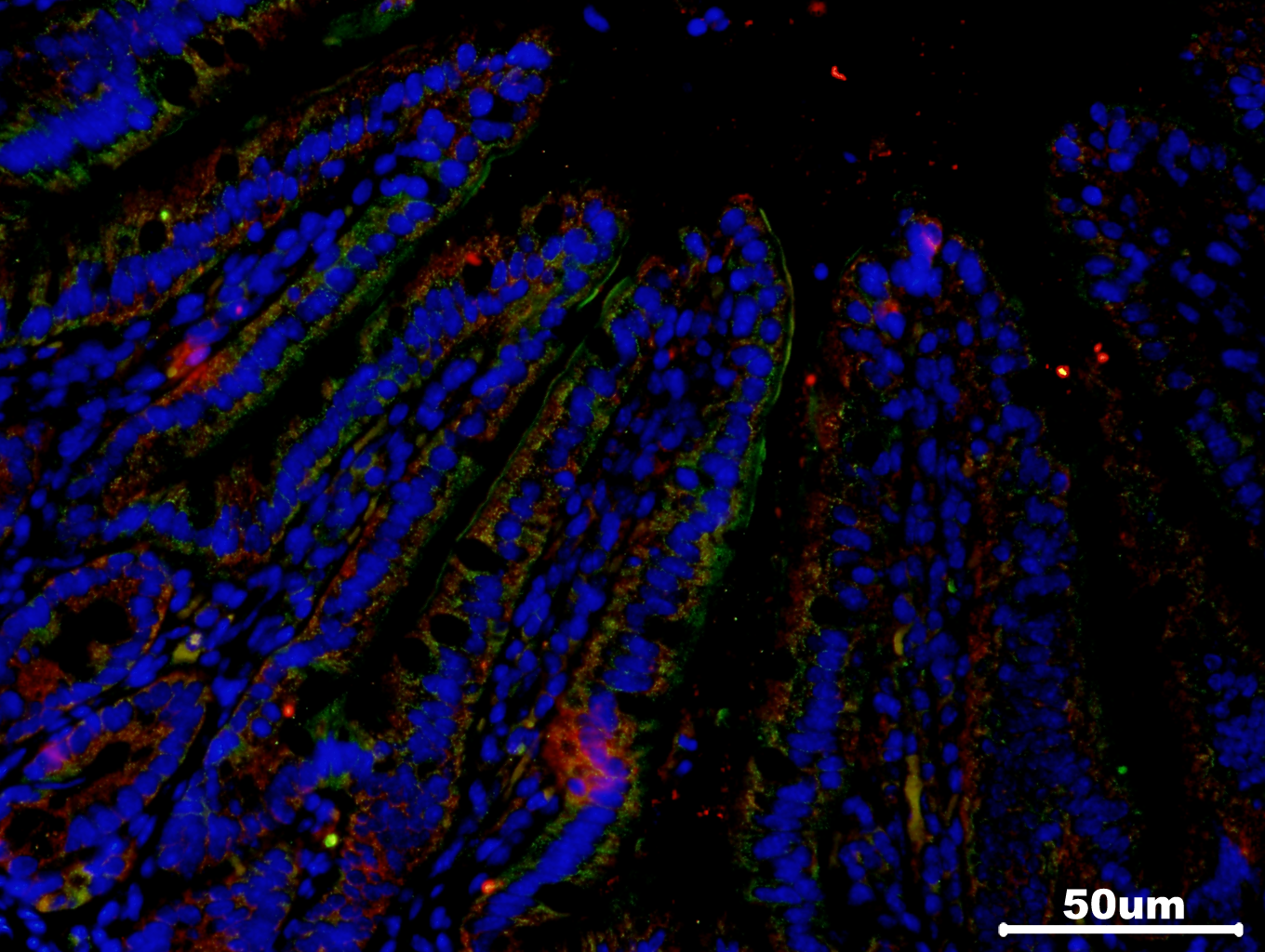

Supplement: Supplementary file 14 [file DataSheet_14.zip › E33-1-200-1-merge.tif]

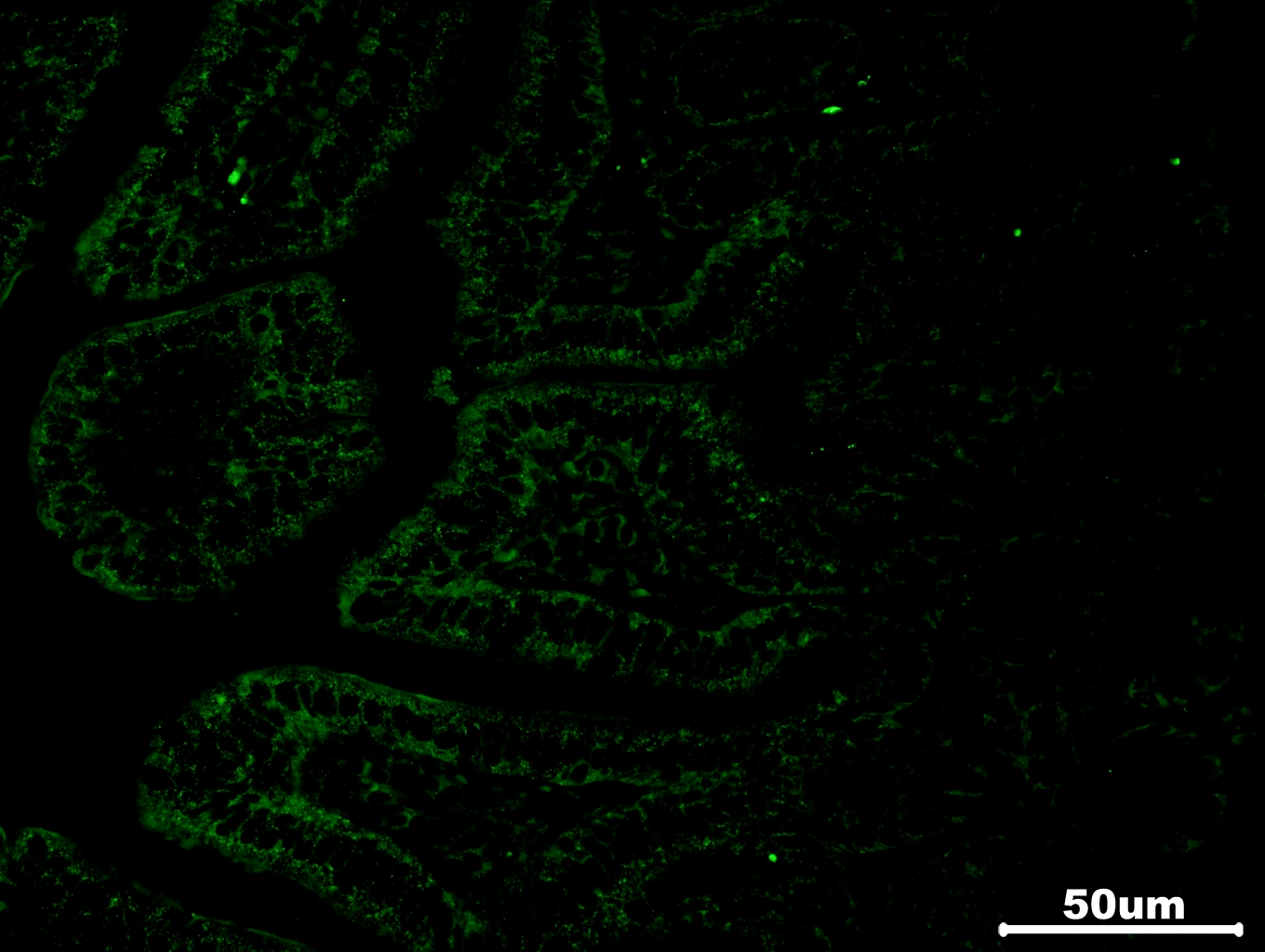

Supplement: Supplementary file 14 [file DataSheet_14.zip › E33-1-200-2-CD86.tif]

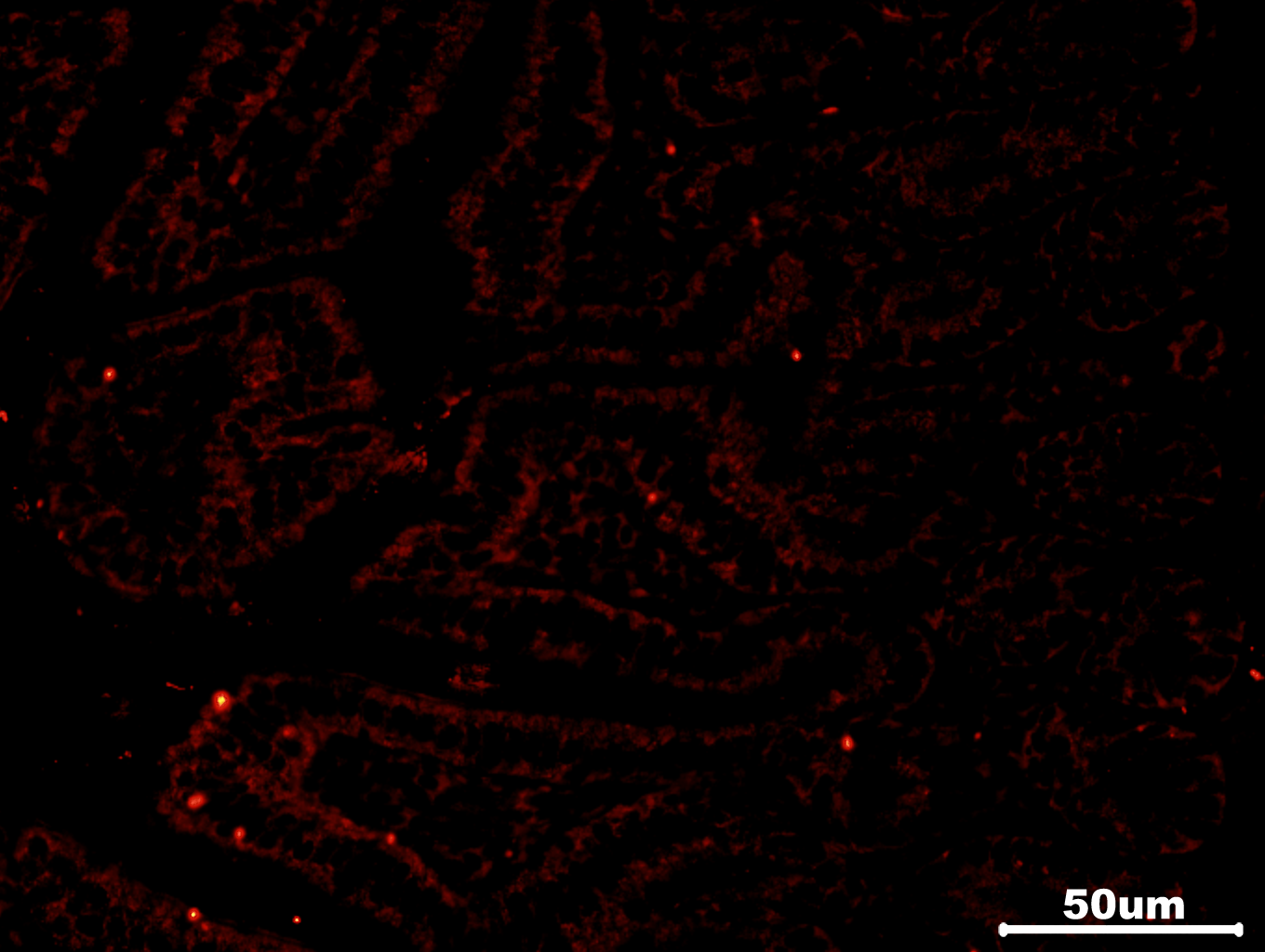

Supplement: Supplementary file 14 [file DataSheet_14.zip › E33-1-200-2-CD206.tif]

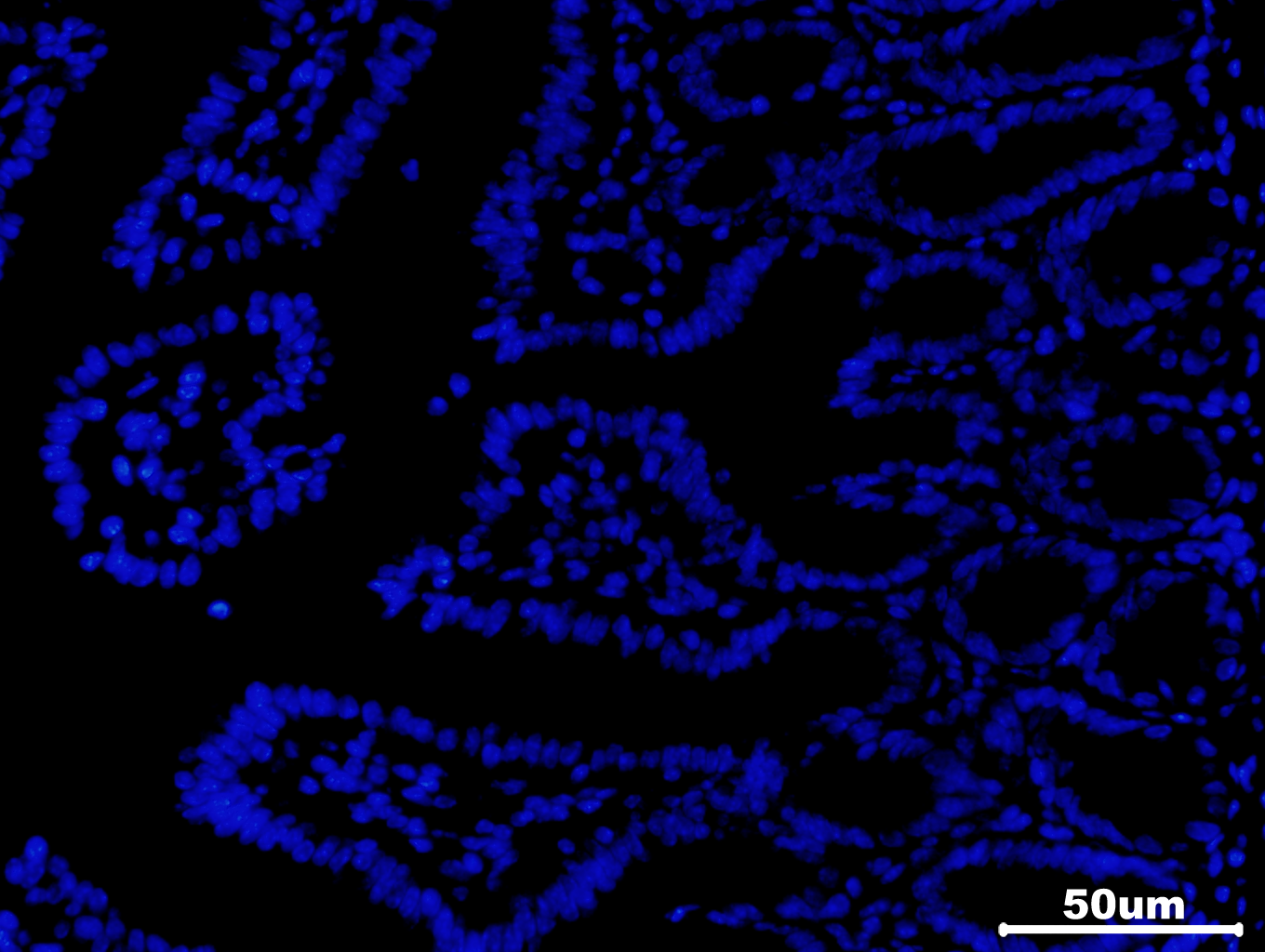

Supplement: Supplementary file 14 [file DataSheet_14.zip › E33-1-200-2-DAPI.tif]

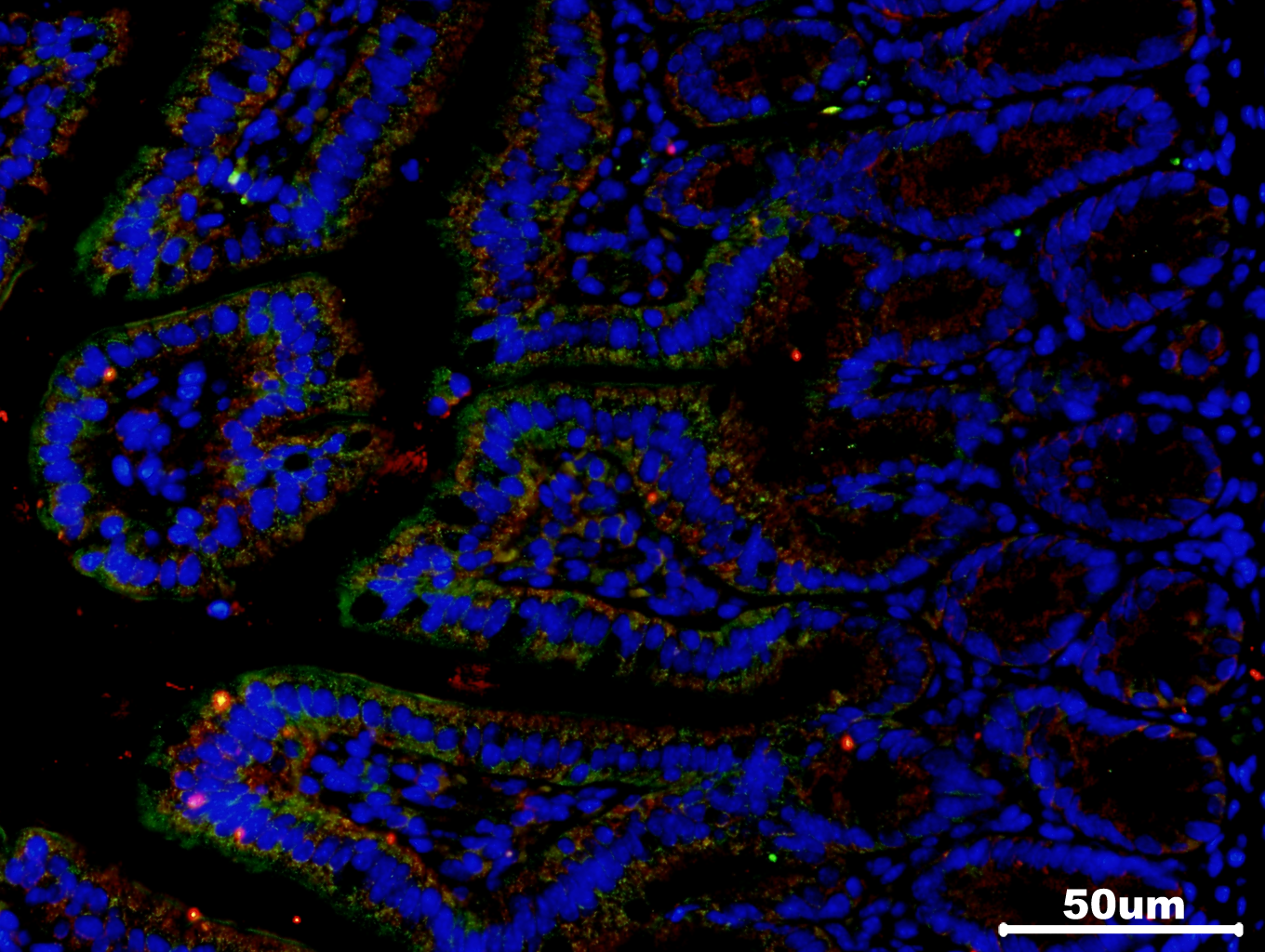

Supplement: Supplementary file 14 [file DataSheet_14.zip › E33-1-200-2-merge.tif]

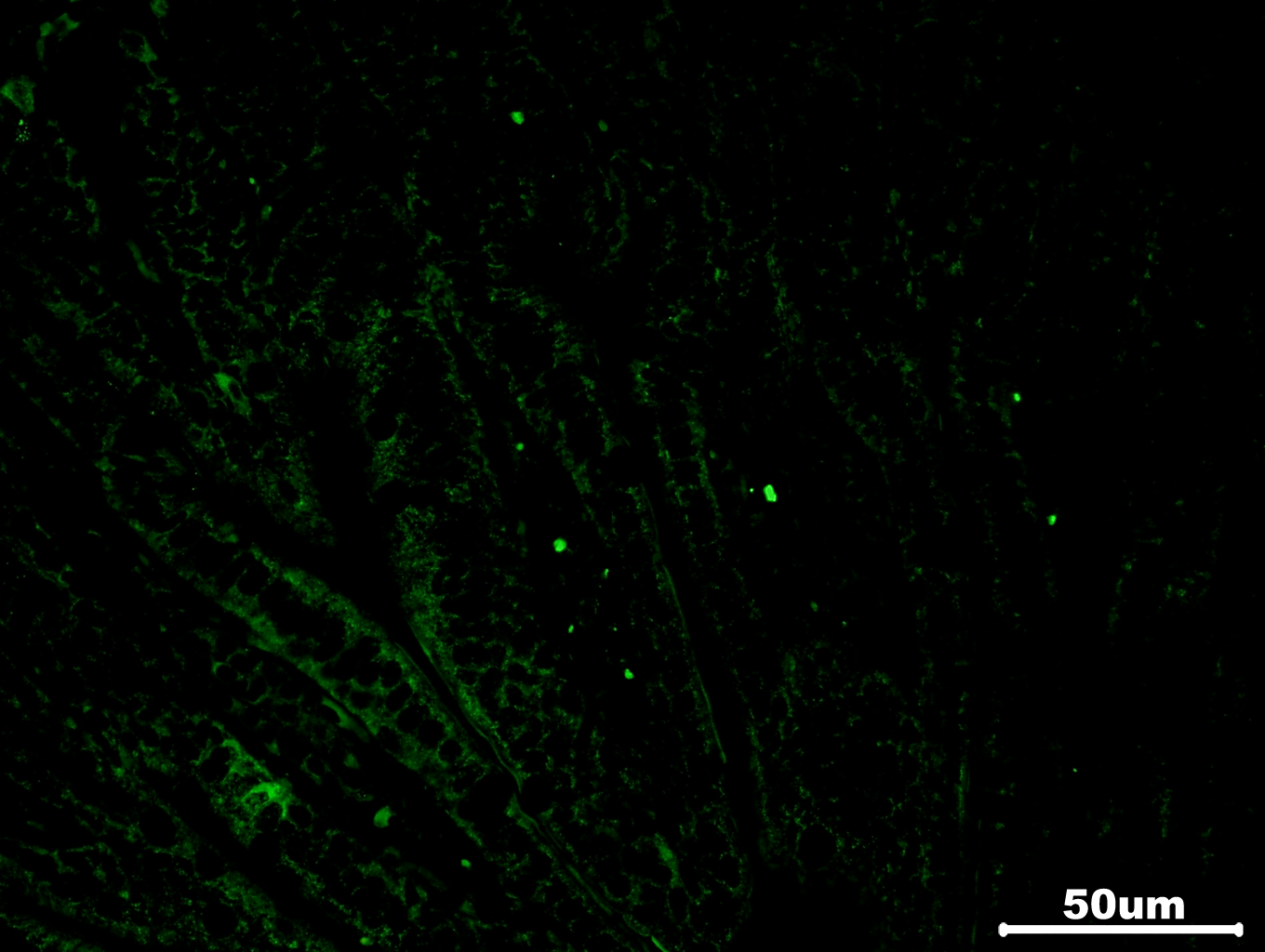

Supplement: Supplementary file 14 [file DataSheet_14.zip › E33-1-200-3-CD86.tif]

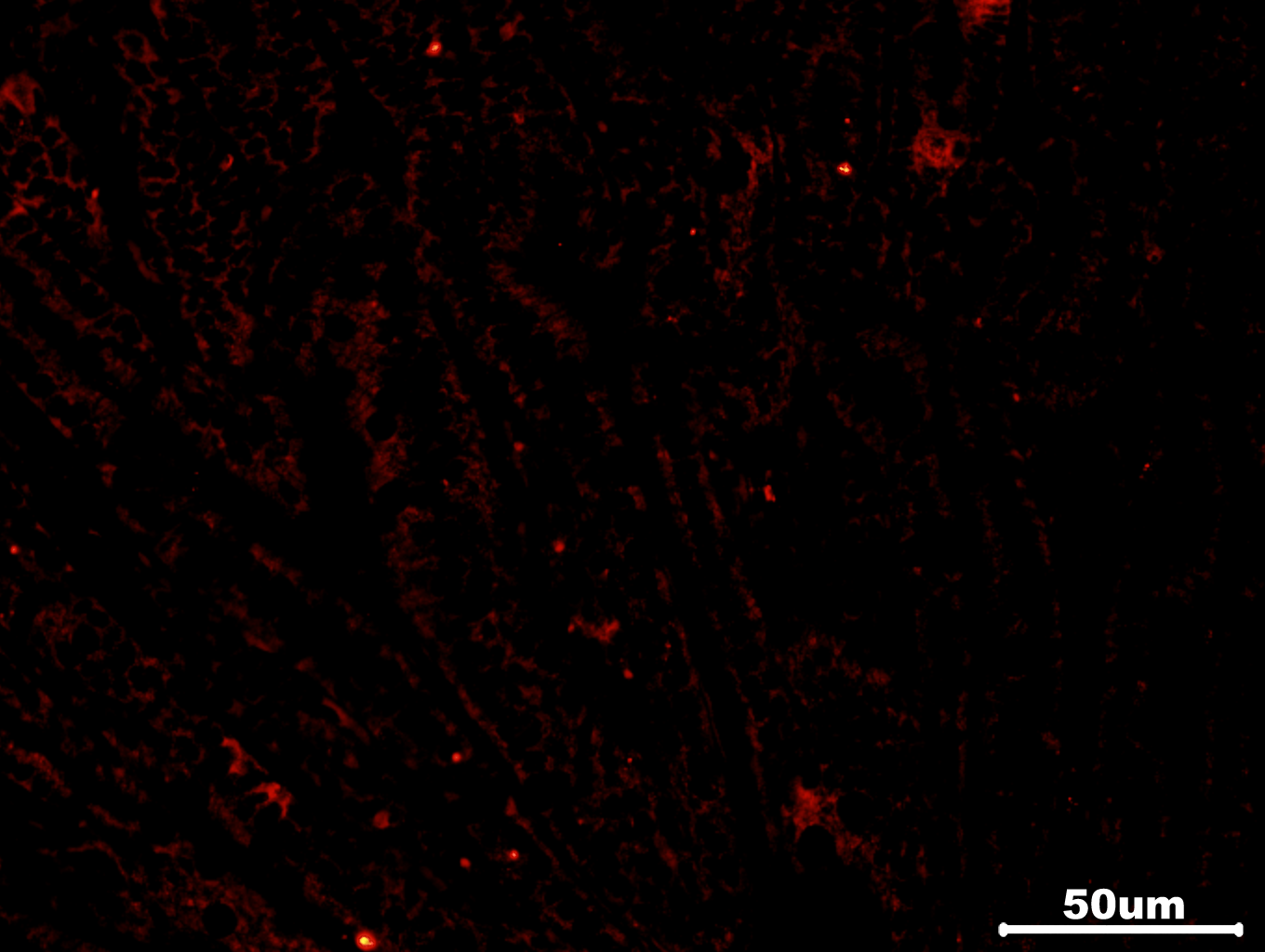

Supplement: Supplementary file 14 [file DataSheet_14.zip › E33-1-200-3-CD206.tif]

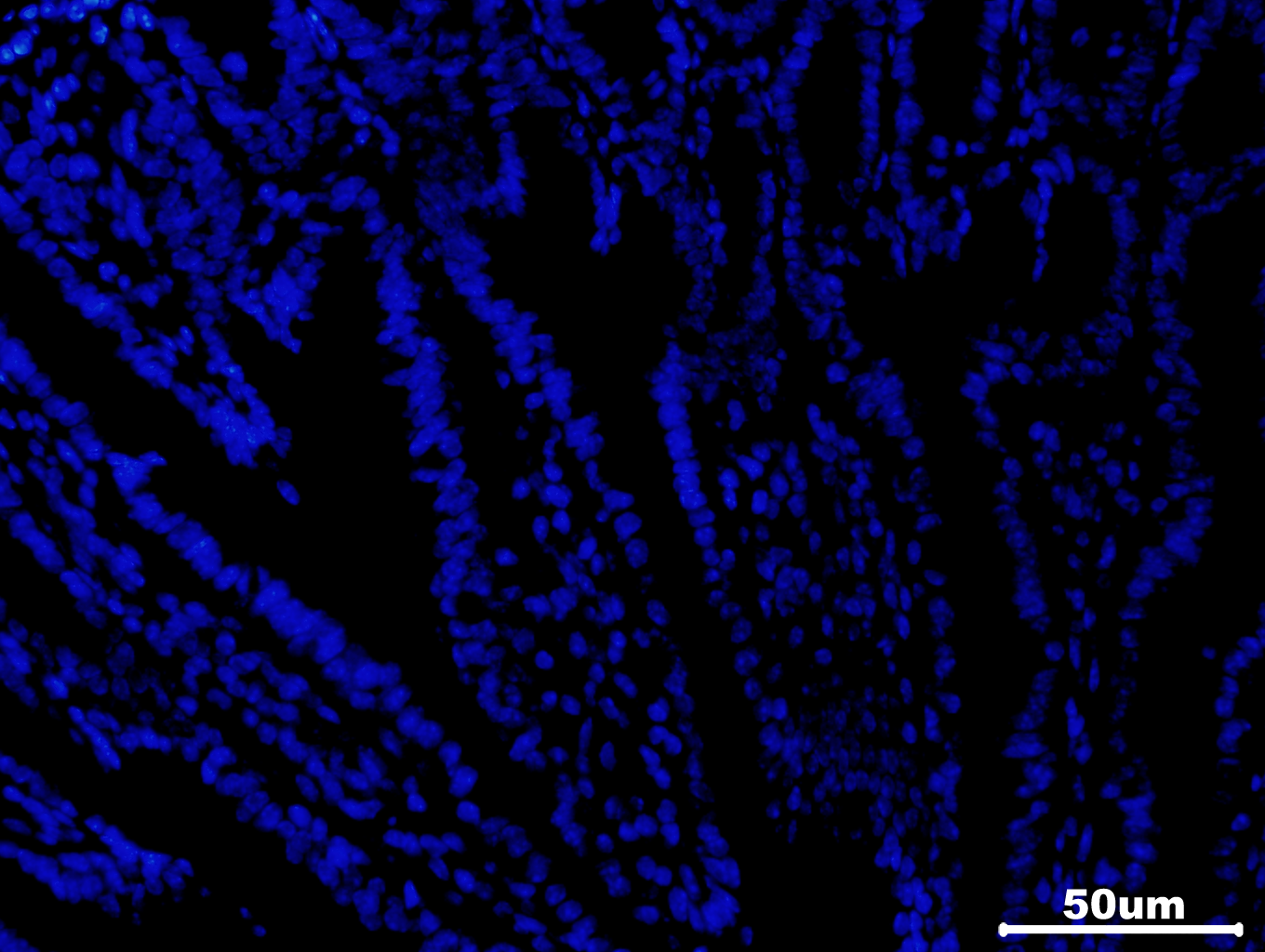

Supplement: Supplementary file 14 [file DataSheet_14.zip › E33-1-200-3-DAPI.tif]

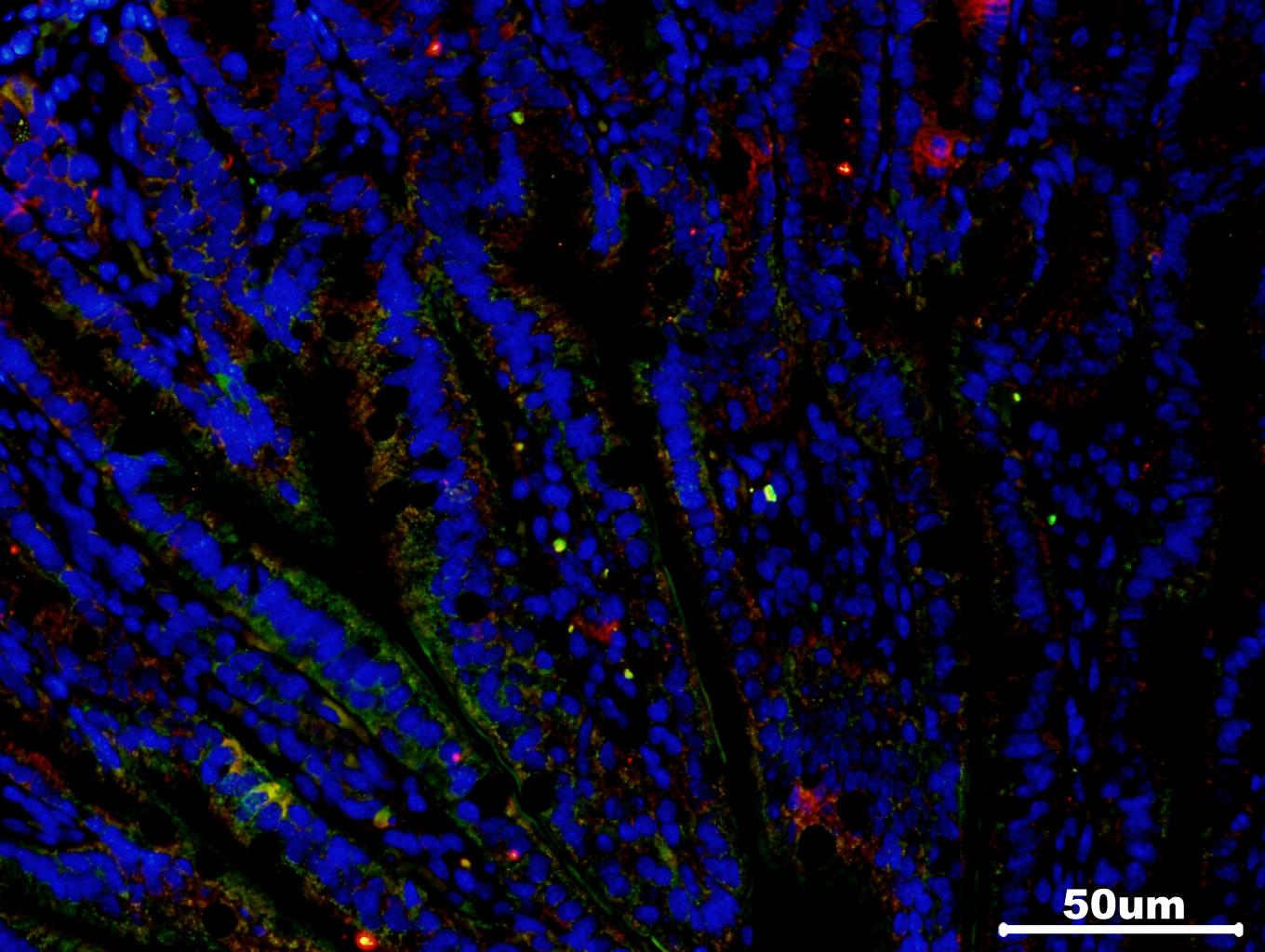

Supplement: Supplementary file 14 [file DataSheet_14.zip › E33-1-200-3-merge.tif]

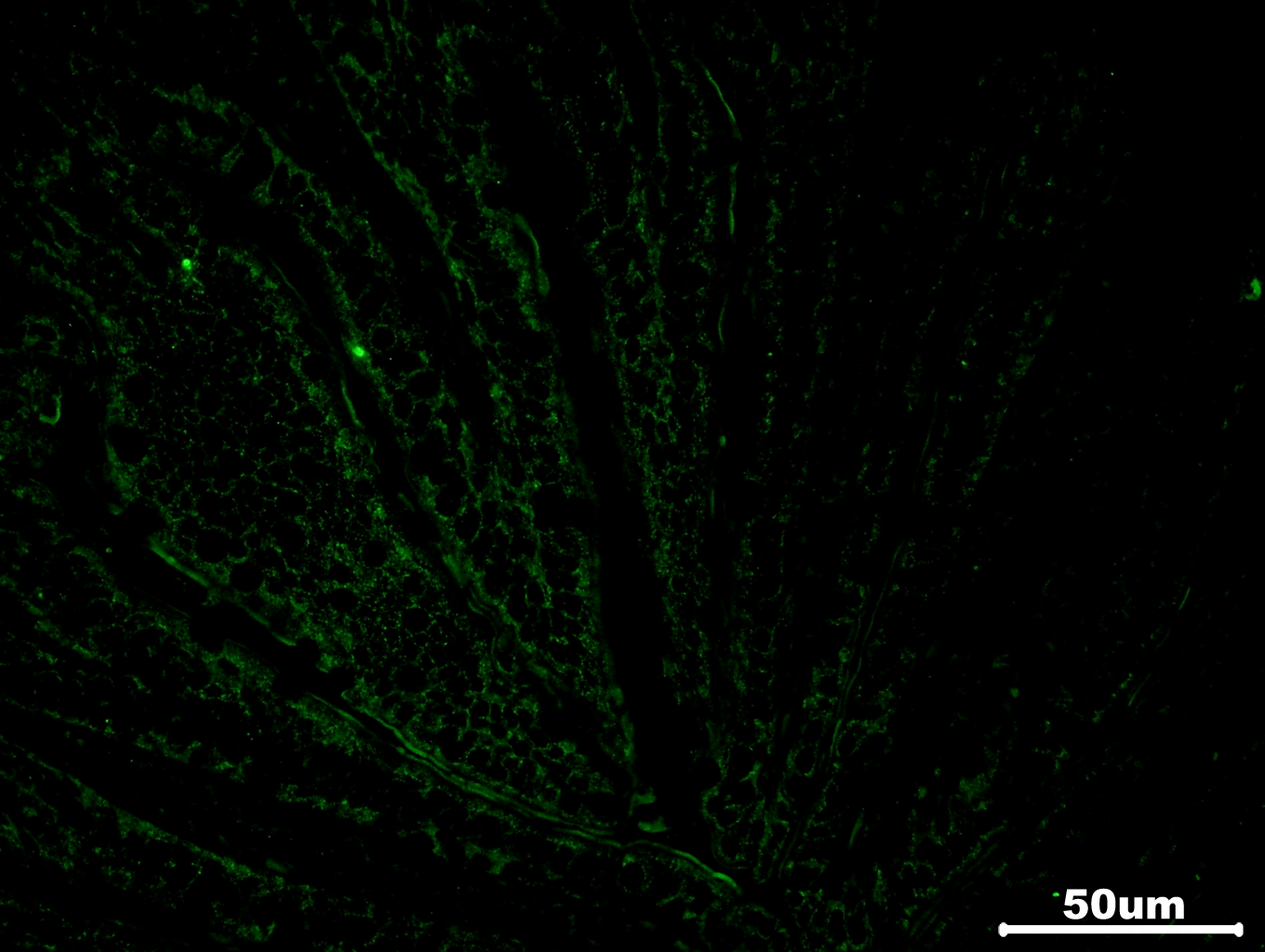

Supplement: Supplementary file 14 [file DataSheet_14.zip › E33-2-200-1-CD86.tif]

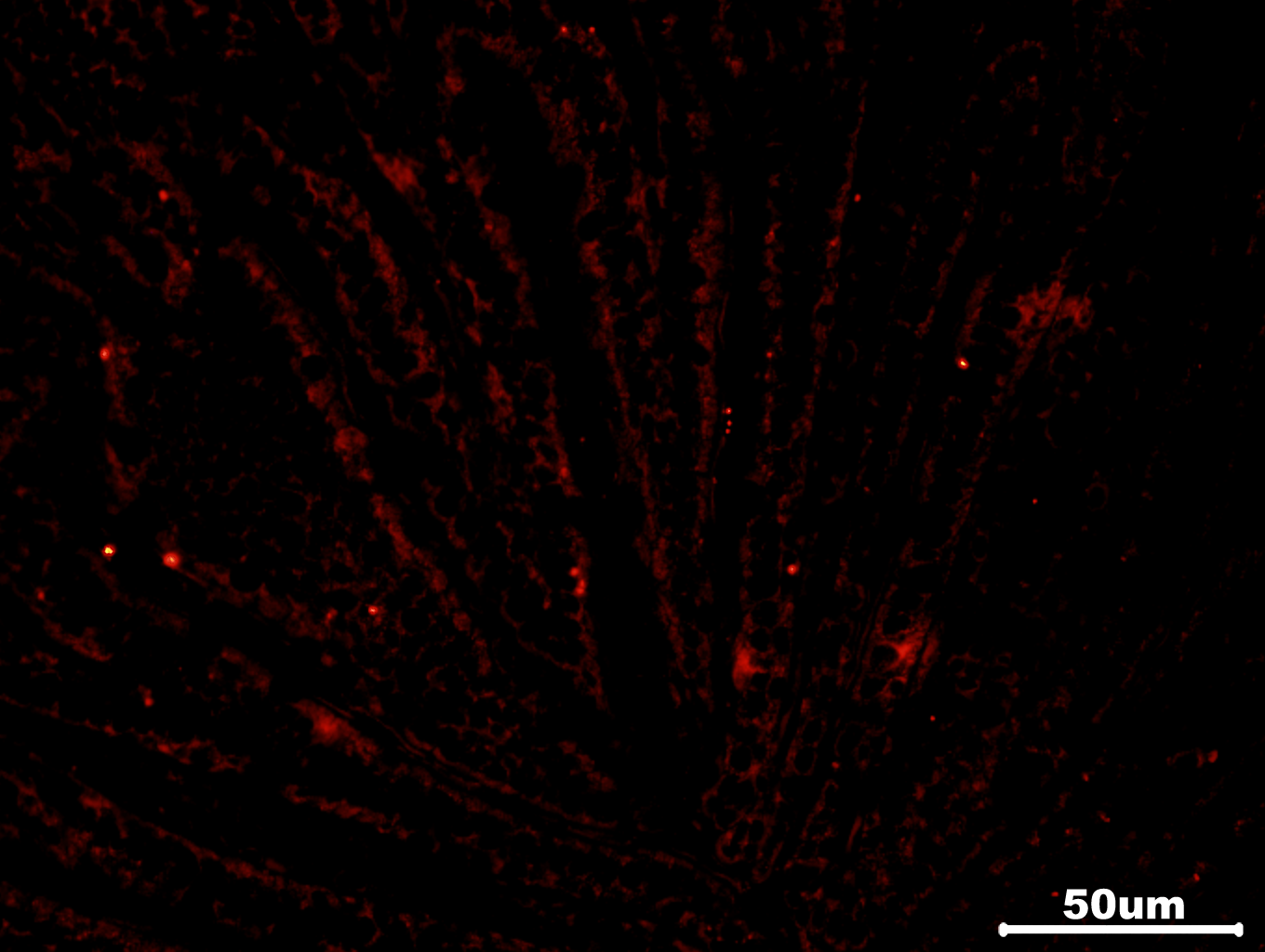

Supplement: Supplementary file 14 [file DataSheet_14.zip › E33-2-200-1-CD206.tif]

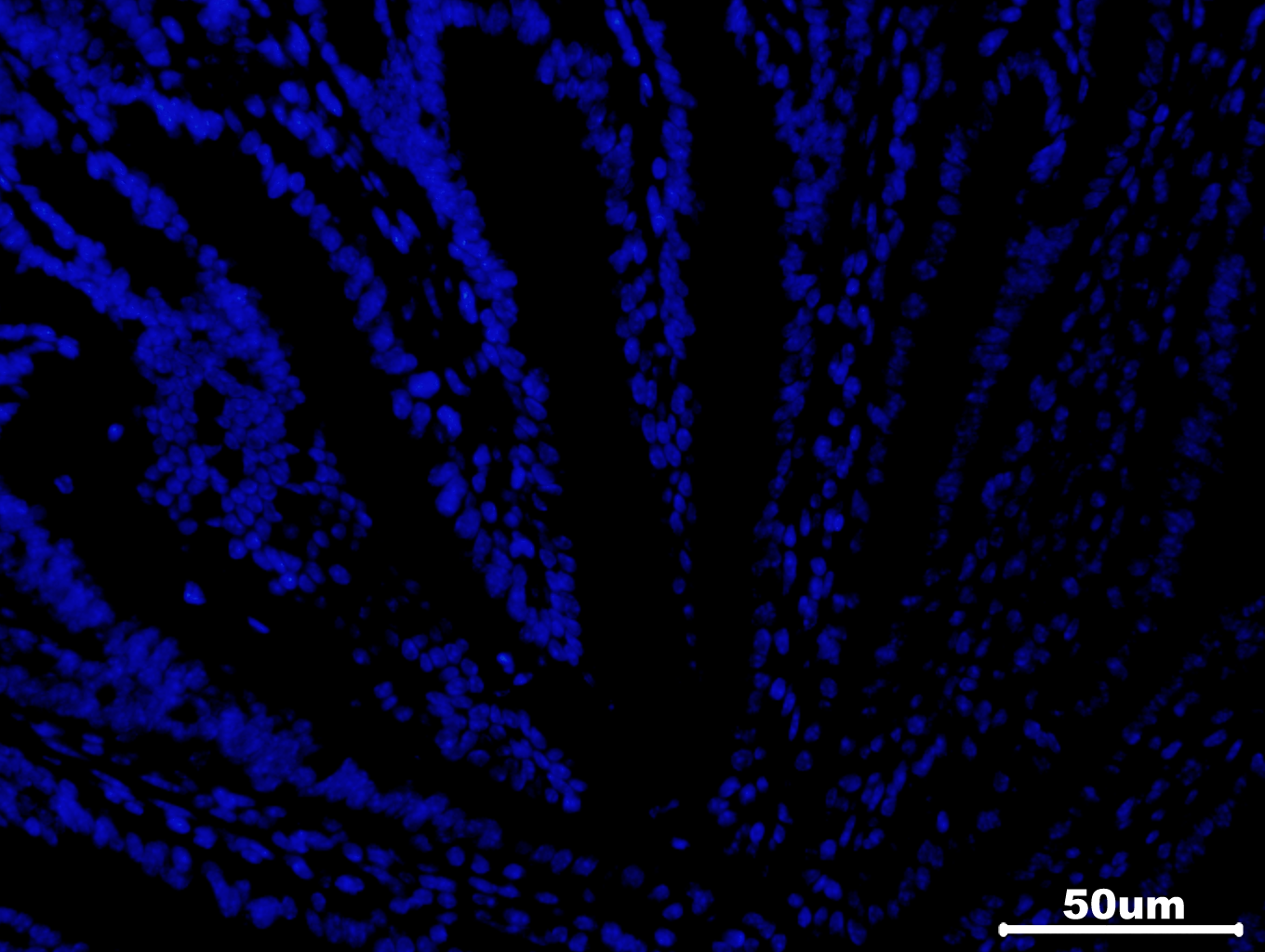

Supplement: Supplementary file 14 [file DataSheet_14.zip › E33-2-200-1-DAPI.tif]

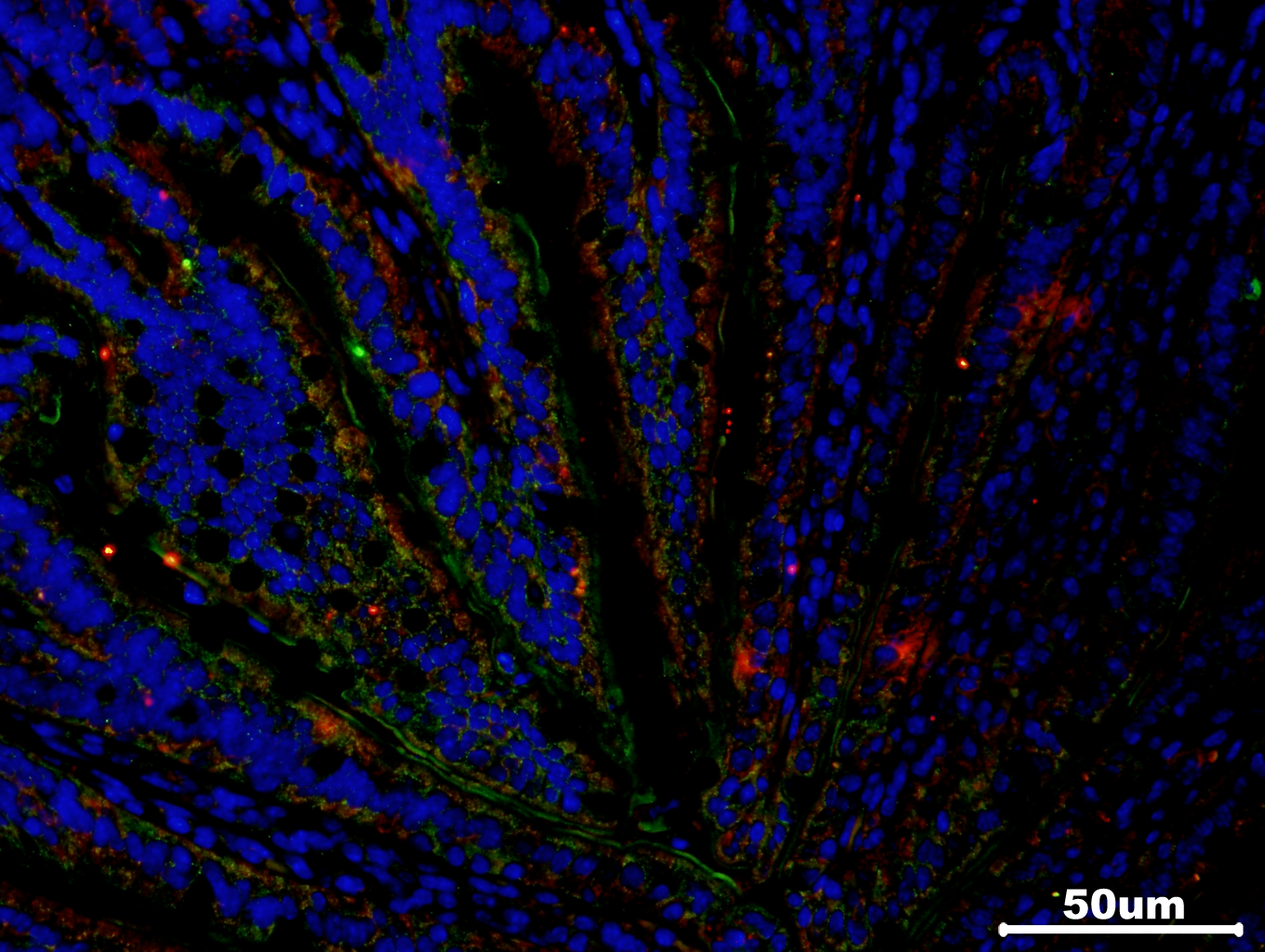

Supplement: Supplementary file 14 [file DataSheet_14.zip › E33-2-200-1-merge.tif]

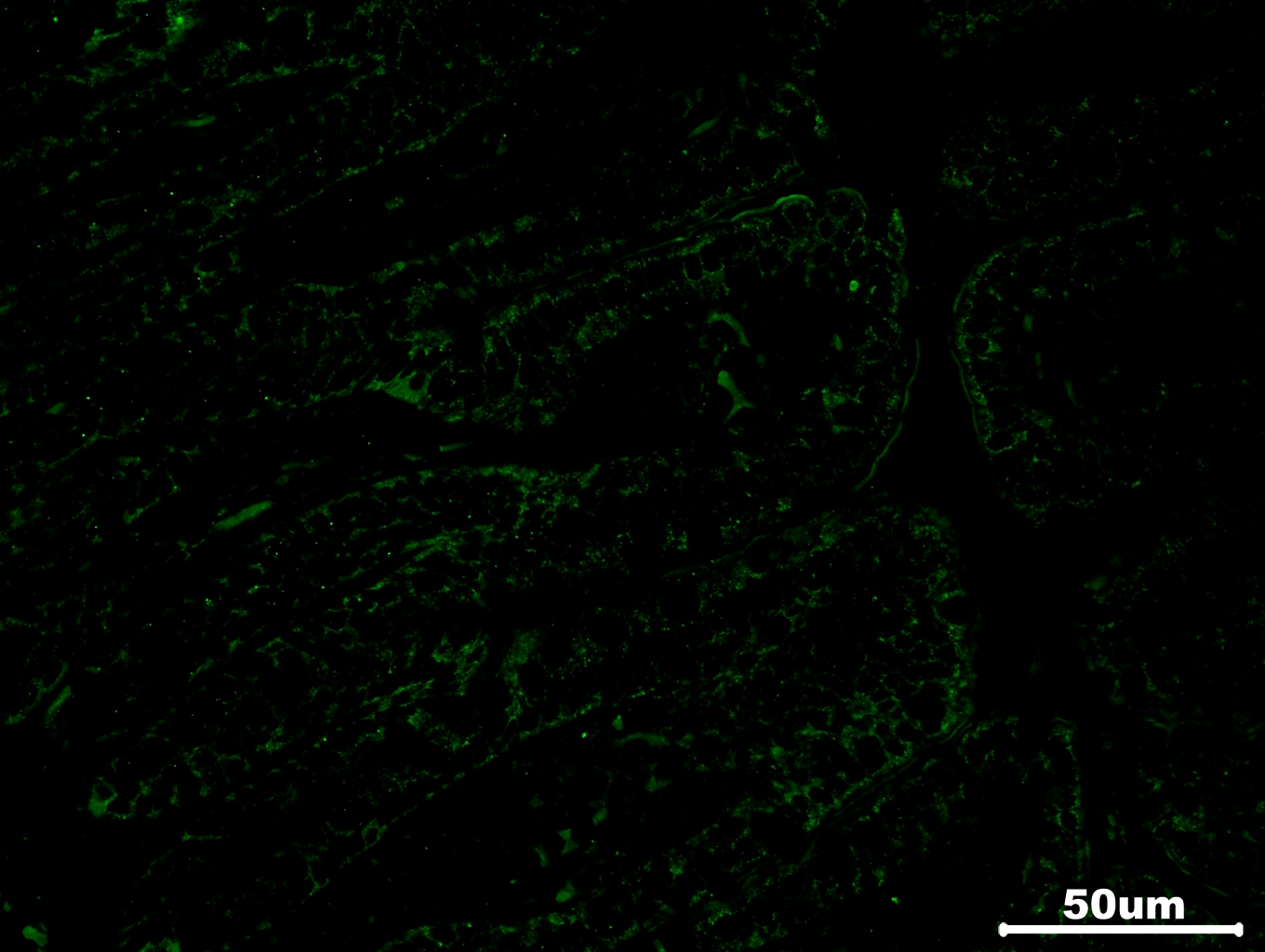

Supplement: Supplementary file 14 [file DataSheet_14.zip › E33-2-200-2-CD86.tif]

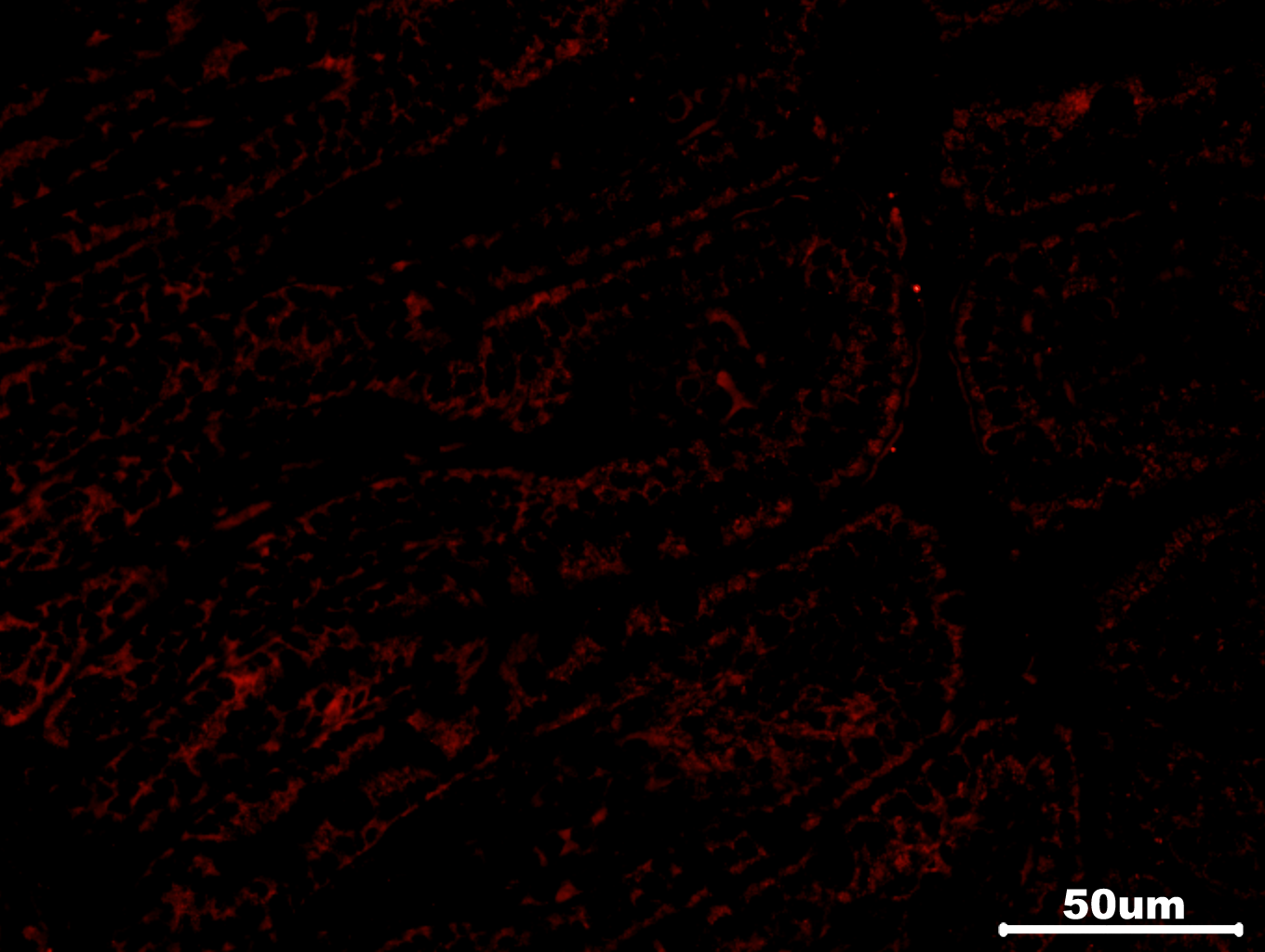

Supplement: Supplementary file 14 [file DataSheet_14.zip › E33-2-200-2-CD206.tif]

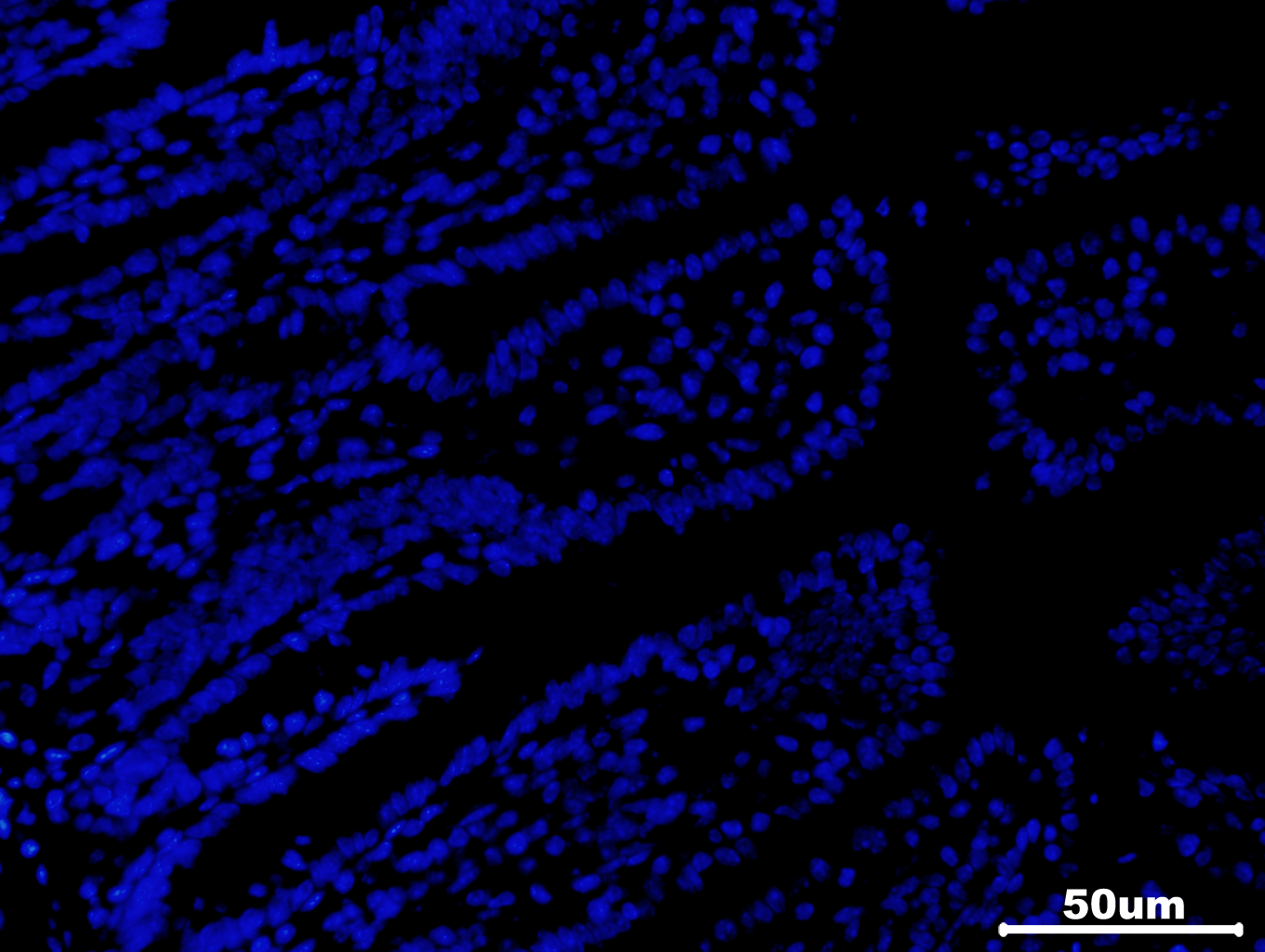

Supplement: Supplementary file 14 [file DataSheet_14.zip › E33-2-200-2-DAPI.tif]

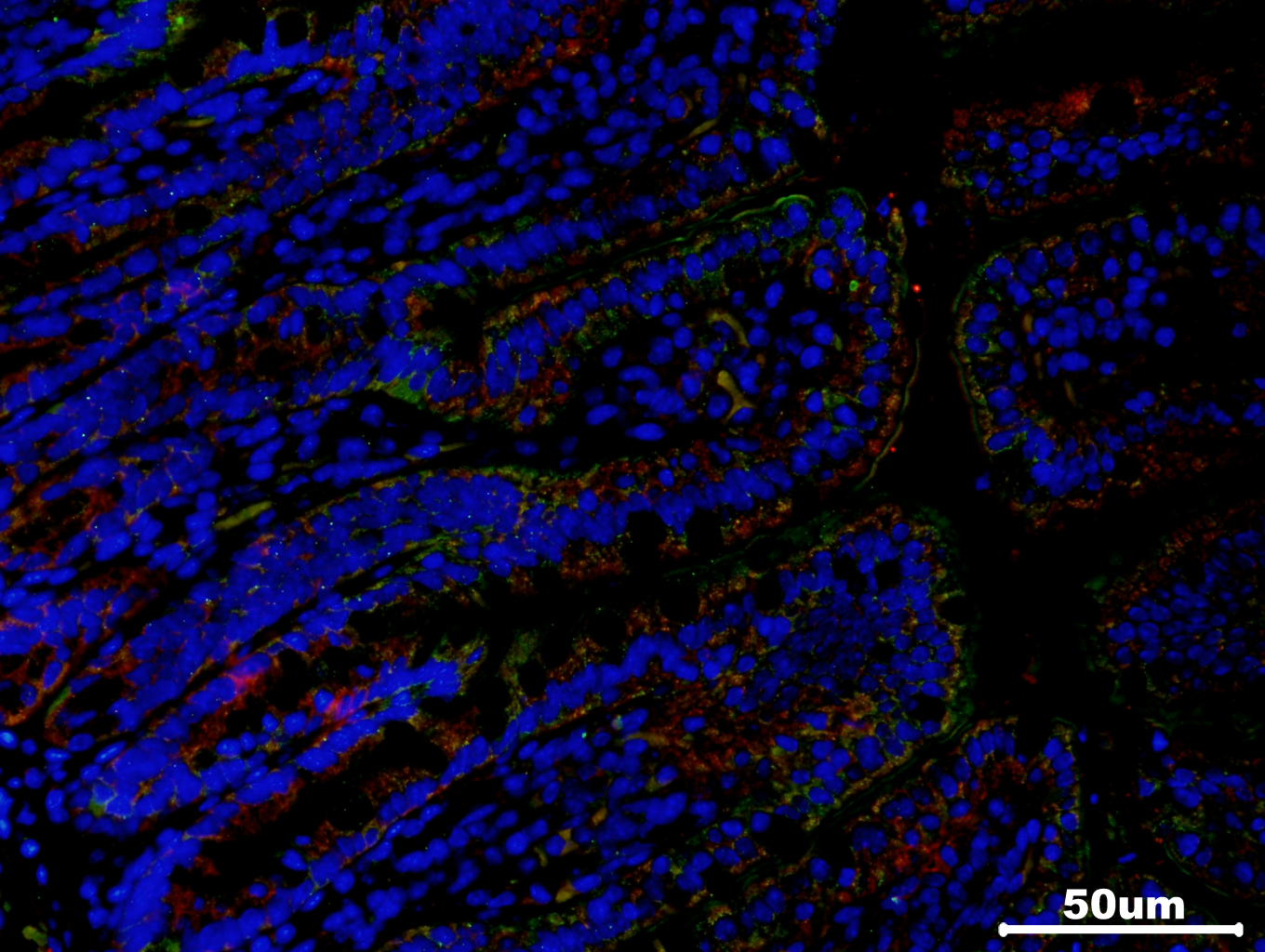

Supplement: Supplementary file 14 [file DataSheet_14.zip › E33-2-200-2-merge.tif]

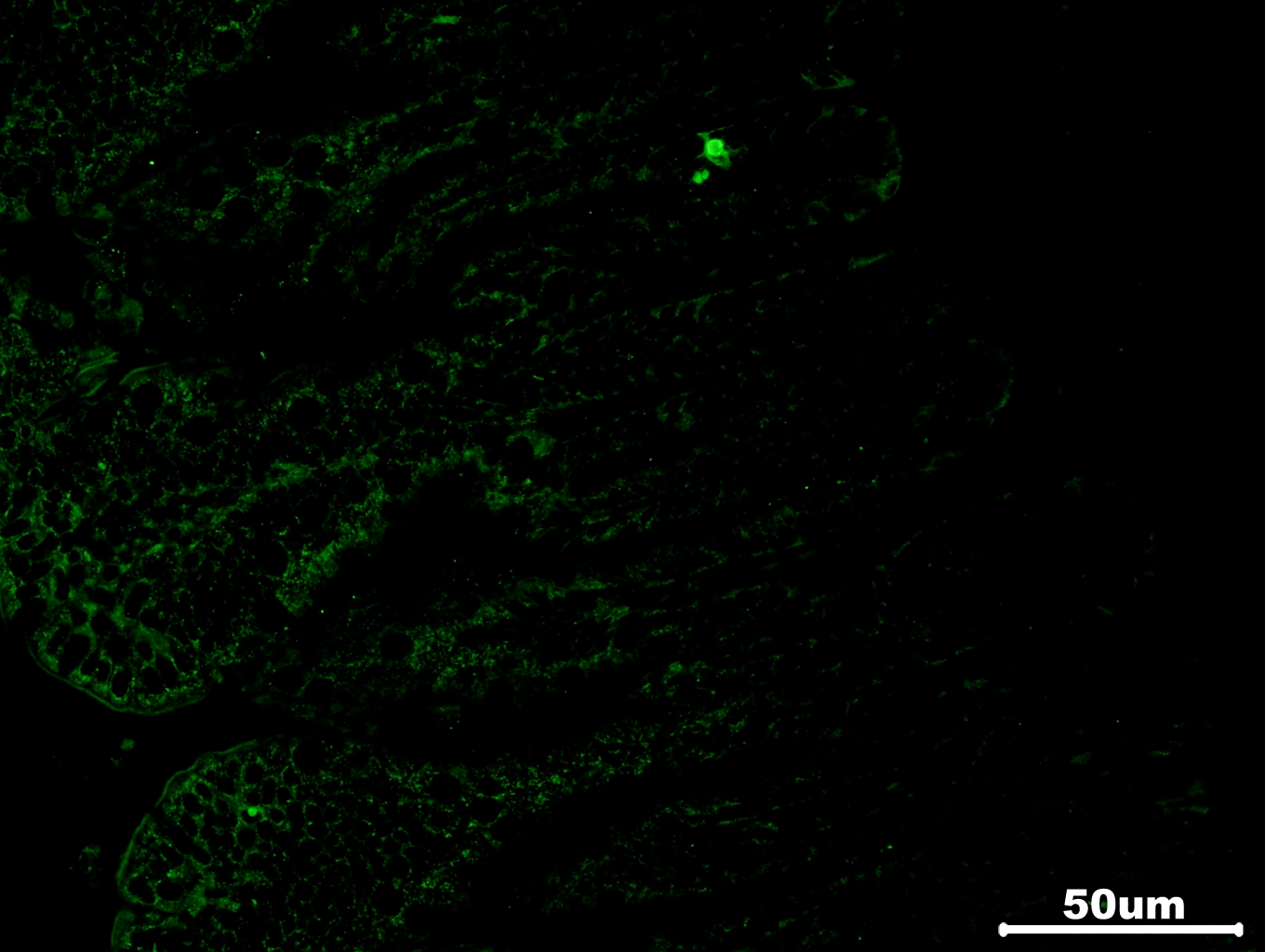

Supplement: Supplementary file 14 [file DataSheet_14.zip › E33-2-200-3-CD86.tif]

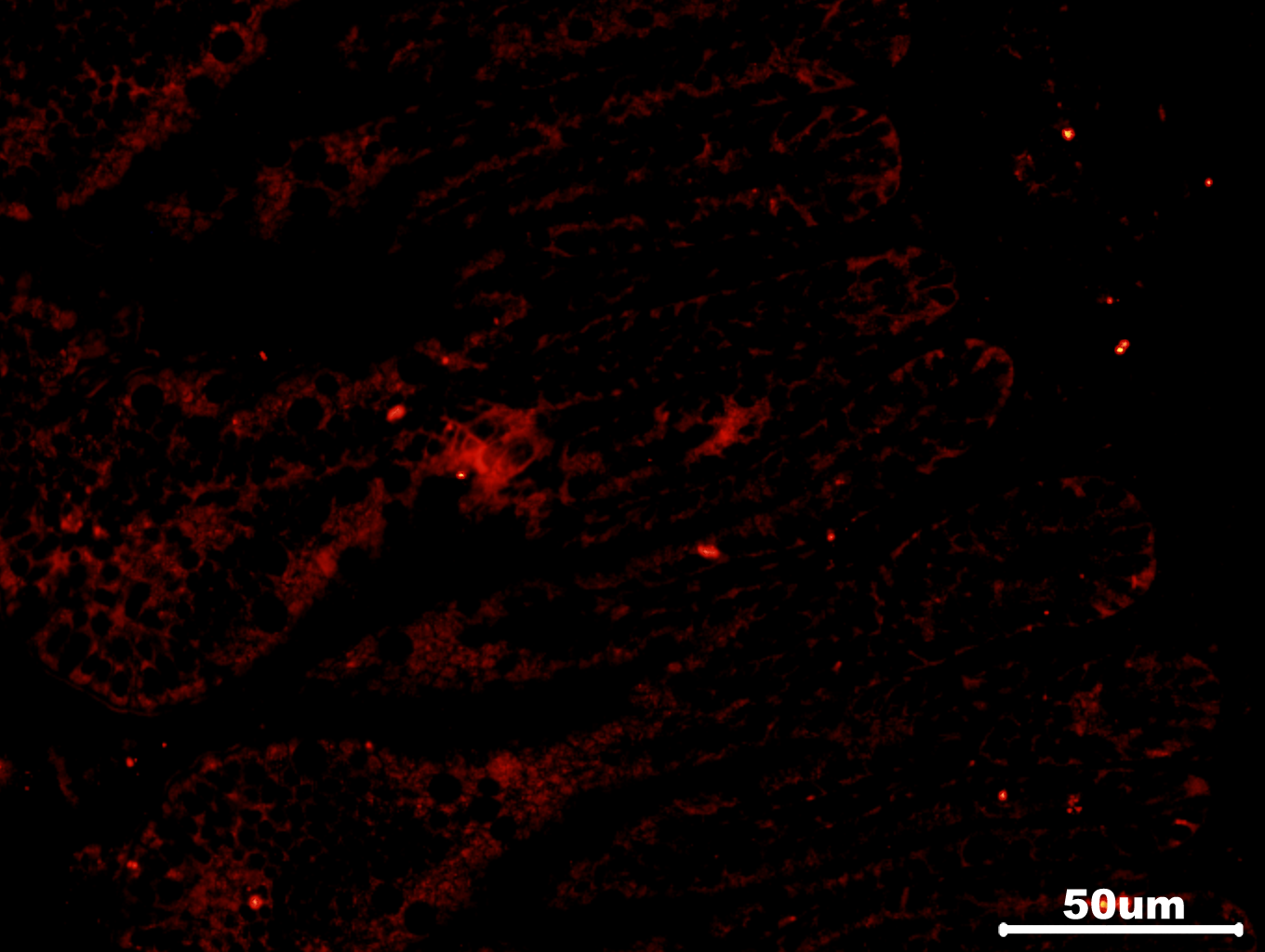

Supplement: Supplementary file 14 [file DataSheet_14.zip › E33-2-200-3-CD206.tif]

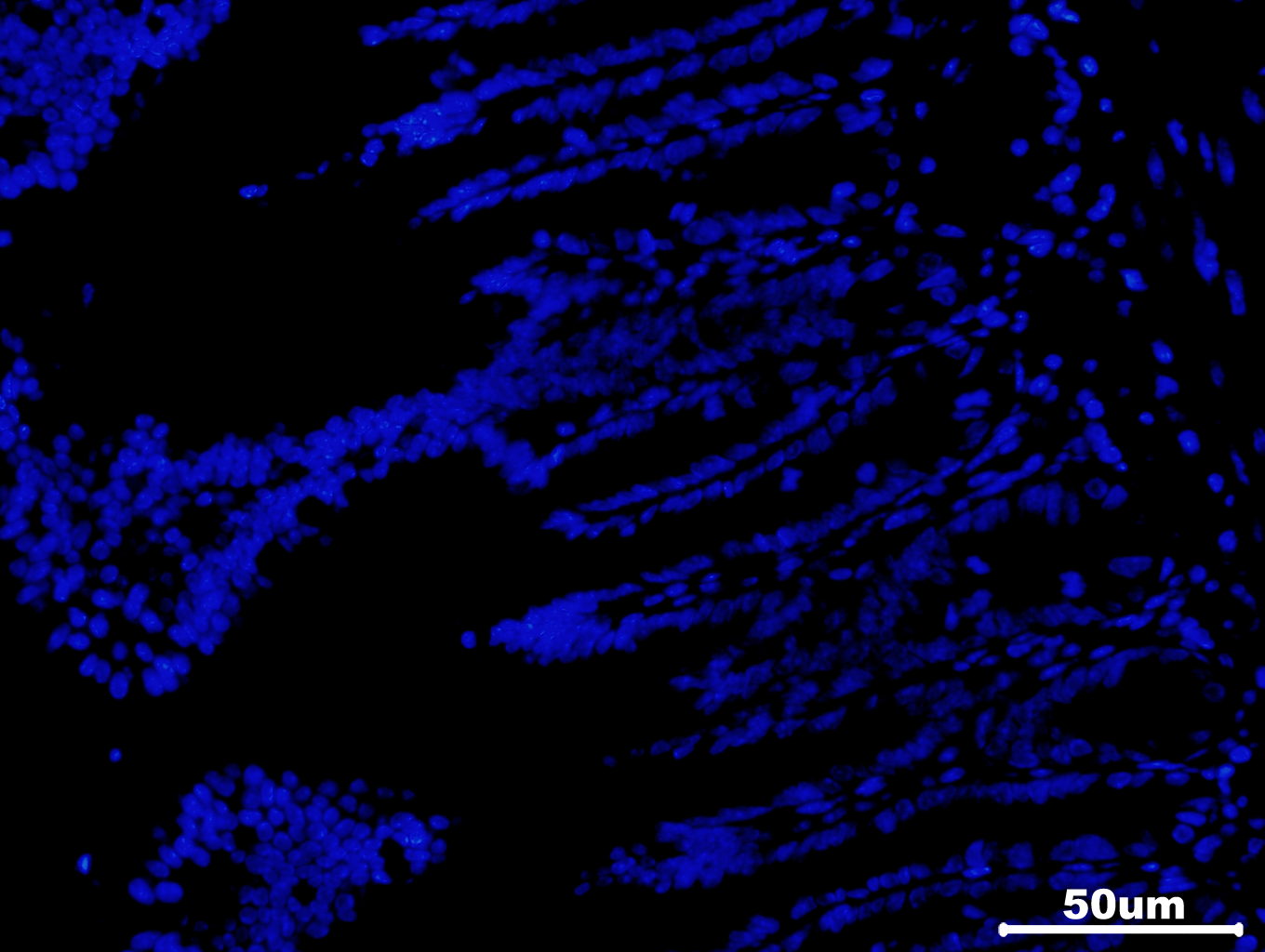

Supplement: Supplementary file 14 [file DataSheet_14.zip › E33-2-200-3-DAPI.tif]

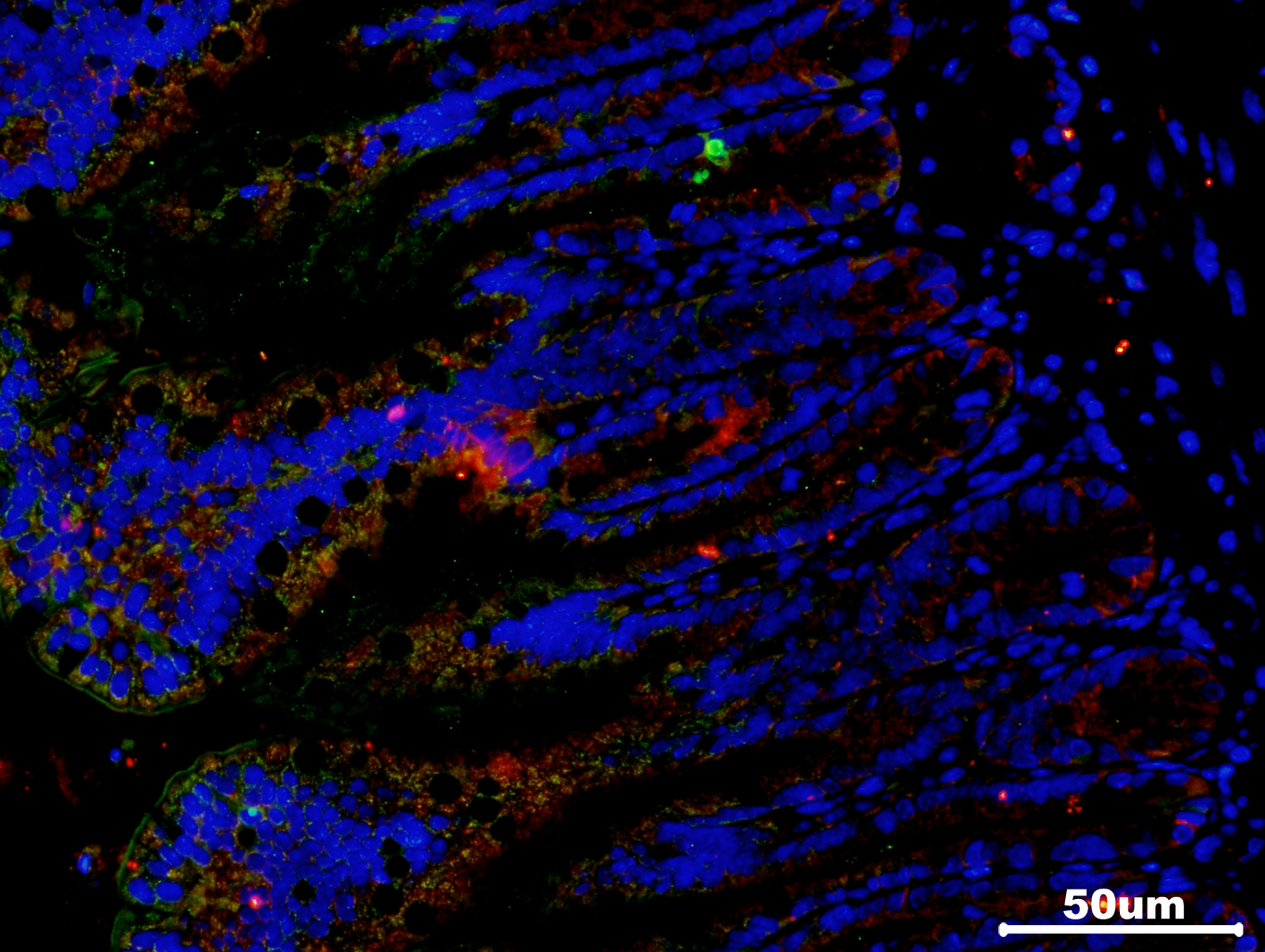

Supplement: Supplementary file 14 [file DataSheet_14.zip › E33-2-200-3-merge.tif]

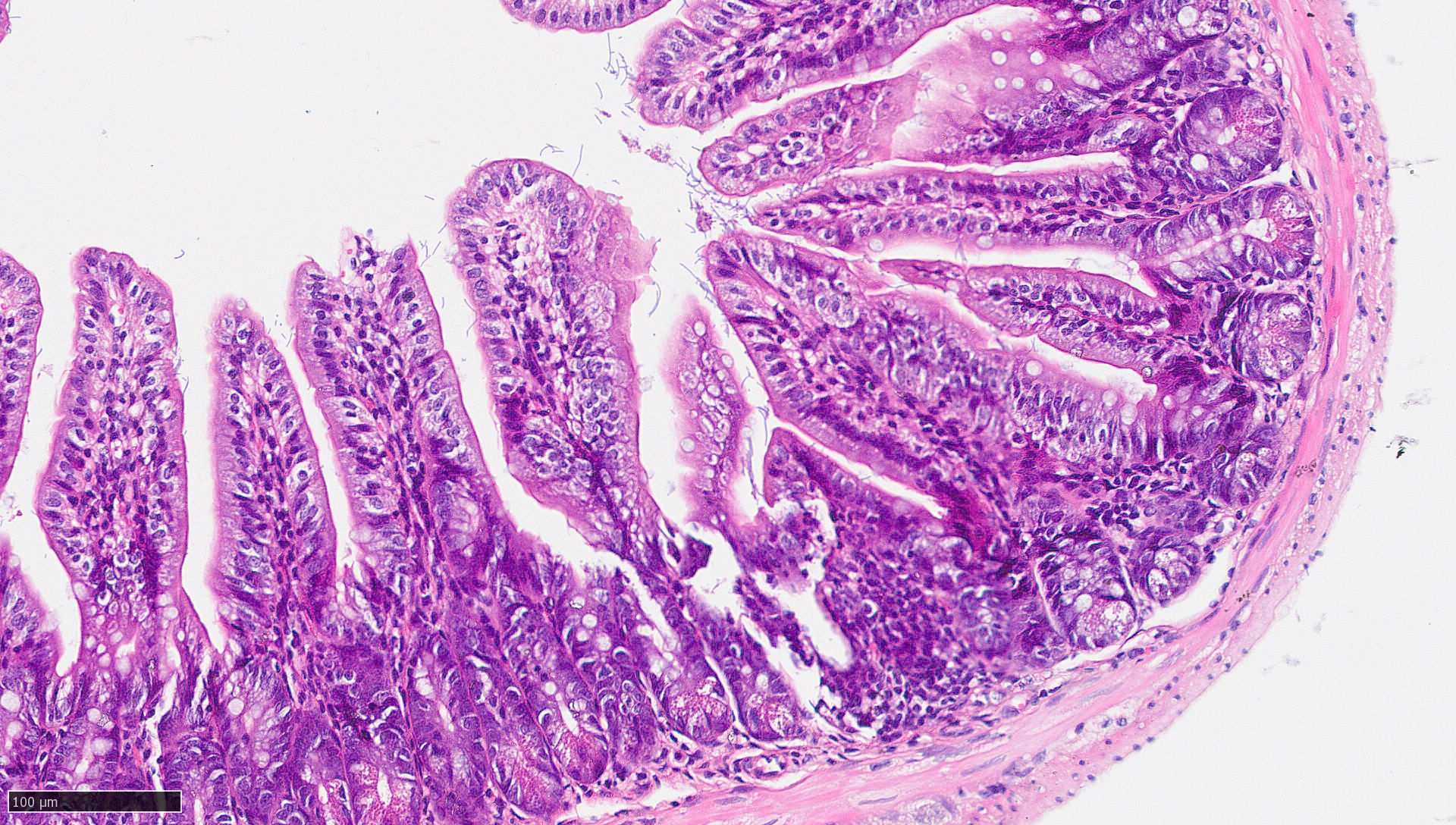

Supplement: Supplementary file 15 [file DataSheet_15.zip › μ£¬σæ╜σÉìμûçΣ╗╢σñ╣/Figure 1/Fig.1 Other files/full scans of Immunohistochemical staining of intestine/CON (2).jpg]

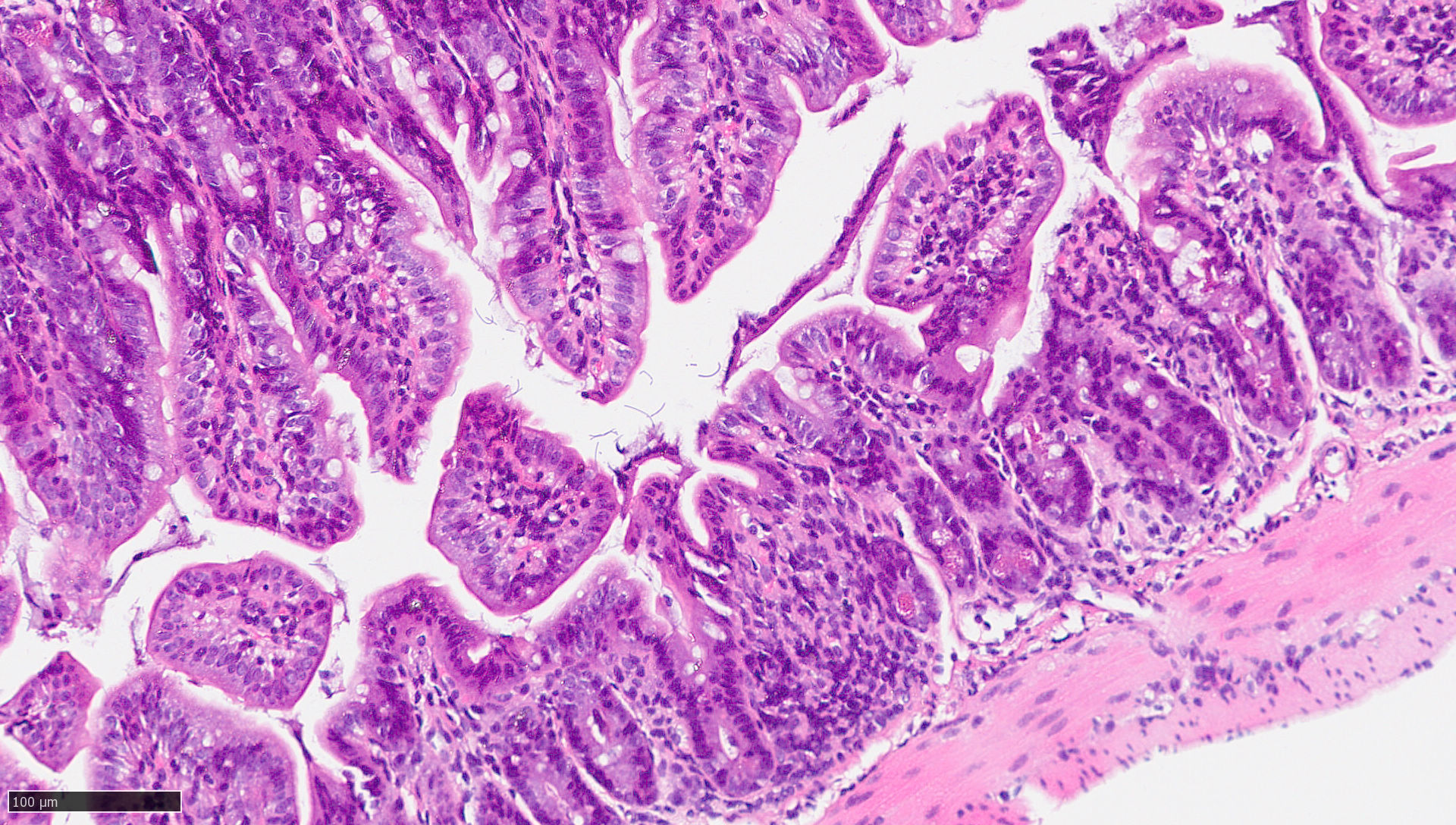

Supplement: Supplementary file 15 [file DataSheet_15.zip › μ£¬σæ╜σÉìμûçΣ╗╢σñ╣/Figure 1/Fig.1 Other files/full scans of Immunohistochemical staining of intestine/EA (1).jpg]

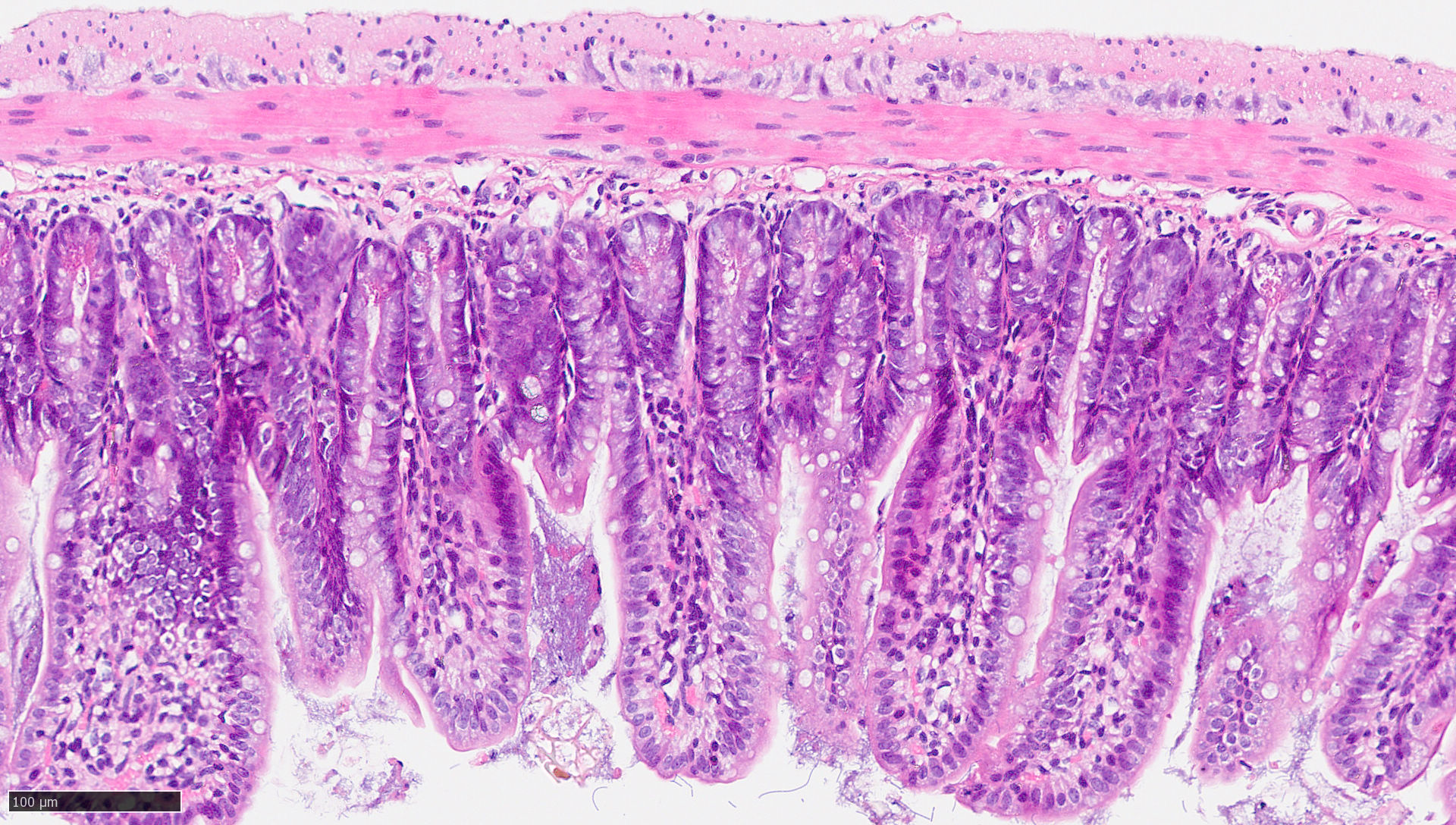

Supplement: Supplementary file 15 [file DataSheet_15.zip › μ£¬σæ╜σÉìμûçΣ╗╢σñ╣/Figure 1/Fig.1 Other files/full scans of Immunohistochemical staining of intestine/NEA (6).jpg]

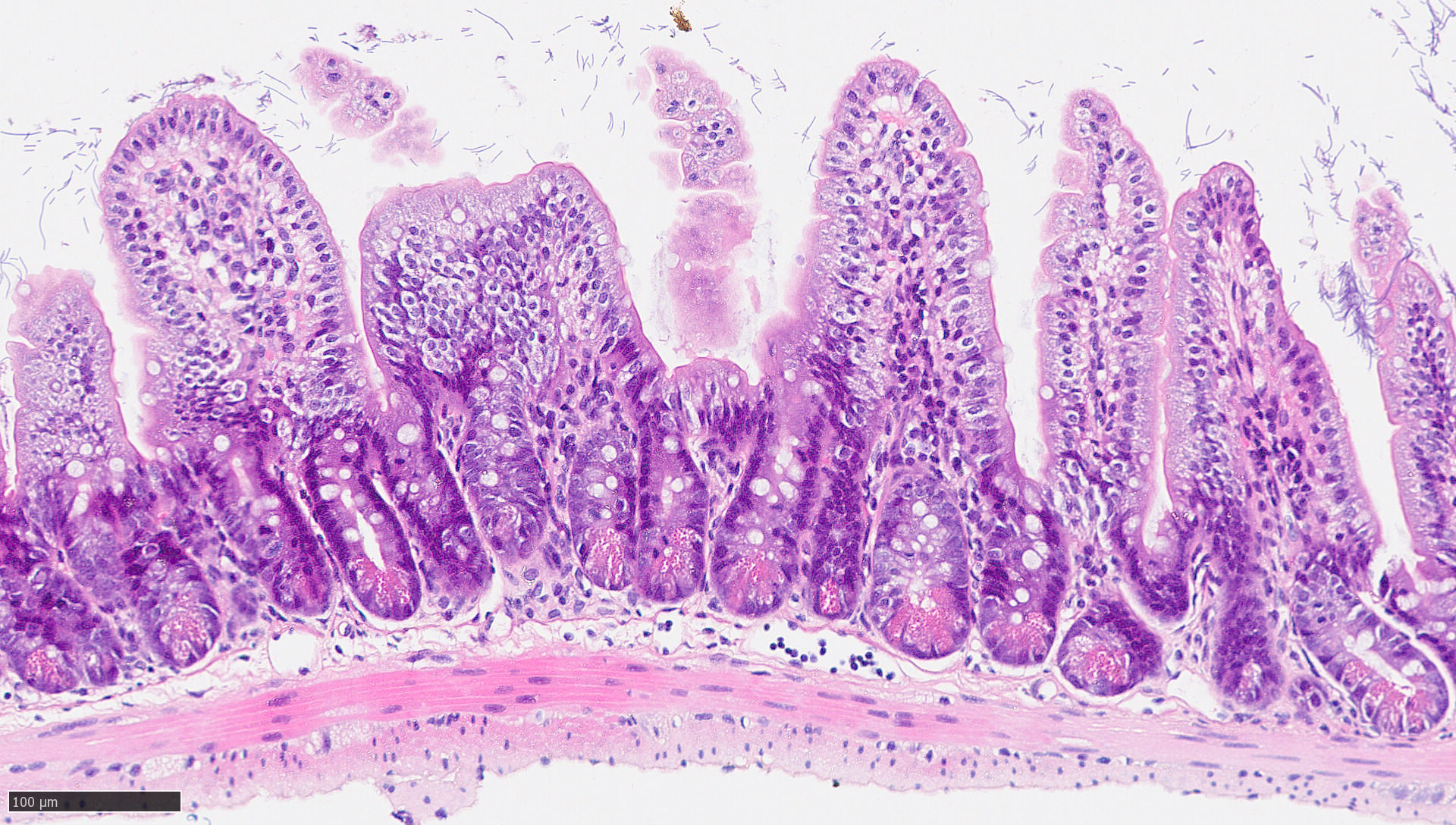

Supplement: Supplementary file 15 [file DataSheet_15.zip › μ£¬σæ╜σÉìμûçΣ╗╢σñ╣/Figure 1/Fig.1 Other files/full scans of Immunohistochemical staining of intestine/CON (3).jpg]

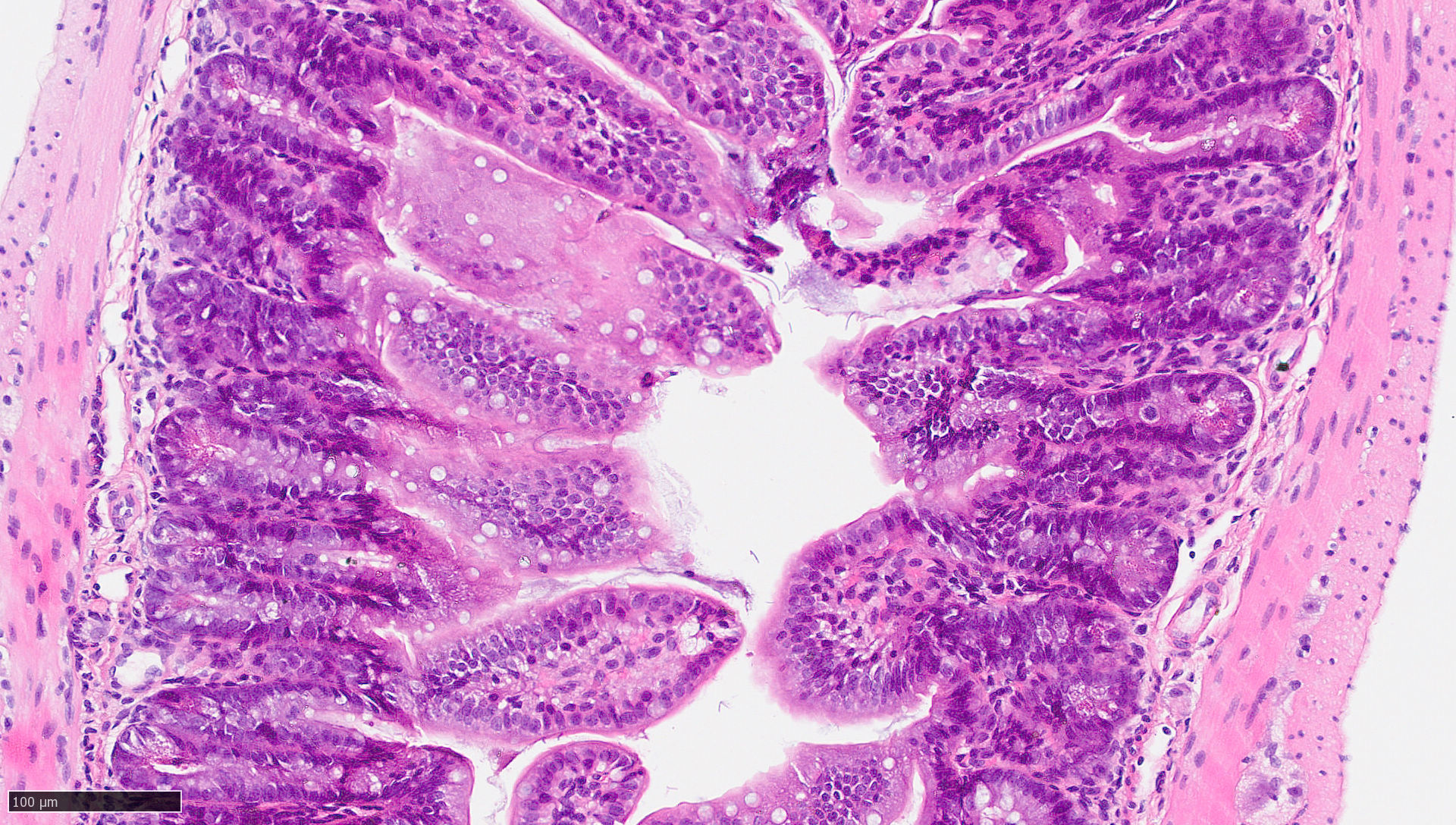

Supplement: Supplementary file 15 [file DataSheet_15.zip › μ£¬σæ╜σÉìμûçΣ╗╢σñ╣/Figure 1/Fig.1 Other files/full scans of Immunohistochemical staining of intestine/NEA (1).jpg]

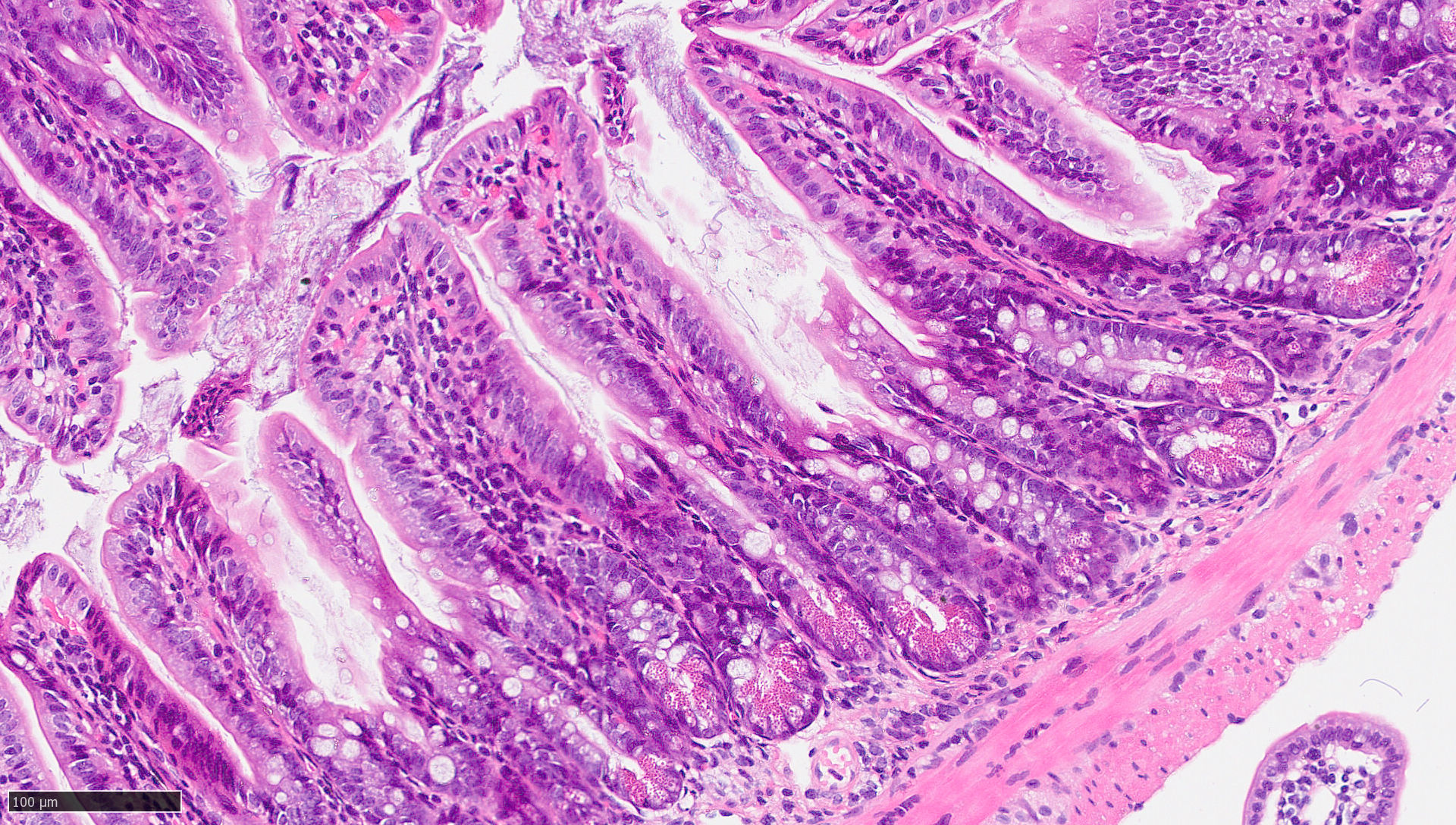

Supplement: Supplementary file 15 [file DataSheet_15.zip › μ£¬σæ╜σÉìμûçΣ╗╢σñ╣/Figure 1/Fig.1 Other files/full scans of Immunohistochemical staining of intestine/EA (6).jpg]

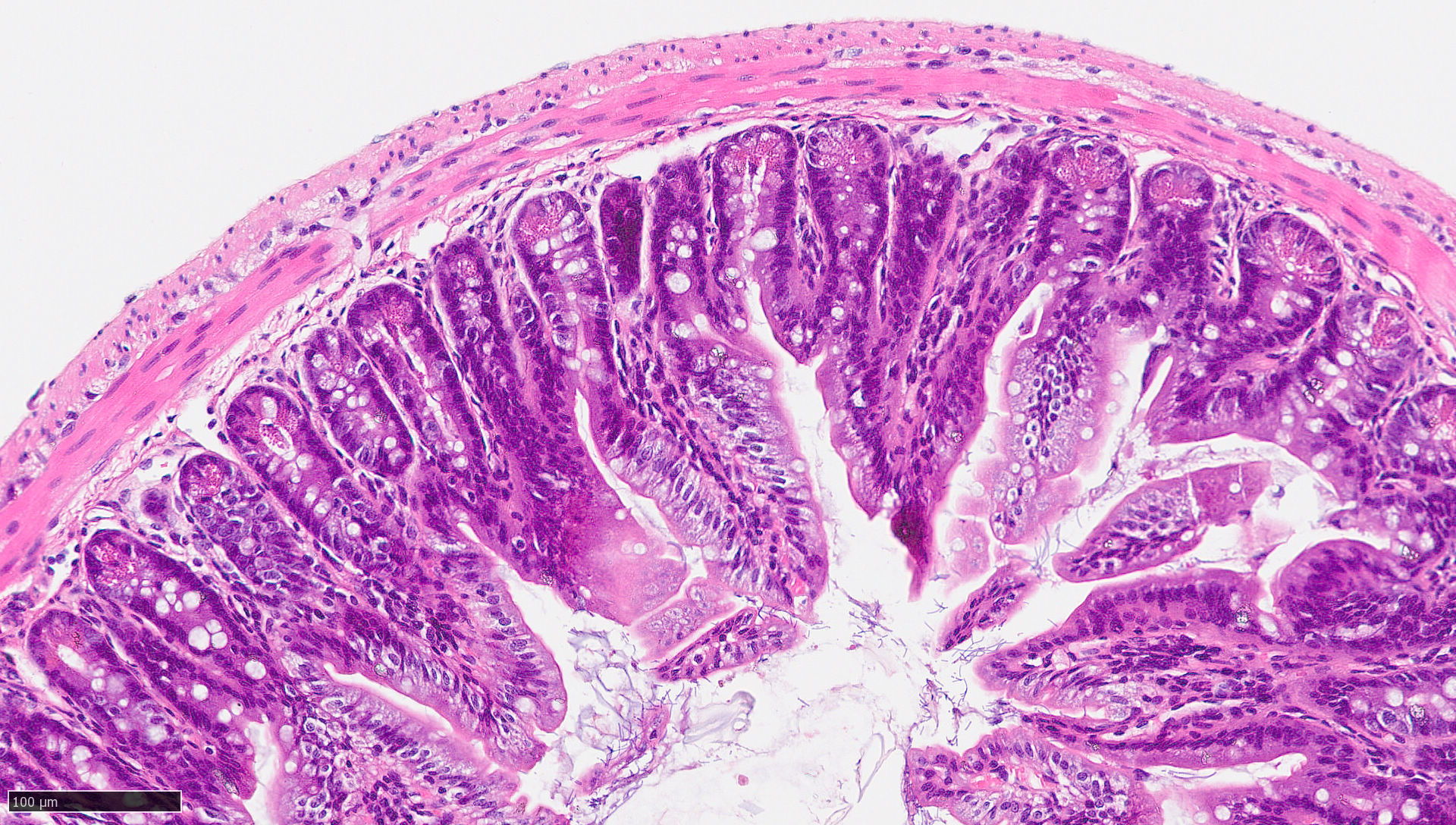

Supplement: Supplementary file 15 [file DataSheet_15.zip › μ£¬σæ╜σÉìμûçΣ╗╢σñ╣/Figure 1/Fig.1 Other files/full scans of Immunohistochemical staining of intestine/CON (4).jpg]

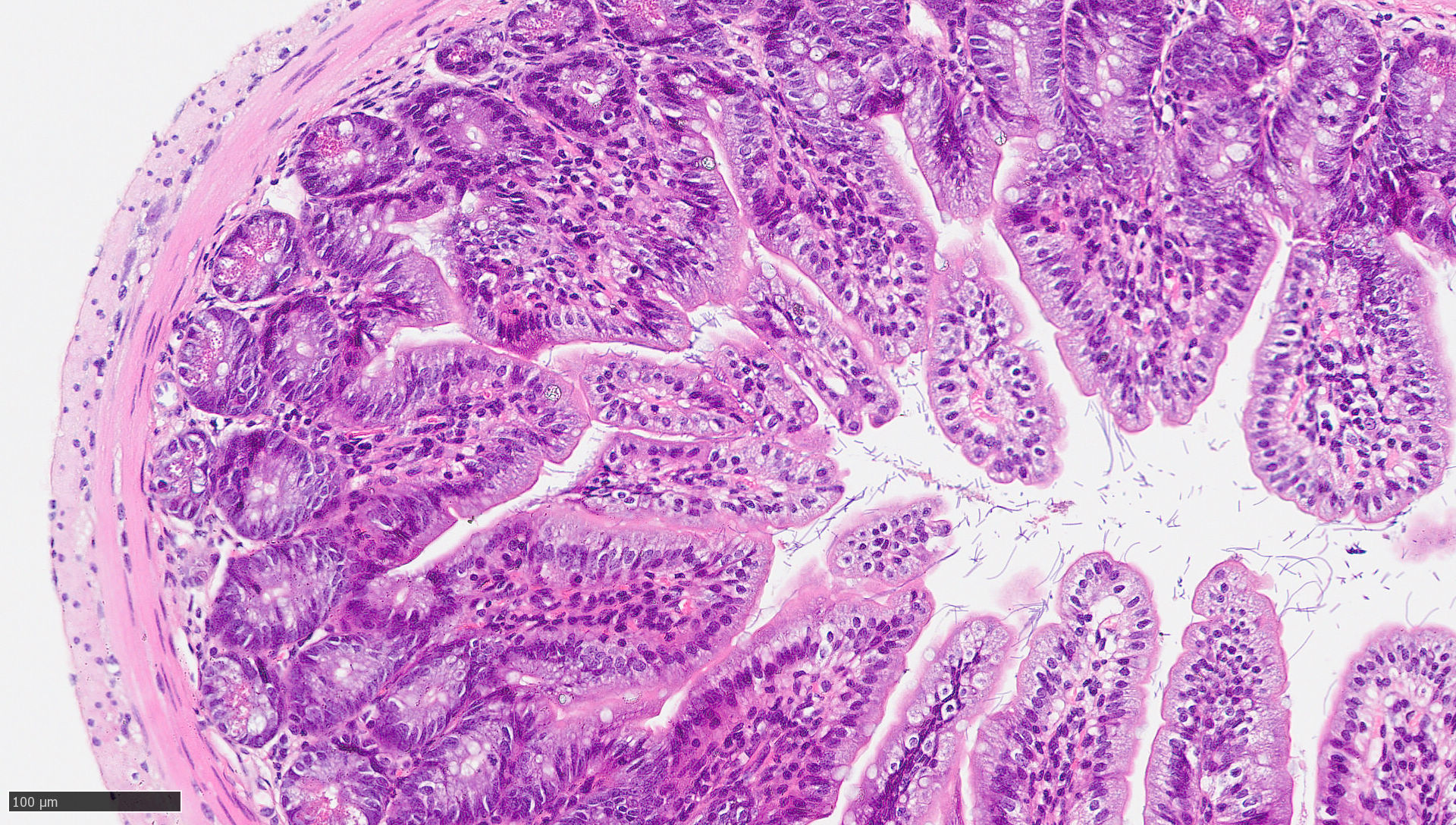

Supplement: Supplementary file 15 [file DataSheet_15.zip › μ£¬σæ╜σÉìμûçΣ╗╢σñ╣/Figure 1/Fig.1 Other files/full scans of Immunohistochemical staining of intestine/CON (5).jpg]

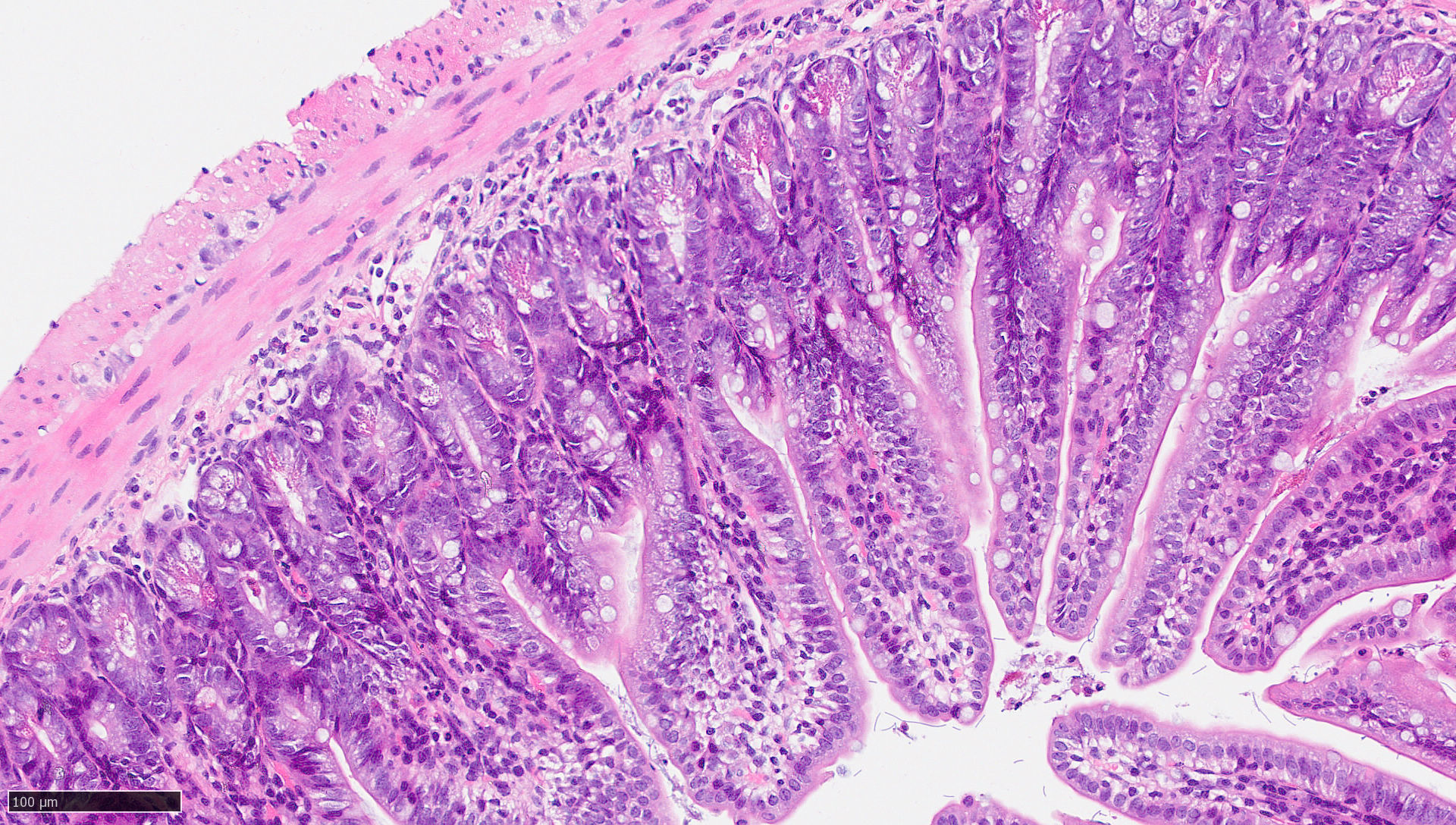

Supplement: Supplementary file 15 [file DataSheet_15.zip › μ£¬σæ╜σÉìμûçΣ╗╢σñ╣/Figure 1/Fig.1 Other files/full scans of Immunohistochemical staining of intestine/EA (4).jpg]

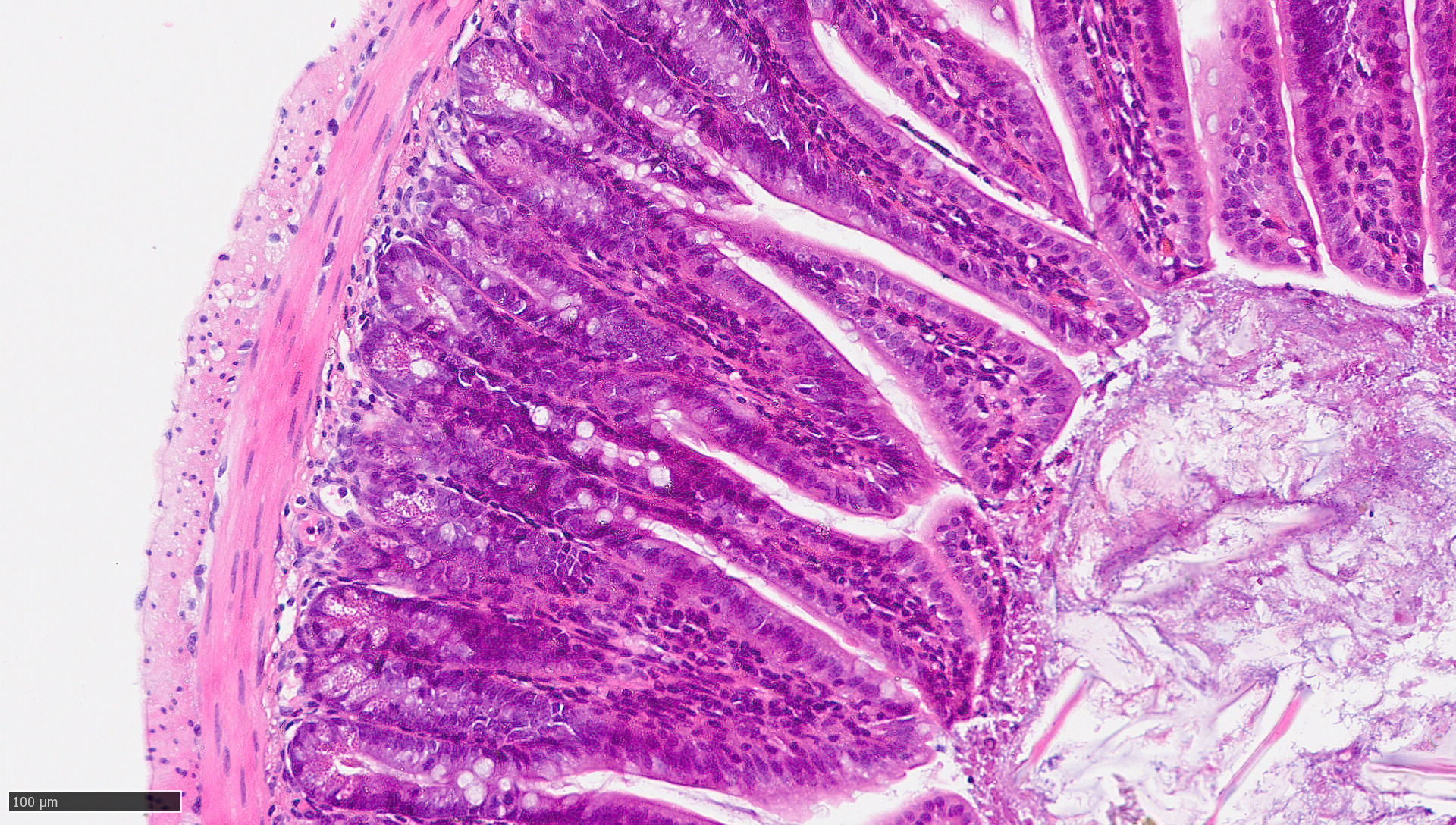

Supplement: Supplementary file 15 [file DataSheet_15.zip › μ£¬σæ╜σÉìμûçΣ╗╢σñ╣/Figure 1/Fig.1 Other files/full scans of Immunohistochemical staining of intestine/NEA (3).jpg]

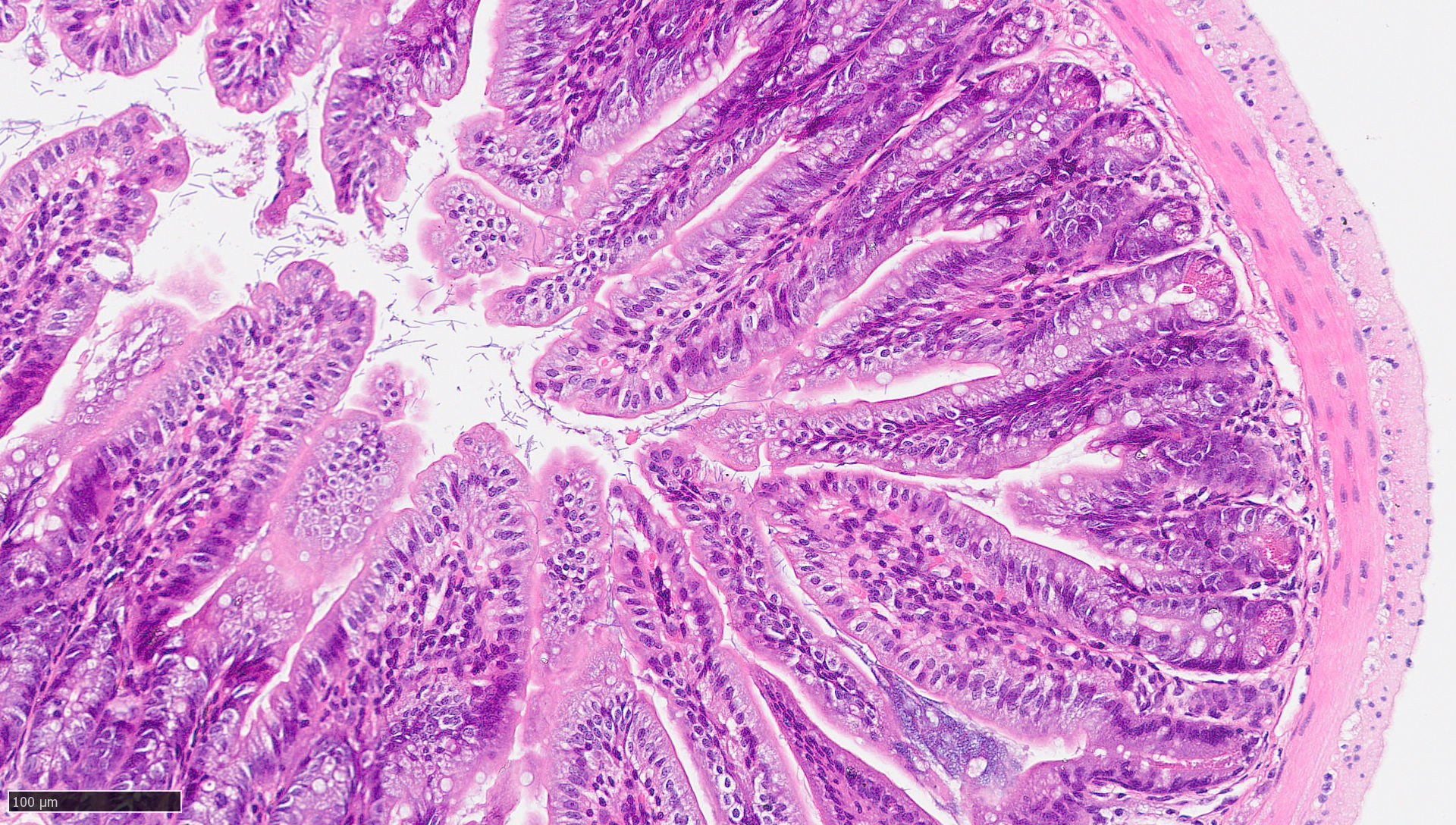

Supplement: Supplementary file 15 [file DataSheet_15.zip › μ£¬σæ╜σÉìμûçΣ╗╢σñ╣/Figure 1/Fig.1 Other files/full scans of Immunohistochemical staining of intestine/CON (6).jpg]

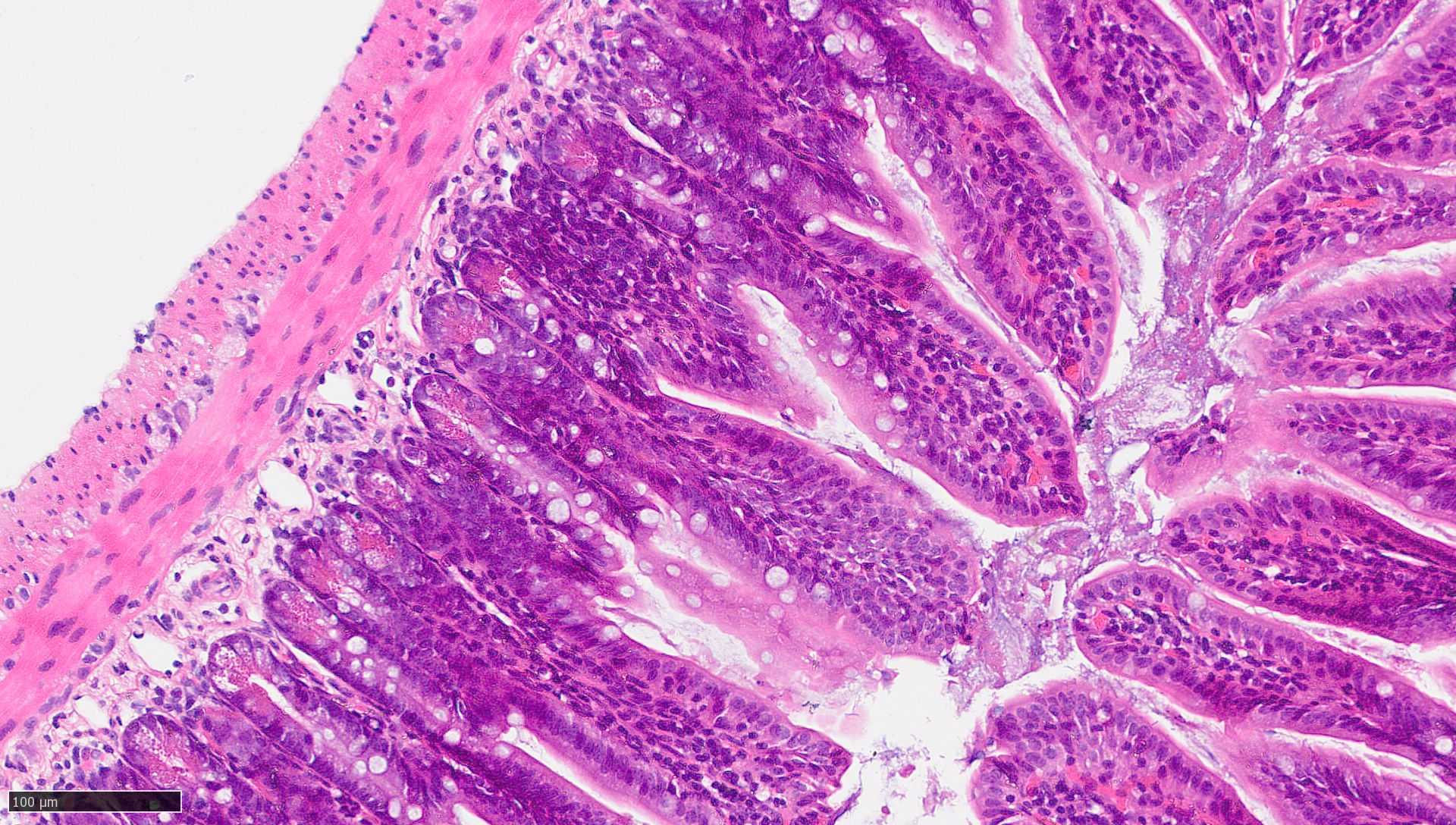

Supplement: Supplementary file 15 [file DataSheet_15.zip › μ£¬σæ╜σÉìμûçΣ╗╢σñ╣/Figure 1/Fig.1 Other files/full scans of Immunohistochemical staining of intestine/NEA (2).jpg]

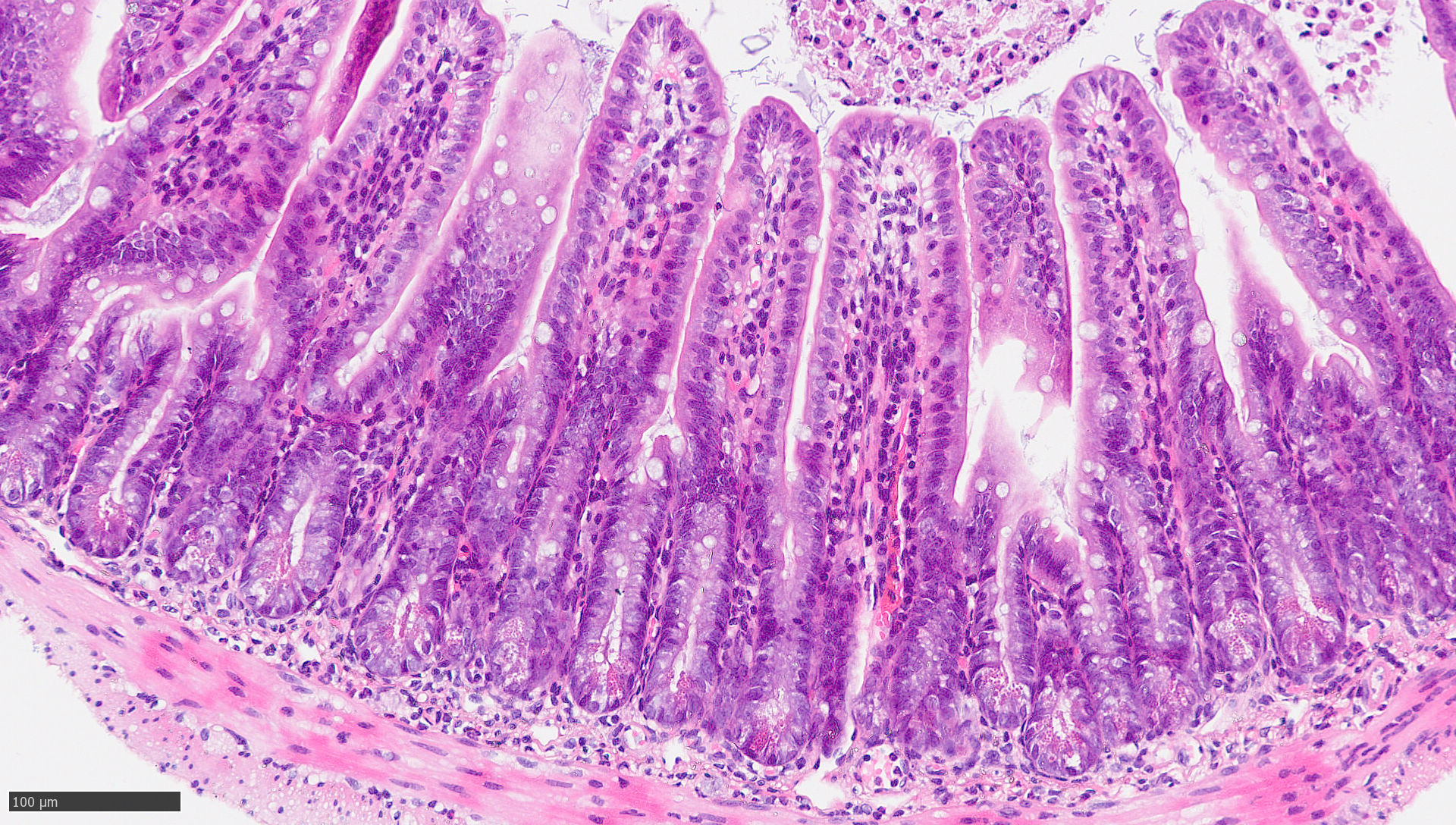

Supplement: Supplementary file 15 [file DataSheet_15.zip › μ£¬σæ╜σÉìμûçΣ╗╢σñ╣/Figure 1/Fig.1 Other files/full scans of Immunohistochemical staining of intestine/EA (5).jpg]

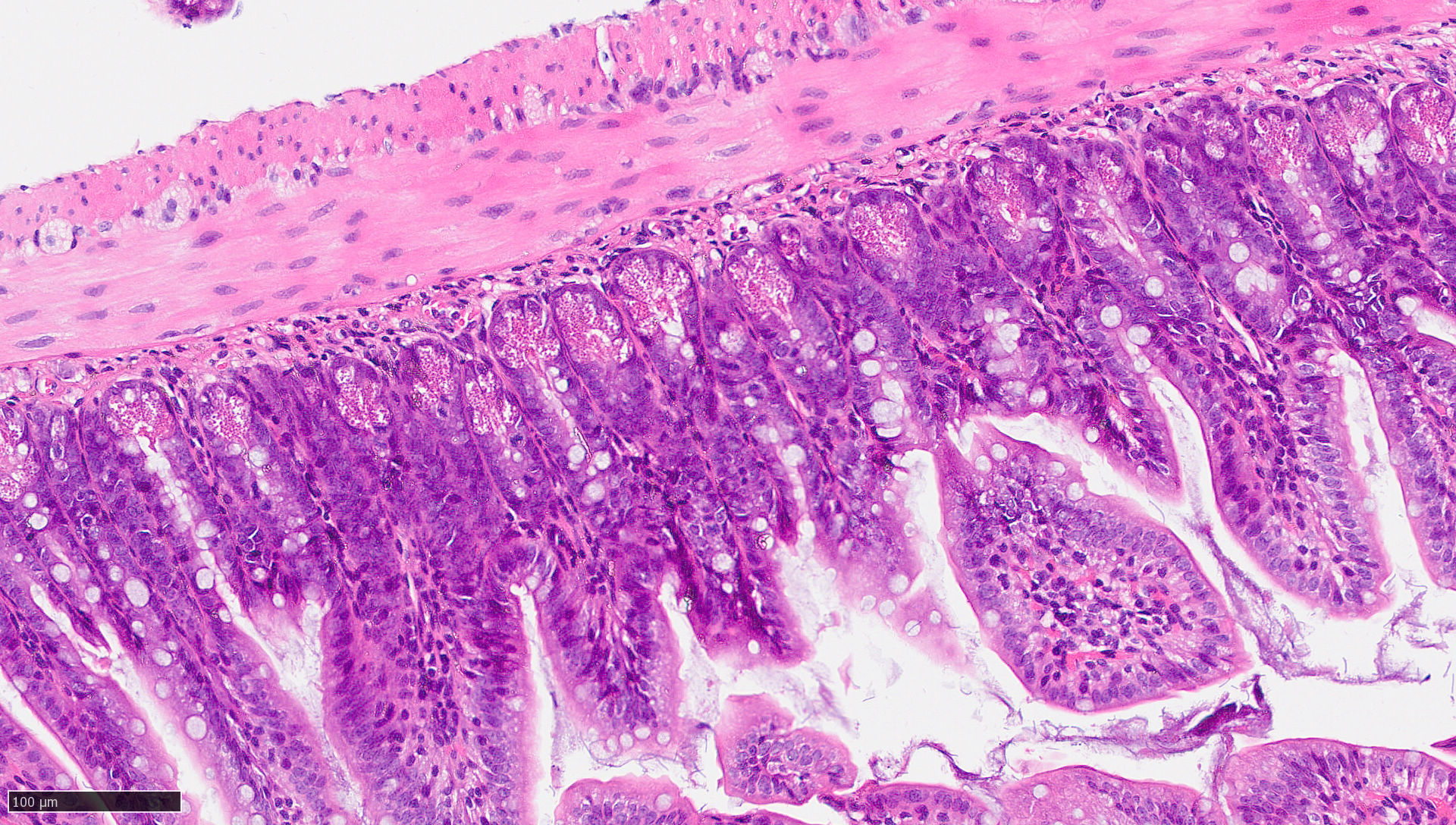

Supplement: Supplementary file 15 [file DataSheet_15.zip › μ£¬σæ╜σÉìμûçΣ╗╢σñ╣/Figure 1/Fig.1 Other files/full scans of Immunohistochemical staining of intestine/EA (2).jpg]

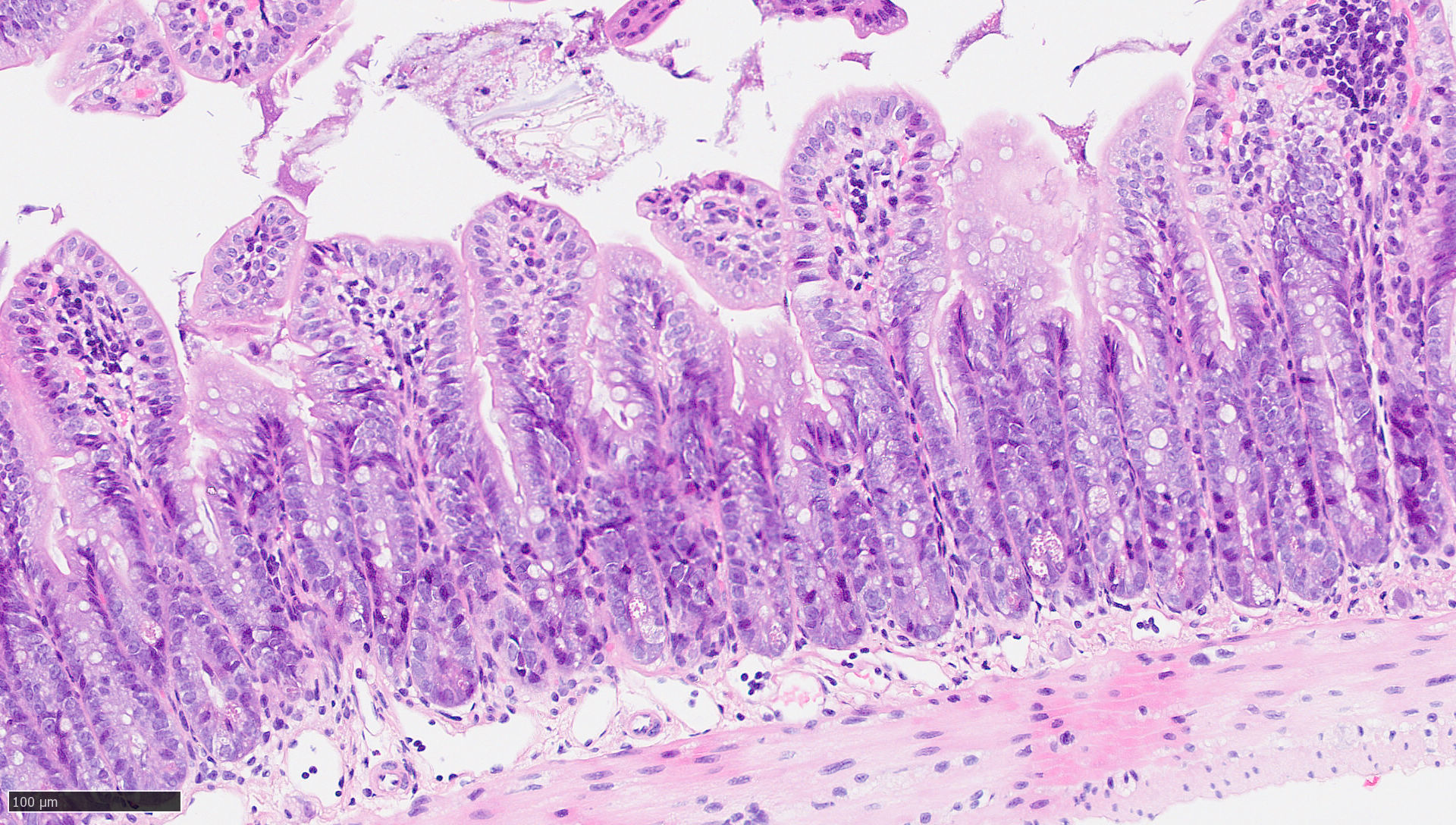

Supplement: Supplementary file 15 [file DataSheet_15.zip › μ£¬σæ╜σÉìμûçΣ╗╢σñ╣/Figure 1/Fig.1 Other files/full scans of Immunohistochemical staining of intestine/NEA (5).jpg]

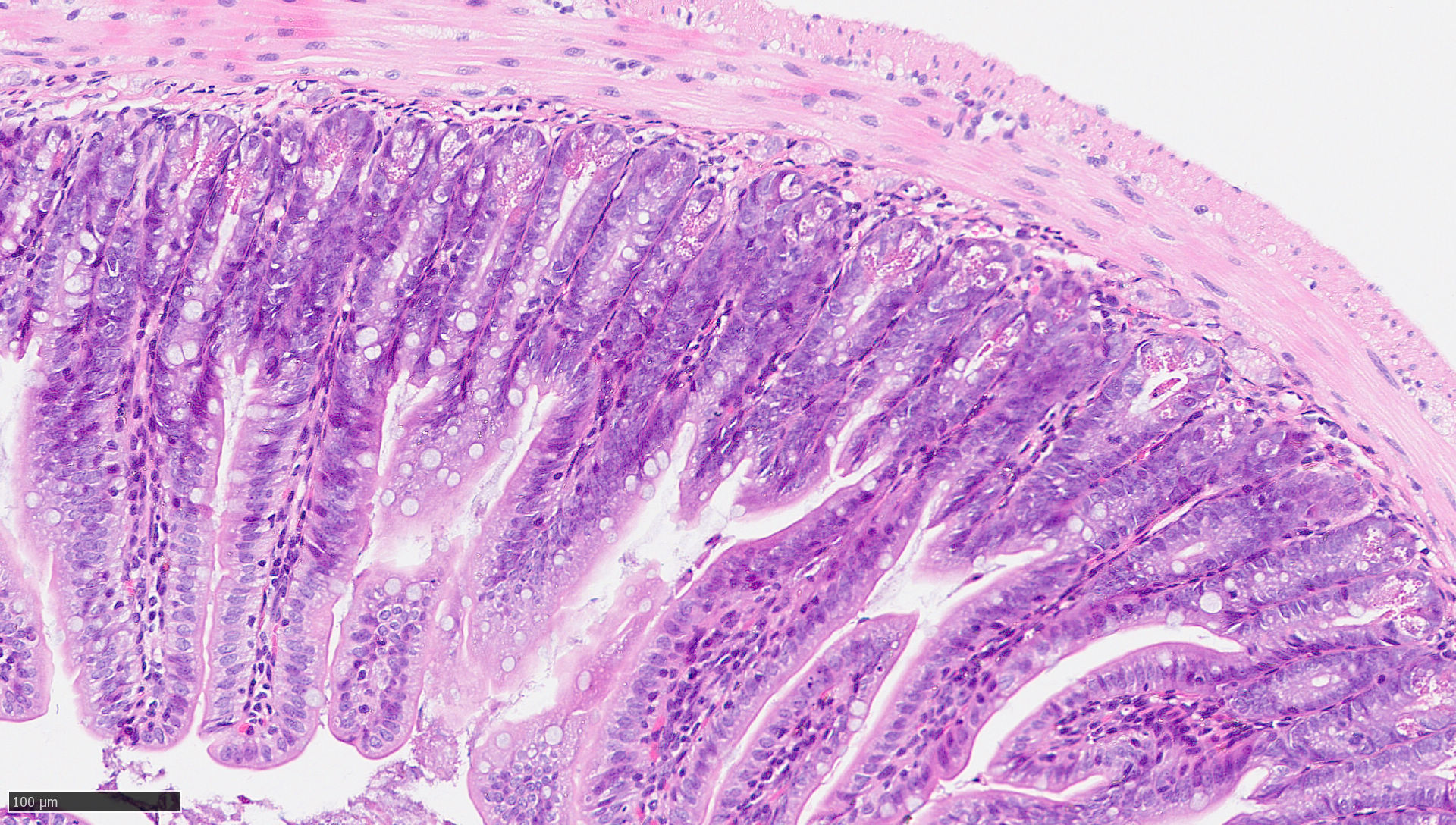

Supplement: Supplementary file 15 [file DataSheet_15.zip › μ£¬σæ╜σÉìμûçΣ╗╢σñ╣/Figure 1/Fig.1 Other files/full scans of Immunohistochemical staining of intestine/NEA (4).jpg]

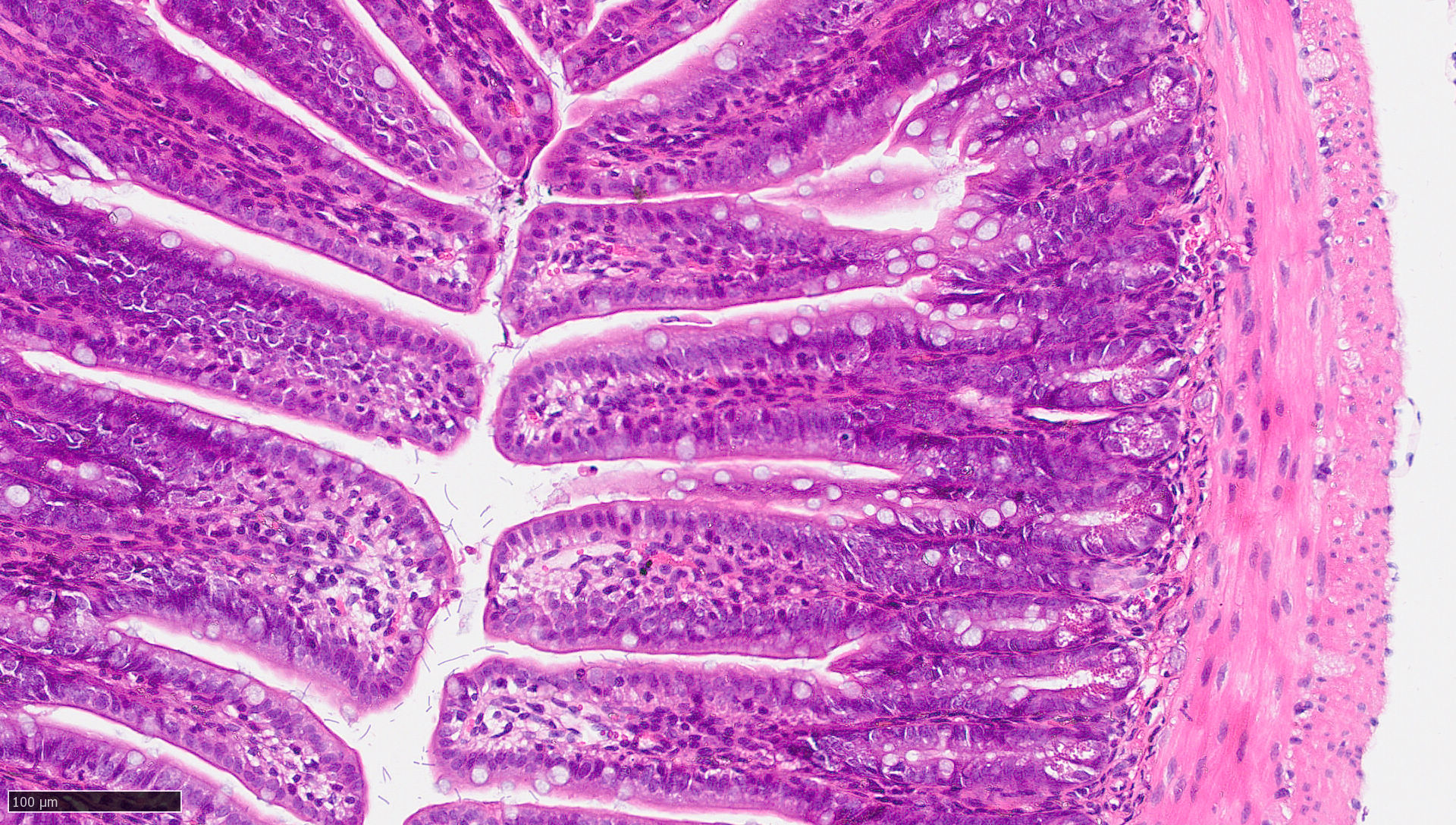

Supplement: Supplementary file 15 [file DataSheet_15.zip › μ£¬σæ╜σÉìμûçΣ╗╢σñ╣/Figure 1/Fig.1 Other files/full scans of Immunohistochemical staining of intestine/EA (3).jpg]

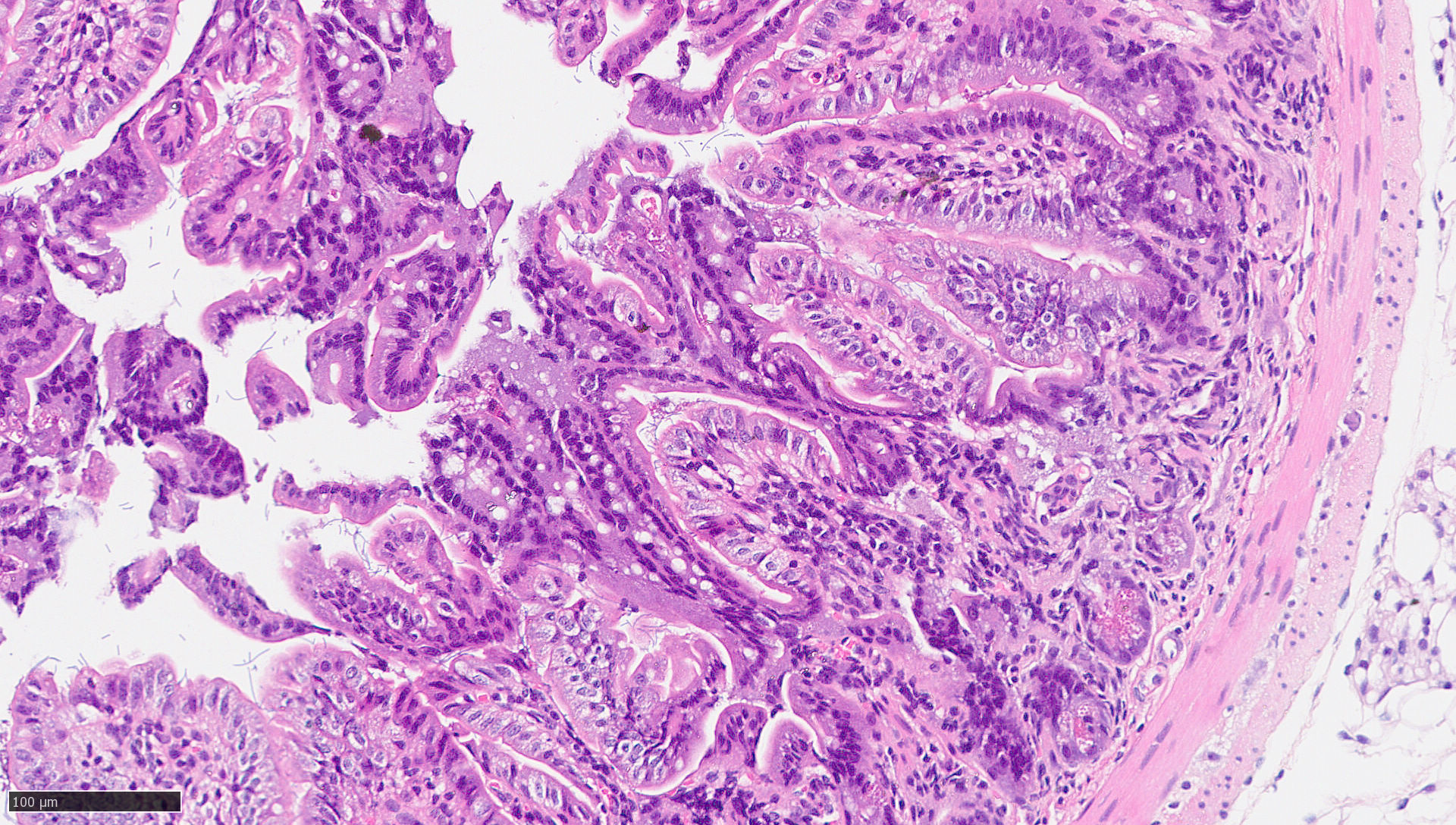

Supplement: Supplementary file 15 [file DataSheet_15.zip › μ£¬σæ╜σÉìμûçΣ╗╢σñ╣/Figure 1/Fig.1 Other files/full scans of Immunohistochemical staining of intestine/CON (1).jpg]
