# Supplementary material for: Mechanism of electro-acupuncture in alleviating intestinal injury in septic mice via polyamine-related M2-macrophage polarization
Source: Front Immunol. 2024 Apr 22;15:1373876. doi: 10.3389/fimmu.2024.1373876 (PMC11075497; doi:10.3389/fimmu.2024.1373876)
Supplement: Supplementary file 15 [file DataSheet_15.zip › μ£¬σæ╜σÉìμûçΣ╗╢σñ╣/Figure 1/Fig.1 Other files/WB Result.pptx]

## Slide 1
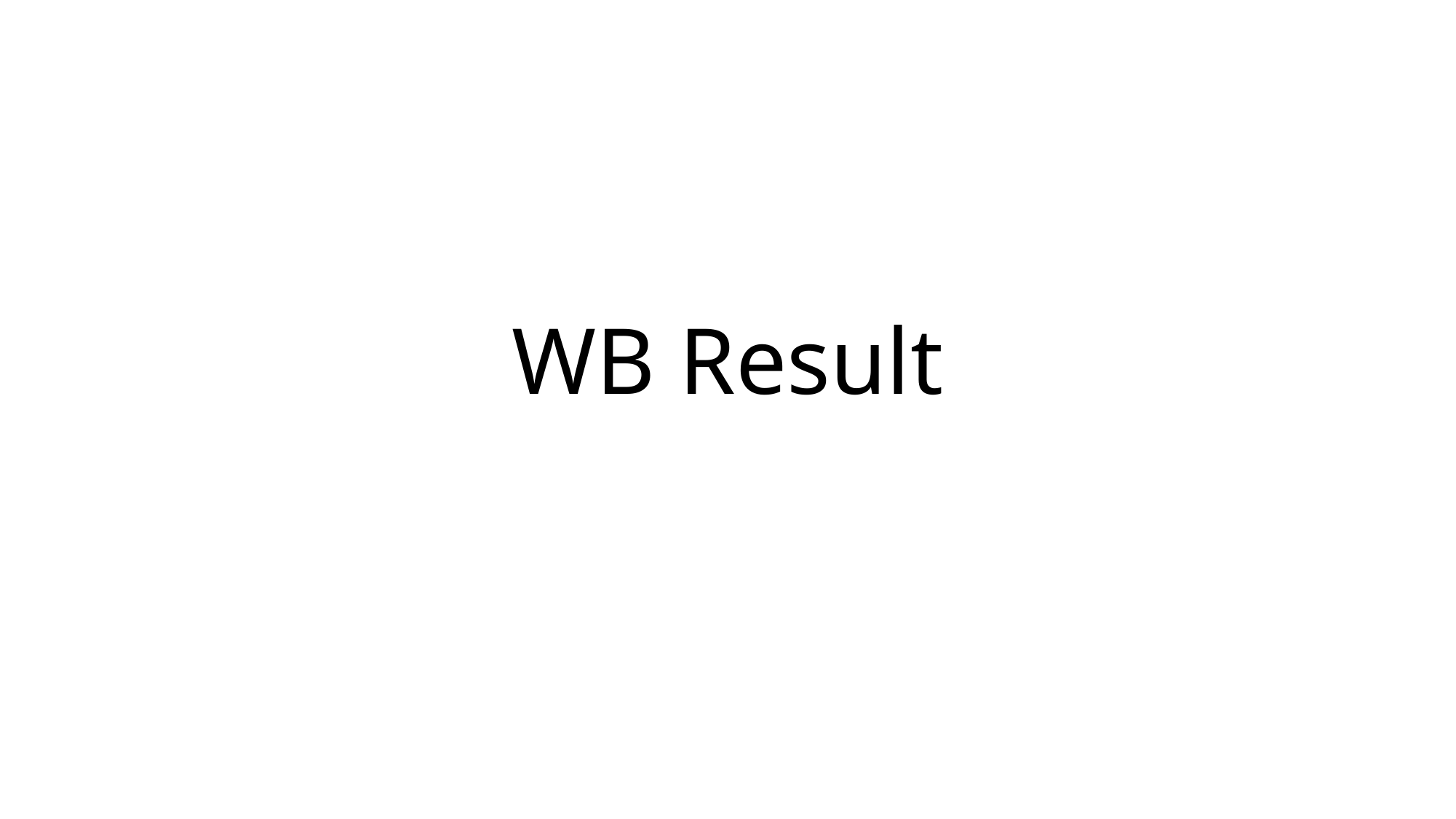

# WB Result

## Slide 2
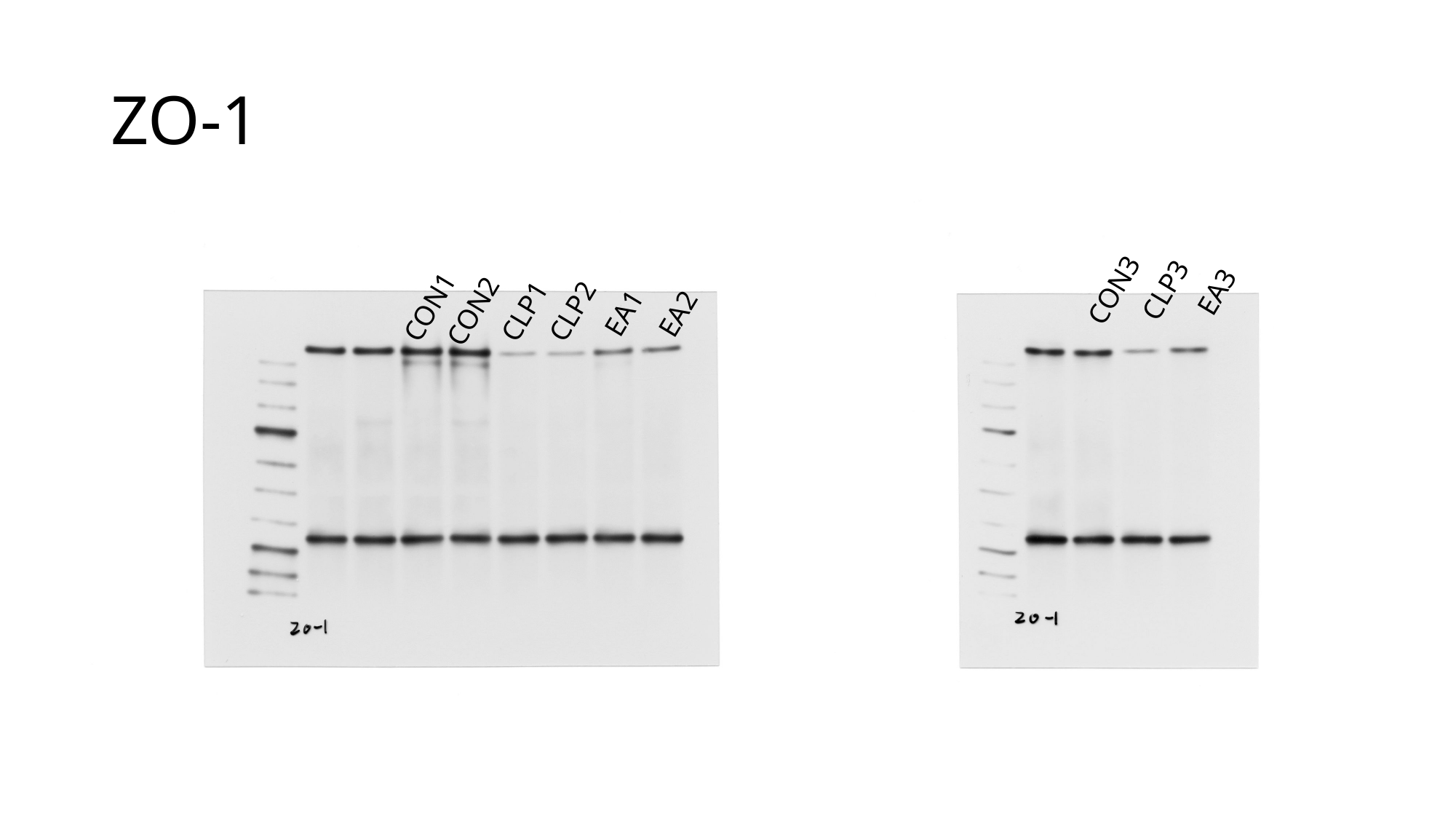

# ZO-1
CON3
CLP3
EA3
CON1
CON2
CLP1
CLP2
EA1
EA2

## Slide 3
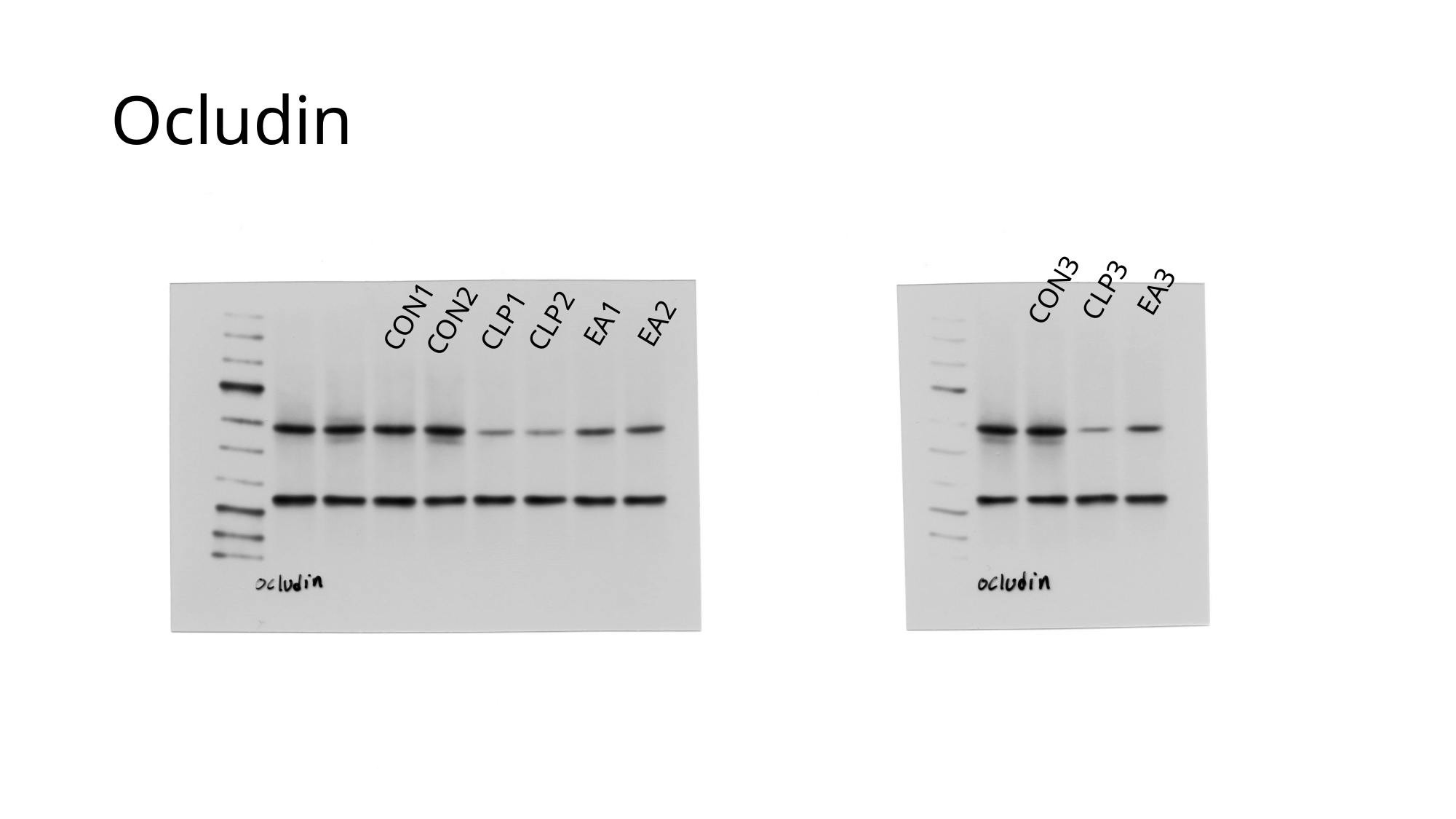

# Ocludin
CON3
CLP3
EA3
CON1
CON2
CLP1
CLP2
EA1
EA2

## Slide 4
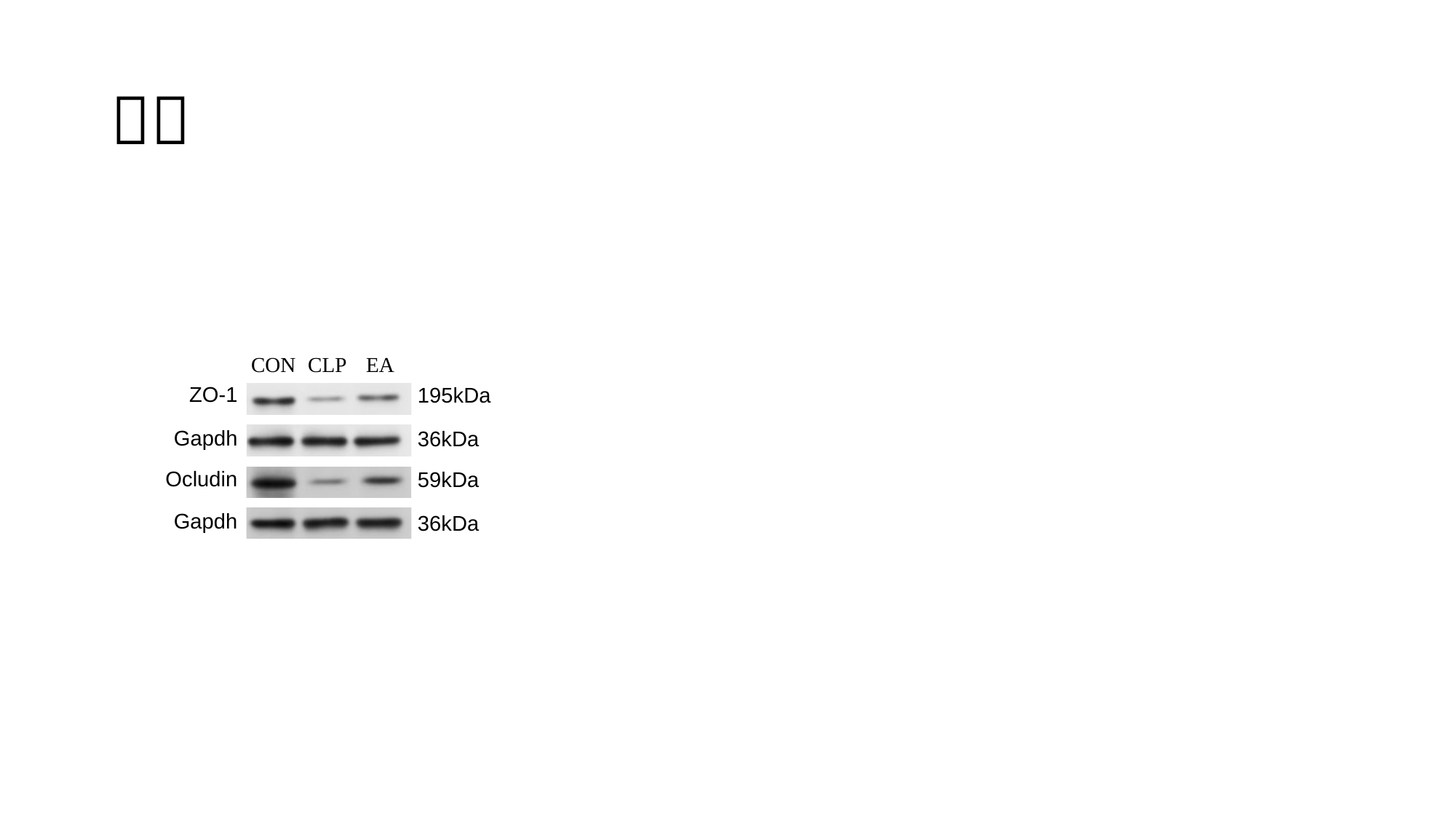

# 组图
CON
CLP
EA
| ZO-1 |
| --- |
| 195kDa |
| --- |
| Gapdh |
| --- |
| 36kDa |
| --- |
| Ocludin |
| --- |
| 59kDa |
| --- |
| Gapdh |
| --- |
| 36kDa |
| --- |
